# Estatística Cardiovascular – Brasil 2020

**DOI:** 10.36660/abc.20200812

**Published:** 2020-09-18

**Authors:** Gláucia Maria Moraes de Oliveira, Luisa Campos Caldeira Brant, Carisi Anne Polanczyk, Andreia Biolo, Bruno Ramos Nascimento, Deborah Carvalho Malta, Maria de Fatima Marinho de Souza, Gabriel Porto Soares, Gesner Francisco Xavier, M. Julia Machline-Carrion, Marcio Sommer Bittencourt, Octavio M. Pontes, Odilson Marcos Silvestre, Renato Azeredo Teixeira, Roney Orismar Sampaio, Thomaz A. Gaziano, Gregory A. Roth, Antonio Luiz Pinho Ribeiro

**Affiliations:** 1 Instituto do Coração Edson Saad Universidade Federal do Rio de Janeiro Rio de Janeiro RJ Brasil Instituto do Coração Edson Saad da Universidade Federal do Rio de Janeiro (UFRJ), Rio de Janeiro , RJ – Brasil; 2 Departamento de Clínica Médica Faculdade de Medicina Universidade Federal do Rio de Janeiro Rio de Janeiro RJ Brasil Disciplina de Cardiologia, Departamento de Clínica Médica da Faculdade de Medicina da Universidade Federal do Rio de Janeiro (UFRJ), Rio de Janeiro , RJ – Brasil; 3 Departamento de Clínica Médica Faculdade de Medicina Universidade Federal de Minas Gerais Belo Horizonte MG Brasil Departamento de Clínica Médica da Faculdade de Medicina da Universidade Federal de Minas Gerais (UFMG), Belo Horizonte , MG – Brasil; 4 Centro de Telessaúde Hospital das Clínicas Universidade Federal de Minas Gerais Belo Horizonte MG Brasil Serviço de Cardiologia e Cirurgia Cardiovascular e Centro de Telessaúde do Hospital das Clínicas da Universidade Federal de Minas Gerais (UFMG), Belo Horizonte , MG – Brasil; 5 Faculdade de Medicina Universidade Federal do Rio Grande do Sul Porto Alegre RS Brasil Faculdade de Medicina da Universidade Federal do Rio Grande do Sul (UFRS), Porto Alegre , RS – Brasil; 6 Serviço de Cardiologia Hospital Moinhos de Vento Porto Alegre RS Brasil Serviço de Cardiologia do Hospital Moinhos de Vento , Porto Alegre , RS – Brasil; 7 Hospital de Clínicas de Porto Alegre Porto Alegre RS Brasil Hospital de Clínicas de Porto Alegre (HCPA), Porto Alegre , RS – Brasil; 8 Programa de Pós-Graduação em Saúde Pública Universidade Federal de Minas Gerais Belo Horizonte MG Brasil Programa de Pós-Graduação em Saúde Pública da Universidade Federal de Minas Gerais (UFMG), Belo Horizonte , MG – Brasil; 9 Organização Vital Strategies Nova York EUA Organização Vital Strategies , Nova York – EUA; 10 Universidade de Vassouras Vassouras RJ Brasil Curso de Medicina da Universidade de Vassouras , Vassouras , RJ – Brasil; 11 Faculdade de Medicina Universidade Federal de Minas Gerais Belo Horizonte MG Brasil Biblioteca da Faculdade de Medicina Universidade Federal de Minas Gerais (UFMG), Belo Horizonte , MG – Brasil; 12 ePHealth Primary Care Solutions Santo Antônio SC Brasil ePHealth Primary Care Solutions , Santo Antônio , SC – Brasil; 13 Divisão de Clínica Médica Hospital Universitário Universidade de São Paulo São Paulo SP Brasil Divisão de Clínica Médica do Hospital Universitário da Universidade de São Paulo , São Paulo , SP – Brasil; 14 Faculdade Israelita de Ciências da Saúde Albert Einstein São Paulo SP Brasil Faculdade Israelita de Ciências da Saúde Albert Einstein , São Paulo , SP – Brasil; 15 Departamento de Neurociências e Ciências do Comportamento Faculdade de Medicina de Ribeirão Preto Universidade de São Paulo São Paulo SP Brasil Serviço de Neurologia Vascular e Emergências Neurológicas, Divisão de Neurologia, Departamento de Neurociências e Ciências do Comportamento , Faculdade de Medicina de Ribeirão Preto da Universidade de São Paulo (USP), São Paulo , SP – Brasil; 16 Universidade Federal do Acre Rio Branco AC Brasil Universidade Federal do Acre (UFAC), Rio Branco , AC – Brasil; 17 Departamento de Cardiopneumologia Faculdade de Medicina Universidade de São Paulo São Paulo SP Brasil Departamento de Cardiopneumologia da Faculdade de Medicina da Universidade de São Paulo (USP), São Paulo , SP – Brasil; 18 Programa de Pós-Graduação Faculdade de Medicina Universidade de São Paulo São Paulo SP Brasil Programa de Pós-Graduação da Faculdade de Medicina da Universidade de São Paulo (USP), São Paulo , SP – Brasil; 19 Hospital das Clínicas Faculdade de Medicina Universidade de São Paulo São Paulo SP Brasil Unidade Clínica de Cardiopatias Valvares do Instituto do Coração (Incor) do Hospital das Clínicas da Faculdade de Medicina da Universidade de São Paulo (HCFMUSP), São Paulo , SP – Brasil; 20 Brigham and Women’s Hospital Boston EUA Brigham and Women’s Hospital , Boston – EUA; 21 Department of Medicine Harvard Medical School Boston EUA Department of Medicine , Cardiovascular, Harvard Medical School , Boston – EUA; 22 Global Health and Health Metrics Sciences Institute for Health Metrics and Evaluation Washington EUA Global Health and Health Metrics Sciences at the Institute for Health Metrics and Evaluation (IHME), Washington – EUA; 23 Division of Cardiology University of Washington School of Medicine Washington EUA Division of Cardiology at the University of Washington School of Medicine , Washington – EUA

**Keywords:** Doenças Cardiovasculares, Doença das Coronárias, Cardiomiopatias. Insuficiência Cardíaca, Doenças das Valvas Cardíacas, Fibrilação Atrial, Flutter Atrial, Estatística, Brasil

## Sobre este Documento

**Table:** 

Abreviaturas usadas nesta Introdução	
CID	Classificação Estatística Internacional de Doenças e Problemas Relacionados à Saúde
DALYs	Anos de vida perdidos ajustados por incapacidade (do inglês, *Disability-Adjusted Life-Year* )
DCV	Doença Cardiovascular
GBD	*Global Burden of Disease*
IBGE	Instituto Brasileiro de Geografia e Estatística
IHME	*Institute for Health Metrics and Evaluation *
IPCA	Índice de Preços ao Consumidor Amplo
PIB	Produto Interno Bruto
PNS	Pesquisa Nacional de Saúde
PPC	Paridade do Poder de Compra
SBC	Sociedade Brasileira de Cardiologia
SIH	Sistema de Informações Hospitalares
SIM	Sistema de Informações sobre Mortalidade
SUS	Sistema Único de Saúde
UF	Unidade Federativa
YLDs	Anos vividos com incapacidade (do inglês, *Years Lived with Disability* )
YLLs	Anos potenciais de vida perdidos (do inglês, *Years of Life Lost* )

 O Brasil é um país continental de extrema diversidade quanto a clima e condições ambientais, densidade populacional, desenvolvimento econômico e características raciais e culturais. Uma das dez economias mais ricas do mundo, o Brasil é também um dos países mais desiguais: segundo o Banco Mundial, está entre os dez países de maior Índice de Gini, uma medida da desigualdade de distribuição de renda (https://data.worldbank.org/indicator/SI.POV.GINI). O Brasil possui o SUS, um dos maiores sistemas públicos de saúde com cobertura universal, que abrange toda a população brasileira, estimada em 210 milhões de habitantes em 2019 (https://www.ibge.gov.br/estatisticas/sociais/populacao.html), ano em que o SUS foi o único sistema de saúde para 76% daquela população (http://www.ans.gov.br). Na verdade, o SUS coexiste com o sistema privado de saúde, que inclui planos e seguros de saúde, além de profissionais de saúde privados. Estabelecido pela Constituição Brasileira de 1988, a implementação e a expansão do SUS permitiram ao Brasil abordar rapidamente as mudanças nas necessidades de saúde da população, com dramática sofisticação da cobertura dos serviços de saúde e aumento da expectativa de vida em apenas três décadas. ^1 , 2^ O Programa de Saúde da Família, lançado em 1994, é uma importante iniciativa da estratégia nacional para reduzir a mortalidade por DCV com base na atenção primária à saúde, cobrindo quase 123 milhões de indivíduos (63% da população brasileira) em 2015. ^3^ Entretanto, a despeito dos sucessos, a análise dos cenários futuros sugere a necessidade urgente de abordar desigualdades geográficas persistentes, financiamento insuficiente, além de questões relacionadas ao acesso ao cuidado e à sua qualidade. ^1 , 2^

 A DCV tem sido a principal causa de mortalidade desde a década de 60, sendo responsável por uma substancial carga de doença no Brasil. ^2 , 4^ Uma quantidade considerável de dados relevantes para a saúde cardiovascular é disponibilizada tanto pelo governo, através de vigilância em saúde e bancos de dados administrativos, quanto pelos estudos epidemiológicos. ^5 - 10^ Entretanto, dados nacionais representativos e confiáveis sobre muitos comportamentos relacionados a saúde e fatores de risco cardiovascular, assim como sobre morbidade, avaliados nos setores público e privado permanecem escassos. ^2^ Nos últimos anos, o Projeto GBD, conduzido pelo IHME da Universidade de Washington, começou a trabalhar com a rede GBD Brasil para fornecer estimativas subnacionais de carga de doença para as UF do Brasil, incluindo as causas cardiovasculares. ^11 - 14^

 Este relatório, **Estatística Cardiovascular – Brasil 2020** , incorpora estatísticas oficiais fornecidas pelo Ministério da Saúde brasileiro e outras agências governamentais, além de dados gerados por outras fontes e estudos científicos sobre doença cardíaca, acidente vascular cerebral e outras DCV, inclusive dados do GBD/IHME. Este projeto tem por objetivo monitorar e avaliar continuamente as fontes de dados sobre doença cardíaca e acidente vascular cerebral no Brasil para fornecer a informação mais atualizada sobre a epidemiologia dessas doenças para a sociedade brasileira anualmente. Esta iniciativa é baseada na metodologia *Heart Disease & Stroke Statistics Update* ^15^ da *American Heart Association* , contando com o suporte da SBC, da rede GBD Brasil e de um comitê internacional. O documento **Estatística Cardiovascular – Brasil 2020** resulta do esforço de clínicos e cientistas dedicados e voluntários, profissionais do governo comprometidos e excepcionais membros da SBC, sem os quais a publicação desta valiosa fonte não seria possível. Este documento foi projetado para ter grande utilidade para pesquisadores, clínicos, pacientes, formuladores de políticas de saúde, profissionais da mídia, o público em geral e todos aqueles que buscam dados nacionais abrangentes sobre doença cardíaca e acidente vascular cerebral. A primeira edição ficou restrita a um número limitado de condições clínicas, listadas a seguir:

1. Doença Cardiovascular Total
2. Doença Cerebrovascular
3. Doença Arterial Coronariana, Síndrome Coronariana Aguda e Angina Pectoris
4. Cardiomiopatia e Insuficiência Cardíaca
5. Doenças Valvares, incluindo Cardiopatia Reumática
6. Fibrilação Atrial

Todos os capítulos estão padronizados e apresentam uma estrutura em comum, que inclui pelo menos os seguintes tópicos: Prevalência, Incidência, Mortalidade, Carga de Doença, Utilização e Custo da Atenção à Saúde, Pesquisa Futura. Nas próximas edições, pretendemos ampliar a cobertura das condições cardíacas clínicas e ainda dos fatores de risco cardiovascular, dos hábitos de vida, da qualidade do cuidado e de outros aspectos relevantes para o estudo das DCV. 

 A ênfase do documento está nos dados epidemiológicos atualizados. Não foca nos mecanismos fisiopatológicos nem nos méritos de tratamentos clínicos específicos, sequer faz recomendações terapêuticas. Ademais, não é um *position paper* nem uma revisão abrangente, mas procura apresentar as melhores e mais novas métricas relacionadas à saúde sobre as estatísticas das DCV para a população brasileira. Importante notar ainda que este documento não tem a pretensão de cobrir outros países e regiões, ficando restrito ao Brasil e às suas regiões e UF. 

 Para o presente documento, foram utilizadas principalmente três fontes de dados: (a) sistemas de informação de mortalidade e saúde no Brasil, disponibilizados pelo governo; (b) estimativas do GBD 2017; (c) revisão sistemática da literatura com ênfase nas publicações dos últimos dez anos. As métricas das diversas fontes não foram idênticas, podendo as diferenças estar relacionadas a diferentes períodos de tempo, localizações, faixas etárias ou a outros aspectos metodológicos (Malta, 2020, ABC Cardiol, *in press* ). Por isso não evitamos citar métricas discordantes e, em geral, mencionamos ou discutimos as possíveis razões para tais diferenças. Como muitos estudos cobrem um longo período de tempo e a expectativa de vida no Brasil aumentou nas últimas décadas, decidimos usar taxas padronizadas por idade, i.e., uma média ponderada de taxas específicas para idade por 100 mil pessoas, onde os pesos são as proporções de pessoas nos grupos etários correspondentes de uma população padrão. A padronização por idade do GBD utiliza um padrão global de idade, embora outras fontes possam ter usado diferentes populações de referência. Para a maioria dos estudos, raça/cor da pele foi usada de acordo com a definição do IBGE, i.e., branca, preta, parda, amarela (oriental) ou indígena (nativa). 

 A seguir, apresentamos um breve resumo das nossas fontes de dados e da metodologia usada para avaliar a utilização do cuidado de saúde. 

##  Sistemas de Informação de Mortalidade e de Saúde no Brasil 

 Na presente versão da **Estatística Cardiovascular – Brasil 2020** , as principais fontes de dados brasileiros são os sistemas de informação de saúde brasileiros, que compreendem o SIM e o SIH, as pesquisas de saúde periódicas, como a PNS, e as estimativas populacionais oficiais, especificados a seguir:

- Sistema de Informação sobre Mortalidade: O SIM foi criado em 1975 pelo Ministério da Saúde brasileiro, sendo responsável por coletar, armazenar, gerenciar e divulgar dados nacionais de mortalidade. Esse sistema de informação em saúde representou um grande avanço na vigilância epidemiológica do país, pois sua principal atribuição é registrar todas as mortes ocorridas no território brasileiro. O Ministério da Saúde implementou um modelo de declaração de óbito padrão para coletar informação sobre morte, que utiliza a CID para codificar as causas de morte. Além disso, um fluxo para coletar, processar e distribuir a informação sobre morte foi implementado em todos os 5.570 municípios do país. ^16 , 17^ A qualidade da estatística sobre causas de morte no Brasil, baixa no início dos anos 2000, em especial em algumas partes do país, melhorou significativamente nas duas últimas décadas. ^18^ Por conhecer a heterogeneidade desses indicadores no Brasil e buscando uma estimativa da informação mais próxima da situação real, o relatório E **statística Cardiovascular – Brasil 2020** tratou os dados, realizando a correção para subnotificação e a redistribuição das causas de morte mal definidas. Mais detalhes podem ser obtidos no artigo de Malta *et al.* ( *in press* ).
- Sistema de Informação Hospitalar: O objetivo da base de dados do SIH é registrar todas as hospitalizações financiadas pelo SUS. O SIH-SUS armazena dados sobre as hospitalizações em nível municipal através da Autorização de Internação Hospitalar, que contém informação sobre as doenças que levaram a hospitalização (usando a CID-10), o tempo de permanência, os procedimentos e os custos. ^19^ A informação do SIH-SUS permite o desenvolvimento de metodologias e a definição de indicadores para identificar disparidades geográficas relacionadas aos recursos hospitalares. ^20^
- Pesquisa Nacional de Saúde: Embora não esteja no escopo do documento deste ano descrever a estatística para fatores de risco cardiovascular, alguns capítulos citam a métrica para alguns fatores de risco no contexto da doença especificada. Nesses casos, deu-se preferência à PNS, que é um inquérito de base domiciliar, representativo do Brasil, de suas grandes regiões e UF, regiões metropolitanas, capitais e de outros municípios em cada UF. A amostragem da PNS 2013 foi composta por 64.348 domicílios. A pesquisa foi conduzida pelo IBGE em parceria com o Ministério da Saúde, tendo incluído a maioria dos tópicos de saúde, como doenças não transmissíveis, função renal, idosos, mulheres, crianças, utilização dos serviços de saúde, desigualdades em saúde, características antropométricas, exames laboratoriais, além de aferição de pressão arterial. ^21^ Os dados da PNS são usados pelo GBD em suas estimativas para o Brasil.
- Para as estimativas populacionais, utilizaram-se no denominador as estimativas populacionais mais atualizadas geradas pelo IBGE (www.ibge.gov.br). Para as hospitalizações e análises de custo, utilizou-se a população residente estimada para o Tribunal de Contas da União anualmente, de 2008 a 2018.

## GBD 2017

 O Estudo GBD (http://www.healthdata.org/gbd) é o mais abrangente estudo epidemiológico observacional de âmbito mundial até o momento. Descreve mortalidade e morbidade decorrentes das principais doenças, injúrias e fatores de risco em níveis global, nacional e regional. O exame das tendências a partir de 1990 até o presente, assim como as comparações entre populações, permitem compreender os desafios em saúde enfrentados pelas pessoas em todo o mundo no século 21. O GBD 2017 é o último conjunto de dados disponibilizado publicamente. ^22 - 25^ A rede GBD Brasil tem colaborado com o IHME, que lidera o projeto em âmbito mundial, para a identificação e a provisão de conjuntos de dados, a revisão de modelos e estimativas, bem como a validação e a publicação de resultados para o Brasil. ^13 , 14^ Detalhes de como as estimativas são calculadas podem ser obtidos nas publicações de base do Estudo GBD ^22 - 25^ e no *website* do IHME (http://www.healthdata.org/acting-data/what-we-measure-and-why). As principais estimativas usadas neste documento estão resumidas abaixo:

- Estimativas de mortes e de causas de morte. A principal fonte de informação é o SIM, uma base de dados do Ministério da Saúde, ajustada para outras fontes nacionais e internacionais. O IHME corrigiu a subnotificação de mortes e as mortes com “código *garbage* ” através da utilização de metodologia com algoritmos previamente publicada, ^26^ atualizada nas versões mais recentes do estudo (http://www.healthdata.org/acting-data/determining-causes-death-how-we-reclassify-miscoded-deaths).
- Os YLLs são os anos perdidos em razão de mortalidade prematura, sendo calculados subtraindo-se a idade à época da morte da maior expectativa de vida possível para uma pessoa. Por exemplo, se a maior expectativa de vida para um homem em um certo país for de 75 anos, e se um homem morre de câncer aos 65 nesse país, tem-se 10 anos potenciais de vida perdidos para o câncer.
- Os YLDs também podem ser descritos como os anos vividos com saúde inferior à ideal. Estão aqui incluídas condições como influenza, que pode durar apenas uns poucos dias, ou epilepsia, que pode durar uma vida inteira. Os YLDs podem ser calculados ao se multiplicar a prevalência da condição pelo peso da incapacidade por ela gerada. Os pesos da incapacidade refletem a gravidade de diferentes condições e são desenvolvidos através de pesquisas com o público em geral.
- Os DALYs são uma métrica universal que permite que pesquisadores e formuladores de políticas comparem populações e condições de saúde muito diferentes ao longo do tempo. Os DALYs correspondem à soma dos YLLs e YLDs, sendo 1 DALY igual a 1 ano de vida saudável perdido. Esse índice permite que se estime o número total de anos perdidos devido a causas específicas e fatores de risco em níveis global, nacional e regional.

### Revisão Sistemática da Literatura

 Os descritores para a elaboração das estratégias de busca foram selecionados no MeSH e no DeCS, os vocabulários controlados da MEDLINE e da LILACS, respectivamente. O plano da Embase foi desenhado com descritores Emtree em associação com MeSH. Além disso, termos livres foram usados, i.e., palavras-chave significativas e seus sinônimos, variações ortográficas e acrônimos essenciais para a busca no domínio pesquisado, mas que não são descritores controlados (ou não estão na lista de sinônimo desses descritores). É importante lembrar que, para manter a uniformidade, os mesmos descritores foram usados em todas as estratégias de busca. Entretanto, as estratégias foram customizadas conforme as especificidades de cada base de dados. Vale ainda lembrar que o grupo de termos relacionados a “Brasil” foi em geral utilizado em todos os campos de pesquisa (assunto, autor, título, afiliação institucional, nome do periódico, etc.). 

 As bases selecionadas para busca foram a MEDLINE através da PubMed, Embase, LILACS, CINAHL, *Cochrane Library Scopus* e *Web of Science* . Os seguintes filtros e limites da pesquisa bibliográfica foram utilizados: período de publicação (2004-2019); línguas: português, inglês e espanhol; tipo de estudo/publicação: Revisão, Meta-Análise, Ensaio Clínico, Ensaio Randomizado Controlado, Estudo Comparativo, Diretriz de Prática, Diretriz, Revisão Sistemática, Estudo de Avaliação, Publicação Governamental e Estudo Multicêntrico. Todas as referências foram organizadas usando-se o *EndNote Web* . A partir da busca, os artigos foram incluídos se os estudos fossem de base populacional ou comunitária. Deu-se preferência aos estudos de âmbito nacional ou estadual. Os estudos conduzidos em serviços de saúde ou hospitais foram incluídos caso fossem multicêntricos e possuíssem tamanho amostral adequado (> 200 participantes foi o ponto de corte sugerido). Além dos artigos identificados na busca sistemática, os autores puderam incluir outros encontrados nas referências dos artigos buscados ou outros de que tivessem conhecimento em suas áreas de especialidade, caso os estudos atendessem aos critérios acima mencionados. Por fim, a decisão de quais estudos incluir em cada capítulo coube principalmente aos especialistas designados para o tema em questão. 

### Utilização da Atenção à Saúde

 Os estudos sobre o custo da atenção à saúde apresentam grande variabilidade metodológica e precisam ser interpretados com cautela. No presente documento, a maior parte dos dados sobre custo foi obtida das tabelas de reembolso do Sistema Público de Saúde de 2008 a 2018. Durante esse período, o reajuste pela inflação não foi realizado de maneira regular nem homogênea nos grupos e procedimentos de DCV. A taxa de inflação brasileira (baseada no IPCA) de 2008 a 2018 foi 76,3%, enquanto a inflação média para os procedimentos cardiovasculares foi 43,5%. Para alguns códigos de procedimento, o reajuste foi mínimo, como para a implantação de stent coronariano, cujo reajuste foi de 8,7%. Para outros códigos, o reajuste ficou acima da inflação, como para o tratamento de arritmias (83,4%). 

 Para minimizar o viés na notificação e na interpretação dos dados de custo, aplicou-se uma abordagem sistemática em todos os capítulos. Nas análises de custo geral, foram utilizadas as unidades monetárias originais [ *Reais* (R$) ou dólares americanos (US$) em um determinado ano] e dólares internacionais. Os dólares internacionais foram convertidos em PPC ajustados para US$ 2018 (Int$ 2018), usando-se o conversor de custo do Centro *Campbell and Cochrane Economics Methods Group Evidence for Policy and Practice Information and Coordination* (https://eppi.ioe.ac.uk/costconversion/default.aspx ) . Nesse método, aplicou-se uma abordagem em duas etapas. Na primeira, ajustou-se a estimativa original de custo no preço-ano original para o preço-ano alvo, usando-se o índice de deflação do PIB (valores PIBD). Na segunda, houve conversão dessa estimativa ajustada da moeda original para a moeda alvo, usando-se as taxas de conversão baseadas em PPC para o PIB (valores PPC). ^27^ Para estudos econômicos originais, quando o ano-base da moeda não foi informado ou não pôde ser inferido a partir do manuscrito (p. e., coleta de dados do ano passado), recomendou-se adotar o ano anterior ao da publicação do manuscrito. 

## 1. DOENÇA CARDIOVASCULAR TOTAL

### CID-9 390 a 459; CID-10 I00 a I99.

Ver Tabelas 1-1 até 1-9 e Figuras 1-1 até 1-16

**Table:** 

Abreviaturas usadas no Capítulo 1	
AHA	*American Heart Association*
AVC	Acidente Vascular Cerebral
CID	Classificação Estatística Internacional de Doenças e Problemas Relacionados à Saúde
CRVM	Cirurgia de Revascularização do Miocárdio
DALYs	Anos de vida perdidos ajustados por incapacidade (do inglês, *Disability-Adjusted Life-Year* )
DATASUS	Departamento de Informática do Sistema Único de Saúde
DCNT	Doenças Crônicas Não Transmissíveis
DCV	Doença Cardiovascular
DIC	Doença Isquêmica do Coração
ELSA-Brasil	Estudo Longitudinal da Saúde do Adulto
ELSI-Brasil	Estudo Longitudinal da Saúde dos Idosos Brasileiros
GBD	*Global Burden of Disease*
IAM	Infarto Agudo do Miocárdio
IBGE	Instituto Brasileiro de Geografia e Estatística
IC	Intervalo de Confiança
IDH	Índice de Desenvolvimento Humano
IDHm	Índice de Desenvolvimento Humano Municipal
II	Intervalo de Incerteza
OR	*Odds Ratio*
PIB	Produto Interno Bruto
PSF	Programa Saúde da Família
RAP	Risco Atribuível na População
RR	Risco Relativo
SCA	Síndrome Coronariana Aguda
SDI	Índice Sociodemográfico (do inglês, *Sociodemographic Index* )
SIDRA	Sistema IBGE de Recuperação Automática
SIH	Sistema de Informações Hospitalares
SIM	Sistema de Informações sobre Mortalidade
SNS	Sistema Nacional de Saúde
SUS	Sistema Único de Saúde
UF	Unidade Federativa

### Panorama

- As DCNT constituem o principal grupo de causa de morte em todo o mundo, sendo responsáveis por mortes prematuras, perda de qualidade de vida, além de impactos adversos econômicos e sociais. As DCNT são responsáveis por cerca de 70% das mortes globais, equivalendo a mais de 38 milhões de mortes por ano, excedendo significativamente as mortes por causas externas e por doenças infecciosas. ^28 - 31^ Cerca de 45% de todas as mortes por DCNT no mundo, mais de 17 milhões, são causadas por DCV. O mesmo ocorre no Brasil, onde 72% das mortes resultam de DCNT, sendo 30% devidas a DCV, 16% a neoplasias e 6% a doenças respiratórias. ^32 - 34^
- A definição de DCV pode variar de acordo com o estudo, desde a inclusão de todas as doenças listadas no Capítulo IX da CID-10 até o simples agrupamento das 3 principais causas (DIC, AVC e insuficiência cardíaca). Para o GBD, a definição de DCV total engloba 10 causas: cardiopatia reumática, DIC, doença cerebrovascular, cardiopatia hipertensiva, cardiomiopatia, miocardite, fibrilação e *flutter* atrial, aneurisma aórtico, doença vascular periférica e endocardite. ^35^
- As DCV eram a principal causa de morte no Brasil em 1990 e 2017 (Figura 1-1). De acordo com as estimativas do Estudo GBD 2017, entre as DCV, a DIC era a causa número 1 de morte no país, seguida por AVC, em 1990 e 2017 (Figura 1-2). Na verdade, em 2017, a DIC foi a principal causa de morte em todas as UF brasileiras, embora, em 1990, o AVC ainda fosse a causa de morte número 1 nos estados de Alagoas e Sergipe (Figuras 1-3 e 1-4).

### Prevalência

- Gonçalves *et al.* publicaram em 2019 um estudo transversal que analisou informação da Pesquisa Nacional de Saúde conduzida em 2013 em uma amostra de 60.202 adultos com mais de 18 anos, estratificados por sexo e 6 grupos etários, usando um modelo de regressão logística binário e hierárquico. O diagnóstico autorreferido de doença cardíaca no Brasil foi de 4,2% (IC 95%: 4,0-4,3 ) e associado com as seguintes características: sexo feminino (OR = 1,1; IC 95%: 1,1-1,1), indivíduos de 65 anos ou mais, hipertensão (OR = 2,4; IC 95%: 2,2-2,7), elevação de colesterol (OR = 1,6; IC 95%: 1,5-1,8), sobrepeso (OR = 1,5; IC 95%: 1,4-1,8) ou obesidade (OR = 2,0; IC 95%: 1,7-2,2), sedentarismo (OR = 1,5; IC 95%: 1,02-2,1) e tabagismo (OR = 1,2; IC 95%: 1,03-1,3). ^36^
- No estudo ELSA-Brasil, uma coorte que incluiu 15.105 funcionários públicos de 6 universidades ou institutos de pesquisa (54% mulheres, 35-74 anos, com avaliação basal entre 2008 e 2010), a prevalência autorreferida foi a seguinte: DIC, 4,7% (homens=5,7%, mulheres=4,0%); insuficiência cardíaca, 1,5% (homens=1,9%, mulheres=1,5%); AVC, 1,3% para ambos os sexos; febre reumática, 2,9% (homens=2,2%, mulheres=3,4%); e doença de Chagas, 0,4% para ambos os sexos. ^37^
- A prevalência de DCV aumenta significativamente com a idade. Em um estudo longitudinal com idosos a partir dos 60 anos, do estado de São Paulo, em 2000, 2006 e 2010, a prevalência de DCV foi definida como resposta positiva à pergunta: “Algum médico ou enfermeiro já lhe disse que você teve um ataque cardíaco, doença isquêmica do coração, angina, doença congestiva ou outros problemas cardíacos?” A prevalência de DCV foi de 17,9%, 22,2% e 22,9% em 2000, 2006 e 2010, respectivamente. A presença de DCV foi associada a idade mais avançada, história de tabagismo, presença de diabetes e hipertensão. ^38^
- De acordo com o Estudo GBD 2017, a prevalência padronizada por idade de DCV no Brasil em 1990 foi de 6.290 (II 95%, 6048-6549) por 100 mil habitantes e, em 2017, de 6.025 (II 95%, 5.786-6.275) por 100 mil habitantes, acometendo 6% da população com idade ≥ 20 anos, havendo leve redução de 4,2% (II 95%, -3,2 a -5,1) de 1990 a 2017. Os homens apresentaram maior prevalência padronizada por idade do que as mulheres de 1990 a 2017 (Figuras 1-5 e 1-6), embora a variação percentual tenha sido maior para os homens -5,5 (-4,2; -6,7) do que para as mulheres -2,4 (-1,3; -3,4) no período (Figura 1-6). Considerando-se o número total em 2017, 13.702.303 indivíduos (II 95%, 13.110.682-14.281.540) apresentaram DCV prevalente no Brasil, 6.784.523 homens (II 95%, 6.517.523-7.167.162) e 6.917.779 mulheres (6.616.359-7.220.572) (Tabela 1-1). ^39^
- O Estudo GBD 2017 revela que a prevalência padronizada por idade de DCV diminuiu desigualmente nas UF, sendo a redução maior nas regiões Sudeste e Sul, em particular nos estados do Espírito Santo, Rio de Janeiro, Santa Catarina e Rio Grande do Sul (Tabela 1-1), que estão entre os mais desenvolvidos do país.

### Incidência

- De acordo com o Estudo GBD 2017, a taxa de incidência padronizada por idade de DCV no Brasil em 2017 foi de 687,5 (II 95%, 663,4-712,4) casos por 100 mil habitantes, menor do que em 1990, quando havia 755,6 (II 95%, 731,6-783) casos por 100 mil habitantes (Tabela 1-2). Importante notar que, em toda a série temporal, as taxas de incidência do GBD para DCV total podem estar subestimadas em razão dos critérios de inclusão e exclusão dos modelos para cada doença específica incluída. A incidência de DCV total é o agregado das incidências de todas as doenças contidas no grupo. ^39^
- A UF com a maior taxa de incidência em 2017 foi o estado do Rio de Janeiro, com 709 (II 95%, 683,9-734,5) casos por 100 mil habitantes, e a de menor taxa de incidência em 2017 foi o estado do Rio Grande do Sul, com 646,6 (II 95%, 621,9-674,4) casos por 100 mil habitantes (Tabela 1-2).

### Mortalidade

- No Brasil, Mansur e Favarato relataram que a taxa de mortalidade por DCV padronizada por idade diminuiu significativamente nas últimas décadas. Um estudo de 2016 analisou as taxas de mortalidade por DCV a partir dos 30 anos de idade, por sexo, por 100 mil habitantes. As variações anuais na mortalidade cardiovascular para os períodos 1980-2006 e 2007-2012 foram, respectivamente, para ambos os sexos: -1,5% e -0,8%; para homens: -1,4% e -0,6%; para mulheres: -1,7% e -1,0%. ^40^
- Baptista *et al.* investigaram como a composição etária e as taxas de mortalidade específicas por idade se relacionam à diferença observada nas mortes por DCV na população adulta, por sexo, nas microrregiões brasileiras de 1996 a 2015. Aqueles autores sugeriram, após correção para subnotificação das mortes, que haja uma redução nas taxas de morte por DCV no período estudado. Entretanto, o principal motivo da mudança nas taxas de mortalidade foi heterogêneo nas microrregiões brasileiras. Em geral, nas áreas mais desenvolvidas socioeconomicamente, a estrutura etária relacionou-se de maneira mais importante às taxas de mortalidade, com as populações mais idosas morrendo por DCV. É interessante notar que os principais motivos da mudança nas taxas de mortalidade por DCV diferiram ainda entre as UF brasileiras. ^41^
- Dados do Estudo GBD 2017 revelam que, embora as taxas de mortalidade por DCV no Brasil tenham diminuído significativamente nos últimos anos, o número total de mortes por DCV aumentou, provavelmente como resultado do crescimento e envelhecimento da população. Houve 266.958 (II 95%, 264.385-269.671) e 388.268 (II 95%, 383.815-392.698) mortes por DCV no país em 1990 e 2017, respectivamente. A taxa de mortalidade padronizada por idade por 100 mil habitantes foi 341,8 (II 95%, 338,7-345,2) em 1990 e 178,0 (II 95%, 175,9-180) em 2017, diminuindo -47,9 (II 95%, -48,5 a -47,2) no período (Figura 1-7). Embora as taxas de mortalidade padronizadas por idade fossem maiores nos homens em todo o período, a redução percentual foi similar para ambos os sexos, e a mortalidade proporcional por DCV foi maior nas mulheres, excedendo 30% em todo o período, enquanto para os homens permaneceu sempre ligeiramente superior a 25% (Figura 1-8).
- A Tabela 1-3 mostra o número de mortes, a taxa de mortalidade padronizada por idade por 100 mil habitantes e a variação percentual por DCV, por UF e no Brasil, em 1990-2017. As UF com as maiores porcentagens de redução observadas entre 1990 e 2017 foram os estados do Espírito Santo, Paraná, Minas Gerais, Santa Catarina, Rio de Janeiro, São Paulo, e Rio Grande do Sul, nessa ordem.
- A Figura 1-9 mostra a distribuição geográfica das taxas de mortalidade por 100 mil habitantes, padronizadas por idade nas UF brasileiras, para ambos os sexos, em 2000 e 2017, de acordo com dados do SIM e utilizando a população do IBGE, a redistribuição de causas mal definidas e a correção para subnotificação de acordo com os coeficientes do GBD 2017. Houve diminuição nas taxas de mortalidade padronizadas em ambos os sexos, exceto para os homens do Maranhão e de Roraima. Malta *et al.* compararam uma série histórica de taxa de mortalidade por DCV no Brasil, usando a base de dados do SIM com e sem correção (dados brutos) e as estimativas do GBD 2017 entre 2000 e 2017. Os autores indicaram que o aumento na taxa de mortalidade bruta por DCV padronizada por idade, a partir do SIM, observado em 2017 em comparação a 2010 na maioria das UF do Norte e Nordeste resultou, na verdade, das melhorias nos registros de morte e na definição das causas básicas de morte nos últimos anos. Quando foram utilizados dados corrigidos do SIM ou as estimativas do GBD 2017, as tendências de 2010 a 2017 foram similares em todas as UF brasileiras. ^42^
- Ao analisarem dados do GBD 2015, Brant *et al.* observaram uma redução na taxa de mortalidade por DCV padronizada por idade de 429,5 (1990) para 256,0 (2015) por 100 mil habitantes (40,4%), com acentuadas diferenças entre as UF. Essa redução foi mais pronunciada nas UF do Sudeste e Sul e no Distrito Federal, regiões que concentram as maiores populações e renda, sendo mais modesta na maioria dos estados do Norte e Nordeste. Importante salientar ainda que Brant *et al.* enfatizaram que a redução anual nas taxas de mortalidade por DCV no Brasil foi menor nos últimos anos da série analisada (1990-2015). Ao se considerar o período de 1990-2017, as estimativas do GBD 2017 confirmam a mesma tendência para homens e mulheres. As Figuras 1-10 e 1-11 mostram o declínio na taxa de mortalidade por DCV padronizada por idade de 1990 a 2017, revelando uma redução no declínio nos últimos 5 anos da série, quando as taxas alcançaram um platô ou até aumentaram em algumas regiões. ^35^
- Quanto à tendência por grupo etário, as maiores reduções nas taxas de mortalidade por DCV por 100 mil, entre 1990 e 2017, foram observadas no grupo ‘abaixo de 5’ [-65,4 (-71,2; -60)], seguido pelos grupos etários ‘5-14 anos’ [-48,3 (-52,5; -42,1)], ‘50-69 anos’ [-46,5 (-47,4; -45,6)] e finalmente ‘>70 anos’, revelando um deslocamento na mortalidade por DCV para os indivíduos mais idosos.
- A cobertura do PSF foi associada com redução nas hospitalizações e na mortalidade por DCV que foram incluídas na Lista de Condições Sensíveis à Atenção Primária no Brasil, tendo seu efeito aumentado de acordo com a duração da implementação do PSF no município. Rasella *et al.* relataram reduções nas mortalidades por doença cerebrovascular e por doença cardíca de 0,82 (IC 95%: 0,79-0,86) e 0,79 (IC 95%: 0,75-0,80), respectivamente, chegando a 0,69 (IC 95%: 0,66-0,73) e 0,64 (IC 95%: 0.59-0.68), respectivamente, quando a cobertura do PSF foi consolidada no total dos 8 anos estudados. ^43^
- De acordo com o banco de dados do SIM, em 2017, as DCV corresponderam a 27,3% do total de mortes, com a maior proporção na região Sudeste e a menor na região Norte. A DIC foi responsável por 32,1% do total de mortes por DCV no Brasil e o AVC, por 28,2%. A maior proporção de mortalidade por DIC ocorreu nos estados de Mato Grosso do Sul, Pernambuco e Espírito Santo, enquanto a maior proporção de mortes por AVC ocorreu nos estados de Amazonas e Pará e no Distrito Federal (Tabela 1-4).
- A proporção de mortes por DCV diminuiu entre homens (de 30,1% para 27,6%) e mulheres (31,1% para 29,9%) de 2000-2002 a 2015-2017. Além disso, Lotufo notou um constante excesso de mortes prematuras por DCV entre os homens naquele período, com uma razão homem:mulher de 2:1. ^44^
- O SDI é uma estimativa do nível socioeconômico e pode ser usado para avaliar a associação desse nível com a carga de DCV. A Figura 1-12 mostra a correlação entre a variação percentual da taxa de mortalidade padronizada por idade 2017/1990 e o SDI de 2017. Revela uma correlação entre a maior redução na variação percentual das taxas de mortalidade por DCV padronizadas por idade, entre 1990 e 2017, e o maior SDI de 2017, sugerindo que a diminuição da mortalidade por DCV seguiu-se a uma melhora nas condições socioeconômicas locais, como observado em outros estudos. ^32 , 45 - 47^
- Lotufo *et al.* compararam 3 diferentes níveis de renda por domicílio (alto, médio e baixo) com taxas de mortalidade por DCV, na cidade de São Paulo, de 1996 a 2010. As variações percentuais anuais e os IC 95% para homens residentes em áreas de renda alta, média e baixa foram -4,1 (IC 95%: -4,5 a -3,8), -3,0 (IC 95%: -3,5 a -2,6) e -2,5 (IC 95%: -2,8 a -2,1), respectivamente. As tendências para as taxas de mulheres residentes em áreas de renda alta foram -4,4 (IC 95%: -4,8 a -3,9) em 1996-2005 e -2,6 (IC 95%: -3,8 a -1,4) em 2005-2010. A redução nas mortes por DCV foi mais significativa para homens e mulheres residentes em áreas mais abastadas, com um gradiente decrescente para risco de morte, maior para os residentes de áreas mais abastadas em comparação àqueles de áreas mais carentes. ^48^
- Observou-se associação inversa do IDHm e da cobertura de saúde suplementar com a mortalidade por DCV, sugerindo uma relação entre fatores socioeconômicos e DCV. ^45^ O IDHm aumentou entre 2000 e 2010 em todas as UF, sendo 0,7 ou maior na metade das UF. A cobertura de saúde suplementar aumentou no país durante o período estudado e associou-se inversamente com mortalidade por DCV entre 2004 e 2013. ^45^
- Soares *et al.* observaram uma diminuição na mortalidade por DCV nos estados do Rio de Janeiro, São Paulo e Rio Grande do Sul que precedeu a melhoria no índice socioeconômico. A evolução do PIB per capita, o declínio da mortalidade infantil, o maior nível educacional (representado pela escolaridade, em anos, dos indivíduos com idade superior a 25 anos) e o IDHm mostraram uma grande correlação com a redução na taxa de mortalidade por DCV. A redução nas taxas de mortalidade por DCV, AVC e DIC no estado do Rio de Janeiro nas últimas décadas foi precedido por um aumento no IDH, com números significativos, pois um aumento de 0,1 no IDH correlacionou-se com as seguintes reduções no número de mortes por 100 mil habitantes: 53,5 por DCV; 30,2 por AVC; e 10,0 por DIC. ^46 , 47^
- Baptista e Queiroz investigaram a relação entre a taxa de mortalidade bruta por DCV e o desenvolvimento econômico no tempo e no espaço, medido pelo PIB per capita, nas microrregiões brasileiras, de 2001 a 2015. Os autores, usando as bases de dados SIM-DATASUS e SIDRA do IBGE, observaram um rápido declínio nas taxas brutas de mortalidade por DCV em microrregiões do Sul e Sudeste, assim como um declínio mais lento na região Centro-Oeste. Por outro lado, as regiões Norte e Nordeste apresentaram um aumento nas taxas brutas de mortalidade por DCV ao longo do tempo, refletindo o envelhecimento mais tardio da população nessas regiões brasileiras e talvez menor acesso ao cuidado em saúde e outros fatores socioeconômicos. ^49^
- Silveira *et al.* , estudando o efeito da temperatura ambiente na mortalidade cardiovascular em 27 cidades brasileiras, observaram maior número de mortes cardiovasculares associado com temperaturas baixas e altas na maioria das cidades brasileiras e nas regiões Centro-Oeste, Norte, Sul e Sudeste. O RR geral para o Brasil foi 1,26 (IC 95%: 1,17-1,35) para o percentil 1 de temperatura e 1,07 (IC 95%: 1,01-1,13) para o percentil 99 de temperatura em comparação ao percentil 79 (27,7 °C), cujo RR foi o menor. ^50^

### Carga de Doença

- As taxas de DALYs padronizadas por idade no Brasil foram 6.907 (II 95%, 6.783-7.039) por 100 mil habitantes em 1990, caindo para 3.735 (II 95%, 3.621-3.849) por 100 mil habitantes em 2017. A região Sudeste apresentou as mais altas taxas de DALYs, enquanto as regiões Norte e Nordeste, as mais baixas (Figura 1-13). A tendência das taxas de DALYs entre 1990 e 2017 no Brasil foi similar à relatada para a taxa de mortalidade padronizada por idade: houve redução heterogênea em todas as UF, mais acentuada naquelas com melhor SDI ( Figura 1-14 e Tabela 1-5 ).
  **Figura 1-1:**
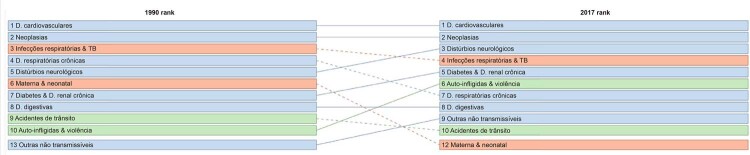
–Ranking das causas de morte no Brasil, 1990 e 2017, de acordo com as taxas de mortalidade padronizadas por idade por 100 mil habitantes, ambos os sexos, 1990 e 2017.

Dados derivados do Estudo Global Burden of Disease 2017 (GBD 2017). ^66^
  **Figura 1-2:**
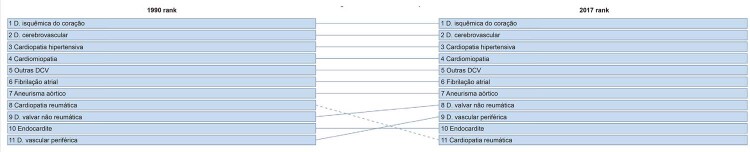
Dados derivados do Estudo Global Burden of Disease 2017 (GBD 2017). ^66^
  **Figura 1-3:**
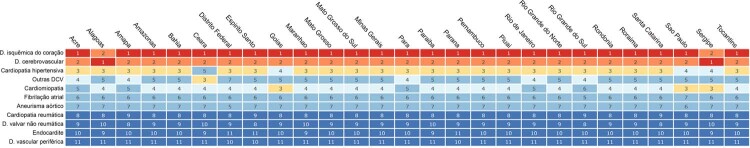
– Ranking das causas de morte cardiovascular por unidade federativa brasileira em 1990, de acordo com as taxas de mortalidade padronizadas por idade por 100 mil habitantes, ambos os sexos.

Dados derivados do Estudo Global Burden of Disease 2017 (GBD 2017).^66^
  **Figura 1-4:**
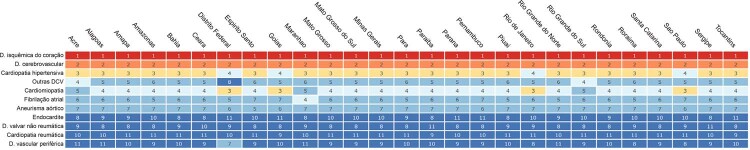
- Ranking das causas de morte cardiovascular por unidade federativa brasileira em 2017, de acordo com as taxas de mortalidade padronizadas por idade por 100 mil habitantes, ambos os sexos.

Dados derivados do Estudo Global Burden of Disease 2017 (GBD 2017). ^66^
  **Figura 1-5:**
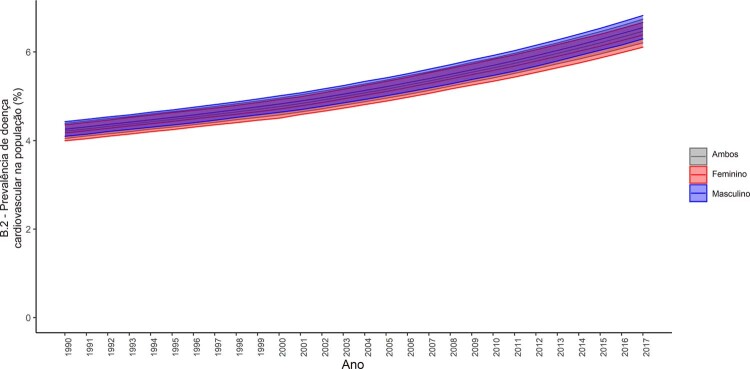
- Prevalência percentual de doença cardiovascular, por sexo, no Brasil, 1990-2017.

Dados derivados do Estudo Global Burden of Disease 2017 (GBD 2017). ^66^
  **Figura 1-6:**
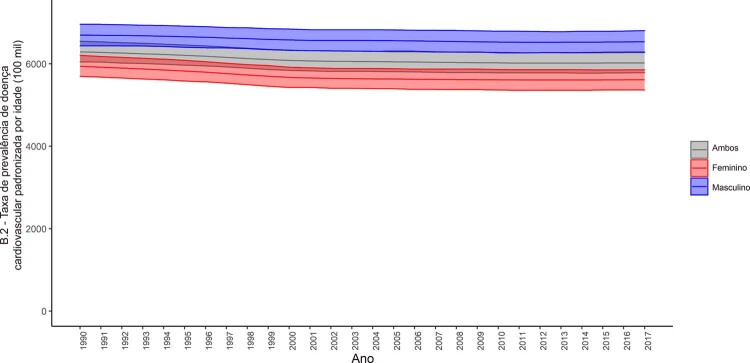
-Taxa de prevalência de doença cardiovascular padronizada por idade, por 100 mil habitantes, por sexo, Brasil, 1990-2017.

Dados derivados do Estudo Global Burden of Disease 2017 (GBD 2017). ^66^
  **Figura 1-7:**
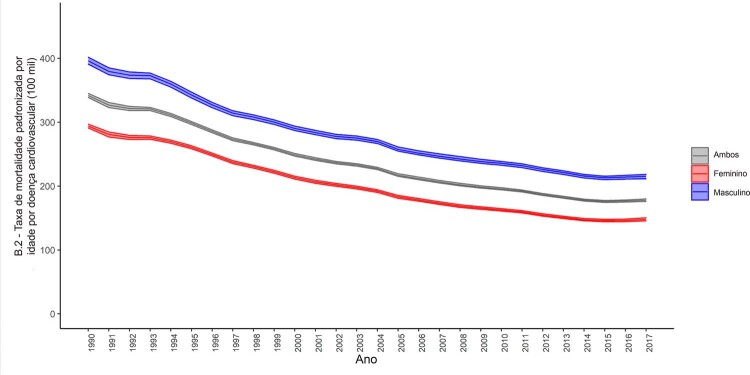
-Taxa de mortalidade padronizada por idade por doença cardiovascular, por 100 mil habitantes, por sexo, Brasil, 1990-2017.

Dados derivados do Estudo Global Burden of Disease 2017 (GBD 2017). ^66^
  **Figura 1-8:**
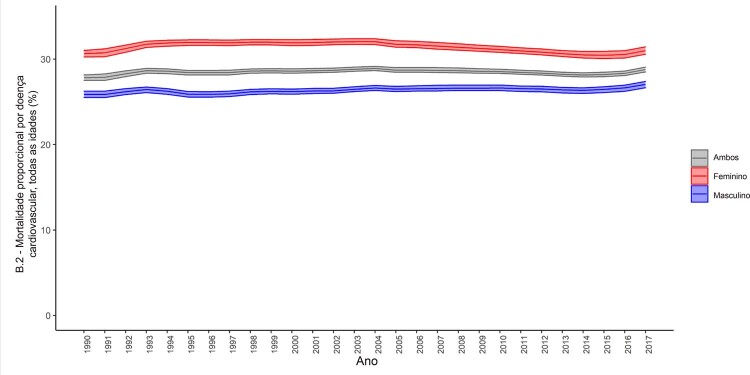
-Mortalidade proporcional por doença cardiovascular, por sexo, Brasil, 1990-2017.

Dados derivados do Estudo Global Burden of Disease 2017 (GBD 2017). ^66^
  **Figura 1-9:**
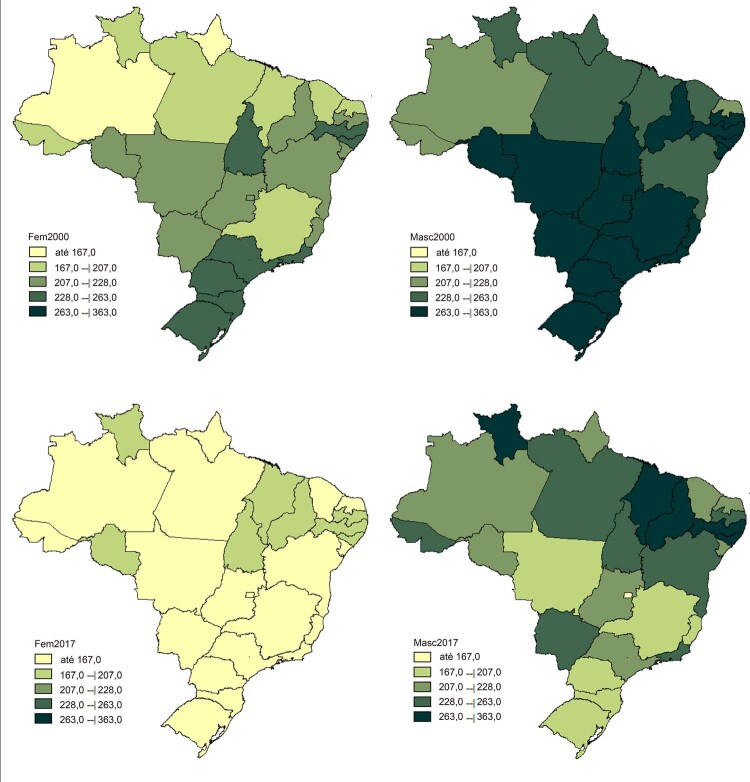
- Distribuição geográfica das taxas de mortalidade padronizadas por idade, por 100 mil habitantes, nas unidades federativas do Brasil, de acordo com sexo, 2000 e 2017.

Fonte: Sistema de Informações sobre Mortalidade – SIM/DATASUS, com redistribuição de causas mal definidas e correção para subnotificação (de acordo com os coeficientes do GBD 2017), e população IBGE. ^56^
  **Figura 1-10:**
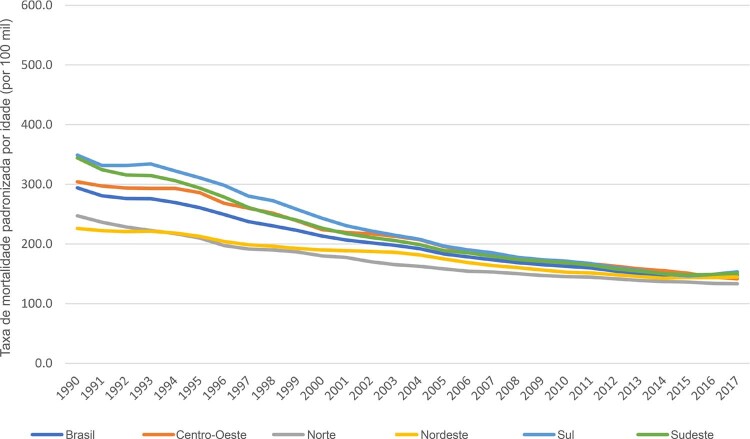
Dados derivados do Estudo Global Burden of Disease 2017 (GBD 2017). ^66^
  **Figura 1-11:**
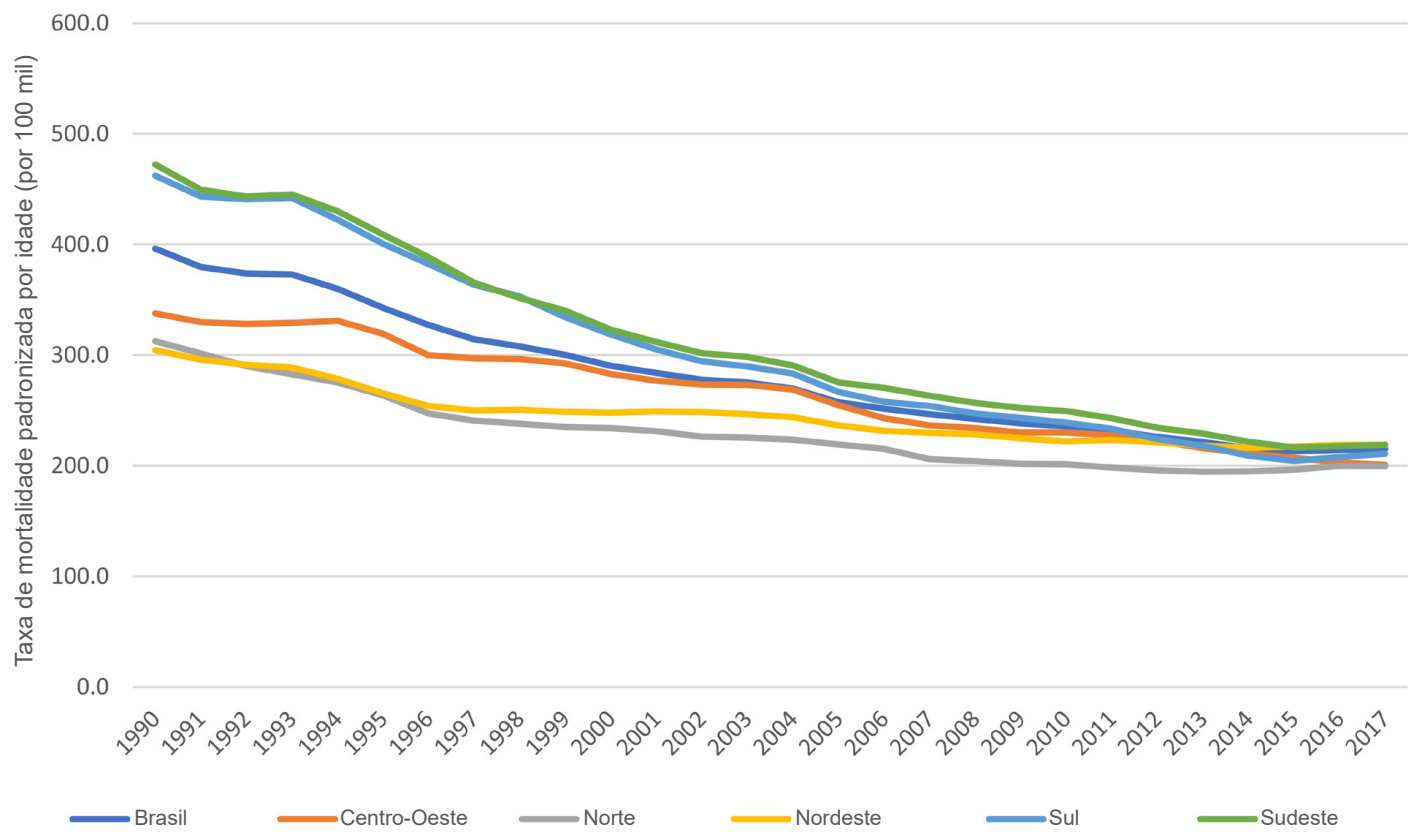
- Taxa de mortalidade padronizada por idade por doença cardiovascular, por 100 mil habitantes, por regiões brasileiras, para homens, 1990-2017.

Dados derivados do Estudo Global Burden of Disease 2017 (GBD 2017). ^66^
  **Figura 1-12:**
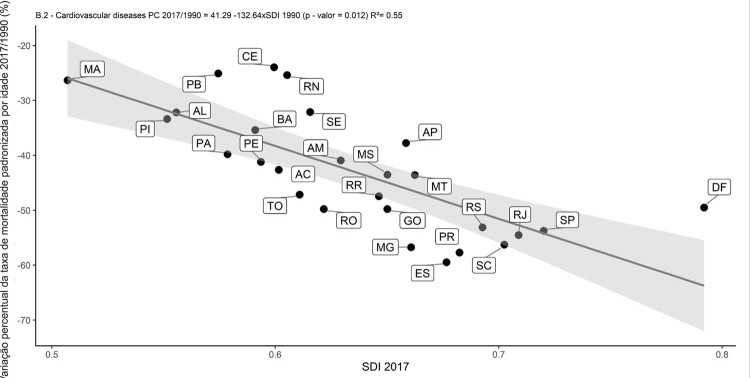
- Correlação entre variação percentual da taxa de mortalidade padronizada por idade 2017/1990 e o índice sociodemográfico 2017 (SDI 2017).

Dados derivados do DATASUS. ^56^
  **Figura 1-13:**
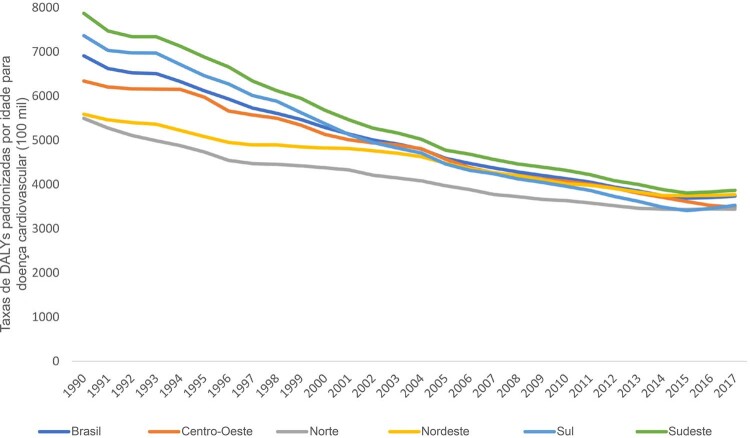
- Taxas de DALYs padronizadas por idade para doença cardiovascular, por 100 mil habitantes, 1990-2017, Brasil e suas regiões.

Dados derivados do Estudo Global Burden of Disease 2017 (GBD 2017). ^66^
  **Figura 1-14:**
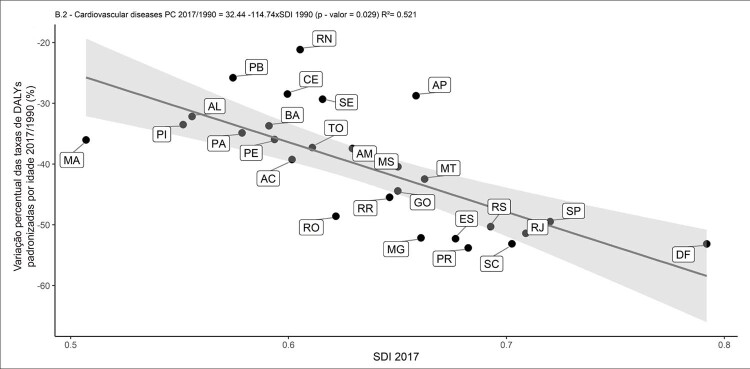
- Correlação entre a variação percentual das taxas de DALYs padronizadas por idade 2017/1990 e o índice sociodemográfico 2017 (SDI 2017).

Dados derivados do Estudo Global Burden of Disease 2017 (GBD 2017). ^66^
  **Figura 1-15:**
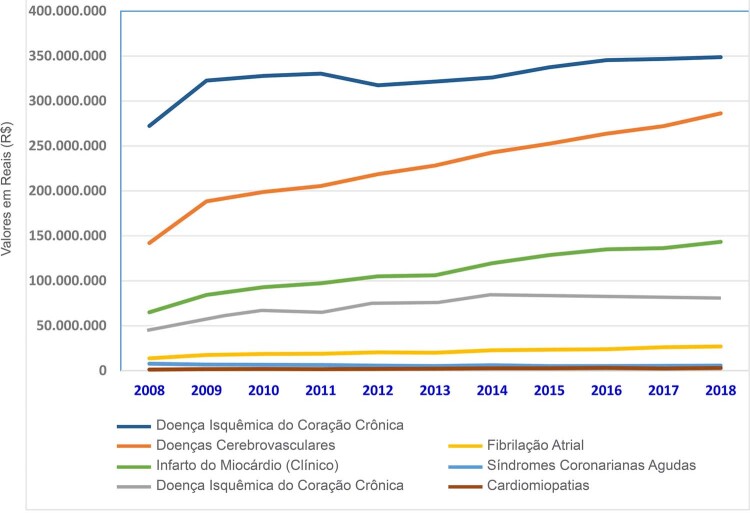
- Custos das hospitalizações clínicas referentes aos códigos dos procedimentos das mais relevantes doenças cardiovasculares de 2008 a 2018, Brasil.

Dados derivados do DATASUS. ^56^
  **Figura 1-16:**
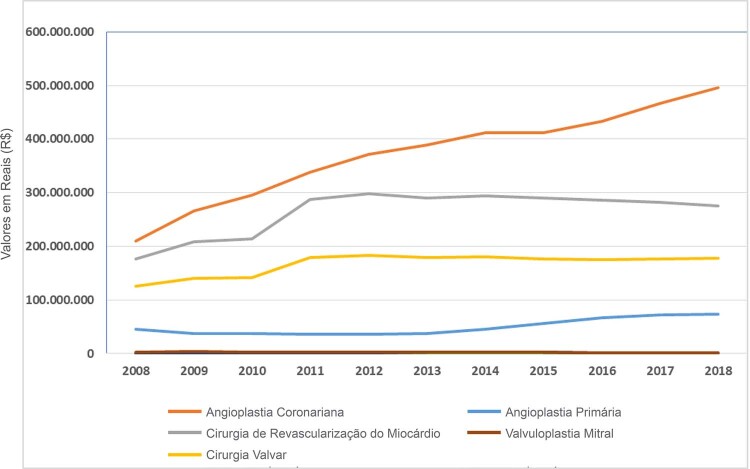
- Custos das hospitalizações cirúrgicas referentes aos códigos dos procedimentos das mais relevantes doenças cardiovasculares de 2008 a 2018, Brasil.

Dados derivados do DATASUS. ^56^

### Utilização e Custo da Atenção à Saúde

#### (Ver Tabelas 1-6 a 1-9)

- No Brasil, as DCV foram responsáveis pelos gastos diretos mais substanciais com hospitalização e pelos custos indiretos por redução da produtividade devida a ausência do trabalho. ^51 - 53^ As DCV e suas complicações resultaram em um gasto de US$ 4,18 bilhões na economia brasileira entre 2006 e 2015. ^54 , 55^
- A partir da base de dados SIH/SUS, avaliamos o número de hospitalizações de 2008 a 2018 e seus custos com base nos códigos dos procedimentos relacionados aos tópicos do presente documento: AVC; doença arterial coronariana aguda e crônica; cardiomiopatias (incluindo doença de Chagas) e insuficiência cardíaca; doenças valvares; e fibrilação atrial ( Tabela 1-6 ). Procedimentos relacionados a cardiopatia congênita, doença arterial periférica, endocardite, e arritmias, exceto fibrilação atrial, não foram incluídos.
- Quanto às hospitalizações clínicas, a insuficiência cardíaca liderou as admissões por DCV, com 2.862.739 hospitalizações (131 por 100 mil habitantes), sendo seguida por doenças cerebrovasculares com 2.042.195 hospitalizações (93 por 100 mil habitantes), IAM e SCA com 1.461.388 hospitalizações (76 por 100 mil habitantes), e fibrilação atrial com 321.866 hospitalizações (14,7 por 100 mil habitantes) ( Tabela 1-7 ). ^54^ As taxas (por 100 mil habitantes) de admissões clínicas relacionadas aos códigos dos procedimentos avaliados (324 a 293) mostraram uma diminuição de 10% de 2008 a 2018, embora o número absoluto tenha apresentado apenas discreta redução, de 615.433 em 2008 para 610.273 em 2018.
- Dos 1.149.602 procedimentos cardiovasculares cirúrgicos/intervencionistas realizados sob os códigos de procedimento selecionados ( Tabela 1-6 ), a angioplastia coronariana correspondeu a 66% (755.557), sendo seguida por CRVM (21%, 244.105) e cirurgia valvar cardíaca (8%, 88.280). A relação angioplastia/CRVM em 2008 foi 2,2, aumentando para 4,3 em 2018. Durante o período, houve aumento no número de procedimentos relacionados a infarto do miocárdio e SCA (70%). A taxa de hospitalização cirúrgica por 100 mil habitantes relacionada aos códigos dos procedimentos avaliados de 2008 a 2018 (42 a 59) mostrou aumento de 29%, enquanto o número absoluto cresceu de 80.010 em 2008 para 122.890 em 2018 ( Tabela 1-7 ).
- O estudo ELSI-Brasil, conduzido em 2015-2016 com uma amostra nacionalmente representativa da população brasileira com idade a partir de 50 anos, avaliou as hospitalizações de 9.389 participantes (idade média, 63 anos; 54% mulheres) e descobriu que 10,2% deles haviam sido hospitalizados nos 12 meses anteriores. A análise do RAP para hospitalização revelou importantes contribuições de AVC (RAP = 10,7%), DCV (RAP = 10,0%) e câncer (RAP = 8,9%). ^56 , 57^
- Cerca de metade dos custos em saúde no Brasil é financiada pelo governo. ^54^ As DCV são responsáveis pelos maiores gastos com hospitalização no SUS e criam o principal número de pensões por incapacidade e maior carga de morbidade para os pacientes. ^58 - 61^ Em 2015, o gasto estimado direto do setor público com hospitalizações e consultas para DCV no Brasil excedeu R$ 5 bilhões. Estima-se que o custo com licença temporária ou permanente por DCV tenha excedido R$ 380 milhões. ^62^
- A AHA ^62^ projetou que, para o período 2012-2030, 61% dos gastos diretos com saúde para DCV nos Estados Unidos serão atribuídos a custos hospitalares. No Brasil, em 2012, o SUS gastou US$ 608,9 milhões com procedimentos terapêuticos altamente complexos realizados durante hospitalizações por DCV, 34% dos quais foram associados com angioplastia coronariana e 25%, com CRVM. ^58 , 63^ As Tabelas 1-8 e 1-9 mostram o valor pago pelos procedimentos clínicos e cirúrgicos, respectivamente, no Brasil de 2008 a 2018 pelo sistema público de saúde. O AVC e a insuficiência cardíaca foram responsáveis pelos mais altos valores cumulativos reembolsados para procedimentos clínicos, totalizando R$ 8,4 bilhões. O total pago pelos procedimentos cirúrgicos foi R$ 9,5 bilhões, tendo angioplastia coronariana e CRVM correspondido aos maiores valores.
- De 2008 a 2018, a taxa de inflação no Brasil foi 88% (variando de 76% a 96%) e os valores não ajustados gastos pelo SUS cresceram em uma proporção similar para a maioria das condições clínicas, com duplicação no período, exceto para hospitalizações por insuficiência cardíaca, cujos valores totais reembolsados aumentaram apenas 28%, e por DIC crônica, cujos custos apresentaram uma redução de 27%. Nas admissões cirúrgicas, todos os grupos relacionados apresentaram elevação dos valores gastos – a maioria quase dobrou os valores, exceto a valvuloplastia mitral, que mostrou diminuição de 53% no valor pago pelo SUS na década ( Tabelas 1-8 e 1-9). Entretanto, ao se calcularem os valores pagos pelo SUS em dólares internacionais, os custos para a maior parte dos grupos de hospitalização clínica variaram menos do que 10%, exceto para insuficiência cardíaca, cujas despesas caíram 35%, e para infarto do miocárdio e cardiomiopatias, cujas despesas subiram 13% e 23%, respectivamente. Quanto aos procedimentos cirúrgicos/intervencionistas, houve redução nos custos em todos os grupos relatados, exceto para angioplastia coronariana, cujos custos aumentaram 20%, e cardiomiopatias, cujos custos aumentaram 30% ( Tabelas 1-8 e 1-9 ).

### Pesquisa Futura

- O SIM, implementado em 1975, é uma ferramenta essencial para monitorar as estatísticas de mortalidade no Brasil, pois o registro de todas as mortes é obrigatório nas UF, sendo que, em 2017, a cobertura do território nacional foi de 98%, menor na região Norte do que na Sul. A região Nordeste apresenta a menor cobertura, ainda inferior a 95%. ^57^ Embora o SIM tenha melhorado através de projetos específicos do Ministério da Saúde, ^65 , 66^ ainda persistem problemas, como as causas mal definidas (cerca de 6%), ‘códigos *garbage* ’ e subnotificação de mortes, que geram vieses que podem comprometer a métrica apresentada. Portanto, pesquisa adicional é necessária para promover ajustes metodológicos para cobertura e redistribuição de causas mal definidas, especialmente nos primeiros anos da série histórica. Por outro lado, as estimativas do Estudo GBD requerem mais pesquisa para a implementação de modelos com melhor distribuição de ‘códigos *garbage* ’ adaptados às realidades locais.
- Vale mencionar que, devido à falta de dados de incidência primária (coortes) no Brasil, há necessidade de pesquisa que permita compreender como enfrentar a DCV nos estados e nas populações com baixos índices socioeconômicos.
- Devido à redução da tendência de declínio da mortalidade por DCV padronizada por idade nos últimos 5 anos, novas estratégias para combater tal mortalidade devem ser estudadas. É fundamental que se compreendam os motivos para tal redução para que se implementem políticas efetivas, em particular em face ao envelhecimento da população, que vai aumentar o número de indivíduos com DCV no país.
- A maior parte dos dados sobre custo foi obtida a partir das tabelas de reembolso do Sistema Público de Saúde de 2008 a 2018, não capturando o verdadeiro custo relacionado àquelas condições. Informação confiável e abrangente do custo da prestação do cuidado nas condições cardiovasculares é extremamente importante para melhor entender o impacto financeiro dessas doenças e melhor reavaliar estratégias de prevenção e tratamento. Além disso, os dados de custo do sistema suplementar de saúde, assim como do cuidado ambulatorial, e custos indiretos são fundamentais para uma avaliação econômica abrangente das DCV no Brasil.

**Tabela 1-1:** – Taxa de prevalência de DCV padronizada por idade por 100 mil habitantes, por sexo, no Brasil e suas unidades federativas, 1990 e 2017, e variação percentual

Unidades federativas	Ambos os sexos			Feminino			Masculino		
	1990	2017	Variação percentual	1990	2017	Variação percentual	1990	2017	Variação percentual
Brasil	6290 (6048.3;6548.9)	6025 (5785.8;6274.8)	-4.2 (-3.2;-5.1)	5939.5 (5694.8;6205.5)	5612.9 (5366.3;5856.6)	-5.5 (-4.2;-6.7)	6697.3 (6433.7;6961.9)	6536.8 (6282.7;6806.6)	-2.4 (-1.3;-3.4)
Acre	6011.6 (5762.4;6272.3)	5814.9 (5568.2;6071.4)	-3.3 (-1.4;-5.3)	5595.5 (5340.7;5865.5)	5350.3 (5101.6;5618.9)	-4.4 (-1.5;-7.2)	6361.8 (6076.6;6659.2)	6299 (6033;6583.4)	-1 (1.8;-3.9)
Alagoas	5960.7 (5700.5;6233.7)	5790.1 (5543.7;6044.7)	-2.9 (-0.8;-4.8)	5603.9 (5346;5888.2)	5381 (5123;5659.8)	-4 (-1;-6.9)	6360 (6070.2;6651.8)	6307.2 (6024.2;6580.8)	-0.8 (2;-3.5)
Amapá	6219.5 (5950.1;6490.2)	6185.3 (5922.8;6478.7)	-0.5 (1.5;-2.7)	5945.1 (5646.7;6260.9)	5817.8 (5542.5;6137.7)	-2.1 (1.3;-5.4)	6515.4 (6227.7;6811.6)	6587.8 (6304.5;6889.1)	1.1 (3.8;-1.6)
Amazonas	5728.7 (5485.7;5987.5)	5701.6 (5455.9;5955)	-0.5 (1.8;-2.6)	5367.7 (5112.8;5643.3)	5244.5 (4984.1;5506.4)	-2.3 (0.8;-5.5)	6092 (5823.9;6361.4)	6183.2 (5892.3;6475.4)	1.5 (4.5;-1.3)
Bahia	5760.9 (5521.5;6019.9)	5685.9 (5433.9;5942.7)	-1.3 (0.9;-3.4)	5420.2 (5164;5706.9)	5247 (4989.7;5508.9)	-3.2 (-0.2;-6)	6147.9 (5863.3;6431.4)	6225 (5936.4;6513.3)	1.3 (4.3;-1.6)
Ceará	5860.4 (5600.4;6118.9)	5943.7 (5668.7;6231.8)	1.4 (3.8;-0.9)	5545.9 (5270.6;5820.4)	5541.3 (5256.3;5850.7)	-0.1 (3.2;-3.3)	6211.7 (5923.9;6506.7)	6448.9 (6145.4;6775.2)	3.8 (7.1;0.7)
Distrito Federal	5798.5 (5563.4;6037.2)	5529.8 (5303.6;5752.8)	-4.6 (-2.7;-6.5)	5461.1 (5211.3;5732.7)	5144.9 (4908.1;5382.3)	-5.8 (-3;-8.7)	6209.3 (5957.1;6458.7)	6061.9 (5797;6321.5)	-2.4 (0.3;-4.9)
Espírito Santo	6214.8 (5957;6478.6)	5748.2 (5490.1;5998.6)	-7.5 (-5.6;-9.5)	5868.6 (5600.7;6137.1)	5341.6 (5086.6;5596.6)	-9 (-6.1;-11.8)	6591.9 (6320.7;6883.9)	6240.1 (5950.8;6518.6)	-5.3 (-2.8;-7.8)
Goiás	5642.8 (5395.9;5901.5)	5527.3 (5295.5;5762.8)	-2 (0.1;-4.2)	5318.3 (5037.2;5601.6)	5136.8 (4890.8;5398.2)	-3.4 (0;-6.6)	5947.9 (5701.8;6217.5)	5969.3 (5701.1;6245.8)	0.4 (3.2;-2.6)
Maranhão	5596.3 (5341.3;5857.4)	5592.5 (5348.5;5844.7)	-0.1 (2;-2.1)	5196.3 (4944.4;5463.5)	5174.8 (4941.4;5424)	-0.4 (2.6;-3.3)	6026.1 (5740.7;6311.6)	6065.4 (5785.2;6348.3)	0.7 (3.5;-2.2)
Mato Grosso	5995.6 (5727.7;6258.6)	5869.7 (5612.3;6121.4)	-2.1 (0.2;-4.1)	5622 (5350.5;5896.6)	5441.8 (5189.2;5704.2)	-3.2 (-0.1;-6)	6297.7 (6001.6;6588.6)	6285.3 (5998.3;6582.1)	-0.2 (2.8;-3)
Mato Grosso do Sul	6168.4 (5915.1;6427.7)	5964.9 (5727.2;6210.6)	-3.3 (-1.3;-5.3)	5730.5 (5467.9;5987.4)	5472.6 (5219.8;5732.1)	-4.5 (-1.6;-7.4)	6571.7 (6308.5;6860.6)	6512.2 (6248.7;6790.3)	-0.9 (1.8;-3.7)
Minas Gerais	6269 (5985.9;6552.4)	6031.6 (5768.1;6308.7)	-3.8 (-1.4;-5.9)	5902 (5602;6201.4)	5614.5 (5333.5;5907.9)	-4.9 (-1.8;-7.9)	6699.1 (6401.3;6989.1)	6532.8 (6246.7;6839)	-2.5 (0.2;-5)
Pará	5842.4 (5588.6;6111.7)	5751 (5495.1;6017.5)	-1.6 (0.6;-3.6)	5511.2 (5245.2;5786.3)	5291.5 (5022.7;5567.5)	-4 (-0.7;-7.2)	6191.5 (5918.1;6485.6)	6236.9 (5947.8;6540.6)	0.7 (3.7;-2)
Paraíba	5802.6 (5546.1;6053.4)	5755.9 (5515.6;6004.5)	-0.8 (1.4;-3)	5477.7 (5212.5;5728.9)	5371 (5114.4;5651.9)	-1.9 (1.3;-5)	6170.5 (5892.1;6456.2)	6253.3 (5978.4;6539.4)	1.3 (4.1;-1.5)
Paraná	6350.3 (6083.3;6621.1)	5998 (5747.6;6250.1)	-5.5 (-3.5;-7.6)	5947.5 (5671;6238.5)	5549.2 (5289.1;5808)	-6.7 (-3.8;-9.5)	6765.8 (6472.5;7054)	6538.5 (6262.5;6813.3)	-3.4 (-0.5;-6)
Pernambuco	5864.3 (5618.3;6114.8)	5642.3 (5399.5;5887.5)	-3.8 (-1.7;-5.9)	5544.4 (5296;5814.4)	5239.5 (4986.3;5490.8)	-5.5 (-2.5;-8.3)	6252.9 (5972.2;6545.7)	6184.6 (5912.5;6459.7)	-1.1 (1.7;-3.9)
Piauí	5511.3 (5276.3;5755.1)	5545.1 (5314.7;5786.3)	0.6 (2.8;-1.4)	5145.2 (4900.4;5394.3)	5131.4 (4894.1;5385.3)	-0.3 (2.8;-3.3)	5911.2 (5642.8;6176.5)	6034.4 (5776.8;6317.2)	2.1 (4.8;-0.6)
Rio de Janeiro	6714.2 (6446.8;7008.6)	6230.8 (5980.5;6490.7)	-7.2 (-5.3;-9.2)	6350.5 (6067.4;6659.1)	5820 (5546.8;6108.8)	-8.4 (-5.6;-11.1)	7208.1 (6917.8;7498.2)	6800.2 (6527.3;7073.1)	-5.7 (-3.1;-8.1)
Rio Grande do Norte	5701.2 (5452.1;5951.2)	5672.5 (5438;5939.2)	-0.5 (1.6;-2.4)	5393 (5131.9;5659.9)	5280.7 (5029.7;5550.9)	-2.1 (0.8;-5)	6041.8 (5780.8;6327.3)	6170.4 (5892.3;6466.5)	2.1 (5.1;-0.6)
Rio Grande do Sul	6600.3 (6315.3;6880.9)	6182.1 (5906.1;6460.9)	-6.3 (-4.4;-8.3)	6322.7 (6028.2;6645.2)	5828.4 (5536.3;6116.6)	-7.8 (-4.9;-10.6)	6961.6 (6661.6;7269.6)	6639.1 (6336.7;6946.7)	-4.6 (-2;-7.3)
Rondônia	5985.6 (5731;6240.1)	5705.1 (5457.5;5949.3)	-4.7 (-2.4;-6.8)	5599.4 (5348.2;5868.1)	5285.8 (5041.4;5543.5)	-5.6 (-2.6;-8.6)	6276.9 (5998.5;6564.3)	6111.8 (5837.6;6398.4)	-2.6 (0.4;-5.3)
Roraima	6064.1 (5818.4;6308.8)	5814.1 (5583.7;6060.7)	-4.1 (-2.1;-6)	5617 (5371;5884)	5317.9 (5070;5562.2)	-5.3 (-2.4;-8.1)	6407.7 (6148.3;6694.1)	6269.6 (6021.5;6541.3)	-2.2 (0.4;-4.9)
Santa Catarina	6679.1 (6397.8;6964.3)	6217 (5941.2;6488.4)	-6.9 (-5;-8.8)	6375.3 (6069.7;6674.6)	5844.5 (5564.4;6133.5)	-8.3 (-5.4;-11.1)	7026.3 (6731;7339.3)	6667 (6373.5;6959.6)	-5.1 (-2.3;-7.8)
São Paulo	6801.7 (6517.3;7096.7)	6423.9 (6151.9;6705.8)	-5.6 (-3.4;-7.5)	6406.6 (6114.7;6715.3)	6000.7 (5715;6302.4)	-6.3 (-3.1;-9.3)	7284.4 (6975.5;7612.3)	6975.7 (6661.7;7268.7)	-4.2 (-1.4;-6.8)
Sergipe	5922.8 (5667.3;6171.5)	5851.4 (5597.1;6110.4)	-1.2 (1.2;-3.4)	5582.1 (5328.1;5864.7)	5442.8 (5194.8;5717)	-2.5 (0.7;-5.3)	6324.1 (6036.1;6611.2)	6374.1 (6091.1;6662.7)	0.8 (3.9;-1.8)
Tocantins	5849.3 (5606.1;6103.5)	5849.2 (5606.6;6094.9)	0 (2.1;-2.1)	5387.1 (5153.3;5644.6)	5307.1 (5065.3;5571.5)	-1.5 (1.5;-4.3)	6261.9 (5993.8;6548.1)	6368.8 (6094;6643.8)	1.7 (4.6;-1)

*Fonte: Estudo Global Burden of Disease 2017, Institute for Health Metrics and Evaluation. ^66^*

**Tabela 1-2:** – Número de casos e incidência padronizada por idade (por 100 mil) de DCV no Brasil e em suas unidades federativas, 1990 e 2017, e variação percentual

Unidades federativas brasileiras	1990		2017		Variação percentual (II 95%)
	Número (II 95%)	Taxa (II 95%)	Número (II 95%)	Taxa (II 95%)	
Brasil	723595.1 (700074.3;750549.6)	755.6 (731.6;783)	1550188.5 (1495647.3;1607115.8)	687.5 (663.4;712.4)	-9 (-9.9;-8.2)
Acre	1400.9 (1352.7;1453.7)	701.5 (675.5;730.5)	4219.8 (4070.7;4381.3)	673.9 (648.3;700.9)	-3.9 (-5.4;-2.4)
Alagoas	10692.8 (10301.7;11114.3)	715.2 (688;744.7)	21304 (20503.3;22148.2)	675.8 (649.9;702.9)	-5.5 (-7;-4)
Amapá	845.7 (817.2;875.8)	697.3 (672.7;723.9)	3589.8 (3464.8;3720.7)	680.8 (655.6;706.2)	-2.4 (-3.9;-0.9)
Amazonas	6580.8 (6356.7;6809.1)	683.2 (658.2;709.5)	19228.5 (18527.4;19964.4)	667.3 (643.2;695.3)	-2.3 (-3.7;-0.9)
Bahia	53499.5 (51571.2;55573.5)	715.2 (689.1;744.7)	108386.9 (104426.4;112633.4)	686 (660.4;713)	-4.1 (-5.6;-2.5)
Ceará	29391.6 (28364.7;30541.6)	668.4 (644.1;695.3)	64457.4 (61963.2;67082.7)	657.3 (631.5;684.4)	-1.7 (-3.2;-0.1)
Distrito Federal	5460.4 (5280.9;5656.2)	735.9 (711.2;762.2)	17461 (16845.3;18144.4)	681 (657.1;706.6)	-7.5 (-8.8;-6.1)
Espírito Santo	12056.9 (11646.4;12504.3)	761.1 (733.7;789.4)	28206.7 (27136.4;29299.3)	669.3 (644.8;695.5)	-12.1 (-13.6;-10.6)
Goiás	16069.6 (15492.1;16700.6)	721.2 (695.6;749.2)	44756.7 (43051.4;46526.6)	667.8 (643.2;694.3)	-7.4 (-8.8;-6.1)
Maranhão	19179.5 (18453.2;19975.2)	668.8 (642.1;698.5)	42991.2 (41329.7;44766)	660.5 (634.6;689.6)	-1.3 (-2.8;0.3)
Mato Grosso	6719.6 (6494.8;6965.5)	704 (679.9;731.2)	21357.5 (20543;22189.1)	675.9 (649.6;702.9)	-4 (-5.4;-2.4)
Mato Grosso do Sul	7537.2 (7281.1;7807.5)	744.2 (717.8;772.7)	19409.2 (18668.4;20188)	693.7 (667.6;721.3)	-6.8 (-8.1;-5.4)
Minas Gerais	80523.3 (77820.9;83428.5)	768.2 (742;795.2)	171252.6 (164841.8;177705.6)	685.1 (660.4;709.9)	-10.8 (-12.3;-9.4)
Pará	17011.1 (16426.2;17625.4)	688.6 (662.5;716.5)	45519.2 (43870.8;47258)	661.7 (637.2;688.8)	-3.9 (-5.4;-2.5)
Paraíba	16176.5 (15565.7;16826.5)	667.2 (642.6;693.4)	29872.4 (28740.8;31073.8)	657.2 (632.5;684.2)	-1.5 (-3;0)
Paraná	41419.6 (40008.6;42968.7)	800.3 (773;830.5)	87883.8 (84611.7;91450.2)	695.9 (670.7;722.7)	-13 (-14.5;-11.6)
Pernambuco	35110.1 (33804.7;36547.8)	732.2 (705.7;761.9)	66763.4 (64155.4;69477.8)	675.2 (649.1;703)	-7.8 (-9.3;-6.2)
Piauí	10631.7 (10240.3;11034.3)	670.2 (644.4;697.2)	23550.9 (22654.2;24486.5)	655.2 (629.6;681.5)	-2.2 (-3.7;-0.7)
Rio de Janeiro	81868.9 (78983.8;85145.6)	821.9 (793.4;852.5)	150842.8 (145276.6;156396.1)	709 (683.9;734.5)	-13.7 (-15.1;-12.3)
Rio Grande do Norte	11276.8 (10853;11708.8)	654.7 (629.9;680.7)	24101.3 (23205;25082.8)	646.6 (621.9;674.4)	-1.2 (-2.6;0.3)
Rio Grande do Sul	53751.8 (51853.1;55798.7)	788.9 (761.7;817.8)	101785.9 (97835;105973.3)	695.5 (669.9;722.4)	-11.8 (-13.3;-10.3)
Rondônia	3371.8 (3252.6;3505)	720.6 (695.4;749.2)	9991.1 (9629.9;10396.4)	668.7 (643.9;697)	-7.2 (-8.7;-5.7)
Roraima	568.1 (548.3;589.3)	720.4 (694.8;748.1)	2537.1 (2444.2;2635)	677 (651.8;703.1)	-6 (-7.4;-4.7)
Santa Catarina	21007.9 (20283;21746.3)	763.1 (736.1;790.2)	52679.6 (50763.8;54847.6)	685.8 (661.2;712.9)	-10.1 (-11.6;-8.7)
São Paulo	171665.5 (165924.5;178117.1)	803.6 (775.3;833.8)	364049.8 (350776.2;377905.8)	707.2 (681.6;733.4)	-12 (-13.4;-10.6)
Sergipe	6408.5 (6185.3;6643.9)	693.3 (667.4;720.5)	14467 (13942.5;15036.2)	667.5 (642.1;694.7)	-3.7 (-5.2;-2.2)
Tocantins	3369.1 (3254.2;3500)	711.8 (686.8;739.6)	9522.9 (9187.6;9895.5)	676.5 (652.2;704)	-5 (-6.5;-3.5)

*II: intervalo de incerteza; Fonte: Estudo Global Burden of Disease 2017, Institute for Health Metrics and Evaluation. ^66^*

**Tabela 1-3:** – Número de mortes e taxa de mortalidade padronizada por idade (por 100 mil) por DCV, no Brasil e em suas unidades federativas, 1990 e 2017, e variação percentual

	1990		2017		Variação percentual (II 95%)
	Número (II 95%)	Taxa (II 95%)	Número (II 95%)	Taxa (II 95%)	
Brasil	266957.7 (264384.5;269670.5)	341.8 (338.7;345.2)	388268.1 (383814.8;392697.7)	178 (175.9;180)	-47.9 (-48.5;-47.2)
Acre	398.2 (388.1;407.4)	276.3 (269.5;282.7)	860.3 (827.9;893.3)	158.5 (152.2;164.7)	-42.6 (-45.5;-39.5)
Alagoas	4053.8 (3962.9;4148.6)	312.4 (306.1;318.9)	6330.3 (6126.2;6536.9)	211.9 (204.9;218.9)	-32.2 (-34.8;-29.5)
Amapá	204.7 (199.9;209.6)	252.5 (246.8;258.8)	668.5 (646.7;691.1)	157.2 (152.2;162.6)	-37.8 (-40.2;-35.1)
Amazonas	1671.4 (1607.3;1730)	248.9 (239.5;257.4)	3566.2 (3452.4;3679.5)	147 (142.3;151.9)	-40.9 (-43.7;-37.8)
Bahia	16748.2 (15951.3;17572.4)	252.2 (240.6;264.6)	25924.4 (25261.1;26649.5)	162.9 (158.6;167.6)	-35.4 (-38.8;-31.7)
Ceará	8157.7 (7634.1;8698.6)	200.3 (187.1;213.5)	15199.6 (14788.3;15643.8)	152.4 (148.2;156.9)	-24 (-28.8;-18.3)
Distrito Federal	1564 (1530.6;1593.1)	347.4 (340.8;353.3)	3196.5 (3061;3340.7)	175.4 (168;183.2)	-49.5 (-51.8;-47)
Espírito Santo	4547.7 (4479.3;4621.1)	409.1 (403.1;415.6)	6692 (6473.6;6899.1)	165.8 (160.3;171)	-59.5 (-60.9;-58.1)
Goiás	5034.5 (4904.9;5160.8)	326.3 (317.6;334.9)	10071.3 (9753.2;10423.5)	163.9 (158.7;169.5)	-49.8 (-51.4;-48)
Maranhão	6355.1 (5965.1;6814.3)	250.6 (234.1;269)	11471.5 (11001.2;11997)	184.6 (177;193.1)	-26.3 (-30.8;-22)
Mato Grosso	1969.9 (1862.5;2089.6)	288.6 (274.1;305)	4470.8 (4292.6;4648.3)	162.8 (156.5;169.3)	-43.6 (-47.1;-39.8)
Mato Grosso do Sul	2619.6 (2546.5;2676.5)	351.7 (344.2;358.2)	5149.6 (4987.1;5336)	198.6 (192.5;205.5)	-43.5 (-45.6;-41.2)
Minas Gerais	29369 (28899.2;29849.4)	357.1 (351.8;362.5)	38721.8 (37782;39823.5)	154.5 (150.8;159)	-56.7 (-58;-55.3)
Pará	5266.9 (5055.8;5475)	280 (269.1;290.5)	10353.6 (9971.8;10725.7)	168.6 (162.5;174.7)	-39.8 (-43.2;-36.4)
Paraíba	5718.5 (5474.8;5964.4)	254.9 (244.2;265.8)	8984.7 (8426.7;9582.7)	190.9 (179;203.9)	-25.1 (-30.9;-18.6)
Paraná	16504.7 (16270.4;16731.9)	445.3 (438.8;451.5)	22160.3 (21559.6;22775.3)	188.3 (183.3;193.6)	-57.7 (-59.1;-56.4)
Pernambuco	14360.8 (14092.9;14607.3)	364.9 (358.4;371)	20620.7 (20029;21264.3)	214.6 (208.3;221.3)	-41.2 (-43;-39.3)
Piauí	3514.8 (3300.4;3730.5)	262.9 (247.1;279.1)	6327.5 (6132.3;6535.5)	175.1 (169.7;181)	-33.4 (-37.3;-28.8)
Rio de Janeiro	37561.8 (37050.8;38028.3)	456.7 (450.5;462.1)	43858 (42710.9;45057.9)	207.7 (202.4;213.3)	-54.5 (-55.8;-53.2)
Rio Grande do Norte	3386.4 (3206.1;3566.2)	213.3 (202;224.8)	6068 (5855.2;6289.3)	159.2 (153.6;165.1)	-25.4 (-30;-19.9)
Rio Grande do Sul	20393.7 (20091.6;20681.9)	378.1 (372.6;383.2)	25992.2 (25243.6;26731.3)	177.2 (172.1;182.3)	-53.1 (-54.6;-51.6)
Rondônia	1006.4 (941.1;1071.3)	368.1 (347.8;387.7)	2351.5 (2146.6;2592.9)	184.8 (169.6;202.6)	-49.8 (-54.8;-44.5)
Roraima	146.8 (135.1;159.4)	373.6 (349.4;399.7)	495.4 (447.4;549.9)	196.3 (178.6;216)	-47.5 (-53.5;-41.2)
Santa Catarina	7674.7 (7557;7795.4)	389.1 (383.2;395.1)	11764.8 (11388.8;12125)	170.2 (164.9;175.2)	-56.3 (-57.8;-54.7)
São Paulo	65661.8 (64717.7;66544.9)	401.2 (395.5;406.2)	91136 (88785.8;93412.4)	185.6 (180.7;190.2)	-53.8 (-55;-52.5)
Sergipe	2100.9 (2048.7;2155)	252.8 (246.6;259.1)	3529.6 (3404.6;3655.8)	171.6 (165.6;177.7)	-32.1 (-35;-29.2)
Tocantins	965.7 (864.4;1053.9)	329.1 (304.8;353.4)	2302.8 (2183.2;2429)	173.9 (165.1;183.1)	-47.2 (-51.4;-42.2)

*II: intervalo de incerteza; Fonte: Estudo Global Burden of Disease 2017, Institute for Health Metrics and Evaluation. ^66^*

**Tabela 1-4:** – Mortalidade proporcional por doença cardiovascular, doença isquêmica do coração e acidente vascular cerebral, por região brasileira, unidade federativa e no Brasil, 2017

Região Unidade federativa	DCV/Total %	DIC/DCV %	AVC/DCV %
**Norte**	**22,9**	**30,6**	**33,4**
Rondônia	24,2	33,0	27,7
Acre	22,3	27,6	32,4
Amazonas	18,1	27,7	36,9
Roraima	21,3	24,8	30,3
Pará	23,4	31,4	35,1
Amapá	19,6	32,3	34,2
Tocantins	30,8	31,0	27,7
**Nordeste**	**27,2**	**32,2**	**30,3**
Maranhão	30,8	33,0	33,2
Piauí	32,5	30,0	33,1
Ceará	26,3	31,4	31,7
Rio Grande do Norte	25,8	38,9	24,0
Paraíba	29,2	35,4	26,1
Pernambuco	28,1	37,8	28,2
Alagoas	30,2	29,6	30,2
Sergipe	23,5	29,5	31,5
Bahia	24,0	26,4	31,8
**Sudeste**	**28,3**	**32,4**	**25,7**
Minas Gerais	25,4	24,4	28,4
Espírito Santo	28,8	36,8	28,0
Rio de Janeiro	27,9	32,9	25,1
São Paulo	29,8	34,9	24,8
**Sul**	**27,2**	**31,1**	**30,2**
Paraná	28,3	29,4	29,5
Santa Catarina	27,4	32,4	25,7
Rio Grande do Sul	26,2	32,1	32,9
**Centro-Oeste**	**26,4**	**33,0**	**28,4**
Mato Grosso do Sul	28,6	37,9	29,3
Mato Grosso	24,3	31,2	27,6
Goiás	26,2	31,8	26,3
Distrito Federal	26,7	32,2	35,1
**BRASIL**	**27,3**	**32,1**	**28,2**

* Fonte: Sistema de Informações sobre Mortalidade – SIM/DATASUS. ^56^*

**Tabela 1-5:** – Códigos dos procedimentos incluídos em cada grupo de condições para análise de custo

Tipo	Grupo de Doença	Código	Descrição do Código
Cirúrgico	Ablação de fibrilação atrial	0406050074	Estudo Eletrofisiológico Terapêutico II (Ablação de Fibrilação atrial)
Cirúrgico	Angioplastia coronariana	0406030073	Angioplastia em Enxerto Coronariano (c/ Implante de Stent)
Cirúrgico	Angioplastia coronariana	0406030014	Angioplastia Coronariana
Cirúrgico	Angioplastia coronariana	0406030065	Angioplastia em Enxerto Coronariano
Cirúrgico	Angioplastia coronariana	0406030022	Angioplastia Coronariana c/ Implante de dois Stents
Cirúrgico	Angioplastia coronariana	0406030030	Angioplastia Coronariana c/ Implante de Stent
Cirúrgico	Cirurgia de Revascularização do Miocárdio	0406010927	Revascularização Miocárdica c/ uso de Extracorpórea
Cirúrgico	Cirurgia de Revascularização do Miocárdio	0406010935	Revascularização Miocárdica c/ uso de Extracorpórea (c/ 2 ou mais enxertos)
Cirúrgico	Cirurgia de Revascularização do Miocárdio	0406010943	Revascularização Miocárdica s/ uso de Extracorpórea
Cirúrgico	Cirurgia de Revascularização do Miocárdio	0406010951	Revascularização Miocárdica s/ uso de Extracorpórea (c/ 2 ou mais enxertos)
Cirúrgico	Cirurgia valvar	0406010811	Plástica valvar c/ Revascularização Miocárdica
Cirúrgico	Cirurgia valvar	0406010340	Correção de Insuficiência da Válvula Tricúspide
Cirúrgico	Cirurgia valvar	0406010692	Implante de Prótese Valvar
Cirúrgico	Cirurgia valvar	0406010021	Abertura de estenose Aórtica Valvar
Cirúrgico	Cirurgia valvar	0406010803	Plástica Valvar
Cirúrgico	Cirurgia valvar	0406010030	Abertura de Estenose Pulmonar Valvar
Cirúrgico	Cardiomiopatias	0406011397	Correção de Hipertrofia Septal Assimétrica (criança e adolescente)
Cirúrgico	Cirurgia valvar	0406011206	Troca valvar c/ Revascularização Miocárdica
Cirúrgico	Angioplastia primária	0406030049	Angioplastia Coronariana Primária
Cirúrgico	Cardiomiopatias	0406011397	Correção de Hipertrofia Septal Assimétrica (criança e adolescente)
Cirúrgico	Outras valvuloplastias	0406030146	Valvuloplastia Tricúspide Percutânea
Cirúrgico	Outras valvuloplastias	0406030138	Valvuloplastia Pulmonar Percutânea
Cirúrgico	Outras valvuloplastias	0406030111	Valvuloplastia Aórtica Percutânea
Cirúrgico	Valvuloplastias mitrais	0406030120	Valvuloplastia Mitral Percutânea
Clínico	Doença isquêmica do coração crônica	0303060042	Tratamento de Cardiopatia Isquêmica Crônica
Clínico	Acidente vascular cerebral	0303040149	Tratamento de Acidente Vascular Cerebral - AVC (isquêmico ou hemorrágico agudo)
Clínico	Acidente vascular cerebral	0303040076	Tratamento Conservador da Hemorragia Cerebral
Clínico	Acidente vascular cerebral	0303040300	Tratamento do Acidente Vascular Cerebral Isquêmico Agudo com uso de Trombolítico
Clínico	Doenças valvares	0303060123	Tratamento de Doença Reumática s/ Cardite
Clínico	Doenças valvares	0303060115	Tratamento de Doença Reumática c/ Comprometimento Cardíaco
Clínico	Fibrilação atrial	0303060026	Tratamento de Arritmias*
Clínico	Infarto do miocárdio – Tratamento clínico	0303060190	Tratamento de Infarto Agudo do Miocárdio
Clínico	Insuficiência cardíaca	0303060131	Tratamento de Edema Agudo de Pulmão
Clínico	Insuficiência cardíaca	0303060212	Tratamento de Insuficiência Cardíaca
Clínico	Cardiomiopatias	0303060239	Tratamento de Miocardiopatias
Clínico	Cardiomiopatias	0303060034	Tratamento de Cardiopatia Hipertrófica
Clínico	Síndromes coronarianas agudas	0303060280	Tratamento de Síndrome Coronariana Aguda

*If CID 10 I48

* Fonte: Sistema de Informações sobre Mortalidade – SIM/DATASUS. ^56^*

**Tabela 1-6:** – Procedimentos pagos pelo SUS de 2008 a 2018, por grupo de procedimentos.

Grupo de Procedimentos	Número de procedimentos pagos pelo SUS
**Cardiomiopatias e Insuficiência Cardíaca**	
Insuficiência cardíaca	2.862.739
Cardiomiopatias	24.964
**Doença Isquêmica do Coração**	
Crônica, Tx clínico	87.894
Infarto do miocárdio, Tx clínico	676.467
Angioplastia primária	85.664
Síndrome coronariana aguda, Tx clínico	784.921
Angioplastia coronariana	669.893
CRVM	244.105
**Acidente Vascular Cerebral**	2 042.195
**Doença Valvar**	
Tx clínico	32.795
Cirurgias	139.131
Valvuloplastia mitral	4.204
Outras valvuloplastias	5.087
**Fibrilação Atrial **	321.866
Ablação de fibrilação atrial	1.250

*CRVM: Cirurgia de Revascularização do Miocárdio; Tx: Tratamento.*

* Fonte: Sistema de Informações sobre Mortalidade – SIM/DATASUS. ^56^*

**Tabela 1-7:** – Número absoluto e taxas de procedimentos anuais pagos pelo SUS, de 2008 a 2018, por grupo de doença*

Procedimento code	2008		2009		2010		2011		2012		2013		2014		2015		2016		2017		2018	
	**N**	**por 100 mil**	**N**	**por 100 mil**	**N**	**por 100 mil**	**N**	**por 100 mil**	**N**	**por 100 mil**	**N**	**por 100 mil**	**N**	**por 100 mil**	**N**	**por 100 mil**	**N**	**por 100 mil**	**N**	**por 100 mil**	**N**	**por 100 mil**
**Cardiomiopatias e Insuficiência Cardíaca**																						
Insuficiência cardíaca	298,474	157.41	297,763	155.48	289,11	151.58	284,844	148.06	264,469	136.34	254,285	126.47	243,913	120.27	240,832	117.78	236,358	114.67	230,297	110.90	222,394	106.67
Cardiomiopatias	2,092	1.10	2,363	1.23	2,459	1.29	2,302	1.20	2,357	1.22	2,293	1.14	2,370	1.17	2,230	1.09	2,250	1.09	1,997	0.96	2,251	1.08
**Doença Isquêmica do Coração**																						
Crônica, Tx clínico	12,393	6.54	9,743	5.09	9,300	4.88	8,497	4.42	8	4.12	7,197	3.58	7,581	3.74	6,403	3.13	6,317	3.06	6,171	2.97	6,292	3.02
Infarto do miocárdio, Tx clínico	47,358	24.98	50,987	26.62	55,513	29.11	58,194	30.25	59,562	30.71	58,552	29.12	62,809	30.97	66,647	32.59	70,441	34.18	71,835	34.59	74,569	35.77
Angioplastia primária	7,648	4.03	6,362	3.32	6,262	3.28	6,033	3.14	5,865	3.02	6,055	3.01	7,135	3.52	8,524	4.17	10,195	4.95	10,774	5.19	10,811	5.19
Síndrome coronariana aguda, Tx clínico	63,300	33.38	68,833	35.94	72,912	38.23	71,523	37.18	75,734	39.04	73,432	36.52	76,945	37.94	72,686	35.55	70,430	34.17	70,713	34.05	68,413	32.81
Angioplastia coronariana	38,635	20.38	45,648	23.84	49,492	25.95	55,931	29.07	60,959	31.43	63,838	31.75	66,492	32.79	66,55	32.55	69,802	33.87	73,971	35.62	78,575	37.69
CRVM	20,515	10.82	22,077	11.53	21,225	11.13	23,187	12.05	23,9	12.32	23,249	11.56	22,997	11.34	22,559	11.03	22,248	10.79	21,474	10.34	20,674	9.92
AVC	159,545	84.14	176,047	91.93	181,035	94.92	184,751	96.03	182,065	93.86	183,043	91.04	187,110	92.26	191,678	93.74	195,787	94.99	198,068	95.38	203,066	97.40
**Doenças Valvares**																						
Tx clínico	3,237	1.71	4,156	2.17	3,526	1.85	3,637	1.89	3,285	1.69	2,996	1.49	2,753	1.36	2,400	1.17	2,244	1.09	2,231	1.07	2,330	1.12
Cirurgias	12,201	6.43	12,664	6.61	12,169	6.38	13,181	6.85	13,435	6.93	13,067	6.50	12,993	6.41	12,624	6.17	12,432	6.03	12,277	5.91	12,088	5.80
Valvuloplastia mitral	477	0.25	551	0.29	478	0.25	473	0.25	403	0.21	431	0.21	408	0.20	341	0.17	206	0.10	236	0.11	200	0.10
Outras valvuloplastias	451	0.24	477	0.25	445	0.23	486	0.25	456	0.24	527	0.26	515	0.25	513	0.25	399	0.19	427	0.21	391	0.19
**Fibrilação Atrial**																						
FA	29,034	15.31	28,174	14.71	28,382	14.88	28,583	14.86	28,760	14.83	28,268	14.06	29,799	14.69	29,754	14.55	29,889	14.50	30,265	14.57	30,958	14.85
Ablação de FA	68	0.04	72	0.04	90	0.05	85	0.04	123	0.06	139	0.07	143	0.07	161	0.08	124	0.06	120	0.06	125	0.06

* Grupos de doença de acordo com os códigos de procedimentos da Tabela 1-5.

*AVC: Acidente Vascular Cerebral, CRVM: Cirurgia de Revascularização do Miocárdio, FA: fibrilação atrial, Tx: tratamento.*

* Fonte: Sistema de Informações sobre Mortalidade – SIM/DATASUS. ^56^*

**Tabela 1-8:** –Valores totais reembolsados não ajustados e ajustados para Int$2018 das internações clínicas cardiovasculares pagas pelo SUS de 2008 a 2018. Dados expressos em milhares (1.000)

	2008		2009		2010		2011		2012		2013		2014		2015		2016		2017		2018		TOTAL		
	R$	Int$ 2018	R$	Int$ 2018	R$	Int$ 2018	R$	Int$ 2018	R$	Int$ 2018	R$	Int$ 2018	R$	Int$ 2018	R$	Int$ 2018	R$	Int$ 2018	R$	Int$ 2018	R$	Int$ 2018	R$	R$ + IPCA 10 ANOS	Int$ 2018
Doença isquêmica do coração crônica	7.799	7.519	6.861	6.167	6.593	5.465	6.251	4.781	5.690	4.031	5.248	3.460	6.214	3.798	5.141	2.921	5.327	2.800	5.532	2.811	5.672	2.797	66,328	125,224	46,550
Acidente vascular cerebral	142.062	136.975	188.450	169.388	198.813	164.774	205.448	157.139	218.628	154.902	228.141	150.393	242.664	148.315	252.441	143.442	263.771	138.669	272.140	138.253	286.293	141.170	2,498,850	4,717,691	1,643,419
Doenças valvares	1.052	1.014	1.589	1.428	1.439	1.193	1.607	1.229	1.509	1.069	1.510	995	1.584	968	1.672	950	1.675	881	1.679	853	2.043	1.008	17,361	32,776	11,590
Fibrilação atrial	13.791	13.297	17.396	15.636	18.537	15.363	18.858	14.424	20.371	14.433	19.969	13.164	22.637	13.835	23.330	13.256	23.929	12.580	26.061	13.239	26.971	13.300	231,850	437,720	152,529
Infarto do miocárdio (clínico)	65.020	62.691	84.308	75.780	92.969	77.051	97.324	74.439	104.898	74.328	106.246	70.040	119.583	73.088	128.724	73.143	134.912	70.925	136.438	69.313	143.349	70.685	1,213,077	2,291,532	791,479
Insuficiência cardíaca	272.281	262.532	322.849	290.192	327.914	271.771	330.492	252.781	317.586	225.015	321.712	212.077	326.141	199.336	337.610	191.834	345.566	181.670	346.841	176.203	348.832	172.008	3,598,825	6,792,495	2,435,420
Cardiomiopatias	1.288	1.242	1.902	1.709	2.144	1.777	1.900	1.453	2.110	1.495	2.302	1.517	2.696	1.648	2.682	1.524	3.065	1.611	2.556	1.299	3.120	1.538	25,764	48,641	16,813
Síndromes coronarianas agudas	44.711	43.110	57.922	52.063	64.612	53.550	65.586	50.165	75.210	53.288	74.619	49.190	83.607	51.100	82.095	46.648	80.185	42.155	82.072	41.694	80.037	39.466	790,657	1,492,716	522,427
**Total**	**548.001**	**528.381**	**681.277**	**612.364**	**713.021**	**590.944**	**727.466**	**556.412**	**746.003**	**528.555**	**759.747**	**500.835**	**805.126**	**492.089**	**833.695**	**473.719**	**858.430**	**451.291**	**873.320**	**443.665**	**896.319**	**441.972**	**8,442,405**	**15,938,795**	**5,620,226**

* Fonte: Sistema de Informações sobre Mortalidade – SIM/DATASUS. ^29^*

**Tabela 1-9:** – Valores totais reembolsados não ajustados e ajustados para Int$2018 das internações para intervenções cardiovasculares pagas pelo SUS de 2008 a 2018. Dados expressos em milhares (1.000)

	2008		2009		2010		2011		2012		2013		2014		2015		2016		2017		2018		TOTAL		
	R$	Int$ 2018	R$	Int$ 2018	R$	Int$ 2018	R$	Int$ 2018	R$	Int$ 2018	R$	Int$ 2018	R$	Int$ 2018	R$	Int$ 2018	R$	Int$ 2018	R$	Int$ 2018	R$	Int$ 2018	R$	R$ + IPCA	Int$ 2018
Ablação de fibrilação atrial	360	348	378	339	471	390	457	349	690	489	788	520	771	471	906	515	707	372	691	351	732	361	6.951	11.430	5.636
Angioplastia coronariana	210.529	202.991	266.654	239.681	295.641	245.024	337.972	258.502	372.063	263.613	388.920	256.381	411.252	251.355	412.073	234.147	433.590	227.946	466.696	237.091	495.885	244.519	4.091.276	6.728.694	3.317.896
CRVM	176.032	169.730	208.585	187.486	214.484	177.762	287.851	220.167	297.844	211.027	290.541	191.528	294.854	180.213	289.638	164.577	286.160	150.439	282.175	143.351	275.110	135.656	2.903.274	4.774.145	2.354.114
Cirurgia valvar	125.954	121.445	140.684	126.453	142.383	118.006	179.111	136.995	183.271	129.851	178.564	117.711	180.088	110.069	176.814	100.469	175.319	92.168	176.134	89.481	177.584	87.566	1.835.908	3.018.967	1.488.643
Angioplastia primária	45.267	43.647	37.888	34.055	37.113	30.759	35.577	27.211	35.545	25.185	37.288	24.581	45.883	28.044	56.101	31.877	66.515	34.968	71.624	36.387	73.429	36.208	542.231	891.645	439.667
Cardiomiopatias	168.993	163	545	490	192	159	326	249	436	309	354	233	306	187	298	169	527	277	452	230	426	210	4.031	6.623	3.266
Outras valvuloplastias	1.519	1.464	1.662	1.493	1.718	1.423	1.919	1.468	1.871	1.325	2.052	1.352	2.128	1.301	2.086	1.185	1.594	838	1.889	960	1.690	833	20.126	33.095	16.319
Valvuloplastia mitral	3.115	3.004	3.585	3.223	3.147	2.608	3.228	2.469	2.718	1.926	2.970	1.958	2.809	1.717	2.394	1.360	1.378	724	1.721	874	1.462	721	28.525	46.907	23.130
**Total**	**562.946**	**542.790**	**659.980**	**593.221**	**695.149**	**576.132**	**846.441**	**647.411**	**894.438**	**633.724**	**901.476**	**594.265**	**938.091**	**573.356**	**940.309**	**534.300**	**965.790**	**507.732**	**1.001.383**	**508.723**	**1.026.318**	**506.074**	**9.432.322**	**15.510.510**	**7.648.180**

*CRVM: cirurgia de revascularização do miocárdio.* *Fonte: Sistema de Informações sobre Mortalidade – SIM/DATASUS. ^29^*

##  2. ACIDENTE VASCULAR CEREBRAL (DOENÇAS CEREBROVASCULARES) 

### CID-9 430 a 438; CID-10 I60 a I69

Ver Tabelas 2-1 a 2-10 e Figuras 2-1 a 2-4

**Table:** 

Abreviaturas usadas no Capítulo 2	
AIT	Ataque Isquêmico Transitório
AVCH	Acidente Vascular Cerebral Hemorrágico
CID	Classificação Estatística Internacional de Doenças e Problemas Relacionados à Saúde
CID-9	Classificação Estatística Internacional de Doenças e Problemas Relacionados à Saúde, 9a Revisão
CID-10	Classificação Estatística Internacional de Doenças e Problemas Relacionados à Saúde, 10a Revisão
CIF	Classificação Internacional de Funcionalidade, Incapacidade e Saúde
DAC	Doença Arterial Coronariana
DALYs	Anos de vida perdidos ajustados por incapacidade (do inglês, *Disability-Adjusted Life-Year* )
DCV	Doença Cardiovascular
DP	Desvio-Padrão
GBD	*Global Burden of Disease*
GWTG-S	Programa ‘ *Get With The Guidelines-Stroke* ’
HR	*Hazard Ratio*
HSA	Hemorragia Subaracnóidea
IBGE	Instituto Brasileiro de Geografia e Estatística
IC	Intervalo de Confiança
IECA/BRA	Inibidor da Enzima de Conversão da Angiotensina/ Bloqueador do Receptor de Angiotensina
II	Intervalo de Incerteza
JCI	*Joint Commission International *
LDL	Colesterol de baixa densidade (do inglês, *Low-Density Lipoprotein* )
NCEP	* National Cholesterol Education Program Adult Treatment Panel *
NIHSS	Escala do *National Institutes of Health* para avaliar AVC (do inglês, *National Institutes of Health Stroke Scale* )
OMS	Organização Mundial da Saúde
OR	*Odds Ratio*
PNS	Pesquisa Nacional de Saúde
PSF	Programa Saúde da Família
QALY	*Quality-Adjusted Life-Year*
rtPA	Ativador do Plasminogênio Tecidual Recombinante
SIH	Sistema de Informações Hospitalares
SIM	Sistema de Informações sobre Mortalidade
SSQOL	Escala Específica de Qualidade de Vida no AVC (em inglês, *Stroke Specific Quality of Life Scale* )
SUS	Sistema Único de Saúde

### Prevalência

- As estimativas de prevalência de AVC podem diferir levemente entre os estudos, pois cada um seleciona e recruta uma amostra de participantes para representar sua população-alvo (estado, região ou país).
- Em um estudo de base comunitária no Brasil, que aplicou um questionário para 4.496 indivíduos com idade superior a 35 anos, residentes de uma área carente da cidade de São Paulo em 2011, Abe *et al* . identificaram, em uma triagem inicial, 243 indivíduos positivos para AVC. A taxa de prevalência padronizada por idade para homens foi 4,6% (IC 95%, 3,5 - 5,7) e, para mulheres, 6,5% (IC 95%, 5,5 - 7,5). ^67^
- Em um estudo transversal de base populacional que incluiu 3.391 indivíduos com idade igual ou superior a 20 anos, realizado em Porto Alegre, sul do Brasil, de julho a dezembro de 2009, com amostragem sistemática, Copstein *et al* . encontraram 285 indivíduos que relatavam diagnóstico ou sintomas consistentes com AVC prévio (8,4% da amostra). ^68^
- Usando uma ferramenta de rastreio, um questionário de sintomas de AVC, Fernandes *et al* . estudaram a prevalência de AVC na cidade de Coari, na Bacia Amazônica Brasileira, e compararam essa prevalência entre ribeirinhos e a população urbana do mesmo município. Em 4.897 respondentes da área urbana e 1.028 da rural, os autores encontraram uma prevalência bruta de AVC de 6,3% na área rural e de 3,7% na urbana, com diferenças mantidas após ajuste para sexo e idade. ^69^
- Pereira *et al* . conduziram um estudo para estimar a prevalência de AVC entre idosos na cidade de Vassouras, no Rio de Janeiro, em 2007, usando dados do Sistema de Informação da Atenção Básica, do censo populacional conduzido pelo IBGE e da ficha de atendimento padronizada pelo PSF do Ministério da Saúde. A qualidade dos diagnósticos de AVC do PSF foi analisada. De 4.154 idosos rastreados, o estudo detectou 122 com diagnóstico de AVC (prevalência, 2,9%; homens, 3,2%; mulheres, 2,7%) e aumento progressivo com o avançar da idade. A taxa de prevalência foi a mesma nas áreas rural e urbana do município (2,9%). ^70^
- Utilizando o *Stepwise Approach to Stroke Surveillance* da OMS, Goulart *et al* . realizaram um estudo para verificar as taxas de mortalidade e morbidade por AVC em uma área de São Paulo. O questionário para determinar a prevalência de AVC foi aplicado de porta em porta em uma vizinhança do PSF (etapa 3). Dos 3.577 indivíduos com mais de 35 anos avaliados em casa, foram identificados 244 (6,8%) sobreviventes de AVC através do questionário validado por um neurologista certificado. ^71^
- Benseñor *et al* ., analisando um inquérito epidemiológico de base domiciliar (PNS - 2013) com uma amostra representativa nacional, avaliaram o número absoluto de indivíduos com AVC e com incapacidade por AVC, com as respectivas prevalências. Foram estimados 2.231.000 indivíduos com AVC e 568.000 com incapacidade grave por AVC. As prevalências pontuais de AVC foram 1,6% e 1,4% para homens e mulheres, respectivamente. ^72^
- De acordo com o Estudo GBD 2017, a prevalência de AVC padronizada por idade diminuiu 44,2% (II 95%, -41,7 a -46,9) no Brasil, passando de 1.810,9 (II 95%, 1.530,9 - 2.131,5) em 1990 para 1.010,6 (II 95%, 843,2 - 1.197,8) em 2017. ^7^ A prevalência padronizada por idade diminuiu similarmente entre homens e mulheres, mas foi maior entre os homens em todo o período ( Tabela 2-1 ). A Tabela 2-1 também mostra as taxas de prevalência padronizada por idade de AVC no Brasil e em suas unidades federativas.
- Dados do estudo ELSA-Brasil, uma coorte de seis centros com funcionários públicos e incluindo 15.105 adultos (45,8% homens; idade variando de 35 a 74 anos), mostraram prevalência de AVC de 1,3% para homens e mulheres. ^73^

### Incidência

#### Subtipos de AVC

- Em um estudo de base hospitalar realizado na região de Fortaleza, no nordeste do Brasil, AVC isquêmico foi o subtipo mais frequente (72,9%), seguido por hemorragia intracerebral (15,2%), HSA (6,0%), AIT (3,0%) e AVC indeterminado (2,9%). ^74^ A distribuição é similar àquela do estudo de base comunitária conduzido de 2005 a 2006 na cidade de Joinville, no sul do Brasil, onde, dos 759 primeiros episódios de AVC, 610 (80,3%) foram isquêmicos, 94 (12,3%) foram hemorrágicos e 55 (7,2%) foram HSA. No estudo de Joinville, a incidência anual por 100 mil pessoas-ano foi 61,8 (IC 95%, 57,0 - 66,9) para AVC isquêmico, 9,5 (5% CI, 7,7 - 11,6) para AVCH e 5,6 (IC 95%, 4,2 - 7,3) para HSA. A incidência de AVC ajustada para a população mundial por 1.000 habitantes com idade acima de 55 anos foi 5,8 (IC 95%, 5,4 - 6,2). A incidência de infarto foi 4,7 (IC 95%, 4,3 - 5,1), de hemorragia intracerebral, 0,6 (IC 95%, 0,5 - 0,8), e de HSA, 0,3 (IC 95%, 0,2 - 0,4). ^75^
- Em estudo de base populacional conduzido em Matão, no sudeste do Brasil, de 2003 a 2004, a incidência anual bruta por 100 mil por ano foi 108 (IC 95%, 85,7 - 134,1) e a taxa ajustada por idade e sexo, usando a População Padrão de Segi, foi 137 (IC 95%, 112,0 - 166,4) por 100 mil habitantes por ano. Acidente vascular cerebral isquêmico ocorreu em 69 (85,2%) indivíduos, hemorragia intracerebral, em 11 (13,6%) e HSA, em 1 (1,2%). ^76^
- Dados do estudo de base comunitária de Joinville mostraram que, ao comparar diferentes períodos (1995, 2005-2006, 2010-2011 e 2012-2013), a incidência de AVC diminuiu. Nos últimos 18 anos, a incidência de AVC geral (todos os tipos principais de AVC) em Joinville diminuiu em 37% (IC 95%, 32 - 42). ^75^ A incidência de primeiro episódio de AVC ajustada para a população brasileira foi 86,6 por 100 mil (IC 95%, 80,5 - 93,0) em 2005-2006 e 113,46 por 100 mil (IC 95%, 101,5 - 126,8) em 1995. ^77^ A incidência geral ajustada por idade e para a população mundial por 100 mil pessoas-ano foi 143,7 (IC 95%, 128,4 - 160,3) em 1995, caindo para 105,4 (IC 95%, 98,0 - 113,2) em 2005-2006 e para 90,9 (IC 95%, 85,1 - 96,9) em 2012-2013. A incidência padronizada por idade de primeiro episódio de AVC estratificada por gênero e idade também caiu significativamente ao longo do tempo. A redução foi 11% maior nos homens (42%; IC 95%, 35 - 49) do que nas mulheres (31%; IC 95%, 23 - 39), e 16% maior nos jovens (≤ 44 anos: 54%; IC 95%, 41 – 66; > 44 anos: 38%; IC 95%, 33 - 43). De 1995 a 2013, a proporção de AVC isquêmico aumentou 12%, enquanto a de AVCH diminuiu 16%. Entretanto, a proporção de HSA permaneceu relativamente estável, variando de 7,5% em 1995 a 6% em 2012-2013. O peso da diminuição na incidência de AVC padronizada por idade foi proporcionalmente maior para AVCH do que para AVC isquêmico, enquanto o de HSA permaneceu estável. Nos últimos 8 anos, as incidências de AVC isquêmico e de AVCH apresentaram reduções absolutas significativas de 15% (IC 95%, 1,00 - 28,00) e de 60% (IC 95%, 13,00 - 86,00), respectivamente. Entretanto, a incidência de HSA apresentou redução absoluta não significativa de 29% (IC 95%, 15,00 - 92,00). ^78^
- Um estudo incluindo 213 pacientes consecutivos com cardiomiopatia chagásica no Brasil explorou o risco cumulativo de AVC e AIT de longo prazo e suas relações com disfunção ventricular esquerda naqueles pacientes de junho de 1999 a janeiro de 2007. Após um seguimento médio de 36 meses, a incidência geral de AVC isquêmico foi 2,67 eventos por 100 pacientes/ano. Os fatores de risco independentes para AVC e AIT incluíram fração de ejeção de ventrículo esquerdo (HR 0,95; IC 95%, 0,91 - 0,99, p=0,009) e volume atrial esquerdo corrigido para área de superfície corporal (HR 1,04; IC 95%, 1,01 - 1,07, p=0,007), que persistiram após ajuste para uso de anticoagulação. ^79^

### Mortalidade

- Dados do Registro de AVC de Joinville (n=759 casos de primeiro AVC) mostraram que a taxa de mortalidade ajustada para a população brasileira no período 2005-2006 foi de 20,5 por 100 mil (IC 95%, 17,5 - 23,8) e, quando ajustada para a população mundial, 23,9 por 100 mil (IC 95%, 20,4 - 27,8), revelando uma tendência decrescente a partir de 1995. Houve ainda uma redução na mortalidade ajustada por idade, embora muito mais acentuada nos homens (48%) do que nas mulheres (3%). A taxa de letalidade foi 19,1% (145/759) no período 2005-2006, que é também menor do que a de 1995 [26,6% (84/320)]. Portanto, em aproximadamente 10 anos, a mortalidade caiu 37%. A taxa de letalidade de 30 dias diminuiu 28,2% no período (de 26,6% para 7,5%). ^77 ,^ ^80^
- Em um estudo de base populacional realizado em Matão, na região sudeste (n=141), a taxa de letalidade geral de 30 dias foi 18,5% (IC 95%, 10,7 - 28,7%). Quanto aos subtipos de AVC, as taxas de letalidade de 30 dias foram 13% (IC 95%, 6,1 - 23,3%) para AVC isquêmico e 45,4% (IC 95%, 16,7 - 76,2%; p=0,02) para AVCH. A taxa de letalidade geral de 1 ano foi 30,9% (IC 95%, 21,1 - 42,1%). Quanto aos subtipos de AVC, as taxas de letalidade de 1 ano foram 24,6% (IC 95%, 23,7 - 47,2%) para AVC isquêmico e 63,6% (IC 95%, 30,7 - 89,0%; p=0,01) para AVCH. ^76^
- Dados do Estudo GBD 2017 mostraram taxas de mortalidade por AVC padronizadas por idade por 100 mil de 122,9 (II 95%, 120,6 - 125) em 1990 e de 56,6 (II 95%, 55,2 - 57,8) em 2017, sendo a variação percentual de -54 (II 95%, -55,1 a -53) ( Figura 2-1 e Tabela 2-4 ). A maior variação percentual ocorreu no Espírito Santo, -68,3 (II 95%, -69,9 a -66,5), e a menor, no Maranhão, -31,7 (II 95%, -36,6 a -26,6) ( Tabela 2-2 ). Para os adultos, a maior variação percentual foi observada no grupo etário 50-69 anos, -56 (II 95%, -57,5 a -54,5) ( Tabela 2-4 ).
  **Figura 2-1:**
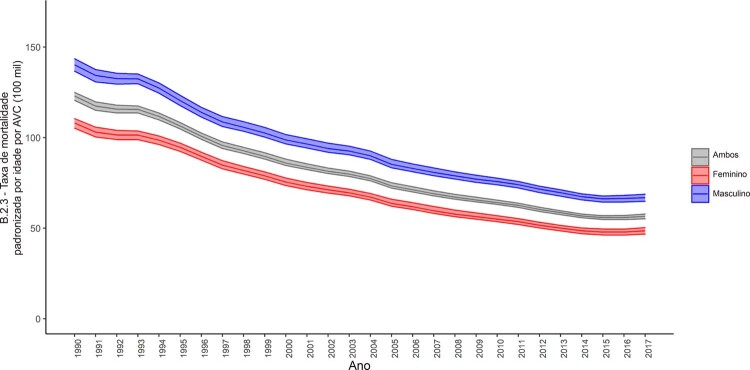
- Taxa de mortalidade padronizada por idade por AVC (100 mil habitantes), 1990-2017.

Dados derivados do Estudo Global Burden of Disease 2017 (GBD 2017). ^109^
- Quanto aos subtipos de AVC, dados do Estudo GBD 2017 revelaram taxas de mortalidade por AVC isquêmico padronizadas por idade por 100 mil de 54,8 (II 95%, 53,6 - 55,9) em 1990 e de 22,6 (II 95%, 21,9 - 23,2) em 2017, representando uma variação percentual de -58,7 (II 95%, -60 a -57,4) ( Figura 2-2 e Tabela 2-2 ). Para os adultos, a maior variação percentual foi observada no grupo etário 50-69 anos, -63,7 (II 95%, -65,8 a -61,6) ( Tabela 2-4 ).
  **Figura 2-2:**
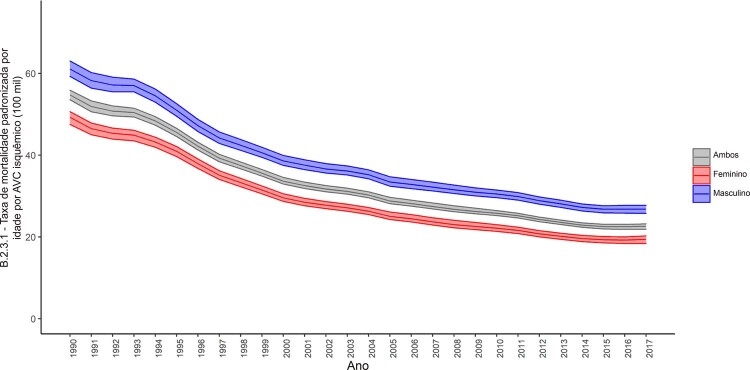
- Taxa de mortalidade padronizada por idade por AVC isquêmico (100 mil habitantes), 1990-2017.

Dados derivados do Estudo Global Burden of Disease 2017 (GBD 2017). ^109^
- Segundo o Estudo GBD 2017, para hemorragia intracerebral, as taxas de mortalidade padronizadas por idade por 100 mil foram 58,6 (II 95%, 57,3 - 59,9) e 27,9 (II 95%, 27,1 - 28,7) em 1990 e 2017, respectivamente, representando uma variação percentual de -52,4 (II 95%, -53,8 a -51,1) ( Figura 2-3 e Tabela 2-4 ). Para os adultos, a maior variação percentual foi observada no grupo etário 15-49 anos, -57,6 (II 95%, -60,9 a -55,4) ( Tabela 2-4 ).
  **Figura 2-3:**
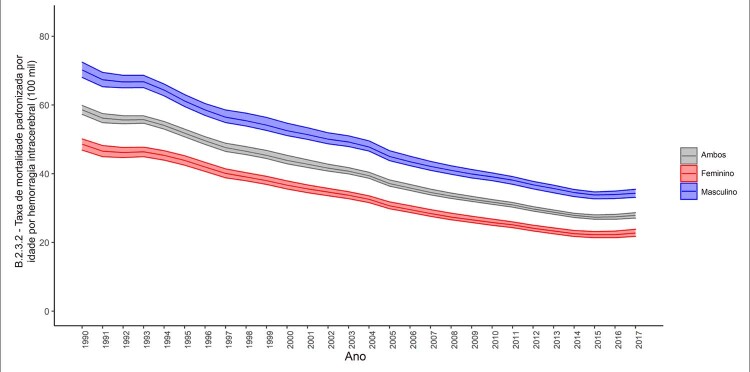
- Taxa de mortalidade padronizada por idade por hemorragia intracerebral (100 mil habitantes), 1990-2017.

Dados derivados do Estudo Global Burden of Disease 2017 (GBD 2017). ^109^
- Segundo o Estudo GBD 2017, para HSA, as taxas de mortalidade padronizadas por idade por 100 mil foram 9,6 (II 95%, 8,8 - 9,9) e 6,1 (II 95%, 5,0 - 6,7) em 1990 e 2017, respectivamente, representando uma variação percentual de -36,5 (II 95%, -39,8 a -32,2) ( Figura 2-4 e Tabela 2-4 ). Para os adultos, a maior variação percentual foi observada no grupo etário 15-49 anos, -39,4 (II 95%, -43,6 a -29,4) ( Tabela 2-4 ).
  **Figura 2-4:**
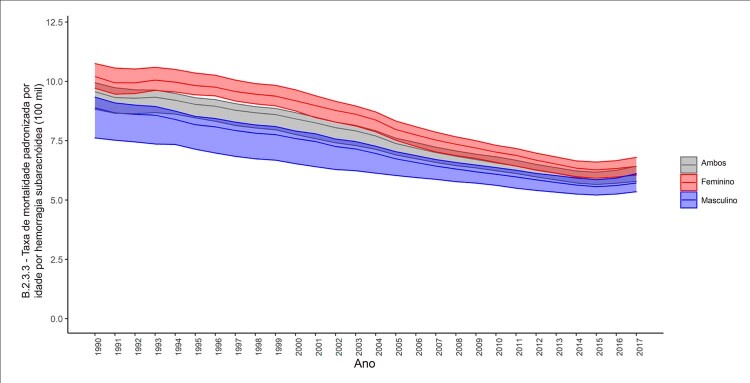
- Taxa de mortalidade padronizada por idade por hemorragia subaracnóidea (100 mil habitantes), 1990-2017.

Dados derivados do Estudo Global Burden of Disease 2017 (GBD 2017). ^109^

**Figura 3-1:**
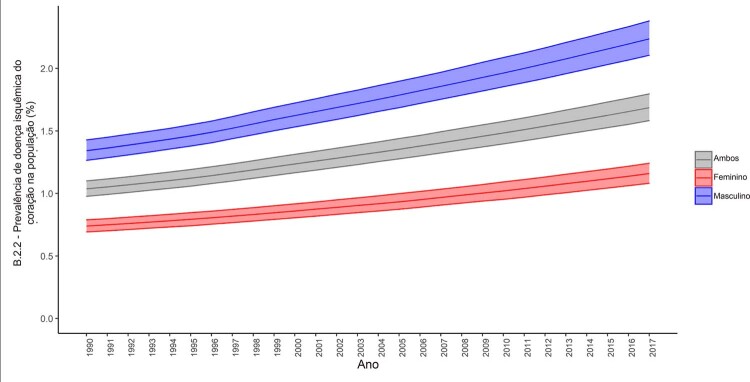
- Prevalência de doença isquêmica do coração no Brasil, por sexo, porcentagem (1990-2017).

Dados derivados do Estudo Global Burden of Disease 2017 (GBD 2017). ^151^

**Figura 3-2:**
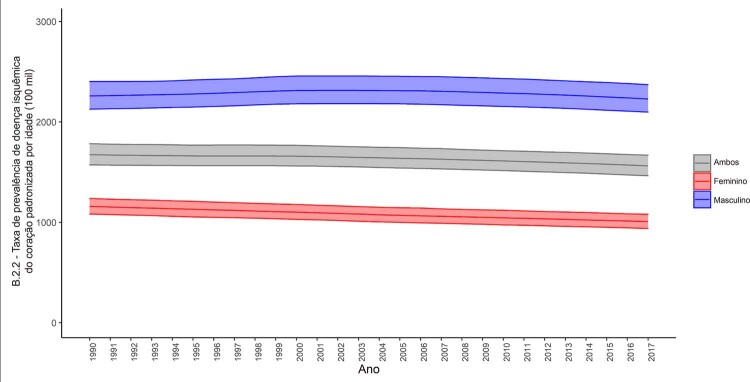
- Taxa de prevalência de doença isquêmica do coração padronizada por idade por 100 mil habitantes no Brasil por sexo (1990-2017).

Dados derivados do Estudo Global Burden of Disease 2017 (GBD 2017). ^151^
- Analisando as estimativas do GBD 2015 em 27 unidades federativas brasileiras entre 1990 e 2015, Lotufo *et al.* mostraram que, apesar do aumento no número absoluto de mortes por doença cerebrovascular, a proporção de mortes antes dos 70 anos de idade foi reduzida à metade entre 1990 e 2015. Nesse período, o risco de morte atribuível a AVC decresceu tanto para homens (-2,41% por ano) quanto para mulheres (-2,51% por ano). No entanto, a redução anual nas taxas de mortalidade ajustadas para idade, para ambos os sexos, desacelerou entre 2005 e 2015 quando comparada ao período de 1990-2005. Estados com índice de desenvolvimento social no tercil inferior apresentaram reduções anuais menos significativas para homens e mulheres (-1,23% e -1,84%, respectivamente) quando comparados àqueles com índice no tercil médio (-1,94 e -2,22%, respectivamente) e àqueles com índice no tercil superior (-2,85 e -2,82%, respectivamente). Além disso, houve diminuição nos anos vividos com incapacidade nos estados, mas de maneira menos expressiva. ^81^
- André *et al.* , usando dados do SIM corrigido para mortes por causas mal definidas, mostram que as taxas de mortalidade por AVC padronizadas por idade decresceram consistentemente entre 1980-1982 e 2000-2002, passando de 68,2 para 40,9 por 100 mil habitantes. Durante o mesmo período, as taxas de mortalidade cardiovascular total também diminuíram significativamente, passando de 208,2 para 126,1 por 100 mil habitantes. O declínio na taxa de mortalidade por AVC padronizada por idade foi evidente nas duas décadas, sendo maior entre 1990-1992 e 2000-2002. Tomando as taxas de 1980 como referência, houve uma redução de risco de 30% (IC 95%, 30% - 31%) em 1990 e de 55% (IC 95%, 55% - 56%) em 2000 (P<0,001 para ambas as medidas). A redução proporcional na mortalidade por AVC foi evidente para homens e mulheres, ainda que mais acentuada para os homens. Além disso, a diminuição foi observada em todas as faixas etárias. Detectou-se uma interação entre sexo e idade, com declínio mais acentuado nas taxas de mortalidade padronizadas por idade na população masculina jovem (até 45 anos) e declínio mais abrupto para as mulheres de todas as faixas etárias (P<0,001 para todos os achados). A redução nas taxas de mortalidade por AVC padronizadas por idade ocorreu em todas as regiões geopolíticas. Detectou-se uma interação entre a região estudada e a magnitude da redução. As regiões mais ricas (Sul e Sudeste) exibiram taxas iniciais mais altas e reduções mais marcadas durante o período do estudo. Esses achados foram confirmados pelo modelo de regressão de Poisson, no qual a redução menos marcante na taxa de mortalidade por AVC padronizada foi observada na região Nordeste, 41% (IC 95%, 40% - 42%). Os valores correspondentes para as outras regiões foram: Norte, 52% (IC 95%, 51% - 52%); Centro-Oeste, 53% (IC 95%, 53% - 54%); Sul, 57% (IC 95%, 56% - 57%); e Sudeste, 59% (IC 95%, 58% - 59%). O número total de mortes relacionadas a AVC no Brasil, no entanto, vem aumentando consistentemente nas últimas 3 décadas. O número anual médio de mortes atribuíveis a AVC aumentou de 79.862, em 1980-1982, para 101.625, em 2000-2002. Tendência semelhante foi evidenciada na mortalidade cardiovascular total: 239.876 mortes em 1980-1982 e 311.138 em 2000-2002. Esse aumento reflete principalmente o envelhecimento progressivo da população brasileira. ^82^
- Em outra avaliação das tendências da mortalidade por AVC no Brasil de 1979 a 2009, após a exclusão das mortes devidas às sequelas de AVC, para os homens, as variações percentuais anuais (IC 95%) foram: 1979-1984, 0,7 (-0,8 a 2,1); 1984-1994, -1,8 (-2,4 a -1,2); 1994-2007, -5,0 (-5,4 a -4,7); e 2007-2009, -0,8 (-7,0 a 5,8). Para as mulheres, as variações percentuais anuais (IC 95%) foram: 1979-1994, -1,9 (-2,2 a -1,6); 1994-1997, -7,5 (-14,0 a -0,6); 1997-2007, -4,0 (-4,6 a -3,3); e 2007-2009, 1,6 (-5,5 a 9,2). No período 2006-2009, a média da variação percentual anual (IC 95%) para todos os AVC foi -3,1 (-3,3 a -2,9) para os homens e -2,9 (-3,1 a -2,8) para as mulheres. No mesmo período, a média da variação percentual anual das taxas de morte pelos subtipos de AVC foram, para homens e mulheres, respectivamente: hemorragia intracerebral, -4,0 (-4,9 a -3,1) e -2,9 (-3,4 a -2,3); e AVC isquêmico, -3,2 (-3,3 a -3,0) e -1,4 (-2,0 a -0,9). ^83^
- Uma avaliação considerando a realocação dos óbitos sem registro de sexo ou idade, a redistribuição de ‘códigos *garbage* ’ e a correção de subnotificação mostrou as seguintes taxas de mortalidade por AVC para 1996 e 2011, ajustadas por idade, antes e depois de correção, respectivamente: 1) para homens: em 1996, 82,9 e 113,6; e, em 2011, 49,6 e 60,9; e 2) para mulheres: em 1996, 58,2 e 84,4; e, em 2011, 34,7 e 42,3. ^84^
- Um estudo avaliando diferenças regionais na transição de mortalidade e utilizando dados do SIM de 1990 a 2012 mostrou uma variação de -48,05% no coeficiente de mortalidade por AVC. A maioria das regiões apresentou redução nas taxas de mortalidade padronizadas por idade: -62% no Sudeste; -55,5% no Sul; -26,91% no Centro-Oeste; e -20,8% no Norte. Apenas no Nordeste ocorreu aumento (13,77%). ^85^
- Na cidade de São Paulo, de 1996 a 2011, 77.848 óbitos por AVC foram confirmados, 51,4% dos quais entre indivíduos com 35-74 anos de idade. Naquele período, taxas de mortalidade ajustadas por idade por doenças cerebrovasculares diminuíram 46,6% nos homens e 47,8% nas mulheres. Para os homens nas áreas de maior renda, a tendência decrescente foi constante; na área de renda média, houve um declínio marcado de 1996 a 2000, seguido por um de menor velocidade entre 2000 e 2011. Nas áreas de renda mais baixa, a variação percentual anual foi maior entre 1996 e 2002, com discreto declínio entre 2002 e 2011. Para as mulheres nas áreas de alta renda, houve um declínio marcado de 1996 a 2003, que foi menor na segunda metade do período; nas áreas de renda baixa e média, o declínio foi constante em todos os períodos. Para todo o período, ambos os sexos e grupo etário de 35-74 anos, a diminuição nas taxas ajustadas por idade foi mais pronunciado entre os residentes da área de maior renda em comparação àqueles da área de menor renda. Esse mesmo padrão, mas com diferente magnitude de declínio, foi observado nos indivíduos com idade ≥75 anos em todas as áreas ao se comparar aos outros grupos etários, para os dois sexos. Além disso, a evolução temporal das razões entre as taxas ajustadas por idade de indivíduos de 35-74 anos vivendo em áreas de renda baixa e alta foi: para homens, de 1996 a 1998, a razão das taxas foi 2,03, e, de 2009 a 2011, 2,34. Para as mulheres, de 1996 a 1998, a razão das taxas foi 2,09, e, de 2009 a 2011, 2,58. A tendência das razões entre as taxas ajustadas por idade dessas áreas mostrou um crescimento da variação percentual anual de 1,4 (0,5 - 2,4) para os homens e de 1,1 (0,1 - 2,0) para as mulheres. ^86^
- Em estudo de base hospitalar realizado nas regiões Nordeste e Sudeste (n=962), as taxas de letalidade geral para 10 dias e 28 dias foram 7,9 (IC 95%, 6,2 - 9,7) e 12,5 (IC 95%, 10,4 - 14,5), respectivamente. As taxas de morte por AVCH foram maiores do que aquelas por AVC isquêmico tanto para 10 dias (12,3[IC 95%, 7,2 - 14,4] vs. 7,0[IC 95%, 5,3 - 8,8]) quanto para 28 dias (19.8[IC 95%, 13,6 - 26,0] vs. 11,1[IC 95%, 8,9 - 13,3]). Além de idade avançada, os fatores de risco para letalidade por AVC isquêmico aos 28 dias foram diabetes (OR=1,69; IC 95%, 1,06 - 2,68) e doença cardíaca prévia (OR=1,86; IC 95%, 1,17 - 2,96) após ajuste por idade. ^87^

### Carga de Doença

- Dados do Estudo GBD 2017 mostraram que as taxas de DALYs por AVC padronizadas por idade por 100 mil foram, em 1990, 2.511,9 (II 95%, 2.457,3 - 2.567,6) e, em 2017, 1.145,3 (1.107,8 - 1.185,3), representando uma variação percentual de -54,4 (II 95%, -55,5 a -53,2) ( Tabela 2-6 ). Para homens, a variação percentual foi -54 (II 95%, -55,4 a -52,5) e, para mulheres, -54,2 (II 95%, -55,9 a -52,7) ( Tabelas 2-9 e 2-10 ). A maior variação percentual ocorreu no Espírito Santo, -64,7 (II 95%, -66,5 a -62,8), e a menor, no Amapá, -29,5 (-33,8 a -25,2) ( Tabela 2-5 ).
- As taxas de DALYs por AVC isquêmico padronizadas por idade por 100 mil foram 871,4 (II 95%, 841,1 - 902,3) e 387,3 (II 95%, 363,9 - 411,5) em 1990 e 2017, respectivamente, representando variação percentual de -55,6 (II 95%, 55,5 a -53,2) ( Tabela 2-6 ). Para adultos, a maior variação percentual foi observada entre indivíduos de 50-69 anos, -58,7 (II 95%, -60,9 a -56,4) ( Tabela 2-6 ), sendo -58,3 (II 95%, -61,1 a -55,6) para homens e -58,7 (II 95%, -61,7 a -55,6) para mulheres ( Tabelas 2-9 e 2-10 ).
- As taxas de DALYs por hemorragia intracerebral padronizadas por idade por 100 mil foram 1.322,1 (II 95%, 1.291,8 - 1.358,2) em 1990 e 576,9 (II 95%, 560,7 - 594,9) em 2017, representando variação percentual de -56,4 (II 95%, -57,8 a -55,1) ( Tabela 2-6 ). Para adultos, a maior variação percentual foi observada em indivíduos de 15-49 anos, -57,9 (II 95%, -61,3 a -55,8) ( Tabela 2-6 ), sendo -60,1 (II 95%, -64,4 a -57,1) para homens e -57,1 (II 95%, -59,8 a -54,7) para mulheres ( Tabelas 2-9 e 2-10 ).
- As taxas de DALYs por HSA padronizadas por idade por 100 mil foram 318,4 (II 95%, 287 - 332,2) em 1990 e 181,0 (II 95%, 173,1 - 191,0) em 2017, representando variação percentual de -43,1 (II 95%, -46,3 a -37,2) ( Tabela 2-6 ). Para adultos, a maior variação percentual foi observada entre indivíduos de 15-49 anos, -40 (II 95%, -43,9 a -30,9), sendo -44,2 (II 95%, -49,5 a -22,9) para homens e -36,8 (II 95%, -41,6 a -31,8) para mulheres ( Tabelas 2-9 e 2-10 ).

### Complicações

- Benseñor *et al* . analisaram um inquérito epidemiológico de base domiciliar (PNS - 2013), com uma amostra representativa nacional, para avaliar o número absoluto e as taxas de prevalência de AVC e de incapacidade por AVC. Foram estimados 2.231.000 indivíduos com AVC e 568.000 com incapacidade grave por AVC. As prevalências pontuais de AVC foram 1,6% e 1,4% para homens e mulheres, respectivamente. A prevalência de incapacidade por AVC foi 29,5% para homens e 21,5% para mulheres. As taxas de prevalência de AVC aumentaram com a idade, o baixo nível educacional e nos residentes de áreas urbanas, mas não apresentaram diferença de acordo com a raça autorreferida. O grau de incapacidade por AVC não diferiu estatisticamente de acordo com sexo, raça, nível educacional ou local de residência. ^72^
- Carvalho-Pinto *et al.* , conduzindo estudo observacional retrospectivo, coletaram dados dos prontuários médicos e de visitas domiciliares após AVC de pacientes seguidos em uma unidade de atenção primária em Belo Horizonte, entre maio de 2013 e maio de 2014. Os dados incluíram condição de saúde, assistência recebida após AVC, fatores pessoais e ambientais, funcionalidade e incapacidade organizados de acordo com a estrutura conceitual da CIF. A maioria dos participantes apresentou boa percepção da própria habilidade manual (2,39 [DP, 2,29] logits) e limitada habilidade para caminhar (88%), sendo capaz de melhorar a velocidade da marcha natural, apresentou mudança de equilíbrio (51,43%) e mobilidade funcional (54,16%) com risco de queda, e teve percepção negativa da própria qualidade de vida (escore médio de 164,21 [DP, 35,16] pontos na SSQOL-Brasil). ^88^
- De acordo com o Estudo GBD 2016, a maior porcentagem de óbitos por AVC em geral ocorreu em indivíduos com 70 anos ou mais (60,2%; II 95%, 59,9-60,5%) e em homens (52,9%; II 95%, 52,6-53,2%). O AVC isquêmico foi o tipo mais comum, sendo responsável por 61,8% (II 95%, 61,5-62,1%) dos óbitos por AVC em 2016. A maioria dos indicadores epidemiológicos de AVC em geral ou de um subtipo de AVC (incidência, prevalência, razão mortalidade/incidência, mortalidade, DALYs, anos perdidos por incapacidade e anos de vida perdidos) foi maior nos homens e nos indivíduos com 70 anos ou mais. ^89^

### Utilização e Custo da Atenção à Saúde

#### (Tabelas 1-6 a 1-9 e Figuras 1-15 a 1-16)

##### Admissões Hospitalares

- Lopes *et al* . conduziram um estudo ecológico desenhado com abordagem analítica e coleta de dados do SIH sobre episódios de AVC no período de 1998-2012. Todos os dados foram estratificados por sexo e idade, criando um indicador para internação hospitalar relacionada a AVC. Os autores observaram redução de 73,64% nas internações hospitalares relacionadas a AVC, que passaram de 37,56/105 habitantes em 1998-2001 para 10,33/105 habitantes em 2002-2005. Essa redução ocorreu nos dois sexos e em todos os grupos etários. ^90^
- Em uma análise de série temporal, Katz *et al* . avaliaram a relação entre taxa de desemprego relacionada a AVC e internação hospitalar no Brasil em um período recente de 11 anos. Dados sobre hospitalizações mensais por AVC de março de 2002 a dezembro de 2013 foram extraídos da base de dados do Sistema Público de Saúde Brasileiro, revelando 1.581.675 internações por AVC no período. A taxa de desemprego diminuiu de 12,9% em 2002 para 4,3% em 2013, enquanto as internações por AVC aumentaram. Entretanto, o modelo ajustado não mostrou associação positiva entre taxa de desemprego e internação por AVC (coeficiente estimado = 2,40 ± 4,34; p=0,58). ^91^
- Utilizando dados do SIH, do SIM e do IBGE, Adami *et al* . analisaram taxas de mortalidade e incidência de hospitalizações relacionadas a AVC em brasileiros com 15-49 anos, por região e grupo etário, entre 2008 e 2012. Definiu-se AVC de acordo com a CID-10 (I60-I64). Mortalidade bruta e padronizada (OMS) e incidência de hospitalizações por 100 mil habitantes, estratificadas por região e grupo etário, foram estimadas. Os autores relataram 131.344 internações por AVC em brasileiros com 15-49 anos entre 2008 e 2012. No mesmo período, a taxa de hospitalizações estabilizou: 24,67 (IC 95%, 24,66 - 24,67) em 2008 e 25,11 (IC 95%, 25,10 - 25,11) em 2012 (β = 0,09, p = 0,692, r2 = 0,05). ^92^
- Dantas *et al* . realizaram um estudo para avaliar hospitalizações públicas por AVC no Brasil de 2009 a 2016. Aqueles autores selecionaram registros de hospitalização de acordo com os códigos de diagnóstico de AVC da CID-10. De 2009 a 2016, o número de internações subiu de 131.122 para 146.950, tendo o número absoluto de mortes hospitalares aumentado de 28.731 para 31.937. Idade mais jovem e sexo masculino mostraram associação significativa com a sobrevida do paciente. As taxas anuais de hospitalização e de mortalidade hospitalar ajustadas por idade caíram 11,8% e 12,6%, respectivamente, mas a taxa de letalidade aumentou para pacientes acima de 70 anos. ^93^

Utilização da Atenção à Saúde

- Uma análise da tendência de expansão da cobertura do PSF e internação por condições sensíveis à atenção primária no Rio de Janeiro, entre 1998 e 2015, mostrou uma queda de 7,6% nas hospitalizações por doenças cerebrovasculares. ^94^
- Um estudo realizado em Joinville avaliou o impacto de uma unidade dedicada a AVC, a primeira estabelecida no Brasil, na fase aguda do manejo de AVC em comparação ao tratamento convencional em enfermaria geral. O estudo avaliou 35 e 39 pacientes alocados em unidade de AVC e em enfermaria geral, respectivamente, em 2000, mostrando mortalidade em 10 dias de 8,5% e 12,8%, respectivamente (p=0,41). Para os tratamentos em unidade de AVC e em enfermaria geral, as taxas de mortalidade foram, respectivamente: no dia 30, 14,2% e 28,2% (p=0,24); no terceiro mês, 17,4% e 28,7% (p=0,39); e no sexto mês, 25,7% e 30,7% (p=0,41). A curva de sobrevida de 30 dias mostrou tendência não significativa de menor letalidade na unidade de AVC. Para evitar uma morte em 6 meses na unidade de AVC, o número necessário para tratar foi 20; para se mandar para casa mais um paciente independente, o número necessário para tratar foi 15. ^95^
- Um estudo descreveu as características e os cuidados prestados a 148 pacientes admitidos com AVC isquêmico na emergência neurológica e na enfermaria da neurologia de um grande hospital público acadêmico de São Paulo. O estudo mostrou que o AVC isquêmico foi diagnosticado em 79,6% dos pacientes com DCV admitidos na emergência neurológica, sendo trombólise oferecida a 2,7%. A extensão da investigação e o manejo do AVC isquêmico diferiram significativamente entre a emergência neurológica e a enfermaria da neurologia. ^96^
- Um estudo de base hospitalar, avaliando 2.407 pacientes consecutivos (idade média, 67,7 ± 14,4 anos; 51,8% mulheres) admitidos em 19 hospitais de Fortaleza com diagnóstico de AVC ou AIT, mostrou que AVC isquêmico foi o subtipo mais frequente (72,9%), sendo seguido por hemorragia intraparenquimatosa (15,2%), HSA (6,0%), AIT (3%) e AVC indeterminado (2,9%). O tempo médio entre o aparecimento dos sintomas e a internação foi de 12,9 (3,8 - 32,5) horas. Hipertensão foi o fator de risco mais comum. Apenas 1,1% dos pacientes com AVC isquêmico receberam trombólise. O tempo médio entre a internação e a obtenção de neuroimagem foi de 3,4 (1,2 - 26,5) horas. ^74^
- Um estudo comparou os indicadores de qualidade para manejo de AVC de um hospital privado terciário, certificado pela JCI como Centro Primário de AVC, com aqueles do programa GWTG-Stroke da *American Heart Association/American Stroke Association* : (1) uso do ativador do plasminogênio tecidual em pacientes que chegam até 2 horas após o início dos sintomas; (2) uso de medicação antitrombótica nas primeiras 48 horas de internação; (3) profilaxia de trombose venosa profunda nas primeiras 48 horas de internação para pacientes com contraindicação ou impossibilidade de deambulação; (4) uso de antitrombóticos na alta hospitalar; (5) uso de anticoagulação para fibrilação atrial na alta hospitalar; (6) medida de LDL e tratamento para LDL > 100 mg/dL para pacientes que atendam às recomendações do NCEP III; e (7) aconselhamento para cessação de tabagismo. O estudo avaliou 343 pacientes consecutivos com AVC isquêmico agudo (70,8%) ou AIT (29,2%) de agosto de 2008 a dezembro de 2010. Medicação antitrombótica foi administrada nas primeiras 48 horas em 98,5% dos pacientes elegíveis e profilaxia de trombose venosa profunda foi realizada em 100%. Do total, 123 pacientes chegaram em até 2 horas após o início dos sintomas, 23 eram elegíveis para trombólise intravenosa e 16 foram tratados (69,5%). Todos os pacientes elegíveis receberam alta em uso de medicação antitrombótica e 86,9% dos elegíveis que apresentavam fibrilação atrial receberam anticoagulação. Apenas 56,1% dos pacientes elegíveis foram tratados de acordo com as recomendações do NCEP III. Realizou-se aconselhamento para cessação de tabagismo em 63,6% dos pacientes elegíveis. ^97^
- Um estudo analisou os fatores que influenciam as tendências temporais dos indicadores de qualidade para AVC isquêmico em um Centro Primário de AVC certificado pela JCI. Para tal, 551 pacientes com AVC isquêmico, que receberam alta de hospital terciário de janeiro de 2009 a dezembro de 2013, foram avaliados. A mediana da idade foi 77,0 anos (intervalo interquartil, 64,0-84,0), sendo 58,4% homens. Dez indicadores de desempenho predefinidos, selecionados do programa GWTG-Stroke, foram avaliados. Os indicadores de qualidade que melhoraram com o tempo foram o uso de terapia hipolipemiante (P = 0,02) e orientação sobre AVC (P = 0,04). A mediana do desfecho composto cuidado ideal não melhorou consistentemente no período (P = 0,13). Após ajuste multivariável, apenas tratamento trombolítico (OR 2,06; P < 0,01), dislipidemia (OR 2,03; P < 0,01) e alta em um ano de visita da JCI (OR 1,8; P < 0,01) permaneceram como preditores de um índice de cuidado ideal igual ou superior a 85%. Os indicadores de qualidade com pior desempenho (anticoagulação para fibrilação atrial e redução de colesterol) foram semelhantes nos hospitais comunitários terciários e secundários. A medida geral de cuidado ideal não melhorou e foi influenciada por: receber alta em ano de visita da JCI, ter dislipidemia e ter recebido tratamento trombolítico. ^98^
- Outro estudo avaliou indicadores-chave de desempenho do Ministério da Saúde para AVC nas unidades para AVC de dois centros, incluindo a porcentagem de pacientes admitidos nessas unidades, profilaxia de tromboembolismo venoso nas primeiras 48 horas de internação, pneumonia e mortalidade hospitalar por AVC e alta hospitalar em uso de antitrombóticos para pacientes sem mecanismo cardioembólico. A análise revelou que os dois centros internaram mais de 80% dos pacientes em suas unidades para AVC. A incidência de profilaxia de tromboembolismo venoso foi superior a 85% e a de pneumonia hospitalar foi inferior a 13%. A taxa de mortalidade hospitalar por AVC foi inferior a 15% e a de alta hospitalar em uso de antitrombóticos foi superior a 70%. ^99^
- Um estudo avaliou as taxas de mortalidade antes e depois da implementação de uma unidade cardiovascular dedicada a AVC no setor de emergência de um hospital público terciário em Porto Alegre. No período anterior à implementação da unidade vascular (2002 a 2005), foram incluídos 4.164 pacientes e, no período posterior à implementação (2007 a 2010), foram incluídos 6.280 pacientes. A taxa de letalidade geral por condições vasculares agudas diminuiu de 9% para 7,3% com a implementação (p = 0,002). Entretanto, a taxa de letalidade por AVC não diminuiu a despeito das melhorias nos indicadores de qualidade de cuidado para AVC. ^100^
- Um ensaio clínico randomizado por *cluster* avaliou o efeito de uma intervenção multifacetada de melhoria da qualidade da adesão às terapias baseadas em evidência para o cuidado de pacientes com AVC isquêmico agudo e AIT (incluindo manejo de caso, lembretes, *roadmap* e *checklist* para o plano terapêutico, materiais educativos, auditorias periódicas e relatórios de *feedback* para cada *cluster* da intervenção). O estudo avaliou 1.624 pacientes de 36 hospitais, cobrindo todas as regiões brasileiras. O desfecho primário foi um desfecho composto de adesão ao escore para AVC isquêmico agudo e às medidas de desempenho para AIT, e os desfechos secundários incluíram um desfecho composto ‘tudo-ou-nada’ de adesão às medidas de desempenho. A idade média geral (DP) dos pacientes arrolados no estudo foi 69,4 (13,5) anos, e 913 (56,2%) eram homens. As médias (DP) do desfecho composto de adesão ao escore e às 10 medidas de desempenho dos hospitais do grupo ‘intervenção’ e do grupo controle foram 85,3% (20,1%) e 77,8% (18,4%), respectivamente, com diferença média de 4,2% (IC 95%, -3,8% a 12,2%). Como desfecho secundário, 402 de 817 pacientes (49,2%) dos hospitais do grupo ‘intervenção’ receberam todas as terapias para as quais eram elegíveis, enquanto que, nos do grupo controle, 203 de 807 (25,2%) receberam aquelas terapias (OR, 2,59; IC 95%, 1,22 - 5,53; P = 0,01). A intervenção não resultou em significativo aumento de adesão às terapias baseadas em evidência em pacientes com AVC isquêmico agudo ou AIT. Entretanto, ao usar a abordagem ‘tudo-ou-nada’, a intervenção resultou em melhoria da adesão às terapias baseadas em evidência e melhores taxas de trombólise. ^101^

### Custo

#### (Ver Tabelas 1-6 a 1-9 e Figuras 1-15 e 1-16)

- De acordo com a base de dados administrativos do SUS, o total de gastos atribuídos a doenças cerebrovasculares aumentou de 2008 a 2018, considerando os procedimentos clínicos relacionados a internações hospitalares. Em 2008 e 2018, houve 159.545 e 203.066 hospitalizações por doenças cerebrovasculares, respectivamente, de um total de 2.042.195 internações no período, representando um total de custos de R$ 142.061.641 (2018 Int$ 136.975.201) em 2008 e de R$ 286.293.302 (2018 Int$ 141.170.268) em 2018, com acumulado de R$ 2.498.850.166 no referido período. Houve um real aumento no total de custos atribuídos a doenças cerebrovasculares na última década após ajuste para inflação, provavelmente em razão do aumento na complexidade do tratamento oferecido para aquelas condições.
- Um estudo de custo-efetividade avaliando os trombolíticos no Brasil relatou que, para homens e em 1 ano, o custo do tratamento com rtPA foi maior do que o custo do tratamento conservador, com ganho de QALY de 0,06 para ambos os sexos. Esse resultado deve-se principalmente ao custo da medicação. Parte desse custo adicional é compensado pelo menor custo da reabilitação e menor perda de produtividade nos primeiros 2 anos, pois os pacientes tratados com rtPA apresentaram menos sequelas do que aqueles que receberam tratamento conservador. Depois do segundo ano que se segue a um AVC, para os dois sexos, o tratamento com rtPA (alteplase), considerando-se os custos diretos e indiretos, começou a ter menor custo em comparação ao conservador. Daí para a frente, o custo adicional da medicação começa a ser mais do que compensado pela menor perda de produtividade e menores custos com seguridade social e reabilitação do paciente. ^102^

### Prevenção

- O Estudo PURE ( *Prospective Urban Rural Epidemiological)* examinou as taxas e preditores do uso de medicação de prevenção secundária baseada em evidência (IECA/BRA, antiagregantes plaquetários, estatinas e betabloqueadores) em pacientes com DCV, incluindo DAC e AVC, em países da América do Sul, entre os quais o Brasil. O estudo mostrou que um menor número de pacientes com AVC recebeu antiagregantes plaquetários (24,3%), IECA/BRA (37,6%) e estatinas (9,8%) em comparação àqueles com DAC (30,1%, 36,0% e 18,0%, respectivamente). Essa subutilização de terapias em pacientes com AVC variou substancialmente entre os países, tendo a Colômbia o menor uso (sem prescrição de estatinas). Quando pacientes com DAC e AVC foram combinados, a proporção de uso de antiagregantes plaquetários foi mais alta no Chile (38,1%) e mais baixa na Argentina (23,0%). O uso de IECA/BRA e estatinas foi maior no Brasil (46,4% e 26,4%, respectivamente) e menor na Colômbia (26,4% e 1,4%, respectivamente). Entre os participantes com DAC e AVC, o uso foi maior naqueles com maior nível educacional se comparados àqueles sem nenhuma educação, educação primária ou desconhecida [35,6% vs. 23,6%, respectivamente, para antiagregantes plaquetários (p = 0,002); 20,6% vs. 10,9%, respectivamente, para estatinas (p = 0,0007)]. Ex-fumantes com DAC ou AVC tiveram maior probabilidade de receber terapias comprovadas do que fumantes atuais ou aqueles que nunca fumaram [35,2% vs. 26,6% e 27,7%, respectivamente, para antiagregantes plaquetários (p = 0,039); 19,9% vs. 10,6% e 13,0%, respectivamente, para estatinas (p = 0,004)]. Apenas 4,1% dos pacientes receberam todas as 4 terapias (IECA/BRA, antiagregantes plaquetários, estatinas e betabloqueadores), sendo a maior taxa do Brasil (5,5%) e a menor, da Colômbia (0,5%) (p = 0,02). Além disso, observou-se que 30% dos brasileiros com AVC não usam qualquer medicação. ^103^

### Conscientização, Tratamento e Controle

- Vários estudos mostraram alarmante falta de conhecimento sobre os fatores de risco para AVC, seu tratamento e reconhecimento dos sintomas de AVC como uma emergência médica. Em um estudo de base comunitária, Pontes-Neto *et al* . entrevistaram indivíduos em locais públicos de 4 importantes cidades no Brasil entre julho de 2004 e dezembro de 2005, usando um questionário estruturado aberto em português, baseado na apresentação de um caso típico de AVC agudo domiciliar. Os autores identificaram 28 termos diferentes em português para denominar AVC. Quanto aos entrevistados, 22% deles não reconheceram qualquer sinal de alarme de AVC. Apenas 34,6% dos entrevistados responderam corretamente quando perguntados sobre o número de telefone de emergência no Brasil (#192). Apenas 51,4% dos entrevistados relataram que chamariam uma ambulância para um familiar com sintomas de AVC. ^104^
- Falavigna *et al* . usaram um questionário fechado e autoadministrado para avaliar o conhecimento sobre AVC entre 952 residentes da cidade de Caxias do Sul. Baixa renda e baixo nível educacional foram preditores independentes da incapacidade de reconhecer que o AVC afeta o cérebro. Renda mais baixa e idade < 50 anos foram preditores independentes da falta de conhecimento sobre os fatores de risco para AVC. ^105^
- Panicio *et al* . entrevistaram 104 pacientes consecutivos com AVC agudo admitidos em um hospital público terciário de São Paulo, de março a dezembro de 2012, para avaliar o conhecimento sobre AVC e o impacto da falta de conscientização sobre a demora em se chegar ao hospital em caso de AVC. Embora 66,2% dos pacientes conhecessem os sinais de alerta de AVC, apenas 7,8% mostraram saber da limitada janela de tempo para a terapia de reperfusão. A gravidade do AVC medida pela escala NIHSS foi o único preditor independente de chegada precoce ao hospital. ^106^
- Em estudo transversal de base comunitária, Pitton Rissardo *et al* . aplicaram um inquérito de conhecimento sobre AVC em uma amostra de conveniência composta por 633 passantes de uma praça pública na cidade de Santa Maria, Rio Grande do Sul, de dezembro de 2015 a outubro de 2016. Dos respondentes, 33% informaram corretamente o significado do acrônimo ‘AVC’, o termo mais recomendado para designar ‘acidente vascular cerebral’ pela Sociedade Brasileira de Doenças Cerebrovasculares. Cerca de 30% dos respondentes localizaram incorretamente o AVC no coração. Apenas 50% dos respondentes identificaram corretamente um sinal de alarme de AVC. Indivíduos de nível educacional mais alto apresentaram maior probabilidade de chamar uma ambulância para um familiar com sintomas de AVC. ^107^
- Recentemente, tem havido várias iniciativas para promover a conscientização do público sobre AVC no Brasil, em especial através de campanhas anuais por ocasião do Dia Mundial do AVC (29 de outubro), conduzidas pela Organização Mundial do AVC. A despeito desses esforços, apenas 30-40% dos pacientes com AVC são hospitalizados nas primeiras 4 horas após início dos sintomas. ^108^

### Pesquisa Futura

- O portfólio de pesquisa brasileira em neurologia vascular evoluiu muito nos últimos anos. No entanto, ainda há inúmeras oportunidades para melhoria. Os estudos comunitários mais expressivos sobre prevalência e incidência de AVC são provenientes principalmente de 2 cidades. Embora representem uma importante realização para a epidemiologia do AVC, avaliação mais abrangente se faz necessária, compreendendo a representação de todas as regiões geográficas, das diversas culturas, dos níveis de renda e das etnias.
- Além disso, há limitações para os estudos relacionadas à identificação do AVC usando os códigos da CID. Não é incomum que os usuários utilizem um código mais amplo na admissão, que, por não ser ajustado durante a hospitalização, não representa o verdadeiro subtipo de AVC (e.g., um AVC isquêmico pode ser codificado como AVC não específico ou até AIT). Com o advento das tecnologias *big data* (e.g., mineração de dados textuais), informação clínica adicional proveniente dos registros de admissão ou alta pode ser uma fonte confiável de contrarreferência, confirmando ou corrigindo um dado código.
- Como desafio mundial, não restrito ao Brasil, estudos dos serviços de saúde utilizando metodologia robusta, avaliando não apenas a realidade da prestação do cuidado em saúde, mas também a efetividade das políticas em saúde através de ensaios clínicos randomizados, precisam se tornar a base dos programas de melhoria da qualidade nos níveis comunitário e populacional.

**Tabela 2-1:** – Taxa de prevalência de AVC padronizada por idade (por 100 mil) para ambos os sexos, para homens e para mulheres, no Brasil e unidades federativas, 1990 e 2017, e variação percentual das taxas

	Ambos os sexos			Mulheres			Homens		
	1990	2017	Variação percentual	1990	2017	Variação percentual	1990	2017	Variação percentual
Brasil	6290 (6048.3;6548.9)	6025 (5785.8;6274.8)	-4.2 (-3.2;-5.1)	5939.5 (5694.8;6205.5)	5612.9 (5366.3;5856.6)	-5.5 (-4.2;-6.7)	6697.3 (6433.7;6961.9)	6536.8 (6282.7;6806.6)	-2.4 (-1.3;-3.4)
Acre	6011.6 (5762.4;6272.3)	5814.9 (5568.2;6071.4)	-3.3 (-1.4;-5.3)	5595.5 (5340.7;5865.5)	5350.3 (5101.6;5618.9)	-4.4 (-1.5;-7.2)	6361.8 (6076.6;6659.2)	6299 (6033;6583.4)	-1 (1.8;-3.9)
Alagoas	5960.7 (5700.5;6233.7)	5790.1 (5543.7;6044.7)	-2.9 (-0.8;-4.8)	5603.9 (5346;5888.2)	5381 (5123;5659.8)	-4 (-1;-6.9)	6360 (6070.2;6651.8)	6307.2 (6024.2;6580.8)	-0.8 (2;-3.5)
Amapá	6219.5 (5950.1;6490.2)	6185.3 (5922.8;6478.7)	-0.5 (1.5;-2.7)	5945.1 (5646.7;6260.9)	5817.8 (5542.5;6137.7)	-2.1 (1.3;-5.4)	6515.4 (6227.7;6811.6)	6587.8 (6304.5;6889.1)	1.1 (3.8;-1.6)
Amazonas	5728.7 (5485.7;5987.5)	5701.6 (5455.9;5955)	-0.5 (1.8;-2.6)	5367.7 (5112.8;5643.3)	5244.5 (4984.1;5506.4)	-2.3 (0.8;-5.5)	6092 (5823.9;6361.4)	6183.2 (5892.3;6475.4)	1.5 (4.5;-1.3)
Bahia	5760.9 (5521.5;6019.9)	5685.9 (5433.9;5942.7)	-1.3 (0.9;-3.4)	5420.2 (5164;5706.9)	5247 (4989.7;5508.9)	-3.2 (-0.2;-6)	6147.9 (5863.3;6431.4)	6225 (5936.4;6513.3)	1.3 (4.3;-1.6)
Ceará	5860.4 (5600.4;6118.9)	5943.7 (5668.7;6231.8)	1.4 (3.8;-0.9)	5545.9 (5270.6;5820.4)	5541.3 (5256.3;5850.7)	-0.1 (3.2;-3.3)	6211.7 (5923.9;6506.7)	6448.9 (6145.4;6775.2)	3.8 (7.1;0.7)
Distrito Federal	5798.5 (5563.4;6037.2)	5529.8 (5303.6;5752.8)	-4.6 (-2.7;-6.5)	5461.1 (5211.3;5732.7)	5144.9 (4908.1;5382.3)	-5.8 (-3;-8.7)	6209.3 (5957.1;6458.7)	6061.9 (5797;6321.5)	-2.4 (0.3;-4.9)
Espírito Santo	6214.8 (5957;6478.6)	5748.2 (5490.1;5998.6)	-7.5 (-5.6;-9.5)	5868.6 (5600.7;6137.1)	5341.6 (5086.6;5596.6)	-9 (-6.1;-11.8)	6591.9 (6320.7;6883.9)	6240.1 (5950.8;6518.6)	-5.3 (-2.8;-7.8)
Goiás	5642.8 (5395.9;5901.5)	5527.3 (5295.5;5762.8)	-2 (0.1;-4.2)	5318.3 (5037.2;5601.6)	5136.8 (4890.8;5398.2)	-3.4 (0;-6.6)	5947.9 (5701.8;6217.5)	5969.3 (5701.1;6245.8)	0.4 (3.2;-2.6)
Maranhão	5596.3 (5341.3;5857.4)	5592.5 (5348.5;5844.7)	-0.1 (2;-2.1)	5196.3 (4944.4;5463.5)	5174.8 (4941.4;5424)	-0.4 (2.6;-3.3)	6026.1 (5740.7;6311.6)	6065.4 (5785.2;6348.3)	0.7 (3.5;-2.2)
Mato Grosso	5995.6 (5727.7;6258.6)	5869.7 (5612.3;6121.4)	-2.1 (0.2;-4.1)	5622 (5350.5;5896.6)	5441.8 (5189.2;5704.2)	-3.2 (-0.1;-6)	6297.7 (6001.6;6588.6)	6285.3 (5998.3;6582.1)	-0.2 (2.8;-3)
Mato Grosso do Sul	6168.4 (5915.1;6427.7)	5964.9 (5727.2;6210.6)	-3.3 (-1.3;-5.3)	5730.5 (5467.9;5987.4)	5472.6 (5219.8;5732.1)	-4.5 (-1.6;-7.4)	6571.7 (6308.5;6860.6)	6512.2 (6248.7;6790.3)	-0.9 (1.8;-3.7)
Minas Gerais	6269 (5985.9;6552.4)	6031.6 (5768.1;6308.7)	-3.8 (-1.4;-5.9)	5902 (5602;6201.4)	5614.5 (5333.5;5907.9)	-4.9 (-1.8;-7.9)	6699.1 (6401.3;6989.1)	6532.8 (6246.7;6839)	-2.5 (0.2;-5)
Pará	5842.4 (5588.6;6111.7)	5751 (5495.1;6017.5)	-1.6 (0.6;-3.6)	5511.2 (5245.2;5786.3)	5291.5 (5022.7;5567.5)	-4 (-0.7;-7.2)	6191.5 (5918.1;6485.6)	6236.9 (5947.8;6540.6)	0.7 (3.7;-2)
Paraíba	5802.6 (5546.1;6053.4)	5755.9 (5515.6;6004.5)	-0.8 (1.4;-3)	5477.7 (5212.5;5728.9)	5371 (5114.4;5651.9)	-1.9 (1.3;-5)	6170.5 (5892.1;6456.2)	6253.3 (5978.4;6539.4)	1.3 (4.1;-1.5)
Paraná	6350.3 (6083.3;6621.1)	5998 (5747.6;6250.1)	-5.5 (-3.5;-7.6)	5947.5 (5671;6238.5)	5549.2 (5289.1;5808)	-6.7 (-3.8;-9.5)	6765.8 (6472.5;7054)	6538.5 (6262.5;6813.3)	-3.4 (-0.5;-6)
Pernambuco	5864.3 (5618.3;6114.8)	5642.3 (5399.5;5887.5)	-3.8 (-1.7;-5.9)	5544.4 (5296;5814.4)	5239.5 (4986.3;5490.8)	-5.5 (-2.5;-8.3)	6252.9 (5972.2;6545.7)	6184.6 (5912.5;6459.7)	-1.1 (1.7;-3.9)
Piauí	5511.3 (5276.3;5755.1)	5545.1 (5314.7;5786.3)	0.6 (2.8;-1.4)	5145.2 (4900.4;5394.3)	5131.4 (4894.1;5385.3)	-0.3 (2.8;-3.3)	5911.2 (5642.8;6176.5)	6034.4 (5776.8;6317.2)	2.1 (4.8;-0.6)
Rio de Janeiro	6714.2 (6446.8;7008.6)	6230.8 (5980.5;6490.7)	-7.2 (-5.3;-9.2)	6350.5 (6067.4;6659.1)	5820 (5546.8;6108.8)	-8.4 (-5.6;-11.1)	7208.1 (6917.8;7498.2)	6800.2 (6527.3;7073.1)	-5.7 (-3.1;-8.1)
Rio Grande do Norte	5701.2 (5452.1;5951.2)	5672.5 (5438;5939.2)	-0.5 (1.6;-2.4)	5393 (5131.9;5659.9)	5280.7 (5029.7;5550.9)	-2.1 (0.8;-5)	6041.8 (5780.8;6327.3)	6170.4 (5892.3;6466.5)	2.1 (5.1;-0.6)
Rio Grande do Sul	6600.3 (6315.3;6880.9)	6182.1 (5906.1;6460.9)	-6.3 (-4.4;-8.3)	6322.7 (6028.2;6645.2)	5828.4 (5536.3;6116.6)	-7.8 (-4.9;-10.6)	6961.6 (6661.6;7269.6)	6639.1 (6336.7;6946.7)	-4.6 (-2;-7.3)
Rondônia	5985.6 (5731;6240.1)	5705.1 (5457.5;5949.3)	-4.7 (-2.4;-6.8)	5599.4 (5348.2;5868.1)	5285.8 (5041.4;5543.5)	-5.6 (-2.6;-8.6)	6276.9 (5998.5;6564.3)	6111.8 (5837.6;6398.4)	-2.6 (0.4;-5.3)
Roraima	6064.1 (5818.4;6308.8)	5814.1 (5583.7;6060.7)	-4.1 (-2.1;-6)	5617 (5371;5884)	5317.9 (5070;5562.2)	-5.3 (-2.4;-8.1)	6407.7 (6148.3;6694.1)	6269.6 (6021.5;6541.3)	-2.2 (0.4;-4.9)
Santa Catarina	6679.1 (6397.8;6964.3)	6217 (5941.2;6488.4)	-6.9 (-5;-8.8)	6375.3 (6069.7;6674.6)	5844.5 (5564.4;6133.5)	-8.3 (-5.4;-11.1)	7026.3 (6731;7339.3)	6667 (6373.5;6959.6)	-5.1 (-2.3;-7.8)
São Paulo	6801.7 (6517.3;7096.7)	6423.9 (6151.9;6705.8)	-5.6 (-3.4;-7.5)	6406.6 (6114.7;6715.3)	6000.7 (5715;6302.4)	-6.3 (-3.1;-9.3)	7284.4 (6975.5;7612.3)	6975.7 (6661.7;7268.7)	-4.2 (-1.4;-6.8)
Sergipe	5922.8 (5667.3;6171.5)	5851.4 (5597.1;6110.4)	-1.2 (1.2;-3.4)	5582.1 (5328.1;5864.7)	5442.8 (5194.8;5717)	-2.5 (0.7;-5.3)	6324.1 (6036.1;6611.2)	6374.1 (6091.1;6662.7)	0.8 (3.9;-1.8)
Tocantins	5849.3 (5606.1;6103.5)	5849.2 (5606.6;6094.9)	0 (2.1;-2.1)	5387.1 (5153.3;5644.6)	5307.1 (5065.3;5571.5)	-1.5 (1.5;-4.3)	6261.9 (5993.8;6548.1)	6368.8 (6094;6643.8)	1.7 (4.6;-1)

* Fonte: Estudo Global Burden of Disease 2017, Institute for Health Metrics and Evaluation. ^109^*

**Tabela 2-2:** – Número de mortes e taxa de mortalidade padronizada por idade (por 100 mil) por AVC e AVC isquêmico no Brasil e suas unidades federativas, 1990 e 2017, e variação percentual das taxas

Causa de morte e localização	1990		2017		Variação percentual (II 95%)
	Número (II 95%)	Taxa (II 95%)	Número (II 95%)	Taxa (II 95%)	
***B.2.3 - AVC***					
Acre	146 (140;152)	101 (96,3;104,9)	318 (297;337)	59,3 (55,4;63,1)	-41,3 (-45,4;-36,8)
Alagoas	1767 (1697;1843)	138,2 (133;143,6)	2341 (2227;2457)	79,4 (75,4;83,5)	-42,6 (-45,8;-39,3)
Amapá	74 (70;77)	90,1 (85;93,9)	242 (226;256)	57,7 (54;61,3)	-35,9 (-40;-31,5)
Amazonas	679 (636;714)	99 (92,7;103,9)	1407 (1332;1476)	58,7 (55,7;61,7)	-40,7 (-44,4;-36,6)
Bahia	6405 (6059;6787)	96 (90,8;101,7)	9239 (8841;9681)	58,3 (55,7;61,4)	-39,3 (-43,1;-35)
Brasil	97101 (95272;98780)	122,9 (120,6;125)	122783 (119899;125348)	56,6 (55,2;57,8)	-54 (-55,1;-53)
Ceará	3266 (3038;3496)	80,7 (74,7;86,7)	5444 (5193;5691)	54,8 (52,2;57,2)	-32,2 (-37,5;-26,3)
Distrito Federal	536 (516;556)	117,2 (113;121,2)	961 (902;1028)	53,5 (49,9;57,1)	-54,4 (-57,5;-51)
Espírito Santo	1972 (1914;2030)	172,1 (166,9;177,1)	2188 (2079;2303)	54,6 (51,9;57,4)	-68,3 (-69,9;-66,5)
Goiás	1920 (1845;2004)	121,4 (116,7;126,6)	2925 (2779;3091)	48,2 (45,8;50,9)	-60,3 (-62,4;-58)
Maranhão	2563 (2374;2779)	101 (93,1;110,6)	4261 (4020;4592)	68,9 (65;74,3)	-31,7 (-36,6;-26,6)
Mato Grosso	637 (596;683)	93,3 (87,3;99,6)	1407 (1328;1491)	51,9 (48,9;55)	-44,4 (-48,8;-38,9)
Mato Grosso do Sul	937 (904;973)	121,6 (117,6;126,2)	1554 (1477;1642)	60,4 (57,4;63,8)	-50,3 (-53,4;-47,3)
Minas Gerais	10859 (10542;11270)	127 (123,3;131,2)	12638 (12067;13238)	50,5 (48,2;53)	-60,2 (-62,2;-58,1)
Pará	2210 (2066;2326)	117,3 (109,8;123,5)	3993 (3776;4201)	66 (62,3;69,5)	-43,8 (-47,6;-39,4)
Paraíba	2070 (1958;2194)	92,5 (87,4;98,1)	2856 (2642;3073)	60,8 (56,2;65,4)	-34,3 (-40,6;-27,8)
Paraná	6603 (6394;6801)	174,4 (169;179,4)	7455 (7111;7818)	63,8 (60,9;66,8)	-63,4 (-65,2;-61,5)
Pernambuco	5744 (5538;5948)	145,5 (140,2;150,6)	6553 (6236;6877)	68,7 (65,3;72,2)	-52,8 (-55,1;-50)
Piaui	1422 (1322;1536)	106,4 (98,8;115,1)	2294 (2169;2512)	63,7 (60,2;69,6)	-40,1 (-44,6;-35,2)
Rio de Janeiro	13533 (13076;13920)	160,9 (155,4;165,5)	12650 (12074;13184)	60 (57,3;62,5)	-62,7 (-64,6;-60,8)
Rio Grande do Norte	1180 (1106;1257)	74,2 (69,7;79,2)	1704 (1604;1806)	45 (42,3;47,7)	-39,4 (-44,7;-33,7)
Rio Grande do Sul	7282 (6967;7528)	135,4 (129,5;139,9)	8818 (8373;9257)	60,3 (57,2;63,3)	-55,5 (-57,9;-53)
Rondônia	384 (357;413)	140,1 (131,2;149,5)	764 (691;849)	60,9 (55,2;67,3)	-56,5 (-61,2;-51,4)
Roraima	50 (45;54)	126,6 (117,3;136,8)	152 (135;171)	61,3 (54,7;68,5)	-51,6 (-58;-44,9)
São Paulo	20625 (19917;21335)	121,8 (117,5;126,1)	24963 (23878;26094)	51 (48,7;53,3)	-58,1 (-60,4;-55,8)
Santa Catarina	2947 (2832;3048)	148,9 (142,8;153,8)	3539 (3358;3729)	51,9 (49,3;54,6)	-65,1 (-66,9;-63)
Sergipe	919 (877;957)	110,6 (105,7;115,2)	1328 (1269;1392)	65,5 (62,5;68,7)	-40,8 (-44,4;-37,2)
Tocantins	373 (330;409)	121,4 (110,6;132,1)	790 (741;853)	60,1 (56,3;64,9)	-50,5 (-55,4;-44,6)
***B.2.3.1 - AVC Isquêmico***					
Acre	51 (48;54)	43,7 (41,2;45,9)	110 (102;120)	22,5 (20,6;24,4)	-48,5 (-53,1;-43,4)
Alagoas	708 (669;750)	61,4 (58,2;64,9)	906 (851;964)	31,9 (29,9;34,1)	-48 (-52,3;-43,7)
Amapá	29 (27;31)	43,2 (40,2;45,5)	83 (76;89)	22,9 (21;24,6)	-46,9 (-51,3;-42,1)
Amazonas	247 (228;263)	43,4 (40;46,1)	492 (461;525)	22,6 (21,1;24,2)	-47,8 (-52;-42,6)
Bahia	2466 (2310;2638)	40,2 (37,7;42,9)	3488 (3281;3697)	21,9 (20,6;23,3)	-45,4 (-50;-40,4)
Brasil	37045 (36222;37909)	54,8 (53,6;55,9)	47453 (45939;48713)	22,6 (21,9;23,2)	-58,7 (-60;-57,4)
Ceará	1279 (1165;1399)	33,5 (30,5;36,6)	2266 (2119;2411)	22,5 (21,1;24)	-32,7 (-39,9;-24,2)
Distrito Federal	153 (144;162)	50,9 (48,2;53,5)	325 (299;353)	22,4 (20,6;24,3)	-56,1 (-60,1;-51,7)
Espírito Santo	746 (712;782)	80,9 (77,5;84,4)	810 (759;869)	21,1 (19,7;22,6)	-74 (-75,8;-71,9)
Goiás	623 (589;658)	52,2 (49,4;55,1)	1005 (940;1081)	18,1 (16,9;19,5)	-65,3 (-67,9;-62,5)
Maranhão	812 (712;937)	37,2 (32,6;43)	1582 (1457;1733)	26,3 (24,3;28,9)	-29,2 (-36,4;-21,1)
Mato Grosso	226 (209;245)	42 (39;45,2)	492 (459;529)	20,5 (19,1;22)	-51,2 (-55,9;-46)
Mato Grosso do Sul	317 (300;334)	51,3 (48,8;54)	543 (508;584)	22,7 (21,2;24,4)	-55,8 (-59,7;-51,7)
Minas Gerais	3976 (3792;4182)	56,4 (53,8;59)	4748 (4435;5040)	19,2 (17,9;20,4)	-65,9 (-68,5;-63,4)
Pará	959 (890;1015)	58 (53,8;61,2)	1551 (1442;1657)	27,5 (25,5;29,3)	-52,6 (-56,7;-48,4)
Paraíba	853 (787;928)	40 (37;43,3)	1163 (1065;1266)	24,2 (22,1;26,5)	-39,4 (-46,7;-31,4)
Paraná	2537 (2416;2649)	82,5 (78,8;86,1)	3107 (2930;3296)	28,1 (26,5;29,8)	-65,9 (-68,2;-63,6)
Pernambuco	2296 (2183;2413)	65,8 (62,7;69)	2429 (2266;2589)	26,3 (24,4;28,1)	-60,1 (-63,1;-56,7)
Piaui	523 (472;587)	43,8 (39,6;49,1)	922 (859;1023)	25,6 (23,8;28,4)	-41,7 (-47,5;-35,4)
Rio de Janeiro	4769 (4552;4985)	68,3 (65,2;71,3)	4520 (4224;4807)	22 (20,6;23,4)	-67,8 (-70,1;-65,2)
Rio Grande do Norte	565 (523;611)	36,9 (34,2;39,8)	699 (648;751)	18,2 (16,8;19,6)	-50,7 (-56,3;-44,9)
Rio Grande do Sul	3219 (3048;3377)	70,1 (66,5;73,2)	3691 (3446;3933)	25,6 (23,9;27,4)	-63,4 (-66,1;-60,6)
Rondônia	110 (101;120)	61,3 (57;65,7)	282 (253;314)	25,1 (22,7;28)	-59 (-63,6;-53,7)
Roraima	15 (13;16)	59,1 (54,5;64,2)	52 (46;58)	26,2 (23,2;29,3)	-55,7 (-62,1;-48,8)
São Paulo	7894 (7483;8299)	56,8 (53,9;59,6)	9945 (9357;10577)	21,3 (20;22,7)	-62,5 (-65,3;-59,5)
Santa Catarina	1164 (1104;1224)	71,1 (67,5;74,5)	1419 (1328;1513)	22,4 (20,9;23,8)	-68,6 (-70,8;-66,1)
Sergipe	389 (367;411)	49,1 (46,2;51,8)	526 (494;559)	26,9 (25,2;28,6)	-45,3 (-49,7;-40,5)
Tocantins	119 (107;132)	53,8 (48,5;59,7)	297 (275;320)	23,6 (21,9;25,5)	-56 (-60,9;-49,9)

* Fonte: Estudo Global Burden of Disease 2017, Institute for Health Metrics and Evaluation. ^109^*

**Tabela 2-3:** – Número de mortes e taxa de mortalidade padronizada por idade (por 100 mil) por hemorragias intracerebral e subaracnóidea no Brasil e suas unidades federativas, 1990 e 2017, e variação percentual das taxas

Causa de morte e localização	1990		2017		Variação percentual (II 95%)
	Número (II 95%)	Taxa (II 95%)	Número (II 95%)	Taxa (II 95%)	
***B.2.3.2 - Hemorragia Intracerebral***					
Acre	76 (72;80)	48,6 (46;51)	169 (157;181)	30,8 (28,4;33)	-36,7 (-42,1;-31,1)
Alagoas	892 (840;948)	66,8 (63;70,9)	1220 (1147;1292)	40,7 (38,3;43,2)	-39 (-43,7;-34,4)
Amapá	37 (34;39)	40,5 (37,7;42,7)	130 (120;140)	29,4 (27;31,7)	-27,3 (-33,1;-20,8)
Amazonas	362 (336;383)	48,7 (45,2;51,4)	761 (711;806)	30,8 (28,8;32,7)	-36,8 (-41,7;-31,4)
Bahia	3297 (3089;3521)	47,9 (44,9;51,1)	4782 (4521;5060)	30,4 (28,6;32,2)	-36,6 (-41,6;-31,4)
Brasil	50247 (49123;51383)	58,6 (57,3;59,9)	61518 (59874;63290)	27,9 (27,1;28,7)	-52,4 (-53,8;-51,1)
Ceará	1646 (1506;1782)	40,2 (36,6;43,6)	2649 (2491;2795)	26,9 (25,3;28,4)	-33,1 (-39,2;-26,5)
Distrito Federal	302 (288;322)	56,1 (53,6;59,1)	498 (463;537)	25,4 (23,6;27,3)	-54,8 (-58,3;-50,7)
Espírito Santo	1035 (996;1079)	79,5 (76,3;82,6)	1124 (1059;1194)	27,6 (26;29,2)	-65,3 (-67,5;-63)
Goiás	1078 (1027;1143)	59,9 (57,1;63,4)	1543 (1447;1638)	24,6 (23;26,1)	-59 (-61,9;-56,1)
Maranhão	1396 (1276;1520)	53,2 (48,5;58,2)	2198 (2042;2416)	35,3 (32,8;38,8)	-33,7 (-39,7;-26,9)
Mato Grosso	325 (300;351)	43,3 (39,9;46,5)	733 (686;785)	25,7 (24,1;27,6)	-40,6 (-46,1;-33,8)
Mato Grosso do Sul	516 (492;540)	60,6 (57,8;63,3)	831 (781;883)	31,3 (29,5;33,3)	-48,2 (-52;-44,6)
Minas Gerais	5752 (5521;6069)	60,5 (58,1;63,3)	6361 (6029;6729)	25,3 (23,9;26,8)	-58,3 (-61,1;-55,5)
Pará	1060 (979;1128)	52,1 (48,1;55,6)	2025 (1888;2161)	32,6 (30,4;34,7)	-37,5 (-43;-31,5)
Paraíba	1008 (938;1082)	44,1 (41;47,2)	1419 (1306;1550)	30,6 (28,1;33,4)	-30,6 (-38,1;-22,5)
Paraná	3442 (3301;3597)	80,3 (76,8;83,8)	3538 (3335;3750)	29,3 (27,7;31,1)	-63,4 (-65,9;-60,8)
Pernambuco	3010 (2866;3154)	70,7 (67,5;74,1)	3459 (3259;3659)	35,8 (33,7;37,9)	-49,4 (-52,5;-45,9)
Piauí	746 (682;809)	53,5 (48,8;57,9)	1156 (1085;1276)	32,2 (30,1;35,5)	-39,9 (-45,1;-33,8)
Rio de Janeiro	7389 (7096;7680)	79,6 (76,6;82,8)	6678 (6329;7056)	31,2 (29,5;32,9)	-60,8 (-63,4;-58,1)
Rio Grande do Norte	515 (477;553)	31,9 (29,5;34,2)	842 (783;908)	22,5 (20,9;24,3)	-29,4 (-36,1;-21,3)
Rio Grande do Sul	3471 (3287;3636)	56,9 (53,9;59,7)	4291 (4020;4547)	28,9 (27,1;30,7)	-49,2 (-52,4;-45,5)
Rondônia	225 (208;243)	69,2 (64;74,6)	394 (352;442)	30 (26,9;33,6)	-56,7 (-61,7;-50,8)
Roraima	28 (25;31)	58,4 (53,7;63,5)	80 (70;91)	29,5 (26;33,4)	-49,5 (-56,8;-41,3)
São Paulo	10472 (10014;10931)	55,4 (52,9;57,9)	11829 (11219;12484)	23,6 (22,3;24,9)	-57,5 (-60,1;-54,3)
Santa Catarina	1508 (1438;1573)	68 (64,8;71)	1727 (1613;1825)	24,4 (22,9;25,9)	-64,1 (-66,6;-61,5)
Sergipe	453 (428;476)	53,7 (50,8;56,5)	677 (638;716)	32,9 (31;34,9)	-38,7 (-43,1;-33,8)
Tocantins	206 (181;230)	58 (52,1;63,5)	404 (373;439)	30,2 (27,9;32,9)	-47,9 (-53,5;-40,8)
***B.2.3.3 - Hemorragia Subaracnóidea***					
Acre	19 (18;20)	8,7 (8,1;9,4)	38 (35;41)	6 (5,5;6,6)	-30,8 (-38,1;-23,1)
Alagoas	168 (147;186)	10 (8,8;11)	215 (197;239)	6,7 (6,1;7,5)	-33 (-42,5;-19,3)
Amapá	8 (7;9)	6,4 (5,9;7,5)	29 (26;33)	5,4 (4,9;6,1)	-15,8 (-24,5;-6,4)
Amazonas	70 (64;76)	6,9 (6,4;7,8)	154 (142;169)	5,3 (4,9;5,9)	-23 (-30,9;-14,5)
Bahia	641 (591;706)	8 (7,3;8,9)	968 (899;1065)	6 (5,6;6,7)	-24,2 (-32;-16)
Brasil	9809 (8917;10192)	9,6 (8,8;9,9)	13811 (13189;14611)	6,1 (5,8;6,4)	-36,5 (-39,8;-32,2)
Ceará	340 (294;402)	7,1 (6,2;8,3)	530 (489;573)	5,4 (4,9;5,8)	-24,1 (-37,1;-13,3)
Distrito Federal	81 (70;87)	10,2 (8,8;10,9)	139 (121;153)	5,8 (4,9;6,4)	-43,8 (-49,1;-36,8)
Espírito Santo	191 (155;204)	11,8 (9,6;12,6)	254 (232;274)	5,9 (5,5;6,4)	-49,7 (-54,5;-38,7)
Goiás	219 (202;234)	9,3 (8,6;9,9)	378 (348;416)	5,5 (5,1;6,1)	-40,2 (-45,4;-33,2)
Maranhão	354 (286;418)	10,5 (8,2;12,7)	482 (423;527)	7,3 (6,4;8)	-30,6 (-41,4;-18,5)
Mato Grosso	85 (78;93)	8 (7,3;8,8)	181 (167;199)	5,6 (5,2;6,2)	-29,8 (-37,3;-21,9)
Mato Grosso do Sul	103 (93;111)	9,7 (8,9;10,4)	180 (165;197)	6,3 (5,8;6,9)	-34,8 (-41,1;-27,3)
Minas Gerais	1130 (977;1203)	10,1 (8,9;10,7)	1529 (1402;1639)	6,1 (5,5;6,5)	-40 (-45,2;-33,5)
Pará	191 (175;211)	7,2 (6,7;8,1)	416 (381;459)	5,9 (5,4;6,6)	-17,9 (-25,6;-8,7)
Paraíba	209 (179;243)	8,5 (7,2;9,9)	274 (247;305)	6 (5,4;6,7)	-29,7 (-42,5;-15,7)
Paraná	624 (516;667)	11,7 (9,7;12,5)	810 (739;871)	6,4 (5,8;6,9)	-45,2 (-50,2;-37,7)
Pernambuco	438 (410;487)	8,9 (8,3;10)	665 (615;733)	6,7 (6,2;7,3)	-25,6 (-32,9;-18,1)
Piaui	153 (128;179)	9,1 (7,6;10,7)	216 (200;235)	6 (5,5;6,5)	-34 (-43,5;-21,9)
Rio de Janeiro	1375 (1099;1473)	12,9 (10,4;13,8)	1452 (1314;1571)	6,8 (6,2;7,3)	-47,4 (-52,3;-38,2)
Rio Grande do Norte	100 (88;125)	5,5 (4,8;7,1)	163 (149;212)	4,3 (3,9;5,6)	-21,3 (-32,2;-9,9)
Rio Grande do Sul	593 (554;667)	8,4 (7,8;9,5)	835 (768;922)	5,7 (5,2;6,3)	-32,1 (-38,5;-25,1)
Rondônia	49 (45;53)	9,6 (8,8;10,6)	88 (77;101)	5,8 (5,1;6,6)	-40 (-48,2;-30,3)
Roraima	7 (7;8)	9,1 (8,1;10,9)	19 (17;24)	5,6 (4,9;6,6)	-38,6 (-48,5;-27,7)
São Paulo	2259 (2009;2403)	9,6 (8,8;10,3)	3190 (2945;3415)	6,1 (5,7;6,6)	-36,3 (-42,3;-30)
Santa Catarina	275 (251;292)	9,8 (8,9;10,5)	392 (362;439)	5,2 (4,8;5,8)	-47,4 (-52,3;-40,2)
Sergipe	77 (71;85)	7,8 (7,2;8,7)	125 (115;138)	5,7 (5,2;6,3)	-27,4 (-36,2;-17,9)
Tocantins	48 (38;57)	9,6 (7,9;11,3)	90 (82;99)	6,2 (5,7;6,8)	-35,2 (-46,1;-21,6)

* Fonte: Estudo Global Burden of Disease 2017, Institute for Health Metrics and Evaluation. ^109^*

**Tabela 2-4:** – Taxa de mortalidade padronizada por idade (por 100 mil) por todos os tipos de AVC, AVC isquêmico, hemorragias intracerebral e subaracnóidea, no Brasil, 1990 e 2017, e variação percentual das taxas

Causa de morte e grupo etário	1990	2017	Variação percentual (II 95%)
***B.2.3 - AVC***			
15-49 anos	17,8 (17,3;18,3)	8,3 (8,1;8,6)	-53,4 (-55,2;-51,6)
50-69 anos	215,8 (210,8;220,9)	95 (92,4;97,6)	-56 (-57,5;-54,5)
5-14 anos	0,9 (0,8;1)	0,4 (0,3;0,4)	-57,4 (-61,8;-51,7)
70+ anos	1145,5 (1119,4;1166)	639,5 (622,2;655,6)	-44,2 (-45,6;-42,7)
Padronizada por idade	122,9 (120,6;125)	56,6 (55,2;57,8)	-54 (-55,1;-53)
Todas as idades	65 (63,8;66,1)	58 (56,6;59,2)	-10,8 (-13;-8,8)
Abaixo de 5	4,3 (3,5;5,4)	0,7 (0,6;0,8)	-84,1 (-88,7;-78)
***B.2.3.1 - AVC Isquêmico***			
15-49 anos	2,2 (2,1;2,4)	0,9 (0,8;0,9)	-59,7 (-64,2;-56,7)
50-69 anos	58 (56;60,2)	21,1 (20,1;21,9)	-63,7 (-65,8;-61,6)
5-14 anos	0,1 (0;0,1)	0 (0;0)	-71,6 (-76,9;-65,9)
70+ anos	618,1 (603,3;632,5)	319,7 (309,7;328,7)	-48,3 (-49,9;-46,5)
Padronizada por idade	54,8 (53,6;55,9)	22,6 (21,9;23,2)	-58,7 (-60;-57,4)
Todas as idades	24,8 (24,2;25,4)	22,4 (21,7;23)	-9,6 (-12,6;-6,8)
Abaixo de 5	0,2 (0,2;0,3)	0 (0;0)	-89,7 (-94,3;-82,2)
***B.2.3.2 - Hemorragia Intracerebral***			
15-49 anos	11,2 (10,9;11,9)	4,8 (4,5;5)	-57,6 (-60,9;-55,4)
50-69 anos	133,4 (129,6;137,1)	58,3 (56,4;60,3)	-56,3 (-58;-54,5)
5-14 anos	0,3 (0,2;0,3)	0,1 (0,1;0,1)	-59,6 (-66,9;-51,7)
70+ anos	482,2 (468,2;494,2)	281 (272,2;289,9)	-41,7 (-43,8;-39,6)
Padronizada por idade	58,6 (57,3;59,9)	27,9 (27,1;28,7)	-52,4 (-53,8;-51,1)
Todas as idades	33,6 (32,9;34,4)	29 (28,3;29,9)	-13,6 (-16,2;-11,1)
Abaixo de 5	1 (0,6;1,6)	0,1 (0,1;0,1)	-89 (-94,1;-79,7)
***B.2.3.3 - Hemorragia Subaracnóidea***			
15-49 anos	4,4 (3,8;4,5)	2,6 (2,5;2,9)	-39,4 (-43,6;-29,4)
50-69 anos	24,4 (22;25,6)	15,6 (14,8;16,6)	-36 (-40,2;-29,6)
5-14 anos	0,6 (0,5;0,6)	0,3 (0,2;0,3)	-54,9 (-59,2;-47,8)
70+ anos	45,3 (43,2;49,1)	38,8 (35,8;41)	-14,2 (-22,7;-8,1)
Padronizada por idade	9,6 (8,8;9,9)	6,1 (5,8;6,4)	-36,5 (-39,8;-32,2)
Todas as idades	6,6 (6;6,8)	6,5 (6,2;6,9)	-0,7 (-6;7,4)
Abaixo de 5	3 (2,4;3,6)	0,5 (0,5;0,6)	-82 (-86,4;-74,1)

* Fonte: Estudo Global Burden of Disease 2017, Institute for Health Metrics and Evaluation. ^109^*

**Tabela 2-5:** – Número de DALYs, taxas de DALYs padronizadas por idade (por 100 mil) por AVC e subtipos de AVC, no Brasil e unidades federativas, 1990 e 2017, e variação percentual das taxas

Causa de morte e localização	1990		2017		Variação percentual (II 95%)
	Número (II 95%)	Taxa (II 95%)	Número (II 95%)	Taxa (II 95%)	
***B.2.3- AVC***					
Acre	3953.6 (3783.3;4122.2)	1979.4 (1889.4;2059)	7266.7 (6834.2;7705)	1186.3 (1115.8;1257.3)	-40.1 (-43.8;-36.1)
Alagoas	43233.8 (40973.6;45710.8)	2817.5 (2696.9;2939.7)	50039.4 (47688.1;52560.1)	1603.7 (1528;1685.4)	-43.1 (-46.4;-39.8)
Amapá	1920.5 (1817.3;2003.4)	1670.5 (1572;1744.3)	6037.8 (5631.2;6387.6)	1177.4 (1098.1;1246.7)	-29.5 (-33.8;-25.2)
Amazonas	17676.9 (16548.2;18641.9)	1941.3 (1817.2;2042.1)	31700.8 (29773.6;33426.1)	1140 (1076.7;1203.3)	-41.3 (-44.6;-37.3)
Bahia	156852.9 (148273.2;166581.9)	2060 (1952.1;2187.7)	195170.9 (186401.1;204704.4)	1235.6 (1179.1;1295.2)	-40 (-43.7;-36.1)
Brasil	2448379.5 (2393632.7;2503724.8)	2511.9 (2457.3;2567.6)	2594661.6 (2510573.1;2684848.2)	1145.3 (1107.8;1185.3)	-54.4 (-55.5;-53.2)
Ceará	77492.6 (71681.9;83089.4)	1703.7 (1591.4;1819.1)	103566.8 (98110.9;108379.4)	1057.1 (1000.6;1107)	-38 (-42.2;-33.4)
Distrito Federal	16605 (15926.8;17311.1)	2251.3 (2165.6;2333.6)	22403.1 (21031.9;23843.1)	920.1 (864.6;978.9)	-59.1 (-61.8;-56.6)
Espírito Santo	48506.9 (47021.8;49978.7)	3107.9 (3018.3;3201)	46467.9 (44131.5;48868.8)	1096.7 (1041.8;1153.2)	-64.7 (-66.5;-62.8)
Goiás	53132.8 (51019.6;55603.8)	2378.7 (2290.8;2484.2)	67110 (63427;71058)	992.6 (937.7;1051.6)	-58.3 (-60.4;-56.1)
Maranhão	75263.4 (68906.4;82104.3)	2388.4 (2215.7;2565.2)	91064 (85853.5;96952.9)	1402.6 (1322.8;1493.3)	-41.3 (-46;-36.2)
Mato Grosso	18394.3 (17193.1;19706.7)	1921.2 (1795;2051.7)	33309.4 (31413.2;35286.5)	1053.1 (994.2;1114.2)	-45.2 (-49.3;-40.3)
Mato Grosso do Sul	24999.9 (23740.6;26097.7)	2462.2 (2369.6;2564.2)	34030.3 (32246.1;35981.7)	1208.7 (1146.7;1277.8)	-50.9 (-53.7;-48)
Minas Gerais	285548.6 (275818.9;297328.5)	2638.6 (2555.4;2738.7)	271355.2 (257504.2;284521)	1075.3 (1020.5;1128.8)	-59.2 (-61.3;-57)
Pará	53548.4 (50133.8;56655)	2253.4 (2113.7;2377)	88611.2 (83452.1;93804)	1314.4 (1238.6;1389.9)	-41.7 (-45.7;-37.3)
Paraíba	45144.4 (42515.4;47988.9)	1858.6 (1751.2;1976.6)	54910.4 (50876.5;59063.6)	1206.4 (1118.5;1299.4)	-35.1 (-40.9;-29.1)
Paraná	161625.7 (156492.6;166651.6)	3179.7 (3077;3274.8)	151291.7 (143947.2;158892.6)	1197.3 (1140.6;1255.3)	-62.3 (-64.2;-60.3)
Pernambuco	130872.4 (125974.8;135596)	2751.6 (2651.9;2849.7)	136137.9 (129804.7;142784.5)	1375 (1311.6;1441)	-50 (-52.3;-47.6)
Piauí	34432.1 (31750.1;37132.4)	2154.9 (1999.1;2316.3)	44933.5 (42457;48250.5)	1250.2 (1181.4;1343.6)	-42 (-46.3;-37.4)
Rio de Janeiro	347453.9 (334592.7;357975.7)	3346.4 (3230.9;3445.4)	273760.5 (261736;286898.1)	1271.2 (1214.1;1331.4)	-62 (-63.9;-60.1)
Rio Grande do Norte	25680.8 (24130.7;27242.4)	1487.2 (1395.3;1582.5)	34501.8 (32491.3;36566.5)	927.5 (872.6;983.7)	-37.6 (-42.5;-32.5)
Rio Grande do Sul	172339.3 (165146.5;178677.8)	2524.5 (2421.8;2615.5)	170330.5 (160060.7;179998.5)	1150.9 (1082.3;1214.1)	-54.4 (-56.8;-52)
Rondônia	12073.7 (11218.4;12924.2)	2741.2 (2563.3;2929.9)	17322.9 (15670.4;19262)	1178.2 (1069.9;1308.7)	-57 (-61.6;-51.8)
Roraima	1569.1 (1426.3;1728.5)	2172.9 (2005;2364.6)	3644.2 (3249.6;4101.8)	1057.5 (946.3;1182.6)	-51.3 (-57.5;-44.5)
Santa Catarina	69910.9 (67055.5;72514.6)	2666.7 (2559.3;2763.6)	71975.6 (67852.1;76213.3)	950.5 (898.1;1006.1)	-64.4 (-66.2;-62.4)
São Paulo	539524.3 (519316.5;557948.6)	2449 (2360.9;2532.4)	542703.5 (515509.8;569546.5)	1043.5 (993.5;1094.6)	-57.4 (-59.5;-55.2)
Sergipe	20033.1 (19174.8;20849.7)	2166.3 (2075.3;2253.8)	27552 (26196.3;28951.2)	1282.7 (1220.2;1347.5)	-40.8 (-44;-37.2)
Tocantins	10590 (8966.2;11793.5)	2245 (1990.4;2461.4)	17463.5 (16283.5;18745.7)	1235.7 (1153.4;1326.5)	-45 (-50.5;-37.6)
***B.2.3.1- AVC Isquêmico***					
Acre	1063.5 (996.8;1132.3)	667.8 (626.7;707.7)	2076 (1893.9;2264.6)	383.1 (351;417.7)	-42.6 (-47.4;-37.9)
Alagoas	13197.8 (12462.3;14030.4)	985.2 (929.6;1045.1)	15974 (14833.8;17114.8)	542.2 (503.3;580.4)	-45 (-48.8;-40.7)
Amapá	587.7 (545;627.3)	640.8 (595.3;680.4)	1748.6 (1589.7;1898.5)	402.8 (368.8;435.9)	-37.1 (-41.9;-32.4)
Amazonas	5084.6 (4695.8;5429.5)	688.6 (637.7;735)	9130.4 (8393.2;9894.1)	375.1 (345.4;405.2)	-45.5 (-49.4;-41)
Bahia	46074.5 (43055.7;49389.4)	671.6 (627.8;719.8)	60202.2 (56130.2;64836.4)	388.2 (361.5;418.2)	-42.2 (-46.8;-37.5)
Brasil	731917.9 (704484.3;760431.9)	871.4 (841.1;902.4)	846622.4 (794644.5;900331.8)	387.3 (363.8;411.5)	-55.6 (-57.3;-53.8)
Ceará	22421 (20485.5;24419.3)	546.3 (497.9;596.2)	35205.6 (32642.9;37754.2)	361.7 (335.2;388.2)	-33.8 (-40.2;-27.1)
Distrito Federal	3906.4 (3644.7;4163.9)	766.5 (721.7;813)	6593.1 (5992;7230)	325.9 (298.5;356.9)	-57.5 (-61.1;-53.7)
Espírito Santo	14282 (13533.3;15095.1)	1098.2 (1045.3;1153.6)	14731.1 (13585.4;15935.6)	363.2 (335.4;393.2)	-66.9 (-69.2;-64.5)
Goiás	13916.2 (12991.6;14820.9)	800.2 (752;844.9)	19898.4 (18199.2;21777)	320.9 (293.9;350)	-59.9 (-62.8;-56.7)
Maranhão	17377 (15585.8;19607.3)	660 (586.2;752.6)	27215.8 (24986.8;29637.2)	439.9 (404.1;479)	-33.3 (-40;-26)
Mato Grosso	5212.7 (4803.6;5633.7)	705.7 (650.9;759.9)	10002 (9113.3;10867.5)	356.6 (326.3;386.9)	-49.5 (-54.1;-44.5)
Mato Grosso do Sul	6723.6 (6322.8;7131.6)	813.8 (767.3;859.5)	10101.1 (9292.6;10911.3)	384.6 (354.4;414.8)	-52.7 (-56.5;-48.9)
Minas Gerais	81390.3 (76723.4;86023.9)	881.5 (836.4;927.8)	86497.3 (79441.7;93590)	346.9 (318.9;375.4)	-60.6 (-63.5;-57.6)
Pará	18296.6 (16994.5;19445.1)	906.6 (843.3;962.1)	28488.5 (26185.1;30787)	465.3 (428.2;501.5)	-48.7 (-53;-44.3)
Paraíba	14128.8 (12998.2;15518.4)	609.2 (560.5;666.9)	18006.3 (16468.6;19631.7)	392.4 (358.4;427.9)	-35.6 (-42.9;-27.8)
Paraná	51172.4 (48539.5;53822.5)	1204 (1146.1;1259.9)	55096.3 (51203.8;59213.8)	455.8 (424.5;489.8)	-62.1 (-64.8;-59.4)
Pernambuco	41435.4 (39396.4;43702.5)	970.5 (925.1;1020.6)	41014.9 (38043.8;44085.7)	431.5 (400.1;463.9)	-55.5 (-58.6;-52.1)
Piauí	9568.2 (8668;10635.1)	687.6 (623.1;765.2)	14673.1 (13540.8;15959.7)	411.7 (379.6;448)	-40.1 (-45.6;-34)
Rio de Janeiro	95808.8 (90420.4;100946.9)	1069 (1012.6;1122.6)	83858 (77688.3;90710.3)	394.7 (366.4;426.8)	-63.1 (-65.9;-60.3)
Rio Grande do Norte	9503.9 (8745.6;10253.4)	584.6 (538.8;631.1)	11733.2 (10776.4;12756)	319.1 (292.9;346.8)	-45.4 (-50.7;-39.9)
Rio Grande do Sul	60826.8 (57517.2;64097.9)	1027.2 (972.9;1078)	61657.7 (56558.6;66642)	417.5 (382.7;451.1)	-59.4 (-62.2;-56.3)
Rondônia	3034.7 (2783.7;3293.1)	970.7 (899.5;1042.4)	5412.4 (4855.6;6020.7)	415.3 (373.7;461)	-57.2 (-61.8;-51.9)
Roraima	372.6 (336.5;412.4)	795.4 (729;867.3)	1055.5 (934.1;1183.6)	379.8 (337.7;421.7)	-52.2 (-58.4;-45.5)
Santa Catarina	22415.1 (21207.6;23730.7)	1029.7 (974;1083.9)	25161.9 (23015.1;27290.2)	354.4 (325.4;383.2)	-65.6 (-68;-62.8)
São Paulo	164767.5 (155579.3;173984.4)	890.4 (843.4;938)	186699.6 (171816.4;201713.6)	372 (342.1;401.3)	-58.2 (-61;-55.2)
Sergipe	6658.2 (6265.3;7056.5)	781.8 (736.7;828.1)	8987.9 (8329;9671)	443.8 (411.4;476.9)	-43.2 (-47.3;-38.6)
Tocantins	2692 (2368.4;3009.9)	750 (674.7;828.6)	5401.9 (4954.1;5885.6)	408.6 (374.8;444.9)	-45.5 (-51.5;-38.7)
***B.2.3.2- Hemorragia Intracerebral***					
Acre	2042.4 (1946.7;2151.3)	1021.1 (972.1;1070.2)	3907 (3628.3;4180.9)	625.6 (581;667.5)	-38.7 (-43.7;-33.5)
Alagoas	22644.7 (21077;24235.6)	1476.9 (1388.9;1567.2)	27097.9 (25636;28599.9)	855.7 (809.8;904.3)	-42.1 (-46.4;-37.8)
Amapá	1000.2 (936.5;1047.5)	829.7 (774.6;869.6)	3256.8 (2973.7;3497.4)	613.2 (561;657.1)	-26.1 (-31.8;-19.8)
Amazonas	9657.5 (8944.9;10226.1)	1033.8 (957;1093.8)	17298.4 (16028.8;18370.1)	607.4 (564.8;644.9)	-41.2 (-45.5;-36.3)
Bahia	84377.2 (79355.7;90141)	1108.1 (1040.1;1183.5)	104105.8 (98385.8;110053.4)	656.6 (620.6;694)	-40.7 (-45.3;-35.8)
Brasil	1334369 (1303152.8;1373801.4)	1322.1 (1291.8;1358.2)	1326910.8 (1288958.3;1368112.2)	576.9 (560.7;594.9)	-56.4 (-57.8;-55.1)
Ceará	40070.2 (36879.4;43154.2)	893.2 (817;960.7)	52333.9 (49236;55146.4)	533.9 (501.6;562.5)	-40.2 (-45.3;-34.6)
Distrito Federal	9370.1 (8908.1;10046.1)	1179 (1125.2;1252.5)	11358.7 (10528;12284.1)	443.7 (413.3;477.7)	-62.4 (-65;-59.3)
Espírito Santo	27092.1 (26043.2;28443.7)	1651.2 (1587.7;1725.3)	23985.1 (22605.5;25435.7)	557 (525.7;591.2)	-66.3 (-68.3;-64.1)
Goiás	30618.2 (29152.7;32584.3)	1291.1 (1232.1;1366.3)	35332.4 (33203.1;37574)	509.5 (478.7;541.7)	-60.5 (-63;-57.8)
Maranhão	40946.2 (36530.2;45483)	1334.4 (1213.3;1456.3)	48119.1 (44681.6;52607.1)	738.8 (686.2;810.1)	-44.6 (-50.9;-37.9)
Mato Grosso	9481.7 (8758.9;10235.2)	952.6 (879.1;1027.3)	17317.5 (16178.9;18505.5)	529.8 (495.5;566.5)	-44.4 (-49.2;-38.3)
Mato Grosso do Sul	14326.7 (13580.5;15036)	1346.6 (1285.1;1408.5)	18396.2 (17315.1;19515.4)	639.5 (601.6;678.8)	-52.5 (-55.9;-49.1)
Minas Gerais	160746.7 (153946.9;171975.7)	1422.7 (1364.4;1509)	139119.5 (131593;146724)	546.2 (516.9;575.9)	-61.6 (-64.2;-58.9)
Pará	27317.6 (25130.3;29082.4)	1111.4 (1017.5;1182.7)	46066.2 (42810.4;49272.9)	668.3 (622.2;712.9)	-39.9 (-45;-34.3)
Paraíba	23051.3 (21538.9;24766)	954 (892.6;1024.9)	28591.1 (26253.3;30997.8)	629.8 (578.8;682.3)	-34 (-40.8;-26.8)
Paraná	88216.4 (84443;91983)	1633.9 (1570.7;1702)	72564.6 (68483;76843.2)	561.1 (530.1;593.1)	-65.7 (-67.9;-63.3)
Pernambuco	73380.8 (69895.3;76702.7)	1499.7 (1430.9;1568.9)	74615.6 (70543.1;78768.3)	744.4 (703.9;786.5)	-50.4 (-53.4;-47.5)
Piauí	18501.1 (16948.8;20251.5)	1160.3 (1060.5;1264)	23443.1 (22029.8;25610)	651 (611.5;710.8)	-43.9 (-48.8;-38.6)
Rio de Janeiro	201658.8 (193417.1;211873.7)	1858 (1782.2;1943.7)	147613.4 (140085.6;156033.4)	676.3 (641.5;713.3)	-63.6 (-66;-61.1)
Rio Grande do Norte	12099.4 (11196.2;12951.9)	706.5 (651.7;757.1)	17465.8 (16141.3;18793.4)	468.6 (433.9;504.3)	-33.7 (-39.7;-26.6)
Rio Grande do Sul	90116 (85634.7;94313.2)	1235.2 (1173.1;1292.4)	85329.9 (79196.9;90723.5)	570.3 (529.1;605.5)	-53.8 (-56.9;-50.3)
Rondônia	6920.4 (6371.4;7465.9)	1469.9 (1361.9;1583.8)	8998.9 (8019.1;10137.8)	592.7 (529.6;666.4)	-59.7 (-64.6;-53.9)
Roraima	868.7 (786;962.9)	1116.5 (1020.8;1223.5)	1914.8 (1686.6;2190.9)	527.6 (464.5;599.3)	-52.7 (-59.7;-45.1)
Santa Catarina	37531.6 (35812;39073.7)	1347.1 (1285.7;1402.4)	35160.6 (32444.1;37333.3)	451.6 (418.2;478.8)	-66.5 (-68.8;-64.1)
São Paulo	286216.8 (273648.3;299941.9)	1235.3 (1182.4;1289)	259904.1 (245650.4;275069.5)	490.6 (464.5;518.7)	-60.3 (-62.8;-57.4)
Sergipe	10290.6 (9777.3;10791)	1120.9 (1062.3;1176.2)	14532.9 (13755.7;15333.1)	666.3 (631.3;703.4)	-40.6 (-44.7;-35.7)
Tocantins	5825.5 (4890.2;6575)	1189.3 (1037.1;1325.3)	9081.5 (8410.7;9866.8)	633.5 (587.1;688.8)	-46.7 (-52.9;-38.2)
***B.2.3.3- Hemorragia Subaracnóidea***					
Acre	847.7 (773.2;919.3)	290.6 (269.9;311.1)	1283.8 (1183.9;1391)	177.6 (164.1;193.1)	-38.9 (-44.9;-32.4)
Alagoas	7391.3 (6373.7;8529.4)	355.3 (311.7;397.2)	6967.4 (6414.6;7757.1)	205.9 (189.4;228.9)	-42.1 (-50.3;-29.8)
Amapá	332.7 (309.9;365)	200.1 (186;223.5)	1032.5 (948.2;1149)	161.5 (148.4;179.7)	-19.3 (-26.7;-10.9)
Amazonas	2934.8 (2699.5;3205.7)	218.9 (203.2;238.9)	5271.9 (4852.8;5795.8)	157.6 (145.2;173.4)	-28 (-34.4;-20.6)
Bahia	26401.2 (23947.3;28838.7)	280.2 (256.7;306.5)	30862.9 (28723;33700.7)	190.8 (177.6;208.7)	-31.9 (-38.9;-24.6)
Brasil	382092.5 (341738.8;399795.1)	318.4 (287;332.2)	421128.4 (402202.4;445263.2)	181 (173.1;191)	-43.1 (-46.3;-37.2)
Ceará	15001.4 (12391.2;18119.3)	264.2 (225.1;311.9)	16027.3 (14761.6;17474.1)	161.5 (148.7;176)	-38.9 (-49.2;-28.2)
Distrito Federal	3328.5 (2921.2;3564.8)	305.8 (267.9;326.7)	4451.2 (3996.2;4855.6)	150.5 (134.4;164.3)	-50.8 (-55.3;-45.2)
Espírito Santo	7132.8 (5899.9;7604.3)	358.5 (294.5;382.4)	7751.8 (7128.6;8367.7)	176.5 (162.5;190)	-50.8 (-55.4;-39.5)
Goiás	8598.4 (7989.3;9184.5)	287.4 (265.6;307)	11879.3 (10974.1;13114.1)	162.2 (150.1;178.6)	-43.6 (-48.6;-36.8)
Maranhão	16940.2 (13560.2;20200.9)	394 (321;459.7)	15729.1 (14113.5;17089.8)	223.9 (199;243.2)	-43.2 (-50.9;-32.3)
Mato Grosso	3699.9 (3386.4;4012)	262.9 (241.4;287.3)	5989.9 (5522;6551.1)	166.7 (154.3;182)	-36.6 (-43.1;-29.6)
Mato Grosso do Sul	3949.6 (3524;4259.3)	301.8 (272.8;324.2)	5533 (5124.3;6033)	184.7 (171.2;201.1)	-38.8 (-45.1;-31.2)
Minas Gerais	43411.7 (36926.5;46441.3)	334.4 (286;356.7)	45738.5 (42358.9;49050.9)	182.2 (169;195.2)	-45.5 (-50.4;-37.9)
Pará	7934.2 (7204.8;8740.1)	235.4 (216.8;259.9)	14056.5 (12883.9;15453.7)	180.9 (166.2;198.4)	-23.2 (-30.1;-15.8)
Paraíba	7964.3 (6876.5;9209)	295.5 (255.4;339.2)	8313 (7521.9;9221.7)	184.2 (166.7;204.3)	-37.7 (-48.2;-25.5)
Paraná	22236.9 (18814.4;23684.3)	341.8 (287.1;364.5)	23630.8 (21760.6;25484.3)	180.4 (166.5;194)	-47.2 (-52.1;-39.6)
Pernambuco	16056.3 (14930.2;17673.3)	281.4 (262.2;311.8)	20507.5 (19042.9;22982.6)	199.2 (185;222.9)	-29.2 (-35.8;-22.5)
Piauí	6362.7 (5250.5;7430)	307 (258.7;356.2)	6817.4 (6299.7;7358.7)	187.4 (173.4;202.5)	-39 (-47.3;-28.2)
Rio de Janeiro	49986.3 (39896.5;53598.5)	419.4 (335.1;449.1)	42289.2 (38909;45633.2)	200.1 (184.6;215.2)	-52.3 (-56.7;-41.4)
Rio Grande do Norte	4077.6 (3612.7;4762.8)	196.1 (174.2;236.3)	5302.8 (4835.1;6667.4)	139.8 (127.7;175.3)	-28.7 (-37.7;-18.9)
Rio Grande do Sul	21396.6 (19895.4;23508.5)	262.1 (244.7;290.7)	23342.9 (21439;25660.3)	163.1 (150.5;178.8)	-37.8 (-43.1;-31.4)
Rondônia	2118.6 (1933.7;2303)	300.6 (275.8;327.4)	2911.6 (2578.8;3318.4)	170.2 (151;193.1)	-43.4 (-51;-34.1)
Roraima	327.8 (294;368.6)	261.1 (234.8;298.9)	673.8 (592.6;805.6)	150.1 (132.2;178.6)	-42.5 (-50.8;-32.5)
Santa Catarina	9964.3 (9246.9;10542.8)	289.9 (267.1;306.6)	11653.1 (10732.6;13019.9)	144.5 (133.5;160.9)	-50.2 (-54.8;-43)
São Paulo	88540 (77303.1;94399.5)	323.3 (286.2;343.1)	96099.9 (89106.8;103155.3)	181 (168.3;194.1)	-44 (-49.1;-36.7)
Sergipe	3084.4 (2757.9;3439.9)	263.6 (241.5;290.9)	4031.2 (3725.2;4445.7)	172.6 (159.7;190.2)	-34.5 (-42.1;-26.6)
Tocantins	2072.4 (1546;2522.9)	305.7 (242.4;363.9)	2980.1 (2699;3279)	193.6 (175.8;212.5)	-36.7 (-47.4;-21.4)

* Fonte: Estudo Global Burden of Disease 2017, Institute for Health Metrics and Evaluation. ^109^*

**Tabela 2-6:** – Taxas de DALYs padronizadas por idade (por 100 mil) por AVC e subtipos de AVC, no Brasil, 1990 e 2017, e variação percentual das taxa

Causa de morte e grupo etário	1990	2017	Variação percentual (II 95%)
***B.2.3- AVC***			
15-49 anos	900,8 (875,2;928)	427,2 (412;442,6)	-52,6 (-54,4;-50,8)
50-69 anos	6472,1 (6299,3;6661,2)	2930,7 (2823,1;3041,8)	-54,7 (-56,2;-53,1)
5-14 anos	76,6 (65,6;82,7)	34,2 (31;37,5)	-55,3 (-59,6;-49,8)
70+ anos	15340,3 (14965,2;15714,6)	8044,9 (7764,8;8361)	-47,6 (-49;-46,2)
Padronizadas por idade	2511,9 (2457,3;2567,6)	1145,3 (1107,8;1185,3)	-54,4 (-55,5;-53,2)
Todas as idades	1638,6 (1601,9;1675,6)	1225 (1185,3;1267,6)	-25,2 (-27,2;-23,1)
Abaixo de 5	374,3 (302,2;473,7)	60,2 (51,7;70,9)	-83,9 (-88,5;-77,8)
***B.2.3.1- AVC Isquêmico***			
15-49 anos	125,4 (118,1;134)	60,6 (54,5;67,2)	-51,6 (-56,6;-48,1)
50-69 anos	1815 (1728,7;1901,4)	750,4 (693,5;808,9)	-58,7 (-60,9;-56,4)
5-14 anos	9,2 (7,6;11,3)	4,2 (3,3;5,5)	-53,9 (-59,4;-48,5)
70+ anos	8120,6 (7833,9;8406,8)	4071,1 (3858,5;4306,3)	-49,9 (-51,8;-47,8)
Padronizadas por idade	871,4 (841,1;902,4)	387,3 (363,8;411,5)	-55,6 (-57,3;-53,8)
Todas as idades	489,8 (471,5;508,9)	399,7 (375,2;425,1)	-18,4 (-21,8;-15,1)
Abaixo de 5	21,6 (15,5;30,9)	2,7 (2,2;3,4)	-87,4 (-92,3;-79,9)
***B.2.3.2- Hemorragia Intracerebral***			
15-49 anos	551,1 (532,8;582,6)	231,9 (219,7;241,4)	-57,9 (-61,3;-55,8)
50-69 anos	3889,2 (3785,6;4001,8)	1681,4 (1626,7;1736,2)	-56,8 (-58,4;-55,1)
5-14 anos	22,7 (19;25,3)	9,7 (8,4;11)	-57,2 (-64,5;-49,3)
70+ anos	6557,5 (6353,6;6729,9)	3451,2 (3343,6;3564,8)	-47,4 (-49,2;-45,5)
Padronizadas por idade	1322,1 (1291,8;1358,2)	576,9 (560,7;594,9)	-56,4 (-57,8;-55,1)
Todas as idades	893 (872,1;919,4)	626,5 (608,5;645,9)	-29,9 (-32,3;-27,7)
Abaixo de 5	89,5 (56,4;137,1)	10 (7,9;12,6)	-88,8 (-93,9;-79,4)
***B.2.3.3- Hemorragia Subaracnóidea***			
15-49 anos	224,3 (198,4;234,1)	134,6 (127,8;146,1)	-40 (-43,9;-30,9)
50-69 anos	767,9 (694,1;806,4)	499 (472,4;530,5)	-35 (-39,1;-28,4)
5-14 anos	44,7 (38;48,3)	20,3 (18,3;22,3)	-54,7 (-58,9;-47,7)
70+ anos	662,2 (627,5;712,9)	522,6 (484,9;553)	-21,1 (-27,7;-15,6)
Padronizadas por idade	318,4 (287;332,2)	181 (173,1;191)	-43,1 (-46,3;-37,2)
Todas as idades	255,7 (228,7;267,6)	198,8 (189,9;210,2)	-22,2 (-26,8;-13,2)
Abaixo de 5	263,3 (204;315,7)	47,5 (40,8;56,2)	-82 (-86,4;-74)

* Fonte: Estudo Global Burden of Disease 2017, Institute for Health Metrics and Evaluation. ^109^*

**Tabela 2-7:** – Número de DALYs, taxas de DALYs padronizadas por idade (por 100 mil) por AVC e subtipos de AVC, para homens, no Brasil e unidades federativas, 1990 e 2017, e variação percentual das taxas

Causa de morte e localização	1990		2017		Variação percentual (II 95%)
	Número (II 95%)	Taxa (II 95%)	Número (II 95%)	Taxa (II 95%)	
***B.2.3- AVC***					
Acre	2185.8 (2055.7;2310)	2076.4 (1959.4;2190.7)	4056.6 (3718;4393)	1351.9 (1238.4;1462.5)	-34.9 (-40.6;-28.6)
Alagoas	23037.9 (21383.3;24816.4)	3180.3 (2982.5;3389.5)	25242.2 (23619.6;26883.8)	1808.9 (1691.8;1922.6)	-43.1 (-47.4;-38.5)
Amapá	1034.6 (965.2;1094.9)	1809.2 (1692.8;1912.7)	3381.8 (3134.1;3607)	1374.3 (1282.1;1461.9)	-24 (-29.8;-17.9)
Amazonas	9553.6 (8816.9;10213.4)	2093.4 (1938.6;2239.5)	17349.2 (15990.9;18542.4)	1276.7 (1184.2;1362.4)	-39 (-44;-33.1)
Bahia	79746.6 (73741.8;86039.8)	2199.7 (2029.8;2372.4)	105759.2 (99250.7;112353.9)	1484.6 (1393.9;1577.1)	-32.5 (-38.3;-26.2)
Brasil	1340726.1 (1301608;1381197.7)	2901.8 (2824.3;2983.3)	1365881.1 (1320048.1;1415334.4)	1334.5 (1289.5;1381.5)	-54 (-55.4;-52.5)
Ceará	40155.1 (36356.6;44353)	1867.9 (1704.3;2040.7)	52142.8 (49017.4;55405.8)	1187.3 (1115.9;1260.7)	-36.4 (-42.4;-29.7)
Distrito Federal	8537.6 (8039.6;9059.3)	2500.7 (2380;2633.5)	11737.5 (10824.2;12680.1)	1133 (1049;1220.2)	-54.7 (-58.4;-50.9)
Espírito Santo	27222.3 (26107.5;28325.5)	3603.3 (3461.5;3739.1)	24872.8 (23306.7;26481.7)	1282 (1202.4;1361.2)	-64.4 (-66.8;-61.8)
Goiás	29376.9 (27812.5;31309.7)	2478.4 (2351.1;2632)	36251.9 (33907.6;38650.9)	1128.3 (1056.4;1200.3)	-54.5 (-57.7;-50.9)
Maranhão	48618.1 (43268.6;54586.2)	3233.4 (2920.4;3559.2)	52331.8 (48765.6;56040.7)	1736.2 (1619.1;1857)	-46.3 (-52.5;-39.3)
Mato Grosso	10626.9 (9676.9;11652.5)	2101.4 (1919.4;2294.5)	18983.8 (17675.4;20267.4)	1183.4 (1106.4;1259.7)	-43.7 (-49;-37.5)
Mato Grosso do Sul	14122.2 (13298.1;14951)	2705.6 (2569.5;2851.5)	18664 (17425.5;19960.7)	1387.8 (1299.8;1482.5)	-48.7 (-52.3;-44.6)
Minas Gerais	161794.5 (154754.4;170766.6)	3124.2 (2993.3;3285.8)	143536.1 (135085.5;153049.9)	1231.9 (1157.6;1313.9)	-60.6 (-63.5;-57.7)
Pará	28382.9 (26206.8;30473.3)	2437.9 (2254.7;2606.9)	51273.2 (47548.3;54706.6)	1571.3 (1462.4;1670.3)	-35.5 (-41.2;-29.3)
Paraíba	22193 (20368;24263.9)	1936.3 (1764.3;2121.9)	27109.7 (24423.5;30026.9)	1350.3 (1216.8;1495.6)	-30.3 (-38.8;-20.4)
Paraná	92749.7 (88831.5;96784.9)	3678.5 (3538.6;3825.8)	80945 (75854.2;86058.7)	1400.7 (1313.7;1487)	-61.9 (-64.5;-59.2)
Pernambuco	65395.8 (62156.5;68861.5)	2991 (2847;3142.5)	69666.6 (65443.1;74126)	1614.1 (1513.1;1713.9)	-46 (-49.6;-42.1)
Piauí	19669.4 (17738.3;21781.8)	2619.3 (2388.7;2872.8)	23916.7 (22435.2;25778.9)	1448.6 (1356.9;1561.6)	-44.7 (-50;-38.8)
Rio de Janeiro	184276.5 (175451.6;192077)	3962.5 (3782.2;4120.9)	137791.2 (129209;147280.8)	1484.8 (1394;1583.2)	-62.5 (-65.1;-59.8)
Rio Grande do Norte	13443.1 (12350.1;14560.8)	1633.8 (1495.3;1773.6)	18039.1 (16816.2;19404)	1085.1 (1009.3;1170.2)	-33.6 (-40.6;-26.1)
Rio Grande do Sul	90124.8 (85087.9;94419.2)	2897.9 (2730.7;3029.6)	85557 (78513.9;91514.3)	1310.6 (1204.4;1399.5)	-54.8 (-58.2;-51.3)
Rondônia	7265.8 (6579.4;8016.9)	3011.3 (2742.2;3291.6)	9560.1 (8267.2;11057.2)	1294.1 (1124.2;1488.5)	-57 (-63.3;-49.7)
Roraima	949.2 (833.8;1085.1)	2284.8 (2043.1;2564.1)	2144.1 (1803.1;2514.8)	1185.9 (1008.1;1374.6)	-48.1 (-57.3;-38.1)
Santa Catarina	37772.9 (35903.6;39627.8)	3023.9 (2877.4;3170.4)	37174.2 (34286.7;39901.5)	1075 (995.5;1150.9)	-64.4 (-67;-61.7)
São Paulo	306031.7 (291022;320718.4)	2950.4 (2815.7;3084)	284773.8 (266487.2;302539.1)	1234.2 (1156.7;1308.1)	-58.2 (-61;-55)
Sergipe	10495.6 (9904.8;11088.7)	2433.8 (2298.2;2564.6)	14207 (13364.2;15173.1)	1497.7 (1410.4;1596.9)	-38.5 (-43.4;-33.2)
Tocantins	5963.4 (4989.1;6786.8)	2363.3 (2064.7;2648.7)	9413.7 (8582.8;10360.7)	1316 (1200;1448.6)	-44.3 (-51.5;-34.9)
***B.2.3.1- AVC Isquêmico***					
Acre	618.6 (570.6;666.7)	717.8 (663.9;771.4)	1173.2 (1053.7;1293.6)	441.9 (397;487.4)	-38.4 (-45.2;-31.1)
Alagoas	7174.6 (6632.2;7849.1)	1129.9 (1044.1;1234.1)	8207.6 (7511.2;8930.5)	631.6 (579.3;686.7)	-44.1 (-50.1;-37.8)
Amapá	321.8 (294.1;347.8)	700 (641.2;755.6)	983.4 (886.2;1080.5)	479 (432.3;523.2)	-31.6 (-38.3;-24.5)
Amazonas	2794 (2546.9;3043.9)	750.3 (685.8;817.2)	4980.4 (4506.6;5491.5)	422.3 (382.8;464.2)	-43.7 (-49.3;-37.3)
Bahia	23360.8 (21326.4;25677.3)	723.9 (659.9;793.4)	32950.3 (30333.4;35703.4)	486 (447.3;526.4)	-32.9 (-40;-25.2)
Brasil	399936.6 (384258.7;417610.5)	1011 (973.7;1050.4)	444686.8 (419137.4;471530)	463.4 (436.9;490.2)	-54.2 (-56.5;-52.2)
Ceará	11960.3 (10725.4;13374.7)	616.7 (550.4;690.6)	17787 (16310.4;19333.8)	418.8 (383.3;456.6)	-32.1 (-41;-23.5)
Distrito Federal	2081.9 (1927.3;2271.5)	863.5 (801.5;934.9)	3486.6 (3111.4;3853.3)	415.1 (374.3;456)	-51.9 (-56.9;-46.2)
Espírito Santo	8136.6 (7612.8;8726.9)	1280.8 (1203.2;1368.2)	7872 (7206.4;8608.3)	435.9 (399.3;475.3)	-66 (-69.1;-62.6)
Goiás	7772.7 (7148.3;8470.7)	799.3 (741.5;864.3)	10856.7 (9840;11962.2)	369.8 (336.1;405.8)	-53.7 (-58.1;-48.6)
Maranhão	11361.5 (10150.7;12898.4)	901.9 (799.8;1020.4)	16393.4 (14900.7;17870.1)	578.9 (527.5;630.6)	-35.8 (-43.4;-26.8)
Mato Grosso	3275.8 (2935.7;3611.7)	809.8 (728.8;890)	5907.8 (5344;6475.2)	410.9 (371.6;449.3)	-49.3 (-54.9;-43.2)
Mato Grosso do Sul	4003.7 (3719.2;4320.3)	920.9 (857.5;990.1)	5616.4 (5139.2;6154.2)	451.4 (413.3;493.6)	-51 (-55.7;-45.4)
Minas Gerais	45397.8 (42188;49142.4)	1032 (966.8;1106.1)	45377.1 (41325.5;49776.8)	404.9 (369;443.4)	-60.8 (-64.6;-56.6)
Pará	9746.8 (8897.6;10553.4)	988.9 (905.2;1067)	16640.4 (15023.9;18274.6)	567 (512.9;618.9)	-42.7 (-48.5;-35.9)
Paraíba	7338.7 (6537.4;8331.7)	662.5 (589.7;752.4)	8861 (7875.9;9862.6)	451.6 (402;502.2)	-31.8 (-42.2;-19.6)
Paraná	29844.1 (27803.5;31821.9)	1395.8 (1309.4;1479.1)	29745 (27186.2;32365.5)	545.8 (499;592.4)	-60.9 (-64.7;-56.8)
Pernambuco	20621.5 (19138.5;22088.7)	1056.1 (986.5;1126.5)	20528.9 (18761.4;22301)	512.2 (469;555.5)	-51.5 (-56.3;-46.5)
Piauí	5761.5 (5099.4;6525)	885.1 (784;1003.8)	7811.8 (7126.5;8529.2)	487.7 (445.2;532.9)	-44.9 (-51.4;-37.5)
Rio de Janeiro	49857.6 (46267.7;53745.2)	1274.3 (1191;1361.4)	41594.5 (37885.7;45512.7)	476.1 (433.5;520.1)	-62.6 (-66.3;-58.8)
Rio Grande do Norte	5114.5 (4628.4;5664.3)	660.2 (597.4;731)	6177.2 (5613.2;6753.1)	387.4 (351.2;423.7)	-41.3 (-48.9;-33.4)
Rio Grande do Sul	31368.4 (29142.6;33607.5)	1192.5 (1112.2;1269.8)	29881.3 (26896.7;32904.7)	479.7 (434.4;526.7)	-59.8 (-63.4;-55.4)
Rondônia	1976.1 (1763.6;2194.1)	1102.7 (997.2;1205.7)	3130.3 (2728.8;3606.5)	471.6 (411.8;543.1)	-57.2 (-63.5;-49.8)
Roraima	232.6 (203.3;266.8)	829.1 (738.7;936)	622.5 (532.3;717.9)	422.1 (361.6;485.3)	-49.1 (-57.1;-39.7)
Santa Catarina	12066 (11272.6;12969.4)	1161.9 (1090.7;1245.2)	12855.1 (11617.8;14085.1)	406.9 (370.4;444.8)	-65 (-68.3;-61.2)
São Paulo	92643.3 (86120.5;99963.6)	1076.5 (1003.8;1149)	97487.5 (88497;106189.9)	452.3 (411.4;491.7)	-58 (-62;-53.9)
Sergipe	3514.4 (3243.3;3784.8)	896.1 (827.4;963)	4688.6 (4301.9;5103)	537 (492.6;584.8)	-40.1 (-46.2;-32.9)
Tocantins	1590.9 (1380.1;1806.6)	799.2 (704.9;903.2)	3070.7 (2762.7;3412.2)	454.9 (409.6;504.6)	-43.1 (-50.8;-33.4)
***B.2.3.2- Hemorragia Intracerebral***					
Acre	1167.7 (1091.3;1249.8)	1103.5 (1027.3;1177)	2307.8 (2068.3;2536.5)	749.5 (674.7;823.3)	-32.1 (-39.6;-23.9)
Alagoas	12772.1 (11548.1;13954.3)	1755.3 (1608.7;1896.2)	14334.6 (13275.7;15483.4)	1004.8 (929.5;1085.5)	-42.8 (-48.1;-36.7)
Amapá	560.5 (514.3;598.7)	932.7 (858.7;997.3)	1937.6 (1732.9;2099.3)	748.9 (677.5;810.1)	-19.7 (-27.5;-11)
Amazonas	5416 (4917.7;5858.5)	1146.7 (1043.6;1240.1)	10027.2 (8951.6;10869.8)	713.8 (645.2;772.7)	-37.8 (-44;-30.6)
Bahia	44882.2 (40975.9;49072.8)	1234.7 (1126.8;1348.4)	59717.2 (55175.3;64266.8)	825.2 (762.5;886.5)	-33.2 (-40.1;-25.3)
Brasil	770369.3 (745080;807453.6)	1601 (1552.2;1665.3)	746738 (720304;771926)	709.9 (684.6;733.7)	-55.7 (-57.5;-53.9)
Ceará	21836.7 (19497.2;24283.5)	1026.3 (913.7;1136)	27995 (25907.6;30104.9)	631 (584.4;678.5)	-38.5 (-44.8;-30)
Distrito Federal	5129.2 (4782;5672.3)	1378.5 (1293.1;1493.8)	6413 (5860.8;7055.5)	577.3 (530.7;632.9)	-58.1 (-62.2;-53.6)
Espírito Santo	15950.1 (15082.9;17014.5)	2001.8 (1896;2121.2)	13712.2 (12714;14769.9)	687.1 (637.9;738.7)	-65.7 (-68.6;-62.6)
Goiás	17762.5 (16634;19235.8)	1430.3 (1342.8;1543.6)	20310.6 (18792.9;21876.5)	613.1 (566.7;659.2)	-57.1 (-61.3;-52.8)
Maranhão	28903.2 (24480.3;33872.2)	1930 (1685.5;2188.7)	28870.1 (26565.9;31653.8)	946.4 (871.3;1037.4)	-51 (-58.3;-42.1)
Mato Grosso	5628.1 (5070.6;6250.4)	1062.2 (956.7;1176.1)	10360.1 (9507.4;11268.1)	622.6 (573.7;675.4)	-41.4 (-48.1;-33.7)
Mato Grosso do Sul	8383 (7809.6;8980.7)	1528.5 (1437.3;1632.1)	10743.1 (9937.4;11597)	776.6 (718.3;836.1)	-49.2 (-53.7;-44.1)
Minas Gerais	96380.6 (91111.2;106323.6)	1776.3 (1681.8;1936)	78424.5 (72806.4;84694.7)	659.8 (613.3;712.3)	-62.9 (-66.6;-59.4)
Pará	15142.2 (13771.6;16408.1)	1245 (1128.4;1348.2)	28065.2 (25497.2;30355.1)	832.9 (758.6;901.5)	-33.1 (-40.6;-24.6)
Paraíba	11940.1 (10701.6;13143.1)	1052.4 (943.8;1157.3)	15086.6 (13425.1;17023.7)	746.3 (664.9;841.2)	-29.1 (-39;-17.3)
Paraná	52954.5 (49873.2;56180.2)	1974.1 (1867.5;2087)	41469.1 (38186.9;44696.4)	694.9 (641.3;748.6)	-64.8 (-68.2;-61.6)
Pernambuco	38213.7 (35818.5;40711.7)	1697.4 (1591.6;1806.2)	40983.9 (38031;44233.3)	927.6 (861.4;998.2)	-45.4 (-49.9;-40.2)
Piauí	11080.4 (9834.8;12452)	1461.3 (1308.5;1628.8)	13195.7 (12198.6;14309.5)	791.9 (731.9;859.9)	-45.8 (-51.9;-38.3)
Rio de Janeiro	112722 (106431.2;121878.2)	2297.5 (2175.7;2458.4)	79599.8 (73519.4;85665.3)	832.6 (772;895.4)	-63.8 (-66.8;-60.5)
Rio Grande do Norte	6460 (5806.7;7074.2)	795 (712.9;873.8)	9624.8 (8747.5;10531.3)	571.4 (519.9;624.3)	-28.1 (-37.1;-17.4)
Rio Grande do Sul	49620.5 (46236.1;52719.3)	1469.8 (1370.4;1556.4)	46196.2 (41693.4;49971.8)	687 (620.3;740.7)	-53.3 (-57.8;-48.8)
Rondônia	4291.7 (3827.1;4779.5)	1649.2 (1485.9;1829.5)	5219.4 (4433.5;6116.6)	681.4 (579.1;794.2)	-58.7 (-65.9;-50.3)
Roraima	540.1 (467.9;624.4)	1208.3 (1063.9;1371.2)	1177.9 (971;1399.6)	616 (514.5;726.2)	-49 (-59.4;-37.6)
Santa Catarina	21310.9 (20042.4;22571.8)	1599.1 (1505.4;1692.3)	19391.8 (17418.6;21070.9)	538.4 (485.1;583.1)	-66.3 (-69.7;-63)
São Paulo	172309 (162089.8;184997.1)	1566.5 (1474;1673.6)	148579.1 (137505.7;159473)	622.3 (576.6;667)	-60.3 (-63.5;-56.5)
Sergipe	5586 (5171.6;5990.8)	1301.8 (1199.4;1396)	7869.5 (7245.8;8471.6)	807.4 (743.9;870.3)	-38 (-44.4;-30.7)
Tocantins	3426.4 (2825.3;3946.7)	1306.7 (1114.8;1488.8)	5125.9 (4593.1;5697.7)	703.9 (629.4;782.3)	-46.1 (-53.8;-35.7)
***B.2.3.3- Hemorragia Subaracnóidea***					
Acre	399.5 (338.4;452.9)	255 (224.7;282.1)	575.6 (512.3;647.3)	160.5 (142.9;180.2)	-37.1 (-46.3;-25.3)
Alagoas	3091.2 (2560.5;3728.8)	295.1 (256.9;338.7)	2700.1 (2424.9;3128.4)	172.5 (155.2;198.2)	-41.5 (-51.6;-28)
Amapá	152.3 (137.1;172.9)	176.6 (159.2;208.9)	460.9 (405.1;563.3)	146.5 (129;179.3)	-17.1 (-28.4;-3.6)
Amazonas	1343.6 (1203.1;1505.9)	196.3 (176.8;224.8)	2341.6 (2099;2674.1)	140.6 (126.5;160.4)	-28.4 (-37.6;-17.3)
Bahia	11503.7 (10112.3;12962.3)	241.1 (213.5;272.3)	13091.8 (11817.2;15500)	173.4 (156.8;204.8)	-28.1 (-37.4;-15.5)
Brasil	170420.1 (137803.5;181911.2)	289.9 (237.9;307.2)	174456.3 (165617.4;190897.1)	161.2 (152.9;175.4)	-44.4 (-48.2;-31.2)
Ceará	6358.1 (4960.5;8487.7)	224.9 (181.7;281.6)	6360.8 (5714;7533.9)	137.4 (123.6;162)	-38.9 (-51.8;-23.8)
Distrito Federal	1326.5 (1012.9;1471.9)	258.7 (192.2;284.4)	1837.9 (1527.4;2085.5)	140.6 (113.6;158.6)	-45.7 (-52.6;-36.5)
Espírito Santo	3135.6 (2225.9;3470.1)	320.7 (225.1;353.4)	3288.6 (2923.6;3700.8)	159 (141.6;178.1)	-50.4 (-57.2;-31.6)
Goiás	3841.6 (3321.9;4212.2)	248.8 (213.8;272.9)	5084.6 (4525.1;5969.8)	145.5 (130.1;169.2)	-41.5 (-48.9;-27.5)
Maranhão	8353.4 (6626.3;10082.5)	401.5 (324.6;464)	7068.2 (6109.5;7958.4)	210.9 (181.9;237.7)	-47.5 (-55.7;-34.1)
Mato Grosso	1723 (1532.7;1937.7)	229.3 (203.9;259.4)	2715.9 (2409.9;3105)	149.9 (133.5;169.8)	-34.6 (-43.9;-24.4)
Mato Grosso do Sul	1735.6 (1459.8;1929.6)	256.1 (216.3;282.8)	2304.4 (2053.5;2587.7)	159.7 (142.7;178.4)	-37.7 (-46.5;-26.4)
Minas Gerais	20016 (14378.2;22114)	315.9 (228;348.2)	19734.6 (17598.5;21729.3)	167.2 (149.8;183.9)	-47.1 (-53.4;-30.5)
Pará	3493.9 (3052.8;3985.1)	204 (180.5;243)	6567.6 (5796.3;7642.6)	171.5 (151.3;200.7)	-15.9 (-26.5;-2.6)
Paraíba	2914.1 (2445;3552.5)	221.5 (186.3;261.9)	3162 (2739.4;3774.1)	152.3 (132;181.2)	-31.2 (-44.8;-12.3)
Paraná	9951.1 (7039.7;10916.8)	308.7 (213.4;339.7)	9730.8 (8612.3;10810.2)	160 (141.5;176.9)	-48.2 (-54.6;-32.2)
Pernambuco	6560.6 (5947.2;7353.4)	237.5 (216.1;264.8)	8153.8 (7346.1;9731.2)	174.3 (157.3;207.5)	-26.6 (-35.5;-15.6)
Piauí	2827.6 (2252.6;3497.2)	273 (224.6;324.2)	2909.2 (2626.4;3236.3)	168.9 (152.7;187.7)	-38.1 (-49;-24.2)
Rio de Janeiro	21696.8 (12606.3;24282.3)	390.7 (228.4;436.3)	16596.8 (14887;18587.5)	176.1 (157.6;196.4)	-54.9 (-61.3;-27.9)
Rio Grande do Norte	1868.7 (1569.2;2321.5)	178.6 (151.8;242.9)	2237 (1953.7;3000.2)	126.3 (110.4;169.3)	-29.3 (-41.4;-13.1)
Rio Grande do Sul	9135.9 (8004;9984.6)	235.6 (207.7;257.1)	9479.5 (8496.8;10627.1)	143.9 (129.5;161.1)	-38.9 (-46;-29.1)
Rondônia	998 (861.6;1120.5)	259.4 (226.5;292.9)	1210.5 (998.6;1457.5)	141.1 (116.3;169)	-45.6 (-56.4;-32.5)
Roraima	176.6 (151.6;207.6)	247.4 (214.1;288.5)	343.6 (283.1;418.6)	147.7 (122.5;178.1)	-40.3 (-52.6;-26)
Santa Catarina	4396 (3777.2;4803.4)	262.9 (223.5;286.9)	4927.3 (4405.4;5845.1)	129.8 (117;151.9)	-50.6 (-57.1;-36.4)
São Paulo	41079.4 (30860;45135.8)	307.4 (238.8;335.9)	38707.2 (35088.4;43735.2)	159.6 (144.9;178.4)	-48.1 (-54.5;-31.5)
Sergipe	1395.2 (1215.1;1623.4)	235.9 (209.6;267.2)	1648.9 (1489.4;1907.3)	153.3 (138.8;176.8)	-35 (-44.4;-23.1)
Tocantins	946.1 (683.9;1191.2)	257.4 (195.8;315.3)	1217 (1071.4;1397.6)	157.3 (138.6;181.4)	-38.9 (-51.6;-20.1)

* Fonte: Estudo Global Burden of Disease 2017, Institute for Health Metrics and Evaluation. ^109^*

**Tabela 2-8:** – Número de DALYs, taxas de DALYs padronizadas por idade (por 100 mil) por AVC e subtipos de AVC, para mulheres, no Brasil e unidades federativas, 1990 e 2017, e variação percentual das taxas

Causa de morte e localização	1990		2017		Variação percentual (II 95%)
	Número (II 95%)	Taxa (II 95%)	Número (II 95%)	Taxa (II 95%)	
***B.2.3- AVC***					
Acre	1767.8 (1667.5;1870.6)	1862.6 (1754.2;1968.1)	3210.2 (2987;3441.9)	1026.8 (954.3;1102.8)	-44.9 (-49.1;-40.5)
Alagoas	20195.9 (18814.5;21710)	2493.7 (2346.1;2633.1)	24797.1 (23207.9;26570.3)	1438.5 (1347.2;1540)	-42.3 (-46.8;-36.9)
Amapá	885.9 (829.3;941.5)	1535.4 (1426.6;1629.7)	2656 (2406.8;2886.7)	998.8 (904.3;1088)	-34.9 (-40.6;-29)
Amazonas	8123.3 (7479.5;8682.2)	1784.6 (1640.3;1906.5)	14351.6 (13285.1;15406.9)	1007.4 (932.8;1079.1)	-43.5 (-48.1;-38.3)
Bahia	77106.2 (71098.5;83950.6)	1933.3 (1780.5;2103.5)	89411.6 (83626.9;95362.2)	1033.8 (966.7;1102)	-46.5 (-51.4;-41.3)
Brasil	1107653.4 (1072336.2;1140018.5)	2162.9 (2095.1;2225.5)	1228780.5 (1175631.6;1283687.7)	990.7 (947.7;1034.7)	-54.2 (-55.9;-52.7)
Ceará	37337.5 (33441.1;40681.5)	1555.4 (1404.6;1695.6)	51423.9 (47690.4;54659.7)	951.5 (881.9;1012.3)	-38.8 (-44.5;-32.1)
Distrito Federal	8067.4 (7639.3;8468)	2039.3 (1936;2134.4)	10665.5 (9807.7;11556.2)	770.8 (709.6;836.4)	-62.2 (-65.4;-58.8)
Espírito Santo	21284.7 (20412.8;22164.8)	2644.9 (2544.4;2750.1)	21595.1 (20097.5;23121.8)	941.9 (877;1008)	-64.4 (-67;-61.7)
Goiás	23755.9 (22547.1;25022.6)	2280.3 (2172.2;2397.1)	30858.1 (28577.4;33385.6)	870.2 (806.8;940.7)	-61.8 (-64.7;-58.9)
Maranhão	26645.2 (23289;30127.1)	1598.1 (1391.9;1827.3)	38732.2 (35890.3;42214.2)	1118.5 (1036;1221.3)	-30 (-37.8;-20.8)
Mato Grosso	7767.3 (7044.7;8529.5)	1700 (1544;1865.8)	14325.6 (13207.9;15664.7)	918.2 (846.6;1003.1)	-46 (-52.2;-38.9)
Mato Grosso do Sul	10877.7 (10222.6;11460.6)	2195.2 (2083.8;2306.8)	15366.3 (14302.6;16510.3)	1046.1 (973.5;1122.9)	-52.3 (-56.3;-48.4)
Minas Gerais	123754.1 (117958.9;129630.2)	2196.3 (2102.1;2298.1)	127819.1 (119352.2;136487.9)	939 (876.7;1002.2)	-57.2 (-60.1;-54.4)
Pará	25165.5 (23222.5;26905.8)	2068.3 (1909.5;2211.6)	37338 (34648.9;39990.2)	1071.3 (992.2;1149.1)	-48.2 (-52.7;-43.4)
Paraíba	22951.4 (21103.1;24889.3)	1790 (1649;1937.3)	27800.7 (25165.5;30858.9)	1093.1 (988.4;1214.1)	-38.9 (-45.7;-30.9)
Paraná	68876 (65849.9;71792.3)	2694.2 (2581.2;2801.1)	70346.7 (65904.8;75129.8)	1027.9 (964.2;1097.5)	-61.8 (-64.3;-59.1)
Pernambuco	65476.7 (62212.1;68590.4)	2548.1 (2424;2666.7)	66471.3 (61893.2;70853.2)	1189.6 (1108.5;1266.9)	-53.3 (-56.3;-50)
Piauí	14762.7 (13259.2;16152.3)	1745.7 (1576.7;1913.4)	21016.8 (19347.1;23129.4)	1080.9 (996;1188.6)	-38.1 (-44;-30.8)
Rio de Janeiro	163177.5 (155826.3;170272.8)	2857.2 (2732.3;2972.6)	135969.3 (127343.8;144657.3)	1113.5 (1043.6;1182.9)	-61 (-63.6;-58.3)
Rio Grande do Norte	12237.7 (11243.8;13264.5)	1354.1 (1243.1;1464.4)	16462.8 (15243.4;17811.1)	800.9 (741;867.3)	-40.9 (-46.3;-34.5)
Rio Grande do Sul	82214.5 (78371.8;85849.8)	2219.2 (2112.8;2314.9)	84773.5 (78795.6;90367.8)	1020.9 (950.7;1088.9)	-54 (-57.2;-50.6)
Rondônia	4807.8 (4391.3;5259.5)	2385 (2193.9;2590.8)	7762.8 (6762.2;8807.3)	1058.4 (926.2;1198.1)	-55.6 (-62.1;-48.7)
Roraima	619.9 (547.3;696.3)	2014.6 (1792.5;2238)	1500.1 (1285.1;1737.4)	914.8 (787.5;1048.3)	-54.6 (-62;-45.2)
Santa Catarina	32138 (30574;33582.2)	2345.2 (2228.7;2447.9)	34801.4 (32383.6;37454.8)	846.2 (788.9;910.2)	-63.9 (-66.6;-61.2)
São Paulo	233492.6 (222621.3;243756.9)	2013.9 (1920.5;2105.9)	257929.8 (239779.5;274998.1)	894 (832.2;952.5)	-55.6 (-58.7;-52.6)
Sergipe	9537.5 (8911.3;10080.5)	1935.3 (1815.6;2042.8)	13345 (12402.1;14314.6)	1112.9 (1034.8;1191.9)	-42.5 (-47.1;-37.1)
Tocantins	4626.6 (3885.4;5271.5)	2097.4 (1835.7;2348.9)	8049.8 (7349.6;8763.6)	1151.2 (1051.9;1253.8)	-45.1 (-52.1;-36.8)
***B.2.3.1- AVC Isquêmico***					
Acre	444.9 (409.5;479.3)	608.7 (561.8;654.7)	902.7 (811.5;996.9)	326.8 (294.5;360.3)	-46.3 (-51.5;-40.3)
Alagoas	6023.2 (5547.1;6516)	856.7 (788.5;926.5)	7766.5 (7055.1;8495.2)	470.6 (428;514.9)	-45.1 (-50.8;-38.9)
Amapá	265.9 (242.6;289.3)	581.6 (530;629.3)	765.2 (674.4;854.7)	336.3 (297;375.7)	-42.2 (-48.1;-35.8)
Amazonas	2290.6 (2070;2488.4)	624 (566.8;677.8)	4150 (3727.4;4582.2)	329.5 (295.8;363.5)	-47.2 (-52.5;-41.2)
Bahia	22713.7 (20772.4;24878.4)	623.8 (569.8;683.6)	27251.9 (24803.3;29866.6)	310.7 (282.1;341)	-50.2 (-55.4;-44.5)
Brasil	331981.3 (316368.4;347711)	747.7 (713.7;780.1)	401935.6 (371989.2;433351)	326.3 (302.2;351.8)	-56.4 (-58.8;-54.1)
Ceará	10460.7 (9309.4;11614.4)	483.2 (430.3;536)	17418.5 (15794.8;18990.5)	315.7 (285.6;344.3)	-34.7 (-42.2;-25.5)
Distrito Federal	1824.5 (1676.1;1966.4)	685.8 (636.7;737.7)	3106.5 (2760.9;3501.9)	266.2 (237.8;298.3)	-61.2 (-65.4;-56.8)
Espírito Santo	6145.4 (5729.6;6600.7)	927.7 (871.2;989.9)	6859.1 (6208.3;7536.9)	304 (275.3;334.3)	-67.2 (-70.2;-63.9)
Goiás	6143.4 (5644.8;6602.7)	804.8 (746.4;862.9)	9041.8 (8039.9;10070.3)	277 (247.5;307)	-65.6 (-69;-61.8)
Maranhão	6015.4 (5053;7099.9)	444.2 (367.5;530.1)	10822.4 (9615.6;12156.4)	324.4 (288.3;364.7)	-27 (-37.1;-14.1)
Mato Grosso	1936.9 (1730.7;2144.1)	579.9 (519.9;639.6)	4094.2 (3653.7;4555.7)	300.5 (269;333.6)	-48.2 (-54.3;-40.9)
Mato Grosso do Sul	2719.9 (2519.2;2923.8)	696.2 (644.8;746.8)	4484.7 (4031.8;4957.1)	324.5 (292.4;358.5)	-53.4 (-58.3;-48.2)
Minas Gerais	35992.5 (33442.6;38488.3)	745.3 (697.4;793.9)	41120.2 (37036.1;45138.4)	296.9 (267;326.4)	-60.2 (-63.8;-56.4)
Pará	8549.8 (7800.8;9221)	821.7 (750.3;883.5)	11848 (10668.4;13072.2)	370.9 (334.4;408.8)	-54.9 (-60;-49.8)
Paraíba	6790.1 (6100.7;7542.4)	562.3 (505.8;621.7)	9145.2 (8214.6;10210.5)	345.4 (310;386.5)	-38.6 (-46.9;-30)
Paraná	21328.3 (19837.5;22825.2)	1018.3 (954.7;1085.5)	25351.3 (22978.4;27818.6)	381.4 (346;418.7)	-62.5 (-65.9;-58.8)
Pernambuco	20813.8 (19337.3;22311.5)	897.4 (835.9;959.7)	20486 (18571.5;22484.9)	371.3 (336.9;407.6)	-58.6 (-62.6;-54.3)
Piauí	3806.7 (3359.2;4298.2)	518 (458.2;583.8)	6861.3 (6175.8;7614.2)	348.2 (313.2;386.4)	-32.8 (-40.7;-24)
Rio de Janeiro	45951.2 (42906.6;49127.3)	911.1 (852.2;970.1)	42263.4 (38418.9;46502.1)	336.1 (305.4;369.6)	-63.1 (-66.4;-59.5)
Rio Grande do Norte	4389.4 (3954;4826.9)	516.8 (465.8;567.9)	5556.1 (4975.4;6161.1)	265.8 (237.2;294.6)	-48.6 (-54.5;-41.7)
Rio Grande do Sul	29458.4 (27295.1;31550.4)	895.7 (833.5;953.9)	31776.4 (28691.4;34814.6)	367.6 (331.6;403.4)	-59 (-62.7;-55.1)
Rondônia	1058.6 (955.1;1174.3)	804 (731.9;882.3)	2282.1 (1981;2588.8)	357.4 (311.9;404)	-55.5 (-62.1;-48.7)
Roraima	140 (122.6;157.4)	739.9 (656.3;827)	433 (371.9;499.1)	331.8 (287.4;379.6)	-55.2 (-62.2;-46.2)
Santa Catarina	10349.1 (9571.8;11121)	910.1 (847;972.5)	12306.8 (11098;13645.2)	310.4 (281;342.7)	-65.9 (-69.2;-62.4)
São Paulo	72124.2 (66732.6;77418.9)	733.7 (681.9;784.3)	89212.1 (80399.2;97949)	310.1 (280.1;340.3)	-57.7 (-61.6;-53.7)
Sergipe	3143.7 (2870.4;3420.7)	683.8 (624.9;743.8)	4299.2 (3883.7;4707.7)	372 (335.7;406.8)	-45.6 (-51.6;-39.2)
Tocantins	1101.1 (958.3;1252.5)	685.3 (606.7;768.8)	2331.2 (2078.4;2585.9)	360.1 (321.8;399.9)	-47.5 (-54.6;-39.4)
***B.2.3.2- Hemorragia Intracerebral***					
Acre	874.7 (812.9;936.3)	923.1 (856.1;989.3)	1599.2 (1461.9;1744.8)	505.7 (461;552.2)	-45.2 (-51.2;-39.3)
Alagoas	9872.6 (9062.5;10730.1)	1226.4 (1127.7;1322.2)	12763.3 (11791.5;13850.8)	733.8 (676.7;796.4)	-40.2 (-46.2;-33.1)
Amapá	439.7 (406.5;470)	729.9 (668.2;779.6)	1319.2 (1165.7;1460.5)	485.9 (429.7;538.1)	-33.4 (-40.7;-24.9)
Amazonas	4241.5 (3841.4;4559.2)	918 (830.8;989.4)	7271.3 (6627.5;7883.3)	503.5 (459.3;545.9)	-45.1 (-50.5;-38.7)
Bahia	39495.1 (36010.5;43294.5)	993.4 (903.6;1090.2)	44388.6 (40962.7;47759)	515.9 (475.7;555.3)	-48.1 (-53.8;-42.1)
Brasil	563999.7 (545387.7;584682.8)	1070.2 (1035.3;1106.6)	580172.8 (555092.6;606176.7)	465.2 (445;485.9)	-56.5 (-58.7;-54.5)
Ceará	18233.6 (16299.5;20228.9)	772.8 (688.7;861.4)	24338.9 (22304.9;26159.7)	453.1 (415.4;487.5)	-41.4 (-48.8;-33.2)
Distrito Federal	4240.9 (3979.9;4508.9)	1005.6 (949;1069.6)	4945.6 (4453.2;5505.7)	345.3 (314.1;384.1)	-65.7 (-69.4;-61.7)
Espírito Santo	11142.1 (10603;11788.2)	1321.2 (1257.8;1394.8)	10272.9 (9453.4;11232.8)	445.3 (409.9;487.5)	-66.3 (-69.3;-63.3)
Goiás	12855.7 (12049.1;13698)	1147.9 (1079.5;1224.7)	15021.7 (13681.7;16488.2)	415.3 (379.8;455)	-63.8 (-67.3;-60.4)
Maranhão	12042.9 (9978.3;14209.6)	765.5 (599.9;923.2)	19249 (17310;21828.7)	557.1 (499.7;633.4)	-27.2 (-37.8;-11.3)
Mato Grosso	3853.5 (3437.3;4288.6)	817 (728.9;905.6)	6957.4 (6329.9;7833.2)	433.6 (394.8;486.6)	-46.9 (-54.1;-38.6)
Mato Grosso do Sul	5943.8 (5561.7;6313.3)	1148.3 (1080.5;1215)	7653.1 (7025.6;8338.4)	513.4 (471.2;560.2)	-55.3 (-59.8;-50.7)
Minas Gerais	64366 (60674.3;68220.4)	1099.1 (1037.2;1163.7)	60695 (56080.7;65783.6)	445.7 (411.9;483)	-59.4 (-63;-55.4)
Pará	12175.4 (11139.9;13184)	978.4 (892.1;1059.1)	18001 (16375.4;19576.3)	509.9 (464;555.1)	-47.9 (-54;-41.2)
Paraíba	11111.2 (10082.5;12219.4)	868.2 (787.5;953.5)	13504.5 (12040;15189.3)	535.8 (476.9;602.6)	-38.3 (-46.7;-28.4)
Paraná	35261.9 (33355.6;37203.2)	1301.5 (1228.2;1375.8)	31095.5 (28671.9;33664.6)	447.4 (412.9;484.6)	-65.6 (-68.7;-62.4)
Pernambuco	35167.1 (32910.5;37242.7)	1332.3 (1249;1406.7)	33631.6 (31062.3;36327.8)	599.2 (553.1;646.5)	-55 (-59;-50.7)
Piauí	7420.7 (6473.6;8346.5)	888.3 (775.7;998.5)	10247.3 (9366.6;11531.5)	529 (483;595.7)	-40.4 (-47.8;-31.4)
Rio de Janeiro	88936.8 (84337.9;93896)	1501.8 (1423.9;1582.9)	68013.5 (62977;73431)	555.9 (515.3;599.1)	-63 (-66.3;-59.6)
Rio Grande do Norte	5639.4 (5056.1;6175)	626.4 (561.9;686.3)	7840.9 (7108.5;8599.3)	383.7 (347.4;421.2)	-38.7 (-46.2;-29.8)
Rio Grande do Sul	40495.5 (37779.2;42885.8)	1036.9 (967.6;1098.7)	39133.7 (35903.4;42395.5)	472.7 (432.7;511.1)	-54.4 (-58.6;-49.5)
Rondônia	2628.7 (2371.6;2911.8)	1227.9 (1117.7;1354.8)	3779.6 (3245.4;4369.3)	501 (433.1;579.1)	-59.2 (-65.6;-51.6)
Roraima	328.6 (286.2;373.3)	992.7 (870.9;1116.1)	736.9 (617.6;867)	430 (361.4;504.1)	-56.7 (-64.8;-46.4)
Santa Catarina	16220.7 (15245.9;17192.1)	1118.6 (1052.1;1188.3)	15768.8 (14405.3;17123)	377.2 (345.2;409.4)	-66.3 (-69.7;-62.9)
São Paulo	113907.8 (107421.3;120928.2)	942.3 (886.5;1000.5)	111325 (103027.9;120702.2)	383.3 (354.6;414.6)	-59.3 (-63;-55.4)
Sergipe	4704.6 (4381.8;5022.3)	963.7 (897.4;1029.7)	6663.5 (6108.6;7225.7)	552.1 (505.7;600.2)	-42.7 (-48.5;-35.7)
Tocantins	2399.1 (1989.3;2787)	1052.1 (902.5;1202.8)	3955.6 (3568.2;4425.3)	560.4 (506.6;627.1)	-46.7 (-54.7;-36.9)
***B.2.3.3- Hemorragia Subaracnóidea***					
Acre	448.2 (407.7;497.6)	330.8 (302.6;363.3)	708.2 (638.6;792.2)	194.2 (174.9;216.9)	-41.3 (-48.2;-34)
Alagoas	4300.1 (3589.8;5036.8)	410.6 (351;468.2)	4267.3 (3820.8;4772.1)	234.1 (208.7;261.8)	-43 (-52.3;-29.4)
Amapá	180.3 (163.7;201.9)	223.8 (202.9;252.3)	571.6 (511;643.2)	176.7 (158;198.7)	-21.1 (-31.2;-9.9)
Amazonas	1591.2 (1418;1789.1)	242.6 (217.8;270.9)	2930.3 (2642.6;3273.2)	174.4 (156.1;192.6)	-28.1 (-37.5;-18.8)
Bahia	14897.5 (13287.5;16507.2)	316.1 (283.5;351.9)	17771.1 (16139.9;19611.7)	207.1 (188.6;228.5)	-34.5 (-42.6;-24.3)
Brasil	211672.4 (199208.9;221899.8)	345.1 (324.7;363.2)	246672.1 (231937.6;260803.4)	199.2 (187.4;210.1)	-42.3 (-46.1;-39)
Ceará	8643.3 (6852.4;10486.6)	299.4 (244.9;357.4)	9666.5 (8677.7;10600.5)	182.6 (164.3;200.1)	-39 (-50.4;-26.6)
Distrito Federal	2002 (1803;2168.1)	347.8 (316.2;375.5)	2613.4 (2319;2911.4)	159.4 (141;177.5)	-54.2 (-59.3;-47.8)
Espírito Santo	3997.2 (3523.8;4296.8)	396 (345.6;425)	4463.2 (4009.2;4908.2)	192.6 (173.2;211.2)	-51.4 (-56.9;-43.7)
Goiás	4756.8 (4346.9;5221.2)	327.6 (300.5;359.9)	6794.6 (6134.7;7577.9)	177.8 (160.7;197.9)	-45.7 (-51.6;-38.3)
Maranhão	8586.9 (6391.4;11138.3)	388.5 (299.2;491.9)	8660.9 (7731.3;9594.6)	237 (211.2;263.3)	-39 (-51.4;-23.6)
Mato Grosso	1976.9 (1748.4;2227.8)	303.1 (269.1;341.7)	3274 (2926.5;3658.6)	184.1 (165.4;204.9)	-39.2 (-47.7;-29.8)
Mato Grosso do Sul	2214 (1954.7;2430)	350.7 (315.4;383.4)	3228.6 (2883.8;3619.9)	208.3 (186.4;233)	-40.6 (-48.1;-31.7)
Minas Gerais	23395.6 (21454;25318.2)	351.9 (322.6;381.9)	26003.9 (23613.8;28559.9)	196.4 (178.8;214.9)	-44.2 (-50;-37.7)
Pará	4440.4 (3942.4;4957.1)	268.2 (240.2;299.8)	7488.9 (6775.2;8285.9)	190.5 (171.6;211.1)	-29 (-37.1;-19.3)
Paraíba	5050.1 (4155.5;5968.1)	359.5 (295.4;422.2)	5151 (4436.2;5838.5)	211.9 (182.7;240.2)	-41.1 (-53;-27.7)
Paraná	12285.8 (11347.4;13264.7)	374.4 (343.4;405.7)	13900 (12510.5;15373.5)	199.1 (179.6;219.1)	-46.8 (-53;-40.1)
Pernambuco	9495.7 (8636;10672.6)	318.4 (290.2;361.1)	12353.7 (11186.9;13775.6)	219.1 (198.7;243.7)	-31.2 (-39.6;-22.6)
Piauí	3535.2 (2860;4197.6)	339.4 (278.5;403.6)	3908.2 (3521;4310.2)	203.7 (183.8;224.5)	-40 (-49.9;-27.8)
Rio de Janeiro	28289.5 (25607.4;30525.9)	444.3 (403.8;479.4)	25692.3 (23122.1;28299.6)	221.6 (200.2;244.4)	-50.1 (-55.6;-43.3)
Rio Grande do Norte	2208.9 (1905.2;2660.8)	210.9 (182.9;259.7)	3065.8 (2734.3;3771.2)	151.5 (135.3;186)	-28.2 (-39.4;-16.6)
Rio Grande do Sul	12260.7 (11247.3;14498.1)	286.6 (262.9;339.3)	13863.3 (12280.1;15559.9)	180.6 (160.3;202.5)	-37 (-44.8;-29)
Rondônia	1120.6 (1002.6;1250.6)	353.1 (315.9;395.2)	1701.2 (1439.6;1988.5)	200 (169.9;234.3)	-43.4 (-53.1;-32.1)
Roraima	151.2 (130.8;181.3)	282 (242.8;345.6)	330.2 (273;406.4)	153 (127.5;188.8)	-45.8 (-56.1;-32.4)
Santa Catarina	5568.2 (5137.7;6024.6)	316.5 (290.8;342.4)	6725.8 (6071.4;7486.2)	158.6 (143.3;176.2)	-49.9 (-55.5;-43.4)
São Paulo	47460.6 (43883.2;51172.3)	337.9 (312.7;365.3)	57392.7 (51539.2;62558)	200.5 (180.5;218)	-40.7 (-47.3;-33.9)
Sergipe	1689.1 (1486.9;1900.9)	287.8 (256.9;325.8)	2382.3 (2149.2;2638.5)	188.8 (170.3;210.1)	-34.4 (-43.9;-25.1)
Tocantins	1126.4 (839.2;1386.1)	360 (280.2;438.7)	1763.1 (1534.2;1962.8)	230.6 (201.3;256.2)	-35.9 (-48.5;-18.3)

* Fonte: Estudo Global Burden of Disease 2017, Institute for Health Metrics and Evaluation. ^109^*

**Tabela 2-9:** – Taxas de DALYs padronizadas por idade (por 100 mil) por AVC e subtipos de AVC, para homens, no Brasil, 1990 e 2017, e variação percentual das taxas

Causa de morte e grupo etário	1990	2017	Variação percentual (II 95%)
***B.2.3- AVC***			
15-49 anos	986,5 (948,5;1024,5)	432,8 (415,2;452,5)	-56,1 (-58,4;-53,6)
50-69 anos	7867,2 (7612,7;8119,2)	3538 (3397,9;3689,8)	-55 (-56,9;-53,1)
5-14 anos	83,1 (69,7;91,1)	35,7 (31,8;39,7)	-57,1 (-62,4;-50,4)
70+ anos	17208,4 (16711,1;17728,9)	9407,6 (9006,8;9773,9)	-45,3 (-47,4;-43,3)
Padronizadas por idade	2901,8 (2824,3;2983,3)	1334,5 (1289,5;1381,5)	-54 (-55,4;-52,5)
Todas as idades	1814,8 (1761,9;1869,6)	1319,5 (1275,2;1367,3)	-27,3 (-29,6;-24,7)
Abaixo de 5	410,2 (325,6;536,1)	70,8 (58,7;84,7)	-82,8 (-87,9;-75,9)
***B.2.3.1- AVC Isquêmico***			
15-49 anos	146,5 (137,3;158,8)	66,3 (60;73,2)	-54,7 (-59,7;-50,7)
50-69 anos	2283,9 (2165,8;2401,8)	952,4 (887,3;1020)	-58,3 (-61,1;-55,6)
5-14 anos	10,9 (8,9;13,6)	4,2 (3,3;5,5)	-61,2 (-66,6;-55,8)
70+ anos	8926,8 (8590,6;9283,7)	4660,5 (4394,5;4925)	-47,8 (-50,4;-45,3)
Padronizadas por idade	1011 (973,7;1050,4)	463,4 (436,9;490,2)	-54,2 (-56,5;-52,2)
Todas as idades	541,4 (520,1;565,3)	429,6 (404,9;455,5)	-20,6 (-25;-17)
Abaixo de 5	24,6 (16,8;34,3)	3 (2,2;3,9)	-87,6 (-93,3;-78,5)
***B.2.3.2- Hemorragia Intracerebral***			
15-49 anos	644,8 (614,9;701,4)	257,6 (241,6;271,2)	-60,1 (-64,4;-57,1)
50-69 anos	4886,7 (4702,7;5077,9)	2143 (2055,1;2228,8)	-56,1 (-58,2;-54)
5-14 anos	25 (20,4;28,9)	10 (8,2;11,8)	-60,1 (-69,4;-49,4)
70+ anos	7674,2 (7361,5;7956,2)	4238,2 (4048,2;4415,7)	-44,8 (-47,4;-41,9)
Padronizadas por idade	1601 (1552,2;1665,3)	709,9 (684,6;733,7)	-55,7 (-57,5;-53,9)
Todas as idades	1042,8 (1008,5;1093)	721,4 (695,9;745,7)	-30,8 (-34,2;-27,8)
Abaixo de 5	108,9 (61,6;169)	12,1 (8,8;16)	-88,9 (-94,1;-76,9)
***B.2.3.3- Hemorragia Subaracnóidea***			
15-49 anos	195,1 (150,6;208,4)	108,9 (102;128,8)	-44,2 (-49,5;-22,9)
50-69 anos	696,6 (566,1;745,5)	442,6 (414,3;479,7)	-36,5 (-41,7;-23,2)
5-14 anos	47,2 (39,3;52,5)	21,4 (18,9;24,2)	-54,6 (-60,4;-44,4)
70+ anos	607,4 (558;657,4)	508,9 (436,7;551,9)	-16,2 (-24,4;-7,6)
Padronizadas por idade	289,9 (237,9;307,2)	161,2 (152,9;175,4)	-44,4 (-48,2;-31,2)
Todas as idades	230,7 (186,5;246,2)	168,5 (160;184,4)	-26,9 (-32,5;-7,7)
Abaixo de 5	276,7 (203,1;344,3)	55,7 (46,8;65,9)	-79,9 (-85,1;-70,4)

* Fonte: Estudo Global Burden of Disease 2017, Institute for Health Metrics and Evaluation. ^109^*

**Tabela 2-10:** – Taxas de DALYs padronizadas por idade (por 100 mil) por AVC e subtipos de AVC, para mulheres, no Brasil, 1990 e 2017, e variação percentual das taxas.

Causa de morte e grupo etário	1990	2017	Variação percentual (II 95%)
***B.2.3- AVC***			
15-49 anos	817,7 (790,3;848,7)	421,7 (400,5;440)	-48,4 (-51,1;-45,8)
50-69 anos	5185 (4992,7;5389,2)	2392,8 (2276,5;2511)	-53,9 (-56;-51,8)
5-14 anos	69,9 (60,4;76,3)	32,7 (29,4;36,3)	-53,2 (-57,8;-47,5)
70+ anos	13838,3 (13364,1;14288,7)	7055,4 (6735,7;7403,1)	-49 (-51;-46,9)
Padronizadas por idade	2162,9 (2095,1;2225,5)	990,7 (947,7;1034,7)	-54,2 (-55,9;-52,7)
Todas as idades	1466,3 (1419,5;1509,1)	1134,6 (1085,5;1185,3)	-22,6 (-25,6;-20)
Abaixo de 5	337,3 (257,9;439)	49,3 (41,5;60,4)	-85,4 (-89,9;-78,1)
***B.2.3.1- AVC Isquêmico***			
15-49 anos	104,9 (97,4;113,8)	55,1 (48,1;62,3)	-47,5 (-53,4;-42,3)
50-69 anos	1382,3 (1287,6;1474,6)	571,4 (514,2;629,2)	-58,7 (-61,7;-55,6)
5-14 anos	7,5 (6,2;9,1)	4,3 (3,2;5,5)	-43 (-49,6;-36,6)
70+ anos	7472,4 (7131,5;7787,8)	3643,1 (3416,1;3892)	-51,2 (-53,8;-48,8)
Padronizadas por idade	747,7 (713,7;780,1)	326,3 (302,2;351,8)	-56,4 (-58,8;-54,1)
Todas as idades	439,5 (418,8;460,3)	371,1 (343,5;400,1)	-15,5 (-20,3;-11,3)
Abaixo de 5	18,4 (13;29,6)	2,4 (1,9;3)	-87 (-93,1;-79,5)
***B.2.3.2- Hemorragia Intracerebral***			
15-49 anos	460,3 (440,4;483,1)	207 (194,5;220,2)	-55 (-58,2;-51,4)
50-69 anos	2968,9 (2849,9;3089,3)	1272,5 (1215,6;1335,2)	-57,1 (-59,8;-54,7)
5-14 anos	20,3 (17,3;22,7)	9,4 (8,1;10,8)	-53,5 (-60,8;-45,8)
70+ anos	5659,7 (5417,6;5890,7)	2879,7 (2743,7;3027,5)	-49,1 (-52;-46,1)
Padronizadas por idade	1070,2 (1035,3;1106,6)	465,2 (445;485,9)	-56,5 (-58,7;-54,5)
Todas as idades	746,6 (722;774)	535,7 (512,6;559,7)	-28,2 (-31,8;-24,9)
Abaixo de 5	69,4 (45,1;118,1)	7,9 (5,9;10,3)	-88,6 (-94,6;-79,9)
***B.2.3.3- Hemorragia Subaracnóidea***			
15-49 anos	252,5 (238,8;265,9)	159,6 (149,6;169,8)	-36,8 (-41,6;-31,8)
50-69 anos	833,8 (780,9;891,9)	548,9 (513;588,7)	-34,2 (-39,3;-29,1)
5-14 anos	42,1 (35,8;46,5)	19 (17;21,3)	-54,8 (-59,7;-48,2)
70+ anos	706,2 (661,2;794,7)	532,6 (494,2;573,3)	-24,6 (-32,3;-18,3)
Padronizadas por idade	345,1 (324,7;363,2)	199,2 (187,4;210,1)	-42,3 (-46,1;-39)
Todas as idades	280,2 (263,7;293,7)	227,8 (214,2;240,8)	-18,7 (-24,1;-13,6)
Abaixo de 5	249,5 (190,9;317,3)	39 (32,9;48,1)	-84,4 (-89,1;-77,2)

* Fonte: Estudo Global Burden of Disease 2017, Institute for Health Metrics and Evaluation. ^109^*

## 3. DOENÇA ARTERIAL CORONARIANA AGUDA E CRÔNICA

### CID-9-CM 410 a 414; CID-10 I10 a I25

Ver Tabelas 3-1 até 3-3 e Figuras 3-1 até 3-6

**Table:** 

Abreviaturas usadas no Capítulo 3	
ACCEPT/SBC	Registro Brasileiro da Prática Clínica nas Síndromes Coronarianas Agudas da Sociedade Brasileira de Cardiologia
BRACE	*Brazilian Registry on Acute Coronary Syndrome*
BYPASS	Registro Brasileiro de Pacientes Adultos Submetidos a Cirurgia Cardiovascular
CRVM	Cirurgia de Revascularização do Miocárdio
DAC	Doença Arterial Coronariana
DALYs	Anos de vida perdidos ajustados por incapacidade (do inglês, *Disability-Adjusted Life-Year* )
DATASUS	Departamento de Informática do Sistema Único de Saúde
DCV	Doença Cardiovascular
DIC	Doença Isquêmica do Coração
GBD	*Global Burden of Disease*
IAM	Infarto Agudo do Miocárdio
IAMCSST	Infarto Agudo do Miocárdio com Supradesnível do Segmento ST
IAMSSST	Infarto Agudo do Miocárdio sem Supradesnível do Segmento ST
IC	Intervalo de Confiança
ICP	Intervenção Coronária Percutânea
II	Intervalo de Incerteza
IM	Infarto do Miocárdio
MASS	*Medicine, Angioplasty, or Surgery Study*
OMS	Organização Mundial da Saúde
PIB	Produto Interno Bruto
PNS	Pesquisa Nacional de Saúde
RBSCA	Registro Brasileiro de Síndrome Coronariana Aguda
SCA	Síndrome Coronariana Aguda
SUS	Sistema Único de Saúde
YLLs	Anos potenciais de vida perdidos (do inglês, *Years of Life Lost* )

### Panorama e Prevalência

- A DAC, também conhecida como DIC, compreende um espectro de condições clínicas sintomáticas e assintomáticas tipicamente relacionadas à redução do fluxo sanguíneo para o músculo cardíaco. A causa mais comum é a doença aterosclerótica das artérias coronárias, uma condição crônica de apresentação variável, que progride desde uma longa fase assintomática até angina estável, IM e angina instável. A DAC é uma causa comum de insuficiência cardíaca, com fração de ejeção ventricular esquerda reduzida ou preservada, arritmias ventriculares e parada cardíaca súbita.
- A DAC foi a principal causa de morte no Brasil na última década, para homens e mulheres. Devido ao seu amplo espectro de apresentação clínica, suas prevalência, incidência e mortalidade relatadas variam muito, dependendo da população e do contexto da atenção à saúde estudados.

#### Doença Arterial Coronariana

- De acordo com dados do GBD 2017, a prevalência total de DAC foi 1,75% em brasileiros >20 anos. Homens apresentaram maior prevalência em comparação a mulheres, 2,33% e 1,19%, respectivamente. Para adultos de 15-49 anos, a prevalência estimada de DAC foi 0,53%; para aqueles de 50-69 anos, 4,34%; e para aqueles >70 anos, 10,99% (Figura 3-1).
- A prevalência geral padronizada por idade de DAC foi 1,63% (1.564 por 100 mil habitantes), sendo 2,35% (2.229 por 100 mil habitantes) para homens e 1,05% (1.008 por 100 mil habitantes) para mulheres. Isso significa que pelo menos ~3,3 milhões de pessoas viviam com DAC nas unidades federativas brasileiras em 2017.
- Houve uma diferença, entre as regiões brasileiras, na prevalência padronizada por idade de DAC, que foi maior nas regiões Sudeste e Sul (estado de São Paulo, 1.617 por 100 mil habitantes; Rio Grande do Sul, 1.642 por 100 mil habitantes) e menor nas regiões Norte e Centro-Oeste (Amazonas, 1.407 por 100 mil habitantes; Distrito Federal, 1.404 por 100 mil habitantes). A região Nordeste ocupou uma posição intermediária, mas com tendência a aumento da prevalência de DAC nas últimas décadas (Pernambuco, 1.523 por 100 mil habitantes) ( Tabela 3-1 ).
- No período 1990-2017, a prevalência de DAC aumentou nos dois sexos (de 1,08% para 1,75%), de maneira mais proeminente em homens do que em mulheres. Tal aumento deveu-se provavelmente ao envelhecimento da população, pois as taxas de prevalência padronizadas por idade permaneceram estáveis nas 2 últimas décadas (variação anual, -0,25%) para ambos os sexos (Figura 3-2).

#### Angina Estável

- Inquéritos populacionais regionais conduzidos antes de 2000, aplicando o questionário de Rose de angina em 2 cidades (Ribeirão Preto, em São Paulo, e Pelotas, no Rio Grande do Sul), identificaram prevalência de angina de 12,3% e 8,2%, respectivamente, em adultos com idade ≥ 40 anos. ^110 , 111^
- O Inquérito Nacional de Saúde, conduzido em 2003 entre 5.000 indivíduos, mostrou prevalência de angina autorrelatada de 6,7% em brasileiros com idade ≥18 anos e de 13% naqueles com idade >50 anos. Apenas 72,8% dos indivíduos relataram adesão a terapia medicamentosa nas 2 semanas anteriores. ^112^
- De acordo com a PNS 2013, um inquérito epidemiológico de base domiciliar e com representatividade nacional, a prevalência geral de angina classe I foi 7,6% (IC 95%, 7,2% - 8,0%), e de angina classe II, 4,2% (IC 95%, 3,9% - 4,5%). ^113^
- A angina *pectoris* autorreferida foi mais prevalente em mulheres do que em homens em todos os estudos descritos.
- É importante ressaltar as maiores taxas de prevalência observadas nos inquéritos prospectivos em comparação às estatísticas nacionais. Avaliações autorreferidas de angina são muito sensíveis, mas não específicas para DAC, pois não requerem exames confirmatórios nem relatórios de saúde. Além disso, considerando-se a natureza assintomática da DAC, a sua verdadeira epidemiologia pode estar sub-representada nas estatísticas nacionais.

### Incidência

- O GBD estimou uma incidência de 84 eventos de DAC por 100 mil habitantes em 2017 no Brasil. A incidência padronizada por idade foi 79 por 100 mil habitantes e os números ajustados por idade foram maiores para homens do que para mulheres, 104 e 58 por 100 mil habitantes, respectivamente. Houve aumento exponencial na incidência por grupo etário, de 19 por 100 mil habitantes no grupo de 15-49 anos para 198 por 100 mil habitantes no grupo de 59-69 anos, e 744 por 100 mil habitantes no grupo ≥ 70 anos. A variação temporal de 1990 a 2017 foi pequena, 1,03% bruta e -1,29% padronizada por idade anualmente.
- De acordo com dados do DATASUS, em 2018, houve 142.982 novos casos de IAM e SCA.
- Em revisão sistemática de dados de saúde pública em 2009, as taxas de SCA e IM por 100 mil habitantes foram 38 e 29,8, respectivamente. ^114^

### Mortalidade

 • De acordo com as estimativas do GBD 2017, houve 175.791 óbitos atribuídos a DAC, correspondendo a 13% do total de óbitos no Brasil ( Figura 3-3 ). 

**Figura 3-3:**
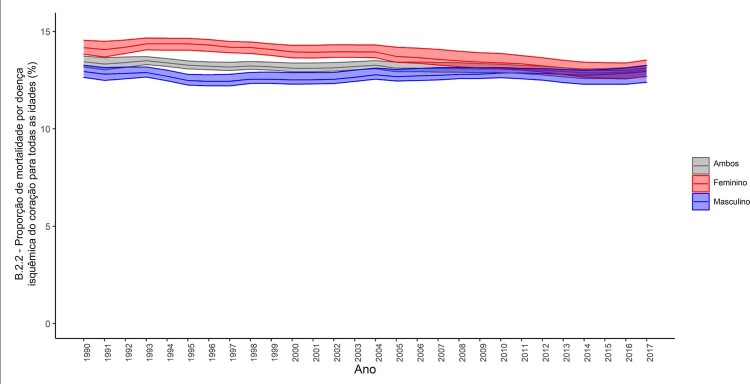
- Proporção de mortalidade por doença isquêmica do coração no Brasil para ambos os sexos (1990-2017).

Dados derivados do Estudo Global Burden of Disease 2017 (GBD 2017). ^151^

- A taxa de mortalidade bruta atribuída a DAC foi 83 por 100 mil habitantes em 2017 (GBD 2017), sendo maior para homens do que para mulheres (95 e 72, respectivamente). Como esperado, as taxas foram mais altas para os grupos etários avançados: 161 por 100 mil habitantes para o grupo de 50-69 anos e 837 por 100 mil habitantes para o grupo ≥ 70 anos.
- Segundo o GBD 2017, a taxa de mortalidade padronizada por idade por DIC foi 80 (IC 95%, 78 - 82) por 100 mil habitantes, representando 13% das causas de morte no Brasil. A DIC foi a principal causa de morte em todas as unidades federativas brasileiras em 2017.
- As variações regionais nas taxas de mortalidade são significativas. A menor taxa de óbito foi observada no Amazonas (59 por 100 mil habitantes), enquanto a maior, em Pernambuco (102 por 100 mil habitantes). Em todas as regiões brasileiras, a DIC foi a principal causa de morte nas últimas 3 décadas. ^115^
- De acordo com dados submetidos à OMS e ao Banco Mundial, em 2015, houve 111.849 óbitos por DAC no Brasil, cerca de 50 por 100 mil indivíduos, fazendo da DAC a principal causa de mortalidade de 2010 a 2015. ^116^
- De acordo com dados do Ministério da Saúde de 2009, de 962.931 mortes acima dos 30 anos, 95.449 foram causadas por DAC, enquanto 193.309, por aterosclerose. ^117^
- Dados do GBD 2017 mostraram uma queda na mortalidade por DIC de 1990 a 2017 ( Figura 3-4 ), com variações anuais não ajustadas de -0,15%, correspondendo a uma variação acumulada de -53% (-54% a -51%) no período. Essa queda foi observada em todas as unidades federativas, sendo, no entanto, menos expressiva na região Nordeste (Ceará, -22%) do que na Sudeste (Minas Gerais, -63%). A taxa padronizada por idade diminuiu de 169 por 100 mil habitantes em 1990 para 80 por 100 mil habitantes em 2017. Essa tendência foi semelhante nos dois sexos e em todos os grupos etários ( Tabela 3-2 ).
  **Figura 3-4:**
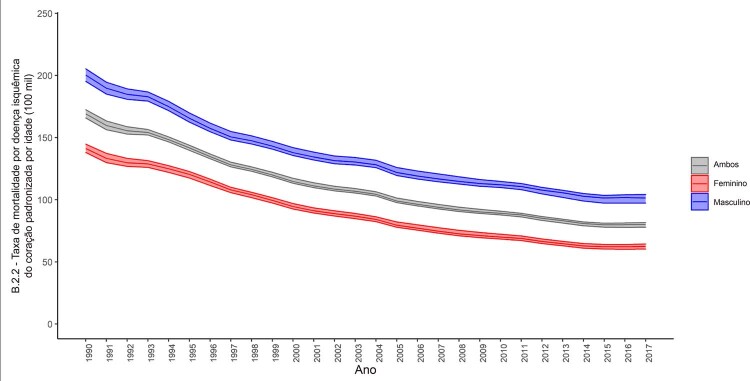
- Taxas de mortalidade por doença isquêmica do coração padronizadas por idade no Brasil para ambos os sexos (1990-2017).

Dados derivados do Estudo Global Burden of Disease 2017 (GBD 2017). ^151^
- A análise temporal da mortalidade por DCV entre 1981 e 2001 mostrou que o coeficiente de mortalidade por DAC permaneceu estável para mulheres nas regiões Norte e Centro-Oeste, enquanto diminuiu nas regiões Sul e Sudeste e aumentou na região Nordeste. ^118^ Para homens, houve tendência a menor número de eventos nas regiões Sul e Sudeste. ^118^
- Análise conduzida a partir de dados do DATASUS, de 1990 a 2009, mostrou uma redução nas mortes por DAC no Brasil. ^117^ A taxa caiu de 195 por 100 mil habitantes para 149 por 100 mil habitantes para homens e de 120 por 100 mil habitantes para 84 por 100 mil habitantes para mulheres ( Figura 3-5 ).
  **Figura 3-5:**
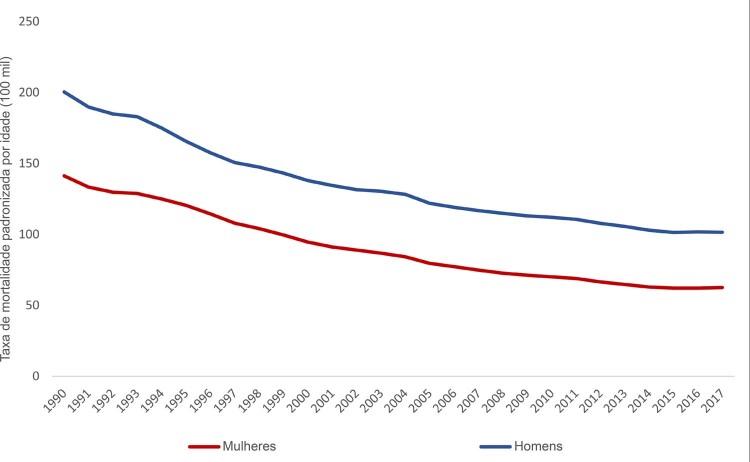
- Taxa de mortalidade por doença isquêmica do coração por 100 mil habitantes para homens e mulheres de 1990 a 2017.

Dados derivados do DATASUS. ^152^

**Figura 3-6:**
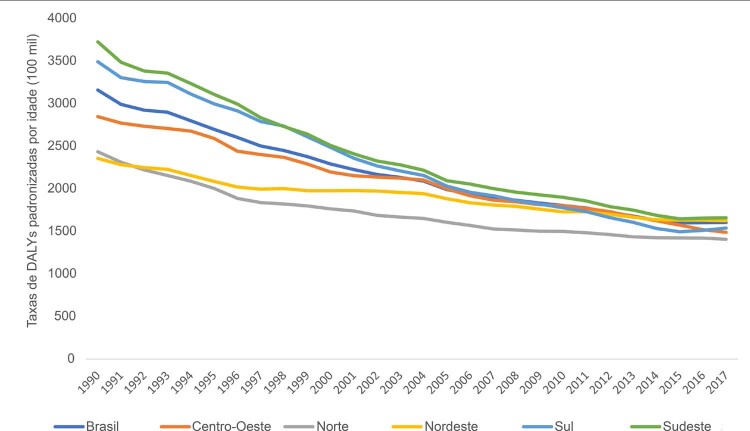
- Taxas de DALYs por doença isquêmica do coração padronizadas por idade no Brasil e em cada região brasileira.

Dados derivados do Estudo Global Burden of Disease 2017 (GBD 2017). ^151^
- A proporção de óbitos por DAC permaneceu estável nas últimas décadas, com relatos variando de 26% a 32%, de acordo com o ano. Um estudo ecológico realizado em Porto Alegre, incluindo indivíduos com 45-64 anos, mostrou que a DCV foi responsável por 28,5% de todos os óbitos em 2009. Desses, 40% estavam relacionados a DAC, cuja proporção foi maior entre indivíduos com nível socioeconômico mais baixo (42,7%) do que entre aqueles com nível socioeconômico mais alto (26,3%). ^119^
- Em um estudo ecológico nacional incluindo indivíduos com 35-64 anos, de 1999 a 2001, a taxa de morte relacionada a DAC foi 84 ± 30 por 100 mil habitantes. Nesse estudo, a incidência de eventos relacionou-se diretamente à taxa de pobreza e ao menor nível educacional. ^120^ É importante notar que houve grande variação nos resultados entre as 98 cidades participantes, provavelmente em razão da qualidade dos dados.

####  Mortalidade Relacionada à Síndrome Coronariana Aguda

- De acordo com o DATASUS, houve 142.982 hospitalizações por IAM em 2018, com mortalidade hospitalar de 11%.
- Vários registros brasileiros de SCA relataram os desfechos de indivíduos admitidos com SCA. Em geral, a mortalidade nos registros é menor do que a relatada nos Sistemas Nacionais de Informação em Saúde.
- Entre 2003 e 2008, o Registro RBSCA arrolou 2.693 pacientes, 45% dos quais com IAM. A mortalidade hospitalar para aqueles com angina instável foi 3,1%, enquanto para aqueles com IM, foi 7,7%, levando a uma mortalidade geral de 5,5%. ^121^
- Resultados do BRACE, um registro epidemiológico observacional transversal de pacientes com SCA, publicados em 2012 mostraram mortalidade hospitalar geral de 5,2% entre 1.150 pacientes de 72 hospitais incluídos no registro. ^122 , 123^
- Em estudo retrospectivo multicêntrico de 3.745 pacientes admitidos com SCA entre 2010 e 2015 em hospital de São Paulo, a mortalidade hospitalar por todas as causas foi 3,3%, tendo 454 (12,2%) pacientes experienciado pelo menos um evento adverso maior (reinfarto, choque, sangramento, acidente vascular cerebral ou morte). ^124^
- Em outro registro de base hospitalar, o Estudo ERICO, uma coorte de indivíduos com SCA admitidos em hospital comunitário regional do estado de São Paulo, as taxas de mortalidade em 30 dias e 1 ano foram 4,4% e 12%, respectivamente. ^125^
- O Registro ACCEPT/SBC, conduzido de 2010 a 2011 em 47 hospitais, arrolou 2.485 pacientes com SCA, 35% com IAMSSST e 33% com IAMCSST. A mortalidade por todas as causas em 30 dias foi 1,8% para angina instável, 3,0% para IAMSSST, e 3,4% para IAMCSST. ^126^
- Um estudo observacional longitudinal realizado de 2011 a 2014 em um hospital de alta complexidade, em Belo Horizonte, incluindo 1.129 pacientes com IAMCSST e IAMSSST, reportou mortalidade hospitalar de 8,7%. Dos pacientes com IAMCSST, 56% receberam terapia de reperfusão e 67% foram tratados de acordo com as práticas recomendadas nas diretrizes. ^127^
- Em estudo do Projeto Minas Telecardio 2, conduzido em 2013 e 2014 em 6 unidades de emergência da cidade de Montes Claros, Minas Gerais, entre 593 pacientes com SCA, a mortalidade hospitalar foi 9,4%, variando de 4,9% para angina instável a 17% para os casos de IAMCSST. ^128^ Em um registro de atendimento ao IAMCSST na cidade de Salvador (RESISST), conectado a uma rede regional de atendimento ao IAM, de janeiro de 2011 a junho de 2013, apenas 41% dos pacientes receberam terapia de reperfusão, sendo a taxa de mortalidade em 30 dias de 15,3%. ^129^
- Dados estaduais de Sergipe coletados de 2014 a 2017 identificaram 707 casos de IAMCSST, com mortalidade hospitalar de 10,9%. A taxa de mortalidade de indivíduos admitidos em hospitais públicos foi significativamente maior quando comparada à daqueles admitidos em hospitais privados (11,9% *vs.* 5,9%, respectivamente), como descrito em estudos prévios. ^129 , 130^

#####  A. Mortalidade relacionada a intervenções coronárias percutâneas

- De acordo com o DATASUS, em 2018, 10.811 angioplastias primárias foram realizadas por IAM, com mortalidade hospitalar de 6,3% e média de permanência hospitalar de 5,1 dias. Quando todos os outros procedimentos coronários foram considerados, 78.575 angioplastias coronárias foram identificadas, com mortalidade hospitalar de 2,96% e média de permanência hospitalar de 4,5 dias.
- De acordo com o DATASUS, em um conjunto de dados de 3.874 indivíduos, a mortalidade hospitalar relacionada a ICP foi 2,33% entre 2005 e 2008. ^121^ Essa taxa foi menor na região Sudeste (2,03%) e maior na região Norte (3,64%) (p < 0,001). O volume de procedimentos não foi associado com desfecho naquelas análises. ^121^
- Em um estudo de coorte realizado de 2009 a 2013, avaliando 4.806 pacientes submetidos a ICP (Registro ICP-BR) em 8 centros médicos terciários de referência, considerando todas as condições clínicas (69% com IM recente), a mortalidade hospitalar foi 2,6%. ^131^
- No Registro ACCEPT/SBC, que arrolou 2.485 pacientes com SCA de 2010 a 2011, a maioria em centros terciários, mais de 90% deles foram submetidos a cateterismo cardíaco e cirurgia cardiovascular no local. Naqueles pacientes, a taxa de revascularização foi 39% para angina instável, 54% para IAMSSST e 78% para IAMCSST. No grupo de IAMCSST, a terapia de reperfusão foi usada em 88% dos pacientes, tendo a maioria (73%) sido submetida a angioplastia primária. O atraso no tratamento foi, em média, de 125 ± 90 minutos. ^126^
- Outro registro de ICP, que incluiu 1.249 pacientes consecutivos em 2009, encontrou mortalidade total de 2,3%, variando de 0,2% para angina estável a 6,1% para IAMCSST. ^132^
- Em outra série de ICP em hospitais públicos de 2005 a 2008, 166.514 procedimentos foram realizados em 180 hospitais. A mortalidade hospitalar média foi de 2,33%, variando de 0% a 11,35%. Essa taxa foi mais baixa na região Sudeste (2,03%) e mais alta na região Norte (3,64%). A taxa de mortalidade hospitalar foi 2,33% nos hospitais de alto volume, responsáveis por 101.218 (60,8%) ICP, tendo sido 2,29% nos hospitais de médio volume e 2,52% nos hospitais de baixo volume. A mortalidade foi maior entre mulheres e acima dos 65 anos. ^133^
- A maioria dos relatos é proveniente de instituições públicas, sendo os dados dos hospitais privados limitados. Uma análise de 440 procedimentos realizados entre 2013 e 2014 em um hospital público e outro privado na cidade do Rio de Janeiro mostrou baixa mortalidade (0,5%), com taxas semelhantes nas duas instituições. ^134^
- Dados sobre as taxas de sobrevida de longo prazo para pacientes submetidos a ICP são escassos. Em uma análise de procedimentos realizados no estado do Rio de Janeiro entre 1999 e 2000 em todos os hospitais públicos, incluindo 19.263 indivíduos, a sobrevida de 1 ano foi 93% e a de 15 anos, 57%. Nesse estudo, em comparação aos homens, as mulheres apresentaram maior taxa de sobrevida 15 anos após a ICP. ^135^

#####  B. Mortalidade relacionada a revascularização cirúrgica

- De acordo com dados de 2017 do Sistema Nacional de Saúde, 21.474 CRVM foram realizadas em instituições públicas no Brasil, com mortalidade hospitalar de 5,37% e média de permanência hospitalar de 12,2 dias ( Tabela 1-7 ).
- O Projeto BYPASS é um banco de dados em andamento estabelecido em 2015 pela Sociedade Brasileira de Cirurgia Cardiovascular, envolvendo 17 instituições representativas de todas as regiões brasileiras. Entre os 2.292 pacientes arrolados até novembro de 2018, submetidos a CRVM isolada ou combinada, a mortalidade hospitalar foi 2,8%, 5,3% permaneceram em ventilação mecânica por mais de 24 horas e 1,2% apresentaram um acidente vascular cerebral intra-hospitalar. ^136 , 137^
- O MASS II foi um ensaio clínico randomizado unicêntrico, desenhado para comparar os efeitos de longo prazo de terapia medicamentosa, angioplastia ou estratégias cirúrgicas para tratar DAC multivascular com angina estável e função ventricular preservada, conduzido antes de 2007. As taxas de mortalidade hospitalar para ICP e CRVM foram 2,4% e 2,5%, respectivamente. ^138^ As taxas de sobrevida de 10 anos não diferiram significativamente entre os grupos: 74,9% para CRVM, 75,1% para ICP e 69% para terapia medicamentosa (p=0,089). ^139^ No ensaio MASS III, taxas de sobrevida de 10 anos similares foram descritas. ^140^
- Várias outras experiências unicêntricas, com análise tanto retrospectiva quanto prospectiva, relataram mortalidade hospitalar para pacientes submetidos a CRVM variando de 1,9% a 8,7%. ^141 - 143^

### Carga de Doença

- O GBD 2017 estimou 1.736 (IC 95%, 1.689 - 1.779) DALYs perdidos por DAC por 100 mil indivíduos, com taxas menores para mulheres (1.298; IC 95%, 1.340 - 1.250) do que para homens (2.194; IC 95%, 2.112 - 2.258). Essa perda de DALYs correspondeu a 6,1% (IC 95%, 5,5% - 6,7%) de todos os DALYs perdidos. Houve significativa queda nessas taxas nas 2 últimas décadas em todas as regiões (variação anual de -2,51%) (Figura 3-6).
- De 1990 a 2017, houve declínio nos DALYs perdidos tanto para homens (-47%) quanto para mulheres (-52%) em todas as unidades federativas. Foram observadas reduções relativas mais expressivas nos estados do Sul e Sudeste, e menores reduções nos estados do Norte e Nordeste ( Tabela 3-3 ).
- A taxa de YLLs por DIC foi 1.653 por 100 mil indivíduos (IC 95%, 1.607 - 1.688), sendo menor para mulheres do que para homens. Esses YLLs corresponderam a 9,7% (IC 95%, 9,4% - 9,9%) de todos os YLLs no GBD 2017.

### Utilização e Custo da Atenção à Saúde

#### (Ver Tabelas 1-6 a 1-9 e Figuras 1-15 e 1-16)

- Uma análise da base de dados administrativos do SUS mostrou que, em 2018, a quantia total reembolsada por procedimentos coronários intervencionistas foi de R$ 569.314.580 (Int$ 280.727.110), dos quais, R$ 73.429.322 (13%) (Int$ 36.202.821) foram relacionados a angioplastias primárias. O valor médio pago por paciente foi de R$ 6.369 (Int$ 3.230). Quanto à CRVM, a quantia total foi de R$ 275.110.234 (Int$ 135.655.933), correspondendo a um valor médio de R$ 13.307 (Int$ 6.561) por hospitalização cirúrgica.
- Os valores reembolsados não ajustados associados com procedimentos de revascularização coronária (códigos para angioplastia e CRVM) aumentaram significativamente de 2008 a 2018, embora em diferentes magnitudes. Para as angioplastias percutâneas, os valores médios subiram 16% (de R$ 5.437 para R$ 6.351) e para as CRVM, 46% (de R$ 9.192 para R$ 13.140) no período.
- Em 2015, utilizou-se uma abordagem de modelagem global para avaliar o impacto econômico (sistema de saúde e produtividade) de quatro condições cardíacas no Brasil, com estimativas de custo anual para 2015. Estimou-se que as quatro condições cardíacas afetassem ˜45,7 milhões de pessoas no Brasil, correspondendo a 32,0% da população adulta. O IM representou o maior custo financeiro, com prevalência estimada de 0,2% (334.978 casos), com custo para o sistema de saúde por caso de US$ 48.118 e custo de produtividade de US$ 18.678. ^115^
- O custo anualizado para um indivíduo com DAC crônica foi estimado em R$ 2.733 ± 2.307 pelo SUS, com o custo para paciente ambulatorial representando 54% do total. Para os planos de saúde privados, o custo foi estimado em R$ 6.788 ± 7.842, dos quais, 69% estavam relacionados a pacientes internados. Quanto ao custo dos pacientes ambulatoriais, os medicamentos foram responsáveis por R$ 1.154, representando, para os pagadores públicos e privados, 77% e 55% dos custos com os pacientes ambulatoriais, respectivamente, e 42% e 17% do custo total, respectivamente. ^144^
- Outro registro de uma clínica de DIC de um hospital público mostrou um custo médio anual para manejo ambulatorial de US$ 1.521 por paciente (valores de 2015). O custo médio por hospitalização foi US$ 1.976 e os gastos foram maiores no primeiro e último anos de seguimento. Angina instável, procedimentos de revascularização, diabetes, hipertensão e obesidade foram preditores de maior custo de hospitalização. ^145^
- Os custos anuais com atenção à saúde de indivíduos com DCV são 3 vezes maiores do que aqueles de indivíduos sem DCV no sistema público de saúde (R$ 4.626 *vs* . R$ 1.312). No setor privado de saúde, a diferença é ainda maior (R$ 13.453 *vs* . R$ 1.789 – ajustado para 2014). ^146^
- De acordo com dados do DATASUS, de 2008 a 2014, 4.653.884 procedimentos cardíacos diagnósticos foram realizados no Brasil, incluindo 3.015.993 eletrocardiogramas, 862.627 angiografias invasivas e 669.969 testes nucleares, levando a um custo geral de US$ 271 milhões. Na avaliação geoespacial nacional do acesso à saúde, mortalidade por DAC foi associada a menor renda, assim como à realização de menor número de testes nucleares e maior número de testes de esforço e de cateterismos cardíacos. ^147^
- De acordo com dados administrativos do SUS, na última década, houve aumento de 40% no número absoluto de angioplastias primárias para manejo de IAM, passando de 7.648 em 2008 (4,03 por 100 mil habitantes) para 10.811 (5,19 por 100 mil habitantes) em 2018. Observou-se tendência similar para admissões hospitalares por DAC. O número de angioplastias coronárias quase dobrou no período, enquanto o de CRVM permaneceu estável.
- Quanto às angioplastias não classificadas como primárias segundo os dados administrativos do SUS (códigos de procedimento: 0406030073, 0406030014, 0406030065, 0406030022, 0406030030), a proporção daqueles procedimentos que ocorreu no contexto de hospitalização por IM aumentou de 2008 para 2018 (de 12% para 31%, respectivamente). Além disso, as angioplastias coronárias realizadas durante hospitalizações por IAM aumentaram em 518%, enquanto aquelas por DAC crônica aumentaram apenas em 70%. Isso revela uma mudança no perfil dos pacientes submetidos a intervenções coronárias, seguindo as recomendações das atuais diretrizes, nas quais angioplastias são mais recomendadas para DAC aguda do que para DAC crônica. De fato, em 2018, 70% de todas as angioplastias para as quais foi designado um daqueles códigos de procedimento ocorreram no contexto de DAC aguda.
- A análise de custo de 101 pacientes submetidos a ICP em 2014 e 2015 mostrou um custo mediano de R$ 6.705 ± 3.116 por paciente. Esse custo foi menor para ICP eletiva, R$ 5.085 ± 16, do que aquele para SCA, R$ 6.854 ± 3.396. ^148^
- Um estudo quantitativo, descritivo e transversal realizado em um hospital filantrópico de São Paulo, avaliando 1.913 pacientes consecutivos submetidos a CRVM em 2012, relatou um custo total médio por paciente de US$ 7.993 [mediana, US$ 6.463], uma receita do sistema público de saúde de US$ 3.450 [3.159] e um déficit estimado de -51% do custo total para os fornecedores. ^149^
- Uma análise retrospectiva das solicitações médicas de beneficiários de planos de saúde foi realizada considerando os custos de hospitalização para pacientes admitidos com SCA entre 2010 e 2012. O custo médio por paciente em terapia medicamentosa apenas foi de R$ 18.262, o custo médio por paciente submetido a ICP, de R$ 30.611, e o custo médio por paciente submetido a CRVM, de R$ 37.455. ^150^

### Pesquisa Futura

• Dados adicionais são necessários para a melhor compreensão da distribuição epidemiológica da DAC no Brasil, sugerindo-se, em particular:

— Desenvolvimento de bases de dados nacionais para coletar informação precisa em tempo real sobre a epidemiologia das diferentes apresentações clínicas da DAC, incluindo prestação do cuidado, e sobre medidas de desempenho e desfecho.

— Revisões sistemáticas de taxas de prevalência de SCA e mortalidade por SCA, pacientes estáveis e após ICP e CRVM, incluindo amostras representativas de todas as áreas geográficas do país.

— Avaliação da efetividade de programas estruturados em âmbito nacional para medir a qualidade e o desempenho dos diferentes fornecedores (público, com e sem fins lucrativos) para entender a atual situação, além de desenhar estratégias visando à redução da morbimortalidade por DCV.

- Análises adicionais econômica e de custo-efetividade do impacto da DAC e de suas intervenções diagnóstica e terapêutica são necessárias, a partir de um nível macro e utilizando métodos de microcusteio para os sistemas de saúde público e privado.
- É de fundamental importância que se desenvolvam programas estruturados para avaliar a prevalência, a incidência e o impacto clínico e econômico da DAC crônica no cenário ambulatorial.

**Tabela 3-1:** – Taxas de prevalência de doença arterial coronariana padronizadas por idade (por 100 mil habitantes) no Brasil e suas unidades federativas, para ambos os sexos, e para homens e mulheres, 1990 e 2017, e variação percentual das taxas

Unidades federativas	Ambos os sexos			Mulheres			Homens		
	1990	2017	Variação percentual (II 95%)	1990	2017	Variação percentual (II 95%)	1990	2017	Variação percentual (II 95%)
									
Acre	1640.6 (1532.9;1756.6)	1483.4 (1387.7;1581.8)	-9.6 (-6.5;-12.8)	1083.4 (1008.2;1165.6)	923.1 (852.4;993.5)	-14.8 (-10.9;-18.5)	2120.8 (1978.5;2276.6)	2063.9 (1928.2;2201.2)	-2.7 (1.7;-7.1)
Alagoas	1671.2 (1568;1784.7)	1506.8 (1412.5;1611.2)	-9.8 (-6.8;-12.5)	1150.1 (1070.1;1235.1)	969.9 (901.4;1043.3)	-15.7 (-11.8;-19.4)	2251.6 (2099.8;2410.2)	2164.3 (2031.8;2318.6)	-3.9 (0.8;-7.7)
Amapá	1450.7 (1353.6;1551)	1435.3 (1340.1;1536.3)	-1.1 (2.6;-4.4)	991.2 (916;1066.2)	903.8 (837.3;975.3)	-8.8 (-4.6;-13.2)	1927.9 (1798.7;2058.4)	2003 (1862.3;2143.5)	3.9 (8.8;-0.4)
Amazonas	1411.5 (1316.1;1512.7)	1407.8 (1315.6;1504.9)	-0.3 (3.5;-3.7)	956.9 (888.2;1034.4)	879.4 (815.1;947.3)	-8.1 (-4.1;-12.2)	1865.3 (1737.9;2002.7)	1958.7 (1823.7;2096.5)	5 (9.8;0.5)
Bahia	1644.7 (1540.7;1753.3)	1558.9 (1459.5;1659.1)	-5.2 (-2;-8.4)	1132.7 (1049.9;1215.3)	1001 (922;1077.6)	-11.6 (-7.4;-15.8)	2218.2 (2076.7;2364.7)	2223.4 (2085;2372.7)	0.2 (4.8;-3.9)
**Brasil**	**1674.5 (1573.5;1784)**	**1563.7 (1465.6;1669.4)**	**-6.6 (-5.2;-7.8)**	**1159.3 (1082.7;1238.5)**	**1008 (938.1;1080.6)**	**-13.1 (-11.4;-14.7)**	**2259.7 (2126.8;2403.2)**	**2229.4 (2098.2;2372.8)**	**-1.3 (0.4;-2.9)**
Ceará	1455.2 (1356.6;1559.7)	1452 (1350.5;1561.1)	-0.2 (3.2;-3.8)	1009.3 (931.1;1092.2)	938.9 (860.1;1021.8)	-7 (-2.1;-11.6)	1955.1 (1824;2091.9)	2066.7 (1922.7;2221.3)	5.7 (10.6;0.6)
Distrito Federal	1474.8 (1386;1569.3)	1404.4 (1315.5;1499.5)	-4.8 (-1.8;-7.8)	1025.9 (952.7;1100.2)	915.8 (849.4;988.1)	-10.7 (-6.8;-14.6)	2009.9 (1885;2147.1)	2046.6 (1914.8;2184.4)	1.8 (6.4;-2.4)
Espírito Santo	1563.5 (1462.6;1663.4)	1473.9 (1378.5;1573.3)	-5.7 (-2.5;-8.7)	1072.1 (991.1;1153)	942 (867.7;1015.4)	-12.1 (-7.8;-16.1)	2091.3 (1954.6;2229.9)	2094.4 (1960.9;2243.1)	0.1 (4.4;-4)
Goiás	1663.5 (1563.6;1775.3)	1596.3 (1498.4;1700.3)	-4 (-0.5;-7.4)	1115.1 (1037.1;1195.5)	1006.6 (934.1;1083.7)	-9.7 (-5.3;-14)	2184.7 (2051;2333.6)	2249.7 (2117.6;2405.1)	3 (7.9;-1.4)
Maranhão	1604.3 (1501;1714.3)	1491.9 (1391.9;1600.2)	-7 (-3.8;-10.1)	1104.3 (1022.7;1183.9)	949.8 (877.8;1025.4)	-14 (-9.9;-17.8)	2138.3 (1994.2;2294.4)	2095.5 (1953.1;2255)	-2 (2.8;-6.7)
Mato Grosso	1633.2 (1524.5;1746.9)	1528.7 (1428.2;1637.1)	-6.4 (-3.4;-9.5)	1072.6 (997.6;1154.4)	941.6 (873.1;1012.4)	-12.2 (-8.2;-16.2)	2097.9 (1955.3;2247.1)	2099.9 (1963.9;2250.9)	0.1 (4.1;-4.3)
Mato Grosso do Sul	1671.6 (1569.6;1787.9)	1602.8 (1501.1;1711.9)	-4.1 (-0.6;-7.2)	1114.7 (1034.9;1194.8)	1008 (931.9;1089.9)	-9.6 (-5.3;-13.5)	2190.1 (2052.1;2342.3)	2253.6 (2104.9;2408.3)	2.9 (7.8;-1.7)
Minas Gerais	1635 (1533.4;1743.3)	1571.7 (1473.5;1676.6)	-3.9 (-0.9;-6.9)	1130.7 (1054.5;1213.7)	1005 (931.7;1084.4)	-11.1 (-7.2;-15.1)	2209.6 (2062.1;2361.5)	2230 (2092.6;2384.2)	0.9 (4.9;-3.1)
Pará	1494.5 (1393.7;1598.9)	1461.5 (1363.1;1567.8)	-2.2 (1.3;-5.4)	1018.3 (939.5;1099.9)	914.1 (844.1;996.3)	-10.2 (-5.8;-14.6)	1981.7 (1840.9;2123.7)	2031.3 (1893.8;2172.4)	2.5 (7.5;-1.7)
Paraíba	1580.3 (1473.9;1688.4)	1483.4 (1382.1;1589.2)	-6.1 (-3;-9)	1093.7 (1010.1;1178.4)	958 (888.5;1032.7)	-12.4 (-8.2;-16.4)	2137.4 (1994.7;2291.2)	2133.3 (1989.7;2288.1)	-0.2 (4.4;-4.5)
Paraná	1751.9 (1639.3;1864.8)	1612.4 (1508;1723.5)	-8 (-4.5;-10.9)	1193.9 (1111;1282.8)	1033.6 (959.7;1111.8)	-13.4 (-9.4;-17.2)	2327.5 (2172.7;2484.7)	2290.6 (2147.7;2451.6)	-1.6 (3.1;-6)
Pernambuco	1623.1 (1514.3;1734.5)	1523.7 (1426.1;1629.4)	-6.1 (-2.8;-9.1)	1132.6 (1047.6;1214.9)	992.2 (916.5;1067)	-12.4 (-8.1;-16.5)	2210.5 (2065.8;2374.7)	2206.4 (2064.2;2362.1)	-0.2 (4.3;-4.6)
Piauí	1485.8 (1389.6;1590.6)	1446 (1348.2;1549)	-2.7 (0.7;-5.8)	1021.5 (945.7;1100.5)	922.3 (853;992.7)	-9.7 (-5.5;-13.8)	1992.1 (1858.4;2134.9)	2052.7 (1910.4;2199.7)	3 (7.7;-1.2)
Rio de Janeiro	1686.3 (1576;1809)	1572.3 (1471.2;1676.3)	-6.8 (-3.7;-10)	1182.7 (1093.6;1286.2)	1027.8 (948.9;1110.8)	-13.1 (-9;-17.2)	2341.8 (2186.6;2513.1)	2288.8 (2129;2438.1)	-2.3 (2.1;-6.5)
Rio Grande do Norte	1458.3 (1361.9;1562.6)	1471.1 (1371.1;1572.6)	0.9 (4.4;-2.6)	1011.9 (935.3;1092.3)	953.8 (876.8;1029.9)	-5.7 (-1.2;-10.1)	1954.9 (1823.4;2096.9)	2097.1 (1956.4;2244.8)	7.3 (12.2;2.3)
Rio Grande do Sul	1793.9 (1676.8;1914.8)	1641.5 (1532.1;1756.2)	-8.5 (-5.4;-11.4)	1254.7 (1160.7;1358.6)	1064.5 (981.2;1149.1)	-15.2 (-11.1;-19.1)	2457.9 (2294.2;2621.4)	2349.8 (2195.1;2513.4)	-4.4 (-0.1;-8.3)
Rondônia	1648.7 (1539.6;1757.9)	1485.5 (1387.3;1588.9)	-9.9 (-6.6;-13.3)	1072.2 (993.2;1153.8)	914.5 (842.3;990.6)	-14.7 (-10.6;-18.4)	2101 (1957.3;2246.4)	2041.5 (1905;2179.1)	-2.8 (1.8;-7.4)
Roraima	1583.8 (1480.2;1691.3)	1418.8 (1330.3;1512.8)	-10.4 (-7.4;-13.5)	1021.1 (948.7;1092.6)	862.9 (798.2;925.9)	-15.5 (-11.6;-19.1)	2010.7 (1870.7;2154)	1935.6 (1810.6;2075)	-3.7 (0.5;-7.9)
Santa Catarina	1770.3 (1655.4;1892.5)	1651.5 (1547.2;1753.9)	-6.7 (-3.5;-9.5)	1219 (1129.9;1317.9)	1054.9 (977.2;1138.7)	-13.5 (-9.3;-17.2)	2383.9 (2230.9;2553.1)	2346.6 (2193.4;2503.1)	-1.6 (2.6;-5.3)
São Paulo	1802.7 (1689.6;1922.7)	1617.6 (1515.9;1730.3)	-10.3 (-7.3;-13)	1251.7 (1162.4;1346.3)	1051.7 (973.2;1132.7)	-16 (-11.9;-19.8)	2455.1 (2298.6;2620)	2324 (2176.2;2488.7)	-5.3 (-1.1;-9.1)
Sergipe	1497.6 (1400.5;1600.2)	1419.8 (1327.5;1520.9)	-5.2 (-1.8;-8.3)	1041.2 (966.2;1120.6)	918.5 (844;988.3)	-11.8 (-7.3;-16.1)	2024.6 (1889.5;2175)	2038.2 (1905.1;2188.1)	0.7 (5.2;-3.5)
Tocantins	1621 (1515.9;1725.6)	1553.5 (1450;1661.8)	-4.2 (-0.7;-7.4)	1074.3 (1000.6;1152.9)	953.2 (878.9;1032.1)	-11.3 (-6.8;-15.1)	2110.4 (1972.8;2257.3)	2133.4 (1993;2284.5)	1.1 (5.9;-3.2)

*Fonte: Estudo Global Burden of Disease 2017, Institute for Health Metrics and Evaluation.151*

**Tabela 3-2:** – Número de mortes e taxas de mortalidade por doença isquêmica do coração padronizadas por idade (por 100 mil habitantes) no Brasil e suas unidades federativas, 1990 e 2017, e variação percentual das taxas

Unidades federativas	1990		2017		Variação percentual (II 95%)
	Número (II 95%)	Taxa (II 95%)	Número (II 95%)	Taxa (II 95%)	
Acre	189.4 (182.1;196.3)	136.6 (131.4;141.7)	357.5 (333;378.2)	65.8 (61.2;69.6)	-51.8 (-55.6;-48.2)
Alagoas	1651.4 (1570;1736.9)	130.1 (123.8;136.9)	2673 (2560.9;2792.4)	88.9 (85;93.1)	-31.7 (-36.7;-26.7)
Amapá	98 (93.9;101.6)	125.3 (119.8;130)	272.6 (254;287.1)	64.1 (59.8;67.4)	-48.9 (-51.9;-45.6)
Amazonas	759.2 (710.6;793.9)	119.1 (111.3;124.4)	1437.4 (1353.9;1501.7)	59.3 (56;62.1)	-50.2 (-53.2;-47)
Bahia	7454.5 (7013;7908)	114.8 (108.1;121.6)	10948.6 (10506.6;11402.3)	68.9 (66;71.8)	-40 (-44.1;-35.8)
Brasil	129011.3 (126506.8;131646)	169.1 (165.8;172.5)	175791.5 (171318.3;179410.1)	80 (77.9;81.7)	-52.7 (-53.9;-51.4)
Ceará	3476.7 (3149.9;3838)	88 (79.8;97)	6852.5 (6528.4;7135.3)	68.7 (65.3;71.5)	-22 (-30.1;-12.5)
Distrito Federal	693.4 (670.8;723.3)	163.8 (159.2;169)	1255 (1188.1;1334.8)	68.1 (64.3;72.4)	-58.4 (-61;-55.5)
Espírito Santo	1962.9 (1903.6;2020.5)	184 (178.6;189.5)	2849 (2723.4;2991.5)	70.1 (67;73.5)	-61.9 (-63.7;-60.2)
Goiás	2187.7 (2095.2;2284.9)	148.7 (142.7;155.3)	4608.3 (4418.6;4837.6)	73.8 (70.8;77.3)	-50.3 (-52.7;-47.9)
Maranhão	2681.4 (2413.1;2998.8)	109.5 (98.6;122.8)	5059.4 (4771.1;5411.4)	81.3 (76.6;87)	-25.8 (-31.9;-18.4)
Mato Grosso	1038.9 (971.1;1108.6)	157 (147.1;166.7)	2001.7 (1905.6;2108)	71.9 (68.5;75.7)	-54.2 (-57.6;-50.4)
Mato Grosso do Sul	1279 (1236.9;1322.1)	178 (172.7;183.3)	2417 (2305.2;2535.9)	92.1 (87.8;96.5)	-48.3 (-51.1;-45.2)
Minas Gerais	13676.9 (13229;14143.9)	174.9 (169.3;180.2)	16472.4 (15831.1;17142.3)	65.4 (62.8;68)	-62.6 (-64.5;-60.6)
Pará	2429.1 (2281.2;2555)	132.1 (124;138.8)	4475.8 (4255.9;4707.2)	72.4 (68.9;76.1)	-45.2 (-49;-41.4)
Paraíba	2656.4 (2457.3;2857.9)	119.5 (110.8;128.7)	4347.9 (4037.2;4662.3)	92.4 (85.8;99.2)	-22.7 (-30.6;-12.9)
Paraná	7635.2 (7436.3;7844.1)	213.6 (208.1;218.8)	10022.2 (9595.8;10414)	83.9 (80.3;87.2)	-60.7 (-62.5;-59)
Pernambuco	6654.6 (6472.6;6840.5)	172 (167;176.9)	9894.9 (9459.7;10308.8)	102.5 (97.8;106.9)	-40.4 (-43.4;-37.4)
Piauí	1454.4 (1315.5;1623.6)	110.5 (100.2;123.7)	2657.7 (2534.2;2880.6)	73.5 (70;79.6)	-33.5 (-39.4;-26.8)
Rio de Janeiro	19105.3 (18615.3;19589.5)	237.3 (231.6;242.9)	21214.7 (20296.2;22025.8)	99.8 (95.4;103.5)	-58 (-59.7;-56.2)
Rio Grande do Norte	1678.9 (1569.7;1800.4)	107.4 (100.4;115.1)	3054.6 (2909.4;3207.9)	80.2 (76.3;84.4)	-25.3 (-32;-17.8)
Rio Grande do Sul	10753.7 (10391.3;11026.2)	200.1 (193;205.2)	11915.8 (11240.8;12411.3)	80.6 (76.1;84.1)	-59.7 (-61.5;-57.9)
Rondônia	483.1 (449.2;518.8)	183.3 (172.6;193.6)	1115.8 (1002.5;1234.4)	86.9 (78.3;95.9)	-52.6 (-57.9;-47.2)
Roraima	67.3 (61.3;74)	180.3 (167.7;194)	207.7 (184.9;232.6)	82 (73.6;91.2)	-54.5 (-60.4;-48)
Santa Catarina	3677.2 (3564.2;3771.3)	189.9 (183.9;195)	5461 (5194.3;5690.8)	77.7 (74;80.9)	-59.1 (-61.1;-57.2)
São Paulo	33956.3 (33066.3;34860.2)	216.3 (210.7;221.3)	41732.9 (40054.1;43206.1)	84.2 (80.8;87.2)	-61.1 (-62.6;-59.5)
Sergipe	882.9 (837.6;933.2)	108.6 (103.1;114.7)	1509.1 (1440.4;1579.9)	72.8 (69.5;76.3)	-32.9 (-37.1;-28.4)
Tocantins	427.5 (379.8;475.3)	153.9 (139.6;169.6)	977 (912.6;1047.3)	73.4 (68.6;78.6)	-52.3 (-57.4;-46.8)

* Fonte: Estudo Global Burden of Disease 2017, Institute for Health Metrics and Evaluation. ^151^*

**Tabela 3-3:** – Número de DALYs e taxas de DALYs padronizadas por idade (por 100 mil habitantes) por doença isquêmica do coração, no Brasil e suas unidades federativas, 1990 e 2017, e variação percentual das taxas

Unidades federativas	1990		2017		Variação percentual (II 95%)
	Número (II 95%)	Taxa (II 95%)	Número (II 95%)	Taxa (II 95%)	
Acre	4490.5 (4309.4;4679.4)	2481.7 (2383.9;2581.2)	7971 (7417;8477.2)	1295.1 (1208;1374.4)	-47.8 (-51.7;-44.2)
Alagoas	38381.3 (36257.4;40572.7)	2644.8 (2506.1;2789.1)	58660.8 (55937.2;61544.4)	1852.2 (1766.3;1942.7)	-30 (-35.3;-25.1)
Amapá	2256.8 (2161.6;2349.2)	2139.7 (2042.8;2217.4)	6578.5 (6114.7;6944.1)	1275.9 (1188.4;1345.7)	-40.4 (-44;-36.6)
Amazonas	17148.9 (16081.9;18005.8)	2072.2 (1942.7;2171.1)	31464.8 (29293.6;33102.3)	1136.6 (1066.1;1192.7)	-45.1 (-48.5;-41.7)
Bahia	166604.8 (156031.4;177498)	2294.4 (2154;2441)	231348.2 (220711.5;241597.1)	1457.6 (1390.2;1521.1)	-36.5 (-41.1;-31.4)
Brasil	2959511 (2894436.2;3033217.1)	3159.1 (3091.6;3233.5)	3678315.8 (3579145.9;3768782)	1602.4 (1559.2;1641.9)	-49.3 (-50.7;-47.8)
Ceará	71384.3 (64483.4;79273.1)	1691.4 (1530.8;1874.3)	128192.5 (121929.7;134050.4)	1307.4 (1243.4;1368.7)	-22.7 (-30.6;-13.7)
Distrito Federal	19308.3 (18564.4;20275.2)	2845.7 (2756.4;2962.1)	27808.1 (26221.3;29618.7)	1121.3 (1059.8;1190.4)	-60.6 (-62.8;-58.2)
Espírito Santo	44177 (42688.1;45695)	2975.1 (2882.6;3065.8)	59450.4 (56507.8;62558.7)	1381.9 (1314.9;1451.2)	-53.6 (-55.8;-51.3)
Goiás	55914.3 (53327.6;58557.6)	2636.3 (2522.6;2754.3)	105343.2 (100500;110380.6)	1523.3 (1454.2;1595.6)	-42.2 (-45.2;-39.3)
Maranhão	73066.2 (65836.5;81337.6)	2557 (2305.8;2845.1)	109267.8 (103121.4;115769.7)	1680.2 (1586.4;1780.6)	-34.3 (-39.8;-27.3)
Mato Grosso	26946.1 (25171.5;28837)	3010.9 (2820.1;3213.2)	46202.4 (43768.5;48781)	1433.9 (1363;1510.9)	-52.4 (-56.1;-48.3)
Mato Grosso do Sul	30871.7 (29551.5;32110.1)	3216 (3102.7;3326.8)	52574.5 (50174.7;55121.4)	1835.5 (1754.7;1923.1)	-42.9 (-46.1;-39.5)
Minas Gerais	313998.9 (302102.3;326795.2)	3087.6 (2980.2;3202.3)	343731.3 (329343.3;358925.5)	1346.9 (1290.4;1406.4)	-56.4 (-58.7;-53.9)
Pará	54735.8 (51421.7;57718.5)	2415.2 (2272.2;2540.5)	100381.7 (95156.5;105848.4)	1469.8 (1395.9;1547.3)	-39.1 (-43.1;-35.1)
Paraíba	54949.7 (50451.8;59641.8)	2344.6 (2160.6;2543.5)	82981 (77060.1;89538.2)	1821.8 (1692;1966.4)	-22.3 (-30.7;-12.1)
Paraná	174525.4 (169345.9;179809.9)	3561.7 (3464.6;3661)	206379.1 (196027.1;215789.4)	1595.2 (1516.5;1666.7)	-55.2 (-57.4;-53.1)
Pernambuco	144001.4 (139657.6;148414.6)	3110.2 (3018.9;3200.9)	207666.8 (198225.5;216500.9)	2076.1 (1982.5;2164.9)	-33.2 (-36.7;-29.9)
Piauí	33224.1 (29689.6;37114.1)	2189.2 (1970.8;2442.1)	54215.2 (51650.7;57900)	1502.3 (1430.9;1604.2)	-31.4 (-37.5;-24.5)
Rio de Janeiro	454987.3 (442500.6;467807.1)	4481.1 (4365.5;4597)	450050 (429870;467261.3)	2063.8 (1970.8;2142.5)	-53.9 (-55.9;-52)
Rio Grande do Norte	32424.2 (30203.4;34875.9)	1972.3 (1838.5;2120.1)	60710.1 (57559.4;64019.8)	1623.5 (1539.9;1712.6)	-17.7 (-25.2;-9.2)
Rio Grande do Sul	242410.1 (233975.5;249439.7)	3573.4 (3449.5;3665.5)	231413.1 (216004.4;242521.6)	1542.5 (1439.4;1617.4)	-56.8 (-59;-54.6)
Rondônia	14130.4 (13109.8;15219.2)	3388.7 (3169.3;3608.1)	24830 (22172.1;27619.4)	1657 (1487.6;1836.9)	-51.1 (-57;-44.9)
Roraima	1916.4 (1730.6;2127)	2919.1 (2687.8;3186.5)	4797.3 (4262.1;5419.4)	1374.5 (1225;1539.8)	-52.9 (-59.4;-45.6)
Santa Catarina	82034.2 (79539.6;84426.2)	3208.9 (3105;3295.8)	112363.3 (106011.6;117727.3)	1436.9 (1357.2;1503.9)	-55.2 (-57.5;-53)
São Paulo	777221.4 (755207.8;800862.7)	3746.1 (3646.7;3850.3)	880339.1 (842359;915273)	1669.2 (1598.4;1735)	-55.4 (-57.2;-53.5)
Sergipe	17580.3 (16558.7;18785.1)	2012.2 (1900.5;2143.3)	32232.2 (30675;33873.9)	1474.5 (1403.3;1547)	-26.7 (-31.9;-21.1)
Tocantins	10821.1 (9291.9;12213.6)	2516.2 (2233.5;2791.5)	21363.6 (19891.7;22878)	1503.1 (1400.6;1606.9)	-40.3 (-47.2;-32.3)

* Fonte: Estudo Global Burden of Disease 2017, Institute for Health Metrics and Evaluation. ^151^*

## 4. CARDIOMIOPATIA E INSUFICIÊNCIA CARDÍACA

### ICD-10 I42; I50; B57.2.

Ver Tabelas 4-1 até 4-14 e Figuras 4-1 até 4-10

**Table:** 

Abreviaturas usadas no Capítulo 4	
BREATHE	I Registro Brasileiro de Insuficiência Cardíaca
CID-10	Classificação Estatística Internacional de Doenças e Problemas Relacionados à Saúde, 10a Revisão
CMCh	Cardiomiopatia Chagásica
CMH	Cardiomiopatia Hipertrófica
CMNCh	Cardiomiopatia Não Chagásica
DALYs	Anos de vida perdidos ajustados por incapacidade (do inglês, Disability-Adjusted Life-Year)
DCh	Doença de Chagas
GBD	*Global Burden of Disease*
HR	*Hazard Ratio*
IC	Intervalo de Confiança
II	Intervalo de Incerteza
IIQ	Intervalo Interquartil
OR	Odds Ratio
RAP	Risco Atribuível na População
REMADHE	* Repetitive Education and Monitoring for ADherence in Heart Failure *
SDI	Índice Sociodemográfico (do inglês, *Sociodemographic Index* )
SEADE	Fundação Sistema Estadual de Análise de Dados
SIM	Sistema de Informações sobre Mortalidade
SUS	Sistema Único de Saúde
UF	Unidade Federativa
YLDs	Anos vividos com incapacidade (do inglês, *Years Lived with Disability* )

### Cardiomiopatia e Miocardite

#### Prevalência e Incidência

- De acordo com as estimativas do Estudo GBD 2017, a prevalência padronizada por idade de cardiomiopatia e miocardite aumentou no Brasil em 9,4% (95 UI, 15,3-4,1) de 1990 a 2017, passando de 102,8 (II 95%, 82,5-125,7) para 112,4 (II 95%, 92,2-134,2), respectivamente ( Figura 4-1 .A e Tabela 4-1 ). Em número absoluto, as estimativas de prevalência de cardiomiopatia e miocardite no Brasil passaram de menos de 100 mil em 1990 para mais de 200 mil em 2017, principalmente devido ao crescimento e envelhecimento da população (Figura 4-1.B). A prevalência de cardiomiopatia e miocardite foi maior em mulheres (115; II 95%, 95-137) do que em homens (109; II 95%, 88-132) em 2017, mas o aumento da prevalência foi maior em homens naquele período, sendo o aumento percentual de 6,9 (II 95%, 0,2-14,2) para mulheres e de 12,3 (II 95%, 5,4-20) para homens.
  **Figura 4-1:**
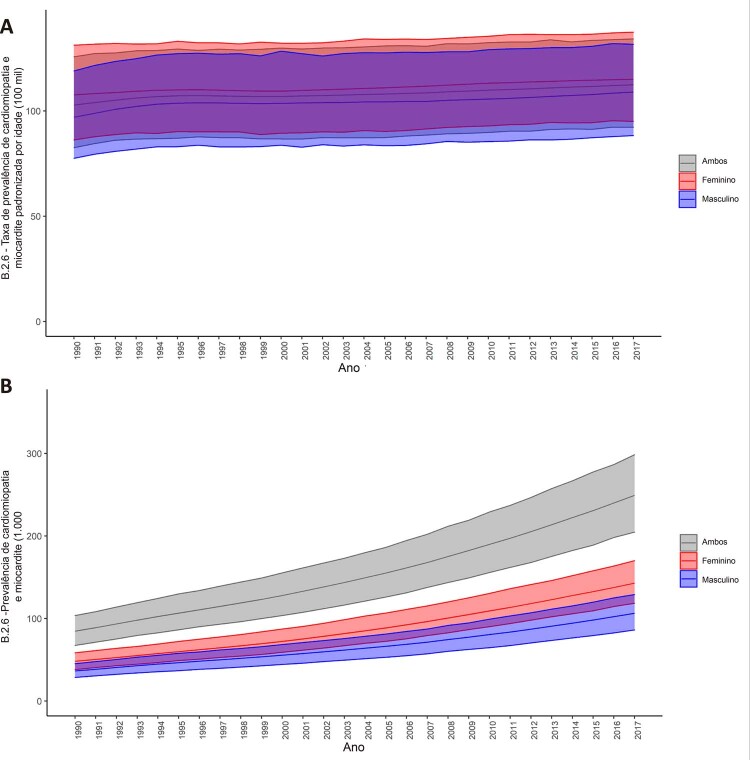
- Taxa de prevalência padronizada por idade (A) e taxa de prevalência bruta (B) por cardiomiopatia e miocardite por 100 mil habitantes, por sexo, Brasil, 1990-2017.

Dados derivados do Estudo Global Burden of Disease 2017 (GBD 2017). ^188^
- De acordo com as estimativas do Estudo GBD 2017, a prevalência de cardiomiopatia e miocardite varia bastante entre as UF brasileiras, não tendo a variação percentual sido homogênea entre 1990 e 2017 ( Tabela 4-1 ). Em 2017, as maiores taxas foram observadas nos estados de São Paulo e Roraima e no Distrito Federal. De 1990 a 2017, houve diminuição da taxa de prevalência padronizada por idade nos estados do Rio de Janeiro, Rio Grande do Sul, Santa Catarina e Espírito Santo, e aumento em todas as outras UF.
- Segundo as estimativas do Estudo GBD 2017, as taxas de incidência padronizadas por idade por 100 mil por ano foram 46,3 (II 95%, 41,5-52,1) em 1990 e 46,7 (II 95%, 41,8-52,6) em 2017, com uma pequena variação de 0,8% (II 95%, -0,3 a 1,8) no período ( Tabela 4-2 ). Os números absolutos de casos incidentes foram 54.520 (II 95%, 48.574-61.321) em 1990 e 103.879 (II 95%, 92.496-117.294) em 2017. Tal aumento acha-se relacionado ao crescimento e envelhecimento da população. A Tabela 4-3 mostra as taxas de incidência de cardiomiopatia e miocardite por 100 mil habitantes, por idade, para ambos os sexos, em 1990 e 2017, e a variação percentual das taxas. Houve um aumento de quase 3 vezes nas taxas de incidência do grupo etário de 15-49 anos para o de 50-69 anos, assim como do último para o de 70+ anos, tendo esses aumentos sido similares para mulheres e homens. De 1990 a 2017, a incidência aumentou em todos os grupos etários para mulheres, enquanto tendeu a diminuir na maioria dos grupos etários para homens.

#### Mortalidade

- De acordo com as estimativas do Estudo GBD 2017, as taxas de mortalidade por cardiomiopatia e miocardite pareceram aumentar na década de 1990, mas diminuíram nas 2 décadas seguintes ( Figura 4-2 ). Como mostra a Tabela 4-4 , as taxas de mortalidade foram 10,9 (II 95%, 9,57-11,38) em 1990 e 8,59 (II 95%, 8,16-9,93) em 2017 por 100 mil habitantes, uma redução de 21,2% (II 95%, -26,8 a -2,6). Apesar dessa diminuição nas taxas de mortalidade, o número de mortes por cardiomiopatia e miocardite aumentou naquele período devido ao crescimento e envelhecimento da população. Cardiomiopatia e miocardite foram responsáveis por 9.734 (II 95%, 8.417-10.163) mortes em 1990, número que se elevou para 18.812 (II 95%, 17.885-21.745) em 2017. As estimativas do Estudo GBD 2017 das taxas de mortalidade por cardiomiopatia referem-se a casos com cardiomiopatia listada como causa básica de morte. As mortes por insuficiência cardíaca que resulta de outras causas específicas são atribuídas à doença de base, i.e., mortes relacionadas a cardiomiopatia isquêmica são codificadas como devidas a doença isquêmica do coração. Além disso, para o projeto GBD, a insuficiência cardíaca não é considerada uma causa de morte primária e, portanto, todas as mortes codificadas como relacionadas a insuficiência cardíaca são recodificadas para a condição de base (ver adiante).
  **Figura 4-2:**
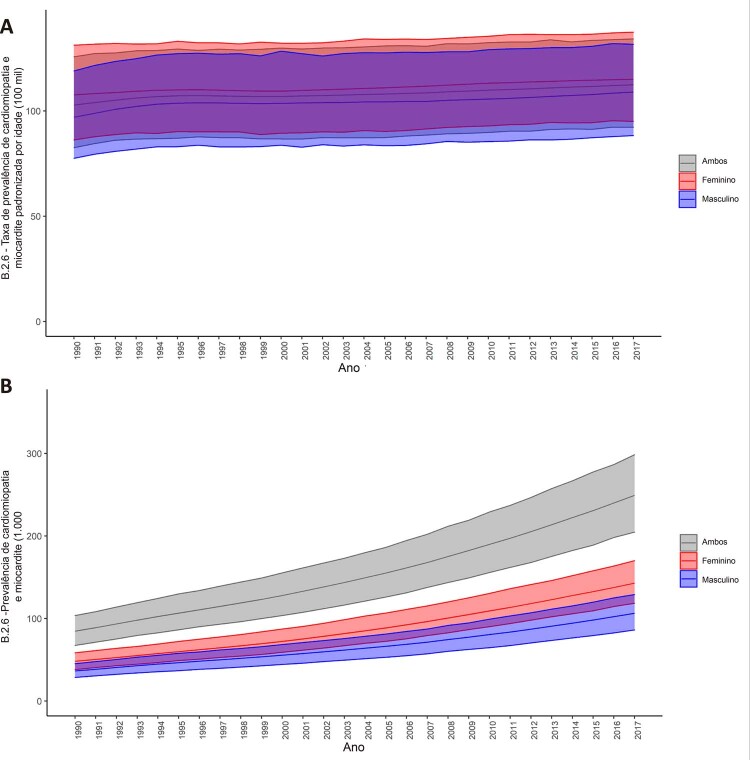
- Taxa de mortalidade padronizada por idade por cardiomiopatia e miocardite, por 100 mil habitantes, por sexo, Brasil, 1990-2017.

Dados derivados do Estudo Global Burden of Disease 2017 (GBD 2017). ^188^
- A Tabela 4-4 também mostra o número total de mortes e taxa de mortalidade padronizada por idade (por 100 mil habitantes) por cardiomiopatia e miocardite, além da variação percentual, por UF e no Brasil, em 1990 e em 2017. Na maioria das UF houve diminuição das taxas de mortalidade, sendo as maiores porcentagens de redução entre 1990 e 2017 observadas no Paraná e em Goiás. Por outro lado, houve aumento das taxas de mortalidade de 1990 a 2017 apenas em 3 UF, sendo o maior aumento, 46,2% (II 95%, 12,6-63,3), observado no estado do Rio de Janeiro. Em 2017, as UF com as mais baixas taxas de mortalidade (abaixo de 5,0) foram Acre, Amazonas, Maranhão, Pará, Rio Grande do Norte e Rio Grande do Sul.
- A Tabela 4-5 mostra as taxas de mortalidade por cardiomiopatia e miocardite de acordo com o sexo e os grupos etários, com base nas estimativas do Estudo GBD 2017. As mulheres apresentaram as mais baixas taxas de mortalidade padronizadas por idade, assim como uma redução mais pronunciada de 1990 a 2017. As taxas de mortalidade por cardiomiopatia e miocardite em mulheres foram 9,20 (II 95%, 8,81-9,93) por 100 mil habitantes em 1990 e 6,3 (II 95%, 6-6,6) por 100 mil habitantes em 2017, uma redução de 31,3% (II 95%, -35,5 a -26,2). As taxas nos homens foram 12,83 (II 95%, 9,96-13,67) por 100 mil habitantes em 1990 e 11,27 (II 95%, 10,46-14,39) por 100 mil habitantes em 2017, uma variação de 12,1% (II 95%, -20,9 a 28,2). Como esperado, as mais altas taxas de mortalidade foram observadas no grupo de 70+ anos, sendo, em 1990, 84,2 (II 95%, 74,2-89,1) por 100 mil habitantes e, em 2017, 76,6 (II 95%, 72,0-89,9) por 100 mil habitantes. Para o grupo de 50-69 anos, as taxas foram 21,2 (II 95%, 17,7-22,5) por 100 mil habitantes em 1990 e 16,5 (II 95%, 15,3-19,3) por 100 mil habitantes em 2017. No geral, as taxas de mortalidade diminuíram entre 1990 e 2017 na maioria dos grupos etários, tendo permanecido estável no grupo de 15-49 anos.
- O Estudo GBD 2017 usa o SDI como uma estimativa do nível socioeconômico de uma localidade. A Figura 4-3 mostra a correlação entre o SDI e a taxa de mortalidade padronizada por idade por cardiomiopatia e miocardite, por 100 mil habitantes, em 1990 e em 2017. As correlações observadas em 1990 e 2017 foram similares e não significativas.
- Em estudo relatando dados da Fundação SEADE, do estado de São Paulo, as cardiomiopatias foram responsáveis por um total de 3.571 óbitos, correspondendo a 23,3% das mortes relacionadas a insuficiência cardíaca em 2006, a saber: cardiomiopatia dilatada, responsável por 17,2% das mortes; cardiomiopatia alcoólica, por 0,45%; e cardiomiopatias restritivas, por 0,37%. A CMCh e a cardiomiopatia alcoólica foram responsáveis por 7,8% e 0,45% das mortes relacionadas a insuficiência cardíaca, respectivamente. ^153^
- Dados de cardiomiopatias específicas são escassos. Em estudo de coorte de 214 pacientes com CMH, acompanhados por 7 anos em um hospital terciário de São Paulo, a idade média foi 37±16 anos, sendo 52% mulheres. Houve 22 mortes (10%), 15 diretamente relacionadas à CMH (11 mortes súbitas). As taxas de sobrevida acumulada foram 94,5% em 5 anos, 91% em 10 anos e 87,9% em 15 anos, com taxa de mortalidade anual de 1%, que é baixa, considerando que o estudo foi realizado em um centro de referência. ^154^

#### Carga de Doença

- De acordo com as estimativas do GBD 2017, as tendências das taxas de DALYs padronizadas por idade por cardiomiopatia e miocardite foram similares àquelas de mortalidade, com pequeno aumento na década de 1990 e diminuição nas décadas seguintes. A Figura 4-4 mostra os DALYs por 100 mil habitantes, em 1990-2017, no Brasil e suas regiões. A região Centro-Oeste apresentou os mais altos DALYs nas 2 primeiras décadas, com redução depois disso, principalmente após 2004. Em 2017, a região Sudeste apresentou os mais altos DALYs, enquanto as regiões Norte e Sul, os mais baixos. Como mostra a Tabela 4-6 , as taxas de DALYs padronizadas por idade foram 286 (II 95%, 247-301) em 1990 e 222 (II 95%, 211-251) em 2017, por 100 mil habitantes, com uma diminuição de 22,4% (II 95%, -27,6 a -7). Tais mudanças são similares àquelas observadas nas taxas de mortalidade. A despeito dessa diminuição nos DALYs, cardiomiopatia e miocardite foram responsáveis por 328.636 (II 95%, 283.325-346.746) DALYs no Brasil em 1990 e por 490.572 (II 95%, 465.903-556.886) DALYs no Brasil em 2017, o que representa 0,81% de todos os DALYs.
- A Tabela 4-7 mostra as taxas de DALYs por cardiomiopatia e miocardite de acordo com sexo e grupo etário, a partir das estimativas do Estudo GBD 2017. As taxas de DALYs padronizadas por idade foram menores nas mulheres, que também apresentaram a redução mais pronunciada de 1990 a 2017. As taxas de DALYs, para mulheres, foram 234 (II 95%, 223-247) por 100 mil habitantes em 1990 e 153 (II 95%, 145-161) por 100 mil habitantes em 2017, uma redução de 34,3% (II 95%, -38,6 a -30). As taxas de DALYs, para homens, foram 343 (II 95%, 262-366) por 100 mil habitantes em 1990 e 299 (II 95%, 280-363) por 100 mil habitantes em 2017, uma redução de 12,8% (II 95%, -21 a 17,6). Como esperado, as mais altas taxas de DALYs foram observadas no grupo de 70+ anos, seguido pelo grupo de 50-69 anos. No geral, as taxas de DALYs diminuíram de 1990 para 2017 na maioria dos grupos etários, aumentando apenas nos homens de 15-49 anos.
- À semelhança do observado para a taxa de mortalidade padronizada por idade, não houve correlação entre o SDI e as taxas de DALYs por cardiomiopatia e miocardite.

####  Doença de Chagas Crônica e Cardiomiopatia Chagásica 

##### Prevalência e Incidência 3

- A prevalência de DCh no Brasil em 2010 foi estimada em 1.156.821 pela Organização Mundial da Saúde, ^155^ sendo essa a última estimativa oficial disponível, publicada em 2015. De acordo com tal estimativa, o número de indivíduos com CMCh no Brasil era 231.364. ^3^ Esses números revelam uma tendência significativa de diminuição de casos humanos de DCh no Brasil em relação a estimativas anteriores, sendo isso atribuído a vários fatores, mas principalmente à quase completa interrupção da transmissão vetorial e transfusional no Brasil.
- De acordo com as estimativas do Estudo GBD 2017, a prevalência padronizada por idade de DCh diminuiu significativamente no Brasil, 44% (II 95%, 42-27), de 1990 a 2017, passando de 1.811 (II 95%, 1.531-2.131) por 100 mil habitantes em 1990 para 1.011 (II 95%, 843-1.198) por 100 mil habitantes em 2017. A prevalência de DCh no Brasil em 2017 foi maior entre os homens [1.029 (II 95%, 863-1.205)] do que entre as mulheres [991 (II 95%, 824-1.186)].
- Em uma revisão sistemática de estudos de base populacional sobre a prevalência de DCh no Brasil realizados de 1980 a setembro de 2012, 42 artigos com dados relevantes de prevalência foram identificados a partir de um total de 4.985 referências. ^156^ A estimativa combinada de prevalência de DCh a partir dos estudos para todo o período foi 4,2% (IC 95%, 3,1-5,7), variando de 4,4% (IC 95%, 2,3-8,3) na década de 1980 a 2,4% (IC 95%, 1,5-3,8) após 2000. A prevalência estimada de DCh para homens e mulheres foi similar (4,1% [IC 95%, 2,6-6,6], 4,2% [IC 95%, 2,6-6,8], respectivamente). A maior estimativa combinada de prevalência foi observada em indivíduos com idade >60 anos (17,7%; IC 95%, 11,4-26,5), nas regiões Nordeste (5,0%; IC 95%, 3,1-8,1) e Sudeste (5,0%; CI, 2,4-9,9) e em áreas mistas (urbana/rural) (6,4%; IC 95%, 4,2-9,4). Estima-se que cerca de 4,6 milhões (IC 95%, 2,9-7,2 milhões) de pessoas tenham sido infectadas com *T. cruzi* em 2010. Essas estimativas são bem maiores do que as da Organização Mundial da Saúde para 2010. ^155^ Os autores observaram grande heterogeneidade na maioria das estimativas combinadas (I(2)>75%; p< 0,001).
- No estudo de coorte retrospectivo sobre Chagas dos *National Institutes of Health* , REDS-II, doadores de sangue inicialmente saudáveis com uma doação-índice soropositiva para *T. cruzi* pareados por idade, sexo e período com doadores soronegativos foram acompanhados por 10 anos. ^157^ A incidência diferencial de cardiomiopatia foi 1,85 por 100 pessoas-ano atribuível à infecção por *T. cruzi* .

##### Mortalidade

- De acordo com o Estudo GBD 2017, o número de mortes por DCh no Brasil diminuiu nas últimas décadas (Figura 4-5). Na década de 1990, a DCh foi responsável por 7.049 (II 95%, 6.816-7.323) mortes, que diminuíram para 5.493 (II 95%, 5.221-6.015) em 2017. A taxa de mortalidade padronizada por idade apresentou redução mais marcante (variação de -67,5%), passando de 7,3 (II 95%, 7,0-7,6) mortes por 100 mil habitantes em 1990 para 2,5 (II 95%, 2,3-2,7) por 100 mil habitantes em 2017, correspondendo a 0,4% de todas as mortes no país. Em 2017, os homens apresentaram maiores taxas de mortalidade padronizadas por idade do que as mulheres (3,1, II 95%, 2,9-3,4; e 1,9, II 95%, 1,8-2,1, respectivamente).
- A Tabela 4-8 demonstra o número total de mortes por DCh, as taxas de mortalidade padronizadas por idade (por 100 mil habitantes), para ambos os sexos, além da variação percentual, por UF e no Brasil, em 1990 e 2017. O número de mortes e as taxas de mortalidade variaram significativamente entre as UF nos dois anos. Em 1990, as maiores taxas de mortalidade (> 10 por 100 mil habitantes) foram observadas em Goiás, Minas Gerais, Bahia e Distrito Federal, com pico em Goiás (60 por 100 mil habitantes, II 95%, 58-66). Todas as UF apresentaram redução nas taxas de mortalidade, que variaram de 43% (II 95%, 52-33) na Bahia a 75% (II 95%, 78-72) em Minas Gerais e Goiás. As Tabelas 4-9 e 4-10 revelam dados estratificados por sexo.
- A redução nas taxas de mortalidade foi mais acentuada (variação de 80%, UI 82-77) no grupo etário de 5-14 anos, passando de 2,6 (UI 2,5-2,7) para 0,5 (UI 0,5-0,6) por 100 mil habitantes. Para os outros grupos etários, a maioria das mortes ocorreu em indivíduos com 70+ anos, que apresentaram a menor redução percentual (47%, UI 51-42) no período 1990-2017: de 41 (UI 39-43) para 22 (20-24) por 100 mil habitantes. A diminuição na taxa de mortalidade padronizada por idade por 100 mil habitantes correlaciona-se com o SDI das UF brasileiras (R2 = 0,40, p=0,01), tendo a UF com mais alto SDI em 1990 apresentado a maior diminuição percentual naquela taxa de 1990 a 2017 (Figura 4-6).
- Vários estudos de base populacional mostraram uma redução na mortalidade por DCh no Brasil nas últimas décadas. Martins-Melo *et al* . ^158^ relataram uma redução gradual em todo o país das taxas de mortalidade padronizadas, de 3,78 (1999) para 2,78 (2007) mortes/ano por 100 mil habitantes (-26,4%). Nóbrega *et al* . ^159^ mostraram que as taxas de mortalidade padronizadas diminuíram em todo o país em 32,4%, passando de 3,4% em 2000 para 2,3% em 2010. A taxa de mortalidade por envolvimento cardíaco diminuiu em todas as regiões do Brasil, exceto na região Norte, onde aumentou em 1,6%. A região Nordeste apresentou a menor redução, enquanto a região Centro-Oeste, a maior. Simões *et al* ., ^160^ estudando a evolução da mortalidade por DCh no Brasil de 1980 a 2014, fizeram uma previsão para a mortalidade de 2015 a 2034. Esses autores estimaram um declínio progressivo na mortalidade por DCh, que seria maior entre os jovens. A redução média esperada foi de 76,1% em comparação ao último período observado (2010-2014) e ao último período previsto (2030-2034). As regiões Centro-Oeste, Sudeste e Sul apresentaram uma redução na taxa de mortes por DCh entre 2000 e 2014. A taxa de mortalidade na região Nordeste não diferiu estatisticamente em nenhum período analisado, mas, na região Norte, apresentou tendência a aumento.
- Tendo por base o SIM, cuja abrangência é nacional, um estudo analisou todas as declarações de óbito emitidas entre 1999 e 2007 no Brasil ^158^ e concluiu que a DCh foi mencionada em 53.930 (0,6%) declarações de óbito: como causa básica em 44.537 (82,6%) declarações de óbito, e como uma causa de morte associada em 9.387 (17,4%) declarações de óbito. A DCh aguda foi responsável por 2,8% das mortes. A taxa de mortalidade padronizada média foi 3,36 por 100 mil habitantes/ano, que é 21% mais alta do que a taxa de mortalidade quando se considera apenas a causa básica de morte (2,78 mortes por 100 mil habitantes/ano). A mortalidade proporcional, considerando-se múltiplas causas de morte foi 0,6%. Os indivíduos que morreram por DCh foram predominantemente do sexo masculino (57%), com idade superior a 60 anos (62,8%) e residentes da região Sudeste (53,6%). A região Centro-Oeste apresentou a maior mortalidade proporcional de todas as regiões (2,17%). ^158^
- Na mesma base de dados, calculando a taxa de mortalidade média para cada município de residência e usando *Empirical Bayesian Smoothing* , uma análise espacial identificou um grande *cluster* com alto risco de mortalidade por DCh, envolvendo 9 estados na região central do Brasil (Figura 4-7). ^161^
- Nóbrega *et al* ., em estudo descritivo usando dados do SIM sobre todas as mortes por DCh no Brasil entre 2000 e 2010, observaram que, no período 2000-2010, a maioria (85,9%) ocorreu em homens com mais de 60 anos, tendo sido causadas por comprometimento cardíaco. No período estudado, a taxa de mortalidade diminuiu em todos as faixas etárias, exceto naquela a partir de 80 anos (Figura 4-8). ^159^
- Um estudo de coorte retrospectivo utilizou ligação probabilística para identificar entre doadores de sangue de 1996 a 2000 (2.842 soropositivos e 5.684 soronegativos para DCh) aqueles que morreram até 2010. ^162^ Os autores identificaram 159 mortes entre os doadores soropositivos (5,6%) e 103 mortes entre os soronegativos (1,8%). Os doadores soropositivos apresentaram um risco 2,3 vezes maior de morte por todas as causas (IC 95%, 1,8-3,0) em comparação aos soronegativos. Entre os doadores soropositivos, apenas 26 tiveram, como causa básica de morte, o código da CID-10 indicativo de DCh (B57.0/B57.5). ^162^ Os autores concluíram que a DCh é uma causa de morte subnotificada na base de dados brasileira de mortalidade.
- Ayub-Ferreira *et al* . compararam o mecanismo de morte na insuficiência cardíaca por CMCh com aquele de outras etiologias em um ensaio clínico prospectivo, o REMADHE, que incluiu pacientes a partir de 18 anos, com insuficiência cardíaca crônica irreversível por pelo menos 6 meses e fração de ejeção ventricular esquerda inferior a 50%. Dos 342 pacientes analisados, 185 (54,1%) morreram, sendo que desses, 56,4% eram portadores de DCh e 53,7% não. De todas as mortes no grupo com DCh, 48,4% foram relacionadas a piora da insuficiência cardíaca, 25,7% a morte súbita e 6,4% a acidente vascular cerebral. A incidência acumulada de mortalidade por todas as causas e mortalidade por insuficiência cardíaca foi significativamente maior em pacientes com DCh do que naqueles sem DCh. ^163^ Não houve diferença na incidência acumulada de mortalidade por morte súbita entre os dois grupos. Na DCh cardíaca grave, insuficiência cardíaca progressiva é o principal mecanismo de morte.
- No Estudo de Coorte de Idosos de Bambuí, um grande estudo de base populacional de idosos residentes em uma área endêmica para DCh, 1.479 indivíduos com idade igual ou superior a 60 anos (38,1% com teste positivo para *T. cruzi* ) foram acompanhados de 1997 a 2007. Durante o acompanhamento médio de 8,72 anos, 567 participantes morreram. A infecção por *T. cruzi* foi um preditor de mortalidade entre os membros da coorte, e essa associação permaneceu altamente significativa após ajustes para idade, sexo e fatores de risco cardiovascular convencionais (HR = 1,56; IC 95%, 1,32-1,85). No geral, o RAP de mortalidade por infecção por *T* . *cruzi* foi 13,2% (IC 95%, 9,8-16,4). ^164^
- Nadruz *et al* . estudaram as tendências temporais no RAP de CMCh para mortalidade em 2 anos entre pacientes com insuficiência cardíaca arrolados nos períodos 2002-2004 (era 1) e 2012-2014 (era 2) em um hospital universitário brasileiro. Foram estudados prospectivamente 362 (15% com CMCh) e 582 (18% com CMCh) pacientes com insuficiência cardíaca e fração de ejeção ≤ 50% nos períodos 1 e 2, respectivamente, tendo-se estimado o RAP de CMCh para mortalidade em 2 anos. Embora os números absolutos de morte tenham diminuído com o tempo nos grupos de CMCh e CMNCh, o RAP de CMCh para mortalidade aumentou entre os pacientes com insuficiência cardíaca [RAP _(era 1)_ = 11,0 (IC 95%: 2,8-18,5%); RAP _(era 2)_ = 21,9 (IC 95%: 16,5-26,9); p=0.023 *vs.* era 1], devido a aumento no HR associado com DCh. ^165^

##### Carga de Doença

- Utilizando achados do Estudo GBD 2016, um estudo mostrou que, em 2016, 141.640 DALYs (II 95%, 129.065-155.941) por DCh foram estimados no Brasil, com redução relativa de 36,7% em comparação a 1990 (223.879 DALYs; II 95%, 209.372-238.591). As taxas de DALYs padronizadas por idade diminuíram em nível nacional (-69,7%) e em todas as UF brasileiras entre 1990 e 2016, mas com diferentes padrões regionais (Figura 4-9). A diminuição nas taxas de DALYs foi primariamente devida a uma redução consistente nos anos de vida perdidos, o principal componente do total de DALYs por DCh. A maior carga fatal e não fatal por DCh foi observada entre os homens e os idosos e nas UF brasileiras com importantes áreas endêmicas de transmissão vetorial no passado, como Goiás, Tocantins, Minas Gerais, Bahia e Distrito Federal. ^166^

### Insuficiência Cardíaca

 • Como a insuficiência cardíaca não é considerada uma causa básica de morte (i.e., código *garbage* ) no Estudo GBD, todas as mortes atribuídas a insuficiência cardíaca nas declarações de óbito são reclassificadas e/ou redistribuídas para outras causas, de acordo com o método do GBD. Assim, não há dados do GBD sobre mortalidade por insuficiência cardíaca. A insuficiência cardíaca é classificada pelo GBD como um “comprometimento”, portanto, os únicos indicadores do GBD para insuficiência cardíaca são prevalência e YLDs, que é o componente de morbidade do DALYs. 

#### Prevalência e Incidência

- De acordo com as estimativas do Estudo GBD 2017, a prevalência padronizada por idade de insuficiência cardíaca no Brasil passou de 818 (II 95%, 718-923) em 1990 para 772 (II 95%, 680-875) em 2017, uma diminuição de 5% (95 UI, -7,1 a -3) no período (Tabela 4-10). Em números absolutos, as estimativas de prevalência de insuficiência cardíaca no Brasil subiram de 0,67 milhão em 1990 para quase 1,7 milhão em 2017, principalmente devido a crescimento e envelhecimento da população. A prevalência de insuficiência cardíaca variou entre as UF brasileiras e a variação percentual não foi uniforme entre 1990 e 2017 ( Tabela 4-11 ). Em 2017, as mais altas taxas foram observadas no Rio Grande do Norte e as mais baixas, no Acre. De 1990 a 2017, taxas de prevalência padronizadas por idade decrescentes foram observadas na maioria das UF, tendo aumento nas taxas ocorrido em 8 UF, principalmente na região Nordeste.
- A Tabela 4-12 mostra a prevalência de insuficiência cardíaca de acordo com sexo e grupo etário, a partir das estimativas do Estudo GBD 2017. A prevalência de insuficiência cardíaca foi maior em mulheres (795; II 95%, 694-901) do que em homens (751; II 95%, 656-845) em 2017, e a redução na prevalência de 1990 a 2017 foi mais pronunciada nos homens, sendo a porcentagem de diminuição 7,5 (II 95%, -10,2 a -4,8) para homens e 3,2 (II 95%, -6,5 a -0,1) para mulheres. Quanto aos grupos etários, as taxas de incidência aumentaram 10 vezes do grupo de 15-49 anos ao de 50-69 anos, e 6 vezes do último grupo ao de 70+ anos, tendo esses aumentos sido similares para mulheres e homens. De 1990 a 2017, a prevalência aumentou apenas no grupo de 15-49 anos, enquanto diminuiu nos demais, provavelmente em associação com a elevação de eventos isquêmicos naquele grupo etário.
- Uma revisão sistemática, avaliando a carga de insuficiência cardíaca na América Latina, incluiu 143 artigos publicados entre janeiro de 1994 e junho de 2014, com pelo menos 50 participantes com idade ≥ 18 anos; a maioria dos estudos incluídos (64%) foi do Brasil. ^167^ A idade média dos pacientes foi 60±9 anos, a fração de ejeção média, 36±9%, e a prevalência de insuficiência cardíaca, 1% (IC 95%, 0,1-2,7). Dos estudos incluídos, apenas um avaliou incidência, com 1.091 indivíduos identificados através de amostragem probabilística em múltiplas etapas na cidade de Porto Alegre. A idade média foi 42,8±16,9 anos e 55% eram mulheres. A incidência de insuficiência cardíaca em um estudo com apenas uma população fornecendo essa informação foi de 199 casos por 100 mil pessoas-ano. ^168^
- Em estudo de base populacional em atenção primária de uma cidade brasileira de tamanho médio, 633 indivíduos com idade ≥45 anos foram selecionados aleatoriamente e registrados em um programa de atenção primária. A idade média foi 59,6±10,4 anos e 62% eram mulheres. A prevalência de insuficiência cardíaca sintomática (estágio C) foi 9,3% e a de insuficiência cardíaca estágio B (anormalidades estruturais) foi 42,7%. Dos pacientes com insuficiência cardíaca, 59% apresentavam fração de ejeção preservada e 41% apresentavam fração de ejeção reduzida. ^169^
- Outro estudo de base populacional com residentes da Zona da Mata, Minas Gerais, envolveu 7.113 idosos frágeis. A idade média foi 72,4 ± 8,0 anos, 67,6% eram mulheres e a prevalência de insuficiência cardíaca foi 7,9%. ^170^
- Em estudo que incluiu 166 pacientes da área rural de Valença, Rio de Janeiro, a idade média foi 61 *±14 anos e 51% eram homens. As principais etiologias foram hipertensiva e isquêmica, sendo 51% portadores de* insuficiência cardíaca com fração de ejeção reduzida *, com características similares àquelas de coortes de centros terciários não rurais.* ^171^

#### Mortalidade

- Em estudo avaliando dados do SIM de 2008 a 2012, insuficiência cardíaca foi um código *garbage* usado com frequência no Brasil. Foi listada como causa básica de morte em 123.268 (3,7%) daqueles registros e como causa múltipla de morte em 233.197 (7%). Utilizando 2 métodos de redistribuição para causas específicas de morte, apenas 38,7-44,8% puderam ser reclassificadas para uma causa definida de morte com o diagnóstico principal, dependendo do método de reclassificação. ^172^ A insuficiência cardíaca não deve ser considerada uma causa básica de morte, mas constar da cadeia de eventos que levam à morte. Portanto, qualquer análise de dados do SIM que use insuficiência cardíaca como causa básica de morte a partir das declarações de óbito deve ser interpretada com cautela, pois pode estar estimando de maneira errada a verdadeira carga de insuficiência cardíaca.
- Dados obtidos da Fundação SEADE para mortalidade no estado de São Paulo em 2006 avaliaram 242.832 mortes em estimativa de 41.654.020 habitantes. ^153^ Insuficiência cardíaca e etiologias a ela associadas (exceto doença valvar primária) foram responsáveis por 6,3% do total de mortes. Para esses dados, não houve distribuição nem reclassificação das causas básicas de morte, tendo todas as etiologias associadas com insuficiência cardíaca sido incluídas ao se considerar o impacto da insuficiência cardíaca na mortalidade total.
- Um estudo sobre mortalidade por insuficiência cardíaca nos estados do Rio de Janeiro, São Paulo e Rio Grande do Sul incluiu dados de 2.960.857 declarações de óbito de 1999 a 2005. As porcentagens de morte por insuficiência cardíaca foram 3,0% no formato restrito (insuficiência cardíaca como causa básica de morte) e 9,0% no formato abrangente (insuficiência cardíaca mencionada em qualquer linha da declaração de óbito) em 1999. As porcentagens diminuíram com o tempo, passando para 2,4% e 8,6%, respectivamente, em 2005. As taxas de mortalidade decresceram na maioria dos grupos etários, exceto naquele a partir de 80 anos. As taxas aumentaram com a idade e foram claramente mais elevadas entre homens até os 80 anos de idade. ^173^
- Um estudo de coorte brasileiro mostrou dados de 1.220 pacientes ambulatoriais de uma clínica especializada em insuficiência cardíaca, acompanhados por 26±26 meses, de 1991 a 2000. Os pacientes encontravam-se em classe funcional III e IV, tinham idade média de 45,5±11 anos e 78% deles eram homens. As principais etiologias foram cardiomiopatia dilatada (37%), DCh (20%) e cardiomiopatia isquêmica (17%). Durante o período de acompanhamento, 415 (34%) pacientes morreram e 71 (6%) foram submetidos a transplante cardíaco. A DCh foi preditor de mau prognóstico. ^174^
- Dados mais recentes de 700 pacientes consecutivos com insuficiência cardíaca com fração de ejeção reduzida de uma clínica ambulatorial de um centro de saúde terciário em São Paulo mostraram mortalidade de 1 ano de 6,8% (47 pacientes). O desfecho composto de morte e hospitalização foi observado em 123 pacientes (17,7%) e 7 pacientes (1%) foram submetidos a transplante cardíaco. A idade média dos pacientes foi 55,4±12,2 anos e 67% eram homens. As principais etiologias foram cardiomiopatia hipertensiva (26,0%), isquêmica (21,9%) e chagásica (17,0%). Níveis séricos elevados de ureia e de peptídeo natriurético cerebral, assim como pressão arterial sistólica baixa, foram preditores independentes de mortalidade geral em 1 ano na amostra. ^175^
- Em estudo relatando dados do Banco Nacional de Marcapasso Multissítio, incluindo 3.526 pacientes de 2002 a 2007 atendidos no SUS, a idade média dos pacientes foi 59,8±13,3 anos e 66% eram homens. A sobrevida geral dos pacientes submetidos à terapia de ressincronização cardíaca no Brasil foi 80,1% (IC 95%, 79,4-80,8) em 1 ano e 55,6% (IC 95%, 54,6-56,6) em 5 anos, enquanto a mediana da sobrevida geral foi 30,3 meses (IIQ, 16,1-50,9). Observou-se ainda melhora da sobrevida na coorte estudada de 2002 a 2007 (p=0,055). ^176^

#### Hospitalizações

- As hospitalizações são a principal consequência de insuficiência cardíaca descompensada, resultando em pior prognóstico e elevando os custos. O Estudo BREATHE avaliou uma amostra de pacientes admitidos por insuficiência cardíaca descompensada aguda. No total, 1.263 pacientes foram incluídos de 51 centros de diferentes regiões brasileiras em 2011 e 2012. A mortalidade hospitalar foi 12,6% e os indicadores de qualidade assistencial baseados nas recomendações de alta hospitalar foram alcançados em menos de 65% dos pacientes. ^177^
- Outros estudos sobre taxa de mortalidade, anteriores ao estudo BREATHE, ^178 - 180^ mostraram taxas de mortalidade hospitalar similares, variando de 9% a 17%. ^179^
- Em uma comparação de pacientes com insuficiência cardíaca descompensada entre hospitais universitários terciários no Brasil e nos Estados Unidos, os pacientes dos Estados Unidos eram mais velhos (p < 0,01) e apresentaram maior prevalência da etiologia isquêmica (p < 0,01). A permanência hospitalar foi significativamente mais curta (5 [IIQ, 3-9] vs. 11 [6-19] dias; p < 0,001) e a mortalidade hospitalar, menor (2,4% vs. 13%; p < 0,001) na coorte dos Estados Unidos, mas menos eventos clínicos nos 3 meses que se seguiram à alta foram observados nos pacientes brasileiros (42% vs. 54%; p = 0,02). Esse estudo ressalta a importância de se melhorar o conhecimento sobre insuficiência cardíaca em pacientes brasileiros para que se melhorem a assistência e os desfechos. ^181^
- Na revisão sistemática citada anteriormente, que avalia a carga de insuficiência cardíaca na América Latina, com 64% dos estudos incluídos provenientes do Brasil, ^167^ as taxas de hospitalização foram 33%, 28%, 31% e 35% em acompanhamentos de 3, 6, 12 e 24 a 60 meses, respectivamente. A mediana de permanência hospitalar foi 7,0 [IIQ, 5,20-11,00] dias. A mortalidade hospitalar foi 11,7% (IC 95%, 10,4%-13,0%), sendo as taxas maiores nos pacientes com fração de ejeção reduzida, doença isquêmica do coração ou DCh. A taxa de mortalidade em 1 ano foi 24,5% (IC 95%, 19,4-30,0).
- A partir de dados do SUS, foram descritos os números de hospitalizações e mortes por insuficiência cardíaca em São Paulo de 1992 a 2010. A taxa de mortalidade hospitalar por insuficiência cardíaca foi 15%. Ao se compararem os períodos de 1992-1993 e 2008-2009, houve diminuição de 32% no número de hospitalizações por insuficiência cardíaca (p = 0,002), aumento de 15% na mortalidade (p = 0,004) e aumento na permanência hospitalar por insuficiência cardíaca de 8,8 para 11,3 dias (p = 0,001). ^182^
- Em 2019, um estudo mais recente com dados do DATASUS avaliou as admissões por insuficiência cardíaca no Brasil no período de 2007 a 2016, comparando-as com as do Rio Grande do Sul e as de Porto Alegre, uma cidade com vários centros de referência. Como ilustra a Figura 4-10, o estudo mostrou declínio nas taxas de mortalidade hospitalar de 2007 a 2016 no Brasil (19% de redução) e no Rio Grande do Sul (25% de redução), e declínio ainda mais pronunciado em Porto Alegre (65%). ^183^

#### Carga de Doença

- De acordo com estimativas do GBD 2017 ( Tabela 4-13 ), as taxas de YLDs padronizadas por idade por insuficiência cardíaca foram 112 (II 95%, 83-141) em 1990 e 109 (II 95%, 81-134) em 2017 por 100 mil habitantes, correspondendo a diminuição de 3% (II 95%, -6,7 a 0,3). Tais variações são similares às observadas nas taxas de prevalência de insuficiência cardíaca. A despeito dessa diminuição nas taxas de YLDs, a insuficiência cardíaca resultou em 88.114 (II 95%, 64.078-112.624) DALYs no Brasil em 1990 e em 234.169 (II 95%, 174.338-291.188) DALYs em 2017, devido ao crescimento e envelhecimento da população.
- A Tabela 4-14 mostra as taxas de YLDs por insuficiência cardíaca de acordo com o sexo e os grupos etários, a partir de estimativas do Estudo GBD 2017. As taxas de YLDs padronizadas por idade foram similares para mulheres e homens em 1990, mas as de 2017 foram 105 (II 95%, 82-127) para homens e 111 (II 95%, 80-141) para mulheres, devido a redução de 6,8% (II 95%, -10,9 a -2,6) para homens e quase nenhuma redução para mulheres (-0,3%, IC 95%, -4,9 a 4,2). Como esperado, as mais altas taxas de YLDs foram observadas no grupo de 70+ anos, seguido pelo grupo de 50-69 anos. À semelhança das variações observadas na prevalência, de 1990 a 2017, os maiores aumentos de YLDs foram verificados no grupo de 15-49 anos.

### Utilização e Custo da Atenção à Saúde

#### (Ver Tabelas 1-6 a 1-9 e Figuras 1-15 a 1-16)

- De acordo com dados do SUS, houve 2.862.739 hospitalizações por insuficiência cardíaca de 2008 a 2018. Esse número representa um terço do total de hospitalizações clínicas relacionadas às condições cardiovasculares no período estudado. Os custos não ajustados foram R$ 3.597.824.618. Em dólares internacionais, valores convertidos para paridade do poder de compra e ajustados para US$ 2018, os custos foram Int$ 866.945.691.
- No período observado, houve redução no número de hospitalizações clínicas por insuficiência cardíaca, que passaram de 298.474 (157 por 100 mil) em 2008 para 222.394 (107 por 100 mil) em 2018, sendo a redução uniforme ao longo dos anos. A despeito dessa redução no número de admissões, os gastos em saúde não ajustados estimados a partir do pagamento direto por assistência a pacientes com insuficiência cardíaca aumentaram de 2008 para 2018 em quase 28%, passando de R$ 272.280.662 (2018 Int$ 65.609.798) em 2008 para R$ 348.832.330 (2018 Int$ 172.008.052) em 2018. O número decrescente de hospitalizações e o gasto crescente representam maiores custos por admissão no período observado (R$ 912 em 2008 para R$ 1.568 em 2018). Insuficiência cardíaca foi responsável pela maioria dos custos relacionados às hospitalizações clínicas por doenças cardiovasculares.
- A carga econômica de insuficiência cardíaca no Brasil foi avaliada usando-se o custo padrão da estrutura da doença para considerar os custos em 2015. Foram analisados a prevalência e os gastos associados ao tratamento, à perda de produtividade por redução do emprego, aos custos da provisão de cuidado formal e informal e à perda de bem-estar. O estudo relatou que a insuficiência cardíaca determina um custo financeiro de R$ 22,1 bilhões/US$ 6,8 bilhões, o segundo dentre as quatro principais condições cardíacas no Brasil: infarto do miocárdio, insuficiência cardíaca, hipertensão e fibrilação atrial. ^184^
- Em estudo utilizando dados do DATASUS sobre admissões por insuficiência cardíaca em 10 anos, Nicolao *et al* . ^183^ mostraram um aumento de 97% no custo médio por paciente das hospitalizações relacionadas com insuficiência cardíaca de 2007 a 2016. Dados de Porto Alegre, uma cidade com vários hospitais de referência, mostraram um aumento ainda mais pronunciado (135%), como também uma diminuição mais pronunciada na mortalidade, em comparação aos dados do Brasil (ver acima).

####  Transplante Cardíaco Aberto e Implantação de Dispositivo de Assistência Ventricular

- O número de transplantes cardíacos realizados no Brasil aumentou de 149 em 2006 para 357 em 2016 e, embora esse aumento tenha sido significativo naquele período, representa cerca de um quinto da necessidade estimada da população. A sobrevida de 1 ano foi 73% (dados de sobrevida de 2010). ^185^
- Uma análise de custo de transplante cardíaco no Brasil, com todos os receptores consecutivos de transplante cardíaco em um único centro de julho de 2015 a junho de 2017, mostrou, para os 27 pacientes incluídos, uma média de custo total de US$ 74.341, que é mais baixa do que as relatadas para países desenvolvidos, mas excede em 60% o valor de reembolso por paciente. ^186^
- Em estudo descritivo de hospital público de referência em cardiologia localizado em Fortaleza, 16 pacientes foram submetidos a implantação de dispositivo de assistência ventricular de 2008 a 2015. A idade média foi 40,1±3,4 anos e 87,5% eram homens. Cardiopatia chagásica foi a principal etiologia (37,5%). Todos os pacientes apresentaram complicações durante o uso do dispositivo, sendo sangramento a mais frequente [11 (68,8%)]. Quanto ao desfecho clínico, 10 pacientes (62,5%) foram submetidos a transplante cardíaco e 5 (31,3%) morreram. ^187^

### Pesquisa Futura

- Por ser a insuficiência cardíaca considerada um código *garbage* quando designada como causa básica de morte, são necessários estudos que investiguem o melhor método para reclassificar e redistribuir essa causa de modo a reduzir viés e propiciar melhor comparabilidade de dados para o aperfeiçoamento das políticas de saúde.
- Estudos brasileiros de coorte sobre cardiomiopatias são raros, tendo alguns estudos clínicos no Brasil informado dados de insuficiência cardíaca, havendo, no entanto, poucos estudos multicêntricos com dados da população brasileira. Vale ressaltar a importância de se poder contar com dados tanto de insuficiência cardíaca quanto de cardiomiopatia, assim como de pacientes ambulatoriais e hospitalizados, além de se compreender de maneira ampla a carga crescente da insuficiência cardíaca nas doenças cardiovasculares. São necessários mais estudos multicêntricos de larga escala para melhor descrever a carga, os desfechos e os custos da insuficiência cardíaca na população brasileira.
- Além disso, estudos que explorem a qualidade e os custos da assistência na insuficiência cardíaca auxiliariam no desenvolvimento de políticas de saúde para melhorar a conscientização, o acesso a intervenções que salvam vidas, a doação de órgãos, assim como o uso de recursos nesta doença tão complexa.
- Embora as taxas de mortalidade por DCh tenham diminuído substancialmente nas últimas décadas, a DCh permanece uma importante causa de morte no Brasil. Há, na verdade, evidência de que a DCh seja uma causa de morte subnotificada, assim como, provavelmente, de hospitalização. São necessários mais dados sobre taxas de hospitalização e desfechos de pacientes com CMCh.

**Tabela 4-1:** – Taxa de prevalência padronizada por idade de cardiomiopatia e miocardite, por 100 mil habitantes, e variação percentual das taxas, para ambos os sexos e para homens e mulheres, no Brasil e suas unidades federativas, 1990 e 2017

	Ambos os sexos	Mulheres					Homens		
	1990	2017	Variação percentual (II 95%)	1990	2017	Variação percentual (II 95%)	1990	2017	Variação percentual (II 95%)
Brasil	102.8 (82.5;125.7)	112.4 (92.2;134.2)	9.4 (15.3;4.1)	107.7 (86.2;131.2)	115 (95;137.4)	6.9 (14.2;0.2)	97.1 (77.5;119)	109 (88.3;131.7)	12.3 (20;5.4)
Acre	61 (49.2;75.6)	65.9 (53.2;81.1)	8 (21.6;-4.5)	63.8 (50.7;79.9)	66.3 (51.7;81.8)	3.9 (26.6;-13.3)	58.6 (46;74.4)	65.5 (51.5;82.3)	11.8 (32.5;-5.6)
Alagoas	84 (65.4;105.2)	103.3 (82.9;126.8)	23.1 (38.9;8.9)	86.6 (67.4;109.4)	101.4 (80.5;125.5)	17.1 (37.4;-0.5)	80.8 (60.5;104)	105.7 (82.7;133.3)	30.8 (56.3;9.4)
Amapá	104.9 (84.8;128.4)	106.4 (86.4;130.8)	1.4 (13.8;-9.1)	102.7 (83.2;126.6)	103.6 (82.8;128.4)	0.9 (19.5;-15)	107.1 (84.7;133.7)	108.9 (86.4;135.3)	1.6 (19.9;-12.4)
Amazonas	95.3 (76.7;117.9)	98.7 (78.8;120.6)	3.5 (15.3;-7.7)	94.3 (75.1;116.1)	95.7 (75.9;118)	1.5 (17.7;-13.2)	96.1 (74.8;120.2)	101.6 (79.7;126.8)	5.7 (24.6;-10.1)
Bahia	67.2 (51.5;84.9)	74.1 (58.9;91.2)	10.4 (25.4;-2.3)	70.4 (53.8;89.7)	73.8 (58.2;91.7)	4.7 (24.1;-11.1)	63.6 (47.9;82.5)	74.7 (58.6;95.4)	17.5 (41.8;-1.9)
Ceará	105.6 (83.9;128.9)	130.2 (105.9;157.5)	23.2 (37.7;10)	96.8 (76.9;119.8)	116.9 (92.7;142.9)	20.7 (41.8;2.5)	115.4 (90.2;145.4)	146.4 (117.2;181.7)	26.8 (49;9.5)
Distrito Federal	125.6 (92.7;161.3)	144 (113;179.2)	14.6 (32.7;1.3)	134.7 (98.8;171.7)	148.5 (116.4;183.6)	10.2 (33.1;-5.9)	116 (82.6;156.4)	138.9 (104.8;177.4)	19.8 (48.5;-0.3)
Espírito Santo	104.2 (83.5;127.5)	104 (84.4;126.2)	-0.1 (10.8;-11.7	95.8 (76.3;120.3)	94.2 (75.1;115.8)	-1.7 (15;-15.7)	112.9 (88.7;139.5)	115.2 (91.7;143)	2 (18.3;-11.9)
Goiás	99.1 (71.5;130.5)	125.9 (97.2;160.3)	27.1 (49.3;10.8)	116.1 (81;154.9)	141.2 (109.2;179.2)	21.6 (51.5;1.9)	83 (58.9;111.8)	108.7 (82.5;142.4)	31 (63.1;8.5)
Maranhão	61.3 (48.3;75.9)	76.7 (61.9;94.2)	25.2 (41.6;10.8)	58.7 (46;73.9)	69.1 (54.3;86.4)	17.6 (41.1;-1.4)	63.8 (48.5;81.1)	85.1 (67.2;106.1)	33.5 (60.4;12.1)
Mato Grosso	92.9 (74;115.1)	100.5 (80.4;123.5)	8.2 (21.7;-3.1)	100.3 (79.3;124.1)	106.4 (84.1;132.4)	6.1 (22.8;-8.4)	86.3 (65.6;109.5)	94.5 (73.9;118.4)	9.6 (30.4;-7.3)
Mato Grosso do Sul	126.2 (100.6;155.9)	141.5 (113.6;172.9)	12.1 (26.2;0.5)	131.9 (105;162.7)	140.3 (113;174)	6.4 (23.2;-9.8)	120.5 (91.5;154.7)	142.4 (112.8;175.3)	18.1 (40.9;0.6)
Minas Gerais	110.7 (85.9;139.6)	123.7 (98;152)	11.7 (27.3;-0.9)	123.3 (95.2;155.8)	132.4 (105.3;163.8)	7.3 (28;-7.5)	95.8 (70.1;124.4)	113 (87.8;142.4)	17.9 (42.6;-0.7)
Pará	72.9 (59;88.7)	74.8 (60.4;92.1)	2.6 (15.9;-9.4)	73.1 (57.6;90.4)	72.6 (58.3;91.3)	-0.7 (16.4;-16.5	72.8 (57.8;90.9)	77.1 (60.5;95.1)	5.8 (25.4;-10.7)
Paraíba	116.2 (92.8;142.4)	138.3 (112.3;168.2)	19 (34.8;7)	118.5 (94.5;145.7)	140.4 (112.6;172.9)	18.5 (39.6;1.2)	113.5 (89.8;142.8)	135.7 (107.6;167.8)	19.6 (40.3;1.7)
Paraná	90.4 (71.4;111.4)	97 (77.5;118.8)	7.3 (21.1;-4.3)	95.8 (74.8;119.6)	99.5 (79.5;123)	3.8 (21.9;-11.1)	84.7 (64.6;106.9)	93.7 (73.7;117)	10.7 (32.5;-7.3)
Pernambuco	81.8 (64.8;101.5)	85.3 (68.5;104)	4.3 (17.8;-7.4)	80.5 (62.3;100.8)	81.9 (64.1;102.3)	1.8 (20.4;-14.8)	83.6 (64.2;106)	89.9 (70.6;112.4)	7.6 (29.5;-9.2)
Piauí	64.6 (50.1;80.1)	75.4 (60.2;92.7)	16.8 (33.1;3.5)	65.9 (51.4;82.2)	74.5 (58.7;92.7)	13.1 (34.9;-3.7)	63.1 (47.5;81.5)	76.4 (59.5;96.4)	21 (45.6;0.3)
Rio de Janeiro	117.3 (94.5;144.3)	113.9 (92.7;138.1)	-2.9 (8.7;-12.7)	115.9 (92.9;145)	112.8 (91.5;137.7)	-2.7 (12.4;-15.7	119.3 (93;149)	115.6 (91.9;142.1)	-3.1 (13.9;-16.3)
Rio Grande do Norte	75.9 (60.8;95.4)	83.6 (67.4;102.5)	10.1 (23.8;-1.8)	68 (53.4;85.4)	74.7 (59.2;93.7)	9.8 (30.5;-6.9)	84.6 (67.8;107.3)	94.9 (75.6;116.7)	12.1 (32;-4.2)
Rio Grande do Sul	75.2 (59.7;93.4)	74.7 (60;91.3)	-0.7 (12.1;-11.7	73.1 (57.3;92.8)	72.1 (57.7;89.4)	-1.4 (17.2;-16.8	77 (60;96.4)	76.9 (60.5;95.5)	0 (18.5;-15.9)
Rondônia	69.9 (54.3;87.9)	76.3 (62;94.4)	9.2 (25.4;-3.8)	74.1 (57.7;94.9)	78.7 (63.2;98.4)	6.1 (25.9;-10.5)	66.2 (49.8;84.5)	74 (57.8;92.9)	11.7 (34.3;-7)
Roraima	141.5 (114.4;172.9)	145 (118.1;174.6)	2.5 (14.5;-7.8)	137.9 (111;168.4)	140.3 (111.4;171.8)	1.7 (18.3;-12.1)	143.8 (113.8;177.9)	149.2 (118.5;183.1)	3.7 (21.1;-10.2)
Santa Catarina	107.2 (86.6;131.2)	106.7 (86.6;130)	-0.5 (10.2;-10.9	109.7 (88.8;135.7)	108.4 (86.2;133.7)	-1.2 (14;-15.3)	103.5 (81.8;129)	103.6 (82.4;127.7)	0.1 (17.4;-14.5)
São Paulo	138.4 (108.2;171.2)	147.7 (120.7;178)	6.7 (21.3;-4.4)	152.1 (118.7;187.1)	158 (128;193.1)	3.8 (21.1;-10.7)	121.3 (92.5;156)	134.1 (105.8;164.7)	10.5 (29;-6.6)
Sergipe	100.2 (80.6;123.9)	115.3 (93.9;141.5)	15.2 (29.1;3.3)	102.5 (81.7;128.8)	115 (92.6;141.2)	12.2 (31.1;-3.9)	97.3 (75.4;120.9)	115.2 (91.3;142.9)	18.4 (39.4;2.2)
Tocantins	71.3 (53.8;89.6)	88.8 (70.6;109.2)	24.6 (43.3;9.2)	71.1 (54.4;91.9)	85.2 (67.3;106.3)	19.9 (45.4;0.7)	71.6 (53;92.1)	92.3 (71.6;116.4)	28.9 (59;7.9)

* Fonte: Estudo Global Burden of Disease 2017, Institute for Health Metrics and Evaluation. ^188^*

**Tabela 4-2:** – Número de casos incidentes e taxa de incidência (por 100 mil) padronizada por idade de cardiomiopatia e miocardite, e variação percentual das taxas, no Brasil e suas unidades federativas, 1990 e 2017

	1990		2017		
	Número (II 95%)	Taxa (II 95%)	Número (II 95%)	Taxa (II 95%)	
Brasil	54520.4 (48574.3;61320.7)	46.3 (41.5;52.1)	103879.4 (92495.6;117294.5)	46.7 (41.8;52.6)	0.8 (-0.3;1.8)
Acre	125.8 (111;143)	44.8 (40;50.7)	340.7 (303.3;383.1)	46 (41.1;52)	2.8 (-0.5;6.4)
Alagoas	826.5 (732.4;929.4)	43.6 (38.8;49.3)	1551.4 (1378.8;1749)	46.5 (41.5;52.4)	6.8 (3.5;10.2)
Amapá	85.4 (75.2;97.2)	47.5 (42.5;53.4)	311.1 (274.3;352.6)	47.6 (42.5;53.6)	0.3 (-2.8;3.9)
Amazonas	645.3 (565.6;732.6)	46.4 (41.5;52.6)	1586.5 (1410.7;1793.3)	46.6 (41.6;52.8)	0.5 (-2.9;4.3)
Bahia	4183 (3713.7;4708.9)	45.3 (40.3;50.9)	7177 (6366.6;8059.8)	45.1 (40;50.7)	-0.3 (-3.4;2.7)
Ceará	2232.8 (1990.7;2511.3)	43.2 (38.7;48.7)	4589.2 (4084.6;5197.2)	46.4 (41.4;52.7)	7.5 (3.4;11)
Distrito Federal	502.7 (439.4;574.4)	45.2 (40.3;51.3)	1258.3 (1107.5;1428.3)	45.3 (40.4;51)	0.2 (-3.3;3.7)
Espírito Santo	976.1 (856.5;1104.3)	48.1 (42.8;54.4)	1992.5 (1757;2263.2)	48.2 (42.9;54.4)	0.2 (-3.2;3.8)
Goiás	1391.1 (1220.9;1574.2)	46.3 (41.4;52.2)	3233.1 (2869.8;3671.6)	47.3 (42.2;53.5)	2.3 (-0.8;5.4)
Maranhão	1568.5 (1390.4;1771.6)	43.3 (38.6;48.9)	3314.6 (2958.6;3716.8)	46.7 (41.7;52.6)	8 (4.5;12)
Mato Grosso	628.1 (549.8;715.5)	45.6 (40.7;51.7)	1567.2 (1394.8;1780.2)	46.8 (41.9;52.9)	2.7 (-1;6.3)
Mato Grosso do Sul	648.6 (572.4;728)	48.6 (43.4;54.8)	1426.5 (1264.1;1616.6)	50.6 (45.2;57)	4.1 (0.8;7.7)
Minas Gerais	5880.7 (5207.1;6646.3)	46 (41;52)	11181 (10003.5;12646.6)	47 (42.2;53.2)	2.2 (-1;5.5)
Pará	1548.2 (1365.8;1749.4)	45.4 (40.6;51.3)	3561 (3155.4;4003.7)	45.5 (40.5;51.4)	0.2 (-3.2;3.9)
Paraíba	1181.3 (1051.4;1331.4)	43.2 (38.6;48.9)	2068.4 (1841.2;2347.2)	46.3 (41.2;52.5)	7.1 (3.6;10.5)
Paraná	3140.9 (2777.5;3572.6)	47.1 (41.9;53.3)	5728 (5060.1;6535.4)	47 (41.9;53.2)	-0.2 (-3.8;3)
Pernambuco	2639 (2333.6;2961.8)	45.6 (40.5;51.3)	4609.7 (4098.5;5160.5)	46.1 (41.1;51.6)	1.1 (-2.4;4.5)
Piauí	865.4 (765.7;974.5)	43.8 (39;49.4)	1652.7 (1471;1863.8)	45.8 (40.7;51.6)	4.5 (0.8;8.3)
Rio de Janeiro	5321.9 (4706.8;6019.7)	46.7 (41.6;52.5)	9236.1 (8183.4;10524.9)	46.4 (41.3;52.5)	-0.5 (-4.1;3.1)
Rio Grande do Norte	886.2 (790.4;995.2)	44.3 (39.7;49.8)	1723.7 (1535.1;1947.9)	46.2 (41.2;52.1)	4.2 (1;7.7)
Rio Grande do Sul	3927.6 (3482.4;4424.5)	49.5 (44.1;55.8)	6475.7 (5749;7325.7)	48.5 (43.2;54.5)	-2.1 (-5.5;1.4)
Rondônia	330.5 (289.3;379.1)	45.6 (40.6;51.4)	746.3 (660.9;847.2)	46 (41.2;52.2)	0.9 (-2.6;4.4)
Roraima	61.2 (53.3;70.9)	46.1 (41.2;52)	212.3 (187.3;240.4)	46.4 (41.3;52.4)	0.9 (-2.4;4.2)
Santa Catarina	1694 (1503.4;1912.2)	47.8 (42.9;54.2)	3554.7 (3148.4;4033.5)	47.5 (42.4;53.4)	-0.7 (-4.2;3.5)
São Paulo	12421.8 (10980.5;13984.7)	47.6 (42.4;53.5)	22999.9 (20352.4;26154.9)	46.6 (41.6;52.7)	-1.9 (-5.5;1.4)
Sergipe	520.8 (463.6;584.1)	45.3 (40.5;51.4)	1075.4 (957.1;1218.6)	47.3 (42.1;53.4)	4.5 (1;7.8)
Tocantins	287 (253.9;325.8)	44.8 (40;50.5)	706.6 (629;798)	47.1 (42.1;53.1)	5.1 (1.6;8.9)

* Fonte: Estudo Global Burden of Disease 2017, Institute for Health Metrics and Evaluation. ^188^*

**Tabela 4-3:** Taxas de incidência de cardiomiopatia e miocardite por 100 mil habitantes e variação percentual das taxas, por idade e sexo, Brasil, 1990 e 2017

Grupo etário	1990	2017	Variação percentual (II 95%)
***Ambos os sexos***			
Padronizada por idade	46,3 (41,5;52,1)	46,7 (41,8;52,6)	0,8 (-0,3;1,8)
Abaixo de 5	11,2 (8,9;13,9)	11,3 (9;14,1)	1,1 (-0,8;3,5)
5-14 anos	16,5 (12;22,2)	16,7 (12,3;22,6)	1,1 (-1,4;3,6)
15-49 anos	32,8 (27,1;39)	34,2 (28,2;40,6)	4,2 (0,4;8,2)
50-69 anos	82,4 (67,1;102)	83,4 (67,5;103,2)	1,2 (-1;3,5)
70+ anos	203,1 (165,4;252,3)	214,8 (176,5;263,2)	5,8 (2,2;10,1)
Todas as idades	36,5 (32,5;41)	49 (43,7;55,4)	34,4 (29;39,9)
***Masculino***			
Padronizada por idade	46 (41,1;51,8)	45,5 (40,7;51,5)	-1 (-2,5;0,5)
Abaixo de 5	11,2 (8,8;13,8)	11 (8,7;13,7)	-1,3 (-4,1;2,5)
5-14 anos	16,5 (11,9;22,2)	16,2 (11,9;22)	-1,5 (-5,1;2,7)
15-49 anos	32,6 (26,9;38,7)	33,2 (27,4;39,5)	1,9 (-2,5;5,9)
50-69 anos	81,6 (65,9;100,9)	81,6 (66;101,9)	0 (-3,2;2,9)
70+ anos	198,9 (161,2;247,1)	205,6 (167,7;253,2)	3,4 (-0,4;7,7)
Todas as idades	35,4 (31,4;39,9)	45,7 (40,6;51,9)	29,1 (23,9;34,9)
***Feminino***			
Padronizada por idade	46,6 (41,6;52,4)	47,7 (42,7;53,8)	2,3 (0,8;3,8)
Abaixo de 5	11,3 (9;14)	11,7 (9,4;14,6)	3,7 (0,8;7,2)
5-14 anos	16,6 (12,2;22,2)	17,2 (12,7;23,1)	3,7 (0,3;7,1)
15-49 anos	33 (27,2;39,4)	35,2 (29;41,8)	6,4 (2,1;11)
50-69 anos	83,2 (67,6;103)	85,1 (69,1;105,6)	2,3 (-1;5,3)
70+ anos	206,5 (168,6;257,1)	221,6 (182,6;270,7)	7,3 (2,9;12,4)
Todas as idades	37,6 (33,4;42,3)	52,3 (46,6;59)	39,1 (33,3;45,3)

* Fonte: Estudo Global Burden of Disease 2017, Institute for Health Metrics and Evaluation, ^188^*

**Tabela 4-4:** – Número de mortes e taxa de mortalidade padronizada por idade (per 100 mil) por cardiomiopatia e miocardite, e variação percentual das taxas, no Brasil e suas unidades federativas, 1990 e 2017

Localização	1990		2017		Variação percentual (II 95%)
	Número (II 95%)	Taxa (II 95%)	Número (II 95%)	Taxa (II 95%)	
Brasil	9734 (8417;10163)	10.9 (9.6;11.4)	18812 (17885;21745)	8.6 (8.2;9.9)	-21.2 (-26.8;-2.6)
Acre	11 (10;15)	5.7 (5.2;7.9)	29 (25;45)	4.8 (4.1;7.6)	-15.2 (-28;26.1)
Alagoas	172 (137;194)	10.6 (8.4;11.9)	262 (239;289)	8.4 (7.7;9.3)	-20.7 (-32.3;1.9)
Amapá	7 (6;9)	6.7 (6;8.4)	32 (28;42)	6.7 (5.9;8.7)	-0.1 (-13.5;36)
Amazonas	55 (50;69)	6.3 (5.8;8)	135 (118;210)	4.9 (4.3;7.7)	-22.4 (-33.1;18.6)
Bahia	581 (503;639)	7.9 (6.6;8.8)	892 (800;1189)	5.6 (5;7.5)	-28.9 (-39.9;10.5)
Ceará	362 (296;425)	7.4 (5.9;8.7)	642 (592;704)	6.5 (6;7.1)	-11.8 (-27.5;15.6)
Distrito Federal	95 (71;103)	16.2 (12.5;17.5)	268 (221;299)	13.1 (10.8;14.5)	-19.4 (-28.2;-5)
Espírito Santo	138 (121;147)	10.5 (9.3;11.3)	261 (236;355)	6.4 (5.8;8.7)	-39.7 (-47.2;-13.5)
Goiás	345 (246;378)	20.1 (14.7;22.2)	730 (609;792)	11.8 (9.8;12.8)	-41.2 (-47.9;-24.8)
Maranhão	232 (193;286)	6.2 (5;8.1)	303 (268;458)	4.6 (4.1;7.1)	-25.7 (-41.7;3.6)
Mato Grosso	75 (68;83)	8.7 (7.8;9.7)	202 (182;277)	7.1 (6.3;9.5)	-18.5 (-29;14)
Mato Grosso do Sul	122 (89;133)	14.5 (10.6;15.9)	261 (236;293)	10 (9.1;11.2)	-30.8 (-39.9;2.9)
Minas Gerais	1238 (962;1334)	13.1 (10.3;14)	2059 (1903;2288)	8.4 (7.7;9.3)	-36 (-42.8;-16.7)
Pará	114 (102;162)	4.9 (4.5;7)	314 (274;482)	4.8 (4.2;7.3)	-3.6 (-15.2;29.3)
Paraíba	279 (220;318)	11.3 (8.9;12.9)	456 (392;515)	9.8 (8.4;11.1)	-12.9 (-27.5;8)
Paraná	567 (479;605)	13 (11;13.9)	769 (685;1168)	6.6 (5.9;9.9)	-49.2 (-56.2;-19.1)
Pernambuco	330 (306;367)	7.1 (6.6;8)	775 (700;884)	7.9 (7.1;9.1)	11.3 (-2.9;26.3)
Piaui	124 (102;146)	7.3 (5.9;8.9)	183 (165;233)	5.1 (4.6;6.5)	-30.7 (-43.4;3.1)
Rio de Janeiro	830 (749;1076)	8.9 (8.2;11.5)	2749 (2069;2994)	13.1 (10;14.2)	46.2 (12.6;63.3)
Rio Grande do Norte	92 (80;119)	5.1 (4.4;6.8)	176 (153;256)	4.6 (4;6.8)	-8.8 (-25.2;34.4)
Rio Grande do Sul	298 (268;487)	4.8 (4.3;7.8)	709 (617;1221)	4.9 (4.3;8.5)	2.7 (-7;14.1)
Rondônia	30 (27;36)	7.8 (7.1;9.3)	83 (70;120)	6.1 (5.2;8.8)	-22.6 (-34.2;4.1)
Roraima	10 (7;11)	17.1 (12.1;19.4)	40 (33;47)	13.5 (11.2;16.1)	-20.9 (-36.3;12.7)
São Paulo	3260 (2466;3519)	17.6 (13.5;19)	5766 (4987;6355)	11.8 (10.3;12.9)	-33.1 (-39.7;-16.4)
Santa Catarina	246 (229;264)	10.9 (10;11.8)	493 (449;681)	7 (6.3;9.5)	-36.3 (-43.8;-14.8)
Sergipe	82 (73;90)	8.7 (7.7;9.7)	139 (125;173)	6.5 (5.9;8.1)	-24.6 (-35.6;2)
Tocantins	41 (31;49)	10.2 (8.1;12.4)	87 (77;113)	6.3 (5.5;8.3)	-38.5 (-51.3;-5.2)

* Fonte: Estudo Global Burden of Disease 2017, Institute for Health Metrics and Evaluation. ^188^*

**Tabela 4-5:** – Taxa de mortalidade (por 100 mil habitantes) por cardiomiopatia e miocardite e variação percentual das taxas, por idade e sexo, Brasil, 1990-2017

	1990	2007	Variação percentual (II 95%)
***Ambos os sexos ***			
Padronizada por idade	10,9 (9,6;11,4)	8,6 (8,2;9,9)	-21,2 (-26,8;-2,6)
Abaixo de 5	4,8 (4;5,6)	2,5 (2,2;2,8)	-47,1 (-56,4;-31,1)
5-14 anos	0,4 (0,4;0,5)	0,4 (0,3;0,4)	-11,3 (-22,3;2,2)
15-49 anos	2,4 (2;2,6)	2,5 (2,2;2,7)	1,4 (-6,7;21,7)
50-69 anos	21,2 (17,7;22,5)	16,5 (15,3;19,3)	-22,4 (-29,3;-0,2)
70+ anos	84,2 (74,2;89,1)	76,6 (72,1;90)	-9 (-16,8;14,7)
Todas as idades	6,5 (5,6;6,8)	8,9 (8,4;10,3)	36,3 (26,6;68,8)
***Masculino ***			
Padronizada por idade	12,8 (10;13,7)	11,3 (10,5;14,4)	-12,1 (-20,9;28,2)
Abaixo de 5	4,8 (3,3;5,7)	2,7 (2,3;3,2)	-43 (-57,1;-3,5)
5-14 anos	0,4 (0,4;0,5)	0,4 (0,4;0,5)	-3,9 (-20;15,3)
15-49 anos	3,3 (2,5;3,6)	3,6 (3,2;4,2)	9,5 (-1;43,7)
50-69 anos	26,2 (19,1;28,4)	23,6 (21,4;29,7)	-10,1 (-20,9;31,7)
70+ anos	91,6 (69,7;99,7)	89,3 (81,2;119,5)	-2,5 (-15,1;53,7)
Todas as idades	7,4 (5,6;7,9)	10,7 (10;13,6)	44,8 (30,7;109,6)
***Feminino ***			
Padronizada por idade	9,2 (8,8;9,6)	6,3 (6;6,6)	-31,3 (-35,5;-26,2)
Abaixo de 5	4,7 (3,9;5,9)	2,3 (2;2,6)	-51,5 (-64,1;-37,5)
5-14 anos	0,4 (0,3;0,5)	0,3 (0,3;0,4)	-19,9 (-31,7;-4,4)
15-49 anos	1,6 (1,5;1,6)	1,3 (1,2;1,4)	-15,9 (-23,1;-7,1)
50-69 anos	16,6 (15,6;17,6)	10,2 (9,5;11)	-38,6 (-43,7;-32,3)
70+ anos	78,3 (73,7;83,3)	67,4 (63,2;71,9)	-13,9 (-21,1;-5,9)
Todas as idades	5,7 (5,4;5,9)	7,1 (6,8;7,5)	26,3 (18,6;35,3)

* Fonte: Estudo Global Burden of Disease 2017, Institute for Health Metrics and Evaluation. ^188^*

**Tabela 4-6:** – Número de DALYs e taxa de DALYs padronizada por idade (por 100 mil) por cardiomiopatia e miocardite, e variação percentual das taxas, no Brasil e suas unidades federativas, 1990 e 2017

Localização	1990		2017		Variação percentual (II 95%)
	Número (II 95%)	Taxa (II 95%)	Número (II 95%)	Taxa (II 95%)	
Brasil	328636.2 (283325;346745.9)	286.3 (246.7;300.6)	490571.8 (465903.3;556885.7)	222.3 (211.1;250.9)	-22.4 (-27.6;-7)
Acre	497.4 (436.2;639.6)	160.9 (145;211.8)	892.9 (770.2;1353.6)	128.3 (110.6;194.8)	-20.3 (-32.5;10.3)
Alagoas	7445.3 (5810.9;8943.2)	325.1 (260.1;373.1)	7889.4 (7045.9;8603.6)	238.4 (215;260)	-26.7 (-37.3;-12.9)
Amapá	260.3 (235.6;324.2)	162.7 (147.6;202.1)	1030.8 (926.3;1349.3)	172.1 (154.9;223)	5.8 (-6.7;41)
Amazonas	2178.8 (1943.2;2658.8)	162.5 (148;203.1)	4500.2 (3953.1;6675.2)	137.2 (120.4;205.4)	-15.6 (-25.7;19.1)
Bahia	19212.5 (17344.2;21147.3)	212.7 (188;232.3)	27181.1 (24338.5;33355.6)	175.2 (156.2;214.1)	-17.6 (-29.2;10.8)
Ceará	16413.7 (13000.4;20157.3)	260.6 (210.1;309.8)	18369.4 (16682.7;20144.2)	188.2 (170.6;206.9)	-27.8 (-41.3;-10.1)
Distrito Federal	3658.3 (2766.2;3988)	376.3 (286.8;405.1)	7351.8 (6159.1;8113.1)	276.6 (233.7;305.7)	-26.5 (-33;-14.2)
Espírito Santo	4540.9 (4041.7;4871.1)	241 (213.7;258.1)	7341.7 (6712.8;9314.8)	177 (161.6;223.8)	-26.5 (-34.9;-2.3)
Goiás	11003.3 (8024.8;12004.6)	428.7 (312.4;467.9)	18299.6 (15675.5;19752.8)	272.9 (234.7;293.9)	-36.3 (-43;-16)
Maranhão	13001.5 (10067.9;16807.4)	242.9 (200.7;299.4)	10003.6 (8755.1;13529.3)	137.8 (121.5;190.6)	-43.3 (-56.7;-23.3)
Mato Grosso	3073.7 (2771.1;3438.3)	229.1 (209.1;254.1)	5796.7 (5242.4;7786.8)	177.7 (161.1;236.5)	-22.4 (-31.3;2.3)
Mato Grosso do Sul	3865.2 (2943.9;4262.8)	329.7 (247;361.6)	6710.7 (6101.4;7492.7)	242 (221.4;268.6)	-26.6 (-35.7;6.5)
Minas Gerais	39564.4 (30507.2;42826)	327.1 (253.6;352.7)	51182.3 (47035.5;56511.4)	216.8 (198.6;238.8)	-33.7 (-40.5;-13.2)
Pará	4344.5 (3801.9;5889.4)	129.8 (116.4;180.3)	9977.8 (8660.1;14959.6)	132.8 (115.4;198.5)	2.3 (-9.4;25.4)
Paraíba	10241.9 (8039.5;12095.6)	341.3 (272.3;396.2)	11470.6 (9717.3;12811.3)	257.1 (217.9;287.7)	-24.7 (-36.3;-9.2)
Paraná	17321.6 (15163;18438.9)	292.2 (250.6;309.9)	19045.2 (16983.3;28587.7)	160.6 (142.8;234.4)	-45 (-52.3;-14.1)
Pernambuco	11872 (10716.4;13371.5)	200.8 (184.9;220.9)	21914.4 (19857.6;24316.1)	218.9 (198.9;243.3)	9 (-3.7;22.2)
Piauí	5486.8 (4473.4;6582.1)	227.8 (188.3;269.8)	5211.8 (4701.4;6542.5)	145.4 (130.7;182.9)	-36.2 (-48;-13)
Rio de Janeiro	28335.5 (24866.7;35902.2)	249.5 (221.8;316.3)	68879.7 (50970.5;75152.9)	334.9 (253.6;363.3)	34.2 (2.5;48.6)
Rio Grande do Norte	3445.5 (2978.7;4084.9)	153.8 (134.2;188.6)	5076.1 (4420.6;6901.5)	137.6 (119.9;186.8)	-10.6 (-24.8;18.6)
Rio Grande do Sul	9946.1 (8932.6;15081.7)	128.4 (115.3;197.2)	16836.2 (14705.4;28348.6)	124.5 (109.3;204.3)	-3.1 (-11.8;8.7)
Rondônia	1298.7 (1165.9;1533.3)	197.3 (178.5;234.3)	2395.2 (2053.6;3399.5)	153.1 (132.1;214.9)	-22.4 (-33.3;0.6)
Roraima	435.5 (314.2;500.3)	376.2 (264.9;423.8)	1242.6 (1054;1445.5)	296.9 (253.9;344.2)	-21.1 (-35.7;10)
Santa Catarina	7573.9 (7109.4;8246.8)	244.7 (229.3;262.6)	12498.4 (11367.3;16891.4)	168.5 (153.3;225.2)	-31.2 (-38.2;-12.2)
São Paulo	98920.7 (75875.1;106392.2)	410.9 (316.9;441.8)	142836.1 (123464.2;155246.1)	288.1 (253.2;311.2)	-29.9 (-36;-15)
Sergipe	2898.6 (2556.6;3238.8)	237.8 (211.1;262.1)	4042.2 (3652.6;4946.7)	180.8 (163.1;219.5)	-24 (-34.8;-0.4)
Tocantins	1799.9 (1350.9;2222.4)	255.1 (199.3;307.4)	2595.3 (2275.4;3210.4)	174.4 (152.9;216.8)	-31.6 (-45.1;-1.4)

* Fonte: Estudo Global Burden of Disease 2017, Institute for Health Metrics and Evaluation. ^188^*

**Tabela 4-7:** – Taxas de DALYs por cardiomiopatia e miocardite, por 100 mil habitantes, e variação percentual das taxas, por idade e sexo, Brasil, 1990 e 2017

Grupo etário	1990	2017	Variação percentual (II 95%)
***Todos ***			
Padronizada por idade	286,3 (246,7;300,6)	222,3 (211,1;250,9)	-22,4 (-27,6;-7)
Abaixo de 5	414 (345,2;487)	219,9 (195,5;246,4)	-46,9 (-56,3;-30,9)
5-14 anos	35,3 (29,9;39,3)	31,3 (28,2;34,5)	-11,3 (-21,8;1,5)
15-49 anos	126,4 (107,3;133,9)	125,6 (115,7;139,5)	-0,7 (-8,1;19,3)
50-69 anos	624 (522,3;660,5)	492,5 (458,8;569,8)	-21,1 (-27,9;-0,2)
70+ anos	1165 (1027,8;1237,6)	957,2 (888,7;1110,9)	-17,8 (-24,6;2,9)
Todas as idades	219,9 (189,6;232,1)	231,6 (220;262,9)	5,3 (-2;24,8)
***Masculino***			
Padronizada por idade	343,1 (262,8;365,7)	299 (279,9;363,4)	-12,8 (-21;17,6)
Abaixo de 5	419,5 (287,2;498,1)	239,9 (202,9;283,3)	-42,8 (-57;-3,3)
5-14 anos	36,9 (30,7;41,7)	35,3 (30,6;41,1)	-4,5 (-19,7;13,8)
15-49 anos	172,3 (132,9;185,4)	184,3 (164,1;213)	7 (-3,2;40,7)
50-69 anos	769,4 (565,3;832,5)	699,1 (634,3;864)	-9,1 (-19,9;30,9)
70+ anos	1293,3 (990,1;1415,9)	1165 (1058,4;1512,1)	-9,9 (-21,7;39,8)
Todas as idades	258,5 (198,1;276,9)	302,3 (282,1;367,3)	16,9 (5,5;55,7)
***Feminino***			
Padronizada por idade	233,8 (222,7;247,3)	153,5 (145,5;161,5)	-34,3 (-38,6;-30)
Abaixo de 5	408,3 (336,9;516,1)	199 (171,3;225)	-51,3 (-63,9;-37,2)
5-14 anos	33,6 (28,6;37,5)	27,2 (23,5;30,4)	-19,1 (-30,3;-4,4)
15-49 anos	82 (77,2;87)	68,4 (64,2;73)	-16,6 (-23,2;-8,8)
50-69 anos	489,8 (460,6;520)	309,6 (288,2;332,4)	-36,8 (-41,7;-30,8)
70+ anos	1061,9 (993,3;1138,6)	806,3 (749,9;868,8)	-24,1 (-30,4;-17,1)
Todas as idades	182,2 (172,3;194,9)	164 (155,3;172,6)	-10 (-16,9;-3,3)

* Fonte: Estudo Global Burden of Disease 2017, Institute for Health Metrics and Evaluation. ^188^*

**Tabela 4-8:** – Número de mortes e taxa de mortalidade padronizada por idade por 100 mil habitantes por doença de Chagas, em 1990 e 2017, e variação percentual das taxas, no Brasil e suas unidades federativas

Localização	1990		2017		Variação percentual (II 95%)
	Número de mortes (II 95%)	Taxa de mortalidade (II 95%)	Número de mortes (II 95%)	Taxa de mortalidade (II 95%)	
Acre	1.8 (1.6;1.9)	0.9 (0.9;1)	2.1 (1.8;2.4)	0.3 (0.3;0.4)	-63.9 (-69.7;-56.2)
Alagoas	68.4 (54.3;78.8)	4.8 (3.8;5.5)	79.9 (72;90.2)	2.5 (2.3;2.9)	-46.6 (-56.5;-28.3)
Amapá	0.5 (0.5;0.5)	0.5 (0.4;0.5)	1 (0.9;1.1)	0.2 (0.2;0.2)	-58.2 (-63.9;-52)
Amazonas	3.8 (3.5;4.1)	0.4 (0.4;0.5)	4.6 (4.1;5.5)	0.2 (0.1;0.2)	-61.3 (-66.6;-53.6)
Bahia	682.2 (629.5;736.3)	9.2 (8.5;9.9)	788.1 (728;871.6)	5 (4.6;5.5)	-45.7 (-51.7;-39.2)
Brasil	7049.3 (6816.6;7323.9)	7.3 (7;7.6)	5493.6 (5221.2;6014.7)	2.5 (2.3;2.7)	-66.3 (-68.3;-63.5)
Ceará	44.2 (36.7;55.8)	1 (0.9;1.3)	53.8 (48.3;61.5)	0.5 (0.5;0.6)	-47.6 (-59.4;-34.1)
Distrito Federal	235.6 (223.8;249.1)	34.9 (33.1;36.8)	211 (191.3;248.7)	9.7 (8.8;11.5)	-72.2 (-74.9;-67.6)
Espírito Santo	10.7 (9.9;11.6)	0.7 (0.7;0.8)	9.2 (8.2;10.9)	0.2 (0.2;0.3)	-69.8 (-73.4;-64.5)
Goiás	1131.6 (1067;1212.8)	55.2 (51.9;59.4)	815.2 (753.8;917.7)	13 (12;14.6)	-76.4 (-78.2;-74.2)
Maranhão	15.7 (12.4;19.5)	0.6 (0.5;0.7)	17.5 (15.5;20.8)	0.3 (0.2;0.3)	-52.4 (-61.3;-32.7)
Mato Grosso	39.6 (35.8;44.1)	4.4 (4;4.9)	57 (51.1;68.5)	1.8 (1.7;2.2)	-58.3 (-63.6;-50.8)
Mato Grosso do Sul	55 (50.5;59.3)	5.7 (5.3;6.1)	53 (47.6;60.1)	1.9 (1.7;2.2)	-66.4 (-70.5;-61.6)
Minas Gerais	2195.4 (2095;2316.9)	21 (20;22.1)	1390.3 (1294.6;1519.6)	5.5 (5.1;6)	-73.6 (-75.7;-71.4)
Pará	24.1 (21.9;26.3)	1 (1;1.1)	29.1 (25.4;35.6)	0.4 (0.4;0.5)	-58.3 (-64.6;-48.2)
Paraíba	35.3 (27.2;42.3)	1.5 (1.2;1.8)	38.3 (34.3;42.9)	0.8 (0.7;0.9)	-45.1 (-57.6;-24.7)
Paraná	384.7 (365.1;406.4)	7.4 (7;7.8)	251.4 (232.2;277.9)	2 (1.9;2.2)	-72.7 (-75.1;-69.5)
Pernambuco	163.5 (149.3;175.1)	3.5 (3.2;3.8)	143.4 (130.4;157.7)	1.5 (1.3;1.6)	-58.7 (-63.5;-51.8)
Piauí	59.5 (49.7;71.3)	4.1 (3.4;4.9)	71 (64;81.2)	2 (1.8;2.3)	-51.4 (-60.2;-38.5)
Rio de Janeiro	77 (71.5;82.6)	0.8 (0.7;0.8)	58.6 (53.1;65.7)	0.3 (0.2;0.3)	-64.8 (-68.7;-60)
Rio Grande do Norte	14.7 (12.8;17.1)	0.9 (0.8;1)	16.2 (14.3;18.3)	0.4 (0.4;0.5)	-51.4 (-62.5;-39.8)
Rio Grande do Sul	61.3 (56.8;65.7)	0.9 (0.9;1)	48.7 (44.4;55.6)	0.3 (0.3;0.4)	-64.8 (-68.6;-58.9)
Rondônia	25 (22.3;27.9)	5.8 (5.2;6.4)	24.1 (20.7;28.4)	1.7 (1.4;1.9)	-71.3 (-75.8;-65.3)
Roraima	0.5 (0.5;0.6)	0.7 (0.6;0.8)	0.8 (0.6;1)	0.2 (0.2;0.3)	-69.2 (-75.4;-60.8)
Santa Catarina	13.1 (12.1;14.1)	0.5 (0.5;0.5)	12.4 (11.1;14.3)	0.2 (0.1;0.2)	-68.2 (-72.2;-62.2)
São Paulo	1653.4 (1583.9;1727.4)	7.4 (7.1;7.8)	1248.9 (1168.1;1356.1)	2.4 (2.3;2.7)	-67 (-69.2;-64)
Sergipe	10.6 (9.4;12.1)	1.2 (1.1;1.4)	11.8 (10.7;13.3)	0.5 (0.5;0.6)	-55.3 (-62.9;-44.5)
Tocantins	42.3 (34.1;52.3)	10.1 (8.4;12.3)	56.3 (50.2;64.6)	4.1 (3.6;4.7)	-59.6 (-68.4;-48.4)

* Fonte: Estudo Global Burden of Disease 2017, Institute for Health Metrics and Evaluation. ^188^*

**Tabela 4-9:** – Número de mortes e taxa de mortalidade padronizada por idade por doença de Chagas, por 100 mil habitantes, para homens, em 1990 e 2017, e variação percentual das taxas, no Brasil e suas unidades federativas

Localização	1990		2017		Variação percentual (II 95%)
	Número de mortes (II 95%)	Taxa de mortalidade (II 95%)	Número de mortes (II 95%)	Taxa de mortalidade (II 95%)	
Acre	1.3 (1.2;1.5)	1.3 (1.2;1.5)	1.4 (1.2;1.7)	0.5 (0.4;0.6)	-64.1 (-71.8;-55.1)
Alagoas	42.6 (32.8;50.5)	6.2 (4.8;7.4)	46.5 (40.2;54.3)	3.3 (2.8;3.8)	-47.3 (-58.1;-26.3)
Amapá	0.3 (0.3;0.4)	0.6 (0.6;0.7)	0.7 (0.6;0.8)	0.3 (0.2;0.3)	-57.3 (-64.9;-48)
Amazonas	2.9 (2.5;3.2)	0.6 (0.6;0.7)	3.3 (2.8;4.1)	0.2 (0.2;0.3)	-62.6 (-69.1;-53.3)
Bahia	449.9 (402.3;501.3)	12.7 (11.3;14.2)	511.7 (459;580.7)	7.2 (6.4;8.1)	-43.3 (-51.7;-33)
Brasil	4411.2 (4207.8;4651.3)	9.5 (9;10)	3094.4 (2868.4;3448.7)	3.1 (2.9;3.4)	-67.5 (-70.1;-63.8)
Ceará	30.1 (24;40.2)	1.5 (1.2;2)	34.5 (29.7;40.3)	0.8 (0.7;0.9)	-48.3 (-62;-32.1)
Distrito Federal	136.9 (127;147.8)	41.3 (38.2;44.7)	108 (93.7;132.2)	11.2 (9.7;13.7)	-72.9 (-76.6;-67.7)
Espírito Santo	7.5 (6.8;8.4)	1 (0.9;1.1)	5.9 (5.1;7.3)	0.3 (0.3;0.4)	-70.4 (-75;-63.1)
Goiás	684.1 (634.4;744.1)	60.3 (55.7;66)	453.1 (406.4;522.9)	15.1 (13.6;17.4)	-75 (-77.7;-71.7)
Maranhão	11.4 (8.5;13.6)	0.9 (0.7;1.1)	11.9 (10.3;14.3)	0.4 (0.3;0.5)	-54.2 (-63.5;-31.4)
Mato Grosso	27 (23.5;30.8)	5.5 (4.8;6.2)	36 (31.3;43.8)	2.2 (2;2.7)	-58.8 (-65.7;-49.3)
Mato Grosso do Sul	38.9 (34.9;42.8)	7.6 (6.9;8.4)	33.5 (29.3;39.5)	2.5 (2.2;3)	-66.8 (-71.9;-60.5)
Minas Gerais	1342.2 (1256.4;1447)	26.6 (24.9;28.7)	744.3 (672;833.9)	6.5 (5.9;7.3)	-75.5 (-78.1;-72.3)
Pará	17.3 (15.4;19.4)	1.5 (1.3;1.7)	20.8 (17.5;26)	0.6 (0.5;0.8)	-58 (-66.1;-47)
Paraíba	22.1 (16.8;27.9)	2 (1.5;2.5)	23 (19.9;26.6)	1.1 (1;1.3)	-43.6 (-58.9;-17.5)
Paraná	253.3 (234.6;273.2)	9.6 (8.9;10.3)	139.8 (125;159.6)	2.5 (2.2;2.8)	-74.2 (-77.5;-70.2)
Pernambuco	103.2 (92.7;113)	4.8 (4.3;5.3)	83.8 (73.8;95)	2 (1.7;2.2)	-59.4 (-65.6;-51.2)
Piauí	40.4 (33.2;49.7)	5.8 (4.8;7.2)	44.6 (39.4;52.1)	2.7 (2.4;3.1)	-53.9 (-63.5;-40.1)
Rio de Janeiro	47.4 (42.8;52.3)	1.1 (1;1.2)	31.7 (27.2;36.8)	0.3 (0.3;0.4)	-67.4 (-72.5;-61.1)
Rio Grande do Norte	10.6 (9;12.8)	1.4 (1.1;1.6)	11.3 (9.6;13.1)	0.7 (0.6;0.8)	-50.4 (-63.4;-35.3)
Rio Grande do Sul	40.4 (36.3;44.5)	1.3 (1.2;1.5)	28.9 (25.3;34.2)	0.4 (0.4;0.5)	-67.3 (-72.2;-60.5)
Rondônia	18.1 (15.5;20.7)	7.2 (6.2;8.2)	14.5 (11.8;18)	1.9 (1.6;2.4)	-73.2 (-78.8;-65.6)
Roraima	0.5 (0.4;0.5)	1.1 (0.9;1.3)	0.6 (0.5;0.8)	0.3 (0.3;0.4)	-68.4 (-76;-58.3)
Santa Catarina	8.6 (7.7;9.5)	0.7 (0.6;0.8)	7.3 (6.2;8.7)	0.2 (0.2;0.2)	-69.9 (-74.9;-62.9)
São Paulo	1039.3 (976.2;1108.4)	9.7 (9.1;10.4)	655 (592.6;739.1)	2.9 (2.7;3.3)	-69.8 (-73.2;-65.6)
Sergipe	7 (6;8.3)	1.7 (1.5;2)	7.7 (6.7;8.8)	0.8 (0.7;0.9)	-54.3 (-64.5;-42)
Tocantins	27.7 (21.6;34.8)	12 (9.6;15.1)	34.6 (29.6;40.9)	4.9 (4.2;5.7)	-59.4 (-69.7;-45.9)

* Fonte: Estudo Global Burden of Disease 2017, Institute for Health Metrics and Evaluation. ^188^*

**Tabela 4-10:** – Número de mortes e taxa de mortalidade padronizada por idade por doença de Chagas, por 100 mil habitantes, para mulheres, em 1990 e 2017, e variação percentual das taxas, no Brasil e suas unidades federativas

Localização	1990		2017		Variação percentual (II 95%)
	Número de mortes (II 95%)	Taxa de mortalidade (II 95%)	Número de mortes (II 95%)	Taxa de mortalidade (II 95%)	
Acre	0.4 (0.4;0.5)	0.5 (0.5;0.6)	0.6 (0.5;0.7)	0.2 (0.2;0.2)	-58.7 (-65.8;-49.8)
Alagoas	25.8 (20.2;30.7)	3.5 (2.7;4.2)	33.4 (28.8;38.2)	1.9 (1.7;2.2)	-44.4 (-57.5;-24.8)
Amapá	0.2 (0.1;0.2)	0.3 (0.3;0.3)	0.3 (0.3;0.4)	0.1 (0.1;0.1)	-58.5 (-67;-49.9)
Amazonas	0.9 (0.8;1)	0.2 (0.2;0.2)	1.3 (1.2;1.6)	0.1 (0.1;0.1)	-56.4 (-63.1;-46.9)
Bahia	232.2 (210.6;255.3)	6.1 (5.5;6.7)	276.5 (253.1;304.8)	3.2 (2.9;3.5)	-47.8 (-54.4;-40)
Brasil	2638.1 (2545.4;2737.8)	5.3 (5.1;5.5)	2399.2 (2255.9;2597.1)	1.9 (1.8;2.1)	-63.7 (-66.3;-60.7)
Ceará	14.1 (11.5;18)	0.6 (0.5;0.8)	19.3 (17;22.2)	0.4 (0.3;0.4)	-44.4 (-59.3;-27.6)
Distrito Federal	98.7 (91.8;106.5)	29.2 (27.3;31.3)	103 (91.5;121.4)	8.5 (7.6;10.1)	-70.9 (-74.4;-66.1)
Espírito Santo	3.2 (2.9;3.5)	0.4 (0.4;0.5)	3.3 (2.9;3.9)	0.1 (0.1;0.2)	-67.3 (-72.1;-61.3)
Goiás	447.5 (419.6;480.6)	50.1 (46.8;54.1)	362 (329.1;407.7)	11.1 (10.1;12.5)	-77.9 (-80;-75.4)
Maranhão	4.3 (3;6.8)	0.3 (0.2;0.5)	5.6 (4.9;6.8)	0.2 (0.1;0.2)	-47.1 (-64.2;-23.3)
Mato Grosso	12.6 (11;14.5)	3.2 (2.8;3.6)	21 (18.4;25.5)	1.4 (1.3;1.7)	-55.4 (-62.5;-45.2)
Mato Grosso do Sul	16.1 (14.6;17.8)	3.6 (3.3;4)	19.5 (17;22.6)	1.4 (1.2;1.6)	-62.8 (-68.3;-56.2)
Minas Gerais	853.2 (809.6;904.2)	15.9 (15.1;16.8)	646 (593.4;706.5)	4.6 (4.3;5.1)	-70.8 (-73.7;-67.6)
Pará	6.8 (6;7.6)	0.6 (0.5;0.7)	8.3 (7.2;10)	0.2 (0.2;0.3)	-58.6 (-65.5;-49.1)
Paraíba	13.3 (9.7;15.6)	1.1 (0.8;1.3)	15.3 (12.9;17.7)	0.6 (0.5;0.7)	-45.9 (-58.7;-22.1)
Paraná	131.4 (122;140.3)	5.3 (4.9;5.6)	111.6 (100.4;125.1)	1.7 (1.5;1.9)	-68.8 (-72.3;-64.8)
Pernambuco	60.3 (53.4;66.2)	2.4 (2.2;2.7)	59.7 (52.8;67.5)	1.1 (0.9;1.2)	-56.4 (-62.7;-47)
Piauí	19 (15.4;24.5)	2.5 (2;3.2)	26.3 (23.1;30.8)	1.3 (1.2;1.6)	-46.1 (-59.2;-30)
Rio de Janeiro	29.5 (27;32.4)	0.5 (0.5;0.6)	26.9 (23.6;30.4)	0.2 (0.2;0.2)	-60.8 (-66.1;-54.6)
Rio Grande do Norte	4 (3.5;4.8)	0.5 (0.4;0.6)	4.9 (4.2;5.6)	0.2 (0.2;0.3)	-50.5 (-63.1;-37.1)
Rio Grande do Sul	20.9 (18.9;22.7)	0.6 (0.5;0.6)	19.8 (17.5;22.6)	0.2 (0.2;0.3)	-60.7 (-65.8;-54.1)
Rondônia	6.9 (6.1;7.8)	3.9 (3.5;4.4)	9.6 (7.9;11.6)	1.4 (1.1;1.7)	-64.8 (-72.1;-56.2)
Roraima	0.1 (0.1;0.1)	0.3 (0.2;0.3)	0.2 (0.1;0.2)	0.1 (0.1;0.1)	-63 (-72.2;-52)
Santa Catarina	4.6 (4.1;5.1)	0.4 (0.3;0.4)	5.1 (4.5;6)	0.1 (0.1;0.1)	-65 (-70;-58.7)
São Paulo	614.1 (583.5;644.5)	5.4 (5.1;5.6)	593.9 (544.1;650.2)	2 (1.9;2.2)	-61.9 (-65.3;-58.1)
Sergipe	3.6 (3.1;4.2)	0.8 (0.7;0.9)	4.1 (3.6;4.7)	0.3 (0.3;0.4)	-55.9 (-63.5;-43)
Tocantins	14.5 (11.6;19)	7.9 (6.5;10.2)	21.7 (18.6;25.7)	3.2 (2.8;3.8)	-59 (-70;-46.2)

* Fonte: Estudo Global Burden of Disease 2017, Institute for Health Metrics and Evaluation. ^188^*

**Tabela 4-11:** – Prevalência e taxa de prevalência padronizada por idade de insuficiência cardíaca por todas as causas (por 100 mil habitantes) e variação percentual das taxas, 1990 e 2017, no Brasil e suas unidades federativas.

Localização	1990	Taxa (II 95%)	2017	Taxa (II 95%)	Variação percentual (II 95%)
	Número (II 95%)		Número (II 95%)		
Brasil	670194.8 (589952.6;753672.6)	818.1 (718.1;922.8)	1686320.1 (1478563.8;1890537.3)	777.2 (680;874.8)	-5 (-7.1;-3)
Acre	1235.9 (1083.5;1395.6)	764.3 (668.5;869)	4025.6 (3559.4;4534.9)	728.8 (638.1;830.1)	-4.6 (-10.1;1.8)
Alagoas	9783 (8509.2;11210.5)	752.5 (654.5;861.9)	22691.5 (19784.3;25922)	764.8 (664.4;879.3)	1.6 (-5.7;8.3)
Amapá	748.2 (662;841.1)	779.1 (680.7;889.6)	3278.9 (2865.5;3672.3)	749.3 (651.9;845.2)	-3.8 (-9.5;2.6)
Amazonas	6097.6 (5376.6;6855.3)	809.8 (709.1;919)	19459.2 (17131;21872.1)	775.9 (678.8;884.1)	-4.2 (-10.2;1.9)
Bahia	52840.3 (46323.4;60082)	783.5 (685.2;893)	118062.7 (103361.3;134066.1)	753.3 (656.9;857.9)	-3.8 (-9.6;2.5)
Ceará	30093.8 (26385.1;34137.4)	739.5 (648;842.1)	77144.8 (67097.6;87800.2)	785.5 (682.6;896)	6.2 (-0.8;14.2)
Distrito Federal	4256.7 (3710.8;4838.9)	813.3 (701.7;932)	16100.7 (13996.8;18333.4)	753.5 (654.5;850.2)	-7.4 (-13.1;-0.6)
Espírito Santo	11320.9 (9847.7;12942.2)	841.5 (730.3;961.6)	31391.5 (27390.7;35566.7)	782.7 (680;889.7)	-7 (-13.2;-0.6)
Goiás	14142 (12371.7;16150.6)	800.7 (703.5;912.7)	46168.1 (40298.2;52244.1)	753 (655.9;854)	-6 (-12.9;1)
Maranhão	18235.7 (15857.1;20802.4)	747.2 (650.9;852.8)	49180.9 (43277;55993.9)	795.1 (697.6;907.8)	6.4 (0.2;13.5)
Mato Grosso	5774.8 (5067;6502.3)	819.3 (712.6;938)	21845.3 (19017.4;24622)	789.4 (688;895.4)	-3.7 (-9.6;3)
Mato Grosso do Sul	6795.1 (5934.8;7652)	846.4 (740.2;961.2)	21183 (18418.4;24002)	816 (710.9;922.6)	-3.6 (-9.3;3.4)
Minas Gerais	74411.2 (64608;84527.1)	826.9 (722;940.5)	187809.8 (163412.5;214570.5)	759.5 (659.4;867)	-8.1 (-14.2;-1.6)
Pará	16002.3 (14153.8;18014.5)	789.4 (694.3;893.2)	46324.1 (40809.4;52186.1)	746.6 (652.1;844.8)	-5.4 (-11.3;0.9)
Paraíba	17922.4 (15619.1;20442.3)	772 (675.4;881)	36827.2 (32186.6;41911.1)	794.6 (693.4;902.5)	2.9 (-3.6;10.2)
Paraná	35843.5 (31360.3;40661.2)	834.4 (726.6;948.5)	93386.5 (81689;106563.6)	779.7 (684;892.6)	-6.6 (-12.2;0.1)
Pernambuco	34084.1 (29826.4;39017.2)	793.9 (695;908.1)	72004 (62756.3;81953.9)	753.9 (655.5;862.9)	-5 (-11.2;1.3)
Piauí	11016.2 (9596.9;12503.6)	803.5 (698.9;912.6)	29097.2 (25425.9;33105.2)	812.7 (708.9;927)	1.1 (-5.6;8.5)
Rio de Janeiro	72976.8 (63619.5;83192)	850.4 (744;970.5)	162697.6 (140265.7;185508.6)	778.9 (674.2;887.1)	-8.4 (-14.1;-1.7)
Rio Grande do Norte	13462.3 (11756.9;15396.2)	827 (720.5;948.1)	31332.8 (27529.6;35470.6)	839 (734;955.6)	1.5 (-4.9;8.1)
Rio Grande do Sul	51590.7 (45263.1;58166.6)	862.8 (754.1;980)	115132.9 (100064.2;130860)	787.4 (685.2;894.6)	-8.7 (-14.7;-2.9)
Rondônia	2451.8 (2150.6;2766.5)	813.4 (710.9;925.4)	9980.6 (8742.3;11325)	766 (669.2;872.1)	-5.8 (-11.9;1)
Roraima	419.1 (368.5;471.8)	809.3 (706.3;925)	2297.9 (2000.3;2611)	774.6 (674.4;884.7)	-4.3 (-10.5;2)
Santa Catarina	19387.5 (16978;21842.4)	847 (741.4;957.7)	55662.9 (48893.1;63470.8)	779.6 (685.4;894.9)	-8 (-13.7;-2)
São Paulo	150009 (131202;169778.7)	842.8 (734.6;959.2)	387169.5 (336629;442688.8)	787.9 (685;899.2)	-6.5 (-12.8;-0.4)
Sergipe	6409.8 (5592.5;7302.6)	754.4 (657.2;860.7)	15587.4 (13661.6;17664.6)	763.8 (668.4;870.4)	1.2 (-4.7;8.8)
Tocantins	2884.2 (2501.8;3284.4)	789.5 (691.7;906.1)	10477.4 (9147.4;11899)	796.5 (692.4;907.9)	0.9 (-5.9;8.5)

* Fonte: Estudo Global Burden of Disease 2017, Institute for Health Metrics and Evaluation.188 *

**Tabela 4-12:** – Taxa de prevalência de insuficiência cardíaca, por 100 mil habitantes, e variação percentual das taxas, por sexo e grupos etários, Brasil, 1990 e 2017

Grupo etário	1990	2017	Variação percentual (II 95%)
	Prevalência	Prevalência	
Padronizada por idade	818,1 (718,1;922,8)	777,2 (680;874,8)	-5 (-7,1;-3)
Abaixo de 5	46,3 (32;63,8)	45 (30,8;61,9)	-2,9 (-5,5;-0,1)
5-14 anos	34,7 (24,2;47,2)	34,1 (23,6;46,7)	-1,6 (-4,5;1,3)
15-49 anos	107,1 (90,7;124,8)	119 (100,2;139,3)	11,1 (5,5;15,6)
50-69 anos	1391,6 (1172,1;1627,5)	1330,4 (1125,6;1570,4)	-4,4 (-6,9;-1,8)
70+ anos	8249,1 (6918,9;9752,5)	8530,2 (7265,9;9922,9)	3,4 (-1;8)
Todas as idades	448,5 (394,8;504,4)	796,1 (698,1;892,6)	77,5 (72,3;82,4)
***Masculino***			
Padronizada por idade	811,8 (714;916,9)	750,6 (656,2;845)	-7,5 (-10,2;-4,8)
Abaixo de 5	46,8 (32,2;64,5)	45,2 (31;62,1)	-3,6 (-7,2;0,4)
5-14 anos	34,3 (23,7;46,9)	33,4 (23,1;45,9)	-2,5 (-6,6;1,7)
15-49 anos	105,3 (89;122,4)	114,2 (95,6;134,7)	8,5 (0,5;14,2)
50-69 anos	1386,9 (1164,2;1643,5)	1311,3 (1102,4;1555,5)	-5,4 (-9,1;-1,5)
70+ anos	8083,9 (6784,9;9549,3)	7926,1 (6721,2;9286,2)	-2 (-6,4;2,9)
Todas as idades	415,6 (367,7;466,8)	685 (602,9;770)	64,8 (59,3;70,4)
***Feminino***			
Padronizada por idade	820,9 (721;933,2)	794,7 (694,4;900,6)	-3,2 (-6,5;-0,1)
Abaixo de 5	45,8 (31,6;63,3)	44,8 (30,7;63,3)	-2,1 (-5,7;2,2)
5-14 anos	35,1 (24,7;47,9)	34,9 (24,3;47,2)	-0,7 (-4,6;3,1)
15-49 anos	109 (91,7;126,6)	123,7 (103,9;144)	13,5 (8,8;18,3)
50-69 anos	1395,9 (1183,9;1632,2)	1347,3 (1137,1;1586,4)	-3,5 (-6,9;0,3)
70+ anos	8381,9 (7012,1;9982,4)	8968,9 (7622,9;10482,3)	7 (1,1;12,8)
Todas as idades	480,8 (422,3;544,4)	902,3 (790,2;1020,9)	87,7 (81;94,4)

* Fonte: Estudo Global Burden of Disease 2017, Institute for Health Metrics and Evaluation. ^188^*

**Tabela 4-13:** – Número de YLDs e taxa de YLDs padronizada por idade (por 100 mil habitantes) por insuficiência cardíaca por todas as causas, e variação percentual das taxas, 1990 e 2017, no Brasil e suas unidades federativas

Localização	1990	Taxa (II 95%)	2017	Taxa (II 95%)	Variação percentual (II 95%)
	Número (II 95%)		Número (II 95%)		
Brasil	88114.2 (64078.1;112623.9)	112.2 (82.8;141.2)	234168.9 (174338.9;291187.7)	108.8 (81.4;134.5)	-3 (-6.7;0.3)
Acre	188.7 (140.7;234.7)	123.8 (95.3;148.9)	636.8 (490.6;767.6)	119.8 (93.4;142.4)	-3.2 (-9.6;5.2)
Alagoas	1230.4 (880.5;1587.3)	97.7 (70.9;124.1)	2951 (2189.4;3685.1)	101 (75;125.8)	3.3 (-5.8;12)
Amapá	100.2 (73.7;127.2)	112.2 (84.5;139.2)	451.5 (337.3;558.9)	108.9 (83.4;132.6)	-3 (-9.5;5)
Amazonas	810.4 (592.2;1020.8)	115.9 (87.9;141.5)	2702.1 (2050;3305.1)	112.2 (86.1;135.8)	-3.1 (-10;3.9)
Bahia	6684.8 (4893.5;8540.9)	101.7 (75.5;129)	15736.9 (11812.7;19456)	100.8 (75.3;125.3)	-0.8 (-7.7;7.9)
Ceará	3969.6 (2897.9;5084.5)	99.2 (72.8;126.2)	10451.4 (7838.4;12882.6)	106.6 (79.8;132)	7.4 (-1.3;17)
Distrito Federal	431.1 (298.6;581.8)	97.6 (70.8;128.8)	1940.3 (1366.1;2560)	96.9 (69.9;125.4)	-0.8 (-8.1;9.2)
Espírito Santo	1462.1 (1054.4;1878.9)	113.4 (84.3;143.6)	4162.7 (3063.9;5243.2)	104.9 (77.5;131.9)	-7.5 (-14.4;0.1)
Goiás	1592.7 (1128;2094.6)	103.8 (76.7;132.4)	6291.2 (4685;7868.1)	106.1 (79.5;131.7)	2.2 (-6.5;11.5)
Maranhão	2190.7 (1560.1;2873.3)	92 (66.4;119.5)	5992.2 (4402.4;7553.5)	97.8 (72;122.9)	6.3 (-1.6;15.9)
Mato Grosso	725.4 (526.7;941)	113.1 (83.9;142.3)	3003 (2248.2;3728.7)	112 (84.7;137.3)	-0.9 (-8.1;7.7)
Mato Grosso do Sul	850.1 (609.1;1106.8)	113.3 (83.8;142.8)	2829.1 (2072.2;3549.4)	110.8 (82.1;137.7)	-2.2 (-9.5;6.5)
Minas Gerais	9477.1 (6808.4;12335.3)	110.9 (82.3;141.3)	25557 (18974.8;31992.6)	103.6 (77.2;129.4)	-6.5 (-14.4;1.6)
Pará	2221.3 (1651.1;2795.8)	115.7 (88.1;142.8)	6624.9 (5036.2;8052.8)	109.8 (84.8;132.1)	-5.2 (-12.2;2.3)
Paraíba	2298.3 (1666.9;2947.6)	99.5 (72.4;126.3)	4808 (3560.3;5995.7)	103.4 (76.3;129.4)	4 (-4.2;12.5)
Paraná	4964.3 (3563.3;6413.6)	123.5 (92.3;154.2)	13883.5 (10128.6;17249.1)	117 (86.8;144.4)	-5.2 (-12.7;3.3)
Pernambuco	4627.4 (3326;5865.3)	110.9 (81.9;137.8)	10375.1 (7650.4;12784)	109.6 (81;134.6)	-1.2 (-9;7.3)
Piauí	1230.3 (866.5;1647.8)	92.1 (65.6;122)	3402.3 (2478.9;4365.3)	95.1 (69.4;122.2)	3.3 (-5;11.9)
Rio de Janeiro	9922.7 (7229.9;12662.8)	119.7 (88.6;150.3)	22953.2 (16743.8;28786)	110 (81.3;137.7)	-8.1 (-14.7;-0.4)
Rio Grande do Norte	1606.1 (1145.6;2057.5)	99.5 (71.8;126.6)	3856.8 (2838.4;4879.3)	103.5 (75.7;132)	4 (-3.9;12)
Rio Grande do Sul	8134 (5994.6;10280.5)	140.5 (106.7;171.9)	18696.6 (14241.9;22612.8)	126.9 (97;152.5)	-9.7 (-16.6;-2.5)
Rondônia	300.3 (209.4;397.4)	117.4 (86.9;145.5)	1408.5 (1062.2;1750.7)	111.9 (85.8;137)	-4.7 (-11.7;4.4)
Roraima	46.3 (32.4;62.3)	101.5 (74.8;130.8)	276.8 (197.7;358.6)	98.7 (72.2;125.2)	-2.8 (-9.7;5)
Santa Catarina	2880 (2095.3;3688.1)	133.3 (99.4;165.6)	8433.4 (6334.3;10482.5)	119.7 (90.8;147.4)	-10.2 (-16.8;-3.4)
São Paulo	18988.5 (13523.4;24849.5)	112.8 (81.9;144.5)	53360.6 (38622.8;68602.6)	109.6 (80.1;140.1)	-2.9 (-10.6;5.1)
Sergipe	853.9 (627.3;1082.5)	102.4 (75.6;129)	2085.5 (1551.2;2578.3)	103.7 (77.4;127.2)	1.3 (-6.3;9.9)
Tocantins	327.5 (226.3;438.8)	96.4 (69.6;126.2)	1298.6 (952.1;1635.1)	100.3 (73.9;125.9)	4 (-4.3;14.5)

* Fonte: Estudo Global Burden of Disease 2017, Institute for Health Metrics and Evaluation. ^188^*

**Tabela 4-14:** – Taxa de YLDs por insuficiência cardíaca (por 100 mil habitantes) e variação percentual das taxas, por idade e sexo, Brasil, 1990 e 2017

Grupo etário	1990	2017	Variação percentual (II 95%)
***Ambos os sexos***			
Padronizada por idade	112,2 (82,8;141,2)	108,8 (81,4;134,5)	-3 (-6,7;0,3)
Abaixo de 5	4,5 (2,7;7)	4,3 (2,6;6,8)	-3,4 (-6;-0,6)
5-14 anos	3,2 (1,9;5,1)	3,2 (1,9;5)	-1,4 (-4,4;1,5)
15-49 anos	8,7 (5,7;12,6)	10,4 (6,8;14,8)	18,7 (12,3;25,5)
50-69 anos	165,8 (112,7;226,8)	166,4 (115,3;228,9)	0,3 (-3,8;5,5)
70+ anos	1263,1 (919,3;1599,5)	1308,3 (988,6;1586)	3,6 (-3,2;10,2)
Todas as idades	59 (42,9;75,4)	110,6 (82,3;137,5)	87,5 (78,8;96,2)
***Masculino***			
Padronizada por idade	112,9 (86,9;137,4)	105,2 (81,6;127,1)	-6,8 (-10,9;-2,6)
Abaixo de 5	4,5 (2,7;7,1)	4,3 (2,6;6,8)	-4,2 (-7,8;-0,2)
5-14 anos	3,2 (1,9;5)	3,1 (1,8;4,9)	-2,2 (-6,4;2)
15-49 anos	7,9 (5,1;11,6)	9,5 (6,1;13,8)	19,8 (10,2;29,3)
50-69 anos	165,5 (111,9;230,9)	165,8 (113,5;229,4)	0,2 (-5,9;6,8)
70+ anos	1282,5 (973,9;1555,8)	1225,7 (980;1439,6)	-4,4 (-11,1;3,4)
Todas as idades	55 (40,6;68,5)	94,5 (72,6;115,7)	71,9 (63,1;81,5)
***Feminino***			
Padronizada por idade	111,2 (79,9;145)	110,9 (80,1;140,6)	-0,3 (-4,9;4,2)
Abaixo de 5	4,4 (2,7;7)	4,3 (2,6;6,7)	-2,6 (-6,3;1,7)
5-14 anos	3,2 (1,9;5,2)	3,2 (1,9;5,1)	-0,6 (-4,5;3,2)
15-49 anos	9,5 (6,3;13,4)	11,2 (7,4;15,9)	17,9 (10,5;26,4)
50-69 anos	166,1 (112,5;225,8)	166,9 (116,6;228,9)	0,5 (-5;6,7)
70+ anos	1247,5 (876,2;1659,2)	1368,3 (992,5;1714,5)	9,7 (0,6;17,6)
Todas as idades	62,9 (44,1;82,5)	125,9 (90,9;159,5)	100,2 (88,8;111,5)

* Fonte: Estudo Global Burden of Disease 2017, Institute for Health Metrics and Evaluation. ^188^*

**Figura 4-3:**
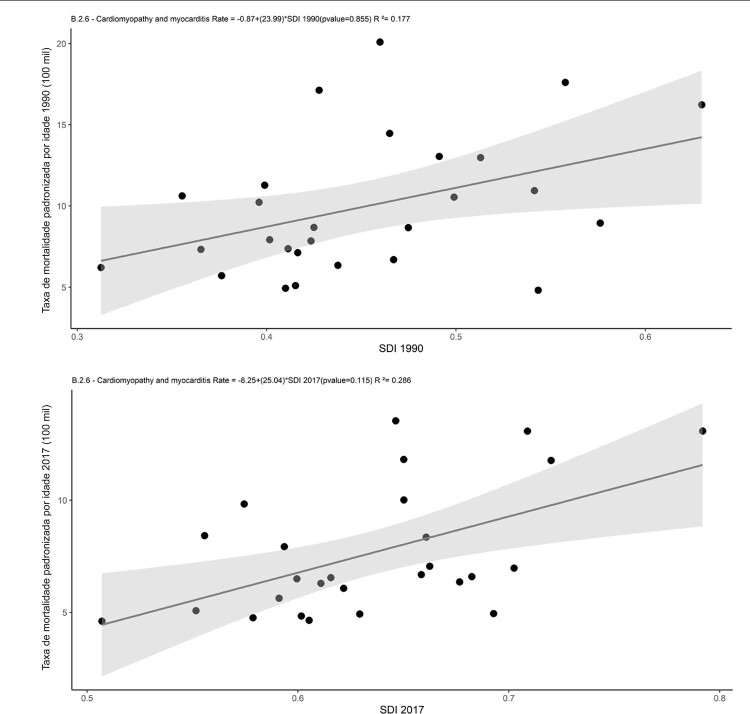
- Correlação entre o índice sociodemográfico (SDI) e a taxa de mortalidade padronizada por idade por cardiomiopatia e miocardite, por 100 mil habitantes, Brasil, 1990 e 2017.

Dados derivados do Estudo Global Burden of Disease 2017 (GBD 2017). ^188^

**Figura 4-4:**
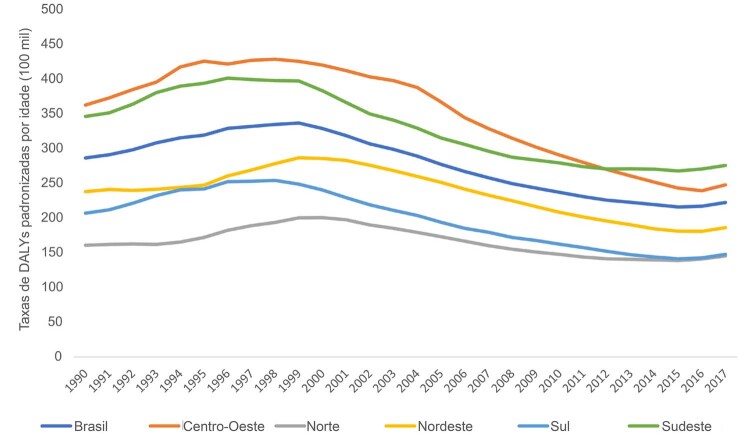
- Taxa de DALYs atribuíveis a cardiomiopatia e miocardite padronizada por idade (por 100 mil habitantes) no Brasil e regiões brasileiras, de 1990 a 2017.

Dados derivados do Estudo Global Burden of Disease 2017 (GBD 2017). ^188^

**Figura 4-5:**
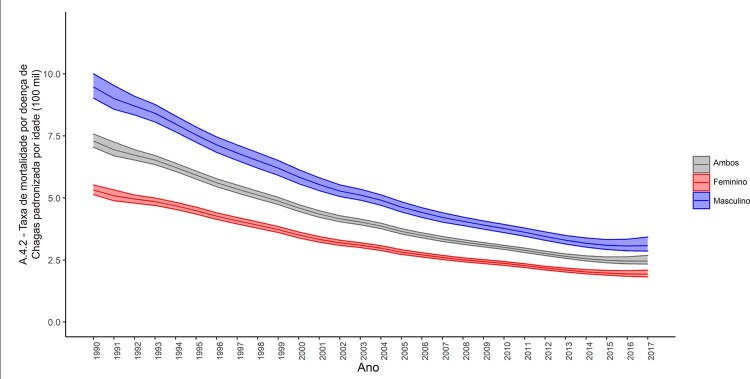
- Taxa de mortalidade padronizada por idade atribuível a doença de Chagas no Brasil de 1990 a 2017.

Dados derivados do Estudo Global Burden of Disease 2017 (GBD 2017). ^188^

**Figura 4-6:**
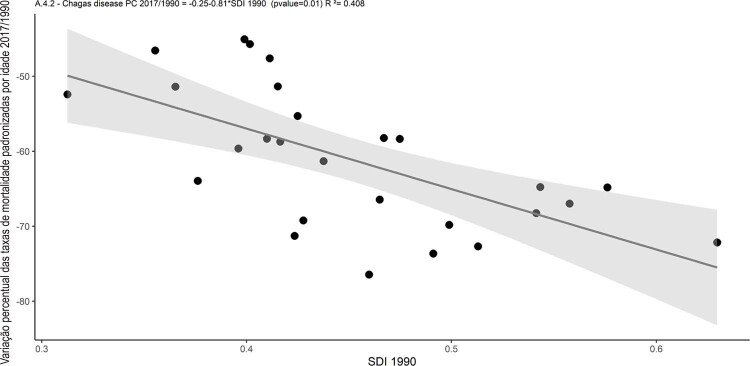
- Correlação entre o índice sociodemográfico (SDI) em 1990 e a variação percentual das taxas de mortalidade padronizadas por idade por doença de Chagas, por 100 mil habitantes, 2017/1990.

Dados derivados do Estudo Global Burden of Disease 2017 (GBD 2017). ^188^

**Figura 4-7:**
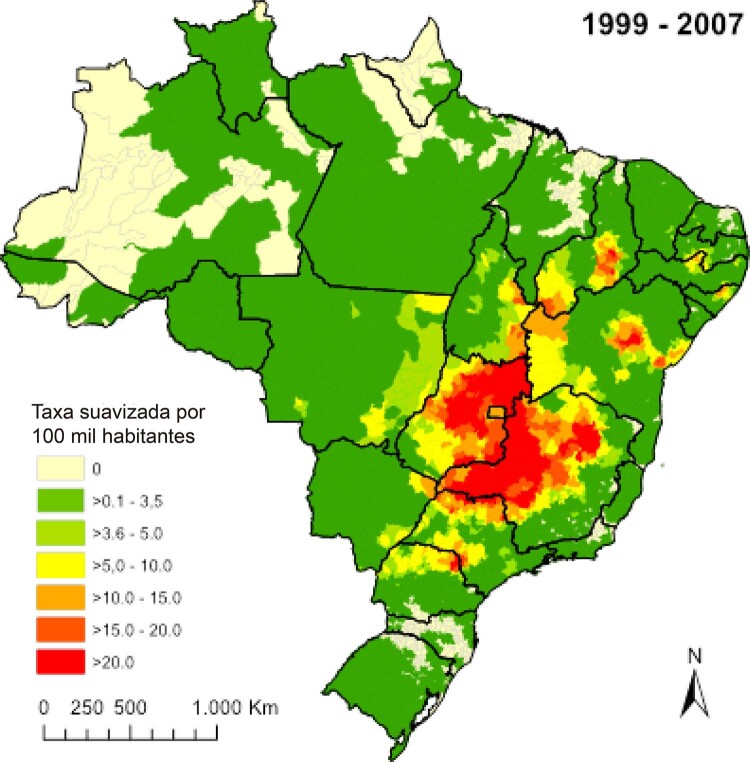
- Distribuição espacial da taxa de mortalidade média relacionada a doença de Chagas (por 100 mil habitantes) com base em causa múltipla de morte, por município, Brasil, 1999–2007.

Fonte: Martins-Melo et al. ^161^

**Figura 4-8:**
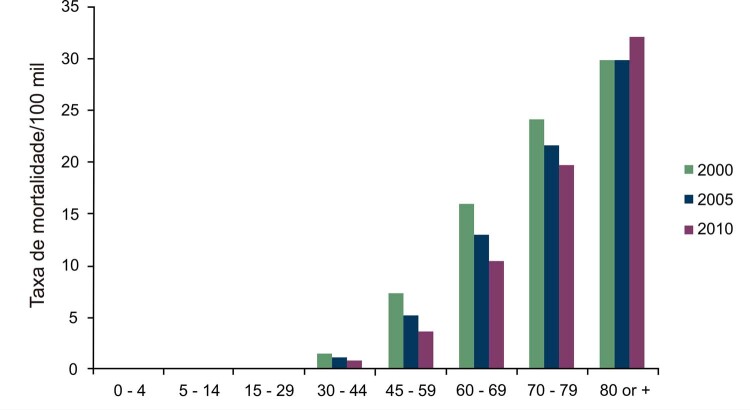
- Taxa de mortalidade padronizada por doença de Chagas no Brasil de acordo com grupo etário e ano de ocorrência, de 2000 a 2010.

Fonte: Nóbrega et al. ^159^

**Figura 4-9:**
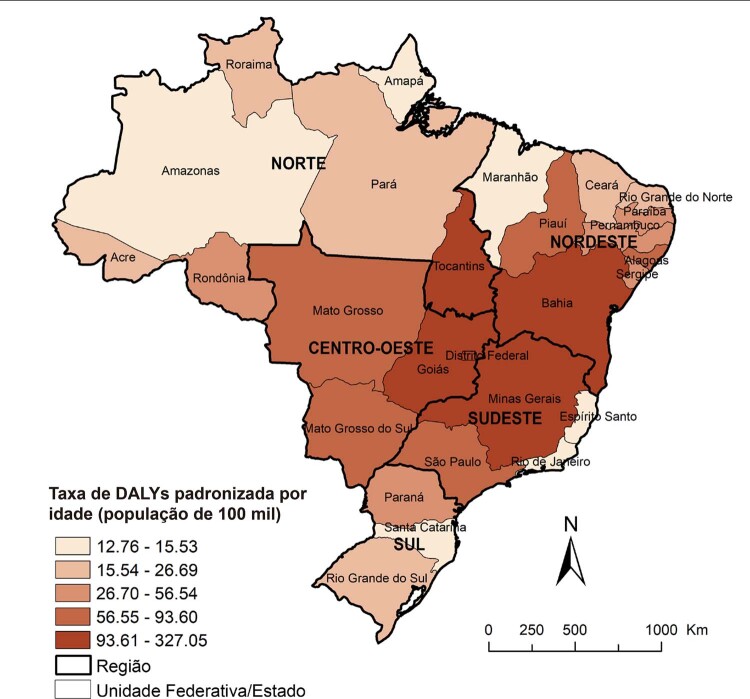
- Taxa de DALYs (por população de 100 mil) padronizada por idade por doença de Chagas em 2016.

Fonte: Martins-Melo et al. ^166^

**Figura 4-10:**
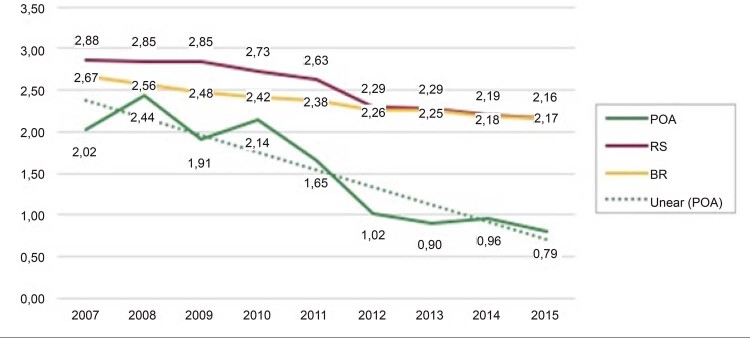
- Tendências da mortalidade por insuficiência cardíaca de 2007 a 2016 no Brasil (BR), no Rio Grande do Sul (RS) e em Porto Alegre (POA).

Fonte: Nicolao et al. ^183^

## 5. DOENÇA VALVAR DO CORAÇÃO

### CID-9 424; CID-10 I34 a I38

Ver Tabelas 5-1 a 5-4 e Figuras 5-1 a 5-10

**Table:** 

Abreviaturas usadas no Capítulo 5	
CID	Classificação Estatística Internacional de Doenças e Problemas Relacionados à Saúde
CID-9	Classificação Estatística Internacional de Doenças e Problemas Relacionados à Saúde, 9 ^a^ Revisão
CID-10	Classificação Estatística Internacional de Doenças e Problemas Relacionados à Saúde, 10 ^a^ Revisão
CRVM	Cirurgia de Revascularização do Miocárdio
DAC	Doença Arterial Coronariana
DALYs	Anos de vida perdidos ajustados por incapacidade (do inglês, *Disability-Adjusted Life-Year* )
DCR	Doença Cardíaca Reumática
DVNR	Doença Valvar Não Reumática
ECG	Eletrocardiograma
FA	Fibrilação Atrial
FRA	Febre Reumática Aguda
GBD	*Global Burden of Doença*
IC	Intervalo de Confiança
II	Intervalo de Incerteza
OR	*Odds Ratio*
SDI	Índice Sociodemográfico (do inglês, *Sociodemographic Index* )
SUS	Sistema Único de Saúde
TAVI	Implantação Percutânea de Valva Aórtica (do inglês, *Transcatheter Aortic Valve Implantation* )
UF	Unidade Federativa
YLLs	Anos potenciais de vida perdidos (do inglês, * Years of Life Lost* )

### Prevalência

#### Doença Cardíaca Reumática

- De acordo com o *Global Atlas on Cardiovascular Disease Prevention and Control* , estima-se que a DCR afete atualmente cerca de 33 milhões de pessoas em todo o mundo, sendo responsável por 1% a 1,5% (319.400 mortes) de todas as mortes cardiovasculares. ^189^ Até a metade do século 20, a DCR foi a principal causa de doença valvar cardíaca no mundo. Melhores condições de saúde, identificação precoce das infecções por *Streptococcus pyogenes* , além do uso de antibióticos, reduziram significativamente a prevalência de DCR em países de alta renda. Dados publicados em 2016 estimaram que a DCR seja a causa primária de 2,5% das doenças valvares cardíacas nos Estados Unidos e no Canadá, chegando a 22% na Europa. ^190^ Taxas ainda mais altas foram relatadas no Brasil, sendo a DCR responsável por cerca de 50% das cirurgias valvares no SUS. ^191 - 193^
- Em países de renda baixa e média, por outro lado, a prevalência de DCR está estimada em 444 por 100 mil habitantes. ^194^ No Brasil, a DCR persiste como a principal etiologia das doenças valvares cardíacas, em especial em pacientes do SUS. Avaliações anteriores mostraram prevalência de 3,6 por 1.000 habitantes no Brasil. ^195^ Outras avaliações encontraram prevalência variando de 1 a 7 por 1.000 crianças em idade escolar. ^196^
- No Brasil, dos 174 pacientes com doença valvar cardíaca aguda que se apresentaram na emergência do Instituto do Coração de São Paulo, observou-se envolvimento reumático em 60%, seguindo-se doença valvar aórtica degenerativa (15%) e prolapso de valva mitral (13%). No total, 27,5% dos pacientes apresentavam regurgitação mitral isolada e 11% tinham estenose mitral, estando a doença valvar aórtica presente nos demais. ^197^
- Um estudo recente no Brasil mostrou que as taxas de resolução da doença valvar cardíaca, em especial em pacientes com FRA moderada/grave, podem ser menores do que anteriormente descrito. Apenas 22/69 pacientes (31,9%) apresentaram resolução total da regurgitação mitral após cardite reumática. A resolução total da regurgitação aórtica também foi menos frequente do que a relatada em estudos anteriores à ‘era ecocardiográfica’ (18%). A maioria dos casos persistiu com regurgitação mitral leve ou aórtica residual. ^198^ Em outro estudo envolvendo 258 crianças e adolescentes com FRA acompanhados por 2 a 15 anos, involução das lesões valvares ocorreu em 25% dos pacientes com cardite leve, em 2,5% daqueles com cardite moderada e em nenhum daqueles com cardite grave. ^199^
- De acordo com o GBD 2017, de 1990 a 2017, a prevalência padronizada por idade de DCR apresentou discreto aumento de 3,0% (II 95%, 1,6 - 4,3), passando de 721,4 (II 95%, 688,7 - 754,5) para 743,2 (II 95%, 709 - 778,6) por 100 mil habitantes, permanecendo mais alta em mulheres do que em homens em todo o período ( Tabela 5-1 e Figura 5-1.A ). Ainda que pequeno em ambos os sexos, o aumento percentual foi numericamente mais pronunciado em mulheres (4,4%) do que em homens. Os aumentos percentuais foram mais altos no Piauí (região Nordeste), Tocantins e em Roraima (região Norte). Embora as estimativas centrais fossem mais altas nesses estados, os II 95% foram amplos e se sobrepuseram àqueles de outras UF ( Tabela 5-1 ). ^200^ Entretanto, pode-se levantar a hipótese de que o pequeno aumento na prevalência de DCR observado no período reflita o avanço ocorrido na coleta de dados epidemiológicos e nas estatísticas de saúde. ^201^
- A prevalência bruta de DCR, entretanto, aumentou 16,0% (II 95%, 14,4 - 17,5%) de 1990 a 2017, passando de 690,2 (657,9 – 723,6) para 800,3 (764,1 – 838,2) por 100 mil habitantes, permanecendo também maior nas mulheres no período ( Figura 5-1.B ). À semelhança da tendência das taxas de prevalência padronizadas por idade, o aumento na prevalência bruta foi mais pronunciado entre as mulheres do que entre os homens. ^200^
- A prevalência proporcional de DCR na população brasileira, portanto, mostrou o mesmo padrão observado para a prevalência padronizada por idade e a bruta, com leve aumento para ambos os sexos de 1990 a 2017 ( Figura 5-1.C ). ^200^
- Mesmo com as tendências relativamente estáveis mostradas através da modelagem GBD 2017, a DCR é a causa mais prevalente de doença valvar mitral no Brasil de acordo com os dados publicados, considerando-se tanto estenose (mais de 90%) quanto regurgitação mitral (cerca de 55-60%). ^197^
- Estenose mitral ocorre com maior frequência em mulheres do que em homens, na razão de 3 para 2. Trata-se de sequela frequente de FRA, afetando mais de 85% dos casos mesmo em países de alta renda, como os europeus, ^202^ com padrão similar ao observado no Brasil. ^193 , 197^ Mais raramente, a estenose mitral está associada com outras doenças, como calcificação do anel mitral, mucopolissacaridose, artrite reumatoide e síndrome carcinoide congênita. ^190 , 197^
- Estudos mais recentes de rastreio em larga escala, analisando DCR subclínica, mostraram prevalência de 42 por 1.000 crianças em idade escolar, com idade média de 11 anos, em Minas Gerais, sendo 37 por 1.000 para DCR *borderline* e 5 por 1.000 para DCR definitiva (0,5%). Nesses estudos, maior prevalência foi observada em meninas (48 por 1.000 vs. 35 por 1.000) e em crianças acima dos 12 anos. ^191^ O mesmo projeto concluiu que os centros de atenção primária são o cenário ideal para rastreio de DCR, considerando-se as maiores taxas de participação e envolvimento da população. ^203^

#### Doença Valvar do Coração Não Reumática

- De acordo com o GBD 2017, a prevalência padronizada por idade de DVNR permaneceu relativamente estável no Brasil de 1990 a 2017, com aumento *borderline* de 8,2%, passando de 216,2 (II 95%, 207,9 – 224,6) por 100 mil em 1990 para 233,9 (224,6 – 243,2) por 100 mil em 2017. A variação percentual foi similar para homens e mulheres (8,9% vs. 7,8%) (Figura 5-2.A). Por outro lado, a doença valvar aórtica calcífica mostrou uma tendência crescente (20,4%), passando de 53,5 (II 95%, 48,1 – 59,9) por 100 mil em 1990 para 64,4 (II 95%, 57,2 – 72,5) por 100 mil em 2017, tanto para homens (18,5%) quanto para mulheres (24,2%). Para doença valvar mitral degenerativa e outras DVNR, a prevalência padronizada por idade também ficou estável, com leves aumentos de 4,0% e 3,8%, respectivamente ( Tabela 5-1 ). ^200^
- Em contraste com as taxas padronizadas por idade, a prevalência bruta mostrou aumento significativo de 89,4% (II 95%, 85,3 – 93,8) de 1990 [131,9 (II 95%, 126,7 – 137,5)] a 2017 [249,9 (II 95%, 239,8 – 260,1)] por 100 mil ( Tabela 5-1 e Figura 5-2.B ). O aumento foi homogêneo para homens e mulheres, sugerindo que a prevalência esteja aumentando desproporcionalmente nas faixas de idade mais avançada (Figura 5-2.B). ^200^
- De modo diferente da doença valvar mitral, a doença valvar aórtica é predominantemente degenerativa ou calcífica. Estudos observacionais mostraram que a estenose aórtica é vista em 4,5% da população com idade superior a 75 anos em países de alta renda, como os Estados Unidos. ^204^ De acordo com estudos observacionais ^197 , 205^ e dados do GBD 2017, ^200^ no Brasil, assim como no resto do mundo, tem-se observado uma tendência para aumento da doença valvar aórtica degenerativa em comparação a de outras etiologias, como DCR.
- Portanto, o aumento na prevalência de DVNR em todas as idades tem sido ‘puxado’ principalmente pela doença valvar aórtica calcífica [114,2% (II 95%, 105,5 – 124,3)], em especial para os grupos mais idosos ( Tabela 5-2 ), mas as taxas também têm sido significativas para doença valvar mitral degenerativa, 79,8% (II 95%, 78,3 – 81,1), e outras DVNR, a despeito da limitada qualidade dos dados sobre as últimas ( Tabela 5-1 ). A prevalência proporcional de DVNR também aumentou significativamente em ambos os sexos de 1990 a 2017 ( Figura 5-2.C ). ^200^
- Contrário ao observado para a etiologia reumática, houve aumento de prolapso de valva mitral como etiologia de regurgitação mitral primária no Brasil: embora na população geral ele atinja taxas de 1% a 2,5% com bom prognóstico na maioria dos casos, entre os pacientes admitidos com doença valvar cardíaca em uma emergência brasileira em 2009 (56±17 anos, 54% mulheres), 13% apresentavam prolapso de valva mitral como etiologia. ^196^ Inversamente, em um registro hospitalar de cirurgia cardíaca de uma das maiores capitais do país (Salvador), de 2002 a 2005, apenas uma pequena proporção de casos foi associada a prolapso mitral. ^193^ Esse resultado é semelhante aos de um estudo com 78.808 pacientes usando 2 grandes bases de dados nacionais (o Sistema Brasileiro de Informações Hospitalares e o Sistema Brasileiro de Informações sobre Mortalidade) de 2001 a 2007, em que apenas 0,24% (187) dos casos informavam prolapso mitral como causa básica. ^206^ Entretanto, os dados podem apresentar viés em razão da ausência de atribuição de código para as etiologias de doença valvar cardíaca no sistema público de saúde e na maior parte do sistema privado de saúde.

### Incidência

- De acordo com um estudo baseado em dados hospitalares no Nordeste do Brasil, de 2002 a 2005 (1.320 cirurgias), a incidência anual média de cirurgia valvar cardíaca foi 4,75 por 100 mil residentes e positivamente associada com a idade. As incidências anuais médias de DCR e doença valvar degenerativa foram 2,86 e 0,73 por 100 mil de população, respectivamente. ^193^

#### Doença Cardíaca Reumática

- Para a DCR, a incidência específica por idade seguiu uma distribuição bimodal de acordo com a fonte do reembolso da cirurgia, aumentando quase que linearmente em 1 caso por 100 mil de população para cada década de vida até a idade de 40–49 anos, com pico em 4,85 casos por 100 mil de população. Após um declínio, um segundo pico ocorreu na faixa etária de 60–69 anos (6,54 casos por 100 mil de população). ^193^
- A incidência de DCR apresentou pequeno aumento de 1,9% (II 95%, 0,8 – 3,1) no Brasil, passando de 21,4 (II 95%, 20,4 – 22,4) por 100 mil em 1990 para 21,8 (II 95%, 20,8 – 22,8) por 100 mil em 2017, de acordo com dados do GBD 2017. Esse pequeno aumento foi relativamente homogêneo em todo o país, com superposição de II 95% mesmo nos estados mais pobres das regiões Norte e Nordeste. ^200^
- Em geral, o aumento na incidência foi ‘puxado’ pelos grupos etários ‘abaixo de 5 anos’, 4,3% (II 95%, 2,5 – 6,2), e ‘5-14 anos’, 2,9% (II 95%, 1,5 – 4,3), enquanto, em indivíduos com idade de 15-49 anos, observou-se redução significativa de 12,1% (II 95%, -13,7 a -10,5). Embora tal padrão seja inesperado, uma vez que, em geral, a incidência cai primeiro nas idades mais jovens e, em seguida, nos adultos jovens, ele pode estar hipoteticamente associado à melhora no diagnóstico precoce e à incorporação de dados de doença subclínica na modelagem GBD. ^2^ ^00^

#### Doença Valvar do Coração Não Reumática

- Em um padrão diferente daquele da DCR, a incidência de DVNR apresentou aumento significativo de 7,9% (II 95%, 6,7 – 9,3), passando de 224,5 (II 95%, 217,5 – 231,6) por 100 mil em 1990 para 242,4 (II 95%, 233,8 – 250,8) por 100 mil em 2017, de acordo com estimativas do GBD 2017. Esse aumento foi ‘puxado’ principalmente pelo aumento de 20,4% (II 95%, 16,0 – 25,4) na doença valvar aórtica calcífica, em especial em indivíduos com idade >70 anos, 37,2% (II 95%, 30,6 – 44,2). ^200^
- Entretanto, a incidência crescente de doença valvar aórtica calcífica também em indivíduos com idade de 15-49 anos, 23,4% (II 95%, 18,1 – 28,7), é atípica considerando-se a epidemiologia da doença, devendo ser interpretada com cautela como uma possível limitação da modelagem GBD, ^200^ pois dados primários para essa causa são escassos no Brasil.

#### Mortalidade

- Doença valvar do coração é uma das principais causas de morte cardiovascular no Brasil, em particular em regiões economicamente desassistidas, e a DCR – a etiologia com maior componente social – ocupou a 8 ^a^ /9 ^a^ posição entre as principais causas nas últimas décadas. ^19^ No cenário mais desassistido, a DCR tem desempenhado importante papel por décadas, com tendência decrescente – nem sempre adequadamente capturada pela modelagem estatística – seguindo melhoria socioeconômica. ^189 , 194 , 206^

#### Doença Cardíaca Reumática

- Contrastando com a tendência crescente de prevalência, as taxas de mortalidade padronizadas por idade atribuíveis à DCR diminuíram significativamente em 50,3% no Brasil, passando de 2,4 (II 95%, 2,3 – 2,5) para 1,2 (II 95%, 1,1 – 1,2) por 100 mil, de acordo com o estudo GBD 2017. A redução percentual foi similar para homens e mulheres ( Tabela 5-3 e Figura 5-3.A ). Observou-se tendência similar para as taxas brutas de mortalidade (Figura 5-3.B). No período, o número total de mortes permaneceu estável [2.648 (II 95%, 2.550 – 2.728) e 2.682 (II 95%, 2.585 – 2.797) em 1990 e 2017, respectivamente], apesar do crescimento da população ( Tabela 5-3 ). Essas tendências podem refletir uma melhoria nas condições de saúde, além de melhor e mais precoce acesso à assistência em saúde. ^207 , 208^
- A mortalidade proporcional atribuível à DCR no Brasil também mostrou um padrão decrescente no mesmo período, com uma tendência menos vertiginosa (Figura 5-3.C). ^207 , 208^
- Em 1990, a DCR ocupou a 9 ^a^ posição entre as causas de morte no Brasil (8 ^a^ ou 9 ^a^ em diferentes estados), tendo passado para a 12 ^a^ posição em 2017 (10 ^a^ a 12 ^a^ na maioria dos estados, permanecendo como 9 ^a^ apenas na Paraíba). ^207 , 208^
- A redução mais significativa nas taxas de mortalidade foi observada nas idades menores, especialmente nos grupos etários ‘abaixo de 5 anos’ e ‘5-14 anos’, -82,5% (II 95%, -87,9 a -74,4) e -69,8% (II 95%, -72,4 a -65,6) por 100 mil, respectivamente (Tabela 5-2). ^207 , 208^ Isso pode estar associado ao melhor tratamento das apresentações precoces e agudas, ainda que as sequelas crônicas persistam sendo um desafio.
- De acordo com dados do GBD 2017, não houve correlação entre as taxas de mortalidade padronizadas por idade e o SDI em 1990 (r ^2^ =0,275, p=0,59) nem em 2017 (r ^2^ =0,233, p=0,83). Entretanto, houve correlação significativa entre a variação percentual nas taxas de mortalidade padronizadas por idade e o SDI em 1990 (Figura 5-4) (r ^2^ =0,073, p< 0,001), que não alcançou significância estatística em 2017 (r ^2^ =0,064, p=0,06). Considerando-se a DCR como a etiologia com maior componente social entre as doenças valvares cardíacas, tal incompatibilidade pode estar associada com: a) diferenças no SDI entre as UF, com padrões socioeconômicos discrepantes nas sub-regiões dos estados, o que poderia enviesar o modelo; b) a variação percentual ao longo das décadas pode representar uma medida mais precisa do aperfeiçoamento do modelo, e a redução no hiato socioeconômico entre as UF brasileiras de 1990 a 2017 pode ter contribuído para reduzir a magnitude da correlação estatística.

#### Doença Valvar do Coração Não Reumática

- De acordo com o estudo GBD 2017, as taxas de mortalidade padronizadas por idade atribuíveis a DVNR permaneceram relativamente estáveis de 1990 a 2017, com um pequeno aumento de 7,1% (II 95%, -9,1 – 13,0) (Tabela 5-3 e Figura 5-5.A). Entretanto, para as taxas de mortalidade brutas, o aumento foi significativo, 87,5% (II 95%, 63,5 – 96,9), com uma considerável contribuição das idades mais avançadas, em especial acima de 70 anos, 45,6% (II 95%, 10,0 – 57,1) (Tabela 5-3 e Figura 5-5.B). Os padrões foram similares para homens e mulheres. Tendências semelhantes foram observadas para as taxas de mortalidade por doença valvar aórtica calcífica, com acentuado aumento de 108% nos idosos (≥70 anos), refletindo associação com envelhecimento populacional (Tabela 5-2). Para a doença valvar mitral degenerativa, as taxas de mortalidade padronizadas por idade diminuíram em 10,8% (II 95%, -47,6 a 2,4), em oposição ao aumento de 50,1% na prevalência bruta (Tabelas 5-1 e 5-3), como resultado das taxas crescentes, 17,7% (II 95%, -42,1 a 39,2), nos septuagenários e mais idosos (Tabela 5-2). ^207 , 20^ ^8^
- As taxas de mortalidade crescentes nas idades mais avançadas por DVNR contrastam bastante com as tendências observadas para DCR, podendo refletir uma maior prevalência e, consequentemente, mortalidade nos grupos etários >70 anos, para DVNR tanto aórtica quanto mitral (Tabela 5-2). De 1990 a 2017, uma carga crescente de doença valvar aórtica calcífica, em homens e mulheres, associou-se com um aumento na mortalidade naquele grupo etário. Os II 95%s são muito amplos em geral para as estimativas de mortalidade por DVNR, em especial para cada doença específica em separado. ^207 , 208^
- A mortalidade proporcional atribuível a DVNR no Brasil mostrou um padrão marcadamente crescente no mesmo período, possivelmente ‘puxada’ pela tendência mais abrupta da doença valvar aórtica calcífica (Figura 5-5.C). ^208^
- Em 1990, a DVNR ocupou a 10 ^a^ posição entre as causas de morte no Brasil (8 ^a^ a 11 ^a^ em diferentes estados), tendo passado para o 9 ^o^ lugar em 2017 (8 ^o^ ao 10 ^o^ na maioria dos estados, permanecendo no 11 ^o^ apenas em Sergipe) (Figura 5-4). ^208^
- Os dados do GBD 2017 demonstraram correlações significativas e fortes entre o SDI e as taxas de mortalidade padronizadas por idade por DVNR em geral em 1990 (r ^2^ =0,62, p=0,002) e 2017 (r ^2^ =0,618, p=0,001), assim como padrão similar para a doença valvar aórtica calcífica (1990: r ^2^ =0,65, p=0,002; e 2017: r=0,65, p< 0,001) (Figura 5-6). Como o desenvolvimento socioeconômico se correlaciona com a transição epidemiológica e a expectativa de vida, um maior SDI está associado com indivíduos mais idosos com risco para condições valvares degenerativas e menos propensos a etiologias infecciosas, como a DCR.
- Além disso, as variações percentuais nas taxas de mortalidade padronizadas por idade de 1990 a 2017 também se correlacionaram com o SDI em 1990 e 2017 para doença valvar aórtica calcífica (1990: r ^2^ =0,17, p=0,005; e 2017: r ^2^ = 0,23, p = 0,003), mas não para DVNR em geral.
- Para a doença valvar mitral degenerativa e outras DVNR, não foram observadas significativas correlações entre o SDI e as taxas de mortalidade padronizadas por idade – e suas variações percentuais ao longo do tempo.

### Carga de Doença

#### Doença Cardíaca Reumática

- De acordo com dados do GBD 2017, a taxa de DALYs padronizada por idade atribuível a DCR diminuiu significativamente em 40% no Brasil, passando de 118,9 (II 95%, 106,1 – 134,7) por 100 mil em 1990 para 76,5 (II 95%, 63,3 – 93,7) por 100 mil em 2017 (Figura 5-7.A). As taxas decrescentes observadas no período foram similares para homens e mulheres, 44,4% (II 95%, 31,2 – 42,3) e 37,9% (II 95%, 31,8 – 44,0), respectivamente (Tabela 5-4). ^209^
- As taxas de DALYs padronizadas por idade diminuíram em todos os estados brasileiros, com tendência mais vertiginosa nas regiões com as mais altas taxas em 1990: Centro-Oeste e Sudeste (Tabela 5-2). A proporção de DALYs mostrou uma tendência mais estável para o Brasil, com discreta redução, embora tenha persistido aumento nas regiões Norte e Nordeste (Figura 5-7.B). As regiões Sudeste e Centro-Oeste apresentaram as mais altas taxas de DALYs padronizadas por idade e DALYs proporcionais no período analisado. ^209^
- Um padrão decrescente similar foi observado para as taxas de YLLs padronizadas por idade por DCR, que variaram de 84,9 (II 95%, 81,1 – 87,8) por 100 mil em 1990 a 36,1 (II 95%, 34,8 – 37,8) por 100 mil em 2017, uma redução de 57,4% (II 95%, 54,8 – 59,4). ^200 , 209^
- As estimativas do GBD 2017 mostraram significativa correlação entre as taxas de DALYs padronizadas por idade e o SDI em 1990 (r ^2^ =0,056, p=0,001) e em 2017 (r ^2^ =0,068, p=0,001). Além disso, houve significativa correlação entre a variação percentual nas taxas de DALYs padronizadas por idade e o SDI em 1990 (r ^2^ =0,03, p< 0,008), embora isso não tenha sido observado em 2017 (r ^2^ =0,019, p=0,146) (Figura 5-8), sugerindo que as áreas menos desenvolvidas socialmente em 1990 – com maior carga de doença – apresentassem mais espaço para melhoria, acompanhando o desenvolvimento social ao longo das décadas.

#### Doença Valvar do Coração Não Reumática

- De acordo com o GBD 2017, as taxas de DALYs padronizadas por idade atribuíveis a DVNR diminuíram discretamente (8,0%) no Brasil, passando de 42,8 (II 95%, 36,6 – 45,8) por 100 mil em 1990 para 39,4 (II 95%, 39,4 – 42,2) por 100 mil em 2017 (Tabela 5-4 e Figura 5-9.A). A discreta redução nas taxas observada no período foi similar para homens e mulheres. Com relação às doenças específicas, as taxas diminuíram mais significativamente para a doença valvar mitral degenerativa, -18,7% (II 95%, -9,3 a -43,4), em comparação à doença valvar aórtica calcífica e a outras doenças valvares, embora os IIs fossem amplos nesse caso. As tendências observadas para os YLLs foram similares. ^200 , 209^
- A tendência relativamente estável foi similar em todos os estados brasileiros e as taxas de DALYs padronizadas por idade permaneceram mais altas nas regiões Sul e Sudeste em todo o período, sendo seguidas pelas regiões Centro-Oeste, Norte e Nordeste (Figura 5-9.A).
- À semelhança do observado para mortalidade, as taxas estáveis de DALYs padronizadas por idade contrastam com o aumento das taxas brutas [38,7% (II 95%, 27,5 – 47,2)] no período, mais uma vez sugerindo que a morbidade associada com DVNR esteja se deslocando para os idosos, possivelmente seguindo mudanças na composição etária da população. ^209 , 210^
- As taxas de DALYs proporcionais no Brasil aumentaram e, de 1990 a 2017, as regiões Sul e Sudeste foram responsáveis pelas maiores proporções de DALYs no período, de acordo com as estimativas do GBD (Figura 5-9.B). ^209 , 210^
- Ainda segundo dados do GBD 2017, houve significativa correlação entre as taxas de DALYs padronizadas por idade por DVNR em geral e o SDI em 1990 (r ^2^ =0,65, p=0,043), mas não em 2017 (r ^2^ =0,49, p=0,067). As variações percentuais nas taxas de DALYs padronizadas por idade (1990 – 2017) correlacionaram-se com o SDI em 1990 (r ^2^ =0,32, p=0,003) e em 2017 (r ^2^ =0,33, p=0,002). Para a doença valvar aórtica calcífica, foram observadas correlações significativas entre DALYs e SDI em 1990 (r ^2^ =0,67, p=0,002) e em 2017 (r ^2^ =0,57, p=0,004) (Figura 5-10), assim como entre as variações percentuais nas taxas de DALYs e o SDI nos dois anos, sugerindo um efeito determinante do desenvolvimento socioeconômico para alguns tipos de DVNR.
- Para a doença valvar mitral degenerativa e outras DVNR, entretanto, não foi observada correlação entre as taxas de DALYs padronizadas por idade – e suas variações percentuais ao longo do tempo – e o SDI, exceto uma fraca associação entre as taxas de DALYs e o SDI em 2017 (r ^2^ =0,08, p=0,02), com base nas estimativas do GBD.

### Complicações e Doenças Associadas

#### Arritmias Associadas com Doença Valvar

- A FA também é um fator complicador para pacientes com doença valvar do coração, ocorrendo, em geral, naqueles com história natural da doença mais avançada. Acha-se mais comumente associada com doença valvar mitral, em especial estenose mitral. A FA foi observada em 34% de uma coorte de 427 pacientes (idade média de 50±16 anos, 84% mulheres) com estenose mitral grave, tendo sido mais frequente naqueles que morreram durante o acompanhamento (27/41, 66%) em comparação aos que sobreviveram (114/378, 30%), reforçando seu papel de marcador de prognóstico na doença valvar cardíaca. ^211^
- A FA também pode se desenvolver na doença valvar aórtica grave, especialmente em pacientes mais idosos e no pós-operatório. Em uma coorte retrospectiva de 348 pacientes (idade média, 76,8±4,6 anos), FA pós-operatória foi observada em 32,8% (n = 114), mas as taxas foram mais altas entre os pacientes com idade a partir de 80 anos (42,9% vs. 28,8% em pacientes com idade de 70-79 anos, p=0,017). ^212^
- Em outra avaliação retrospectiva conduzida em Pernambuco (Nordeste do Brasil), envolvendo 491 pacientes consecutivos após cirurgia por doença valvar do coração, a incidência de FA foi 31,2% e associada com idade >70 anos (OR=6,82; IC 95%, 3,34 – 14,10, p < 0,001), doença valvar mitral (OR=3,18; IC 95%, 1,83 – 5,20, p< 0,001) e a não utilização de betabloqueadores no período peroperatório, entre outros fatores. ^213^
- Doença valvar do coração (17,5%) e arritmias (FA e *flutter* atrial – 50,7%) foram as principais fontes cardioembólicas para acidente vascular cerebral em estudo que envolveu 256 pacientes (60,2 ± 6,9 anos, 132 homens) na região Sul do Brasil. ^214^

####  Associação entre Doença Valvar do Coração e Doença Arterial Coronariana

- Devido ao elevado risco cirúrgico da combinação de procedimentos valvares e revascularização coronária, é essencial que se reconheça a prevalência de DAC obstrutiva em associação com doença valvar do coração. Estudos mostraram menor prevalência de DAC em pacientes com DCR em comparação àqueles com DVNR, possivelmente como reflexo da menor mediana de idade dos pacientes com DCR e da maior prevalência de fatores de risco coronariano na DVNR. ^215^
- Em estudo no Rio de Janeiro incluindo 1.412 candidatos a cirurgia cardíaca por qualquer indicação, 294 casos com doença cardíaca valvar primária de etiologia reumática e não reumática foram selecionados. Todos os pacientes tinham idade ≥40 anos e foram submetidos a angiografia coronária. As prevalências de DAC em pacientes com DCR e DVNR foram 4% e 33,6% (p < 0,0001), respectivamente. Características e fatores de risco, como idade, dor precordial típica, hipertensão, diabetes mellitus e dislipidemia, foram significativamente associados com DAC obstrutiva. ^216^
- Em outro estudo no Brasil avaliando 712 pacientes com doença valvar do coração e idade média de 58±13 anos, a incidência de DAC obstrutiva foi 20%. Entretanto, em pacientes mais jovens (<50 anos), a prevalência foi mais baixa (3,3%). ^217^ Esses dados são similares aos observados em outro estudo que incluiu 3.736 pacientes (idade média, 43,7 anos), nos quais a prevalência de DAC obstrutiva combinada com doença valvar do coração foi 3,42%. ^215^

### Utilização e Custo da Atenção à Saúde

#### (Ver Tabelas 1-6 a 1-9 e Figuras 1-15 e 1-16)

- De acordo com a base de dados administrativos do SUS, o total de despesas brutas (reembolso) com hospitalização para tratamento clínico de doença valvar do coração no Brasil aumentou significativamente em 94%, passando de R$ 1.051.959,34 em 2008 para R$ 2.043.358 em 2018, em um padrão quase linear. Ajustando e convertendo esses valores para dólares internacionais de 2018, o total de custos no sistema público de saúde para hospitalização por condições valvares foi de $ 1.014.294 em 2008 e de $ 1.007.587 em 2018.
- Da mesma forma, os custos não ajustados associados com procedimentos cirúrgicos/intervencionistas valvares (códigos relacionados com cirurgia valvar, comissurotomia mitral percutânea e outros tipos de valvuloplastia) também aumentaram de 2008 a 2018, passando de R$ 130.588.598 (2018Int$ 125.912.942) para R$ 180.735.108 (2018Int$ 89.119.874), embora com menor magnitude em comparação àqueles de internações clínicas (94% vs. 38%). ^201^
- O número de internações cirúrgicas/intervencionistas relacionadas às doenças valvares não cresceu muito no Brasil de 2008 a 2018, variando de 13.129 em 2008 a 14.294 em 2018. Isso está provavelmente associado com a complexidade e os custos crescentes das intervenções (em especial, dispositivos e próteses) e denota a carga econômica imposta pela incorporação de novos procedimentos e tecnologias, além do efeito marcante da inflação sobre os custos da atenção à saúde, considerando-se valores ajustados para Int$. Nesse cenário, a futura incorporação de terapias bem estabelecidas ainda não reembolsadas pelo SUS, como a TAVI, contribuirá para aumentar a carga econômica, embora as despesas com demandas judiciais possam exceder os custos ordinários. ^218^
- O número total de internações no período foi de 172.126, tendo a maioria ocorrido na região Sudeste (41,2%), seguida pelas regiões Nordeste (25,7%), Sul (20,2%), Centro-Oeste (7,5%) e Norte (5,4%). ^201^
- Observou-se queda dramática em alguns tipos de procedimentos, a despeito de suas crescentes indicações, como a comissurotomia mitral percutânea. Especificamente para esse procedimento, os números decrescentes podem estar associados com o atraso das tabelas de reembolso do SUS, limitando o número de hospitais que realizam aquela intervenção. O número absoluto de cirurgias valvares abertas permaneceu estável no período, variando de 12.201 (2008) a 12.088 (2018), a despeito do crescente número de casos de doença valvar do coração – em especial DVNR – e da crescente carga para os idosos. ^200 , 201^
• Em nenhum dos períodos, o aumento no número de internações encontrou paralelo nas despesas crescentes, sugerindo uma progressiva complexidade – e, consequentemente, custo – dos procedimentos para tratar doença valvar cardíaca. ^201^
- A partir da base de dados administrativos do SUS, os procedimentos valvares associados com sequelas de DCR não puderam ser diferenciados daqueles associados com outras etiologias, devido à ausência de código específico disponível, além da imprecisão da notificação dos códigos da CID. ^201^
- É interessante notar que estudos observacionais enfatizaram que a DCR permanece sendo a principal etiologia associada com cirurgia cardíaca em jovens no Brasil, atingindo até 60% em um estudo realizado em Salvador, Bahia. ^193^ No Instituto do Coração de São Paulo, o número de cirurgias cardíacas valvares associadas com DCR aumentou substancialmente nos últimos 10 anos, de cerca de 400 cirurgias/ano em 1990 para mais de 600 após 2000. ^219^ Entre 2008 e 2015, houve 26.054 hospitalizações por sequelas de FRA, 45% das quais por doença cardíaca, levando a um custo total possivelmente subestimado de US$ 3,5 milhões ao ano. ^192 , 201^
- Em geral, as doenças valvares de origem reumática são responsáveis por cerca de 90% das cirurgias cardíacas em crianças e por mais de 30% das cirurgias cardíacas em adultos, a maioria jovem, ^220^ ainda de acordo com estudos observacionais e registros de base hospitalar. Entretanto, poucos estudos epidemiológicos estimaram a carga da doença valvar por causa específica no Brasil.

#### Doença Valvar Mitral

- Com base em dados administrativos do SUS de 2001 a 2007 e referentes a cirurgia de valva mitral, em uma série retrospectiva de 78.808 pacientes cirúrgicos consecutivos, a idade média foi de 50,0 anos (35,9 – 62,5) e 40.106 eram mulheres (50,9%). Novamente a DCR foi a principal etiologia, responsável por 53,7% do total de pacientes submetidos a cirurgia e a mais de 94% dos pacientes submetidos a procedimentos para estenose mitral, que representou a maior indicação cirúrgica única, responsável por 38,9% do total. No geral, a substituição valvar foi realizada em 69,1% das cirurgias. A mortalidade hospitalar foi 7,6%. ^206^
- A mortalidade cirúrgica foi discretamente maior em mulheres do que em homens (7,8% vs. 7,3%; p < 0,001) e consideravelmente mais alta nos indivíduos com idade ≥80 anos. Por outro lado, a mais baixa mortalidade foi observada naqueles entre 20 e 39,9 anos (p < 0,001). Os pacientes com cirurgias aórtica e mitral combinadas (refletindo etiologia reumática) foram os mais jovens (mediana, 43,3 anos). A cirurgia para estenose aórtica foi mais comum em indivíduos mais idosos (mediana, 58,0 anos) (p < 0,001). Reparo valvar apresentou menor mortalidade (3,5%) em comparação a substituição valvar (6,9%), reparo/substituição valvar múltipla (8,2%) e CRVM concomitante (14,6%) (p< 0,001). Associação com CRVM ocorreu em 7.147 pacientes (9,1% da amostra). ^206^
- Quanto à comissurotomia percutânea, estudos no Brasil mostraram uma proporção muito maior de mulheres (85%) – coincidente com a epidemiologia da DCR e principalmente da estenose mitral – e de jovens (<40 anos). ^221 , 222^

#### Doença Valvar Aórtica

- Uma coorte de 724 pacientes consecutivos, submetidos a cirurgia cardíaca no Instituto do Coração de São Paulo, evidenciou, à semelhança de outros estudos, maior taxa de mulheres (55%) e predominância de DCR (60%). Entretanto, nessa série, houve grande proporção de doença valvar aórtica (396 casos) em comparação a doença valvar mitral (306 casos) e outras séries. Dos pacientes com doença valvar mitral, 39,9% apresentavam estenose, 38,4% apresentavam regurgitação e 21,7% apresentavam disfunção de prótese mitral. Em pacientes submetidos a intervenção valvar aórtica, observaram-se estenose em 51,6%, regurgitação em 29,3% e disfunção de prótese em 19,1%. O estudo sugere um aumento de doença valvar aórtica em comparação a doença valvar mitral em hospital terciário da região Sudeste do Brasil. ^223^
- Outro estudo retrospectivo de coorte foi conduzido em Porto Alegre (Sul do Brasil), com 1.065 pacientes (idade média, 61,4 ± 11,8 anos; 38% mulheres). Substituição de valva aórtica foi realizada em 18,8% e de valva mitral, em 13,4%. Revascularização coronária concomitante foi realizada em 60,3% da amostra e cirurgias valvares, em 32,7%. A mortalidade hospitalar geral foi 7,8%, sendo menor para CRVM isolada (5,9%), intermediária para cirurgia valvar (aórtica e/ou mitral e/ou tricúspide = 8,6%) e maior para a combinação de procedimentos valvares e CRVM (20,0%). ^224^

#### Implantação Percutânea de Valva Aórtica no Brasil

- Como em outros países, a TAVI ganhou importância no Brasil nos últimos 20 anos. Estima-se que mais de 100 mil TAVI já foram realizadas no mundo. ^205 ,2018 ^ A primeira TAVI no Brasil ocorreu em 2008. O registro brasileiro de TAVI relatou a realização de 418 TAVI em 18 centros até 2014, tendo esse número crescido exponencialmente desde então. O acesso femoral foi escolhido em 96,2% dos procedimentos e as próteses usadas foram CoreValve ^®^ (86,1%) e Sapien XT ^®^ (13,9%). Nessa experiência inicial, as mortalidades por todas as causas em 30 dias e 1 ano foram 9,1% e 21,5%, respectivamente. ^225^
- Dados do registro de TAVI atualizados em 2017 revelaram um total de 819 pacientes em acompanhamento clínico, demonstrando que o procedimento tem baixa incidência de complicações – em especial desfechos clínicos duros precoces – e ressaltando taxas de insuficiência renal pós-procedimento em torno de 18%. ^226 , 227^
- Em outra avaliação realizada na cidade do Rio de Janeiro com 136 pacientes submetidos a TAVI [mediana de idade, 83 (80-87) anos; 51% homens], a mortalidade peroperatória foi 1,5%, a mortalidade de 30 dias, 5,9%, a mortalidade hospitalar, 8,1% e a mortalidade de 1 ano, 15,5%. ^228^
- De 819 valvas aórticas implantadas por via percutânea até 2017, 135 pacientes (20,1%) precisaram de colocação de marcapasso permanente. Esses pacientes eram mais velhos (82,5 vs. 81,1 anos; p=0,047), predominantemente homens (59,3% vs 45%; p=0,003) e tinham bloqueio de ramo direito prévio (OR=6,19; IC 95%, 3,56 – 10,75, p≤0,001). O uso da prótese CoreValve ^®^ (OR=3,16; IC 95%, 1,74 – 5,72, p≤0,001) e gradiente transaórtico basal >50 mmHg (OR=1,86; IC 95%, 1,08 – 3,20, p=0,025) foram preditores independentes de implantação de marcapasso permanente. ^227^

### Pesquisa Futura

- Apesar da notável melhora nas últimas décadas, ainda há escassez de dados primários sobre a epidemiologia da doença valvar do coração no Brasil e muito espaço para pesquisa futura. Há alguns desafios administrativos para a coleta de dados e o desenvolvimento de registros no âmbito nacional associados com a codificação de hospitalizações e procedimentos. Em especial no SUS, os códigos atuais não permitem a discriminação de variáveis cruciais, como a valva envolvida, o tipo de disfunção valvar, o tipo de prótese e, especialmente, a etiologia e a associação com doenças sistêmicas. Portanto, o refinamento do sistema de codificação ou a implementação mandatória de relatórios clínicos e cirúrgicos – como feito anteriormente para as intervenções coronárias percutâneas – podem ser um passo inicial para melhorar a acurácia na aquisição de dados.
- Como o país tem algumas coortes significativas de pacientes com doença valvar do coração, o acompanhamento de médio e longo prazo dessas amostras deve ser garantido. Importante notar que há iniciativas de pesquisa que requerem incentivos e financiamento para sua continuação, como os estudos em andamento sobre prognóstico de longo prazo de DCR subclínica em crianças e adolescentes, ^191 , 203^ determinantes genéticos e imunes da resposta às infecções estreptocócicas que leva a DCR, ^229^ preditores clínicos e relacionados a procedimento de eventos de curto e longo prazo após comissurotomia mitral percutânea, ^211 , 230^ além de um registro nacional de TAVI. ^225^
- Com relação ao rastreio com ecocardiografia para DCR, dados primários sugerem que a estratégia parece ser custo-efetiva no Brasil, ^231^ mas sua aplicação fora da pesquisa e integração nos sistemas de saúde precisam de esforços investigativos continuados, à semelhança de outros países. Um escore de risco ecocardiográfico para prever a progressão da DCR foi obtido a partir de uma coorte brasileira, ^232^ mas aguarda validação mais ampla em outros cenários. Além disso, esforços continuados foram direcionados para o desenvolvimento de vacinas contra infecções estreptocócicas, ^229^ devendo-se garantir estudos colaborativos sobre sua eficácia e aplicação clínica para reduzir a carga de DCR. No oposto do espectro da doença valvar do coração, a incorporação da TAVI no SUS parece próxima, ^232^ sendo que a avaliação do seu verdadeiro impacto clínico, orçamentário e social nos desfechos da atenção à saúde pública requer extensa pesquisa.
- Por fim, outra estratégia promissora para fornecer diagnóstico precoce e priorizar referências nas áreas de poucos recursos deve ser investigada no Brasil. Como exemplo, a disponibilidade de modalidades de exames de imagem para o manejo da doença valvar do coração – em especial ecocardiografia – é limitada e distribuída de maneira desigual no país. Nesse cenário, avaliou-se a implementação de tele-ecocardiografia, com a mudança da tarefa de aquisição de imagem para não médicos (ainda não permitida fora da pesquisa pela regulamentação da atenção à saúde brasileira) e sua leitura remota. ^233^ Apesar do seu bom desempenho para diagnóstico e discriminação de pacientes com alto risco cardiovascular, ^234^ o impacto sobre os desfechos clínicos, assim como a viabilidade e a custo-efetividade da estratégia, ainda aguardam avaliação. À semelhança do observado para outras novas modalidades, como tele-ECG, rastreio de FA ^235^ e consultas remotas, a incorporação das inovações em técnicas de imagem para melhorar o acesso ao cuidado cardiovascular no Brasil pode requerer extensas discussões baseadas em sólida evidência científica.

**Tabela 5-1:** – Taxas de prevalência padronizadas por idade de doença valvar do coração por 100 mil, e variação percentual das taxas, para ambos os sexos e para homens e mulheres, no Brasil e unidades federativas, 1990 e 2017

Causa de morte e grupo etário	Ambos os sexos			Mulheres			Homens		
	1990	2017	Variação percentual (II 95%)	1990	2017	Variação percentual (II 95%)	1990	2017	Variação percentual (II 95%)
***B.2.1 - Doença cardíaca reumática***									
Acre	703.8 (665.8;739.9)	729.7 (693.1;768.5)	3.7 (7.7;-0.3)	775 (730.9;820.8)	815.5 (768.6;865.4)	5.2 (11.2;0.2)	637.6 (602;673.8)	643.2 (608.9;683.8)	0.9 (6.3;-4.6)
Alagoas	716 (680.1;754.7)	742.7 (704.3;784.6)	3.7 (8.3;-0.2)	792.6 (748;840.8)	831.5 (783;883.6)	4.9 (11.4;-0.9)	633.3 (596;671.4)	644.2 (604.8;683.5)	1.7 (8.5;-4.2)
Amapá	717.6 (682.8;756.8)	738.1 (699;779.4)	2.9 (7;-1.2)	788 (746;835.3)	825.5 (778.8;872.7)	4.8 (10.6;-0.4)	647.3 (612.4;686.3)	649.1 (611.5;689.5)	0.3 (6;-5.3)
Amazonas	725.7 (690.9;766)	745.4 (701.9;787.1)	2.7 (6.6;-1.4)	806.5 (761.8;856.7)	837.9 (785.6;893.9)	3.9 (9.7;-2.3)	646.3 (609.2;688.4)	652.9 (614.4;690.3)	1 (7.2;-5.6)
Bahia	698.3 (664.2;739.1)	726.2 (689.5;765.3)	4 (7.7;-0.1)	764.8 (722.7;814.7)	809.5 (763.4;859.3)	5.8 (11;0.6)	627.7 (589.6;669.6)	637.3 (602.1;676.4)	1.5 (7.7;-4.5)
Brasil	721.4 (688.7;754.5)	743.2 (709;778.6)	3 (4.3;1.6)	797 (758.9;835.7)	832.2 (793.3;871.2)	4.4 (6.1;2.6)	641.9 (611.4;672.8)	648.9 (619;682.4)	1.1 (2.8;-0.7)
Ceará	721.8 (682.3;759.9)	747.5 (708.2;785.5)	3.6 (7.4;0)	798.7 (753;847.5)	838 (787.6;887.3)	4.9 (10.2;-1)	636.7 (596.7;676)	649.9 (611.1;688.1)	2.1 (7.8;-3.5)
Distrito Federal	727.1 (689.8;767.7)	744.2 (705.1;781.2)	2.3 (6.5;-1.6)	802.3 (758.5;851.6)	831.2 (783.8;881.5)	3.6 (9.6;-1.8)	643.2 (607.9;683.5)	646.7 (609.8;685.7)	0.5 (6.4;-5.4)
Espírito Santo	723.1 (686.9;761.9)	743.5 (705.8;783.6)	2.8 (7.2;-1.2)	802.6 (759.6;849.1)	834.1 (785;885.2)	3.9 (9.9;-1.8)	642.2 (602.5;682.4)	648.9 (611;692.3)	1 (7.6;-4.8)
Goiás	712.2 (676.2;749.8)	731.3 (695.6;769.8)	2.7 (6.3;-1.3)	785 (744.4;829.9)	818.3 (772.2;869.3)	4.2 (10.6;-1.6)	640.6 (605.3;678.1)	641.7 (605.2;680.2)	0.2 (6;-5.5)
Maranhão	697.8 (662.7;735.1)	723.3 (686.3;761.1)	3.7 (8.2;0.2)	763.4 (720.7;810.6)	808.1 (761.7;855.2)	5.9 (13;0.7)	629.9 (594.6;666.9)	634.3 (598.3;671.6)	0.7 (6.9;-5.1)
Mato Grosso	718.2 (679.8;759.1)	741.6 (703.4;783)	3.3 (7.8;-1.2)	803.2 (754.4;856.1)	835.7 (789.3;887)	4 (10.1;-2.3)	642.9 (604;683.2)	650.5 (613.5;691.9)	1.2 (7.8;-4.9)
Mato Grosso do Sul	721.6 (682.2;762.9)	743.9 (707;786.6)	3.1 (7.1;-1)	801.8 (755.3;852.3)	836.1 (787.1;887.1)	4.3 (10.1;-1.4)	644.1 (604.1;685.3)	649.6 (613.7;693)	0.9 (7.1;-5.4)
Minas Gerais	724.4 (685;763.2)	746.6 (707;789.2)	3.1 (7.3;-0.8)	801.8 (752.5;848.6)	838 (791;889.4)	4.5 (10.1;-0.9)	643.6 (607;682.9)	651.3 (611.4;692.4)	1.2 (7.5;-5.5)
Pará	697.5 (659.7;733.9)	725.6 (687.9;766.2)	4 (7.8;0.4)	764.8 (718.8;811.3)	812.4 (764.3;861.1)	6.2 (13.1;0.3)	632 (596.3;670.2)	638.8 (601.1;676.1)	1.1 (6.7;-4.3)
Paraíba	722.5 (685.6;764.3)	745.9 (708;784.5)	3.2 (7.7;-0.7)	798.1 (752.6;848.6)	835.4 (788.5;885.1)	4.7 (11.6;-0.4)	637.5 (601.1;676.8)	648.6 (608.5;687.3)	1.7 (7.7;-3.9)
Paraná	726.1 (688.1;765.9)	749 (710.7;789)	3.2 (7.1;-0.8)	805.2 (761.2;853.1)	839.6 (789.9;892.7)	4.3 (10.3;-1.9)	645.8 (608.5;686.5)	653.7 (616.8;695.5)	1.2 (7;-4.8)
Pernambuco	698 (662.3;735)	725.4 (688.9;764.7)	3.9 (8.4;0.2)	765.2 (722.9;809.1)	807.6 (762;853.5)	5.6 (11.7;0)	622.3 (583.7;661.9)	634.3 (597.7;673.3)	1.9 (7.9;-3.3)
Piauí	695.7 (660.7;733)	728.9 (691.5;769.8)	4.8 (9.2;0.6)	760.9 (719.6;805.7)	812.4 (765.2;862.7)	6.8 (13.6;0.6)	625.6 (591.2;663.8)	639.7 (605.1;678.2)	2.3 (8.4;-3.5)
Rio de Janeiro	724 (687.3;761.5)	744.2 (706;786.6)	2.8 (6.7;-1.2)	798.6 (752.6;845.5)	832.1 (784.7;881)	4.2 (10.3;-2.1)	641 (605.8;678.1)	647.6 (609.7;690.4)	1 (7.3;-5.1)
Rio Grande do Norte	728.2 (691.7;770.1)	748.7 (710;789.2)	2.8 (7;-1.5)	807.1 (762.2;859.6)	840.3 (789.7;889.8)	4.1 (10.7;-2.5)	641.6 (603;682.9)	650.7 (611.7;691.2)	1.4 (7.9;-4.9)
Rio Grande do Sul	736 (696.2;776.3)	751.4 (711.4;791)	2.1 (6.2;-2.1)	814.7 (768.3;865.5)	842.7 (791.8;893.9)	3.4 (10.5;-2.6)	652.1 (609.9;690.5)	654.9 (615.9;693.4)	0.4 (6.3;-5.9)
Rondônia	715.4 (677.7;753.8)	739.2 (700.9;778.6)	3.3 (7.5;-0.1)	801.5 (755.8;853.8)	833 (785.4;884.3)	3.9 (10.5;-2)	640.7 (601.2;677.1)	647.5 (608.9;685.7)	1.1 (6.9;-4.3)
Roraima	708.8 (672.4;748.4)	737.8 (697.7;780.1)	4.1 (8.6;0)	798.3 (751.2;847.9)	831.4 (782.3;881.2)	4.1 (11;-2.3)	641.5 (604.8;680.2)	648.6 (610.6;689.5)	1.1 (7.1;-4.4)
Santa Catarina	738 (700.8;775.2)	756.4 (714.4;799.4)	2.5 (6.3;-0.9)	819.4 (771.8;869.5)	849.2 (797.9;900.5)	3.6 (9.8;-1.9)	655.2 (616;694.9)	661.3 (623.1;702.9)	0.9 (7.2;-4.3)
São Paulo	733.8 (694.9;775.3)	753.5 (713;794.1)	2.7 (6.6;-1.4)	813.1 (765.5;864.3)	844.5 (793.5;897.4)	3.9 (9.9;-1.9)	650.5 (611.9;690)	656.5 (615.6;697.4)	0.9 (7.4;-5)
Sergipe	709.6 (673;747.4)	733.5 (695.4;772.8)	3.4 (7.9;-1.2)	776.6 (733.6;822)	816 (769.1;870.3)	5.1 (11.1;-0.7)	636.9 (599.1;673.6)	642.7 (606.2;680.8)	0.9 (7.8;-5.4)
Tocantins	694.9 (660.5;733.7)	726.2 (688.6;767.1)	4.5 (8.4;0.7)	763.1 (720.4;810.2)	813.3 (767.8;861.3)	6.6 (12.5;1.4)	631.9 (596.2;668.5)	640.4 (600.7;679.6)	1.4 (6.8;-3.8)
***B.2.5 - Doença valvar do coração não reumática***									
Acre	205.7 (195;216.6)	224.6 (212.8;236.5)	9.2 (15.3;3.5)	204.6 (191.3;218.3)	222.1 (207.8;236)	8.6 (17.7;0.3)	206.4 (192.7;219.8)	227.3 (212.2;242.4)	10.2 (18.6;2.2)
Alagoas	196.1 (185.1;207.4)	217.3 (205.8;228.9)	10.8 (17.5;4.6)	196.3 (183;210.4)	215 (200.9;228.9)	9.5 (18.8;1.4)	196.1 (181.9;211.2)	220.6 (205.6;236.6)	12.5 (22.1;3.6)
Amapá	215 (204.2;227)	232 (218.9;243.8)	7.9 (13.9;2.2)	213.9 (200.3;228.3)	228.1 (213.2;242.2)	6.7 (15.1;-1.6)	216.6 (202.3;232)	236.6 (220.5;252.2)	9.3 (18.6;1)
Amazonas	212.8 (201.5;223.1)	231.9 (219.4;244.2)	9 (15.1;3)	210.1 (196.7;223.6)	227.8 (212.6;242.9)	8.4 (17.1;0.2)	215.8 (201.1;229.7)	236.5 (219.9;252.6)	9.6 (18.2;1.5)
Bahia	203.9 (192.4;214.8)	221.9 (210;234.1)	8.9 (15.5;2.6)	202.4 (187.9;216.5)	219 (204.8;234.2)	8.2 (17.9;-0.8)	205.7 (191.5;219.8)	226 (211;241.8)	9.9 (18.8;1.7)
Brasil	216.2 (207.9;224.6)	233.9 (224.6;243.2)	8.2 (10.5;6.2)	214.2 (205.6;222.8)	231 (221.7;240.5)	7.8 (10.8;5.1)	218.7 (209.3;228.2)	238.1 (227.4;249.1)	8.9 (12.1;5.8)
Ceará	199.9 (188.5;211.5)	220.7 (208.5;232.9)	10.4 (17.2;4.2)	200 (187;213.9)	219.1 (205;233.9)	9.5 (19.2;1.3)	199.9 (184.7;215.4)	223.3 (207.3;237.7)	11.7 (21.3;2.9)
Distrito Federal	240.9 (227.1;253.5)	258.6 (245.2;271.6)	7.3 (13.1;1.7)	237.1 (221.3;252.1)	254.1 (237.7;270.7)	7.2 (15.7;-0.9)	245.8 (228.3;263.5)	265.5 (248;282.5)	8 (16.6;0.2)
Espírito Santo	220.3 (209.2;233)	236.8 (225.2;250)	7.5 (13.5;1.8)	218 (204.7;233)	233.5 (218.6;248.8)	7.1 (16.2;-1.2)	222.9 (209.1;238.9)	241.2 (225.8;257.4)	8.2 (16.8;0)
Goiás	212.7 (201.9;224.4)	229.7 (217.4;242)	8 (14.4;2.2)	212.7 (197.9;227.3)	227.2 (212.7;241.9)	6.8 (15.8;-1.3)	212.6 (198.6;227.5)	232.8 (216.2;248.7)	9.5 (18.5;1.1)
Maranhão	195.1 (184.6;205.8)	214.5 (202.2;227.9)	9.9 (16.4;3.9)	194.9 (182.5;208.7)	211.6 (197;225.9)	8.6 (17.4;0.3)	195.7 (181.7;209)	218.1 (203.1;233.7)	11.4 (21.2;2.3)
Mato Grosso	214.7 (203.5;226)	233.1 (220.6;245.6)	8.6 (15.4;2.5)	212.6 (198.1;226.8)	229.7 (215;245)	8.1 (17.4;-0.4)	216.3 (202.2;231.3)	236.5 (220.9;252.5)	9.3 (18.2;1.2)
Mato Grosso do Sul	216.7 (204.9;228.2)	233.5 (220.7;246)	7.8 (14.1;1.8)	214 (199.5;227.8)	229.7 (214.8;244.4)	7.4 (16.6;-0.6)	219.1 (203.9;233.4)	238 (222.4;255.5)	8.6 (17.5;0.1)
Minas Gerais	214.7 (203.5;226.9)	232 (220.1;244.2)	8 (14.2;2.1)	212.3 (199.1;227)	228.8 (214.3;243.2)	7.8 (16.3;-0.6)	217.9 (203.8;233.2)	236.2 (220.7;253.4)	8.4 (17;0.1)
Pará	201.6 (191;213)	221.3 (209.1;233.7)	9.8 (16;3.7)	200.7 (187.3;215.1)	218 (202.3;232.6)	8.7 (17.9;0.4)	202.9 (189.7;217.7)	225 (210.3;240.2)	10.9 (19.4;2.5)
Paraíba	199.4 (188.2;210.9)	219.5 (207.7;231.3)	10.1 (16.1;4)	197.7 (183.8;211.4)	217.2 (203.3;232)	9.8 (19.2;1.6)	201.2 (186.2;216.6)	222.9 (207;238.5)	10.8 (19.7;2.8)
Paraná	223.8 (211.7;236.2)	239.7 (228;253)	7.1 (13.1;1.3)	220.7 (207.1;235.2)	236.4 (222;251.8)	7.1 (16.1;-0.8)	227.1 (212.7;243)	244.1 (228.7;261.2)	7.5 (15.9;-0.7)
Pernambuco	204.2 (193.4;215.1)	223 (209.8;235.2)	9.2 (15.5;3.2)	203.7 (190.6;217.2)	221.1 (206.2;236.5)	8.5 (17.1;0.3)	205 (191.8;220.3)	226.3 (210.5;241.5)	10.4 (20.1;2.5)
Piauí	197.2 (186.5;208)	215.7 (204.1;228)	9.4 (16;3)	196.4 (183.2;208.8)	213.2 (199.2;227.8)	8.5 (16.9;0.4)	198.3 (184;212.8)	219 (204.6;235.2)	10.5 (20.3;1.6)
Rio de Janeiro	220.5 (209.3;232.1)	238.3 (224.6;250.7)	8.1 (14.4;2)	219 (204.4;233.4)	235.5 (219.4;250.4)	7.5 (16.6;-1.2)	223.5 (209.1;239)	243.1 (227.6;261)	8.8 (17.2;0.8)
Rio Grande do Norte	203.5 (193;214.7)	223.7 (211.6;235.9)	9.9 (16.1;3.6)	202.9 (188.7;216.4)	221.3 (206;235.7)	9.1 (18.1;0.7)	204.2 (190.3;218.4)	227.3 (212.4;243.5)	11.3 (20.2;3.2)
Rio Grande do Sul	227.5 (215.2;239.3)	241.9 (229.2;254.8)	6.3 (12.6;0.6)	224.4 (208.7;239.5)	237.8 (223;253.3)	6 (14.5;-2)	232.2 (216.5;247.3)	247.8 (231.7;265.4)	6.7 (15.7;-0.9)
Rondônia	209.2 (197.9;220.7)	227.1 (215;239.8)	8.6 (15.4;2.6)	205 (190.9;219)	223.9 (208.6;239.1)	9.2 (18.7;0.9)	212.3 (197.7;226.5)	230.2 (214.3;246.5)	8.4 (17.7;0.1)
Roraima	212 (200;223.9)	229.5 (217;242.8)	8.3 (14.4;2.4)	212.3 (198.8;227.8)	226.9 (211.8;242.6)	6.9 (15.5;-1.2)	211.9 (197.6;226.9)	231.9 (216.3;247.8)	9.4 (18.6;1.3)
Santa Catarina	228.3 (215.6;240.1)	242.5 (229.2;256.5)	6.2 (11.8;0.5)	225.6 (210.8;239.8)	238.7 (223.3;255.3)	5.8 (14.4;-1.9)	231.7 (216.6;248.1)	247.8 (230.8;264.6)	6.9 (15.7;-1.1)
São Paulo	229.8 (217.3;241.6)	244.9 (232.6;258.6)	6.6 (12.6;1.1)	226.1 (211;239.7)	241.7 (225.7;257.7)	6.9 (15.2;-0.9)	234.9 (219.1;251.4)	250 (232.9;267.1)	6.4 (15.1;-1.3)
Sergipe	201.1 (190.2;212.8)	223.1 (210.6;235.4)	11 (17.7;5)	201.1 (187.9;215.2)	220.7 (205.4;235.6)	9.7 (18.3;1.2)	201.2 (187.4;215.7)	226.7 (211.9;242.3)	12.6 (21.7;3.9)
Tocantins	206.2 (194.6;217)	223.1 (210.7;236)	8.2 (14.6;2)	204.3 (190.8;218.4)	219.4 (203.7;234.3)	7.4 (16.5;-1.2)	207.8 (193.3;221.3)	226.6 (210.1;242.4)	9 (17.8;1.2)
*** B.2.5.1 - Doença valvar aórtica calcífica não reumática ***									
Acre	49.8 (44.5;55.8)	61.3 (54.1;68.9)	23.1 (31.2;16)	40.7 (36;45.6)	51.9 (45.5;58.4)	27.5 (38.3;18.7)	57.6 (51.3;64.6)	71.1 (62.4;80.2)	23.4 (34.1;14)
Alagoas	43.4 (38.7;49.1)	58.1 (51;66.2)	34 (42.5;26.3)	36.7 (32.4;41.7)	50 (44.1;57.1)	36.2 (46.5;26.7)	50.9 (45;57.6)	68.2 (59.6;77.9)	34 (46.2;23.8)
Amapá	54.6 (48.7;61.6)	65.6 (58.2;74.2)	20 (27.4;13.1)	45.3 (40.3;51.1)	54.7 (48.1;61.5)	20.7 (30.6;12.2)	64.6 (57.1;72.7)	77.6 (68.8;87.9)	20.2 (30.1;11.4)
Amazonas	51.3 (45.9;58)	63.9 (56.7;72)	24.5 (32.1;17.2)	41 (36.3;46.4)	53.1 (46.5;60.3)	29.4 (39.9;20.2)	62 (55.2;70.2)	75.4 (66.5;85.8)	21.7 (31.1;12.9)
Bahia	48.3 (43.1;54.2)	59 (52.4;67)	22.4 (30.1;15.3)	40.4 (35.8;45.5)	50.1 (43.8;57)	24.2 (35;16)	57.3 (50.9;65.1)	70 (61.5;79.3)	22.2 (31.7;13.3)
Brasil	53.5 (48.1;59.9)	64.4 (57.2;72.5)	20.4 (25.4;16)	44.2 (39.8;49.6)	54.9 (48.7;62)	24.2 (29.7;19.1)	64.3 (57.5;72)	76.2 (67.3;85.6)	18.5 (24.2;13.7)
Ceará	46 (40.9;52.2)	59.6 (52.5;67.7)	29.4 (37;22.2)	38.7 (33.9;44.3)	51 (44.8;58)	31.6 (40.6;22.4)	54.3 (47.8;62)	70.2 (61;80.2)	29.3 (39.1;19.6)
Distrito Federal	63.1 (56.5;70.5)	73.3 (64.7;82.1)	16.2 (23.5;9.5)	51.3 (45.7;58)	62.5 (55;70.6)	21.8 (32;12.6)	77.4 (68.7;87.1)	88.2 (77.6;99.8)	13.9 (22.5;5.8)
Espírito Santo	55.5 (49.5;61.9)	64.9 (57.5;73.3)	17.1 (23.9;10.8)	45.5 (40.4;51.3)	55 (48.4;62.4)	20.7 (29.8;12.5)	66.3 (58.9;74.6)	76.9 (67.6;86.9)	16 (24.5;7.8)
Goiás	52.9 (46.9;59.3)	62.4 (55;70.1)	18.1 (25.3;11.4)	44.8 (39.7;50.6)	52.9 (46.6;59.4)	18.2 (27.4;10.7)	60.5 (53.5;67.8)	73.1 (64.3;83)	20.8 (30.4;11.5)
Maranhão	43.9 (38.9;49.6)	57 (50.1;64.9)	29.9 (37.2;22.8)	36.9 (32.3;42.1)	48.5 (42.5;55.5)	31.3 (41.2;22.2)	51.8 (45.9;58.7)	66.8 (58.8;76.5)	29 (38.5;19.4)
Mato Grosso	52.4 (46.7;58.7)	64.1 (56.6;72.4)	22.4 (30;15.2)	41.8 (37.4;47.1)	53.8 (47.6;61)	28.6 (38.8;19.9)	61.3 (54.3;68.9)	74.3 (65.2;84.3)	21.2 (31.3;11.9)
Mato Grosso do Sul	55.3 (49.5;62)	65.6 (58.3;74.3)	18.7 (26.3;11.8)	45.4 (40.4;51.1)	55.4 (48.6;62.5)	21.9 (31;13.7)	64.5 (57.2;72.6)	77 (67.9;87.4)	19.4 (29.9;9.9)
Minas Gerais	51.9 (46.5;58.3)	63.1 (55.9;71.4)	21.6 (29.2;14.7)	42.7 (38.1;48.1)	53.5 (47.2;60.6)	25.4 (34.1;18)	62.7 (55.9;70.9)	74.7 (65.7;84.8)	19 (28.9;9.5)
Pará	46.9 (41.7;53)	60 (53.2;67.4)	27.8 (35.1;20.3)	38.8 (34.1;44)	50.2 (43.9;57.3)	29.4 (39.6;20.8)	55.7 (49.5;63.2)	70.4 (62;79.3)	26.4 (35.8;16.2)
Paraíba	46.3 (40.4;52.6)	58.8 (51.8;66.9)	26.9 (33.9;21)	38.2 (33.8;43.5)	50.2 (43.8;57.5)	31.2 (41.8;21.7)	55.5 (47.5;63.8)	69.8 (60.8;79.5)	25.7 (36.4;16.8)
Paraná	58.8 (52.7;66.1)	67.9 (59.9;76.5)	15.5 (23.5;8.6)	48.4 (42.9;54.4)	57.7 (50.5;65.4)	19.4 (28.9;10.9)	69.6 (62.3;78.2)	80.1 (70.3;90.4)	15.1 (25.8;6.7)
Pernambuco	47 (41.9;53.1)	60.2 (53;68)	28.2 (35.5;20.8)	39.2 (34.7;44.1)	51.9 (45.4;58.5)	32.2 (42.2;22.9)	56.5 (50.1;63.9)	71.4 (62.5;80.8)	26.5 (36.8;16.7)
Piauí	45.8 (40.5;51.5)	57.8 (51;66)	26.2 (34.8;18.5)	38.1 (33.2;43.4)	49.1 (43;56.1)	28.9 (38.8;20.1)	54.5 (48.4;61.4)	68.2 (59.9;78)	25.1 (36.2;15.5)
Rio de Janeiro	53.1 (47.6;59.6)	64.6 (57.1;72.9)	21.6 (29;14.8)	44.3 (39.5;49.6)	55.5 (48.9;62.9)	25.4 (34.9;16.2)	65.2 (57.8;73.7)	77.1 (67.7;87.3)	18.2 (29;8.7)
Rio Grande do Norte	47.8 (42.7;53.9)	61.4 (54.2;69.4)	28.3 (35.9;21.6)	39.6 (35.1;45)	52.3 (45.9;59.4)	31.9 (42.5;23)	57 (50.5;64.3)	72.8 (64.1;82.2)	27.7 (36.8;18.8)
Rio Grande do Sul	60.4 (54;67.4)	68.6 (60.3;77.2)	13.5 (21.4;6.7)	50.2 (44.6;56.4)	58.5 (51.3;66.5)	16.6 (26.8;7.5)	73.6 (65.3;82.2)	81.6 (71.7;92.5)	10.8 (20.8;2.1)
Rondônia	51.4 (45.7;57.8)	62.6 (55.5;70.1)	21.9 (29.6;14.9)	40 (35.4;45.4)	52.2 (45.7;59)	30.4 (40.4;20.9)	60.4 (53.7;67.8)	72.8 (64.3;81.9)	20.4 (30.1;11.2)
Roraima	53.5 (47.5;59.9)	63.7 (56.3;71.7)	19.1 (26.6;12.4)	45.4 (40.2;51.2)	54.4 (47.7;61.5)	19.7 (29.3;10.8)	59.7 (52.8;67.2)	72.3 (63.9;81.6)	21.2 (30.9;12.1)
Santa Catarina	61.1 (54.5;68.6)	69 (60.8;77.5)	13.1 (20.6;6.4)	50.9 (44.9;57.7)	58.8 (51.3;66.5)	15.5 (26.3;6.6)	72.7 (64.4;82.2)	81.5 (71.7;92)	12.2 (21.6;4.5)
São Paulo	60.2 (53.8;67.5)	68.8 (60.9;77.4)	14.3 (22.1;7.9)	49.1 (43.6;55.2)	58.7 (51.8;66.3)	19.7 (28.7;11)	73.7 (65;83.2)	82 (72.5;92.5)	11.2 (20.4;3.3)
Sergipe	44.7 (39.3;50.9)	59.2 (52.1;67.4)	32.5 (40;25.8)	37.5 (32.7;43)	50.6 (44.6;58.2)	34.8 (45.1;26.2)	53.1 (46.5;60.8)	70.1 (61.2;80.1)	32 (42.5;23.5)
Tocantins	49.8 (44.3;56)	61.4 (54.7;69.2)	23.3 (30.7;16.4)	40.6 (35.6;46.1)	51.2 (45.4;57.8)	26.2 (36.3;18)	58.2 (51.7;65.8)	71.2 (62.5;80.6)	22.2 (31.6;13.3)

* Fonte: Estudo Global Burden of Disease 2017, Institute for Health Metrics and Evaluation. ^236^*

**Tabela 5-2:** – Morte e taxas de DALYs por 100 mil e variação percentual das taxas, por idade e causa de morte, no Brasil, 1990 e 2017

Causa de morte e grupo etário	1990	2017	Variação percentual (II 95%)	1990					2017	Variação percentual (II 95%)	
**Morte (padronizada por idade)**				**Taxa de DALYs (padronizada por idade)**							
***B.2.1- Doença cardíaca reumática***											
15-49 anos	1.4 (1.3;1.4)	0.6 (0.6;0.6)	-56.5 (-59.3;-52.9)	121.8 (104.9;143.4)					79.3 (62.4;101.4)	-34.9 (-41.7;-28.1)	
50-69 anos	4.8 (4.6;5)	2.6 (2.5;2.8)	-45.1 (-48.2;-41.2)	179.7 (164.5;198.4)					117.5 (102.8;136.9)	-34.6 (-39;-30.1)	
5-14 anos	0.7 (0.6;0.8)	0.2 (0.2;0.2)	-69.8 (-72.4;-65.6)	71.3 (61.1;80.6)					34.7 (28;43.5)	-51.4 (-58;-43.6)	
70+ anos	11.4 (11;11.7)	7.5 (7.2;7.8)	-33.9 (-37.1;-30.3)	174.2 (165.3;184.2)					113.8 (105.3;123.7)	-34.7 (-38.1;-31.1)	
Padronizada por idade	2.4 (2.3;2.5)	1.2 (1.1;1.2)	-50.3 (-52.4;-47.5)	118.9 (106.1;134.7)					71.2 (59;87.2)	-40.1 (-45.4;-34.6)	
Todas as idades	1.8 (1.7;1.8)	1.3 (1.2;1.3)	-28.5 (-31.7;-24.3)	109.3 (97;124.6)					76.5 (63.3;93.7)	-30 (-36.3;-23.9)	
Abaixo de 5 anos	0.6 (0.5;0.8)	0.1 (0.1;0.1)	-82.5 (-87.9;-74.4)	52.8 (40.6;68.7)					11 (9.3;13.1)	-79.1 (-85.3;-70.4)	
***B.2.5- Doença valvar do coração não reumática***											
15-49 anos	0.5 (0.4;0.5)	0.4 (0.4;0.5)	-23.5 (-30;1.2)		27 (21.7;28.8)		20 (18.5;24.3)				-26.1 (-32.2;-1.6)
50-69 anos	3.5 (2.9;3.7)	3.3 (2.6;3.4)	-6.9 (-14.5;-1.6)		104.1 (86.8;111.4)		94.9 (78.6;101.4)				-8.9 (-15.4;-3.8)
70+ anos	13.1 (11.7;15.1)	19 (14;20.2)	45.6 (10;57.1)		201 (179.1;230.6)		254.6 (194.7;280)				26.7 (1.3;35.5)
Padronizada por idade	1.7 (1.5;1.9)	1.8 (1.4;1.9)	7.1 (-9.1;13)		42.9 (36.6;45.8)		39.4 (33.2;42.2)				-8 (-15.6;-4)
Todas as idades	1 (0.9;1.1)	1.9 (1.5;2)	87.5 (63.5;96.9)		30.5 (25.9;32.5)		42.3 (35.8;45.3)				38.8 (27.5;47.2)
*** B.2.5.1- Doença valvar aórtica calcífica não reumática ***											
15-49 anos	0.3 (0.2;0.3)	0.2 (0.2;0.3)	-18.6 (-30.8;37.4)			14.3 (9.8;15.8)		11.3 (10.2;15.3)			-21.2 (-32.9;33.7)
50-69 anos	2.2 (1.6;2.4)	2.1 (1.9;2.5)	-4.1 (-14.1;25.2)			64.6 (47.3;70.3)		60.6 (54.6;71.2)			-6.2 (-16;23.2)
70+ anos	9.1 (7.5;9.9)	14.4 (11.1;15.6)	57.4 (35.7;73.3)			127.3 (102.9;138.2)		173.9 (136.8;188.7)			36.6 (22.9;52.4)
Padronizada por idade	1.1 (0.9;1.2)	1.3 (1.1;1.4)	16.3 (9.6;34)			25.4 (19.1;27.4)		25.1 (22.1;28.7)			-1.2 (-9.1;24.2)
Todas as idades	0.6 (0.5;0.7)	1.3 (1.1;1.5)	108.3 (94.8;146.2)			17.7 (13;19.2)		26.9 (23.9;31)			51.5 (38.3;94.9)
*** B.2.5.2- Doença valvar mitral degenerativa não reumática ***											
15-49 anos	0.2 (0.2;0.3)	0.2 (0.1;0.2)	-31.4 (-49.9;-22.8)		11.8 (9.8;14.5)		7.8 (5.6;8.7)				-33.9 (-51.3;-25.7)
50-69 anos	1.2 (1;1.6)	1 (0.6;1.2)	-12.7 (-47.4;3)		37.6 (32.6;49.1)		32.4 (19.6;36.8)				-14 (-44;-0.2)
70+ anos	3.7 (3.3;5.3)	4.3 (2.2;5.2)	17.7 (-42.1;39.2)		70.5 (57.7;92.6)		77.4 (50.2;94.7)				9.8 (-31.8;24.3)
Padronizada por idade	0.5 (0.5;0.7)	0.5 (0.3;0.5)	-10.8 (-47.6;2.4)		16.4 (14.4;20.8)		13.4 (8.8;15.2)				-18.7 (-43.4;-9.3)
Todas as idades	0.3 (0.3;0.5)	0.5 (0.3;0.6)	50.1 (-9.7;71.8)		12 (10.4;15.1)		14.4 (9.5;16.4)				20.3 (-15.9;35.4)

* Fonte: Estudo Global Burden of Disease 2017, Institute for Health Metrics and Evaluation. ^236^*

**Tabela 5-3:** – Número de mortes, taxa de mortalidade padronizada por idade (por 100 mil) e variação percentual das taxas, por grupo cardiovascular de causa de morte, no Brasil e suas unidades federativas, 1990 e 2017

**Causa de morte e localização **	**1990**			2017				**Variação percentual (II 95%) **	
	**Número (II 95%)**	**Taxa (II 95%)**		**Número (II 95%)**		**Taxa (II 95%)**			
***B.2.1- Doença cardíaca reumática***									
Acre	6.8 (6.3;7.3)	2.6 (2.5;2.8)		9.6 (8.8;10.4)		1.4 (1.3;1.5)		-46.2 (-51.8;-40.2)	
Alagoas	43.8 (37.3;51.6)	2.2 (1.9;2.5)		37.5 (34.9;40.2)		1.1 (1.1;1.2)		-47.3 (-54.4;-36.5)	
Amapá	2.7 (2.5;2.9)	1.8 (1.7;2)		6.6 (6;7.3)		1.1 (1;1.3)		-37.3 (-44.6;-29.1)	
Amazonas	18.3 (16.9;19.7)	1.5 (1.4;1.6)		26 (23.7;28.2)		0.8 (0.8;0.9)		-44.7 (-49.7;-39)	
Bahia	171.9 (155.9;186.2)	1.8 (1.6;1.9)		172 (159.2;185.3)		1.1 (1;1.2)		-40.3 (-46;-33.1)	
Brasil	2647.8 (2549.9;2727.7)	2.4 (2.3;2.5)		2682.3 (2584.5;2796.7)		1.2 (1.1;1.2)		-50.3 (-52.4;-47.5)	
Ceará	62.6 (51.4;72)	1.2 (1;1.3)		64.6 (60.2;69.2)		0.7 (0.6;0.7)		-44.3 (-51.3;-33.7)	
Distrito Federal	33.4 (31.2;35.8)	3.7 (3.5;4)		38.9 (35.8;42.7)		1.6 (1.5;1.8)		-56.8 (-61;-52.4)	
Espírito Santo	40.3 (38.1;42.6)	2.2 (2.1;2.4)		46.8 (43.4;50.8)		1.1 (1;1.2)		-50.6 (-54.7;-45.8)	
Goiás	74.6 (69.8;79.9)	2.8 (2.6;3)		100.8 (93.9;108.4)		1.5 (1.4;1.6)		-46.8 (-51.2;-42)	
Maranhão	100.4 (80.9;123.3)	2.3 (2;2.8)		59.8 (55.6;64.6)		0.9 (0.8;0.9)		-62.6 (-68.9;-55.4)	
Mato Grosso	23.9 (22.1;26)	1.9 (1.8;2.1)		32.3 (30;34.9)		1 (0.9;1.1)		-47.7 (-52.6;-42)	
Mato Grosso do Sul	25 (22;27.3)	2.1 (2;2.3)		33.3 (30.8;36.2)		1.2 (1.1;1.3)		-44.7 (-50;-38.8)	
Minas Gerais	331.2 (311.9;350.1)	2.8 (2.6;2.9)		343 (321;367.1)		1.4 (1.3;1.5)		-50.6 (-54.5;-46.2)	
Pará	52.4 (47;57.1)	1.7 (1.6;1.8)		70.3 (65.5;75.3)		1 (0.9;1)		-43.5 (-48.8;-37.2)	
Paraíba	61.5 (53.4;70.2)	2.2 (2;2.5)		49.1 (44.5;53.8)		1.1 (1;1.2)		-51.8 (-58.3;-43.7)	
Paraná	186.5 (177.8;196)	3.3 (3.1;3.4)		206.6 (193;221.9)		1.6 (1.5;1.8)		-49.5 (-53.4;-45.3)	
Pernambuco	113.9 (105.4;122.5)	2 (1.8;2.1)		132.3 (122.1;143.1)		1.3 (1.2;1.4)		-33.3 (-38.9;-26.3)	
Piauí	26.2 (19.9;31.3)	1.3 (1.1;1.5)		25.1 (23.3;27.1)		0.7 (0.6;0.7)		-47.5 (-55.3;-34.8)	
Rio de Janeiro	242.4 (230.8;253.8)	2.2 (2.1;2.3)		230.3 (215.4;247)		1.1 (1;1.2)		-49.2 (-53;-44.7)	
Rio Grande do Norte	31.9 (28.4;35.1)	1.6 (1.5;1.8)		44.1 (40.4;48.1)		1.2 (1.1;1.3)		-28.4 (-37.4;-16.5)	
Rio Grande do Sul	136.7 (129.5;143.6)	1.9 (1.8;2)		128.1 (118.5;137.7)		0.9 (0.8;1)		-52.3 (-56.1;-48.1)	
Rondônia	14.5 (13.3;15.8)	2.4 (2.2;2.6)		18.6 (16.5;21)		1.2 (1.1;1.4)		-49.5 (-56.3;-41.7)	
Roraima	1.8 (1.6;2)	1.8 (1.7;2)		3.5 (3;4)		0.9 (0.8;1.1)		-49.1 (-56.6;-39.5)	
Santa Catarina	72.3 (68.4;76)	2.4 (2.3;2.6)		86.2 (79.9;92.9)		1.2 (1.1;1.2)		-52.9 (-56.6;-48.6)	
São Paulo	735.7 (704.1;764.8)	3.1 (3;3.2)		671.8 (632;718.7)		1.3 (1.2;1.4)		-57.8 (-60.6;-54.7)	
Sergipe	24.3 (22.2;26.6)	2.1 (2;2.3)		28.2 (26.2;30.6)		1.3 (1.2;1.4)		-41.2 (-46.6;-34.5)	
Tocantins	12.6 (8.5;15.5)	2.3 (1.8;2.7)		16.9 (15.3;18.6)		1.1 (1;1.3)		-49.7 (-57.7;-37.2)	
***B.2.5- Doença valvar do coração não reumática***									
Acre	2.4 (2.2;2.8)	1.4 (1.3;1.7)		8.6 (7.3;9.5)		1.5 (1.3;1.7)		6.3 (-18.1;22)	
Alagoas	14.4 (12.8;19)	1.1 (0.9;1.4)		42.3 (37.9;50.9)		1.4 (1.2;1.7)		31.4 (8.4;50.9)	
Amapá	1.9 (1.3;2.1)	1.9 (1.4;2.1)		9.8 (6.4;10.9)		2.1 (1.4;2.4)		10.7 (-13.2;24.9)	
Amazonas	11.9 (10.2;13.6)	1.5 (1.3;1.7)		40.5 (33.5;44.9)		1.5 (1.3;1.7)		5.5 (-12.3;18.3)	
Bahia	96.1 (85.1;116.3)	1.4 (1.2;1.7)		197.5 (179.7;255.7)		1.2 (1.1;1.6)		-9 (-19.7;14.4)	
Brasil	1495.1 (1284.4;1620.4)	1.7 (1.5;1.9)		3974.4 (3116.8;4165.1)		1.8 (1.4;1.9)		7.1 (-9.1;13)	
Ceará	33.7 (26;60.7)	0.8 (0.6;1.5)		106.6 (96.7;136.7)		1.1 (1;1.4)		33.1 (-13.6;68.6)	
Distrito Federal	13.4 (10.3;14.5)	2.3 (1.8;2.5)		41.6 (36.3;47.7)		2.1 (1.9;2.5)		-6.6 (-18.4;16.3)	
Espírito Santo	30.9 (20.9;33.9)	2.4 (1.7;2.6)		86.1 (59.1;95.1)		2.1 (1.4;2.4)		-10.9 (-19.2;-2.6)	
Goiás	31.4 (26.2;34.1)	1.7 (1.4;1.9)		97.3 (83.6;106.9)		1.5 (1.3;1.7)		-9.9 (-17;-0.9)	
Maranhão	26.1 (19.5;46)	1 (0.7;1.8)		62.2 (52.7;100.7)		1 (0.8;1.6)		-1.9 (-16.9;19)	
Mato Grosso	12.9 (11.4;14.5)	1.6 (1.4;1.8)		46.2 (39.6;51.2)		1.6 (1.4;1.8)		3.3 (-10.8;15.6)	
Mato Grosso do Sul	16.5 (13;18)	1.9 (1.5;2.1)		50.6 (39.1;56)		1.9 (1.5;2.1)		-0.4 (-12.2;10.7)	
Minas Gerais	153.8 (135.2;169.9)	1.6 (1.5;1.8)		419.1 (332.3;454.1)		1.7 (1.3;1.8)		2.8 (-15.8;13.1)	
Pará	27.3 (24.8;34.4)	1.3 (1.1;1.6)		90.3 (81.5;107)		1.4 (1.3;1.7)		11.1 (-5.4;24.9)	
Paraíba	17.5 (13.9;31.9)	0.8 (0.6;1.4)		39.3 (32.8;68.9)		0.8 (0.7;1.5)		10.1 (-11.1;33.2)	
Paraná	105.3 (71.9;114.2)	2.4 (1.7;2.6)		293.1 (178.1;325.9)		2.5 (1.5;2.7)		4.1 (-14.8;14.3)	
Pernambuco	66.5 (59.1;75.4)	1.5 (1.4;1.8)		172.1 (138.3;188.5)		1.8 (1.4;2)		17.2 (-4;31)	
Piauí	13.1 (10.2;24.1)	0.9 (0.7;1.7)		34 (29.9;51.7)		0.9 (0.8;1.4)		1.1 (-22.9;23.9)	
Rio de Janeiro	150.6 (135.6;201.8)	1.6 (1.5;2.3)		357.5 (305.4;408)		1.7 (1.5;1.9)		3.7 (-15.2;14.2)	
Rio Grande do Norte	16.5 (13.9;24.9)	1 (0.8;1.5)		49 (44.2;62.1)		1.3 (1.2;1.6)		28.6 (-9.9;57.7)	
Rio Grande do Sul	140.6 (98.8;152.4)	2.3 (1.6;2.5)		389.2 (217.1;436.1)		2.7 (1.5;3)		15.2 (-12.5;27.8)	
Rondônia	5.9 (5.2;7.1)	1.6 (1.4;1.9)		19.5 (16.9;24.1)		1.4 (1.2;1.8)		-9.1 (-20.9;3.3)	
Roraima	0.9 (0.8;1.2)	1.7 (1.4;2.4)		4.3 (3.7;5.6)		1.5 (1.3;1.9)		-6.7 (-32.7;15.8)	
Santa Catarina	61 (37.5;67.1)	2.7 (1.6;3)		177.4 (104.2;198.4)		2.5 (1.5;2.9)		-4.4 (-15.1;4.4)	
São Paulo	431.6 (309.8;460.1)	2.2 (1.7;2.3)		1097 (770;1195.5)		2.2 (1.6;2.4)		1.9 (-14.5;10.9)	
Sergipe	7.7 (6.6;12.2)	0.9 (0.8;1.4)		23.3 (20.8;33.8)		1.1 (1;1.6)		24.3 (-0.4;44.1)	
Tocantins	5.2 (4.1;7.4)	1.5 (1.2;2.3)		19.9 (17.6;22.7)		1.5 (1.3;1.7)		-3.3 (-38.6;21.9)	
*** B.2.5.1- Doença valvar aórtica calcífica não reumática ***									
Acre	1.4 (1.1;1.6)	0.9 (0.7;1)		5.6 (4.8;6.8)		1 (0.9;1.2)		14.9 (-4.1;52.3)	
Alagoas	7.2 (6.1;9.1)	0.6 (0.5;0.7)		26.8 (23.5;34.2)		0.9 (0.8;1.1)		60.5 (35.4;93.6)	
Amapá	1.1 (0.6;1.3)	1.2 (0.7;1.4)		6.3 (4.5;7.4)		1.4 (1;1.7)		17.3 (-0.1;59.6)	
Amazonas	7.9 (4.9;8.9)	1 (0.7;1.1)		29.4 (24.2;33)		1.2 (0.9;1.3)		13 (-2.6;56.4)	
Bahia	62.9 (40.6;72.3)	0.9 (0.6;1)		134.5 (120.5;178)		0.8 (0.8;1.1)		-7.2 (-24.2;59)	
Brasil	950.4 (722.7;1016.7)	1.1 (0.9;1.2)		2806 (2349.2;3132)		1.3 (1.1;1.4)		16.3 (9.6;34)	
Ceará	17.3 (12.9;29.7)	0.4 (0.3;0.7)		68.6 (59.3;99.2)		0.7 (0.6;1)		62.7 (21;108.7)	
Distrito Federal	8.3 (7.1;9.9)	1.5 (1.3;1.8)		29.2 (24.8;40.5)		1.6 (1.3;2.2)		1.4 (-14.4;36.2)	
Espírito Santo	17.6 (11.7;19.9)	1.4 (1;1.6)		56.9 (43.8;64.3)		1.4 (1.1;1.6)		-1.9 (-14.3;21.5)	
Goiás	19.4 (13.8;21.9)	1.1 (0.8;1.3)		67.5 (60;77.2)		1.1 (1;1.2)		-3.8 (-16.4;24.2)	
Maranhão	13.2 (10.3;18.6)	0.5 (0.4;0.8)		41.1 (34.9;62.2)		0.7 (0.6;1)		25.3 (5.3;61.9)	
Mato Grosso	7.7 (6;8.8)	1 (0.8;1.1)		30.9 (27.5;37)		1.1 (1;1.3)		10.3 (-4.6;41.6)	
Mato Grosso do Sul	10.6 (7.1;12)	1.3 (0.9;1.5)		36 (28.9;40.3)		1.4 (1.1;1.5)		6.4 (-7.6;38.8)	
Minas Gerais	98 (73.1;109)	1.1 (0.8;1.2)		295 (242.1;330.1)		1.2 (1;1.3)		10.4 (0.3;30.5)	
Pará	15.1 (12.3;17.6)	0.7 (0.6;0.9)		55.4 (48;72.2)		0.9 (0.8;1.1)		19.9 (3.5;47.8)	
Paraíba	8.9 (6.7;17.1)	0.4 (0.3;0.8)		25.8 (20.1;49.3)		0.6 (0.4;1.1)		39.6 (10.9;72.5)	
Paraná	66.8 (42.3;75.1)	1.6 (1;1.8)		213.8 (136.2;240.2)		1.8 (1.2;2)		15.2 (3;34.3)	
Pernambuco	35.8 (29.4;41.1)	0.8 (0.7;1)		111.3 (95.1;129.6)		1.2 (1;1.3)		36.7 (19.2;56.2)	
Piauí	6 (4.6;10.6)	0.4 (0.3;0.8)		20 (16.4;33.7)		0.6 (0.5;0.9)		24 (-0.4;61.9)	
Rio de Janeiro	94.3 (81.2;132.4)	1.1 (0.9;1.5)		262.7 (234.5;331)		1.3 (1.1;1.6)		17.9 (1.2;32.7)	
Rio Grande do Norte	10.2 (8.3;13.1)	0.6 (0.5;0.8)		34.5 (30.4;44.4)		0.9 (0.8;1.2)		44 (17.3;86.2)	
Rio Grande do Sul	93.5 (61.7;103.8)	1.6 (1.1;1.8)		289 (173.5;325.4)		2 (1.2;2.2)		25.1 (4.5;43.9)	
Rondônia	3.5 (2.6;4.1)	1 (0.8;1.2)		13.1 (11;17.3)		1 (0.8;1.3)		-3 (-21.4;30.3)	
Roraima	0.6 (0.4;0.7)	1.2 (0.9;1.4)		3.3 (2.7;4.1)		1.2 (1;1.5)		3.7 (-16.7;46.1)	
Santa Catarina	40.2 (21.4;46.1)	1.8 (1;2.1)		132.6 (80.9;149.9)		1.9 (1.1;2.2)		4.8 (-6.6;29.4)	
São Paulo	296.7 (198;326.2)	1.5 (1.1;1.7)		790.7 (611;885)		1.6 (1.2;1.8)		5.1 (-4;27.3)	
Sergipe	3.8 (3.1;6.3)	0.5 (0.4;0.7)		14 (11.4;23.5)		0.7 (0.5;1.1)		48.6 (25.2;76.3)	
Tocantins	2.5 (2;3.5)	0.8 (0.6;1.2)		12 (10.3;15.9)		0.9 (0.8;1.2)		13.4 (-11.5;49.4)	
*** B.2.5.2- Doença valvar mitral degenerativa não reumática ***									
Acre	0.8 (0.7;1.4)		0.4 (0.4;0.8)		2.3 (1.7;2.8)		0.4 (0.3;0.5)		-13.6 (-53.6;12)
Alagoas	6.4 (5.2;9.7)		0.4 (0.4;0.7)		13.8 (10.5;16.7)		0.4 (0.3;0.5)		-1 (-35.6;24.3)
Amapá	0.6 (0.5;0.9)		0.6 (0.5;0.9)		2.8 (1.5;3.5)		0.6 (0.3;0.7)		-5.4 (-54.4;19)
Amazonas	3.6 (2.9;6.3)		0.4 (0.3;0.7)		9.6 (7.2;12)		0.3 (0.3;0.4)		-13.8 (-52.1;11.2)
Bahia	30.9 (24.8;55.3)		0.4 (0.3;0.8)		58.3 (44.5;75.6)		0.4 (0.3;0.5)		-12.5 (-45.7;11.9)
Brasil	510.6 (450.7;676.7)		0.5 (0.5;0.7)		1086.1 (621.9;1214.9)		0.5 (0.3;0.5)		-10.8 (-47.6;2.4)
Ceará	14.5 (6.7;28.8)		0.3 (0.2;0.7)		34.4 (22.8;39.9)		0.3 (0.2;0.4)		2.3 (-51.5;71.2)
Distrito Federal	4.8 (2.8;5.5)		0.7 (0.4;0.8)		11.4 (4.4;14.4)		0.5 (0.2;0.7)		-25.4 (-61.7;-7.7)
Espírito Santo	12.7 (7.9;16.4)		0.9 (0.6;1.2)		27.2 (12.3;32.9)		0.7 (0.3;0.8)		-25.7 (-53.2;-11.9)
Goiás	11.2 (9.8;15.1)		0.5 (0.5;0.8)		27.1 (17.8;31.5)		0.4 (0.3;0.5)		-23.2 (-50.5;-5.8)
Maranhão	10.8 (4.8;26.3)		0.4 (0.2;1)		17.1 (9.6;37.7)		0.3 (0.1;0.6)		-33.4 (-48.1;-10.8)
Mato Grosso	4.8 (4;6.5)		0.5 (0.4;0.7)		14.1 (8.2;16.5)		0.5 (0.3;0.5)		-10.3 (-50.3;12.3)
Mato Grosso do Sul	5.6 (4.7;7.5)		0.6 (0.5;0.8)		13.7 (7.5;16.9)		0.5 (0.3;0.6)		-14.8 (-51.4;5.8)
Minas Gerais	52.7 (46.2;74.3)		0.5 (0.5;0.8)		116.6 (70;135.7)		0.5 (0.3;0.5)		-12.1 (-50.7;7.4)
Pará	11.1 (8.9;16.6)		0.5 (0.4;0.7)		30.4 (20.2;35.2)		0.5 (0.3;0.5)		-3.9 (-40.7;18.8)
Paraíba	7.3 (3.7;14.5)		0.3 (0.2;0.6)		11.2 (7.1;19.1)		0.2 (0.2;0.4)		-22 (-45;5.2)
Paraná	36.5 (25.4;49.7)		0.7 (0.5;1)		74.2 (32.6;98.5)		0.6 (0.3;0.8)		-18.9 (-53.8;-3.3)
Pernambuco	29.2 (24.3;36)		0.6 (0.5;0.8)		57.2 (32.8;66.9)		0.6 (0.3;0.7)		-8.1 (-41.3;8.9)
Piauí	6 (2.9;12.6)		0.4 (0.2;0.9)		12 (8.1;17)		0.3 (0.2;0.5)		-18.7 (-50.7;20.3)
Rio de Janeiro	54 (41.3;69.8)		0.6 (0.4;0.7)		89.8 (52;104.2)		0.4 (0.2;0.5)		-23 (-52;-9)
Rio Grande do Norte	5.5 (3.1;11.5)		0.3 (0.2;0.7)		12.5 (9.9;16.1)		0.3 (0.3;0.4)		1.3 (-46.2;62.2)
Rio Grande do Sul	44.8 (30.8;61.3)		0.7 (0.5;0.9)		94.5 (39;135.6)		0.6 (0.3;0.9)		-6.7 (-49.3;10.9)
Rondônia	2.1 (1.7;3.4)		0.5 (0.4;0.8)		5.3 (3.9;6.7)		0.4 (0.3;0.5)		-25.1 (-52.5;-2.4)
Roraima	0.2 (0.2;0.6)		0.4 (0.2;1)		0.7 (0.5;1.5)		0.2 (0.2;0.5)		-41.8 (-65.4;-12.9)
Santa Catarina	19.9 (13.3;30.3)		0.8 (0.5;1.3)		42.4 (18.9;60.8)		0.6 (0.3;0.9)		-25.7 (-54.3;-10.7)
São Paulo	128.9 (98.6;180)		0.6 (0.5;0.9)		292.7 (118.9;351.9)		0.6 (0.2;0.7)		-5.7 (-55.2;16.5)
Sergipe	3.4 (2.1;5.8)		0.4 (0.2;0.7)		8 (5.3;9.6)		0.4 (0.2;0.4)		-2.4 (-41.6;23.1)
Tocantins	2.3 (1.4;3.7)		0.6 (0.3;1)		6.8 (4.2;8)		0.5 (0.3;0.6)		-19.2 (-63.8;33.1)

* Fonte: Estudo Global Burden of Disease 2017, Institute for Health Metrics and Evaluation. ^236^*

**Tabela 5-4:** – Número de DALYs, taxas de DALYs padronizadas por idade (por 100 mil) e variação percentual das taxas, por grupo cardiovascular de causa de morte, no Brasil e suas unidades federativas, 1990 e 2017

Causa de morte e localização	1990		2007		Variação percentual (II 95%)
	Número (II 95%)	Taxa (II 95%)	Número (II 95%)	Taxa (II 95%)	
***B.2.1- Doença cardíaca reumática***					
Acre	460.8 (410.3;524.6)	127.1 (114.1;144.3)	671 (557.8;816.4)	79.5 (67.1;95.5)	-37.5 (-43.8;-30.7)
Alagoas	3143.3 (2600.7;3851.6)	125 (105.1;149.3)	2632.8 (2168.9;3233.3)	73.8 (61.1;90.1)	-40.9 (-50;-30.9)
Amapá	204.8 (173.1;242.1)	91.4 (78.4;106.6)	551.5 (440.6;687.6)	71.1 (57.7;87.5)	-22.2 (-29.7;-14.4)
Amazonas	1528 (1267.3;1848.2)	85.2 (71.4;102.2)	2460.5 (1938.2;3170.4)	62.1 (49.5;78.9)	-27.1 (-33.3;-20.8)
Bahia	12095.4 (10485.4;13973.7)	103.7 (90.3;120.3)	11460 (9428.5;14129.4)	70.5 (58.1;86.8)	-32.1 (-38.7;-24.8)
Brasil	163388 (144922.3;186177.2)	118.9 (106.1;134.7)	162013.6 (134153.5;198443.9)	71.2 (59;87.2)	-40.1 (-45.4;-34.6)
Ceará	5090.4 (4101.6;6158.3)	81.2 (65.9;98.4)	5664.1 (4357.1;7338.2)	56.4 (43.5;72.8)	-30.6 (-39.8;-20.8)
Distrito Federal	2073.5 (1853.8;2341.2)	151.6 (137.6;168.9)	2439.1 (2023.3;2978.4)	78.8 (65.8;94.9)	-48 (-53.7;-42)
Espírito Santo	2628.8 (2295.3;3051.6)	109.2 (96.1;125.8)	2974.6 (2426.3;3661.6)	70.1 (57.1;86.3)	-35.8 (-42;-29.9)
Goiás	4735.6 (4205.1;5429.8)	128 (114.7;145.2)	5840.8 (4903.6;7144.8)	79.8 (67.3;97.3)	-37.7 (-43.5;-31.5)
Maranhão	7473 (5921.2;9327.9)	142.1 (116.4;171.6)	4842.9 (3887.8;6116.6)	63.2 (51;79.3)	-55.6 (-64.4;-46.3)
Mato Grosso	1828.6 (1579.2;2132.2)	101.6 (88.5;117.3)	2466 (1996.8;3072)	66.9 (54.5;82.8)	-34.2 (-40.9;-27.8)
Mato Grosso do Sul	1669 (1402.1;1974.2)	104.5 (90.1;121.8)	2109.8 (1733.1;2612.1)	71.2 (58.6;87.9)	-31.8 (-38.6;-24.4)
Minas Gerais	19249.1 (17181.8;21938.6)	130.8 (117.6;148)	18154.8 (15183.7;21952.5)	76.5 (63.5;92.8)	-41.5 (-47.4;-35.5)
Pará	3950.3 (3349.3;4663.4)	91.5 (78.6;107.5)	5808.4 (4645;7300.4)	65.9 (53.3;82)	-27.9 (-34.8;-20.8)
Paraíba	3916.4 (3288.8;4699.1)	125.1 (106.1;147.5)	3123.4 (2541.7;3837.2)	70.6 (57.5;86.9)	-43.5 (-52;-34.8)
Paraná	10399.6 (9331.5;11680)	136.1 (123.1;152.3)	10256.3 (8682.8;12479.2)	81.7 (68.6;99.4)	-40 (-45.5;-33.8)
Pernambuco	7748.4 (6752.1;8887.8)	110.2 (95.9;126.4)	8150.9 (6836.5;9867.3)	78.7 (66.2;95.3)	-28.5 (-34.6;-22.1)
Piauí	2106.2 (1603.1;2601.3)	83.8 (66;103.3)	2139 (1699.7;2745.7)	58.6 (46.7;75.1)	-30.1 (-40.3;-17.3)
Rio de Janeiro	14806.5 (13066.6;16943.4)	113.9 (100.9;129.8)	13785.8 (11373.1;17049.6)	70.7 (58;88)	-37.9 (-43.9;-31.8)
Rio Grande do Norte	2163 (1817.4;2559.8)	94.3 (79.8;111.1)	2852.8 (2365.9;3507.2)	75.4 (62.7;92.4)	-20.1 (-28.4;-10.9)
Rio Grande do Sul	8371.7 (7163.3;9922.7)	94.4 (81.5;110.9)	7857.9 (6265.8;9956.8)	62 (48.9;78.6)	-34.4 (-40.8;-27.9)
Rondônia	1066 (924.3;1232.7)	113.4 (100;129.4)	1317 (1055;1625.9)	73 (58.9;89.6)	-35.7 (-43.4;-28.3)
Roraima	153.9 (127.6;187.4)	88.8 (75.3;105.5)	335.3 (262.1;430.8)	61.7 (48.9;77.9)	-30.5 (-38.5;-22.8)
Santa Catarina	4413.5 (3816.7;5148.1)	108.6 (95.7;125)	5283.7 (4296.7;6556.9)	68.4 (55.6;85)	-37 (-43.4;-30.6)
São Paulo	39592.8 (35476.3;45103.4)	134.5 (121.5;151.7)	35783 (29557.6;44123.8)	70.9 (58.2;87.7)	-47.3 (-53.3;-41.3)
Sergipe	1627.5 (1401.2;1913.6)	114.8 (100.3;133)	1870.6 (1561.8;2277.1)	77 (64.6;93.1)	-33 (-40.2;-26.3)
Tocantins	891.8 (595.4;1118.1)	109.4 (80.7;133.7)	1181.6 (973.8;1459.3)	72.9 (60.4;89.7)	-33.3 (-44.6;-15.9)
***B.2.5- Doença valvar do coração não reumática***					
Acre	76.6 (68.6;89.8)	34.6 (31.1;41)	231.2 (202.9;258.1)	34.9 (30.4;39)	1.1 (-16.5;14.5)
Alagoas	435.5 (385.3;565.4)	27.1 (24;35.1)	1122.2 (1002.4;1359.9)	34.4 (30.7;41.5)	26.9 (7.9;43.8)
Amapá	60 (41.1;66.2)	43.9 (31;48.5)	276 (187.7;307.7)	48.3 (32.6;53.9)	10 (-7.8;24.5)
Amazonas	383.1 (320.1;430.5)	35.8 (30.9;40.2)	1120.8 (942.5;1248.8)	36.4 (30.5;40.4)	1.7 (-10.3;14)
Bahia	2839.9 (2502.2;3279.7)	35.1 (31.1;40.7)	4949.5 (4486.8;6140.9)	30.8 (28;38.3)	-12.2 (-22.2;12)
Brasil	45589.2 (38652.3;48617.4)	42.9 (36.6;45.8)	89684.3 (75761.8;95891.8)	39.4 (33.2;42.2)	-8 (-15.6;-4)
Ceará	979.5 (757.8;1644.8)	21.4 (16.6;36)	2601.6 (2333.2;3343)	26.2 (23.5;33.6)	22.2 (-13.9;51.7)
Distrito Federal	475.3 (369.5;517.4)	53 (41.8;57.3)	1026.2 (904.4;1164.8)	40.2 (35.3;45.4)	-24.1 (-32.1;-7.6)
Espírito Santo	954.5 (637.9;1049.7)	53.2 (36.8;58.2)	1994.2 (1441.6;2200)	46.7 (33.9;51.6)	-12.2 (-19.7;-3.4)
Goiás	1044.4 (870.5;1136.7)	40.3 (34;43.8)	2513.9 (2156.8;2780.7)	36.3 (31.4;40.2)	-10 (-17;-1)
Maranhão	841.7 (638.4;1353.6)	27.4 (21;44.5)	1646 (1410.8;2450.2)	24.5 (21;36.5)	-10.4 (-24;7.8)
Mato Grosso	448.3 (391.9;502.9)	39.1 (34.6;43.8)	1235 (1068.3;1378.4)	37.4 (32.3;41.7)	-4.3 (-15.6;7.1)
Mato Grosso do Sul	518 (401.4;571.5)	45.4 (35.9;49.7)	1237.3 (960.8;1368.1)	43.2 (33.7;47.7)	-4.9 (-15.2;6.1)
Minas Gerais	4805.1 (4079.6;5198.5)	41.1 (35.6;44.6)	9545.6 (7991.5;10392.3)	38 (31.9;41.5)	-7.7 (-17.5;-0.1)
Pará	871.1 (775.6;1053.9)	31.3 (28.2;38.5)	2471.2 (2222.8;2874.6)	33.9 (30.5;39.6)	8.3 (-6.6;20.6)
Paraíba	485 (382.9;858.3)	19.9 (15.8;34.8)	978.9 (818.3;1667.4)	21.5 (17.9;36.6)	8.1 (-11.5;28.3)
Paraná	3211.1 (2218.8;3517.2)	54.6 (38.2;59.6)	6327.7 (4281.6;6982.9)	49.8 (33.6;55)	-8.8 (-18.1;-1.4)
Pernambuco	2014.5 (1698.1;2212.9)	38.6 (33.3;42.7)	4112.2 (3493.4;4518.3)	41 (34.7;44.9)	6.1 (-4.3;16.3)
Piauí	381.9 (291.7;687.1)	23.2 (18.1;41)	879.2 (770.3;1289.3)	24.2 (21.2;35.5)	4.3 (-18.9;27.5)
Rio de Janeiro	4639.3 (4053.3;5965.9)	41.8 (37.1;54.1)	7855.6 (7028.2;9622.4)	37.1 (33.3;45.6)	-11.2 (-18.7;-3.6)
Rio Grande do Norte	446.7 (374.6;658.4)	25.3 (21.2;37)	1205.9 (1082.5;1495.1)	31.9 (28.6;39.6)	26.3 (-7.3;51.8)
Rio Grande do Sul	4017.2 (2844.8;4352.4)	53.9 (38.7;58.5)	7588.9 (4941.1;8430.1)	51.9 (34.2;57.5)	-3.7 (-19.7;5.2)
Rondônia	221.1 (193.3;264.1)	39.1 (34.9;46.8)	524.4 (452.9;662.3)	33.5 (29;41.7)	-14.5 (-25.9;-1.6)
Roraima	32 (27.8;41.7)	35.3 (30.8;48.9)	120.3 (104;161.8)	31.5 (27.3;41)	-10.9 (-28.3;7.3)
Santa Catarina	1766.7 (1153.7;1937.8)	58.3 (37.4;63.9)	3758.7 (2515;4171.6)	49.2 (32.4;54.7)	-15.6 (-22.8;-7.7)
São Paulo	13252 (9281.2;14232.8)	54.6 (39;58.4)	23226.5 (18142.4;25116.5)	44.9 (35.1;48.6)	-17.7 (-24.1;-10.8)
Sergipe	218.2 (184.3;330.5)	22.6 (19.1;34.2)	615.9 (541.8;879.7)	27.4 (24.2;39.1)	21.2 (1.3;39)
Tocantins	170.7 (124.4;233.6)	33.3 (26.2;46.2)	519.5 (455.3;586.8)	35.4 (31.1;40)	6.4 (-24;35.7)
*** B.2.5.1- Doença valvar aórtica calcífica não reumática ***					
Acre	40.5 (30;46.2)	19.1 (14.6;21.7)	133.9 (112.7;168.1)	20.8 (17.7;25.7)	9 (-10.8;51.7)
Alagoas	189.8 (160.5;241.4)	12.2 (10.4;15.3)	625.7 (549.3;827.4)	19.4 (17.1;25.3)	58.6 (33.4;93.9)
Amapá	32.1 (18.2;37)	24.9 (14.5;28.3)	161.6 (122.6;192)	29.4 (21.7;34.5)	17.8 (-0.4;68.2)
Amazonas	231.6 (136.2;266.9)	22.4 (13.8;25.4)	739.3 (617.9;840)	24.4 (20.1;27.5)	9.1 (-6.7;58.5)
Bahia	1658.3 (1011.9;1940.1)	20.9 (13.1;24.3)	3000.9 (2707.6;3816.1)	18.8 (16.9;23.9)	-10.4 (-27;61.2)
Brasil	26502.4 (19395.6;28697.2)	25.4 (19.1;27.4)	56930.9 (50545.9;65681.9)	25.1 (22.1;28.7)	-1.2 (-9.1;24.2)
Ceará	429.8 (315.7;695.5)	9.6 (7.1;15.5)	1451.9 (1234;2158.8)	14.7 (12.5;21.7)	52.5 (18.4;95)
Distrito Federal	271.8 (234.1;332.2)	32.1 (27.4;38.2)	655.9 (552.3;920.4)	26.3 (22.3;36.6)	-18.1 (-30.1;8.5)
Espírito Santo	488.7 (313.1;557)	28.5 (18.8;32.2)	1182.1 (959.5;1371.1)	27.8 (22.4;32)	-2.4 (-15.2;28.3)
Goiás	589.3 (415.1;674)	23.7 (17;26.7)	1582.9 (1418.5;1838.7)	23 (20.6;26.6)	-3 (-15.7;29.8)
Maranhão	377.9 (295.1;491.2)	12.6 (10;16.7)	951.9 (812.4;1361.8)	14.3 (12.2;20.4)	13.4 (-5.9;44.1)
Mato Grosso	243.7 (182.5;280.4)	22.4 (17.3;25.6)	749.8 (666.3;912.9)	23 (20.5;27.7)	2.6 (-12.2;37)
Mato Grosso do Sul	302.3 (199.2;348.4)	27.5 (18.3;31.2)	806 (658.2;903.3)	28.3 (23;31.6)	2.8 (-12;39)
Minas Gerais	2808.8 (1974.5;3167.3)	24.5 (17.8;27.4)	6005.2 (5096.3;6815.2)	23.9 (20.4;27.2)	-2.5 (-13.6;22.8)
Pará	423.2 (333.3;485.2)	16 (13;18.4)	1351.8 (1171.1;1746.7)	19 (16.5;24.4)	18.6 (1.6;53.1)
Paraíba	206.3 (155.1;396.3)	8.6 (6.5;16.2)	547.1 (426.1;1049)	12 (9.3;23.1)	40.1 (11.5;73.1)
Paraná	1874.7 (1154.1;2123.9)	32.9 (20.9;36.9)	4232.1 (3003.5;4719)	33.4 (23.7;37.2)	1.4 (-9;23.8)
Pernambuco	965.9 (746.3;1081.1)	19 (15.1;21.3)	2374.9 (2111.6;2875.8)	23.8 (21.2;28.7)	25.5 (9.4;53.7)
Piauí	152.4 (113.5;255.6)	9.6 (7.3;16.1)	449.8 (369.5;734.1)	12.4 (10.2;20.3)	29.1 (2.6;69.1)
Rio de Janeiro	2651.5 (2230.2;3550.9)	24.2 (20.8;33)	5140.9 (4615.8;7109.1)	24.2 (21.8;33.6)	0 (-10.3;18.3)
Rio Grande do Norte	241.5 (194.2;300.7)	13.8 (11.2;17.3)	750.5 (657;955.2)	19.9 (17.4;25.4)	43.9 (15.2;93.5)
Rio Grande do Sul	2457.2 (1619.1;2728)	33.6 (22.2;37.2)	5106.1 (3487.4;5690.3)	34.8 (23.9;38.6)	3.8 (-6.2;22.4)
Rondônia	121.9 (90.7;143.3)	22.8 (17.7;26.2)	317.1 (262.3;435)	20.6 (17.2;27.3)	-9.8 (-28;26.9)
Roraima	20.4 (14.5;23.9)	23.2 (17.5;26.6)	82.2 (68.4;107.1)	22.1 (18.7;27.4)	-4.7 (-23.2;36.4)
Santa Catarina	1071.1 (587;1233.8)	36.6 (19.9;41.7)	2571.3 (1727.2;2869.9)	33.9 (22.4;37.9)	-7.3 (-17.4;18.6)
São Paulo	8484.5 (5317.1;9384.8)	35.5 (23.3;39.1)	15357.3 (13142.4;17600.3)	29.7 (25.2;33.9)	-16.2 (-26;10.5)
Sergipe	93.2 (75.2;149.4)	9.9 (8;15.8)	324.7 (258.7;557.4)	14.6 (11.7;24.7)	47.2 (20.2;81.9)
Tocantins	74 (53.8;103.7)	15.3 (11.9;21)	278.2 (234.9;375.9)	19.2 (16.3;25.7)	25.8 (-2.9;66.6)
*** B.2.5.2- Doença valvar mitral degenerativa não reumática ***					
Acre	30 (25.2;46)	13.1 (10.9;20.6)	75.1 (60.5;90.7)	11.2 (8.9;13.4)	-14.8 (-45.7;3.5)
Alagoas	220.5 (183.7;311.8)	13.5 (11.2;19)	444.4 (356.2;522.7)	13.4 (10.8;15.8)	-0.1 (-29.4;20.1)
Amapá	23.8 (19.6;30.9)	16.6 (14;21.9)	91.8 (53.3;112.4)	15.6 (9.2;19)	-6 (-44.1;13.9)
Amazonas	136 (113;215.8)	12.2 (10.1;19.4)	331.9 (263.6;401.4)	10.6 (8.4;12.7)	-13.3 (-43;4.9)
Bahia	1104.1 (905.1;1731.5)	13.3 (10.9;21.1)	1828.7 (1488.1;2227.1)	11.3 (9.2;13.8)	-14.9 (-42.1;2.9)
Brasil	17936 (15500.4;22621.4)	16.4 (14.4;20.8)	30580.8 (20064.3;34729.6)	13.4 (8.8;15.2)	-18.7 (-43.4;-9.3)
Ceará	493.4 (278.6;898.4)	10.7 (6.1;19.1)	1052.9 (767.3;1230.3)	10.6 (7.7;12.4)	-1.2 (-43.9;35.8)
Distrito Federal	190.7 (115.1;218.4)	19.7 (12.4;22.5)	339.3 (159.2;421.1)	12.8 (6.2;15.9)	-35.1 (-59.2;-22.7)
Espírito Santo	443.7 (279.5;557.8)	23.6 (15.4;29.7)	762.6 (387;901.9)	17.7 (9.1;21)	-24.9 (-45.7;-13.2)
Goiás	424.4 (369.9;542.4)	15.6 (13.6;20)	855.1 (613.7;989.7)	12.2 (8.9;14.2)	-21.6 (-43.6;-8.5)
Maranhão	385.9 (203.6;833)	12.5 (6.8;26.7)	582.5 (380.8;1068.8)	8.6 (5.7;15.7)	-31 (-45.2;-11)
Mato Grosso	190.4 (160.8;242.1)	15.7 (13.3;20.6)	447.5 (286.6;524.3)	13.3 (8.7;15.7)	-14.8 (-44.4;3.3)
Mato Grosso do Sul	204.1 (169.1;260.1)	17 (14.4;21.8)	406.4 (251.7;480.2)	14.1 (8.8;16.6)	-17.4 (-46;-0.6)
Minas Gerais	1890.6 (1645.4;2507.9)	15.8 (13.9;21.1)	3353.1 (2245.4;3860.9)	13.3 (9;15.3)	-15.7 (-44.4;-0.2)
Pará	404.9 (333.3;563.2)	14 (11.7;19.8)	983 (706.3;1136.6)	13.3 (9.6;15.4)	-5.5 (-35.3;12.4)
Paraíba	237.4 (142.8;443.7)	9.7 (5.9;17.8)	370 (265.7;569.3)	8.1 (5.8;12.5)	-16.5 (-38.4;3.1)
Paraná	1268 (887.1;1627.5)	20.6 (14.9;26.9)	1968.9 (1084.3;2414.3)	15.4 (8.5;18.9)	-25.5 (-48;-13.3)
Pernambuco	999.8 (786.7;1162.9)	18.8 (15.1;22)	1641.1 (1077;1887.1)	16.2 (10.7;18.7)	-13.7 (-34;-1.5)
Piauí	195.7 (108.4;388.3)	11.8 (6.8;22.7)	376.3 (274.4;508.7)	10.3 (7.5;14)	-12.3 (-42.4;17.5)
Rio de Janeiro	1909.1 (1454.4;2345.8)	16.9 (13;20.8)	2593.4 (1731.6;3056.8)	12.3 (8.2;14.5)	-27.2 (-47.3;-16.1)
Rio Grande do Norte	183.1 (118.7;339.9)	10.3 (6.7;18.9)	401.6 (333.7;489.9)	10.6 (8.8;12.9)	3 (-38.3;45.6)
Rio Grande do Sul	1483.2 (1058.6;1927.1)	19.4 (13.9;25.2)	2354.6 (1214.6;2951.9)	16.1 (8.4;20.2)	-16.7 (-46.7;-2.1)
Rondônia	87 (71.6;132.9)	14.6 (12.1;22.1)	172.4 (133;219)	10.9 (8.5;13.5)	-25.6 (-47.3;-7.7)
Roraima	9.1 (6.4;21.2)	10 (7.1;23)	27 (19.8;52.4)	7 (5.2;12.6)	-30.3 (-53.4;-10)
Santa Catarina	664.1 (474.2;931.5)	20.8 (14.7;29.9)	1126.1 (625.8;1436.1)	14.5 (8.2;18.8)	-30.2 (-50.8;-18.5)
São Paulo	4562.6 (3474.3;6176.4)	18.3 (14.3;24.5)	7528.5 (4049.6;8857.7)	14.5 (7.8;17)	-20.8 (-50.7;-5.8)
Sergipe	111.2 (75.5;172)	11.4 (7.8;17.5)	256 (187.3;305.8)	11.3 (8.3;13.7)	-0.9 (-32.9;20.8)
Tocantins	83.2 (52.6;126.5)	15.7 (10.1;24)	210.5 (142.8;246.4)	14.2 (9.7;16.6)	-9.5 (-49.6;31)

* Fonte: Estudo Global Burden of Disease 2017, Institute for Health Metrics and Evaluation. ^236^*

**Figura 5-1:**
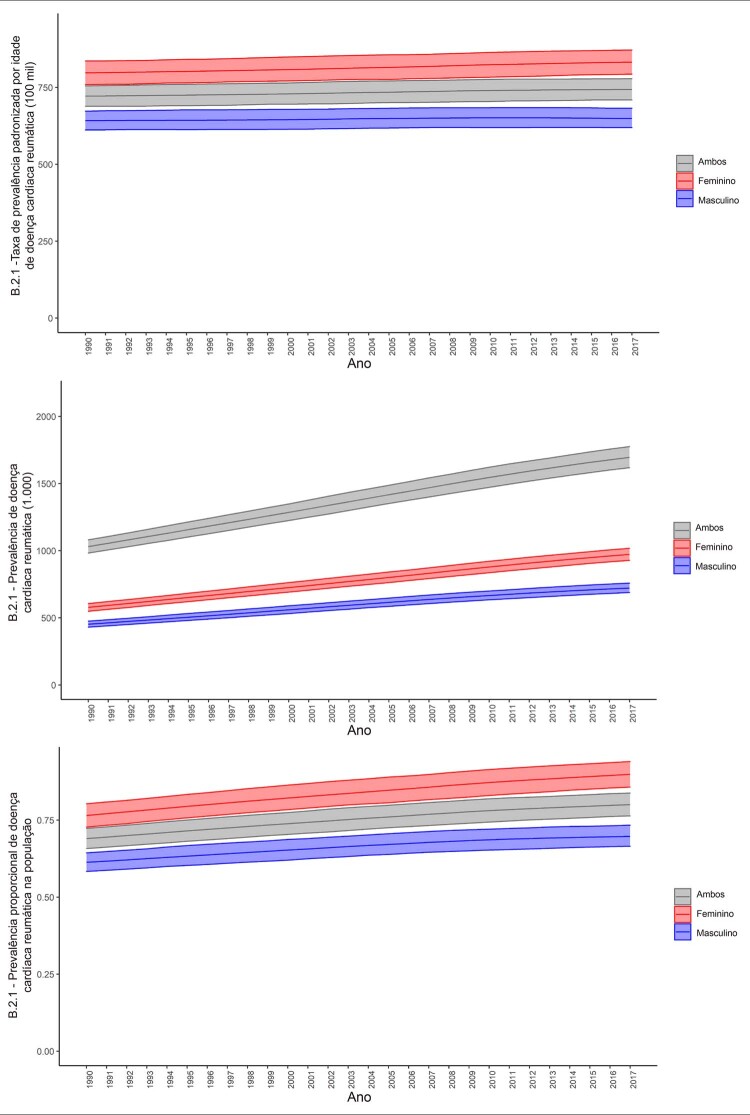
- A: Taxas de prevalência padronizadas por idade de doença cardíaca reumática no Brasil em 1990-2017. B: Taxa bruta de prevalências de doença cardíaca reumática no Brasil em 1990-2017. C: Prevalência proporcional de doença cardíaca reumática na população brasileira em 1990-2017.

Dados derivados do Estudo Global Burden of Disease 2017 (GBD 2017). ^236^

**Figura 5-2:**
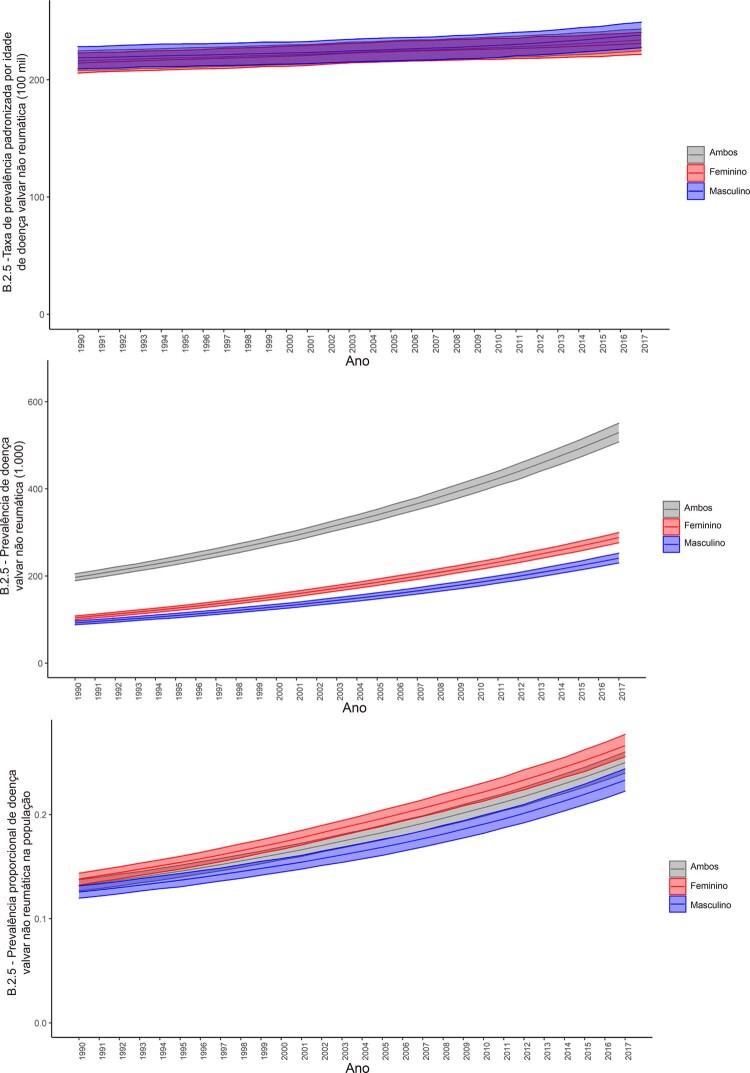
- A: Taxas de prevalência padronizadas por idade de doença valvar do coração não reumática no Brasil em 1990-2017. B: Taxa bruta de prevalência de doença valvar do coração não reumática no Brasil em 1990-2017. C: Prevalência proporcional de doença valvar do coração não reumática na população brasileira em 1990-2017.

Dados derivados do Estudo Global Burden of Disease 2017 (GBD 2017). ^236^

**Figura 5-3:**
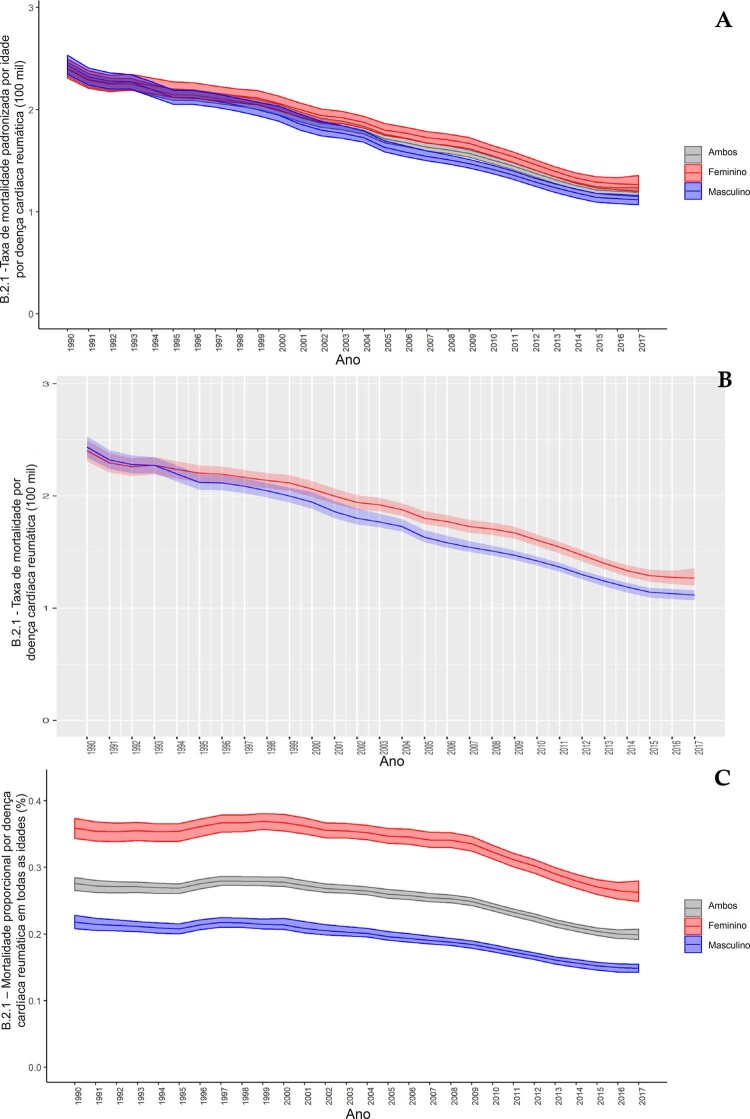
- A: Taxas de mortalidade padronizadas por idade atribuíveis a doença cardíaca reumática no Brasil em 1990–2017. B: Taxa bruta de mortalidade atribuível a doença cardíaca reumática no Brasil em 1990–2017. C: Mortalidade proporcional atribuível a doença cardíaca reumática na população brasileira em 1990–2017.

Dados derivados do Estudo Global Burden of Disease 2017 (GBD 2017). ^236^

**Figura 5-4:**
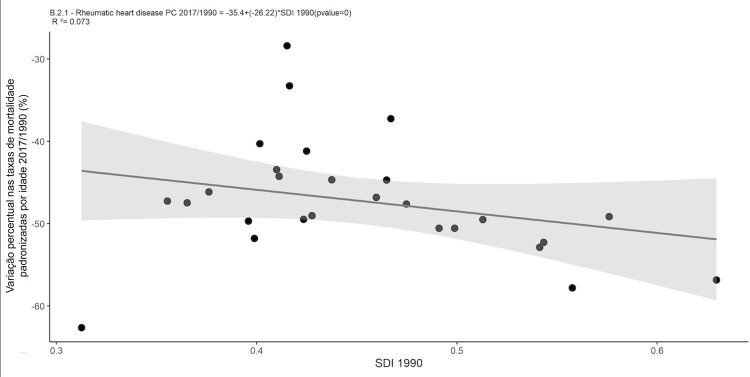
- Correlação entre o Índice Sociodemográfico (SDI) e a variação percentual nas taxas de mortalidade padronizadas por idade atribuíveis a doença cardíaca reumática nas unidades federativas brasileiras em 1990.

Dados derivados do Estudo Global Burden of Disease 2017 (GBD 2017). ^236^

**Figura 5-5:**
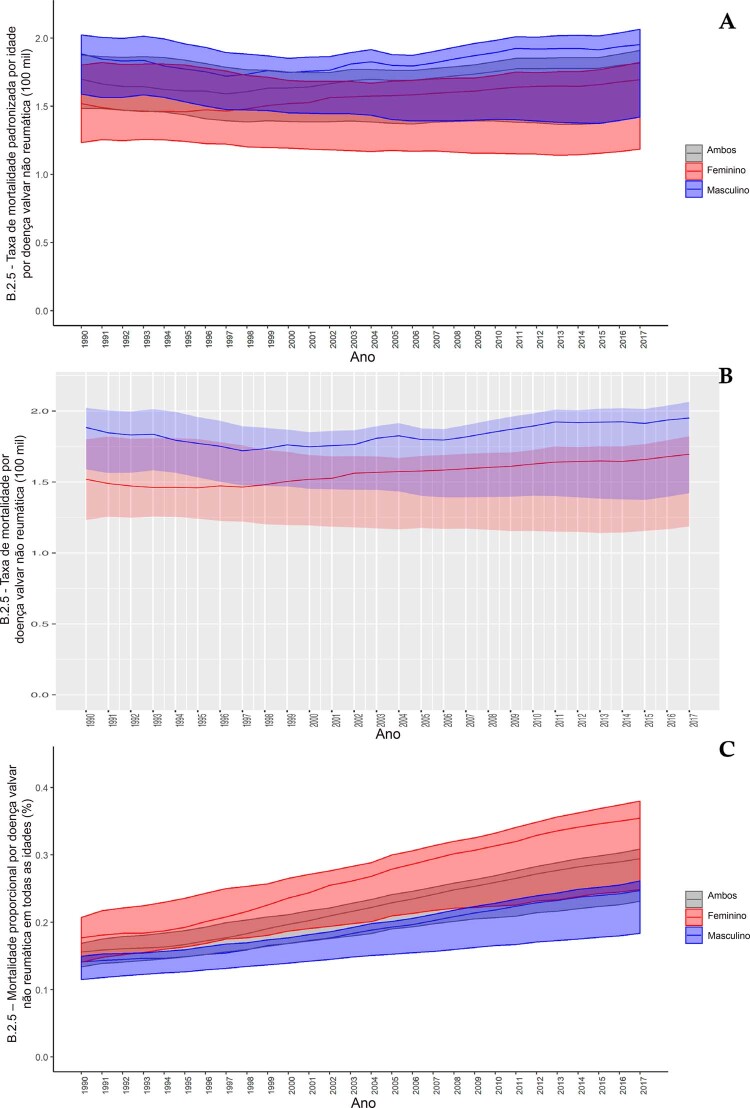
- A: Taxas de mortalidade padronizadas por idade atribuíveis a doença valvar do coração não reumática no Brasil em 1990–2017. B: Taxas brutas de mortalidade atribuíveis a doença valvar do coração não reumática no Brasil em 1990–2017. C: Mortalidade proporcional atribuível a doença valvar do coração não reumática na população brasileira em 1990–2017.

Dados derivados do Estudo Global Burden of Disease 2017 (GBD 2017). ^236^

**Figura 5-6:**
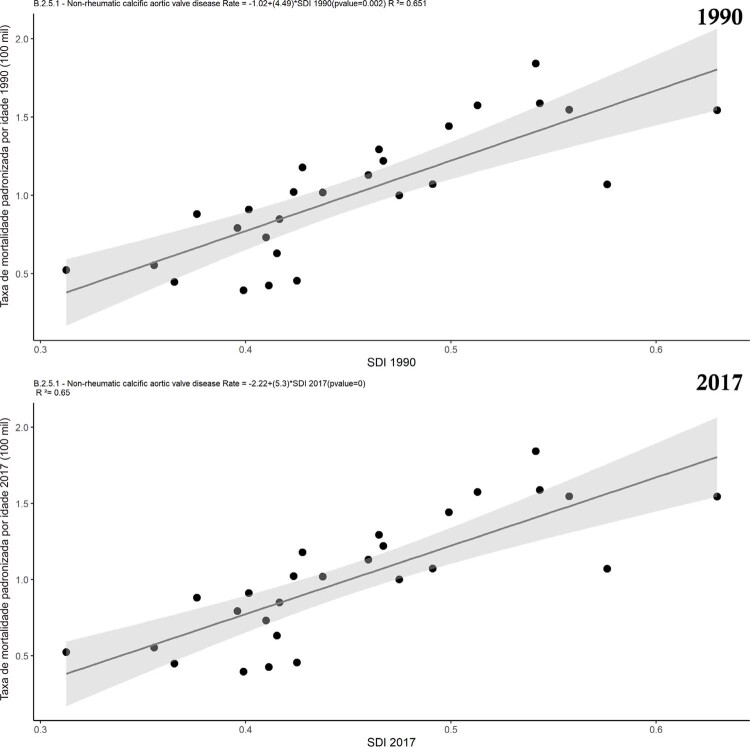
- Correlação entre as taxas de mortalidade padronizadas por idade atribuíveis a doença valvar aórtica calcífica e o Índice Sociodemográfico (SDI) nas unidades federativas brasileiras em 1990 e 2017.

Dados derivados do Estudo Global Burden of Disease 2017 (GBD 2017). ^236^

**Figura 5-7:**
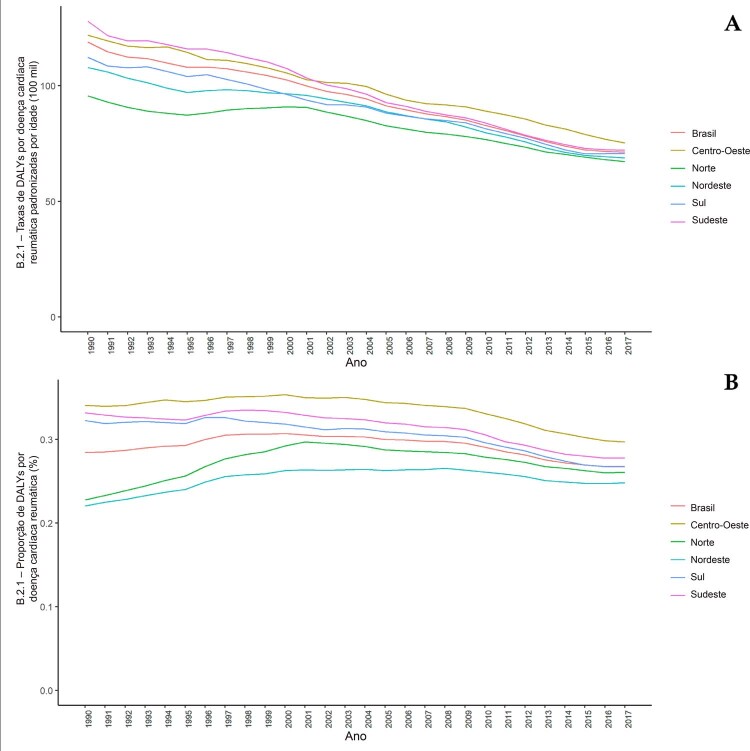
- A: Taxas de DALYs padronizadas por idade atribuíveis a doença cardíaca reumática no Brasil e cada região em 1990–2017. B: Proporção de DALYs atribuíveis a doença cardíaca reumática no Brasil e cada região em 1990–2017.

Dados derivados do Estudo Global Burden of Disease 2017 (GBD 2017). ^236^

**Figura 5-8:**
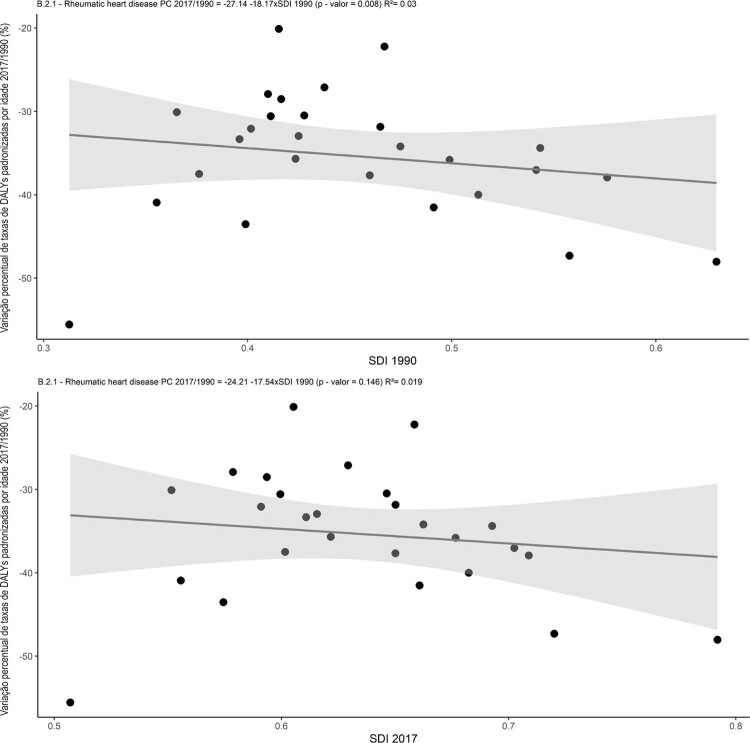
- Correlação entre a variação percentual nas taxas de DALYs atribuíveis a doença cardíaca reumática em 1990 – 2017 e o Índice Sociodemográfico (SDI) nas unidades federativas brasileiras em 1990 e 2017.

Dados derivados do Estudo Global Burden of Disease 2017 (GBD 2017). ^236^

**Figura 5-9:**
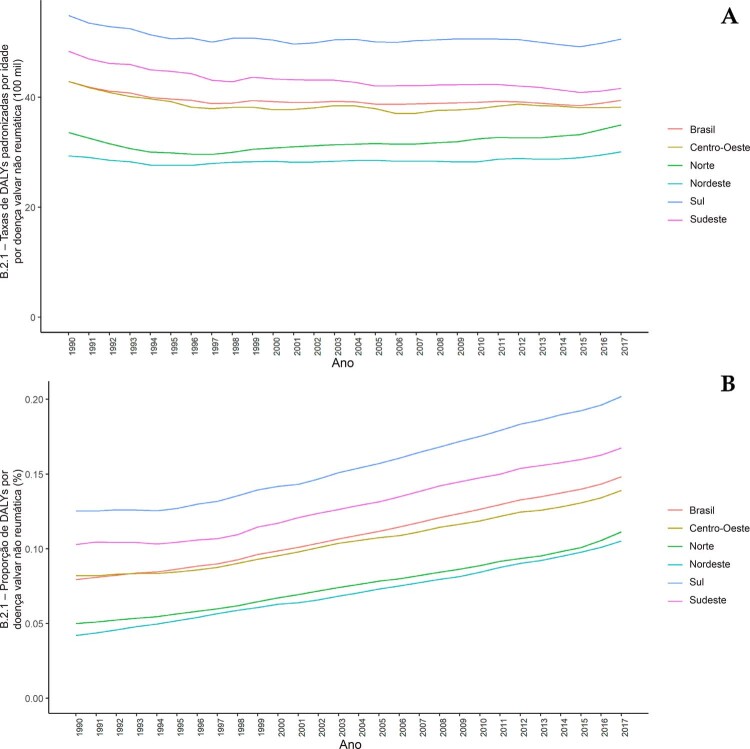
- A: Taxas de DALYs padronizadas por idade atribuíveis a doença valvar do coração não reumática no Brasil e cada região em 1990–2017. B: Proporção de DALYs atribuíveis a doença valvar do coração não reumática no Brasil e cada região em 1990–2017.

Dados derivados do Estudo Global Burden of Disease 2017 (GBD 2017). ^236^

**Figura 5-10:**
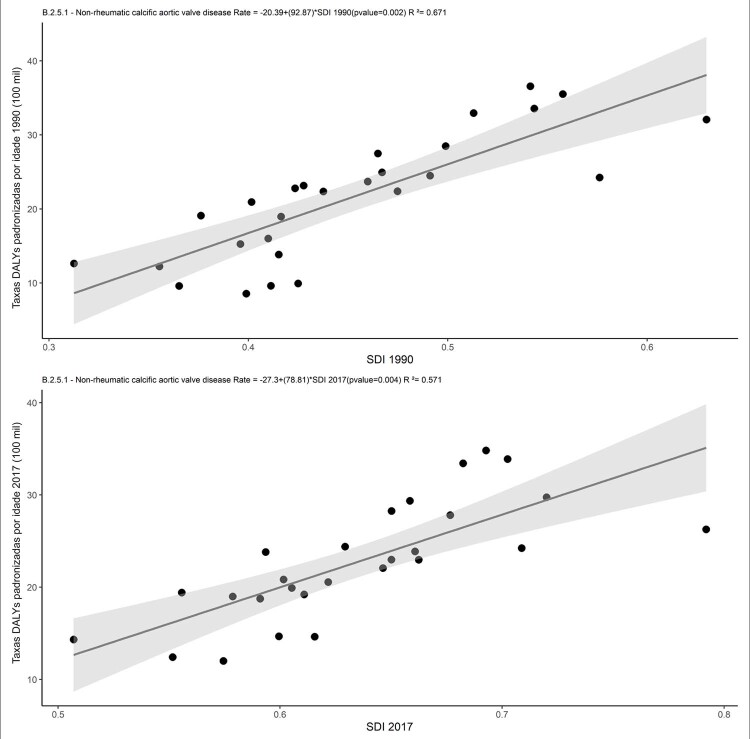
- Correlação entre as taxas de DALYs padronizadas por idade atribuíveis a doença valvar do coração não reumática e o Índice Sociodemográfico (SDI) nas unidades federativas brasileiras em 1990 e 2017.

Dados derivados do Estudo Global Burden of Disease 2017 (GBD 2017). ^236^

## 6. FIBRILAÇÃO ATRIAL E FLUTTER ATRIAL

### CID-10 I48

Ver Tabelas 6-1 até 6-7 e Figuras 6-1 até 6-3

**Table:** 

Abreviaturas usadas no Capítulo 6	
AIT	Ataque Isquêmico Transitório
AVC	Acidente Vascular Cerebral
BNP	Peptídeo Natriurético Cerebral
CID-10	Classificação Estatística Internacional de Doenças e Problemas Relacionados à Saúde, 10a Revisão
CRVM	Cirurgia de Revascularização do Miocárdio
DALYs	Anos de vida perdidos ajustados por incapacidade (do inglês, *Disability-Adjusted Life-Year* )
DCh	Doença de Chagas
DP	Desvio-Padrão
ECG	Eletrocardiograma
ELSA-Brasil	Estudo Longitudinal da Saúde do Adulto
FA	Fibrilação Atrial
GARFIELD-AF	*Global Anticoagulant Registry in the FIELD-AF*
GBD	*Global Burden of Disease*
HR	*Hazard Ratio*
IC	Intervalo de Confiança
II	Intervalo de Incerteza
IMPACT-AF	* A Multifaceted Intervention to Improve Treatment with Oral Anticoagulants in Atrial Fibrillation *
INR	Índice Internacional Normalizado (do inglês, *International Normalized Ratio* )
NOAC	Novos Anticoagulantes Orais
OR	*Odds Ratio*
PPC	Paridade do Poder de Compra
SDI	Índice Sociodemográfico (do inglês, *Sociodemographic Index* )
SUS	Sistema Único de Saúde
TTR	Tempo na Faixa Terapêutica (do inglês, *Time in Therapeutic Range* )
UF	Unidade Federativa
UTI	Unidade de Terapia Intensiva
AVK	Antagonista da Vitamina K

### Prevalência e Incidência

- De acordo com as estimativas do Estudo GBD 2017, a taxa de prevalência padronizada por idade de FA e *flutter* apresentou pequeno aumento no Brasil: de 619 (II 95%, 516-728) em 1990 para 641 (II 95%, 537-751) em 2017, por 100 mil habitantes, para ambos os sexos, com uma variação de 3,5% (II 95%, 1,7-5,3) no período. A prevalência de FA e *flutter* foi maior nos homens [em 1990: 759 (II 95%, 630-893); em 2017: 787 (II 95%, 657-925)] do que nas mulheres [em 1990: 499 (II 95%, 418-587); em 2017: 522 (II 95%, 440-610)], embora com maior variação percentual para as mulheres (4,6%; II 95%, 2,3-7) do que para os homens (3,7%; II 95%, 1,4-5,9) no período. Em números absolutos, as estimativas de prevalência de FA e *flutter* no Brasil subiram de 0,5 milhão em 1990 para 1,4 milhão em 2017, principalmente em razão do crescimento e envelhecimento da população (Tabela 6-1 e Figura 6-1). Em 2017, a proporção de pacientes com FA prevalente foi de 0,67% (II 95%, 0,56-0,78).
- De acordo com o Estudo GBD 2017, a taxa de prevalência de FA e *flutter* difere entre as UF brasileiras, tendo sido maior nas UF da região Sudeste em 1990 e 2017 (estados de São Paulo, Rio de Janeiro e Minas Gerais) e menor nas UF das regiões Nordeste ou Norte (Maranhão, Pernambuco e Alagoas em 1990, e Maranhão, Pernambuco e Pará em 2017). Isso pode resultar de um atraso na transição epidemiológica e da menor taxa de diagnóstico nas UF de mais baixa renda. Os homens apresentaram consistentemente taxas de prevalência mais altas nos dois períodos em todas as UF (Figura 6-1).
- De acordo com as estimativas do Estudo GBD 2017, as taxas de incidência padronizadas por idade por 100 mil por ano foram 44 (II 95%, 36-54) em 1990 e 46 (II 95%, 38-55) em 2017. Essas taxas foram também mais altas para os homens nos dois períodos [mulheres, 1990: 38 (II 95%, 31-45), 2017: 39 (II 95%, 32-47); homens, 1990: 53 (II 95%, 43-64), 2017: 54 (II 95%, 44-65)].
- Dados do ELSA-Brasil, um estudo de coorte com servidores públicos no Brasil de seis centros incluindo 14.424 adultos com ECG válido (45,8% homens, idade variando de 35 a 74 anos), mostraram prevalência de 0,3% (48 casos) de FA e *flutter* atrial (homens, 0,5%; mulheres, 0,2%) no ECG basal. A chance de apresentar FA ou *flutter* atrial aumentou com a idade para ambos os sexos, sendo a maior prevalência observada nos mais idosos, entre 65 e 74 anos (mulheres: OR: 17; IC 95%, 2,1-135,9; homens: OR: 52,3; IC 95%, 3,1-881,8). Não houve diferença na prevalência de acordo com a raça autorrelatada, para ambos os sexos (homens: negros, 0,3%; pardos, 0,4%; brancos, 0,6%; p=0,42; e mulheres: negras, 0,3%; pardas, 0,2%; brancas, 0,2%; p=0,84). ^237^
- O Registro GARFIELD-AF é um estudo mundial que incluiu pacientes (≥18 anos) com diagnóstico de FA nas 6 semanas anteriores e pelo menos um fator de risco adicional para AVC segundo avaliação do investigador do estudo. No Brasil, 41 *sites* (82,3% cardiologistas) incluíram 1.065 pacientes com FA não valvar entre 2010 e 2014 (idade média [DP]: 68 [13] anos; 55% homens). A prevalência dos tipos de FA foi a seguinte: primeiro episódio de FA, 52% dos pacientes; FA paroxística, 25%; FA persistente, 14%; e FA permanente, 8%. ^238^ A alta prevalência de primeiro episódio de FA pode ser atribuída ao critério de inclusão: FA diagnosticada nas 6 semanas anteriores.
- Outro estudo transversal de base populacional em uma área desassistida de São Paulo, incluindo 1.524 indivíduos com 65 anos ou mais de idade, relatou FA em 2,4% dos participantes (homens, 3,9%; mulheres, 2,0%). ^239^
- Os sistemas de telessaúde no Brasil forneceram vastos dados epidemiológicos sobre arritmias, ao incluir pacientes atendidos nas unidades de atenção primária e de emergência. Entre 262.685 pacientes dos centros de atenção primária submetidos a ECG através de telessaúde em Minas Gerais em 2011, a prevalência de FA foi 1,8% e aumentou bruscamente com a idade (OR: 1,08; IC 95%, 1,07-1,08), tendo sido maior nos homens (2,4%) em todos os grupos etários, variado de 0,2% (20-29 anos de idade) a 14,6% (nonagenários), em comparação às mulheres (1,3%), variando de 0,1% (20-29 anos de idade) a 8,7% (nonagenárias), (OR: 1,77; IC 95%, 1,68-1,89). ^240 , 241^
- O serviço de telemedicina da Universidade Federal de São Paulo analisou 676.621 ECG de pacientes (idade média 51±19 anos; 57,5% mulheres) em visitas rotineiras a 125 centros de atenção primária de janeiro de 2009 a abril de 2016. A prevalência de FA em 7 anos foi de 2,2% (n = 14.968). O estudo projetou uma prevalência de FA no Brasil para 2025 de 1,7%. ^246^
- De 260.879 ECG realizados entre março e setembro de 2015 em centros de atenção primária usando um sistema de telessaúde em Minas Gerais, 304 (0,1%) foram estratificados como emergência cardiovascular. Fibrilação atrial e *flutter* com resposta ventricular baixa ou alta foram a causa de 22% dessas emergências. ^243^
- A prevalência de FA em 1.518 indivíduos (idade média 58±16 anos; 66% mulheres), que aguardavam por um ecocardiograma na atenção primária e foram rastreados para FA com um dispositivo portátil, foi de 6,4%. Idade mais avançada foi um fator de risco (9,3% vs. 4,8% naqueles com idade acima e abaixo de 65 anos, respectivamente, P=0,001), tendo FA sido associada com doença cardíaca no ecocardiograma (OR: 3,9; IC 95%, 2,1-7,2, p < 0,001). Os autores sugerem que o rastreio de FA possa ser uma ferramenta útil na atenção primária para estratificar risco e priorizar o ecocardiograma. ^244^

### Mortalidade

- De acordo com o Estudo GBD 2017, o número de mortes por FA no Brasil aumentou nos últimos anos em razão do crescimento e envelhecimento da população. Na década de 1990, a FA foi responsável por 2.649 (II 95%, 2.428-2.836) mortes, que subiram para 10.059 (II 95%, 9.390-10.698) em 2017. Entretanto, a taxa de mortalidade padronizada por idade por FA permaneceu estável, sendo 4,7 (II 95%, 4,4-5,1) por 100 mil habitantes em 1990 e 4,8 (II 95%, 4,5-5,2) por 100 mil habitantes em 2017, correspondendo a 0,7% de todas as mortes no país. Embora as taxas de prevalência padronizadas por idade fossem consistentemente mais altas nos homens, as mulheres apresentaram maiores taxas de mortalidade padronizadas por idade em 2017 (mulheres: 4,9, II 95%, 4,8-5,3; homens: 4,6, II 95%, 3,9-5,2), dados coerentes com os de outros países. ^245 , 246^ Ao considerar indivíduos com idade >70 anos, a taxa de mortalidade aumenta de 1990 (52, II 95%, 48-55) a 2017 (74, II 95%, 69-79). É importante notar que, como a mortalidade baseada apenas em dados de registro vital fornece um implausível aumento vertiginoso ao longo do tempo, possivelmente em consequência de mudanças na apuração mais do que da epidemiologia da FA, o Estudo GBD 2017 presume, a *priori* , que as taxas de mortalidade específicas para idade e sexo não estejam aumentando nem diminuindo com o tempo. ^247^ Assim sendo, as pequenas variações com o tempo aqui relatadas são intencionalmente mais baixas do que as reais variações em dados brutos.
- A Tabela 6-2 mostra o número total de mortes, a taxa de mortalidade padronizada por idade por FA (por 100 mil habitantes, ambos os sexos) e a variação percentual, por UF e no Brasil, em 1990-2017. As UF com os maiores percentuais de redução observados entre 1990 e 2017 foram Espírito Santo, Roraima, Goiás e Minas Gerais, nessa ordem. Por outro lado, as UF com os maiores percentuais de aumento observados entre 1990 e 2017 foram Distrito Federal, Alagoas e Sergipe. Entretanto, esses dados devem ser interpretados com cautela, pois pode haver inconsistência em virtude de questões de notificação. As Tabelas 6-3 e 6-4 mostram os dados estratificados por sexo.
- Com relação às taxas de mortalidade por FA de acordo com os grupos etários, o aumento mais significativo foi observado nos mais idosos: grupo de 70+ anos (51,6, II 95%, 47,7-55,3 por 100 mil em 1990; e 74, II 95%, 69,3-78,7 por 100 mil em 2017), com variação percentual de 43,4 (II 95%, 35,9-50,6), seguido pelo grupo de 50-69 anos (2,6, II 95%, 2,3-2,8 por 100 mil em 1990; e 2,8, II 95%, 2,5-3,0 por 100 mil em 2017), com variação percentual de 6,0 (II 95%, 0,3-10,9).
- O Estudo GBD 2017 usa o SDI como uma estimativa do nível socioeconômico da localidade. Como ilustrado na Figura 6-2, não há correlação estatisticamente significativa entre a taxa de mortalidade padronizada por idade por FA e *flutter* por 100 mil habitantes e o SDI das UF brasileiras (p=0,37), provavelmente porque a prevalência seja mais baixa nas UF de mais baixa renda, como já mencionado.
- Em uma análise prospectiva do estudo de coorte de Bambuí, que incluiu todos os moradores dessa localidade, área endêmica de DCh, com idade ≥ 60 anos em 1 de janeiro de 1997, um total de 1.462 participantes com (38%) e sem DCh [idade média de 69 (63-74) anos; 61% mulheres] foi seguido por 10 anos. Morte ocorreu em 556 participantes. A presença de FA ou *flutter* na linha de base foi independentemente associada com aumento da mortalidade por todas as causas (HR: 2,35; IC 95%, 1,53-3,62) nos pacientes com e sem DCh (HR: 1,92; IC 95%, 1,05-3,51). ^248^
- Um estudo avaliando 302 pacientes [idade média (DP), 58 (15) anos; 53% mulheres] com FA valvar (32%) e FA não valvar, seguidos por 1 ano, mostrou taxa de mortalidade de 10% e nenhuma diferença entre os portadores de FA valvar e não valvar. As causas de morte foram insuficiência cardíaca em 25 pacientes (83%), morte súbita cardíaca em 3 (10%) e trombose em prótese valvar mecânica em 2 (7%). ^249^

### Carga de Doença

- De acordo com as estimativas do GBD 2017, a FA resultou em 226.810 (II 95%, 187.976-272.166) DALYs no Brasil em 2017, que representam 0,37% de todos os DALYs. A taxa de DALYs padronizada por idade foi 104 (II 95%, 86-124) por 100 mil em 2017, maior para homens (115; II 95%, 92-141) do que para mulheres (93; II 95%, 79-109), embora a proporção de DALYs seja maior nas mulheres (0,43%; II 95%, 0,39-0,48) do que nos homens (0,33%; II 95%, 0,28-0,39). Em comparação a 1990, houve pequeno aumento nas taxas de DALYs no país de 4,6% (UI 95%, 1,7-7,2). O número de DALYs e taxas de DALYs por FA e *flutter* em 1990 e 2017, de acordo com as UF brasileiras, para ambos os sexos, são mostrados nas Tabelas 6-5 a 6-7.
- Não se encontrou associação entre o SDI e as taxas de DALYs padronizadas por idade por FA no Brasil, à semelhança do relatado para as taxas de mortalidade padronizadas por idade (Figura 6-3).

### Utilização e Custo da Atenção à Saúde

#### (Ver Tabelas 1-6 a 1-9 e Figuras 1-15 e 1-16)

- De 2008 a 2018, houve 321.866 hospitalizações por FA e 1.250 procedimentos de ablação para FA e *flutter* foram realizados pelo SUS, com custos não ajustados de R$ 231.850.160 e R$ 6.950.612, respectivamente. Após ajuste para a inflação brasileira, os custos passaram a R$ 418.504.911 e R$ 12.546.315, respectivamente. Em dólares internacionais convertidos em PPC ajustados para US$ 2018, os valores foram $ 403.520.568 e $ 6.701.749, respectivamente.
- Uma análise da carga econômica das condições cardíacas no Brasil estimou uma prevalência de FA de 0,8% (1.202.151 casos) em 2015. Os autores estimaram um custo total para FA de R$ 3.921 milhões (US$ 1,2 bilhão). ^250^

### Complicações

#### Acidente Vascular Cerebral

- Um registro de AVC na cidade de Joinville descreveu todos os 429 casos de AVC que ocorreram em 2015, dos quais, 87,2% (374/429) foram AVC isquêmicos. Fibrilação atrial foi detectada em 11,4% (49/429) dos pacientes e em 58% (49/84) de todos os AVC cardioembólicos. ^251^ Similarmente, detectou-se FA em 58% de 359 pacientes com AVC cardioembólico em uma amostra consecutiva de um único centro em Curitiba. ^252^
- Em uma análise retrospectiva de 215 pacientes hospitalizados por AVC, FA ocorreu em 16,3%, tendo havido associação de FA com o avançar da idade: enquanto a prevalência de FA foi 5% em pacientes com idade <65 anos, naqueles com idade > 80 anos, a prevalência de FA aumentou para 26% (p=0,01). ^253^
- Entre pacientes hospitalizados por AVC isquêmico agudo ou AIT, um sistema de escore para diagnóstico de FA com base em variáveis clínicas e ecocardiográficas foi desenvolvido e validado em uma coorte brasileira. Idade (OR: 1,04; IC 95%, 1,02-1,08), uso da escala do *National Institutes of Health* para avaliar AVC na admissão (OR: 1,10; IC 95%, 1,05-1,16) e aumento do átrio esquerdo (OR: 2,5; IC 95%, 1,01-6,29) foram preditores de FA e AIT (estatística-C = 0,76; IC 95%, 0,69-0,83). ^254^

#### Insuficiência Cardíaca

- Em um estudo retrospectivo de 659 pacientes com hospitalizações consecutivas por insuficiência cardíaca descompensada em 2011, a prevalência de FA foi 40% (73% de FA permanente). Fibrilação atrial foi associada com o avançar da idade (p < 0,0001), etiologia não isquêmica (p = 0,02), disfunção ventricular direita (p = 0,03), pressão sistólica mais baixa (p = 0,02), maior fração de ejeção (p < 0,0001) e aumento do átrio esquerdo (p < 0,0001). Pacientes com FA apresentaram maior mortalidade hospitalar (11,0% vs *.* 8,1%, p = 0,21) e mais longa permanência hospitalar (20,5 ± 16 dias vs *.* 16,3 ± 12, p = 0,001). ^255^

#### Quedas

- Dados de um estudo retrospectivo incluindo 107 pacientes com FA e idade média de 78 anos revelaram que 51% haviam relatado pelo menos uma queda no ano anterior. O risco foi maior entre pacientes com diabetes e em uso de amiodarona. ^256^

#### Demência

- Em um estudo transversal com 1.524 participantes com idade > 65 anos, demência foi diagnosticada em 11% daqueles com FA em comparação a 4% daqueles sem FA (p=0,07). Os autores encontraram OR para demência de 2,8 (IC 95%, 1,0-8,1; p = 0,06) entre indivíduos com FA. ^257^

#### Tipos de Fibrilação Atrial e Complicações

- Em um estudo retrospectivo, dados de 407 pacientes tratados para FA na emergência da cardiologia no primeiro trimestre de 2012 revelaram que as prevalências de insuficiência cardíaca e de AVC foram maiores em pacientes com FA persistente (n=188) e *flutter* (n=51) do que naqueles com FA paroxística (n=168) (insuficiência cardíaca: 51,2% vs. 45,1% vs. 19,7%; p < 0,01; e AVC: 10,7% vs. 9,8% vs. 1,6%; p < 0,01). ^258^

###  Influência dos Fatores de Risco em Fibrilação Atrial e Flutter 1

- O Registro GARFIELD-AF, um estudo observacional sem intervenção, de âmbito mundial, incluiu 1.065 pacientes (≥ 18 anos) com FA não valvar diagnosticada nas 6 semanas anteriores em 41 *sites* no Brasil (82,3% cardiologistas). A idade média (DP) foi 68 (13) anos e 55% eram homens. Tabagismo atual/prévio foi encontrado em 32%, hipercolesterolemia, em 42%, obesidade, em 29%, diabetes, em 25% e hipertensão, em 81% dos pacientes. ^2^ ^38^
- Em uma amostra de 262.685 pacientes em centros de atenção primária, submetidos a ECG usando-se telessaúde em Minas Gerais em 2011, a prevalência autorreferida de fatores de risco entre aqueles com FA foi: hipertensão, 51,8%; diabetes, 7,3%; tabagismo, 6,7%; dislipidemia, 3,5%. Apenas hipertensão apresentou associação significativa com FA na comparação com indivíduos sem FA (51,8% vs. 31,7%; OR ajustada para idade e sexo: 1,32; IC 95%, 1,24-1,40).
- Um estudo transversal comparando indivíduos com FA a controles saudáveis encontrou maior frequência de apneia do sono no grupo de FA em comparação ao grupo controle (81,6% vs. 60%, p = 0,03). ^259^

### Doenças Associadas e Comorbidades

- A incidência de FA entre 300 idosos (idade média, 75±8 anos; 56% mulheres) monitorados com marcapassos, sem FA na linha de base, foi 22% em um seguimento de 435 dias, ^2^ ^60^ chegando a 85% dos pacientes com marcapassos e doença renal crônica em seguimento de 1 ano. ^261^
- Em pacientes com doença cardiovascular que procuraram a emergência, a prevalência de FA foi 40% naqueles com insuficiência cardíaca descompensada ^255^ e 44% naqueles com doença valvar cardíaca. ^262^
- Um estudo incluindo pacientes de uma UTI encontrou incidência de FA de 11% durante a permanência na unidade. ^263^

####  Fibrilação Atrial Peroperatória e Cirurgia Cardiovascular

- No pós-operatório de cirurgia cardíaca, FA ocorreu em 12% a 33% dos pacientes. ^264^ ^- 266^ As cirurgias de substituição valvar foram associadas com maior ocorrência de FA (31%-33%) durante a hospitalização em comparação à CRVM (12%-16%).
- Idade avançada, doença valvar mitral e não uso de betabloqueadores foram associados com FA no pós-operatório de cirurgia valvar. ^267^ Entre aqueles submetidos a CRVM, a incidência de FA no pós-operatório foi associada com átrio esquerdo > 40,5 mm e idade > 64,5 anos. ^268^

#### Fibrilação Atrial e Doença de Chagas

- Relatos de caso descreveram FA em pacientes com DCh aguda adquirida por via oral, ^269^ provavelmente relacionada com miocardite chagásica aguda.
- No estudo de coorte de Bambuí, 1.462 participantes com idade ≥ 60 anos (idade média, 69 anos; DCh n=557, 38,1%) e ECG na linha de base foram seguidos por 10 anos; o desfecho foi mortalidade. Fibrilação atrial foi mais frequentemente observada nos indivíduos com DCh, 6,1% vs. 3,4% (OR: 3,43; IC 95%, 1,87-6,32, ajustada para idade, sexo e variáveis clínicas), e foi um fator de risco independente para morte (HR: 2,35; IC 95%, 1,53-3,62, ajustada para idade, sexo, variáveis clínicas e níveis de BNP) nos indivíduos com DCh. ^248^
- Em uma grande amostra de 264.324 pacientes submetidos a tele-ECG em uma unidade de atenção primária à saúde em 2011, DCh foi autorrelatada por 7.590 (2,9%). A idade média dos indivíduos com DCh foi 57,0 (DP: 13,7) anos e a daqueles sem DCh foi 50,4 (DP: 19,1) anos, com 5% de octogenários nos dois grupos. Fibrilação atrial foi observada em 5,35% dos indivíduos com DCh e em 1,65% daqueles sem DCh (OR: 3,15; IC 95%, 2,83-3,51, ajustada para idade, sexo e comorbidades autorrelatadas). Nos octogenários, a prevalência de FA alcançou 16,26% dos pacientes: 15,47% das mulheres e 17,95% dos homens. ^270^
- Na linha de base do estudo de coorte do Projeto NIH-SaMi-Trop, que recrutou pacientes chagásicos (n=1.959; 67,5% mulheres; mediana da idade 59, Q1-Q3: 49-69 anos) com suspeita de doença cardíaca em 21 municípios do norte de Minas Gerais, a prevalência de FA foi 4,5%, sendo maior em homens do que em mulheres (5,6 vs. 2,6%, p< 0,001) e naqueles com níveis altos em comparação a normais de NT-ProBNP (14,6% vs. 2,1%, p< 0,001). ^271^
- Em uma revisão sistemática e meta-análise, Rojas *et al* . avaliaram a frequência de anormalidades eletrocardiográficas da DCh na população geral. Foram selecionados 49 estudos, incluindo 34.023 pacientes (12.276 chagásicos e 21.747 não chagásicos). A prevalência de FA foi significativamente mais alta nos chagásicos (OR: 2,11; IC 95%, 1,40-3,19). ^272^
- Em uma amostra de 424 pacientes chagásicos com idade inferior a 70 anos [41,7% mulheres; idade média, 47 (DP:11)], seguidos por 7,9 (DP: 3,2) anos, Rassi *et al* . encontraram prevalência de FA de 13,3 (DP: 3,1%), com forte associação a risco de morte (HR: 5,43; 2,91-10,13) em análise univariada. ^273^
- Em 330 pacientes com DCh (idade média, 49±12 anos; 58% homens), 26 dos quais com FA, uma análise explorando os fatores de risco para eventos cerebrovasculares isquêmicos não encontrou aumento do risco de AVC entre os chagásicos com FA em comparação aos chagásicos em ritmo sinusal. ^274^

### Conscientização, Tratamento e Controle

#### Anticoagulação

- Houve grande variação no uso de anticoagulação entre os pacientes com FA, de 1,5% a 91%. Estudos com amostras da atenção primária apresentaram maior probabilidade de baixo uso de anticoagulação em comparação a amostras recrutadas de centros terciários ou de cardiologistas, como detalhado a seguir.
- Entre 4.638 indivíduos com FA em centros de atenção primária de 658 municípios em Minas Gerais [idade média (DP), 70 (14) anos; 54% homens], submetidos a ECG através de telessaúde em 2011, o uso de AVK foi relatado por 1,5% e o de aspirina, por 3,1%. ^241^
- Em um estudo de pacientes de 125 centros de atenção primária em 9 estados de 4 regiões geográficas brasileiras, de janeiro de 2009 a abril de 2016, identificou-se um subconjunto de pacientes com FA (n=301), 189 (63%) dos quais com alto risco de AVC; apenas 28 (15%) faziam uso regular de anticoagulantes orais e 102 (54%), de aspirina. ^242^
- O Registro GARFIELD-AF é um estudo mundial que incluiu pacientes (≥18 anos) com diagnóstico de FA nas 6 semanas anteriores e pelo menos um fator de risco adicional para AVC segundo avaliação do investigador do estudo. No Brasil, entre os 1.041 pacientes incluídos (82,3% por cardiologistas) entre 2010 e 2014, a média (DP) de idade foi 68 (13) anos, 55% eram homens, 86% tinham escore CHA _2_ DS _2_ -VASc ≥2, 19% não usavam anticoagulação na linha de base, 26% estavam recebendo apenas terapia antiplaquetária, 29% estavam usando AVK e 26% estavam usando NOAC. ^238^
- O IMPACT-AF, ^275^ um ensaio randomizado por *cluster* para aperfeiçoar o tratamento com anticoagulantes em pacientes com FA, conduzido na Argentina, China, Índia, Romênia e no Brasil, mostrou que dois terços dos pacientes usavam anticoagulação oral na linha de base: 83%, AVK e 15%, um NOAC. Dos pacientes do Brasil (n=360), 91% usavam anticoagulação oral na linha de base e 27% usavam NOAC. De todos os pacientes tomando AVK no Brasil, 40,3% apresentavam valores de INR entre 2 e 3 antes da visita na linha de base.
- Um estudo transversal incluindo 162 pacientes de um hospital universitário em Porto Alegre [idade média (DP), 69 (12) anos; 57% homens; 96% com CHA _2_ DS _2_ -VASc ≥2] identificou 55 (34%) pacientes em uso de anticoagulantes e 80 (50%), de aspirina. ^276^
- Um estudo transversal de um hospital universitário na cidade do Rio de Janeiro incluiu 659 hospitalizações consecutivas por insuficiência cardíaca descompensada de janeiro de 2006 a dezembro de 2011. Os pacientes com FA (n=264; 40%) apresentaram uma mediana de 4 do escore CHA _2_ DS _2_ -VASc, que foi ≥2 em 90% dos pacientes. A taxa de anticoagulação foi 53% na admissão e 67% na alta. ^255^
- Um registro de AVC na cidade de Joinville descreveu todos os 429 casos de AVC que ocorreram em 2015, dos quais, 87,2% (374/429) foram AVC isquêmicos. Fibrilação atrial foi detectada em 11,4% (49/429) dos pacientes e em 58% (49/84) de todos os AVC cardioembólicos. Dos 26 pacientes que sabidamente tinham FA prévia, 19 (73%) não estavam anticoagulados, 20 (77%) tinham escore CHA _2_ DS _2_ -VASc ≥ 3 e 21 (81%) tinham escore HAS-BLED < 3. ^251^
- A qualidade da terapia com varfarina foi avaliada usando TTR como parâmetro em diferentes amostras no Brasil, que, em geral, não são compostas apenas por portadores de FA. Os pacientes com FA em uso de anticoagulantes apresentaram pior TTR em comparação àqueles com outras indicações, como próteses valvares. O TTR variou entre 46% e 67% nos estudos. ^249 , 277 - 279^ Idade > 65 anos, mas não letramento em saúde, foi associada com um TTR mais longo. ^278^

####  Controle de Ritmo ou Frequência (Medicamento, Cardioversão, Ablação por Cateter)

- Um estudo transversal com 167 pacientes com FA relatou que controle da frequência foi mais comum do que controle do ritmo como estratégia de tratamento (79% vs. 21%; p < 0,001). No subgrupo de FA paroxística, ambas as estratégias foram igualmente usadas (controle da frequência, 53%; controle do ritmo, 47%; p = 0,69). Pacientes com FA persistente apresentaram maior probabilidade de serem tratados com controle de frequência (96% vs. 4%; p < 0,001). Entre aqueles tratados com controle de ritmo, as drogas mais prescritas foram amiodarona (43%), sotalol (16%) e propafenona (14%). Os betabloqueadores foram prescritos para 81% dos pacientes em tratamento com controle de frequência. ^280^ Amiodarona foi mencionada por 83% dos médicos como a escolha para controle de ritmo. ^281^
- Dados de 125 centros de atenção primária mostraram que, entre os pacientes com FA (n=301), 91 (30,2%) não recebiam qualquer tipo de tratamento para controle de frequência ou ritmo. Dos 210 pacientes restantes em tratamento, 147 (70%) usavam agentes para controle de frequência (betabloqueadores, digoxina, diltiazem ou verapamil) e 25 (12%) usavam pelo menos um antiarrítmico (amiodarona ou propafenona). O uso simultâneo de antiarrítmicos e betabloqueadores foi relatado por 36 (17%) respondentes. ^242^
- Ablação de FA foi relatada no Primeiro Registro de Ablação Transcateter da América Latina, que incluiu 742 pacientes de ablação de FA, tanto procedimentos de primeira vez quanto repetição, realizados entre 1 de janeiro e 31 de dezembro de 2012 em 18 centros no Brasil. ^282^
- Em uma série de casos de um único centro, 225 pacientes com FA paroxística [64 mulheres (29%) e 161 homens (61%)] foram submetidos a ablação por cateter, havendo recorrência de 21% entre as mulheres e de 20% entre os homens (p = 0,89) em seguimento de 1 ano. ^283^

### Pesquisa Futura

- O estudo RECALL, o primeiro registro cardiovascular brasileiro de FA, encerrou a inclusão de pacientes em 2019, com 4.584 indivíduos, e deverá ser publicado em 2020. Será o maior registro brasileiro com dados de todas as regiões do país, relacionados às características e ao tratamento de pacientes com FA de 73 centros no Brasil. ^284^
- Estudos de coorte em andamento, como o ELSA-Brasil, têm potencial para preencher os hiatos de informação sobre incidência, fatores de risco, predição de risco – incluindo genética – e prevenção de FA no Brasil. Até onde se sabe, não há estudo original publicado com informação sobre a incidência de FA no Brasil nem dados longitudinais sobre fatores de risco.
- Estudos desenhados para rastrear FA em uma base populacional ou populações selecionadas através do uso de ECG ou dispositivos de rastreio estão em andamento e deverão fornecer informação sobre a relevância da inclusão dessa estratégia em centros de atenção primária ou especializados.
- Estratégias de implementação para aumentar o uso da anticoagulação entre pacientes com FA devem ser encorajadas, em particular no contexto da atenção primária.

**Tabela 6-1:** – Número de casos prevalentes e prevalência padronizada por idade de fibrilação atrial e *flutter* , por 100 mil habitantes, em 1990 e 2017, com variação percentual, para ambos os sexos e para mulheres e homens, no Brasil e suas unidades federativas

	Ambos os sexos			Mulheres			Homens		
Brasil e unidades federativas	1990 (II 95%)	2017 (II 95%)	Variação percentual (II 95%)	1990 (II 95%)	2017 (II 95%)	Variação percentual (II 95%)	1990 (II 95%)	2017 (II 95%)	Variação percentual (II 95%)
Acre	585.2 (482.8;692.5)	600.8 (493.1;714.2)	2.7 (7.1;-2)	487.9 (408.1;569.3)	508.3 (427;596.5)	4.2 (10.3;-2.2)	669.8 (540.1;806.8)	697.2 (555;849.8)	4.1 (10.6;-2.5)
Alagoas	426.1 (308.3;568)	428.7 (312.2;556.5)	0.6 (5.9;-5)	320.2 (221.7;432.7)	322.5 (225.4;434.5)	0.7 (7.5;-6.3)	546.6 (398.9;735.7)	562.5 (421.5;734.3)	2.9 (10.7;-5.3)
Amapá	673.7 (580.9;766.1)	696.7 (600.5;800.2)	3.4 (7.3;-0.5)	538.9 (464;616.7)	559.2 (483.3;643.2)	3.8 (8.8;-1.3)	816.7 (703.6;936.5)	848.1 (726.9;976.7)	3.8 (10.1;-1.8)
Amazonas	482.5 (375.5;610.5)	503 (385.7;636.4)	4.3 (9.9;-0.4)	349.6 (257.3;461.3)	360.5 (264.6;476.8)	3.1 (9.9;-3.1)	620 (490.9;766.8)	655.3 (510;813)	5.7 (13;-0.7)
Bahia	447.3 (332.2;579.6)	469.7 (346.1;606.8)	5 (10.1;0.1)	336.9 (242.4;445.2)	359.7 (258.1;475.1)	6.8 (13.6;-0.5)	574.2 (436.2;733.4)	605.7 (454;772.5)	5.5 (14;-1.6)
Brasil	619 (515.5;727.6)	640.9 (537;751)	3.5 (5.3;1.7)	499.3 (417.9;586.5)	522.1 (439.9;610.1)	4.6 (7;2.3)	758.9 (630;893.1)	786.9 (656.6;925)	3.7 (5.9;1.4)
Ceará	470.1 (361.8;598.3)	481.7 (368.6;612.8)	2.5 (7.1;-1.9)	338.8 (242.3;450.9)	348.8 (250.2;463.5)	2.9 (9.6;-3.7)	618.3 (486.1;767.3)	645.7 (505.6;803.7)	4.4 (10.7;-2.2)
Distrito Federal	649.2 (560.8;745.1)	665.2 (569.5;769.2)	2.5 (7.2;-1.6)	528.1 (456.8;602.9)	547.3 (468.8;629.1)	3.6 (9.8;-1.9)	796.8 (686.4;920.9)	824.8 (698.9;968.1)	3.5 (10.7;-2.1)
Espírito Santo	483.6 (371.6;608.6)	505 (386.6;644.5)	4.4 (9.4;-0.7)	381 (295.8;478.9)	401.9 (311.9;509.7)	5.5 (13.1;-0.8)	597 (453;756.4)	629.5 (479.9;809)	5.4 (12.5;-0.9)
Goiás	487.3 (378.1;605.3)	511.8 (397.7;643.5)	5 (10.8;-0.1)	438.8 (359.7;524.3)	461.1 (377.2;557.3)	5.1 (12;-1.8)	533.3 (381.5;695.6)	568.7 (412.3;754.8)	6.6 (14.8;-1.1)
Maranhão	377.7 (251.2;546.2)	351.4 (234.6;502)	-7 (-1.9;-13.4)	300.6 (197.9;439.7)	281.1 (185.6;398.6)	-6.5 (0.1;-14.7)	467.9 (312.4;676.6)	432.4 (290.1;621.1)	-7.6 (0.1;-16)
Mato Grosso	537.9 (432.1;652)	558.1 (443.3;686)	3.8 (8.6;-1.1)	456.4 (381.1;543.6)	477.2 (393.2;572.4)	4.6 (11.2;-1.6)	607.2 (471.4;759.2)	637.4 (491.4;812.9)	5 (11.6;-1.8)
Mato Grosso do Sul	678.5 (588.7;774.3)	690.4 (594.9;796.2)	1.8 (6.6;-2.3)	532.6 (459.2;608.8)	551.4 (477.8;631.7)	3.5 (9.7;-1.9)	815.8 (706.7;938.3)	845.2 (719.9;986)	3.6 (10.2;-2.2)
Minas Gerais	716.3 (620.7;815.9)	732.2 (646.8;820.9)	2.2 (7.8;-2.6)	554.1 (476.8;635)	565.7 (498.8;638.4)	2.1 (9;-3.9)	907.1 (783.5;1034.5)	930.7 (822.7;1040.7)	2.6 (9;-2.9)
Pará	426.2 (310.8;561)	434.7 (314;575.1)	2 (7.4;-3.1)	312 (218.4;434.1)	309.5 (215.6;429.8)	-0.8 (6.5;-7.7)	549.8 (406.6;719.6)	569.2 (416;743)	3.5 (9.7;-3.5)
Paraíba	507.8 (405.5;622)	527.4 (418.9;650.3)	3.9 (8.1;-0.2)	417.8 (341.3;505.7)	438.5 (352.9;531.2)	5 (11;-1.2)	610.3 (472.6;762.7)	640.1 (498.3;810.1)	4.9 (11.5;-1.8)
Paraná	588.3 (488.1;692.4)	607.7 (502.3;721.1)	3.3 (7.6;-0.9)	493.9 (418.7;573.1)	514.7 (434.7;602.9)	4.2 (10;-2)	686.9 (554.8;827.8)	719 (576.4;870.9)	4.7 (12.5;-1)
Pernambuco	422.7 (304.4;559.1)	442 (317.7;588.2)	4.6 (10.1;-0.4)	335.9 (238.6;447.7)	354.9 (255.2;473.6)	5.7 (14;-1.6)	528.9 (379.7;703.9)	558.1 (400.4;750.7)	5.5 (11.9;-1.7)
Piauí	429.2 (311.3;569)	437.6 (318.1;577.5)	2 (7.4;-2.4)	322 (227.4;434.3)	327.8 (229.5;439.6)	1.8 (9.4;-5.2)	550.2 (408.1;719.3)	568.7 (417.2;750.2)	3.4 (9.7;-2.6)
Rio de Janeiro	723.3 (626.3;827)	742.4 (646.8;845.8)	2.6 (6.6;-1.4)	567.3 (487;652.3)	585 (506.3;671.8)	3.1 (8.3;-2.2)	934.3 (812;1071.9)	956.7 (831.5;1094.2)	2.4 (8.2;-3.3)
Rio Grande do Norte	458.2 (345.8;587)	467.1 (346.5;603.8)	1.9 (7.6;-3.1)	337.9 (242.7;446.4)	347.7 (247;466.6)	2.9 (11.2;-4.7)	592.8 (458.7;744.5)	617 (468.6;789.3)	4.1 (11;-3)
Rio Grande do Sul	509.1 (399;629.9)	534.9 (418.9;668)	5.1 (9.5;0.3)	435.5 (351.1;526.3)	456.5 (367.2;558.1)	4.8 (11.9;-1.6)	604.9 (449;773)	635.8 (476.4;823.5)	5.1 (11.8;-0.9)
Rondônia	515.5 (408.2;637)	529.1 (416;655)	2.6 (7.3;-2.3)	436.6 (357.1;521.5)	455.1 (371.5;547.6)	4.2 (11.1;-2.2)	580.5 (446;741.2)	601.5 (453.1;767.6)	3.6 (10.8;-3)
Roraima	702.9 (609.8;804.9)	741.4 (646.1;844.4)	5.5 (9.5;1.6)	523.1 (451.2;603.1)	562.9 (488.7;641.4)	7.6 (13.8;2)	843.5 (728.4;969)	909.8 (793.3;1039.6)	7.9 (13.4;2.4)
Santa Catarina	617.1 (516.7;722.4)	641.9 (539.1;754.4)	4 (8.5;-0.2)	561.6 (482.9;640.9)	582.5 (504;667.2)	3.7 (9.4;-2.4)	679.5 (544.4;820.5)	711.7 (566.7;873)	4.7 (11.1;-1.6)
São Paulo	849.8 (736.7;972.7)	865.6 (753.4;984.3)	1.9 (6.1;-1.9)	702.6 (605.4;807.1)	726.4 (629.4;832.9)	3.4 (9.7;-1.6)	1028.3 (890.9;1176.3)	1042.9 (903.6;1190.2)	1.4 (6.8;-4.1)
Sergipe	518.5 (419.8;629.8)	536.3 (436.6;654.6)	3.4 (8.5;-1.3)	390.7 (312.6;481.3)	408.3 (322.8;515.6)	4.5 (11.9;-2.6)	669.2 (543.8;805.3)	699.4 (570.4;847.3)	4.5 (10.9;-1.6)
Tocantins	613.3 (518.7;713.7)	632.8 (532.1;743)	3.2 (8.2;-1.6)	448.1 (372.1;527.4)	465.7 (385.1;557.3)	3.9 (9.7;-1.4)	765.1 (651.1;890.6)	794.5 (668.7;927.2)	3.8 (10.3;-2.9)

* Fonte: Estudo Global Burden of Disease 2017, Institute for Health Metrics and Evaluation. ^285^*

**Tabela 6-2:** – Número de mortes e taxa de mortalidade padronizada por idade por fibrilação atrial e *flutter* , por 100 mil habitantes, em 1990 e 2017, com variação percentual, no Brasil e suas unidades federativas

	1990		2017		
	Número de mortes (II 95%)	Taxa de mortalidade (II 95%)	Número de mortes (II 95%)	Taxa de mortalidade (II 95%)	Variação percentual (II 95%)
Acre	4 (4;5)	4.9 (4.5;5.5)	22 (20;24)	4.5 (4.1;4.8)	-8.6 (-18.4;-0.9)
Alagoas	35 (33;41)	3.5 (3.3;4.1)	119 (112;135)	4.1 (3.8;4.7)	16.1 (4.2;26.2)
Amapá	3 (3;3)	5.8 (5.4;6.5)	18 (16;19)	5.3 (4.7;5.6)	-9.6 (-20.2;-2.2)
Amazonas	19 (17;22)	3.9 (3.7;4.7)	87 (82;96)	4 (3.8;4.5)	3.2 (-11.6;13)
Bahia	236 (219;265)	4.2 (3.9;4.8)	719 (675;798)	4.3 (4.1;4.8)	2.3 (-6.1;10.2)
Brasil	2649 (2428;2836)	4.7 (4.4;5.1)	10059 (9390;10698)	4.8 (4.5;5.2)	2.5 (-2.9;7.3)
Ceará	128 (117;149)	3.5 (3.2;4.1)	404 (380;443)	3.9 (3.6;4.3)	10.9 (-1.7;21.9)
Distrito Federal	13 (12;14)	6.4 (6.1;7)	97 (88;108)	7.6 (6.8;8.4)	19.8 (5.5;32.9)
Espírito Santo	41 (37;44)	6.2 (5.7;6.7)	169 (157;188)	4.5 (4.1;5)	-28.3 (-33.7;-21)
Goiás	43 (38;46)	4.9 (4.5;5.2)	226 (206;246)	4.3 (3.9;4.6)	-13.7 (-20.1;-5.6)
Maranhão	85 (55;101)	4.9 (3;5.8)	305 (219;342)	5.1 (3.6;5.7)	4.1 (-6.7;26.3)
Mato Grosso	18 (16;21)	4.1 (3.8;5.1)	99 (92;111)	4.4 (4.1;4.9)	5.6 (-8.3;16.1)
Mato Grosso do Sul	24 (22;26)	5.1 (4.7;5.5)	110 (103;122)	4.8 (4.5;5.3)	-6.6 (-13.3;2.2)
Minas Gerais	279 (253;294)	5.2 (4.7;5.4)	1144 (1058;1223)	4.6 (4.3;4.9)	-11.5 (-17.2;-4.6)
Pará	63 (59;71)	4.5 (4.2;5)	258 (232;279)	4.6 (4.1;5)	2.5 (-8.3;11.9)
Paraíba	74 (69;85)	3.8 (3.5;4.3)	196 (179;222)	3.9 (3.6;4.5)	4.1 (-8.4;16.3)
Paraná	123 (115;137)	5.4 (5.1;5.9)	516 (487;575)	4.9 (4.6;5.5)	-8.3 (-14.2;-2)
Pernambuco	135 (127;151)	4.7 (4.5;5.3)	402 (377;446)	4.3 (4;4.8)	-9 (-15.1;-2.3)
Piaui	43 (39;51)	4.1 (3.7;5)	138 (129;157)	3.8 (3.5;4.3)	-9.1 (-20.7;0.5)
Rio de Janeiro	290 (266;306)	5.3 (4.9;5.6)	1052 (972;1124)	5.2 (4.8;5.6)	-0.2 (-6.7;6.5)
Rio Grande do Norte	60 (54;67)	4.1 (3.7;4.6)	169 (158;188)	4.2 (3.9;4.6)	1.5 (-8.4;12.4)
Rio Grande do Sul	183 (168;195)	4.9 (4.5;5.2)	698 (655;761)	4.9 (4.6;5.3)	0.5 (-6.6;7.6)
Rondônia	5 (5;6)	4.5 (4.2;5)	45 (41;52)	4.3 (3.8;4.9)	-5.4 (-16.1;6.2)
Roraima	1 (1;2)	7.7 (7;9.2)	11 (10;13)	6.6 (5.8;7.4)	-14.5 (-30.2;-1.2)
São Paulo	637 (571;670)	5.8 (5.2;6.1)	2605 (2372;2755)	5.8 (5.3;6.2)	1.2 (-6.3;8.4)
Santa Catarina	69 (64;74)	5.2 (4.8;5.6)	312 (293;340)	5.1 (4.8;5.6)	-1.3 (-8.7;6.5)
Sergipe	27 (25;31)	3.6 (3.4;4.1)	81 (75;87)	4 (3.7;4.4)	11.4 (-0.8;21.6)
Tocantins	10 (9;12)	7.1 (6.4;8.3)	55 (50;60)	4.4 (4;4.8)	-37.4 (-46.5;-30.2)

* Fonte: Estudo Burden of Disease 2017, Institute for Health Metrics and Evaluation. ^285^*

**Tabela 6-3:** – Número de mortes e taxa de mortalidade padronizada por idade por fibrilação atrial e flutter, por 100 mil habitantes, para homens, em 1990 e 2017, com variação percentual, no Brasil e suas unidades federativas

	1990		2017		
	Número de mortes (II 95%)	Taxa de mortalidade (II 95%)	Número de mortes (II 95%)	Taxa de mortalidade (II 95%)	Variação percentual (II 95%)
Acre	2.4 (2;2.9)	5.2 (4.5;6.3)	9.9 (7.4;11)	4.3 (3.2;4.7)	-18.5 (-36;-6.3)
Alagoas	16.8 (14.9;21.8)	3.9 (3.5;5.1)	43.9 (36;51)	3.6 (3;4.2)	-6.8 (-25.1;7.4)
Amapá	1.4 (1.2;1.7)	5.3 (4.7;6.9)	8 (5.9;8.9)	5.7 (4.2;6.3)	7.2 (-22.4;24.4)
Amazonas	9 (8;12.5)	4.1 (3.7;5.9)	37.1 (31.5;44.3)	3.8 (3.2;4.5)	-8.9 (-31.4;6.1)
Bahia	106.6 (93.5;133.7)	4.5 (3.9;5.6)	300.3 (246.4;345.5)	4.5 (3.7;5.2)	2 (-12.8;14.6)
Brasil	1134.1 (952.8;1309.1)	4.7 (4.1;5.6)	3773.7 (3076.2;4312)	4.6 (3.8;5.2)	-3.5 (-12.9;1.8)
Ceará	60.8 (52.3;85.1)	3.7 (3.2;5.2)	150.3 (124.7;172.3)	3.6 (3;4.1)	-3.7 (-27.5;14.2)
Distrito Federal	5.1 (4.4;6.3)	5.8 (5;6.9)	33.1 (27.6;44.6)	6.5 (5.5;8.3)	12.6 (-5.3;28.7)
Espírito Santo	18.7 (15.5;21.5)	6 (5;7.1)	68.3 (59.6;85.4)	4.5 (3.9;5.6)	-25.5 (-35.5;-12.6)
Goiás	19.6 (15.7;22.2)	3.6 (2.8;4)	104.4 (86.7;121.5)	4.3 (3.6;5.1)	20.7 (5.6;36.8)
Maranhão	37.7 (28.4;47.6)	6 (4.1;7.9)	152.5 (121.6;180.5)	6.1 (4.8;7.2)	0.5 (-17.7;21.6)
Mato Grosso	9.7 (8.6;13.3)	4.5 (3.9;6.5)	45.7 (39.9;56.6)	4 (3.5;4.9)	-12.1 (-31.4;2)
Mato Grosso do Sul	12 (10;13.7)	4.8 (3.9;5.4)	46.7 (37.9;53)	4.5 (3.7;5.2)	-4.6 (-15;4.9)
Minas Gerais	117.9 (91.5;129.6)	4.9 (3.8;5.3)	448.6 (360.9;504.4)	4.4 (3.6;5)	-8.8 (-18.3;5.3)
Pará	27.4 (24.5;34.2)	4.4 (3.9;5.6)	118.4 (98.2;138.8)	4.7 (3.8;5.5)	6.5 (-11.5;21.6)
Paraíba	34.5 (30.2;43.6)	3.7 (3.2;4.6)	73.6 (58.5;86.4)	3.7 (3;4.4)	1.3 (-16.8;20)
Paraná	56.9 (48.1;67.9)	5.1 (4.3;6.1)	207 (179.8;248.3)	4.7 (4.1;5.6)	-7.7 (-19.6;2.2)
Pernambuco	60.6 (51.5;72.6)	5 (4.2;5.9)	146.5 (118.1;165.8)	4 (3.2;4.5)	-19.1 (-30.3;-10.8)
Piauí	21.7 (18.5;31.3)	5.2 (4.4;7.7)	51.5 (41.7;59.5)	3.3 (2.7;3.8)	-36.2 (-55.3;-22.8)
Rio de Janeiro	111.5 (86.2;124.2)	5.4 (4.3;6.1)	348.2 (286.2;400.2)	4.9 (4.1;5.7)	-9.5 (-20.4;2.3)
Rio Grande do Norte	28.1 (25.1;36.5)	4.4 (3.9;5.6)	64.6 (52.2;74.5)	4 (3.2;4.6)	-7.7 (-27.1;6.6)
Rio Grande do Sul	72.6 (59.1;83.3)	5.2 (4.2;6)	244.4 (205.8;288.7)	4.8 (4;5.6)	-8 (-20.4;2.1)
Rondônia	3.1 (2.7;3.7)	4.9 (4.1;5.7)	21.6 (18;25.5)	4 (3.3;4.7)	-18.7 (-32.2;-4.5)
Roraima	0.7 (0.6;0.9)	7.4 (6.4;10.2)	5.2 (3.5;6.1)	5.6 (3.7;6.6)	-24.1 (-55.7;-2.4)
Santa Catarina	29 (23.2;33.3)	4.9 (3.9;5.6)	111.5 (95.9;136.1)	4.6 (3.9;5.5)	-6.4 (-21.3;9.3)
São Paulo	253.3 (189.4;278.3)	5.4 (4.1;6)	877.3 (668.2;969.6)	5.2 (4;5.8)	-4.1 (-16.6;9.7)
Sergipe	12.1 (10.9;15.6)	3.8 (3.4;4.8)	29.6 (24;33.9)	3.7 (3;4.3)	-1.3 (-19.8;13.2)
Tocantins	5 (4.3;7)	6.7 (5.6;10.1)	25.5 (19.7;28.6)	4.1 (3.1;4.5)	-39.9 (-59.5;-25)

* Fonte: Estudo Global Burden of Disease 2017, Institute for Health Metrics and Evaluation. ^285^*

**Tabela 6-4:** – Número de mortes e taxa de mortalidade padronizada por idade por fibrilação atrial e *flutter* , por 100 mil habitantes, para mulheres, em 1990 e 2017, com variação percentual, no Brasil e suas unidades federativas

	1990		2017		
	Número de mortes (II 95%)	Taxa de mortalidade (II 95%)	Número de mortes (II 95%)	Taxa de mortalidade (II 95%)	Variação percentual (II 95%)
Acre	2.1 (1.9;2.2)	4.7 (4.3;5)	12.1 (11;13.8)	4.7 (4.3;5.4)	0.1 (-12.3;20.5)
Alagoas	18.6 (17.2;20.4)	3.3 (3;3.6)	74.9 (68.7;87)	4.4 (4;5.1)	33.7 (18.1;51.9)
Amapá	1.8 (1.6;1.9)	6.2 (5.7;6.6)	9.8 (8.9;10.9)	5 (4.6;5.6)	-19.1 (-28.5;-6.7)
Amazonas	9.5 (8.8;10.3)	3.8 (3.5;4)	50.3 (46.4;55.1)	4.2 (3.9;4.7)	13 (1.5;26.5)
Bahia	129.5 (119;141.4)	4.1 (3.8;4.5)	419 (386.8;470)	4.1 (3.8;4.6)	1 (-10.1;14.6)
Brasil	1515 (1453.7;1558)	4.7 (4.5;4.9)	6285.3 (6014.2;6615.7)	5 (4.8;5.2)	5.4 (0;12.3)
Ceará	67.3 (55.6;76.4)	3.4 (2.8;3.8)	253.8 (235.8;280.5)	4.1 (3.8;4.5)	21.2 (2.5;55.8)
Distrito Federal	7.9 (7.4;8.5)	6.8 (6.3;7.5)	64.3 (55.9;72)	8 (6.9;9)	17.8 (-4.1;36.2)
Espírito Santo	22.2 (21.1;23.4)	6.3 (5.9;6.6)	100.3 (91.8;109.2)	4.4 (4;4.8)	-29.8 (-36.4;-22.7)
Goiás	23.4 (21.9;24.8)	6.4 (6;6.8)	121.8 (112.8;132.6)	4.2 (3.9;4.6)	-34.1 (-39.9;-27.8)
Maranhão	47.5 (19.2;62)	4.6 (1.8;6)	152.1 (84.2;183)	4.5 (2.5;5.4)	-2.7 (-16.2;44.7)
Mato Grosso	7.9 (7.1;8.8)	3.8 (3.4;4.2)	53 (48.6;58.4)	4.7 (4.3;5.2)	23.7 (7;43.6)
Mato Grosso do Sul	12.3 (11.7;13)	5.4 (5.1;5.7)	63.5 (58.3;71.8)	5 (4.5;5.6)	-8.2 (-16.6;3.4)
Minas Gerais	161 (153.7;169.7)	5.3 (5.1;5.6)	695.6 (644.5;746.7)	4.7 (4.3;5)	-12.6 (-19.7;-5.3)
Pará	35.7 (33;38.6)	4.5 (4.2;4.8)	139.2 (126.4;151.5)	4.5 (4.1;4.9)	-0.5 (-10.9;10.8)
Paraíba	40 (36.6;44)	3.9 (3.5;4.2)	122.3 (108.1;146.1)	4.1 (3.6;4.8)	5.2 (-10.7;27.9)
Paraná	66 (62.6;70.3)	5.6 (5.3;6)	309.3 (287.2;338.3)	5 (4.7;5.5)	-10 (-16.8;-2)
Pernambuco	74.4 (69.4;82.4)	4.6 (4.3;5)	255.8 (234.3;293.1)	4.5 (4.1;5.1)	-2.1 (-10.7;9)
Piauí	21.2 (17.8;24.3)	3.5 (3;4.1)	86.9 (80.1;99)	4 (3.7;4.6)	14.2 (-2.7;46.6)
Rio de Janeiro	178.9 (169.9;190.3)	5.2 (4.9;5.5)	703.5 (652;753.2)	5.3 (5;5.7)	3.5 (-4.7;12.5)
Rio Grande do Norte	31.7 (27.6;35.2)	4 (3.4;4.4)	104.6 (95.4;119)	4.2 (3.8;4.8)	7.2 (-7.4;35.9)
Rio Grande do Sul	110 (104.6;115.1)	4.7 (4.5;5)	454 (421.1;488)	4.9 (4.6;5.3)	3.6 (-4.2;12)
Rondônia	2 (1.8;2.2)	4.1 (3.8;4.5)	23.7 (20.5;28.3)	4.5 (3.9;5.4)	9.7 (-6.2;28.3)
Roraima	0.5 (0.5;0.6)	7.8 (7.1;8.7)	6.1 (5.1;7.7)	7.3 (6.1;9.2)	-6.5 (-26.1;18.5)
Santa Catarina	39.8 (37.7;42.4)	5.4 (5.1;5.7)	200.6 (185.2;217.9)	5.4 (5;5.9)	0.7 (-8;10.2)
São Paulo	384 (366;404.7)	5.9 (5.6;6.2)	1728 (1597.3;1849.2)	6.1 (5.7;6.6)	3.3 (-4.6;11.7)
Sergipe	14.7 (13.6;16)	3.5 (3.2;3.8)	51 (46.9;55.6)	4.2 (3.9;4.6)	20 (5.5;34.7)
Tocantins	5 (4.2;5.6)	7.3 (6;8.4)	29.9 (27.3;33.6)	4.8 (4.4;5.4)	-34.3 (-44.6;-13.8)

* Fonte: Estudo Global Burden of Disease 2017, Institute for Health Metrics and Evaluation. ^285^*

**Tabela 6-5:** – Número de DALYs e taxas de DALYs padronizadas por idade por fibrilação atrial e *flutter* , por 100 mil habitantes, em 1990 e 2017, com variação percentual, no Brasil e suas unidades federativas

	1990		2017		
	Número de DALYs (II 95%)	Taxa de DALYs (II 95%)	Número de DALYs (II 95%)	Taxa de DALYs (II 95%)	Variação percentual (II 95%)
Acre	140.3 (113.7;174.5)	95.2 (79;115.3)	542.9 (445.2;660.8)	99.2 (82.4;119.4)	4.2 (-2.5;9.6)
Alagoas	909.3 (730.5;1123.5)	72.4 (59.1;88.5)	2456 (2031.2;2952.7)	82.7 (68.8;99.8)	14.3 (5.6;21.9)
Amapá	93.7 (76;114.6)	108.7 (91.2;129.2)	499.4 (411.6;598.4)	114.8 (96.3;135.5)	5.6 (-0.8;10.9)
Amazonas	560.7 (445.6;698.8)	80.3 (65.3;98.1)	2138.5 (1735.6;2644.7)	86 (70.9;104.7)	7 (-2.3;13.8)
Bahia	5285.8 (4366.4;6397.1)	81.3 (67.7;97.7)	13791.8 (11451.4;16610.3)	87.7 (72.8;105.1)	7.9 (1.8;14.3)
Brasil	79208.5 (64514;96525.8)	99 (82.3;118.9)	226809.7 (187976.8;272166.3)	103.6 (86.3;123.8)	4.6 (1.7;7.2)
Ceará	3014.4 (2422;3741.1)	75.8 (61.3;93.5)	8054.1 (6619.6;9756.6)	81.9 (67.2;99.4)	8.1 (0.8;15.1)
Distrito Federal	543.8 (441.9;662.7)	115.2 (98.8;135.3)	2469.3 (2043.2;2968.8)	121.7 (104.3;143.6)	5.6 (-0.4;12.4)
Espírito Santo	1099.3 (895.3;1344.2)	92 (77.3;109.3)	3570.3 (2938.2;4327.6)	88 (73;106.3)	-4.3 (-9.7;2)
Goiás	1394.8 (1125.9;1736.9)	88.5 (74.3;106.4)	5412.9 (4410.7;6618)	87.9 (72.8;106.9)	-0.7 (-6;4.2)
Maranhão	1889.2 (1401.3;2435)	82.3 (59.9;105.9)	5041.4 (3929.4;6224.5)	82.1 (63.9;101)	-0.3 (-8.1;9.3)
Mato Grosso	598.8 (479.5;750)	88.1 (72.5;107.8)	2612.4 (2125.1;3202.6)	93.2 (76.9;113.4)	5.7 (-1.8;12.3)
Mato Grosso do Sul	826.2 (672.8;1002.9)	106.5 (89.4;126.4)	2822.2 (2306.6;3401.7)	107.2 (88.5;128.2)	0.7 (-4.2;5.9)
Minas Gerais	9413 (7638.3;11547.3)	108.3 (90.1;129.8)	27059.3 (22340.6;32657.7)	108.2 (89.5;130.4)	-0.1 (-5;4.7)
Pará	1539.4 (1262.2;1889.2)	81.5 (67.9;98.9)	5393.3 (4478.1;6529.9)	87.8 (73.5;105.3)	7.7 (-0.5;15.3)
Paraíba	1821.5 (1468.3;2223.4)	80.9 (65.7;98.4)	4007.5 (3263.1;4821.6)	86.5 (70.3;104.3)	6.9 (-0.9;14.3)
Paraná	3979.9 (3220;4841.8)	99.3 (83.5;118.5)	12001.8 (9814.5;14534.6)	99.9 (82.5;120)	0.6 (-4.5;5.2)
Pernambuco	3220.7 (2629.5;3992.3)	80.7 (67.7;97.7)	7977.1 (6643;9724)	83.5 (69.8;101.6)	3.5 (-2.1;8.8)
Piauí	1028.8 (830.3;1272.4)	78.1 (63.8;95.4)	2789.9 (2291.5;3412.7)	77.8 (64;95)	-0.4 (-9.6;6.5)
Rio de Janeiro	9520.4 (7787.9;11658.2)	111.4 (93.3;133.4)	24316.8 (20202.2;29195.3)	115 (95.9;137.6)	3.2 (-1.1;7.7)
Rio Grande do Norte	1263.4 (1031.4;1535.4)	80.1 (65.7;96.9)	3165.6 (2620.9;3796.6)	84.7 (70.1;101.5)	5.8 (-1;13)
Rio Grande do Sul	5078.4 (4134.3;6186.3)	89.5 (74.8;107.2)	13838.5 (11505.8;16723.4)	93.9 (78.3;113)	5 (-0.4;10.1)
Rondônia	253.4 (200.9;317.6)	90.3 (75.1;109.6)	1179 (948.7;1453.8)	89.7 (73.1;108.8)	-0.7 (-9.2;6.7)
Roraima	56.1 (44.6;69.3)	126.8 (106.6;149.8)	348.9 (280.1;430.2)	121.6 (100.7;145.4)	-4.1 (-14.3;4.4)
Santa Catarina	2181.6 (1779.3;2657.9)	102 (85.4;121.3)	7392.7 (6024.1;8973)	103.4 (85.4;123.9)	1.3 (-4.1;6.7)
São Paulo	22477.6 (18132.5;27717.9)	126.9 (105.3;152.6)	64766.7 (53494.2;78258.7)	129.7 (107.8;155.7)	2.2 (-1.9;6.8)
Sergipe	669 (542.7;814.8)	81.1 (66.1;98.5)	1828.2 (1484.9;2230)	88.9 (72.8;108.1)	9.6 (2;16.3)
Tocantins	349.1 (280.8;431.7)	109.5 (92.1;130.9)	1333.2 (1097.2;1609.2)	100.5 (83.2;120.9)	-8.2 (-17.2;-1.2)

* Fonte: Estudo Global Burden of Disease 2017, Institute for Health Metrics and Evaluation. ^285^*

**Tabela 6-6:** – Número de DALYs e taxas de DALYs padronizadas por idade por fibrilação atrial e *flutter* , por 100 mil habitantes, para homens, em 1990 e 2017, com variação percentual, no Brasil e suas unidades federativas

	1990		2017		
	Número de DALYs (II 95%)	Taxa de DALYs (II 95%)	Número de DALYs (II 95%)	Taxa de DALYs (II 95%)	Variação percentual (II 95%)
Acre	82.4 (64.8;104.4)	103.4 (83.4;127.4)	285.5 (225.7;358.6)	105.6 (83.6;130.8)	2.1 (-9.8;11)
Alagoas	499.5 (389.5;638.7)	85 (67.3;107.9)	1190.5 (947.6;1474.1)	90.7 (72.7;112.3)	6.7 (-5.5;16.7)
Amapá	51.1 (40;63.7)	118 (95.2;144.5)	271.4 (214;333.8)	130.5 (104.5;157)	10.6 (-3;20.7)
Amazonas	329.6 (252.8;421.6)	94 (74.1;118)	1188.9 (930.8;1511.3)	97.5 (77.3;121.8)	3.7 (-9.4;13)
Bahia	2825.4 (2259;3508.4)	93.6 (75.8;114.9)	7090.6 (5714.9;8706.2)	103.8 (83.9;126.9)	10.8 (0.8;20.6)
Brasil	41514.9 (32715.2;51721.2)	111.1 (89.5;136.9)	110643.1 (88439.8;136579.6)	115.1 (92.9;141.4)	3.6 (-1.4;6.8)
Ceará	1685.5 (1306.6;2141)	90 (70.3;113.6)	4062.6 (3213.5;5079.7)	94.3 (74.8;117.5)	4.8 (-9.7;15.1)
Distrito Federal	279.4 (219.3;350.7)	121.6 (100;148)	1168.9 (907.8;1460.2)	129.5 (104.1;159.2)	6.5 (-3.6;16.9)
Espírito Santo	599.5 (468.5;752.8)	102.1 (82.7;126.1)	1800.5 (1424.3;2230.4)	99.9 (79.9;123)	-2.1 (-9.8;6.8)
Goiás	751.4 (581.6;960.7)	85.2 (67.9;107.5)	2773.6 (2210.3;3516.4)	95.5 (77.1;119.9)	12.1 (3.3;20.3)
Maranhão	1002.8 (794.7;1292)	97.2 (76.4;124.3)	2672.9 (2147.4;3307.8)	95.8 (77.1;117.9)	-1.4 (-12.1;9.3)
Mato Grosso	361.1 (280.9;464.2)	97.1 (77.2;122.4)	1416.9 (1121.4;1780.2)	98 (78;122.3)	0.9 (-10.9;10.3)
Mato Grosso do Sul	481.8 (382.6;600.1)	116.5 (94.2;143.5)	1485.7 (1183.7;1835.7)	118.9 (95.7;145.8)	2 (-5;8.3)
Minas Gerais	5075.7 (3941.5;6388.2)	123.2 (98.4;152.2)	13931.2 (11083.4;17144.6)	124.9 (100.2;152.9)	1.4 (-5.6;8.7)
Pará	848 (666.8;1072.3)	92 (73.9;114.8)	3030.6 (2434.6;3752.2)	102.1 (83.5;125.9)	11 (-0.9;21.2)
Paraíba	959.6 (751.8;1199.7)	89.6 (70.4;112.2)	1908.7 (1508.1;2391.4)	96.4 (76.2;120.1)	7.6 (-3.9;19.3)
Paraná	2155.1 (1663;2723.3)	106.1 (85.2;131)	5923.6 (4690.3;7392.9)	109.3 (87.5;134.3)	3 (-4.7;10.2)
Pernambuco	1666.7 (1313.2;2134)	92.1 (74.4;115.9)	3745.8 (2945.1;4730.6)	92.8 (73.4;117.1)	0.8 (-8.9;8.4)
Piauí	576.9 (446.6;737.6)	95.2 (74.8;122.4)	1384.7 (1098.5;1744.7)	85.8 (68.1;107.6)	-9.9 (-26.6;0.8)
Rio de Janeiro	4817.5 (3787.4;6124)	130.7 (106;161.9)	11413.5 (9010.4;14162.3)	132.1 (105.6;162.3)	1.1 (-6.1;7.9)
Rio Grande do Norte	693 (547.8;864.6)	93.1 (74.1;115.7)	1566.9 (1250.7;1936.6)	97.1 (77.6;119.6)	4.3 (-9.9;14.5)
Rio Grande do Sul	2490.2 (1952.9;3132)	100.7 (81.3;123.8)	6341.6 (5040.4;7933.5)	103.5 (83.6;128.3)	2.8 (-6;10)
Rondônia	162 (126.2;207.2)	99.7 (81.4;123.1)	641.9 (500.4;812.1)	94.9 (75.2;118.3)	-4.9 (-14.9;5)
Roraima	37.3 (29;46.7)	140.1 (113.5;170.8)	206.6 (161.2;261.9)	132.4 (105.1;163.7)	-5.5 (-24.5;7.6)
Santa Catarina	1083.7 (847.3;1362.7)	106.2 (85.8;130.2)	3431.7 (2674.2;4328.4)	107 (85.2;133.9)	0.8 (-8.3;9.9)
São Paulo	11426.4 (8857.6;14374.4)	139.5 (111.1;173.2)	30035.6 (23857;37379.4)	140.2 (112.5;172.2)	0.5 (-6.5;7.5)
Sergipe	360.2 (280.2;450.3)	95.4 (74.6;119)	913.8 (721.5;1145.2)	101.8 (81.3;126.9)	6.7 (-4.6;16.1)
Tocantins	213.1 (162.9;269.7)	120.8 (96.4;150)	758.9 (602.2;940.5)	111.6 (89.1;137.6)	-7.6 (-24.8;3.3)

* Fonte: Estudo Global Burden of Disease 2017, Institute for Health Metrics and Evaluation. ^285^*

**Tabela 6-7:** – Número de DALYs e taxas de DALYs padronizadas por idade por fibrilação atrial e *flutter* , por 100 mil habitantes, para mulheres, em 1990 e 2017, com variação percentual, no Brasil e suas unidades federativas

	1990		2017		
	Número de DALYs (II 95%)	Taxa de DALYs (II 95%)	Número de DALYs (II 95%)	Taxa de DALYs (II 95%)	Variação percentual (II 95%)
Acre	57.9 (47.4;70.5)	86.1 (72.7;102.9)	257.4 (215.1;306.5)	92.9 (77.9;110.2)	7.9 (-0.3;18.8)
Alagoas	409.8 (338.1;497)	61.8 (51.4;75)	1265.4 (1065.7;1512.4)	76 (64.2;90.7)	23 (11;37.8)
Amapá	42.6 (35.6;51.1)	99.4 (85;116.6)	227.9 (189.3;270.1)	101.1 (84.9;118.6)	1.6 (-6.7;11.1)
Amazonas	231.1 (189.1;286.3)	67.1 (56.5;81.1)	949.6 (793.8;1139.7)	74.8 (62.7;88.8)	11.4 (2.8;20.8)
Bahia	2460.4 (2058.7;2986.8)	70.7 (59.5;85.1)	6701.3 (5667.8;8010.2)	74.7 (62.9;89.5)	5.6 (-2.8;15)
Brasil	37693.5 (31770.2;45054.9)	88.6 (76.2;103.9)	116166.6 (98895.8;135791.7)	93.7 (79.8;109.5)	5.8 (2.4;9.7)
Ceará	1329 (1065.5;1653.3)	63.3 (51;78.8)	3991.4 (3369.5;4733.9)	71.4 (60.1;85.2)	12.8 (0.7;31.8)
Distrito Federal	264.4 (221.2;315.2)	109.1 (94.7;125)	1300.4 (1089.6;1554.4)	113.9 (96.7;132.4)	4.4 (-7.5;15.1)
Espírito Santo	499.9 (418.4;598.1)	82.4 (71.3;96.2)	1769.9 (1488;2115)	78 (65.7;93)	-5.3 (-11.9;1.5)
Goiás	643.3 (529.2;783.9)	92.3 (80.3;107)	2639.3 (2200.5;3167.7)	81.1 (68.2;96.8)	-12.2 (-18.5;-5.6)
Maranhão	886.4 (483.4;1176.4)	72.3 (38.6;96)	2368.5 (1549.5;2987.1)	71.1 (46.4;89.5)	-1.8 (-12.9;25)
Mato Grosso	237.7 (192.1;291.2)	78.2 (64.8;94.1)	1195.4 (994.5;1433.5)	88.1 (74.1;103.9)	12.7 (3.3;24)
Mato Grosso do Sul	344.4 (290.2;410.1)	95.3 (82.6;111.1)	1336.5 (1121.2;1582.5)	96.4 (81.4;113.9)	1.2 (-5.8;9.8)
Minas Gerais	4337.2 (3603.5;5206.8)	95 (81.1;111.6)	13128.1 (11052.7;15567.4)	93.7 (78.7;111.3)	-1.3 (-7.3;4.6)
Pará	691.4 (579.5;847.2)	71.2 (60.2;85.5)	2362.7 (2004;2820.5)	74.2 (63.4;87.9)	4.2 (-4.6;13.7)
Paraíba	861.9 (711.9;1040.7)	73.2 (61.1;87.8)	2098.8 (1736.5;2506.6)	78.4 (64.5;94)	7.1 (-4;21.5)
Paraná	1824.7 (1515.5;2188.5)	92.4 (79.6;107.7)	6078.2 (5072;7195.1)	91.6 (76.9;107.9)	-0.9 (-6.8;5.3)
Pernambuco	1554 (1305.2;1877.1)	71.4 (61;84.8)	4231.3 (3587.3;5077.2)	76.1 (64.6;91)	6.6 (-0.4;14.6)
Piauí	451.9 (362.3;565.1)	64.6 (52.1;80.5)	1405.3 (1184.9;1674.4)	70.6 (59.4;84.4)	9.2 (-2.9;28)
Rio de Janeiro	4703 (3930;5630.4)	97 (82.5;114.7)	12903.3 (10976.9;15227.6)	101.6 (86.2;120)	4.7 (-1.2;11)
Rio Grande do Norte	570.4 (472.9;691.2)	68.7 (57;82.9)	1598.7 (1346;1893.9)	74.5 (62.2;88.8)	8.4 (-3.2;27.5)
Rio Grande do Sul	2588.2 (2156.8;3097.6)	81.1 (69.1;95.9)	7496.9 (6386.8;8904.7)	86 (72.8;102.1)	5.9 (0;12.8)
Rondônia	91.4 (72.4;113.5)	79.2 (66.7;94.3)	537.1 (434.2;655.1)	84.2 (68.9;101.2)	6.3 (-4.1;18.8)
Roraima	18.8 (15.1;22.9)	108.9 (92.8;126.8)	142.3 (114.4;173.6)	108.9 (89.6;130.4)	0 (-14.6;17.9)
Santa Catarina	1097.9 (912.9;1328.2)	97.8 (83.5;116.1)	3961 (3331.6;4682.9)	99.3 (83.9;116.5)	1.5 (-4.6;8.4)
São Paulo	11051.2 (9167;13335.7)	115.9 (98;137.3)	34731.2 (29277.5;41069.7)	120.4 (101.6;142.3)	3.9 (-1.4;9.9)
Sergipe	308.8 (258.3;371.5)	69 (58;82.6)	914.3 (770.8;1097.2)	78.3 (65.9;93.7)	13.3 (4.5;23.3)
Tocantins	136 (110.1;166.5)	96.4 (80.4;114.7)	574.3 (479.9;679.9)	89 (74.9;105.6)	-7.7 (-18.2;8.4)

* Fonte: Estudo Global Burden of Disease 2017, Institute for Health Metrics and Evaluation. ^285^*

**Figura 6-1:**
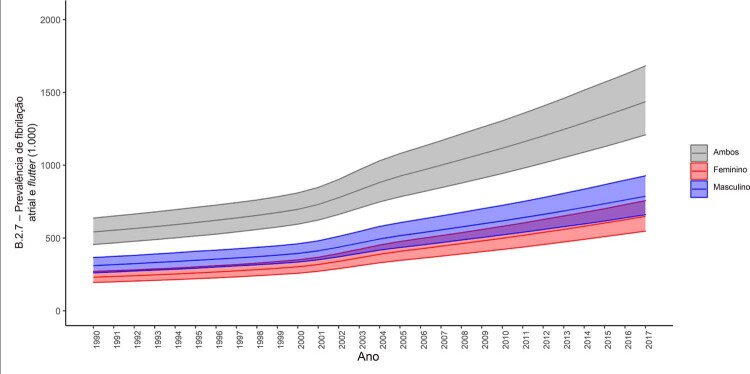
- Número de casos prevalentes de fibrilação atrial e flutter entre 1990 e 2017, por sexo no Brasil.

Dados derivados do Estudo Global Burden of Disease 2017 (GBD 2017). ^285^

**Figura 6-2:**
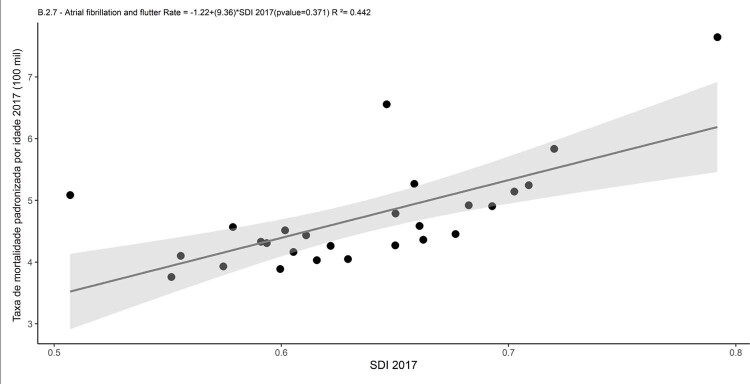
- Correlação entre o Índice Sociodemográfico (SDI) e a taxa de mortalidade padronizada por idade por fibrilação atrial e flutter, por 100 mil habitantes.

Dados derivados do Estudo Global Burden of Disease 2017 (GBD 2017). ^285^

**Figura 6-3:**
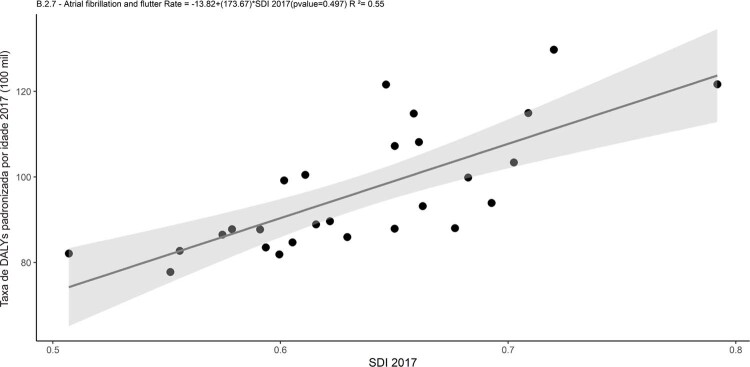
- Correlação entre o Índice Sociodemográfico (SDI) e a taxa de DALYs padronizada por idade por fibrilação atrial e flutter, por 100 mil habitantes.

Dados derivados do Estudo Global Burden of Disease 2017 (GBD 2017). ^285^
