# Supplementary material for: Estatística Cardiovascular – Brasil 2020
Source: Arq Bras Cardiol. 2020 Sep 18;115(3):308–439. [Article in Portuguese] doi: 10.36660/abc.20200812 (PMC9363085; doi:10.36660/abc.20200812)
Supplement: Translation

# Translation: Cardiovascular Statistics – Brazil 2020

**Authors:** Gláucia Maria Moraes de Oliveira, Luisa Campos Caldeira Brant, Carisi Anne Polanczyk, Andreia Biolo, Bruno Ramos Nascimento, Deborah Carvalho Malta, Maria de Fatima Marinho de Souza, Gabriel Porto Soares, Gesner Francisco Xavier, M. Julia Machline-Carrion, Marcio Sommer Bittencourt, Octavio M. Pontes, Odilson Marcos Silvestre, Renato Azeredo Teixeira, Roney Orismar Sampaio, Thomaz A. Gaziano, Gregory A. Roth, Antonio Luiz Pinho Ribeiro

**Affiliations:** 1 Brazil Instituto do Coração Edson Saad da Universidade Federal do Rio de Janeiro (UFRJ), Rio de Janeiro, RJ – Brazil; 2 Brazil Disciplina de Cardiologia, Departamento de Clínica Médica da Faculdade de Medicina da Universidade Federal do Rio de Janeiro (UFRJ), Rio de Janeiro, RJ – Brazil; 3 Brazil Departamento de Clínica Médica da Faculdade de Medicina da Universidade Federal de Minas Gerais (UFMG), Belo Horizonte, MG – Brazil; 4 Brazil Serviço de Cardiologia e Cirurgia Cardiovascular e Centro de Telessaúde do Hospital das Clínicas da Universidade Federal de Minas Gerais (UFMG), Belo Horizonte, MG – Brazil; 5 Brazil Faculdade de Medicina da Universidade Federal do Rio Grande do Sul (UFRS), Porto Alegre, RS – Brazil; 6 Brazil Serviço de Cardiologia do Hospital Moinhos de Vento, Porto Alegre, RS – Brazil; 7 Brazil Hospital de Clínicas de Porto Alegre (HCPA), Porto Alegre, RS – Brazil; 8 Brazil Programa de Pós-Graduação em Saúde Pública da Universidade Federal de Minas Gerais (UFMG), Belo Horizonte, MG – Brazil; 9 USA Organização Vital Strategies, Nova York – USA; 10 Brazil Curso de Medicina da Universidade de Vassouras, Vassouras, RJ – Brazil; 11 Brazil Biblioteca da Faculdade de Medicina Universidade Federal de Minas Gerais (UFMG), Belo Horizonte, MG – Brazil; 12 Brazil ePHealth Primary Care Solutions, Santo Antônio, SC – Brazil; 13 Brazil Divisão de Clínica Médica do Hospital Universitário da Universidade de São Paulo, São Paulo, SP – Brazil; 14 Brazil Faculdade Israelita de Ciências da Saúde Albert Einstein, São Paulo, SP – Brazil; 15 Brazil Serviço de Neurologia Vascular e Emergências Neurológicas, Divisão de Neurologia, Departamento de Neurociências e Ciências do Comportamento, Faculdade de Medicina de Ribeirão Preto da Universidade de São Paulo (USP), São Paulo, SP – Brazil; 16 Brazil Universidade Federal do Acre (UFAC), Rio Branco, AC – Brazil; 17 Brazil Departamento de Cardiopneumologia da Faculdade de Medicina da Universidade de São Paulo (USP), São Paulo, SP – Brazil; 18 Brazil Programa de Pós-Graduação da Faculdade de Medicina da Universidade de São Paulo (USP), São Paulo, SP – Brazil; 19 Brazil Unidade Clínica de Cardiopatias Valvares do Instituto do Coração (Incor) do Hospital das Clínicas da Faculdade de Medicina da Universidade de São Paulo (HCFMUSP), São Paulo, SP – Brazil; 20 USA Brigham and Women’s Hospital, Boston – USA; 21 USA Department of Medicine, Cardiovascular, Harvard Medical School, Boston – USA; 22 USA Global Health and Health Metrics Sciences at the Institute for Health Metrics and Evaluation (IHME), Washington – USA; 23 USA Division of Cardiology at the University of Washington School of Medicine, Washington – USA

**Keywords:** Cardiovascular Diseases, Coronary Disease, Cardiomyopathies. Heart Failure, Heart Valve Diseases, Atrial Fibrillation, Atrial Flutter, Statistics, Brazil

## About these Statistics

**Table:** 

Abbreviations Used in this Introduction	
CVD	Cardiovascular Disease
DALY	Disability-Adjusted Life Year
FU	Federative Unit
GBD	Global Burden of Disease
GDP	Gross Domestic Product
IBGE	Brazilian Institute of Geography and Statistics (in Portuguese, *Instituto Brasileiro de Geografia e Estatística* )
ICD	International Statistical Classification of Diseases and Related Health Problems
IHME	Institute for Health Metrics and Evaluation
Int$	International dollars
IPCA	Brazilian Consumer Price Index (in Portuguese, *Índice de Preços ao Consumidor Amplo* )
PNS	National Health Survey (in Portuguese, *Pesquisa Nacional de Saúde* )
PPP	Purchasing Power Parity
R$	Reais, Brazilian currency
SBC	Brazilian Society of Cardiology (in Portuguese, *Sociedade Brasileira de Cardiologia* )
SIH	Brazilian Hospital Information System (in Portuguese, *Sistema de Informações Hospitalares* )
SIM	Brazilian Mortality Information System (in Portuguese, *Sistema de Informações sobre Mortalidade* )
SUS	Brazilian Unified Health System (in Portuguese, *Sistema Único de Saúde* )
US$	US dollars
YLL	Year of Life Lost
YLD	Year Lived with Disability

Brazil is a continental country of extreme diversity regarding climate and environmental conditions, population density, economic development, and racial and cultural features. One of the 10 richest economies of the world, it is also one of the most unequal countries: according to the World Bank, Brazil ranks among the 10 countries with the highest Gini index, a measurement of inequality in income distribution ( https://data.worldbank.org/indicator/SI.POV.GINI ). Brazil has one of the largest public health systems with universal coverage, the SUS, which covers the whole Brazilian population, estimated at 210 million inhabitants in 2019 ( https://www.ibge.gov.br/estatisticas/sociais/populacao.html ), the year in which SUS was the exclusive health system for 76% of that population ( http://www.ans.gov.br ). Indeed, SUS co-exists with a private health system that includes health plans and insurance, and private health professionals. Established by the Brazilian Constitution of 1988, the implementation and expansion of SUS have allowed Brazil to rapidly address the changing health needs of the population, with dramatic upscaling of health services coverage and increase in life expectancy in just 3 decades.^1 , 2^The Family Health Program, launched in 1994, is a leading initiative in the national strategy to reduce CVD mortality based on primary health care, covering almost 123 million individuals (63% of the Brazilian population) in 2015.^3^However, despite its successes, the analysis of future scenarios suggests the urgent need to address lingering geographical inequalities, insufficient funding, and issues related to access to care and its quality.^1 , 2^

Cardiovascular disease has been the leading cause of mortality since the 1960s and has accounted for a substantial burden of disease in Brazil.^2 , 4^Considerable data relevant to cardiovascular health are now available from government health surveillance and administrative databases, as well as from epidemiological studies.^5 - 10^However, representative and reliable nationwide data on many health behaviors and cardiovascular risk factors, and on morbidity assessed in both the public and the private sectors remain sparse.^2^In recent years, the GBD project, led by the IHME of the University of Washington, began working with a Brazilian GBD network to release subnational estimates of the burden of disease by the Brazilian FUs, including for cardiovascular causes.^11 - 14^

This report, the **Cardiovascular Statistics – Brazil 2020** , incorporates official statistics provided by the Brazilian Ministry of Health and other government agencies, as well as data generated by other sources and scientific studies on heart disease, stroke, and other CVDs, including data from GBD/IHME. The aim of this project is to continuously monitor and evaluate data sources on heart disease and stroke in Brazil, to provide the most current information on the epidemiology of heart diseases and stroke to the Brazilian society, on an annual basis. This initiative is based on the American Heart Association **Heart Disease & Stroke Statistics Update** ^15^methodology, and is supported by the SBC, the GBD Brazil network, and an International Committee. The **Cardiovascular Statistics – Brazil 2020** document is the product of effort of dedicated volunteer clinicians and scientists, committed government professionals, and outstanding SBC members, without whom publication of this valuable resource would be impossible. The document was designed to be a valuable resource for researchers, clinicians, patients, healthcare policy makers, media professionals, the general public, and others who seek comprehensive national data on heart disease and stroke. The first edition was restricted to a limited number of clinical conditions, listed below:

1. Total Cardiovascular Diseases
2. Cerebrovascular Disease
3. Coronary Heart Disease, Acute Coronary Syndrome, and Angina Pectoris
4. Cardiomyopathy and Heart Failure
5. Valvular Diseases including Rheumatic Heart Disease
6. Atrial Fibrillation

All chapters are standardized in a common structure and included at least the following topics: Prevalence, Incidence, Mortality, Burden of Disease, Healthcare Utilization and Costs, Future Research. In the following editions, we intend to cover the clinical cardiac conditions more comprehensively, as well as the cardiovascular risk factors, life habits, quality of care, and other aspects that are relevant to the study of CVDs.

The emphasis of this document is on updated epidemiological data. It neither focuses on pathophysiological mechanisms or the merits of specific clinical treatments nor makes treatment recommendations. In addition, it is neither a position paper nor a comprehensive review, but tries to present the newest and best health-related metrics of CVD statistics for the Brazilian population. Moreover, it is not intended to cover other countries and regions, being restricted to Brazil, its regions, and FUs.

For the present document, we mostly used 3 sources of data: (a) the Brazilian mortality and health information systems, provided by the government; (b) GBD 2017 estimates; (c) systematic review of the literature with emphasis on what was published in the last 10 years. The metrics from the different sources were not identical and differences may be related to different time periods, location, age range or other methodological aspects (Malta, 2020, ABC Cardiol, in press). As such, we did not avoid citing discordant metrics, but possible reasons for these differences were generally mentioned or discussed. Since many studies cover a long period of time and life expectancy increased in Brazil in the last decades, we decided to use age-standardized rates, i.e., a weighted average of the age-specific rates per 100 000 persons, where the weights are the proportions of persons in the corresponding age groups of a standard population. The GBD age-standardization uses a global age pattern, although other sources may have used different reference populations. For most studies, race/skin color was used according to the IBGE definition, i.e., white, black, brown, yellow (oriental) or Indian (native American).

Herein, we present a summary of our data sources and the methodology used to assess healthcare utilization.

## Brazilian Mortality and Health Information Systems

For the present version of the **Cardiovascular Statistics – Brazil 2020** document, the major Brazilian data sources were the Brazilian Health Information Systems, comprising the Brazilian Mortality Information System and the Brazilian Hospital Information System, periodic health surveys, such as the National Health Survey, and official population estimates.

- Brazilian Mortality Information System (SIM): In Brazil, the SIM, created in 1975 by the Brazilian Ministry of Health, is responsible for collecting, storing, managing, and disseminating national mortality data. This health information system represented a major advance in the country’s epidemiological surveillance, since its main task is to record all deaths occurring in the Brazilian territory. The Ministry of Health implemented a Standard Certificate of Death model, a document for collecting information on death that uses the ICD to code the causes of death; in addition, a flow of collection, processing, and distribution of death information was implemented in all 5570 municipalities across the country.^16 , 17^The quality of the statistics on causes of death in Brazil significantly improved in the last 2 decades, but data from the beginning of the 2000 decade are still of low quality, specifically in some parts of the country.^18^Knowing the heterogeneity of these indicators in Brazil, the **Cardiovascular Statistics – Brazil 2020** report treated data to estimate information closer to real, by correcting for underreporting and redistribution of ill-defined causes of death. More details can be found in the article by Malta et al. (In press).
- Brazilian Hospital Information System (SIH): The aim of the SIH database is to register all hospitalizations funded by the SUS. The SIH-SUS compiles the hospitalizations at the municipal level through the ‘Hospital Admission Authorization’, which has information about the diseases leading to hospitalization (using ICD-10), length of stay, procedures, and costs.^19^The SIH-SUS information allows the development of methodologies and the definition of indicators to identify geographical disparities related to hospital resources.^20^
- National Health Survey (PNS): Although it was not in the scope of this year’s document to describe the statistics for cardiovascular risk factors, some chapters cite the metrics for some risk factors in the context of the specified disease. In such cases, a preference for the PNS was made. The PNS is a household-based epidemiological survey, representative of Brazil, its large regions, FUs, metropolitan regions, capitals, and other municipalities in each FU. The PNS 2013 sample was composed of 64 348 households. The survey was carried out by IBGE in partnership with the Ministry of Health. Most health topics were included, such as noncommunicable diseases, renal function, elderly, women, children, use of health services, health inequalities, anthropometric features, laboratory tests, and blood pressure measurements.^21^The PNS data are used by the GBD in its estimates for Brazil.
- For population estimates, the most updated population estimates generated by the IBGE (www.ibge.gov.br) were used in the denominator. For the hospitalizations and cost analyses, the resident population estimated for the National Audit Office yearly, from 2008 to 2018, was used.

## GBD 2017

The GBD Study (http://www.healthdata.org/gbd) is the most comprehensive worldwide observational epidemiological study to date. It describes mortality and morbidity from major diseases, injuries, and risk factors at global, national, and regional levels. Examining trends from 1990 to the present and making comparisons across populations enable the understanding of the changing health challenges faced by people across the world in the 21st century. The GBD 2017 is the latest publicly available dataset.^22 - 25^The GBD Brazil network has been collaborating with the IHME, from the University of Washington, that leads the project in the world, in the identification and provision of datasets, revision of models and estimates, validation and publication of the results for Brazil.^13 , 14^Details on how the estimates are calculated can be obtained in the capstone papers of the GBD Study^22 - 25^and in the IHME website (http://www.healthdata.org/acting-data/what-we-measure-and-why). We summarize below the main estimates used in this document:

- Estimates of deaths and causes of deaths. The main source of information is the SIM, a database from the Brazilian Ministry of Health, adjusted to other national and international sources. The IHME used methods for correcting for underreporting of deaths and “garbage code” deaths according to previously published algorithms,^26^updated in the newer versions of the study ( http://www.healthdata.org/acting-data/determining-causes-death-how-we-reclassify-miscoded-deaths ).
- YLLs are years lost due to premature mortality. The YLLs are calculated by subtracting the age at death from the longest possible life expectancy for a person at that age. For example, if the longest life expectancy for men in a given country is 75 years, but a man dies of cancer at 65, this would be 10 years of life lost due to cancer.
- YLDs, years lived with disability, can also be described as years lived in less than ideal health. This includes conditions such as influenza, which may last for only a few days, or epilepsy, which can last a lifetime. It is measured by taking the prevalence of the condition multiplied by the disability weight for that condition. Disability weights reflect the severity of different conditions and are developed through surveys of the general public.
- DALY is a universal metric that allows researchers and policymakers to compare quite different populations and health conditions across time. The DALYs equal the sum of YLLs and YLDs. One DALY equals one lost year of healthy life. DALYs allow us to estimate the total number of years lost due to specific causes and risk factors at the country, regional, and global levels.

## Systematic Review of the Literature

Descriptors for the elaboration of search strategies were selected in MeSH and DeCS, the controlled vocabularies from MEDLINE and LILACS, respectively. Embase’s plan was designed with Emtree descriptors associated with MeSH. Free terms were also used, that is, significant keywords and their synonyms, spelling variations, and acronyms that are essential for searching in the searched domain, but are not controlled descriptors (or are not in the synonym list of these descriptors). Importantly, to maintain search uniformity, the same descriptors were used in all search strategies. However, search strategies were customized according to the specifics of each database. It is also noteworthy that the group of terms related to “Brazil” was generally searched in all fields of research (subject, author, title, institutional affiliation, journal name, etc.).

The selected bases for research were MEDLINE via PubMed, Embase, LILACS, CINAHL, Cochrane Library Scopus, and Web of Science. The following bibliographic research filters and limits were used: period of publication (2004-2019); languages: Portuguese, English and Spanish; type of study/publication: Review, Meta-Analysis, Clinical Trial, Randomized Controlled Trial, Comparative Study, Practice Guideline, Guideline, Systematic Review, Evaluation Studies, Government Publications, and Multicenter Study. All references were managed using EndNote Web. From the search, articles were included if the studies were population- or community-based; nation- or state-wide studies were preferred. Moreover, articles set at health services or hospitals were included if the study was multicenter and had an adequate sample size (> 200 participants was the suggested cut-off), preferably. In addition to the articles identified by the systematic search, authors could include other studies found in the references of the searched articles or other articles they were aware of in their area of expertise, if the studies fulfilled the criteria above mentioned. Finally, which studies should be described in each chapter was mostly a decision of the experts commissioned to the specific theme.

## Healthcare Utilization

Healthcare costing studies have expressive methodologic variability and, thus, need to be carefully interpreted. In the present document, most of the cost data were gathered from reimbursement tables from the Public Health System from 2008 to 2018. During this period, adjustment for inflation was performed neither regularly nor homogeneously across the CVD groups or procedures. The Brazilian inflation rate (based on the IPCA) from 2008 to 2018 was 76.3%, and the mean inflation for cardiovascular procedures was 43.5%. For some procedure codes, the adjustment was minimal; for coronary stenting, for example, it was 8.7%. Other procedures, however, were adjusted above the inflation rate, such as the treatment of arrhythmias (83.4%).

To minimize biases in reporting and interpreting cost data, a systematic approach was applied to all chapters. Overall costing studies were described in original currency (Reais or US dollars in a specific year) and international dollars. International dollars were converted to PPP adjusted to 2018 US dollars (2018 Int$) using the Campbell and Cochrane Economics Methods Group Evidence for Policy and Practice Information and Coordination Centre cost converter ( https://eppi.ioe.ac.uk/costconversion/default.aspx) . A two-stage approach is applied in this method. First, it adjusts the original estimate of cost from the original price-year to a target price-year, using a GDP deflator index (GDPD values). Second, it converts the price-year adjusted cost estimate in the original currency to a target currency, using conversion rates based on PPP for GDP (PPP values).^27^For original economic studies, when the base year of the currency was not reported or could not be inferred from the manuscript (e.g., the last year of data collection), the recommendation was to assume the year before the publication of the paper.

## 1. TOTAL CARDIOVASCULAR DISEASES

### ICD-9 390 to 459; ICD-10 I00 to I99.

**See Tables 1-1 through 1-9 and Charts 1-1 through 1-16**

**Table:** 

Abbreviations Used in Chapter 1	
ACS	Acute Coronary Syndrome
AHA	American Heart Association
AMI	Acute Myocardial Infarction
CABG	Coronary Artery Bypass Grafting
CI	Confidence Interval
CVD	Cardiovascular Disease
DALY	Disability-Adjusted Life Year
DATASUS	Brazilian Unified Health System Database
ELSA-Brasil	Brazilian Longitudinal Study of Adult Health
ELSI-Brasil	Brazilian Longitudinal Study of the Elderly Health and Wellness
FHP	Family Health Program
FU	Federative Unit
GBD	Global Burden of Disease
GDP	Gross Domestic Product
HDI	Human Development Index
HDIm	Municipal Human Development Index
HF	Heart Failure
IBGE	Brazilian Institute of Geography and Statistics
ICD	International Statistical Classification of Diseases and Related Health Problems
IHD	Ischemic Heart Disease
NCD	Noncommunicable Chronic Disease
NHS	National Health System
OR	Odds Ratio
PAR	Population Attributable Risks
RR	Relative Risk
SDI	Sociodemographic Index
SIDRA	IBGE Automated Retrieval System (in Portuguese, *Sistema IBGE de Recuperação Automática* )
SIH	Brazilian Hospital Information System (in Portuguese, *Sistema de Informações Hospitalares* )
SIM	Brazilian Mortality Information System (in Portuguese, *Sistema de Informações sobre Mortalidade* )
SUS	Brazilian Unified Health System (in Portuguese, *Sistema Único de Saúde* )
UI	Uncertainty Interval

**Table 1-1:** – Age-standardized prevalence rates of CVD per 100 000 inhabitants, by sex, in Brazil, 1990 and 2017, and percent change

Federative Units	Both sexes			Female			Male		
	1990	2017	Percent change	1990	2017	Percent change	1990	2017	Percent change
Brazil	6290 (6048.3;6548.9)	6025 (5785.8;6274.8)	-4.2 (-3.2;-5.1)	5939.5 (5694.8;6205.5)	5612.9 (5366.3;5856.6)	-5.5 (-4.2;-6.7)	6697.3 (6433.7;6961.9)	6536.8 (6282.7;6806.6)	-2.4 (-1.3;-3.4)
Acre	6011.6 (5762.4;6272.3)	5814.9 (5568.2;6071.4)	-3.3 (-1.4;-5.3)	5595.5 (5340.7;5865.5)	5350.3 (5101.6;5618.9)	-4.4 (-1.5;-7.2)	6361.8 (6076.6;6659.2)	6299 (6033;6583.4)	-1 (1.8;-3.9)
Alagoas	5960.7 (5700.5;6233.7)	5790.1 (5543.7;6044.7)	-2.9 (-0.8;-4.8)	5603.9 (5346;5888.2)	5381 (5123;5659.8)	-4 (-1;-6.9)	6360 (6070.2;6651.8)	6307.2 (6024.2;6580.8)	-0.8 (2;-3.5)
Amapá	6219.5 (5950.1;6490.2)	6185.3 (5922.8;6478.7)	-0.5 (1.5;-2.7)	5945.1 (5646.7;6260.9)	5817.8 (5542.5;6137.7)	-2.1 (1.3;-5.4)	6515.4 (6227.7;6811.6)	6587.8 (6304.5;6889.1)	1.1 (3.8;-1.6)
Amazonas	5728.7 (5485.7;5987.5)	5701.6 (5455.9;5955)	-0.5 (1.8;-2.6)	5367.7 (5112.8;5643.3)	5244.5 (4984.1;5506.4)	-2.3 (0.8;-5.5)	6092 (5823.9;6361.4)	6183.2 (5892.3;6475.4)	1.5 (4.5;-1.3)
Bahia	5760.9 (5521.5;6019.9)	5685.9 (5433.9;5942.7)	-1.3 (0.9;-3.4)	5420.2 (5164;5706.9)	5247 (4989.7;5508.9)	-3.2 (-0.2;-6)	6147.9 (5863.3;6431.4)	6225 (5936.4;6513.3)	1.3 (4.3;-1.6)
Ceará	5860.4 (5600.4;6118.9)	5943.7 (5668.7;6231.8)	1.4 (3.8;-0.9)	5545.9 (5270.6;5820.4)	5541.3 (5256.3;5850.7)	-0.1 (3.2;-3.3)	6211.7 (5923.9;6506.7)	6448.9 (6145.4;6775.2)	3.8 (7.1;0.7)
Distrito Federal	5798.5 (5563.4;6037.2)	5529.8 (5303.6;5752.8)	-4.6 (-2.7;-6.5)	5461.1 (5211.3;5732.7)	5144.9 (4908.1;5382.3)	-5.8 (-3;-8.7)	6209.3 (5957.1;6458.7)	6061.9 (5797;6321.5)	-2.4 (0.3;-4.9)
Espírito Santo	6214.8 (5957;6478.6)	5748.2 (5490.1;5998.6)	-7.5 (-5.6;-9.5)	5868.6 (5600.7;6137.1)	5341.6 (5086.6;5596.6)	-9 (-6.1;-11.8)	6591.9 (6320.7;6883.9)	6240.1 (5950.8;6518.6)	-5.3 (-2.8;-7.8)
Goiás	5642.8 (5395.9;5901.5)	5527.3 (5295.5;5762.8)	-2 (0.1;-4.2)	5318.3 (5037.2;5601.6)	5136.8 (4890.8;5398.2)	-3.4 (0;-6.6)	5947.9 (5701.8;6217.5)	5969.3 (5701.1;6245.8)	0.4 (3.2;-2.6)
Maranhão	5596.3 (5341.3;5857.4)	5592.5 (5348.5;5844.7)	-0.1 (2;-2.1)	5196.3 (4944.4;5463.5)	5174.8 (4941.4;5424)	-0.4 (2.6;-3.3)	6026.1 (5740.7;6311.6)	6065.4 (5785.2;6348.3)	0.7 (3.5;-2.2)
Mato Grosso	5995.6 (5727.7;6258.6)	5869.7 (5612.3;6121.4)	-2.1 (0.2;-4.1)	5622 (5350.5;5896.6)	5441.8 (5189.2;5704.2)	-3.2 (-0.1;-6)	6297.7 (6001.6;6588.6)	6285.3 (5998.3;6582.1)	-0.2 (2.8;-3)
Mato Grosso do Sul	6168.4 (5915.1;6427.7)	5964.9 (5727.2;6210.6)	-3.3 (-1.3;-5.3)	5730.5 (5467.9;5987.4)	5472.6 (5219.8;5732.1)	-4.5 (-1.6;-7.4)	6571.7 (6308.5;6860.6)	6512.2 (6248.7;6790.3)	-0.9 (1.8;-3.7)
Minas Gerais	6269 (5985.9;6552.4)	6031.6 (5768.1;6308.7)	-3.8 (-1.4;-5.9)	5902 (5602;6201.4)	5614.5 (5333.5;5907.9)	-4.9 (-1.8;-7.9)	6699.1 (6401.3;6989.1)	6532.8 (6246.7;6839)	-2.5 (0.2;-5)
Pará	5842.4 (5588.6;6111.7)	5751 (5495.1;6017.5)	-1.6 (0.6;-3.6)	5511.2 (5245.2;5786.3)	5291.5 (5022.7;5567.5)	-4 (-0.7;-7.2)	6191.5 (5918.1;6485.6)	6236.9 (5947.8;6540.6)	0.7 (3.7;-2)
Paraíba	5802.6 (5546.1;6053.4)	5755.9 (5515.6;6004.5)	-0.8 (1.4;-3)	5477.7 (5212.5;5728.9)	5371 (5114.4;5651.9)	-1.9 (1.3;-5)	6170.5 (5892.1;6456.2)	6253.3 (5978.4;6539.4)	1.3 (4.1;-1.5)
Paraná	6350.3 (6083.3;6621.1)	5998 (5747.6;6250.1)	-5.5 (-3.5;-7.6)	5947.5 (5671;6238.5)	5549.2 (5289.1;5808)	-6.7 (-3.8;-9.5)	6765.8 (6472.5;7054)	6538.5 (6262.5;6813.3)	-3.4 (-0.5;-6)
Pernambuco	5864.3 (5618.3;6114.8)	5642.3 (5399.5;5887.5)	-3.8 (-1.7;-5.9)	5544.4 (5296;5814.4)	5239.5 (4986.3;5490.8)	-5.5 (-2.5;-8.3)	6252.9 (5972.2;6545.7)	6184.6 (5912.5;6459.7)	-1.1 (1.7;-3.9)
Piauí	5511.3 (5276.3;5755.1)	5545.1 (5314.7;5786.3)	0.6 (2.8;-1.4)	5145.2 (4900.4;5394.3)	5131.4 (4894.1;5385.3)	-0.3 (2.8;-3.3)	5911.2 (5642.8;6176.5)	6034.4 (5776.8;6317.2)	2.1 (4.8;-0.6)
Rio de Janeiro	6714.2 (6446.8;7008.6)	6230.8 (5980.5;6490.7)	-7.2 (-5.3;-9.2)	6350.5 (6067.4;6659.1)	5820 (5546.8;6108.8)	-8.4 (-5.6;-11.1)	7208.1 (6917.8;7498.2)	6800.2 (6527.3;7073.1)	-5.7 (-3.1;-8.1)
Rio Grande do Norte	5701.2 (5452.1;5951.2)	5672.5 (5438;5939.2)	-0.5 (1.6;-2.4)	5393 (5131.9;5659.9)	5280.7 (5029.7;5550.9)	-2.1 (0.8;-5)	6041.8 (5780.8;6327.3)	6170.4 (5892.3;6466.5)	2.1 (5.1;-0.6)
Rio Grande do Sul	6600.3 (6315.3;6880.9)	6182.1 (5906.1;6460.9)	-6.3 (-4.4;-8.3)	6322.7 (6028.2;6645.2)	5828.4 (5536.3;6116.6)	-7.8 (-4.9;-10.6)	6961.6 (6661.6;7269.6)	6639.1 (6336.7;6946.7)	-4.6 (-2;-7.3)
Rondônia	5985.6 (5731;6240.1)	5705.1 (5457.5;5949.3)	-4.7 (-2.4;-6.8)	5599.4 (5348.2;5868.1)	5285.8 (5041.4;5543.5)	-5.6 (-2.6;-8.6)	6276.9 (5998.5;6564.3)	6111.8 (5837.6;6398.4)	-2.6 (0.4;-5.3)
Roraima	6064.1 (5818.4;6308.8)	5814.1 (5583.7;6060.7)	-4.1 (-2.1;-6)	5617 (5371;5884)	5317.9 (5070;5562.2)	-5.3 (-2.4;-8.1)	6407.7 (6148.3;6694.1)	6269.6 (6021.5;6541.3)	-2.2 (0.4;-4.9)
Santa Catarina	6679.1 (6397.8;6964.3)	6217 (5941.2;6488.4)	-6.9 (-5;-8.8)	6375.3 (6069.7;6674.6)	5844.5 (5564.4;6133.5)	-8.3 (-5.4;-11.1)	7026.3 (6731;7339.3)	6667 (6373.5;6959.6)	-5.1 (-2.3;-7.8)
São Paulo	6801.7 (6517.3;7096.7)	6423.9 (6151.9;6705.8)	-5.6 (-3.4;-7.5)	6406.6 (6114.7;6715.3)	6000.7 (5715;6302.4)	-6.3 (-3.1;-9.3)	7284.4 (6975.5;7612.3)	6975.7 (6661.7;7268.7)	-4.2 (-1.4;-6.8)
Sergipe	5922.8 (5667.3;6171.5)	5851.4 (5597.1;6110.4)	-1.2 (1.2;-3.4)	5582.1 (5328.1;5864.7)	5442.8 (5194.8;5717)	-2.5 (0.7;-5.3)	6324.1 (6036.1;6611.2)	6374.1 (6091.1;6662.7)	0.8 (3.9;-1.8)
Tocantins	5849.3 (5606.1;6103.5)	5849.2 (5606.6;6094.9)	0 (2.1;-2.1)	5387.1 (5153.3;5644.6)	5307.1 (5065.3;5571.5)	-1.5 (1.5;-4.3)	6261.9 (5993.8;6548.1)	6368.8 (6094;6643.8)	1.7 (4.6;-1)

*Source: Global Burden of Disease Study 2017, Institute for Health Metrics and Evaluation.^66^*

**Table 1-2:** – Number of cases and age-standardized incidence rates (per 100 000) of CVD, in Brazil and its Federative Units, 1990 and 2017, and percent change

Brazilian Federative Units	1990		2017		Percent change (95% UI)
	Number (95% UI)	Rate (95% UI)	Number (95% UI)	Rate (95% UI)	
Brazil	723595.1 (700074.3;750549.6)	755.6 (731.6;783)	1550188.5 (1495647.3;1607115.8)	687.5 (663.4;712.4)	-9 (-9.9;-8.2)
Acre	1400.9 (1352.7;1453.7)	701.5 (675.5;730.5)	4219.8 (4070.7;4381.3)	673.9 (648.3;700.9)	-3.9 (-5.4;-2.4)
Alagoas	10692.8 (10301.7;11114.3)	715.2 (688;744.7)	21304 (20503.3;22148.2)	675.8 (649.9;702.9)	-5.5 (-7;-4)
Amapá	845.7 (817.2;875.8)	697.3 (672.7;723.9)	3589.8 (3464.8;3720.7)	680.8 (655.6;706.2)	-2.4 (-3.9;-0.9)
Amazonas	6580.8 (6356.7;6809.1)	683.2 (658.2;709.5)	19228.5 (18527.4;19964.4)	667.3 (643.2;695.3)	-2.3 (-3.7;-0.9)
Bahia	53499.5 (51571.2;55573.5)	715.2 (689.1;744.7)	108386.9 (104426.4;112633.4)	686 (660.4;713)	-4.1 (-5.6;-2.5)
Ceará	29391.6 (28364.7;30541.6)	668.4 (644.1;695.3)	64457.4 (61963.2;67082.7)	657.3 (631.5;684.4)	-1.7 (-3.2;-0.1)
Distrito Federal	5460.4 (5280.9;5656.2)	735.9 (711.2;762.2)	17461 (16845.3;18144.4)	681 (657.1;706.6)	-7.5 (-8.8;-6.1)
Espírito Santo	12056.9 (11646.4;12504.3)	761.1 (733.7;789.4)	28206.7 (27136.4;29299.3)	669.3 (644.8;695.5)	-12.1 (-13.6;-10.6)
Goiás	16069.6 (15492.1;16700.6)	721.2 (695.6;749.2)	44756.7 (43051.4;46526.6)	667.8 (643.2;694.3)	-7.4 (-8.8;-6.1)
Maranhão	19179.5 (18453.2;19975.2)	668.8 (642.1;698.5)	42991.2 (41329.7;44766)	660.5 (634.6;689.6)	-1.3 (-2.8;0.3)
Mato Grosso	6719.6 (6494.8;6965.5)	704 (679.9;731.2)	21357.5 (20543;22189.1)	675.9 (649.6;702.9)	-4 (-5.4;-2.4)
Mato Grosso do Sul	7537.2 (7281.1;7807.5)	744.2 (717.8;772.7)	19409.2 (18668.4;20188)	693.7 (667.6;721.3)	-6.8 (-8.1;-5.4)
Minas Gerais	80523.3 (77820.9;83428.5)	768.2 (742;795.2)	171252.6 (164841.8;177705.6)	685.1 (660.4;709.9)	-10.8 (-12.3;-9.4)
Pará	17011.1 (16426.2;17625.4)	688.6 (662.5;716.5)	45519.2 (43870.8;47258)	661.7 (637.2;688.8)	-3.9 (-5.4;-2.5)
Paraíba	16176.5 (15565.7;16826.5)	667.2 (642.6;693.4)	29872.4 (28740.8;31073.8)	657.2 (632.5;684.2)	-1.5 (-3;0)
Paraná	41419.6 (40008.6;42968.7)	800.3 (773;830.5)	87883.8 (84611.7;91450.2)	695.9 (670.7;722.7)	-13 (-14.5;-11.6)
Pernambuco	35110.1 (33804.7;36547.8)	732.2 (705.7;761.9)	66763.4 (64155.4;69477.8)	675.2 (649.1;703)	-7.8 (-9.3;-6.2)
Piauí	10631.7 (10240.3;11034.3)	670.2 (644.4;697.2)	23550.9 (22654.2;24486.5)	655.2 (629.6;681.5)	-2.2 (-3.7;-0.7)
Rio de Janeiro	81868.9 (78983.8;85145.6)	821.9 (793.4;852.5)	150842.8 (145276.6;156396.1)	709 (683.9;734.5)	-13.7 (-15.1;-12.3)
Rio Grande do Norte	11276.8 (10853;11708.8)	654.7 (629.9;680.7)	24101.3 (23205;25082.8)	646.6 (621.9;674.4)	-1.2 (-2.6;0.3)
Rio Grande do Sul	53751.8 (51853.1;55798.7)	788.9 (761.7;817.8)	101785.9 (97835;105973.3)	695.5 (669.9;722.4)	-11.8 (-13.3;-10.3)
Rondônia	3371.8 (3252.6;3505)	720.6 (695.4;749.2)	9991.1 (9629.9;10396.4)	668.7 (643.9;697)	-7.2 (-8.7;-5.7)
Roraima	568.1 (548.3;589.3)	720.4 (694.8;748.1)	2537.1 (2444.2;2635)	677 (651.8;703.1)	-6 (-7.4;-4.7)
Santa Catarina	21007.9 (20283;21746.3)	763.1 (736.1;790.2)	52679.6 (50763.8;54847.6)	685.8 (661.2;712.9)	-10.1 (-11.6;-8.7)
São Paulo	171665.5 (165924.5;178117.1)	803.6 (775.3;833.8)	364049.8 (350776.2;377905.8)	707.2 (681.6;733.4)	-12 (-13.4;-10.6)
Sergipe	6408.5 (6185.3;6643.9)	693.3 (667.4;720.5)	14467 (13942.5;15036.2)	667.5 (642.1;694.7)	-3.7 (-5.2;-2.2)
Tocantins	3369.1 (3254.2;3500)	711.8 (686.8;739.6)	9522.9 (9187.6;9895.5)	676.5 (652.2;704)	-5 (-6.5;-3.5)

*UI: uncertainty interval; Source: Global Burden of Disease Study 2017, Institute for Health Metrics and Evaluation.^66^*

**Table 1-3:** – Number of deaths and age-standardized mortality rates (per 100 000) due to CVD, in Brazil and its Federative Units, 1990 and 2017, and percent change

	1990		2017		Percent change (95% UI)
	Number (95% UI)	Rate (95% UI)	Number (95% UI)	Rate (95% UI)	
Brazil	266957.7 (264384.5;269670.5)	341.8 (338.7;345.2)	388268.1 (383814.8;392697.7)	178 (175.9;180)	-47.9 (-48.5;-47.2)
Acre	398.2 (388.1;407.4)	276.3 (269.5;282.7)	860.3 (827.9;893.3)	158.5 (152.2;164.7)	-42.6 (-45.5;-39.5)
Alagoas	4053.8 (3962.9;4148.6)	312.4 (306.1;318.9)	6330.3 (6126.2;6536.9)	211.9 (204.9;218.9)	-32.2 (-34.8;-29.5)
Amapá	204.7 (199.9;209.6)	252.5 (246.8;258.8)	668.5 (646.7;691.1)	157.2 (152.2;162.6)	-37.8 (-40.2;-35.1)
Amazonas	1671.4 (1607.3;1730)	248.9 (239.5;257.4)	3566.2 (3452.4;3679.5)	147 (142.3;151.9)	-40.9 (-43.7;-37.8)
Bahia	16748.2 (15951.3;17572.4)	252.2 (240.6;264.6)	25924.4 (25261.1;26649.5)	162.9 (158.6;167.6)	-35.4 (-38.8;-31.7)
Ceará	8157.7 (7634.1;8698.6)	200.3 (187.1;213.5)	15199.6 (14788.3;15643.8)	152.4 (148.2;156.9)	-24 (-28.8;-18.3)
Distrito Federal	1564 (1530.6;1593.1)	347.4 (340.8;353.3)	3196.5 (3061;3340.7)	175.4 (168;183.2)	-49.5 (-51.8;-47)
Espírito Santo	4547.7 (4479.3;4621.1)	409.1 (403.1;415.6)	6692 (6473.6;6899.1)	165.8 (160.3;171)	-59.5 (-60.9;-58.1)
Goiás	5034.5 (4904.9;5160.8)	326.3 (317.6;334.9)	10071.3 (9753.2;10423.5)	163.9 (158.7;169.5)	-49.8 (-51.4;-48)
Maranhão	6355.1 (5965.1;6814.3)	250.6 (234.1;269)	11471.5 (11001.2;11997)	184.6 (177;193.1)	-26.3 (-30.8;-22)
Mato Grosso	1969.9 (1862.5;2089.6)	288.6 (274.1;305)	4470.8 (4292.6;4648.3)	162.8 (156.5;169.3)	-43.6 (-47.1;-39.8)
Mato Grosso do Sul	2619.6 (2546.5;2676.5)	351.7 (344.2;358.2)	5149.6 (4987.1;5336)	198.6 (192.5;205.5)	-43.5 (-45.6;-41.2)
Minas Gerais	29369 (28899.2;29849.4)	357.1 (351.8;362.5)	38721.8 (37782;39823.5)	154.5 (150.8;159)	-56.7 (-58;-55.3)
Pará	5266.9 (5055.8;5475)	280 (269.1;290.5)	10353.6 (9971.8;10725.7)	168.6 (162.5;174.7)	-39.8 (-43.2;-36.4)
Paraíba	5718.5 (5474.8;5964.4)	254.9 (244.2;265.8)	8984.7 (8426.7;9582.7)	190.9 (179;203.9)	-25.1 (-30.9;-18.6)
Paraná	16504.7 (16270.4;16731.9)	445.3 (438.8;451.5)	22160.3 (21559.6;22775.3)	188.3 (183.3;193.6)	-57.7 (-59.1;-56.4)
Pernambuco	14360.8 (14092.9;14607.3)	364.9 (358.4;371)	20620.7 (20029;21264.3)	214.6 (208.3;221.3)	-41.2 (-43;-39.3)
Piauí	3514.8 (3300.4;3730.5)	262.9 (247.1;279.1)	6327.5 (6132.3;6535.5)	175.1 (169.7;181)	-33.4 (-37.3;-28.8)
Rio de Janeiro	37561.8 (37050.8;38028.3)	456.7 (450.5;462.1)	43858 (42710.9;45057.9)	207.7 (202.4;213.3)	-54.5 (-55.8;-53.2)
Rio Grande do Norte	3386.4 (3206.1;3566.2)	213.3 (202;224.8)	6068 (5855.2;6289.3)	159.2 (153.6;165.1)	-25.4 (-30;-19.9)
Rio Grande do Sul	20393.7 (20091.6;20681.9)	378.1 (372.6;383.2)	25992.2 (25243.6;26731.3)	177.2 (172.1;182.3)	-53.1 (-54.6;-51.6)
Rondônia	1006.4 (941.1;1071.3)	368.1 (347.8;387.7)	2351.5 (2146.6;2592.9)	184.8 (169.6;202.6)	-49.8 (-54.8;-44.5)
Roraima	146.8 (135.1;159.4)	373.6 (349.4;399.7)	495.4 (447.4;549.9)	196.3 (178.6;216)	-47.5 (-53.5;-41.2)
Santa Catarina	7674.7 (7557;7795.4)	389.1 (383.2;395.1)	11764.8 (11388.8;12125)	170.2 (164.9;175.2)	-56.3 (-57.8;-54.7)
São Paulo	65661.8 (64717.7;66544.9)	401.2 (395.5;406.2)	91136 (88785.8;93412.4)	185.6 (180.7;190.2)	-53.8 (-55;-52.5)
Sergipe	2100.9 (2048.7;2155)	252.8 (246.6;259.1)	3529.6 (3404.6;3655.8)	171.6 (165.6;177.7)	-32.1 (-35;-29.2)
Tocantins	965.7 (864.4;1053.9)	329.1 (304.8;353.4)	2302.8 (2183.2;2429)	173.9 (165.1;183.1)	-47.2 (-51.4;-42.2)

*UI: uncertainty interval; Source: Global Burden of Disease Study 2017, Institute for Health Metrics and Evaluation.^66^*

**Table 1-4:** – Proportional mortality from cardiovascular diseases (CVD), ischemic heart diseases (IHD) and stroke, by Brazilian region and Federative Unit, and in Brazil, 2017

Region Federative Unit	CVD/Total %	IHD/CVD %	Stroke/CVD %
**North**	**22.9**	**30.6**	**33.4**
Rondônia	24.2	33.0	27.7
Acre	22.3	27.6	32.4
Amazonas	18.1	27.7	36.9
Roraima	21.3	24.8	30.3
Pará	23.4	31.4	35.1
Amapá	19.6	32.3	34.2
Tocantins	30.8	31.0	27.7
**Northeast**	**27.2**	**32.2**	**30.3**
Maranhão	30.8	33.0	33.2
Piauí	32.5	30.0	33.1
Ceará	26.3	31.4	31.7
Rio Grande do Norte	25.8	38.9	24.0
Paraíba	29.2	35.4	26.1
Pernambuco	28.1	37.8	28.2
Alagoas	30.2	29.6	30.2
Sergipe	23.5	29.5	31.5
Bahia	24.0	26.4	31.8
**Southeast**	**28.3**	**32.4**	**25.7**
Minas Gerais	25.4	24.4	28.4
Espírito Santo	28.8	36.8	28.0
Rio de Janeiro	27.9	32.9	25.1
São Paulo	29.8	34.9	24.8
**South**	**27.2**	**31.1**	**30.2**
Paraná	28.3	29.4	29.5
Santa Catarina	27.4	32.4	25.7
Rio Grande do Sul	26.2	32.1	32.9
**West-Central**	**26.4**	**33.0**	**28.4**
Mato Grosso do Sul	28.6	37.9	29.3
Mato Grosso	24.3	31.2	27.6
Goiás	26.2	31.8	26.3
Distrito Federal	26.7	32.2	35.1
**BRAZIL**	**27.3**	**32.1**	**28.2**

*Source: Brazilian Mortality Information System (Sistema de Informações sobre Mortalidade – SIM/DATASUS).56*

**Table 1-5:** – Procedure codes included in each group of conditions for cost analysis

Type	Disease Group	Code	Code Description
Surgical	Atrial Fibrillation Ablation	0406050074	*Estudo Eletrofisiológico Terapêutico II (Ablação de Fibrilação atrial)*
Surgical	Coronary Angioplasty	0406030073	*Angioplastia em Enxerto Coronariano (c/ Implante de Stent)*
Surgical	Coronary Angioplasty	0406030014	*Angioplastia Coronariana*
Surgical	Coronary Angioplasty	0406030065	*Angioplastia em Enxerto Coronariano*
Surgical	Coronary Angioplasty	0406030022	*Angioplastia Coronariana c/ Implante de dois Stents*
Surgical	Coronary Angioplasty	0406030030	*Angioplastia Coronariana c/ Implante de Stent*
Surgical	Coronary Artery Bypass Graft Surgery	0406010927	*Revascularização Miocárdica c/ uso de Extracorpórea*
Surgical	Coronary Artery Bypass Graft Surgery	0406010935	*Revascularização Miocárdica c/ uso de Extracorpórea (c/ 2 ou mais enxertos)*
Surgical	Coronary Artery Bypass Graft Surgery	0406010943	*Revascularização Miocárdica s/ uso de Extracorpórea*
Surgical	Coronary Artery Bypass Graft Surgery	0406010951	*Revascularização Miocárdica s/ uso de Extracorpórea (c/ 2 ou mais enxertos)*
Surgical	Valvular Surgery	0406010811	*Plástica valvar c/ Revascularização Miocárdica*
Surgical	Valvular Surgery	0406010340	*Correção de Insuficiência da Válvula Tricúspide*
Surgical	Valvular Surgery	0406010692	*Implante de Prótese Valvar*
Surgical	Valvular Surgery	0406010021	*Abertura de estenose Aórtica Valvar*
Surgical	Valvular Surgery	0406010803	*Plástica Valvar*
Surgical	Valvular Surgery	0406010030	*Abertura de Estenose Pulmonar Valvar*
Surgical	Cardiomyopathies	0406011397	*Correção de Hipertrofia Septal Assimétrica (criança e adolescente)*
Surgical	Valvular Surgery	0406011206	*Troca valvar c/ Revascularização Miocárdica*
Surgical	Primary Angioplasty	0406030049	*Angioplastia Coronariana Primária*
Surgical	Cardiomyopathies	0406011397	*Correção de Hipertrofia Septal Assimétrica (criança e adolescente)*
Surgical	Other Valvuloplasties	0406030146	*Valvuloplastia Tricúspide Percutânea*
Surgical	Other Valvuloplasties	0406030138	*Valvuloplastia Pulmonar Percutânea*
Surgical	Other Valvuloplasties	0406030111	*Valvuloplastia Aórtica Percutânea*
Surgical	Mitral Valvuloplasties	0406030120	*Valvuloplastia Mitral Percutânea*
Clinical	Chronic Ischemic Heart Disease	0303060042	*Tratamento de Cardiopatia Isquêmica Crônica*
Clinical	Stroke	0303040149	*Tratamento de Acidente Vascular Cerebral - AVC (isquêmico ou hemorrágico agudo)*
Clinical	Stroke	0303040076	*Tratamento Conservador da Hemorragia Cerebral*
Clinical	Stroke	0303040300	*Tratamento do Acidente Vascular Cerebral Isquêmico Agudo com uso de Trombolítico*
Clinical	Valve Diseases	0303060123	*Tratamento de Doença Reumática s/ Cardite*
Clinical	Valve Diseases	0303060115	*Tratamento de Doença Reumática c/ Comprometimento Cardíaco*
Clinical	Atrial Fibrillation	0303060026	Tratamento de Arritmias*
Clinical	Myocardial Infarction – Clinical Treatment	0303060190	*Tratamento de Infarto Agudo do Miocárdio*
Clinical	Heart Failure	0303060131	*Tratamento de Edema Agudo de Pulmão*
Clinical	Heart Failure	0303060212	*Tratamento de Insuficiência Cardíaca*
Clinical	Cardiomyopathies	0303060239	*Tratamento de Miocardiopatias*
Clinical	Cardiomyopathies	0303060034	*Tratamento de Cardiopatia Hipertrófica*
Clinical	Acute Coronary Syndromes	0303060280	*Tratamento de Síndrome Coronariana Aguda*

*If ICD 10 I48

*Source: Brazilian Mortality Information System (Sistema de Informações sobre Mortalidade – SIM/DATASUS).^56^*

**Table 1-6:** – Procedures paid by the SUS from 2008 to 2018, by group of procedures

Group of Procedures	Number of procedures paid by the SUS
**Cardiomyopathies and Heart Failure**	
Heart Failure	2 862 739
Cardiomyopathies	24 964
**Ischemic Heart Disease**	
Chronic, Clinical Tx	87 894
Myocardial Infarction, Clinical Tx	676 467
Primary Angioplasty	85 664
Acute Coronary Syndrome, Clinical Tx	784 921
Coronary Angioplasty	669 893
CABG	244 105
**Stroke**	2 042 195
**Valve Disease**	
Clinical Tx	32 795
Surgeries	139 131
Mitral Valvuloplasty	4204
Other Valvuloplasties	5087
**Atrial Fibrillation**	321 866
Atrial Fibrillation Ablation	1250

*CABG: Coronary Artery Bypass Grafting; Tx: Treatment.*

*Source: Brazilian Mortality Information System (Sistema de Informações sobre Mortalidade – SIM/DATASUS).^56^*

**Table 1-7:** – Annual absolute number and rates of procedures paid by the SUS, from 2008 to 2018, by disease groups*

Procedure code	2008		2009		2010		2011		2012		2013		2014		2015		2016		2017		2018	
	**N**	**per 100 000**	**N**	**per 100 000**	**N**	**per 100 000**	**N**	**per 100 000**	**N**	**per 100 000**	**N**	**per 100 000**	**N**	**per 100 000**	**N**	**per 100 000**	**N**	**per 100 000**	**N**	**per 100 000**	**N**	**per 100 000**
**Cardiomyopathies and Heart Failure**																						
Heart Failure	298,474	157.41	297,763	155.48	289,11	151.58	284,844	148.06	264,469	136.34	254,285	126.47	243,913	120.27	240,832	117.78	236,358	114.67	230,297	110.90	222,394	106.67
Cardiomyopathies	2,092	1.10	2,363	1.23	2,459	1.29	2,302	1.20	2,357	1.22	2,293	1.14	2,370	1.17	2,230	1.09	2,250	1.09	1,997	0.96	2,251	1.08
**Ischemic Heart Disease**																						
Chronic, Clinical Tx	12,393	6.54	9,743	5.09	9,300	4.88	8,497	4.42	8	4.12	7,197	3.58	7,581	3.74	6,403	3.13	6,317	3.06	6,171	2.97	6,292	3.02
Myocardial Infarction, Clinical Tx	47,358	24.98	50,987	26.62	55,513	29.11	58,194	30.25	59,562	30.71	58,552	29.12	62,809	30.97	66,647	32.59	70,441	34.18	71,835	34.59	74,569	35.77
Primary Angioplasty	7,648	4.03	6,362	3.32	6,262	3.28	6,033	3.14	5,865	3.02	6,055	3.01	7,135	3.52	8,524	4.17	10,195	4.95	10,774	5.19	10,811	5.19
Acute Coronary Syndrome, Clinical Tx	63,300	33.38	68,833	35.94	72,912	38.23	71,523	37.18	75,734	39.04	73,432	36.52	76,945	37.94	72,686	35.55	70,430	34.17	70,713	34.05	68,413	32.81
Coronary Angioplasty	38,635	20.38	45,648	23.84	49,492	25.95	55,931	29.07	60,959	31.43	63,838	31.75	66,492	32.79	66,55	32.55	69,802	33.87	73,971	35.62	78,575	37.69
CABG	20,515	10.82	22,077	11.53	21,225	11.13	23,187	12.05	23,9	12.32	23,249	11.56	22,997	11.34	22,559	11.03	22,248	10.79	21,474	10.34	20,674	9.92
**Stroke**	159,545	84.14	176,047	91.93	181,035	94.92	184,751	96.03	182,065	93.86	183,043	91.04	187,110	92.26	191,678	93.74	195,787	94.99	198,068	95.38	203,066	97.40
**Valve Diseases**																						
Clinical Tx	3,237	1.71	4,156	2.17	3,526	1.85	3,637	1.89	3,285	1.69	2,996	1.49	2,753	1.36	2,400	1.17	2,244	1.09	2,231	1.07	2,330	1.12
Surgeries	12,201	6.43	12,664	6.61	12,169	6.38	13,181	6.85	13,435	6.93	13,067	6.50	12,993	6.41	12,624	6.17	12,432	6.03	12,277	5.91	12,088	5.80
Mitral Valvuloplasty	477	0.25	551	0.29	478	0.25	473	0.25	403	0.21	431	0.21	408	0.20	341	0.17	206	0.10	236	0.11	200	0.10
Other Valvuloplasties	451	0.24	477	0.25	445	0.23	486	0.25	456	0.24	527	0.26	515	0.25	513	0.25	399	0.19	427	0.21	391	0.19
**Atrial Fibrillation**																						
AF	29,034	15.31	28,174	14.71	28,382	14.88	28,583	14.86	28,760	14.83	28,268	14.06	29,799	14.69	29,754	14.55	29,889	14.50	30,265	14.57	30,958	14.85
AF Ablation	68	0.04	72	0.04	90	0.05	85	0.04	123	0.06	139	0.07	143	0.07	161	0.08	124	0.06	120	0.06	125	0.06

* Disease groups according to the procedure codes of Table 1-5.

*AF: atrial fibrillation, CABG: Coronary Artery Bypass Grafting, Tx: treatment.*

*Source: Brazilian Mortality Information System (Sistema de Informações sobre Mortalidade – SIM/DATASUS).^56^*

**Table 1-8:** – Total unadjusted and adjusted for 2018Int$ reimbursement values of clinical cardiovascular admissions by the Brazilian Unified Health System (SUS) from 2008 to 2018. Data expressed in thousands (1,000)

	2008		2009		2010		2011		2012		2013		2014		2015		2016		2017		2018		TOTAL		
	R$	Int$ 2018	R$	Int$ 2018	R$	Int$ 2018	R$	Int$ 2018	R$	Int$ 2018	R$	Int$ 2018	R$	Int$ 2018	R$	Int$ 2018	R$	Int$ 2018	R$	Int$ 2018	R$	Int$ 2018	R$	R$ + IPCA 10 ANOS	Int$ 2018
Chronic Ischemic Heart Disease	7,799	7,519	6,861	6,167	6,593	5,465	6,251	4,781	5,690	4,031	5,248	3,460	6,214	3,798	5,141	2,921	5,327	2,800	5,532	2,811	5,672	2,797	66,328	125,224	46,550
Stroke	142,062	136,975	188,450	169,388	198,813	164,774	205,448	157,139	218,628	154,902	228,141	150,393	242,664	148,315	252,441	143,442	263,771	138,669	272,140	138,253	286,293	141,170	2,498,850	4,717,691	1,643,419
Valvular Diseases	1,052	1,014	1,589	1,428	1,439	1,193	1,607	1,229	1,509	1,069	1,510	995	1,584	968	1,672	950	1,675	881	1,679	853	2,043	1,008	17,361	32,776	11,590
Atrial Fibrillation	13,791	13,297	17,396	15,636	18,537	15,363	18,858	14,424	20,371	14,433	19,969	13,164	22,637	13,835	23,330	13,256	23,929	12,580	26,061	13,239	26,971	13,300	231,850	437,720	152,529
Myocardial Infarction (Clinical)	65,020	62,691	84,308	75,780	92,969	77,051	97,324	74,439	104,898	74,328	106,246	70,040	119,583	73,088	128,724	73,143	134,912	70,925	136,438	69,313	143,349	70,685	1,213,077	2,291,532	791,479
Heart Failure	272,281	262,532	322,849	290,192	327,914	271,771	330,492	252,781	317,586	225,015	321,712	212,077	326,141	199,336	337,610	191,834	345,566	181,670	346,841	176,203	348,832	172,008	3,598,825	6,792,495	2,435,420
Cardiomyopathy	1,288	1,242	1,902	1,709	2,144	1,777	1,900	1,453	2,110	1,495	2,302	1,517	2,696	1,648	2,682	1,524	3,065	1,611	2,556	1,299	3,120	1,538	25,764	48,641	16,813
Acute Coronary Syndromes	44,711	43,110	57,922	52,063	64,612	53,550	65,586	50,165	75,210	53,288	74,619	49,190	83,607	51,100	82,095	46,648	80,185	42,155	82,072	41,694	80,037	39,466	790,657	1,492,716	522,427
**Total**	**548,001**	**528,381**	**681,277**	**612,364**	**713,021**	**590,944**	**727,466**	**556,412**	**746,003**	**528,555**	**759,747**	**500,835**	**805,126**	**492,089**	**833,695**	**473,719**	**858,430**	**451,291**	**873,320**	**443,665**	**896,319**	**441,972**	**8,442,405**	**15,938,795**	**5,620,226**

*Source: Brazilian Hospital Information System (Sistema de Informações Hospitalares – SIH/DATASUS).^29^*

**Table 1-9:** – Total unadjusted and adjusted for 2018Int$ reimbursement values of interventional cardiovascular admissions by the Brazilian Unified Health System (SUS) from 2008 to 2018. Data expressed in thousands (1,000)

	2008		2009		2010		2011		2012		2013		2014		2015		2016		2017		2018		TOTAL		
	R$	Int$ 2018	R$	Int$ 2018	R$	Int$ 2018	R$	Int$ 2018	R$	Int$ 2018	R$	Int$ 2018	R$	Int$ 2018	R$	Int$ 2018	R$	Int$ 2018	R$	Int$ 2018	R$	Int$ 2018	R$	R$ + IPCA	Int$ 2018
Atrial Fibrilation Ablation	360	348	378	339	471	390	457	349	690	489	788	520	771	471	906	515	707	372	691	351	732	361	6,951	11,430	5,636
Coronary angioplasty	210,529	202,991	266,654	239,681	295,641	245,024	337,972	258,502	372,063	263,613	388,920	256,381	411,252	251,355	412,073	234,147	433,590	227,946	466,696	237,091	495,885	244,519	4,091,276	6,728,694	3,317,896
Coronary Artery Bypass Graft Surgery	176,032	169,730	208,585	187,486	214,484	177,762	287,851	220,167	297,844	211,027	290,541	191,528	294,854	180,213	289,638	164,577	286,160	150,439	282,175	143,351	275,110	135,656	2,903,274	4,774,145	2,354,114
Valvular Surgery	125,954	121,445	140,684	126,453	142,383	118,006	179,111	136,995	183,271	129,851	178,564	117,711	180,088	110,069	176,814	100,469	175,319	92,168	176,134	89,481	177,584	87,566	1,835,908	3,018,967	1,488,643
Primary Angioplasty	45,267	43,647	37,888	34,055	37,113	30,759	35,577	27,211	35,545	25,185	37,288	24,581	45,883	28,044	56,101	31,877	66,515	34,968	71,624	36,387	73,429	36,208	542,231	891,645	439,667
Cardiomyopaathies	168,993	163	545	490	192	159	326	249	436	309	354	233	306	187	298	169	527	277	452	230	426	210	4,031	6,623	3,266
Other Valvoplasties	1,519	1,464	1,662	1,493	1,718	1,423	1,919	1,468	1,871	1,325	2,052	1,352	2,128	1,301	2,086	1,185	1,594	838	1,889	960	1,690	833	20,126	33,095	16,319
Mitral Valvoplasty	3,115	3,004	3,585	3,223	3,147	2,608	3,228	2,469	2,718	1,926	2,970	1,958	2,809	1,717	2,394	1,360	1,378	724	1,721	874	1,462	721	28,525	46,907	23,130
**Total**	**562,946**	**542,790**	**659,980**	**593,221**	**695,149**	**576,132**	**846,441**	**647,411**	**894,438**	**633,724**	**901,476**	**594,265**	**938,091**	**573,356**	**940,309**	**534,300**	**965,790**	**507,732**	**1,001,383**	**508,723**	**1,026,318**	**506,074**	**9,432,322**	**15,510,510**	**7,648,180**

*Source: Brazilian Hospital Information System (Sistema de Informações Hospitalares – SIH/DATASUS).^29^*

**Table 2-3:** – Number of deaths and age-standardized mortality rates (per 100 000) for intracerebral hemorrhage and subarachnoid hemorrhage in Brazil and Brazilian Federative Units, 1990 and 2017, and percent change

Cause of death and location	1990		2017		Percent change (95% UI)
	Number (95% UI)	Rate (95% UI)	Number (95% UI)	Rate (95% UI)	
***B.2.3.2 - Intracerebral hemorrhage***					
Acre	76 (72;80)	48,6 (46;51)	169 (157;181)	30,8 (28,4;33)	-36,7 (-42,1;-31,1)
Alagoas	892 (840;948)	66,8 (63;70,9)	1220 (1147;1292)	40,7 (38,3;43,2)	-39 (-43,7;-34,4)
Amapá	37 (34;39)	40,5 (37,7;42,7)	130 (120;140)	29,4 (27;31,7)	-27,3 (-33,1;-20,8)
Amazonas	362 (336;383)	48,7 (45,2;51,4)	761 (711;806)	30,8 (28,8;32,7)	-36,8 (-41,7;-31,4)
Bahia	3297 (3089;3521)	47,9 (44,9;51,1)	4782 (4521;5060)	30,4 (28,6;32,2)	-36,6 (-41,6;-31,4)
Brazil	50247 (49123;51383)	58,6 (57,3;59,9)	61518 (59874;63290)	27,9 (27,1;28,7)	-52,4 (-53,8;-51,1)
Ceará	1646 (1506;1782)	40,2 (36,6;43,6)	2649 (2491;2795)	26,9 (25,3;28,4)	-33,1 (-39,2;-26,5)
Distrito Federal	302 (288;322)	56,1 (53,6;59,1)	498 (463;537)	25,4 (23,6;27,3)	-54,8 (-58,3;-50,7)
Espírito Santo	1035 (996;1079)	79,5 (76,3;82,6)	1124 (1059;1194)	27,6 (26;29,2)	-65,3 (-67,5;-63)
Goiás	1078 (1027;1143)	59,9 (57,1;63,4)	1543 (1447;1638)	24,6 (23;26,1)	-59 (-61,9;-56,1)
Maranhão	1396 (1276;1520)	53,2 (48,5;58,2)	2198 (2042;2416)	35,3 (32,8;38,8)	-33,7 (-39,7;-26,9)
Mato Grosso	325 (300;351)	43,3 (39,9;46,5)	733 (686;785)	25,7 (24,1;27,6)	-40,6 (-46,1;-33,8)
Mato Grosso do Sul	516 (492;540)	60,6 (57,8;63,3)	831 (781;883)	31,3 (29,5;33,3)	-48,2 (-52;-44,6)
Minas Gerais	5752 (5521;6069)	60,5 (58,1;63,3)	6361 (6029;6729)	25,3 (23,9;26,8)	-58,3 (-61,1;-55,5)
Pará	1060 (979;1128)	52,1 (48,1;55,6)	2025 (1888;2161)	32,6 (30,4;34,7)	-37,5 (-43;-31,5)
Paraíba	1008 (938;1082)	44,1 (41;47,2)	1419 (1306;1550)	30,6 (28,1;33,4)	-30,6 (-38,1;-22,5)
Paraná	3442 (3301;3597)	80,3 (76,8;83,8)	3538 (3335;3750)	29,3 (27,7;31,1)	-63,4 (-65,9;-60,8)
Pernambuco	3010 (2866;3154)	70,7 (67,5;74,1)	3459 (3259;3659)	35,8 (33,7;37,9)	-49,4 (-52,5;-45,9)
Piaui	746 (682;809)	53,5 (48,8;57,9)	1156 (1085;1276)	32,2 (30,1;35,5)	-39,9 (-45,1;-33,8)
Rio de Janeiro	7389 (7096;7680)	79,6 (76,6;82,8)	6678 (6329;7056)	31,2 (29,5;32,9)	-60,8 (-63,4;-58,1)
Rio Grande do Norte	515 (477;553)	31,9 (29,5;34,2)	842 (783;908)	22,5 (20,9;24,3)	-29,4 (-36,1;-21,3)
Rio Grande do Sul	3471 (3287;3636)	56,9 (53,9;59,7)	4291 (4020;4547)	28,9 (27,1;30,7)	-49,2 (-52,4;-45,5)
Rondônia	225 (208;243)	69,2 (64;74,6)	394 (352;442)	30 (26,9;33,6)	-56,7 (-61,7;-50,8)
Roraima	28 (25;31)	58,4 (53,7;63,5)	80 (70;91)	29,5 (26;33,4)	-49,5 (-56,8;-41,3)
São Paulo	10472 (10014;10931)	55,4 (52,9;57,9)	11829 (11219;12484)	23,6 (22,3;24,9)	-57,5 (-60,1;-54,3)
Santa Catarina	1508 (1438;1573)	68 (64,8;71)	1727 (1613;1825)	24,4 (22,9;25,9)	-64,1 (-66,6;-61,5)
Sergipe	453 (428;476)	53,7 (50,8;56,5)	677 (638;716)	32,9 (31;34,9)	-38,7 (-43,1;-33,8)
Tocantins	206 (181;230)	58 (52,1;63,5)	404 (373;439)	30,2 (27,9;32,9)	-47,9 (-53,5;-40,8)
***B.2.3.3 - Subarachnoid hemorrhage***					
Acre	19 (18;20)	8,7 (8,1;9,4)	38 (35;41)	6 (5,5;6,6)	-30,8 (-38,1;-23,1)
Alagoas	168 (147;186)	10 (8,8;11)	215 (197;239)	6,7 (6,1;7,5)	-33 (-42,5;-19,3)
Amapá	8 (7;9)	6,4 (5,9;7,5)	29 (26;33)	5,4 (4,9;6,1)	-15,8 (-24,5;-6,4)
Amazonas	70 (64;76)	6,9 (6,4;7,8)	154 (142;169)	5,3 (4,9;5,9)	-23 (-30,9;-14,5)
Bahia	641 (591;706)	8 (7,3;8,9)	968 (899;1065)	6 (5,6;6,7)	-24,2 (-32;-16)
Brazil	9809 (8917;10192)	9,6 (8,8;9,9)	13811 (13189;14611)	6,1 (5,8;6,4)	-36,5 (-39,8;-32,2)
Ceará	340 (294;402)	7,1 (6,2;8,3)	530 (489;573)	5,4 (4,9;5,8)	-24,1 (-37,1;-13,3)
Distrito Federal	81 (70;87)	10,2 (8,8;10,9)	139 (121;153)	5,8 (4,9;6,4)	-43,8 (-49,1;-36,8)
Espírito Santo	191 (155;204)	11,8 (9,6;12,6)	254 (232;274)	5,9 (5,5;6,4)	-49,7 (-54,5;-38,7)
Goiás	219 (202;234)	9,3 (8,6;9,9)	378 (348;416)	5,5 (5,1;6,1)	-40,2 (-45,4;-33,2)
Maranhão	354 (286;418)	10,5 (8,2;12,7)	482 (423;527)	7,3 (6,4;8)	-30,6 (-41,4;-18,5)
Mato Grosso	85 (78;93)	8 (7,3;8,8)	181 (167;199)	5,6 (5,2;6,2)	-29,8 (-37,3;-21,9)
Mato Grosso do Sul	103 (93;111)	9,7 (8,9;10,4)	180 (165;197)	6,3 (5,8;6,9)	-34,8 (-41,1;-27,3)
Minas Gerais	1130 (977;1203)	10,1 (8,9;10,7)	1529 (1402;1639)	6,1 (5,5;6,5)	-40 (-45,2;-33,5)
Pará	191 (175;211)	7,2 (6,7;8,1)	416 (381;459)	5,9 (5,4;6,6)	-17,9 (-25,6;-8,7)
Paraíba	209 (179;243)	8,5 (7,2;9,9)	274 (247;305)	6 (5,4;6,7)	-29,7 (-42,5;-15,7)
Paraná	624 (516;667)	11,7 (9,7;12,5)	810 (739;871)	6,4 (5,8;6,9)	-45,2 (-50,2;-37,7)
Pernambuco	438 (410;487)	8,9 (8,3;10)	665 (615;733)	6,7 (6,2;7,3)	-25,6 (-32,9;-18,1)
Piaui	153 (128;179)	9,1 (7,6;10,7)	216 (200;235)	6 (5,5;6,5)	-34 (-43,5;-21,9)
Rio de Janeiro	1375 (1099;1473)	12,9 (10,4;13,8)	1452 (1314;1571)	6,8 (6,2;7,3)	-47,4 (-52,3;-38,2)
Rio Grande do Norte	100 (88;125)	5,5 (4,8;7,1)	163 (149;212)	4,3 (3,9;5,6)	-21,3 (-32,2;-9,9)
Rio Grande do Sul	593 (554;667)	8,4 (7,8;9,5)	835 (768;922)	5,7 (5,2;6,3)	-32,1 (-38,5;-25,1)
Rondônia	49 (45;53)	9,6 (8,8;10,6)	88 (77;101)	5,8 (5,1;6,6)	-40 (-48,2;-30,3)
Roraima	7 (7;8)	9,1 (8,1;10,9)	19 (17;24)	5,6 (4,9;6,6)	-38,6 (-48,5;-27,7)
São Paulo	2259 (2009;2403)	9,6 (8,8;10,3)	3190 (2945;3415)	6,1 (5,7;6,6)	-36,3 (-42,3;-30)
Santa Catarina	275 (251;292)	9,8 (8,9;10,5)	392 (362;439)	5,2 (4,8;5,8)	-47,4 (-52,3;-40,2)
Sergipe	77 (71;85)	7,8 (7,2;8,7)	125 (115;138)	5,7 (5,2;6,3)	-27,4 (-36,2;-17,9)
Tocantins	48 (38;57)	9,6 (7,9;11,3)	90 (82;99)	6,2 (5,7;6,8)	-35,2 (-46,1;-21,6)

*Source: Global Burden of Disease Study 2017, Institute for Health Metrics and Evaluation.^109^*

**Table 2-1:** – Age-standardized stroke prevalence rates (per 100 000) for both sexes and for males and females, in Brazil and its Federative Units, 1990 and 2017, and percent change

	Both sexes			Female			Male		
	1990	2017	Percent change	1990	2017	Percent change	1990	2017	Percent change
Brazil	6290 (6048.3;6548.9)	6025 (5785.8;6274.8)	-4.2 (-3.2;-5.1)	5939.5 (5694.8;6205.5)	5612.9 (5366.3;5856.6)	-5.5 (-4.2;-6.7)	6697.3 (6433.7;6961.9)	6536.8 (6282.7;6806.6)	-2.4 (-1.3;-3.4)
Acre	6011.6 (5762.4;6272.3)	5814.9 (5568.2;6071.4)	-3.3 (-1.4;-5.3)	5595.5 (5340.7;5865.5)	5350.3 (5101.6;5618.9)	-4.4 (-1.5;-7.2)	6361.8 (6076.6;6659.2)	6299 (6033;6583.4)	-1 (1.8;-3.9)
Alagoas	5960.7 (5700.5;6233.7)	5790.1 (5543.7;6044.7)	-2.9 (-0.8;-4.8)	5603.9 (5346;5888.2)	5381 (5123;5659.8)	-4 (-1;-6.9)	6360 (6070.2;6651.8)	6307.2 (6024.2;6580.8)	-0.8 (2;-3.5)
Amapá	6219.5 (5950.1;6490.2)	6185.3 (5922.8;6478.7)	-0.5 (1.5;-2.7)	5945.1 (5646.7;6260.9)	5817.8 (5542.5;6137.7)	-2.1 (1.3;-5.4)	6515.4 (6227.7;6811.6)	6587.8 (6304.5;6889.1)	1.1 (3.8;-1.6)
Amazonas	5728.7 (5485.7;5987.5)	5701.6 (5455.9;5955)	-0.5 (1.8;-2.6)	5367.7 (5112.8;5643.3)	5244.5 (4984.1;5506.4)	-2.3 (0.8;-5.5)	6092 (5823.9;6361.4)	6183.2 (5892.3;6475.4)	1.5 (4.5;-1.3)
Bahia	5760.9 (5521.5;6019.9)	5685.9 (5433.9;5942.7)	-1.3 (0.9;-3.4)	5420.2 (5164;5706.9)	5247 (4989.7;5508.9)	-3.2 (-0.2;-6)	6147.9 (5863.3;6431.4)	6225 (5936.4;6513.3)	1.3 (4.3;-1.6)
Ceará	5860.4 (5600.4;6118.9)	5943.7 (5668.7;6231.8)	1.4 (3.8;-0.9)	5545.9 (5270.6;5820.4)	5541.3 (5256.3;5850.7)	-0.1 (3.2;-3.3)	6211.7 (5923.9;6506.7)	6448.9 (6145.4;6775.2)	3.8 (7.1;0.7)
Distrito Federal	5798.5 (5563.4;6037.2)	5529.8 (5303.6;5752.8)	-4.6 (-2.7;-6.5)	5461.1 (5211.3;5732.7)	5144.9 (4908.1;5382.3)	-5.8 (-3;-8.7)	6209.3 (5957.1;6458.7)	6061.9 (5797;6321.5)	-2.4 (0.3;-4.9)
Espírito Santo	6214.8 (5957;6478.6)	5748.2 (5490.1;5998.6)	-7.5 (-5.6;-9.5)	5868.6 (5600.7;6137.1)	5341.6 (5086.6;5596.6)	-9 (-6.1;-11.8)	6591.9 (6320.7;6883.9)	6240.1 (5950.8;6518.6)	-5.3 (-2.8;-7.8)
Goiás	5642.8 (5395.9;5901.5)	5527.3 (5295.5;5762.8)	-2 (0.1;-4.2)	5318.3 (5037.2;5601.6)	5136.8 (4890.8;5398.2)	-3.4 (0;-6.6)	5947.9 (5701.8;6217.5)	5969.3 (5701.1;6245.8)	0.4 (3.2;-2.6)
Maranhão	5596.3 (5341.3;5857.4)	5592.5 (5348.5;5844.7)	-0.1 (2;-2.1)	5196.3 (4944.4;5463.5)	5174.8 (4941.4;5424)	-0.4 (2.6;-3.3)	6026.1 (5740.7;6311.6)	6065.4 (5785.2;6348.3)	0.7 (3.5;-2.2)
Mato Grosso	5995.6 (5727.7;6258.6)	5869.7 (5612.3;6121.4)	-2.1 (0.2;-4.1)	5622 (5350.5;5896.6)	5441.8 (5189.2;5704.2)	-3.2 (-0.1;-6)	6297.7 (6001.6;6588.6)	6285.3 (5998.3;6582.1)	-0.2 (2.8;-3)
Mato Grosso do Sul	6168.4 (5915.1;6427.7)	5964.9 (5727.2;6210.6)	-3.3 (-1.3;-5.3)	5730.5 (5467.9;5987.4)	5472.6 (5219.8;5732.1)	-4.5 (-1.6;-7.4)	6571.7 (6308.5;6860.6)	6512.2 (6248.7;6790.3)	-0.9 (1.8;-3.7)
Minas Gerais	6269 (5985.9;6552.4)	6031.6 (5768.1;6308.7)	-3.8 (-1.4;-5.9)	5902 (5602;6201.4)	5614.5 (5333.5;5907.9)	-4.9 (-1.8;-7.9)	6699.1 (6401.3;6989.1)	6532.8 (6246.7;6839)	-2.5 (0.2;-5)
Pará	5842.4 (5588.6;6111.7)	5751 (5495.1;6017.5)	-1.6 (0.6;-3.6)	5511.2 (5245.2;5786.3)	5291.5 (5022.7;5567.5)	-4 (-0.7;-7.2)	6191.5 (5918.1;6485.6)	6236.9 (5947.8;6540.6)	0.7 (3.7;-2)
Paraíba	5802.6 (5546.1;6053.4)	5755.9 (5515.6;6004.5)	-0.8 (1.4;-3)	5477.7 (5212.5;5728.9)	5371 (5114.4;5651.9)	-1.9 (1.3;-5)	6170.5 (5892.1;6456.2)	6253.3 (5978.4;6539.4)	1.3 (4.1;-1.5)
Paraná	6350.3 (6083.3;6621.1)	5998 (5747.6;6250.1)	-5.5 (-3.5;-7.6)	5947.5 (5671;6238.5)	5549.2 (5289.1;5808)	-6.7 (-3.8;-9.5)	6765.8 (6472.5;7054)	6538.5 (6262.5;6813.3)	-3.4 (-0.5;-6)
Pernambuco	5864.3 (5618.3;6114.8)	5642.3 (5399.5;5887.5)	-3.8 (-1.7;-5.9)	5544.4 (5296;5814.4)	5239.5 (4986.3;5490.8)	-5.5 (-2.5;-8.3)	6252.9 (5972.2;6545.7)	6184.6 (5912.5;6459.7)	-1.1 (1.7;-3.9)
Piauí	5511.3 (5276.3;5755.1)	5545.1 (5314.7;5786.3)	0.6 (2.8;-1.4)	5145.2 (4900.4;5394.3)	5131.4 (4894.1;5385.3)	-0.3 (2.8;-3.3)	5911.2 (5642.8;6176.5)	6034.4 (5776.8;6317.2)	2.1 (4.8;-0.6)
Rio de Janeiro	6714.2 (6446.8;7008.6)	6230.8 (5980.5;6490.7)	-7.2 (-5.3;-9.2)	6350.5 (6067.4;6659.1)	5820 (5546.8;6108.8)	-8.4 (-5.6;-11.1)	7208.1 (6917.8;7498.2)	6800.2 (6527.3;7073.1)	-5.7 (-3.1;-8.1)
Rio Grande do Norte	5701.2 (5452.1;5951.2)	5672.5 (5438;5939.2)	-0.5 (1.6;-2.4)	5393 (5131.9;5659.9)	5280.7 (5029.7;5550.9)	-2.1 (0.8;-5)	6041.8 (5780.8;6327.3)	6170.4 (5892.3;6466.5)	2.1 (5.1;-0.6)
Rio Grande do Sul	6600.3 (6315.3;6880.9)	6182.1 (5906.1;6460.9)	-6.3 (-4.4;-8.3)	6322.7 (6028.2;6645.2)	5828.4 (5536.3;6116.6)	-7.8 (-4.9;-10.6)	6961.6 (6661.6;7269.6)	6639.1 (6336.7;6946.7)	-4.6 (-2;-7.3)
Rondônia	5985.6 (5731;6240.1)	5705.1 (5457.5;5949.3)	-4.7 (-2.4;-6.8)	5599.4 (5348.2;5868.1)	5285.8 (5041.4;5543.5)	-5.6 (-2.6;-8.6)	6276.9 (5998.5;6564.3)	6111.8 (5837.6;6398.4)	-2.6 (0.4;-5.3)
Roraima	6064.1 (5818.4;6308.8)	5814.1 (5583.7;6060.7)	-4.1 (-2.1;-6)	5617 (5371;5884)	5317.9 (5070;5562.2)	-5.3 (-2.4;-8.1)	6407.7 (6148.3;6694.1)	6269.6 (6021.5;6541.3)	-2.2 (0.4;-4.9)
Santa Catarina	6679.1 (6397.8;6964.3)	6217 (5941.2;6488.4)	-6.9 (-5;-8.8)	6375.3 (6069.7;6674.6)	5844.5 (5564.4;6133.5)	-8.3 (-5.4;-11.1)	7026.3 (6731;7339.3)	6667 (6373.5;6959.6)	-5.1 (-2.3;-7.8)
São Paulo	6801.7 (6517.3;7096.7)	6423.9 (6151.9;6705.8)	-5.6 (-3.4;-7.5)	6406.6 (6114.7;6715.3)	6000.7 (5715;6302.4)	-6.3 (-3.1;-9.3)	7284.4 (6975.5;7612.3)	6975.7 (6661.7;7268.7)	-4.2 (-1.4;-6.8)
Sergipe	5922.8 (5667.3;6171.5)	5851.4 (5597.1;6110.4)	-1.2 (1.2;-3.4)	5582.1 (5328.1;5864.7)	5442.8 (5194.8;5717)	-2.5 (0.7;-5.3)	6324.1 (6036.1;6611.2)	6374.1 (6091.1;6662.7)	0.8 (3.9;-1.8)
Tocantins	5849.3 (5606.1;6103.5)	5849.2 (5606.6;6094.9)	0 (2.1;-2.1)	5387.1 (5153.3;5644.6)	5307.1 (5065.3;5571.5)	-1.5 (1.5;-4.3)	6261.9 (5993.8;6548.1)	6368.8 (6094;6643.8)	1.7 (4.6;-1)

*Source: Global Burden of Disease Study 2017, Institute for Health Metrics and Evaluation.^109^*

**Table 2-5:** – Number of DALYs, age-standardized DALY rates (per 100 000) and percent change of rates for stroke and stroke subtypes, in Brazil and its Federative Units, 1990 and 2017

Cause of death and location	1990		2017		Percent change (95% UI)
	Number (95% UI)	Rate (95% UI)	Number (95% UI)	Rate (95% UI)	
***B.2.3- Stroke***					
Acre	3953.6 (3783.3;4122.2)	1979.4 (1889.4;2059)	7266.7 (6834.2;7705)	1186.3 (1115.8;1257.3)	-40.1 (-43.8;-36.1)
Alagoas	43233.8 (40973.6;45710.8)	2817.5 (2696.9;2939.7)	50039.4 (47688.1;52560.1)	1603.7 (1528;1685.4)	-43.1 (-46.4;-39.8)
Amapá	1920.5 (1817.3;2003.4)	1670.5 (1572;1744.3)	6037.8 (5631.2;6387.6)	1177.4 (1098.1;1246.7)	-29.5 (-33.8;-25.2)
Amazonas	17676.9 (16548.2;18641.9)	1941.3 (1817.2;2042.1)	31700.8 (29773.6;33426.1)	1140 (1076.7;1203.3)	-41.3 (-44.6;-37.3)
Bahia	156852.9 (148273.2;166581.9)	2060 (1952.1;2187.7)	195170.9 (186401.1;204704.4)	1235.6 (1179.1;1295.2)	-40 (-43.7;-36.1)
Brazil	2448379.5 (2393632.7;2503724.8)	2511.9 (2457.3;2567.6)	2594661.6 (2510573.1;2684848.2)	1145.3 (1107.8;1185.3)	-54.4 (-55.5;-53.2)
Ceará	77492.6 (71681.9;83089.4)	1703.7 (1591.4;1819.1)	103566.8 (98110.9;108379.4)	1057.1 (1000.6;1107)	-38 (-42.2;-33.4)
Distrito Federal	16605 (15926.8;17311.1)	2251.3 (2165.6;2333.6)	22403.1 (21031.9;23843.1)	920.1 (864.6;978.9)	-59.1 (-61.8;-56.6)
Espírito Santo	48506.9 (47021.8;49978.7)	3107.9 (3018.3;3201)	46467.9 (44131.5;48868.8)	1096.7 (1041.8;1153.2)	-64.7 (-66.5;-62.8)
Goiás	53132.8 (51019.6;55603.8)	2378.7 (2290.8;2484.2)	67110 (63427;71058)	992.6 (937.7;1051.6)	-58.3 (-60.4;-56.1)
Maranhão	75263.4 (68906.4;82104.3)	2388.4 (2215.7;2565.2)	91064 (85853.5;96952.9)	1402.6 (1322.8;1493.3)	-41.3 (-46;-36.2)
Mato Grosso	18394.3 (17193.1;19706.7)	1921.2 (1795;2051.7)	33309.4 (31413.2;35286.5)	1053.1 (994.2;1114.2)	-45.2 (-49.3;-40.3)
Mato Grosso do Sul	24999.9 (23740.6;26097.7)	2462.2 (2369.6;2564.2)	34030.3 (32246.1;35981.7)	1208.7 (1146.7;1277.8)	-50.9 (-53.7;-48)
Minas Gerais	285548.6 (275818.9;297328.5)	2638.6 (2555.4;2738.7)	271355.2 (257504.2;284521)	1075.3 (1020.5;1128.8)	-59.2 (-61.3;-57)
Pará	53548.4 (50133.8;56655)	2253.4 (2113.7;2377)	88611.2 (83452.1;93804)	1314.4 (1238.6;1389.9)	-41.7 (-45.7;-37.3)
Paraíba	45144.4 (42515.4;47988.9)	1858.6 (1751.2;1976.6)	54910.4 (50876.5;59063.6)	1206.4 (1118.5;1299.4)	-35.1 (-40.9;-29.1)
Paraná	161625.7 (156492.6;166651.6)	3179.7 (3077;3274.8)	151291.7 (143947.2;158892.6)	1197.3 (1140.6;1255.3)	-62.3 (-64.2;-60.3)
Pernambuco	130872.4 (125974.8;135596)	2751.6 (2651.9;2849.7)	136137.9 (129804.7;142784.5)	1375 (1311.6;1441)	-50 (-52.3;-47.6)
Piauí	34432.1 (31750.1;37132.4)	2154.9 (1999.1;2316.3)	44933.5 (42457;48250.5)	1250.2 (1181.4;1343.6)	-42 (-46.3;-37.4)
Rio de Janeiro	347453.9 (334592.7;357975.7)	3346.4 (3230.9;3445.4)	273760.5 (261736;286898.1)	1271.2 (1214.1;1331.4)	-62 (-63.9;-60.1)
Rio Grande do Norte	25680.8 (24130.7;27242.4)	1487.2 (1395.3;1582.5)	34501.8 (32491.3;36566.5)	927.5 (872.6;983.7)	-37.6 (-42.5;-32.5)
Rio Grande do Sul	172339.3 (165146.5;178677.8)	2524.5 (2421.8;2615.5)	170330.5 (160060.7;179998.5)	1150.9 (1082.3;1214.1)	-54.4 (-56.8;-52)
Rondônia	12073.7 (11218.4;12924.2)	2741.2 (2563.3;2929.9)	17322.9 (15670.4;19262)	1178.2 (1069.9;1308.7)	-57 (-61.6;-51.8)
Roraima	1569.1 (1426.3;1728.5)	2172.9 (2005;2364.6)	3644.2 (3249.6;4101.8)	1057.5 (946.3;1182.6)	-51.3 (-57.5;-44.5)
Santa Catarina	69910.9 (67055.5;72514.6)	2666.7 (2559.3;2763.6)	71975.6 (67852.1;76213.3)	950.5 (898.1;1006.1)	-64.4 (-66.2;-62.4)
São Paulo	539524.3 (519316.5;557948.6)	2449 (2360.9;2532.4)	542703.5 (515509.8;569546.5)	1043.5 (993.5;1094.6)	-57.4 (-59.5;-55.2)
Sergipe	20033.1 (19174.8;20849.7)	2166.3 (2075.3;2253.8)	27552 (26196.3;28951.2)	1282.7 (1220.2;1347.5)	-40.8 (-44;-37.2)
Tocantins	10590 (8966.2;11793.5)	2245 (1990.4;2461.4)	17463.5 (16283.5;18745.7)	1235.7 (1153.4;1326.5)	-45 (-50.5;-37.6)
***B.2.3.1- Ischemic stroke***					
Acre	1063.5 (996.8;1132.3)	667.8 (626.7;707.7)	2076 (1893.9;2264.6)	383.1 (351;417.7)	-42.6 (-47.4;-37.9)
Alagoas	13197.8 (12462.3;14030.4)	985.2 (929.6;1045.1)	15974 (14833.8;17114.8)	542.2 (503.3;580.4)	-45 (-48.8;-40.7)
Amapá	587.7 (545;627.3)	640.8 (595.3;680.4)	1748.6 (1589.7;1898.5)	402.8 (368.8;435.9)	-37.1 (-41.9;-32.4)
Amazonas	5084.6 (4695.8;5429.5)	688.6 (637.7;735)	9130.4 (8393.2;9894.1)	375.1 (345.4;405.2)	-45.5 (-49.4;-41)
Bahia	46074.5 (43055.7;49389.4)	671.6 (627.8;719.8)	60202.2 (56130.2;64836.4)	388.2 (361.5;418.2)	-42.2 (-46.8;-37.5)
Brazil	731917.9 (704484.3;760431.9)	871.4 (841.1;902.4)	846622.4 (794644.5;900331.8)	387.3 (363.8;411.5)	-55.6 (-57.3;-53.8)
Ceará	22421 (20485.5;24419.3)	546.3 (497.9;596.2)	35205.6 (32642.9;37754.2)	361.7 (335.2;388.2)	-33.8 (-40.2;-27.1)
Distrito Federal	3906.4 (3644.7;4163.9)	766.5 (721.7;813)	6593.1 (5992;7230)	325.9 (298.5;356.9)	-57.5 (-61.1;-53.7)
Espírito Santo	14282 (13533.3;15095.1)	1098.2 (1045.3;1153.6)	14731.1 (13585.4;15935.6)	363.2 (335.4;393.2)	-66.9 (-69.2;-64.5)
Goiás	13916.2 (12991.6;14820.9)	800.2 (752;844.9)	19898.4 (18199.2;21777)	320.9 (293.9;350)	-59.9 (-62.8;-56.7)
Maranhão	17377 (15585.8;19607.3)	660 (586.2;752.6)	27215.8 (24986.8;29637.2)	439.9 (404.1;479)	-33.3 (-40;-26)
Mato Grosso	5212.7 (4803.6;5633.7)	705.7 (650.9;759.9)	10002 (9113.3;10867.5)	356.6 (326.3;386.9)	-49.5 (-54.1;-44.5)
Mato Grosso do Sul	6723.6 (6322.8;7131.6)	813.8 (767.3;859.5)	10101.1 (9292.6;10911.3)	384.6 (354.4;414.8)	-52.7 (-56.5;-48.9)
Minas Gerais	81390.3 (76723.4;86023.9)	881.5 (836.4;927.8)	86497.3 (79441.7;93590)	346.9 (318.9;375.4)	-60.6 (-63.5;-57.6)
Pará	18296.6 (16994.5;19445.1)	906.6 (843.3;962.1)	28488.5 (26185.1;30787)	465.3 (428.2;501.5)	-48.7 (-53;-44.3)
Paraíba	14128.8 (12998.2;15518.4)	609.2 (560.5;666.9)	18006.3 (16468.6;19631.7)	392.4 (358.4;427.9)	-35.6 (-42.9;-27.8)
Paraná	51172.4 (48539.5;53822.5)	1204 (1146.1;1259.9)	55096.3 (51203.8;59213.8)	455.8 (424.5;489.8)	-62.1 (-64.8;-59.4)
Pernambuco	41435.4 (39396.4;43702.5)	970.5 (925.1;1020.6)	41014.9 (38043.8;44085.7)	431.5 (400.1;463.9)	-55.5 (-58.6;-52.1)
Piauí	9568.2 (8668;10635.1)	687.6 (623.1;765.2)	14673.1 (13540.8;15959.7)	411.7 (379.6;448)	-40.1 (-45.6;-34)
Rio de Janeiro	95808.8 (90420.4;100946.9)	1069 (1012.6;1122.6)	83858 (77688.3;90710.3)	394.7 (366.4;426.8)	-63.1 (-65.9;-60.3)
Rio Grande do Norte	9503.9 (8745.6;10253.4)	584.6 (538.8;631.1)	11733.2 (10776.4;12756)	319.1 (292.9;346.8)	-45.4 (-50.7;-39.9)
Rio Grande do Sul	60826.8 (57517.2;64097.9)	1027.2 (972.9;1078)	61657.7 (56558.6;66642)	417.5 (382.7;451.1)	-59.4 (-62.2;-56.3)
Rondônia	3034.7 (2783.7;3293.1)	970.7 (899.5;1042.4)	5412.4 (4855.6;6020.7)	415.3 (373.7;461)	-57.2 (-61.8;-51.9)
Roraima	372.6 (336.5;412.4)	795.4 (729;867.3)	1055.5 (934.1;1183.6)	379.8 (337.7;421.7)	-52.2 (-58.4;-45.5)
Santa Catarina	22415.1 (21207.6;23730.7)	1029.7 (974;1083.9)	25161.9 (23015.1;27290.2)	354.4 (325.4;383.2)	-65.6 (-68;-62.8)
São Paulo	164767.5 (155579.3;173984.4)	890.4 (843.4;938)	186699.6 (171816.4;201713.6)	372 (342.1;401.3)	-58.2 (-61;-55.2)
Sergipe	6658.2 (6265.3;7056.5)	781.8 (736.7;828.1)	8987.9 (8329;9671)	443.8 (411.4;476.9)	-43.2 (-47.3;-38.6)
Tocantins	2692 (2368.4;3009.9)	750 (674.7;828.6)	5401.9 (4954.1;5885.6)	408.6 (374.8;444.9)	-45.5 (-51.5;-38.7)
***B.2.3.2- Intracerebral hemorrhage***					
Acre	2042.4 (1946.7;2151.3)	1021.1 (972.1;1070.2)	3907 (3628.3;4180.9)	625.6 (581;667.5)	-38.7 (-43.7;-33.5)
Alagoas	22644.7 (21077;24235.6)	1476.9 (1388.9;1567.2)	27097.9 (25636;28599.9)	855.7 (809.8;904.3)	-42.1 (-46.4;-37.8)
Amapá	1000.2 (936.5;1047.5)	829.7 (774.6;869.6)	3256.8 (2973.7;3497.4)	613.2 (561;657.1)	-26.1 (-31.8;-19.8)
Amazonas	9657.5 (8944.9;10226.1)	1033.8 (957;1093.8)	17298.4 (16028.8;18370.1)	607.4 (564.8;644.9)	-41.2 (-45.5;-36.3)
Bahia	84377.2 (79355.7;90141)	1108.1 (1040.1;1183.5)	104105.8 (98385.8;110053.4)	656.6 (620.6;694)	-40.7 (-45.3;-35.8)
Brazil	1334369 (1303152.8;1373801.4)	1322.1 (1291.8;1358.2)	1326910.8 (1288958.3;1368112.2)	576.9 (560.7;594.9)	-56.4 (-57.8;-55.1)
Ceará	40070.2 (36879.4;43154.2)	893.2 (817;960.7)	52333.9 (49236;55146.4)	533.9 (501.6;562.5)	-40.2 (-45.3;-34.6)
Distrito Federal	9370.1 (8908.1;10046.1)	1179 (1125.2;1252.5)	11358.7 (10528;12284.1)	443.7 (413.3;477.7)	-62.4 (-65;-59.3)
Espírito Santo	27092.1 (26043.2;28443.7)	1651.2 (1587.7;1725.3)	23985.1 (22605.5;25435.7)	557 (525.7;591.2)	-66.3 (-68.3;-64.1)
Goiás	30618.2 (29152.7;32584.3)	1291.1 (1232.1;1366.3)	35332.4 (33203.1;37574)	509.5 (478.7;541.7)	-60.5 (-63;-57.8)
Maranhão	40946.2 (36530.2;45483)	1334.4 (1213.3;1456.3)	48119.1 (44681.6;52607.1)	738.8 (686.2;810.1)	-44.6 (-50.9;-37.9)
Mato Grosso	9481.7 (8758.9;10235.2)	952.6 (879.1;1027.3)	17317.5 (16178.9;18505.5)	529.8 (495.5;566.5)	-44.4 (-49.2;-38.3)
Mato Grosso do Sul	14326.7 (13580.5;15036)	1346.6 (1285.1;1408.5)	18396.2 (17315.1;19515.4)	639.5 (601.6;678.8)	-52.5 (-55.9;-49.1)
Minas Gerais	160746.7 (153946.9;171975.7)	1422.7 (1364.4;1509)	139119.5 (131593;146724)	546.2 (516.9;575.9)	-61.6 (-64.2;-58.9)
Pará	27317.6 (25130.3;29082.4)	1111.4 (1017.5;1182.7)	46066.2 (42810.4;49272.9)	668.3 (622.2;712.9)	-39.9 (-45;-34.3)
Paraíba	23051.3 (21538.9;24766)	954 (892.6;1024.9)	28591.1 (26253.3;30997.8)	629.8 (578.8;682.3)	-34 (-40.8;-26.8)
Paraná	88216.4 (84443;91983)	1633.9 (1570.7;1702)	72564.6 (68483;76843.2)	561.1 (530.1;593.1)	-65.7 (-67.9;-63.3)
Pernambuco	73380.8 (69895.3;76702.7)	1499.7 (1430.9;1568.9)	74615.6 (70543.1;78768.3)	744.4 (703.9;786.5)	-50.4 (-53.4;-47.5)
Piauí	18501.1 (16948.8;20251.5)	1160.3 (1060.5;1264)	23443.1 (22029.8;25610)	651 (611.5;710.8)	-43.9 (-48.8;-38.6)
Rio de Janeiro	201658.8 (193417.1;211873.7)	1858 (1782.2;1943.7)	147613.4 (140085.6;156033.4)	676.3 (641.5;713.3)	-63.6 (-66;-61.1)
Rio Grande do Norte	12099.4 (11196.2;12951.9)	706.5 (651.7;757.1)	17465.8 (16141.3;18793.4)	468.6 (433.9;504.3)	-33.7 (-39.7;-26.6)
Rio Grande do Sul	90116 (85634.7;94313.2)	1235.2 (1173.1;1292.4)	85329.9 (79196.9;90723.5)	570.3 (529.1;605.5)	-53.8 (-56.9;-50.3)
Rondônia	6920.4 (6371.4;7465.9)	1469.9 (1361.9;1583.8)	8998.9 (8019.1;10137.8)	592.7 (529.6;666.4)	-59.7 (-64.6;-53.9)
Roraima	868.7 (786;962.9)	1116.5 (1020.8;1223.5)	1914.8 (1686.6;2190.9)	527.6 (464.5;599.3)	-52.7 (-59.7;-45.1)
Santa Catarina	37531.6 (35812;39073.7)	1347.1 (1285.7;1402.4)	35160.6 (32444.1;37333.3)	451.6 (418.2;478.8)	-66.5 (-68.8;-64.1)
São Paulo	286216.8 (273648.3;299941.9)	1235.3 (1182.4;1289)	259904.1 (245650.4;275069.5)	490.6 (464.5;518.7)	-60.3 (-62.8;-57.4)
Sergipe	10290.6 (9777.3;10791)	1120.9 (1062.3;1176.2)	14532.9 (13755.7;15333.1)	666.3 (631.3;703.4)	-40.6 (-44.7;-35.7)
Tocantins	5825.5 (4890.2;6575)	1189.3 (1037.1;1325.3)	9081.5 (8410.7;9866.8)	633.5 (587.1;688.8)	-46.7 (-52.9;-38.2)
***B.2.3.3- Subarachnoid hemorrhage***					
Acre	847.7 (773.2;919.3)	290.6 (269.9;311.1)	1283.8 (1183.9;1391)	177.6 (164.1;193.1)	-38.9 (-44.9;-32.4)
Alagoas	7391.3 (6373.7;8529.4)	355.3 (311.7;397.2)	6967.4 (6414.6;7757.1)	205.9 (189.4;228.9)	-42.1 (-50.3;-29.8)
Amapá	332.7 (309.9;365)	200.1 (186;223.5)	1032.5 (948.2;1149)	161.5 (148.4;179.7)	-19.3 (-26.7;-10.9)
Amazonas	2934.8 (2699.5;3205.7)	218.9 (203.2;238.9)	5271.9 (4852.8;5795.8)	157.6 (145.2;173.4)	-28 (-34.4;-20.6)
Bahia	26401.2 (23947.3;28838.7)	280.2 (256.7;306.5)	30862.9 (28723;33700.7)	190.8 (177.6;208.7)	-31.9 (-38.9;-24.6)
Brazil	382092.5 (341738.8;399795.1)	318.4 (287;332.2)	421128.4 (402202.4;445263.2)	181 (173.1;191)	-43.1 (-46.3;-37.2)
Ceará	15001.4 (12391.2;18119.3)	264.2 (225.1;311.9)	16027.3 (14761.6;17474.1)	161.5 (148.7;176)	-38.9 (-49.2;-28.2)
Distrito Federal	3328.5 (2921.2;3564.8)	305.8 (267.9;326.7)	4451.2 (3996.2;4855.6)	150.5 (134.4;164.3)	-50.8 (-55.3;-45.2)
Espírito Santo	7132.8 (5899.9;7604.3)	358.5 (294.5;382.4)	7751.8 (7128.6;8367.7)	176.5 (162.5;190)	-50.8 (-55.4;-39.5)
Goiás	8598.4 (7989.3;9184.5)	287.4 (265.6;307)	11879.3 (10974.1;13114.1)	162.2 (150.1;178.6)	-43.6 (-48.6;-36.8)
Maranhão	16940.2 (13560.2;20200.9)	394 (321;459.7)	15729.1 (14113.5;17089.8)	223.9 (199;243.2)	-43.2 (-50.9;-32.3)
Mato Grosso	3699.9 (3386.4;4012)	262.9 (241.4;287.3)	5989.9 (5522;6551.1)	166.7 (154.3;182)	-36.6 (-43.1;-29.6)
Mato Grosso do Sul	3949.6 (3524;4259.3)	301.8 (272.8;324.2)	5533 (5124.3;6033)	184.7 (171.2;201.1)	-38.8 (-45.1;-31.2)
Minas Gerais	43411.7 (36926.5;46441.3)	334.4 (286;356.7)	45738.5 (42358.9;49050.9)	182.2 (169;195.2)	-45.5 (-50.4;-37.9)
Pará	7934.2 (7204.8;8740.1)	235.4 (216.8;259.9)	14056.5 (12883.9;15453.7)	180.9 (166.2;198.4)	-23.2 (-30.1;-15.8)
Paraíba	7964.3 (6876.5;9209)	295.5 (255.4;339.2)	8313 (7521.9;9221.7)	184.2 (166.7;204.3)	-37.7 (-48.2;-25.5)
Paraná	22236.9 (18814.4;23684.3)	341.8 (287.1;364.5)	23630.8 (21760.6;25484.3)	180.4 (166.5;194)	-47.2 (-52.1;-39.6)
Pernambuco	16056.3 (14930.2;17673.3)	281.4 (262.2;311.8)	20507.5 (19042.9;22982.6)	199.2 (185;222.9)	-29.2 (-35.8;-22.5)
Piauí	6362.7 (5250.5;7430)	307 (258.7;356.2)	6817.4 (6299.7;7358.7)	187.4 (173.4;202.5)	-39 (-47.3;-28.2)
Rio de Janeiro	49986.3 (39896.5;53598.5)	419.4 (335.1;449.1)	42289.2 (38909;45633.2)	200.1 (184.6;215.2)	-52.3 (-56.7;-41.4)
Rio Grande do Norte	4077.6 (3612.7;4762.8)	196.1 (174.2;236.3)	5302.8 (4835.1;6667.4)	139.8 (127.7;175.3)	-28.7 (-37.7;-18.9)
Rio Grande do Sul	21396.6 (19895.4;23508.5)	262.1 (244.7;290.7)	23342.9 (21439;25660.3)	163.1 (150.5;178.8)	-37.8 (-43.1;-31.4)
Rondônia	2118.6 (1933.7;2303)	300.6 (275.8;327.4)	2911.6 (2578.8;3318.4)	170.2 (151;193.1)	-43.4 (-51;-34.1)
Roraima	327.8 (294;368.6)	261.1 (234.8;298.9)	673.8 (592.6;805.6)	150.1 (132.2;178.6)	-42.5 (-50.8;-32.5)
Santa Catarina	9964.3 (9246.9;10542.8)	289.9 (267.1;306.6)	11653.1 (10732.6;13019.9)	144.5 (133.5;160.9)	-50.2 (-54.8;-43)
São Paulo	88540 (77303.1;94399.5)	323.3 (286.2;343.1)	96099.9 (89106.8;103155.3)	181 (168.3;194.1)	-44 (-49.1;-36.7)
Sergipe	3084.4 (2757.9;3439.9)	263.6 (241.5;290.9)	4031.2 (3725.2;4445.7)	172.6 (159.7;190.2)	-34.5 (-42.1;-26.6)
Tocantins	2072.4 (1546;2522.9)	305.7 (242.4;363.9)	2980.1 (2699;3279)	193.6 (175.8;212.5)	-36.7 (-47.4;-21.4)

*Source: Global Burden of Disease Study 2017, Institute for Health Metrics and Evaluation.^109^*

**Table 2-4:** – Age-standardized mortality rates (per 100 000) for all strokes, ischemic stroke, intracerebral hemorrhage, and subarachnoid hemorrhage, in Brazil, 1990 and 2017, and percent change

Cause of death and age group	1990	2017	Percent change (95% UI)
B.2.3 - Stroke			
15-49 years	17.8 (17.3;18.3)	8.3 (8.1;8.6)	-53.4 (-55.2;-51.6)
50-69 years	215.8 (210.8;220.9)	95 (92.4;97.6)	-56 (-57.5;-54.5)
5-14 years	0.9 (0.8;1)	0.4 (0.3;0.4)	-57.4 (-61.8;-51.7)
70+ years	1145.5 (1119.4;1166)	639.5 (622.2;655.6)	-44.2 (-45.6;-42.7)
Age-standardized	122.9 (120.6;125)	56.6 (55.2;57.8)	-54 (-55.1;-53)
All Ages	65 (63.8;66.1)	58 (56.6;59.2)	-10.8 (-13;-8.8)
Under 5	4.3 (3.5;5.4)	0.7 (0.6;0.8)	-84.1 (-88.7;-78)
***B.2.3.1 - Ischemic stroke***			
15-49 years	2.2 (2.1;2.4)	0.9 (0.8;0.9)	-59.7 (-64.2;-56.7)
50-69 years	58 (56;60.2)	21.1 (20.1;21.9)	-63.7 (-65.8;-61.6)
5-14 years	0.1 (0;0.1)	0 (0;0)	-71.6 (-76.9;-65.9)
70+ years	618.1 (603.3;632.5)	319.7 (309.7;328.7)	-48.3 (-49.9;-46.5)
Age-standardized	54.8 (53.6;55.9)	22.6 (21.9;23.2)	-58.7 (-60;-57.4)
All Ages	24.8 (24.2;25.4)	22.4 (21.7;23)	-9.6 (-12.6;-6.8)
Under 5	0.2 (0.2;0.3)	0 (0;0)	-89.7 (-94.3;-82.2)
***B.2.3.2 - Intracerebral hemorrhage***			
15-49 years	11.2 (10.9;11.9)	4.8 (4.5;5)	-57.6 (-60.9;-55.4)
50-69 years	133.4 (129.6;137.1)	58.3 (56.4;60.3)	-56.3 (-58;-54.5)
5-14 years	0.3 (0.2;0.3)	0.1 (0.1;0.1)	-59.6 (-66.9;-51.7)
70+ years	482.2 (468.2;494.2)	281 (272.2;289.9)	-41.7 (-43.8;-39.6)
Age-standardized	58.6 (57.3;59.9)	27.9 (27.1;28.7)	-52.4 (-53.8;-51.1)
All Ages	33.6 (32.9;34.4)	29 (28.3;29.9)	-13.6 (-16.2;-11.1)
Under 5	1 (0.6;1.6)	0.1 (0.1;0.1)	-89 (-94.1;-79.7)
***B.2.3.3 - Subarachnoid hemorrhage***			
15-49 years	4.4 (3.8;4.5)	2.6 (2.5;2.9)	-39.4 (-43.6;-29.4)
50-69 years	24.4 (22;25.6)	15.6 (14.8;16.6)	-36 (-40.2;-29.6)
5-14 years	0.6 (0.5;0.6)	0.3 (0.2;0.3)	-54.9 (-59.2;-47.8)
70+ years	45.3 (43.2;49.1)	38.8 (35.8;41)	-14.2 (-22.7;-8.1)
Age-standardized	9.6 (8.8;9.9)	6.1 (5.8;6.4)	-36.5 (-39.8;-32.2)
All Ages	6.6 (6;6.8)	6.5 (6.2;6.9)	-0.7 (-6;7.4)
Under 5	3 (2.4;3.6)	0.5 (0.5;0.6)	-82 (-86.4;-74.1)

*Source: Global Burden of Disease Study 2017, Institute for Health Metrics and Evaluation.^109^*

**Table 2-10:** – Age-standardized DALY rates (per 100 000) for stroke and stroke subtypes, for women, in Brazil, 1990 and 2017, and percent change

Cause of death and age group	1990	2017	Percent change (95% UI)
***B.2.3- Stroke***			
15-49 years	817.7 (790.3;848.7)	421.7 (400.5;440)	-48.4 (-51.1;-45.8)
50-69 years	5185 (4992.7;5389.2)	2392.8 (2276.5;2511)	-53.9 (-56;-51.8)
5-14 years	69.9 (60.4;76.3)	32.7 (29.4;36.3)	-53.2 (-57.8;-47.5)
70+ years	13838.3 (13364.1;14288.7)	7055.4 (6735.7;7403.1)	-49 (-51;-46.9)
Age-standardized	2162.9 (2095.1;2225.5)	990.7 (947.7;1034.7)	-54.2 (-55.9;-52.7)
All Ages	1466.3 (1419.5;1509.1)	1134.6 (1085.5;1185.3)	-22.6 (-25.6;-20)
Under 5	337.3 (257.9;439)	49.3 (41.5;60.4)	-85.4 (-89.9;-78.1)
***B.2.3.1- Ischemic stroke***			
15-49 years	104.9 (97.4;113.8)	55.1 (48.1;62.3)	-47.5 (-53.4;-42.3)
50-69 years	1382.3 (1287.6;1474.6)	571.4 (514.2;629.2)	-58.7 (-61.7;-55.6)
5-14 years	7.5 (6.2;9.1)	4.3 (3.2;5.5)	-43 (-49.6;-36.6)
70+ years	7472.4 (7131.5;7787.8)	3643.1 (3416.1;3892)	-51.2 (-53.8;-48.8)
Age-standardized	747.7 (713.7;780.1)	326.3 (302.2;351.8)	-56.4 (-58.8;-54.1)
All Ages	439.5 (418.8;460.3)	371.1 (343.5;400.1)	-15.5 (-20.3;-11.3)
Under 5	18.4 (13;29.6)	2.4 (1.9;3)	-87 (-93.1;-79.5)
***B.2.3.2- Intracerebral hemorrhage***			
15-49 years	460.3 (440.4;483.1)	207 (194.5;220.2)	-55 (-58.2;-51.4)
50-69 years	2968.9 (2849.9;3089.3)	1272.5 (1215.6;1335.2)	-57.1 (-59.8;-54.7)
5-14 years	20.3 (17.3;22.7)	9.4 (8.1;10.8)	-53.5 (-60.8;-45.8)
70+ years	5659.7 (5417.6;5890.7)	2879.7 (2743.7;3027.5)	-49.1 (-52;-46.1)
Age-standardized	1070.2 (1035.3;1106.6)	465.2 (445;485.9)	-56.5 (-58.7;-54.5)
All Ages	746.6 (722;774)	535.7 (512.6;559.7)	-28.2 (-31.8;-24.9)
Under 5	69.4 (45.1;118.1)	7.9 (5.9;10.3)	-88.6 (-94.6;-79.9)
***B.2.3.3- Subarachnoid hemorrhage***			
15-49 years	252.5 (238.8;265.9)	159.6 (149.6;169.8)	-36.8 (-41.6;-31.8)
50-69 years	833.8 (780.9;891.9)	548.9 (513;588.7)	-34.2 (-39.3;-29.1)
5-14 years	42.1 (35.8;46.5)	19 (17;21.3)	-54.8 (-59.7;-48.2)
70+ years	706.2 (661.2;794.7)	532.6 (494.2;573.3)	-24.6 (-32.3;-18.3)
Age-standardized	345.1 (324.7;363.2)	199.2 (187.4;210.1)	-42.3 (-46.1;-39)
All Ages	280.2 (263.7;293.7)	227.8 (214.2;240.8)	-18.7 (-24.1;-13.6)
Under 5	249.5 (190.9;317.3)	39 (32.9;48.1)	-84.4 (-89.1;-77.2)

*Source: Global Burden of Disease Study 2017, Institute for Health Metrics and Evaluation.^109^*

**Table 3:** 1 – Age-standardized prevalence rates (per 100 000 inhabitants) of coronary heart disease in Brazil and its Federative Units, for both sexes, and for males and females, 1990 and 2017, and percent changes of rates

Federative Units	Both sexes			Females			Males		
	1990	2017	Percent change (95% UI)	1990	2017	Percent change (95% UI)	1990	2017	Percent change (95% UI)
Acre	1640.6 (1532.9;1756.6)	1483.4 (1387.7;1581.8)	-9.6 (-6.5;-12.8)	1083.4 (1008.2;1165.6)	923.1 (852.4;993.5)	-14.8 (-10.9;-18.5)	2120.8 (1978.5;2276.6)	2063.9 (1928.2;2201.2)	-2.7 (1.7;-7.1)
Alagoas	1671.2 (1568;1784.7)	1506.8 (1412.5;1611.2)	-9.8 (-6.8;-12.5)	1150.1 (1070.1;1235.1)	969.9 (901.4;1043.3)	-15.7 (-11.8;-19.4)	2251.6 (2099.8;2410.2)	2164.3 (2031.8;2318.6)	-3.9 (0.8;-7.7)
Amapá	1450.7 (1353.6;1551)	1435.3 (1340.1;1536.3)	-1.1 (2.6;-4.4)	991.2 (916;1066.2)	903.8 (837.3;975.3)	-8.8 (-4.6;-13.2)	1927.9 (1798.7;2058.4)	2003 (1862.3;2143.5)	3.9 (8.8;-0.4)
Amazonas	1411.5 (1316.1;1512.7)	1407.8 (1315.6;1504.9)	-0.3 (3.5;-3.7)	956.9 (888.2;1034.4)	879.4 (815.1;947.3)	-8.1 (-4.1;-12.2)	1865.3 (1737.9;2002.7)	1958.7 (1823.7;2096.5)	5 (9.8;0.5)
Bahia	1644.7 (1540.7;1753.3)	1558.9 (1459.5;1659.1)	-5.2 (-2;-8.4)	1132.7 (1049.9;1215.3)	1001 (922;1077.6)	-11.6 (-7.4;-15.8)	2218.2 (2076.7;2364.7)	2223.4 (2085;2372.7)	0.2 (4.8;-3.9)
**Brazil**	**1674.5 (1573.5;1784)**	**1563.7 (1465.6;1669.4)**	**-6.6 (-5.2;-7.8)**	**1159.3 (1082.7;1238.5)**	**1008 (938.1;1080.6)**	**-13.1 (-11.4;-14.7)**	**2259.7 (2126.8;2403.2)**	**2229.4 (2098.2;2372.8)**	**-1.3 (0.4;-2.9)**
Ceará	1455.2 (1356.6;1559.7)	1452 (1350.5;1561.1)	-0.2 (3.2;-3.8)	1009.3 (931.1;1092.2)	938.9 (860.1;1021.8)	-7 (-2.1;-11.6)	1955.1 (1824;2091.9)	2066.7 (1922.7;2221.3)	5.7 (10.6;0.6)
Distrito Federal	1474.8 (1386;1569.3)	1404.4 (1315.5;1499.5)	-4.8 (-1.8;-7.8)	1025.9 (952.7;1100.2)	915.8 (849.4;988.1)	-10.7 (-6.8;-14.6)	2009.9 (1885;2147.1)	2046.6 (1914.8;2184.4)	1.8 (6.4;-2.4)
Espírito Santo	1563.5 (1462.6;1663.4)	1473.9 (1378.5;1573.3)	-5.7 (-2.5;-8.7)	1072.1 (991.1;1153)	942 (867.7;1015.4)	-12.1 (-7.8;-16.1)	2091.3 (1954.6;2229.9)	2094.4 (1960.9;2243.1)	0.1 (4.4;-4)
Goiás	1663.5 (1563.6;1775.3)	1596.3 (1498.4;1700.3)	-4 (-0.5;-7.4)	1115.1 (1037.1;1195.5)	1006.6 (934.1;1083.7)	-9.7 (-5.3;-14)	2184.7 (2051;2333.6)	2249.7 (2117.6;2405.1)	3 (7.9;-1.4)
Maranhão	1604.3 (1501;1714.3)	1491.9 (1391.9;1600.2)	-7 (-3.8;-10.1)	1104.3 (1022.7;1183.9)	949.8 (877.8;1025.4)	-14 (-9.9;-17.8)	2138.3 (1994.2;2294.4)	2095.5 (1953.1;2255)	-2 (2.8;-6.7)
Mato Grosso	1633.2 (1524.5;1746.9)	1528.7 (1428.2;1637.1)	-6.4 (-3.4;-9.5)	1072.6 (997.6;1154.4)	941.6 (873.1;1012.4)	-12.2 (-8.2;-16.2)	2097.9 (1955.3;2247.1)	2099.9 (1963.9;2250.9)	0.1 (4.1;-4.3)
Mato Grosso do Sul	1671.6 (1569.6;1787.9)	1602.8 (1501.1;1711.9)	-4.1 (-0.6;-7.2)	1114.7 (1034.9;1194.8)	1008 (931.9;1089.9)	-9.6 (-5.3;-13.5)	2190.1 (2052.1;2342.3)	2253.6 (2104.9;2408.3)	2.9 (7.8;-1.7)
Minas Gerais	1635 (1533.4;1743.3)	1571.7 (1473.5;1676.6)	-3.9 (-0.9;-6.9)	1130.7 (1054.5;1213.7)	1005 (931.7;1084.4)	-11.1 (-7.2;-15.1)	2209.6 (2062.1;2361.5)	2230 (2092.6;2384.2)	0.9 (4.9;-3.1)
Pará	1494.5 (1393.7;1598.9)	1461.5 (1363.1;1567.8)	-2.2 (1.3;-5.4)	1018.3 (939.5;1099.9)	914.1 (844.1;996.3)	-10.2 (-5.8;-14.6)	1981.7 (1840.9;2123.7)	2031.3 (1893.8;2172.4)	2.5 (7.5;-1.7)
Paraíba	1580.3 (1473.9;1688.4)	1483.4 (1382.1;1589.2)	-6.1 (-3;-9)	1093.7 (1010.1;1178.4)	958 (888.5;1032.7)	-12.4 (-8.2;-16.4)	2137.4 (1994.7;2291.2)	2133.3 (1989.7;2288.1)	-0.2 (4.4;-4.5)
Paraná	1751.9 (1639.3;1864.8)	1612.4 (1508;1723.5)	-8 (-4.5;-10.9)	1193.9 (1111;1282.8)	1033.6 (959.7;1111.8)	-13.4 (-9.4;-17.2)	2327.5 (2172.7;2484.7)	2290.6 (2147.7;2451.6)	-1.6 (3.1;-6)
Pernambuco	1623.1 (1514.3;1734.5)	1523.7 (1426.1;1629.4)	-6.1 (-2.8;-9.1)	1132.6 (1047.6;1214.9)	992.2 (916.5;1067)	-12.4 (-8.1;-16.5)	2210.5 (2065.8;2374.7)	2206.4 (2064.2;2362.1)	-0.2 (4.3;-4.6)
Piauí	1485.8 (1389.6;1590.6)	1446 (1348.2;1549)	-2.7 (0.7;-5.8)	1021.5 (945.7;1100.5)	922.3 (853;992.7)	-9.7 (-5.5;-13.8)	1992.1 (1858.4;2134.9)	2052.7 (1910.4;2199.7)	3 (7.7;-1.2)
Rio de Janeiro	1686.3 (1576;1809)	1572.3 (1471.2;1676.3)	-6.8 (-3.7;-10)	1182.7 (1093.6;1286.2)	1027.8 (948.9;1110.8)	-13.1 (-9;-17.2)	2341.8 (2186.6;2513.1)	2288.8 (2129;2438.1)	-2.3 (2.1;-6.5)
Rio Grande do Norte	1458.3 (1361.9;1562.6)	1471.1 (1371.1;1572.6)	0.9 (4.4;-2.6)	1011.9 (935.3;1092.3)	953.8 (876.8;1029.9)	-5.7 (-1.2;-10.1)	1954.9 (1823.4;2096.9)	2097.1 (1956.4;2244.8)	7.3 (12.2;2.3)
Rio Grande do Sul	1793.9 (1676.8;1914.8)	1641.5 (1532.1;1756.2)	-8.5 (-5.4;-11.4)	1254.7 (1160.7;1358.6)	1064.5 (981.2;1149.1)	-15.2 (-11.1;-19.1)	2457.9 (2294.2;2621.4)	2349.8 (2195.1;2513.4)	-4.4 (-0.1;-8.3)
Rondônia	1648.7 (1539.6;1757.9)	1485.5 (1387.3;1588.9)	-9.9 (-6.6;-13.3)	1072.2 (993.2;1153.8)	914.5 (842.3;990.6)	-14.7 (-10.6;-18.4)	2101 (1957.3;2246.4)	2041.5 (1905;2179.1)	-2.8 (1.8;-7.4)
Roraima	1583.8 (1480.2;1691.3)	1418.8 (1330.3;1512.8)	-10.4 (-7.4;-13.5)	1021.1 (948.7;1092.6)	862.9 (798.2;925.9)	-15.5 (-11.6;-19.1)	2010.7 (1870.7;2154)	1935.6 (1810.6;2075)	-3.7 (0.5;-7.9)
Santa Catarina	1770.3 (1655.4;1892.5)	1651.5 (1547.2;1753.9)	-6.7 (-3.5;-9.5)	1219 (1129.9;1317.9)	1054.9 (977.2;1138.7)	-13.5 (-9.3;-17.2)	2383.9 (2230.9;2553.1)	2346.6 (2193.4;2503.1)	-1.6 (2.6;-5.3)
São Paulo	1802.7 (1689.6;1922.7)	1617.6 (1515.9;1730.3)	-10.3 (-7.3;-13)	1251.7 (1162.4;1346.3)	1051.7 (973.2;1132.7)	-16 (-11.9;-19.8)	2455.1 (2298.6;2620)	2324 (2176.2;2488.7)	-5.3 (-1.1;-9.1)
Sergipe	1497.6 (1400.5;1600.2)	1419.8 (1327.5;1520.9)	-5.2 (-1.8;-8.3)	1041.2 (966.2;1120.6)	918.5 (844;988.3)	-11.8 (-7.3;-16.1)	2024.6 (1889.5;2175)	2038.2 (1905.1;2188.1)	0.7 (5.2;-3.5)
Tocantins	1621 (1515.9;1725.6)	1553.5 (1450;1661.8)	-4.2 (-0.7;-7.4)	1074.3 (1000.6;1152.9)	953.2 (878.9;1032.1)	-11.3 (-6.8;-15.1)	2110.4 (1972.8;2257.3)	2133.4 (1993;2284.5)	1.1 (5.9;-3.2)

*Source: Global Burden of Disease Study 2017, Institute for Health Metrics and Evaluation.^151^*

**Table 3-2:** – Number of deaths and age-standardized mortality rates (per 100 000 inhabitants) due to ischemic heart disease, in Brazil and its Federative Units, 1990 and 2017, and percent changes of rates

Federative Units	1990		2017		Percent change (95% UI)
	Number (95% UI)	Rate (95% UI)	Number (95% UI)	Rate (95% UI)	
Acre	189.4 (182.1;196.3)	136.6 (131.4;141.7)	357.5 (333;378.2)	65.8 (61.2;69.6)	-51.8 (-55.6;-48.2)
Alagoas	1651.4 (1570;1736.9)	130.1 (123.8;136.9)	2673 (2560.9;2792.4)	88.9 (85;93.1)	-31.7 (-36.7;-26.7)
Amapá	98 (93.9;101.6)	125.3 (119.8;130)	272.6 (254;287.1)	64.1 (59.8;67.4)	-48.9 (-51.9;-45.6)
Amazonas	759.2 (710.6;793.9)	119.1 (111.3;124.4)	1437.4 (1353.9;1501.7)	59.3 (56;62.1)	-50.2 (-53.2;-47)
Bahia	7454.5 (7013;7908)	114.8 (108.1;121.6)	10948.6 (10506.6;11402.3)	68.9 (66;71.8)	-40 (-44.1;-35.8)
**Brazil**	**129011.3 (126506.8;131646)**	**169.1 (165.8;172.5)**	**175791.5 (171318.3;179410.1)**	**80 (77.9;81.7)**	**-52.7 (-53.9;-51.4)**
Ceará	3476.7 (3149.9;3838)	88 (79.8;97)	6852.5 (6528.4;7135.3)	68.7 (65.3;71.5)	-22 (-30.1;-12.5)
Distrito Federal	693.4 (670.8;723.3)	163.8 (159.2;169)	1255 (1188.1;1334.8)	68.1 (64.3;72.4)	-58.4 (-61;-55.5)
Espírito Santo	1962.9 (1903.6;2020.5)	184 (178.6;189.5)	2849 (2723.4;2991.5)	70.1 (67;73.5)	-61.9 (-63.7;-60.2)
Goiás	2187.7 (2095.2;2284.9)	148.7 (142.7;155.3)	4608.3 (4418.6;4837.6)	73.8 (70.8;77.3)	-50.3 (-52.7;-47.9)
Maranhão	2681.4 (2413.1;2998.8)	109.5 (98.6;122.8)	5059.4 (4771.1;5411.4)	81.3 (76.6;87)	-25.8 (-31.9;-18.4)
Mato Grosso	1038.9 (971.1;1108.6)	157 (147.1;166.7)	2001.7 (1905.6;2108)	71.9 (68.5;75.7)	-54.2 (-57.6;-50.4)
Mato Grosso do Sul	1279 (1236.9;1322.1)	178 (172.7;183.3)	2417 (2305.2;2535.9)	92.1 (87.8;96.5)	-48.3 (-51.1;-45.2)
Minas Gerais	13676.9 (13229;14143.9)	174.9 (169.3;180.2)	16472.4 (15831.1;17142.3)	65.4 (62.8;68)	-62.6 (-64.5;-60.6)
Pará	2429.1 (2281.2;2555)	132.1 (124;138.8)	4475.8 (4255.9;4707.2)	72.4 (68.9;76.1)	-45.2 (-49;-41.4)
Paraíba	2656.4 (2457.3;2857.9)	119.5 (110.8;128.7)	4347.9 (4037.2;4662.3)	92.4 (85.8;99.2)	-22.7 (-30.6;-12.9)
Paraná	7635.2 (7436.3;7844.1)	213.6 (208.1;218.8)	10022.2 (9595.8;10414)	83.9 (80.3;87.2)	-60.7 (-62.5;-59)
Pernambuco	6654.6 (6472.6;6840.5)	172 (167;176.9)	9894.9 (9459.7;10308.8)	102.5 (97.8;106.9)	-40.4 (-43.4;-37.4)
Piauí	1454.4 (1315.5;1623.6)	110.5 (100.2;123.7)	2657.7 (2534.2;2880.6)	73.5 (70;79.6)	-33.5 (-39.4;-26.8)
Rio de Janeiro	19105.3 (18615.3;19589.5)	237.3 (231.6;242.9)	21214.7 (20296.2;22025.8)	99.8 (95.4;103.5)	-58 (-59.7;-56.2)
Rio Grande do Norte	1678.9 (1569.7;1800.4)	107.4 (100.4;115.1)	3054.6 (2909.4;3207.9)	80.2 (76.3;84.4)	-25.3 (-32;-17.8)
Rio Grande do Sul	10753.7 (10391.3;11026.2)	200.1 (193;205.2)	11915.8 (11240.8;12411.3)	80.6 (76.1;84.1)	-59.7 (-61.5;-57.9)
Rondônia	483.1 (449.2;518.8)	183.3 (172.6;193.6)	1115.8 (1002.5;1234.4)	86.9 (78.3;95.9)	-52.6 (-57.9;-47.2)
Roraima	67.3 (61.3;74)	180.3 (167.7;194)	207.7 (184.9;232.6)	82 (73.6;91.2)	-54.5 (-60.4;-48)
Santa Catarina	3677.2 (3564.2;3771.3)	189.9 (183.9;195)	5461 (5194.3;5690.8)	77.7 (74;80.9)	-59.1 (-61.1;-57.2)
São Paulo	33956.3 (33066.3;34860.2)	216.3 (210.7;221.3)	41732.9 (40054.1;43206.1)	84.2 (80.8;87.2)	-61.1 (-62.6;-59.5)
Sergipe	882.9 (837.6;933.2)	108.6 (103.1;114.7)	1509.1 (1440.4;1579.9)	72.8 (69.5;76.3)	-32.9 (-37.1;-28.4)
Tocantins	427.5 (379.8;475.3)	153.9 (139.6;169.6)	977 (912.6;1047.3)	73.4 (68.6;78.6)	-52.3 (-57.4;-46.8)

*Source: Global Burden of Disease Study 2017, Institute for Health Metrics and Evaluation.^151^*

**Table 3-3:** – Number of DALYs and age-standardized DALY rates (per 100 000 inhabitants) due to ischemic heart disease, in Brazil and its Federative Units, 1990 and 2017, and percent changes of rates

Federative Units	1990		2017		Percent change (95% UI)
	Number (95% UI)	Rate (95% UI)	Number (95% UI)	Rate (95% UI)	
Acre	4490.5 (4309.4;4679.4)	2481.7 (2383.9;2581.2)	7971 (7417;8477.2)	1295.1 (1208;1374.4)	-47.8 (-51.7;-44.2)
Alagoas	38381.3 (36257.4;40572.7)	2644.8 (2506.1;2789.1)	58660.8 (55937.2;61544.4)	1852.2 (1766.3;1942.7)	-30 (-35.3;-25.1)
Amapá	2256.8 (2161.6;2349.2)	2139.7 (2042.8;2217.4)	6578.5 (6114.7;6944.1)	1275.9 (1188.4;1345.7)	-40.4 (-44;-36.6)
Amazonas	17148.9 (16081.9;18005.8)	2072.2 (1942.7;2171.1)	31464.8 (29293.6;33102.3)	1136.6 (1066.1;1192.7)	-45.1 (-48.5;-41.7)
Bahia	166604.8 (156031.4;177498)	2294.4 (2154;2441)	231348.2 (220711.5;241597.1)	1457.6 (1390.2;1521.1)	-36.5 (-41.1;-31.4)
**Brazil**	**2959511 (2894436.2;3033217.1)**	**3159.1 (3091.6;3233.5)**	**3678315.8 (3579145.9;3768782)**	**1602.4 (1559.2;1641.9)**	**-49.3 (-50.7;-47.8)**
Ceará	71384.3 (64483.4;79273.1)	1691.4 (1530.8;1874.3)	128192.5 (121929.7;134050.4)	1307.4 (1243.4;1368.7)	-22.7 (-30.6;-13.7)
Distrito Federal	19308.3 (18564.4;20275.2)	2845.7 (2756.4;2962.1)	27808.1 (26221.3;29618.7)	1121.3 (1059.8;1190.4)	-60.6 (-62.8;-58.2)
Espírito Santo	44177 (42688.1;45695)	2975.1 (2882.6;3065.8)	59450.4 (56507.8;62558.7)	1381.9 (1314.9;1451.2)	-53.6 (-55.8;-51.3)
Goiás	55914.3 (53327.6;58557.6)	2636.3 (2522.6;2754.3)	105343.2 (100500;110380.6)	1523.3 (1454.2;1595.6)	-42.2 (-45.2;-39.3)
Maranhão	73066.2 (65836.5;81337.6)	2557 (2305.8;2845.1)	109267.8 (103121.4;115769.7)	1680.2 (1586.4;1780.6)	-34.3 (-39.8;-27.3)
Mato Grosso	26946.1 (25171.5;28837)	3010.9 (2820.1;3213.2)	46202.4 (43768.5;48781)	1433.9 (1363;1510.9)	-52.4 (-56.1;-48.3)
Mato Grosso do Sul	30871.7 (29551.5;32110.1)	3216 (3102.7;3326.8)	52574.5 (50174.7;55121.4)	1835.5 (1754.7;1923.1)	-42.9 (-46.1;-39.5)
Minas Gerais	313998.9 (302102.3;326795.2)	3087.6 (2980.2;3202.3)	343731.3 (329343.3;358925.5)	1346.9 (1290.4;1406.4)	-56.4 (-58.7;-53.9)
Pará	54735.8 (51421.7;57718.5)	2415.2 (2272.2;2540.5)	100381.7 (95156.5;105848.4)	1469.8 (1395.9;1547.3)	-39.1 (-43.1;-35.1)
Paraíba	54949.7 (50451.8;59641.8)	2344.6 (2160.6;2543.5)	82981 (77060.1;89538.2)	1821.8 (1692;1966.4)	-22.3 (-30.7;-12.1)
Paraná	174525.4 (169345.9;179809.9)	3561.7 (3464.6;3661)	206379.1 (196027.1;215789.4)	1595.2 (1516.5;1666.7)	-55.2 (-57.4;-53.1)
Pernambuco	144001.4 (139657.6;148414.6)	3110.2 (3018.9;3200.9)	207666.8 (198225.5;216500.9)	2076.1 (1982.5;2164.9)	-33.2 (-36.7;-29.9)
Piauí	33224.1 (29689.6;37114.1)	2189.2 (1970.8;2442.1)	54215.2 (51650.7;57900)	1502.3 (1430.9;1604.2)	-31.4 (-37.5;-24.5)
Rio de Janeiro	454987.3 (442500.6;467807.1)	4481.1 (4365.5;4597)	450050 (429870;467261.3)	2063.8 (1970.8;2142.5)	-53.9 (-55.9;-52)
Rio Grande do Norte	32424.2 (30203.4;34875.9)	1972.3 (1838.5;2120.1)	60710.1 (57559.4;64019.8)	1623.5 (1539.9;1712.6)	-17.7 (-25.2;-9.2)
Rio Grande do Sul	242410.1 (233975.5;249439.7)	3573.4 (3449.5;3665.5)	231413.1 (216004.4;242521.6)	1542.5 (1439.4;1617.4)	-56.8 (-59;-54.6)
Rondônia	14130.4 (13109.8;15219.2)	3388.7 (3169.3;3608.1)	24830 (22172.1;27619.4)	1657 (1487.6;1836.9)	-51.1 (-57;-44.9)
Roraima	1916.4 (1730.6;2127)	2919.1 (2687.8;3186.5)	4797.3 (4262.1;5419.4)	1374.5 (1225;1539.8)	-52.9 (-59.4;-45.6)
Santa Catarina	82034.2 (79539.6;84426.2)	3208.9 (3105;3295.8)	112363.3 (106011.6;117727.3)	1436.9 (1357.2;1503.9)	-55.2 (-57.5;-53)
São Paulo	777221.4 (755207.8;800862.7)	3746.1 (3646.7;3850.3)	880339.1 (842359;915273)	1669.2 (1598.4;1735)	-55.4 (-57.2;-53.5)
Sergipe	17580.3 (16558.7;18785.1)	2012.2 (1900.5;2143.3)	32232.2 (30675;33873.9)	1474.5 (1403.3;1547)	-26.7 (-31.9;-21.1)
Tocantins	10821.1 (9291.9;12213.6)	2516.2 (2233.5;2791.5)	21363.6 (19891.7;22878)	1503.1 (1400.6;1606.9)	-40.3 (-47.2;-32.3)

*Source: Global Burden of Disease Study 2017, Institute for Health Metrics and Evaluation.^151^*

**Table 4-1:** – Age-standardized prevalence rates of cardiomyopathy and myocarditis, per 100 000 inhabitants, and percent change of rates, for both sexes, and for males and females, in Brazil and its Federative Units, 1990 and 2017

	Both sexes	Female					Male		
	1990	2017	Percent change (95% UI)	1990	2017	Percent change (95% UI)	1990	2017	Percent change (95% UI)
Brazil	102.8 (82.5;125.7)	112.4 (92.2;134.2)	9.4 (15.3;4.1)	107.7 (86.2;131.2)	115 (95;137.4)	6.9 (14.2;0.2)	97.1 (77.5;119)	109 (88.3;131.7)	12.3 (20;5.4)
Acre	61 (49.2;75.6)	65.9 (53.2;81.1)	8 (21.6;-4.5)	63.8 (50.7;79.9)	66.3 (51.7;81.8)	3.9 (26.6;-13.3)	58.6 (46;74.4)	65.5 (51.5;82.3)	11.8 (32.5;-5.6)
Alagoas	84 (65.4;105.2)	103.3 (82.9;126.8)	23.1 (38.9;8.9)	86.6 (67.4;109.4)	101.4 (80.5;125.5)	17.1 (37.4;-0.5)	80.8 (60.5;104)	105.7 (82.7;133.3)	30.8 (56.3;9.4)
Amapá	104.9 (84.8;128.4)	106.4 (86.4;130.8)	1.4 (13.8;-9.1)	102.7 (83.2;126.6)	103.6 (82.8;128.4)	0.9 (19.5;-15)	107.1 (84.7;133.7)	108.9 (86.4;135.3)	1.6 (19.9;-12.4)
Amazonas	95.3 (76.7;117.9)	98.7 (78.8;120.6)	3.5 (15.3;-7.7)	94.3 (75.1;116.1)	95.7 (75.9;118)	1.5 (17.7;-13.2)	96.1 (74.8;120.2)	101.6 (79.7;126.8)	5.7 (24.6;-10.1)
Bahia	67.2 (51.5;84.9)	74.1 (58.9;91.2)	10.4 (25.4;-2.3)	70.4 (53.8;89.7)	73.8 (58.2;91.7)	4.7 (24.1;-11.1)	63.6 (47.9;82.5)	74.7 (58.6;95.4)	17.5 (41.8;-1.9)
Ceará	105.6 (83.9;128.9)	130.2 (105.9;157.5)	23.2 (37.7;10)	96.8 (76.9;119.8)	116.9 (92.7;142.9)	20.7 (41.8;2.5)	115.4 (90.2;145.4)	146.4 (117.2;181.7)	26.8 (49;9.5)
Distrito Federal	125.6 (92.7;161.3)	144 (113;179.2)	14.6 (32.7;1.3)	134.7 (98.8;171.7)	148.5 (116.4;183.6)	10.2 (33.1;-5.9)	116 (82.6;156.4)	138.9 (104.8;177.4)	19.8 (48.5;-0.3)
Espírito Santo	104.2 (83.5;127.5)	104 (84.4;126.2)	-0.1 (10.8;-11.7	95.8 (76.3;120.3)	94.2 (75.1;115.8)	-1.7 (15;-15.7)	112.9 (88.7;139.5)	115.2 (91.7;143)	2 (18.3;-11.9)
Goiás	99.1 (71.5;130.5)	125.9 (97.2;160.3)	27.1 (49.3;10.8)	116.1 (81;154.9)	141.2 (109.2;179.2)	21.6 (51.5;1.9)	83 (58.9;111.8)	108.7 (82.5;142.4)	31 (63.1;8.5)
Maranhão	61.3 (48.3;75.9)	76.7 (61.9;94.2)	25.2 (41.6;10.8)	58.7 (46;73.9)	69.1 (54.3;86.4)	17.6 (41.1;-1.4)	63.8 (48.5;81.1)	85.1 (67.2;106.1)	33.5 (60.4;12.1)
Mato Grosso	92.9 (74;115.1)	100.5 (80.4;123.5)	8.2 (21.7;-3.1)	100.3 (79.3;124.1)	106.4 (84.1;132.4)	6.1 (22.8;-8.4)	86.3 (65.6;109.5)	94.5 (73.9;118.4)	9.6 (30.4;-7.3)
Mato Grosso do Sul	126.2 (100.6;155.9)	141.5 (113.6;172.9)	12.1 (26.2;0.5)	131.9 (105;162.7)	140.3 (113;174)	6.4 (23.2;-9.8)	120.5 (91.5;154.7)	142.4 (112.8;175.3)	18.1 (40.9;0.6)
Minas Gerais	110.7 (85.9;139.6)	123.7 (98;152)	11.7 (27.3;-0.9)	123.3 (95.2;155.8)	132.4 (105.3;163.8)	7.3 (28;-7.5)	95.8 (70.1;124.4)	113 (87.8;142.4)	17.9 (42.6;-0.7)
Pará	72.9 (59;88.7)	74.8 (60.4;92.1)	2.6 (15.9;-9.4)	73.1 (57.6;90.4)	72.6 (58.3;91.3)	-0.7 (16.4;-16.5	72.8 (57.8;90.9)	77.1 (60.5;95.1)	5.8 (25.4;-10.7)
Paraíba	116.2 (92.8;142.4)	138.3 (112.3;168.2)	19 (34.8;7)	118.5 (94.5;145.7)	140.4 (112.6;172.9)	18.5 (39.6;1.2)	113.5 (89.8;142.8)	135.7 (107.6;167.8)	19.6 (40.3;1.7)
Paraná	90.4 (71.4;111.4)	97 (77.5;118.8)	7.3 (21.1;-4.3)	95.8 (74.8;119.6)	99.5 (79.5;123)	3.8 (21.9;-11.1)	84.7 (64.6;106.9)	93.7 (73.7;117)	10.7 (32.5;-7.3)
Pernambuco	81.8 (64.8;101.5)	85.3 (68.5;104)	4.3 (17.8;-7.4)	80.5 (62.3;100.8)	81.9 (64.1;102.3)	1.8 (20.4;-14.8)	83.6 (64.2;106)	89.9 (70.6;112.4)	7.6 (29.5;-9.2)
Piauí	64.6 (50.1;80.1)	75.4 (60.2;92.7)	16.8 (33.1;3.5)	65.9 (51.4;82.2)	74.5 (58.7;92.7)	13.1 (34.9;-3.7)	63.1 (47.5;81.5)	76.4 (59.5;96.4)	21 (45.6;0.3)
Rio de Janeiro	117.3 (94.5;144.3)	113.9 (92.7;138.1)	-2.9 (8.7;-12.7)	115.9 (92.9;145)	112.8 (91.5;137.7)	-2.7 (12.4;-15.7	119.3 (93;149)	115.6 (91.9;142.1)	-3.1 (13.9;-16.3)
Rio Grande do Norte	75.9 (60.8;95.4)	83.6 (67.4;102.5)	10.1 (23.8;-1.8)	68 (53.4;85.4)	74.7 (59.2;93.7)	9.8 (30.5;-6.9)	84.6 (67.8;107.3)	94.9 (75.6;116.7)	12.1 (32;-4.2)
Rio Grande do Sul	75.2 (59.7;93.4)	74.7 (60;91.3)	-0.7 (12.1;-11.7	73.1 (57.3;92.8)	72.1 (57.7;89.4)	-1.4 (17.2;-16.8	77 (60;96.4)	76.9 (60.5;95.5)	0 (18.5;-15.9)
Rondônia	69.9 (54.3;87.9)	76.3 (62;94.4)	9.2 (25.4;-3.8)	74.1 (57.7;94.9)	78.7 (63.2;98.4)	6.1 (25.9;-10.5)	66.2 (49.8;84.5)	74 (57.8;92.9)	11.7 (34.3;-7)
Roraima	141.5 (114.4;172.9)	145 (118.1;174.6)	2.5 (14.5;-7.8)	137.9 (111;168.4)	140.3 (111.4;171.8)	1.7 (18.3;-12.1)	143.8 (113.8;177.9)	149.2 (118.5;183.1)	3.7 (21.1;-10.2)
Santa Catarina	107.2 (86.6;131.2)	106.7 (86.6;130)	-0.5 (10.2;-10.9	109.7 (88.8;135.7)	108.4 (86.2;133.7)	-1.2 (14;-15.3)	103.5 (81.8;129)	103.6 (82.4;127.7)	0.1 (17.4;-14.5)
São Paulo	138.4 (108.2;171.2)	147.7 (120.7;178)	6.7 (21.3;-4.4)	152.1 (118.7;187.1)	158 (128;193.1)	3.8 (21.1;-10.7)	121.3 (92.5;156)	134.1 (105.8;164.7)	10.5 (29;-6.6)
Sergipe	100.2 (80.6;123.9)	115.3 (93.9;141.5)	15.2 (29.1;3.3)	102.5 (81.7;128.8)	115 (92.6;141.2)	12.2 (31.1;-3.9)	97.3 (75.4;120.9)	115.2 (91.3;142.9)	18.4 (39.4;2.2)
Tocantins	71.3 (53.8;89.6)	88.8 (70.6;109.2)	24.6 (43.3;9.2)	71.1 (54.4;91.9)	85.2 (67.3;106.3)	19.9 (45.4;0.7)	71.6 (53;92.1)	92.3 (71.6;116.4)	28.9 (59;7.9)

*Source: Global Burden of Disease Study 2017, Institute for Health Metrics and Evaluation.^188^*

**Table 4-3:** – Incidence rates of cardiomyopathy and myocarditis per 100 000 inhabitants, and percent change of rates, by age and sex, Brazil, 1990 and 2017

Age group	1990	2017	Percent change (95% UI)
***Both sexes***			
Age-standardized	46.3 (41.5;52.1)	46.7 (41.8;52.6)	0.8 (-0.3;1.8)
Under 5	11.2 (8.9;13.9)	11.3 (9;14.1)	1.1 (-0.8;3.5)
5-14 years	16.5 (12;22.2)	16.7 (12.3;22.6)	1.1 (-1.4;3.6)
15-49 years	32.8 (27.1;39)	34.2 (28.2;40.6)	4.2 (0.4;8.2)
50-69 years	82.4 (67.1;102)	83.4 (67.5;103.2)	1.2 (-1;3.5)
70+ years	203.1 (165.4;252.3)	214.8 (176.5;263.2)	5.8 (2.2;10.1)
All Ages	36.5 (32.5;41)	49 (43.7;55.4)	34.4 (29;39.9)
***Male***			
Age-standardized	46 (41.1;51.8)	45.5 (40.7;51.5)	-1 (-2.5;0.5)
Under 5	11.2 (8.8;13.8)	11 (8.7;13.7)	-1.3 (-4.1;2.5)
5-14 years	16.5 (11.9;22.2)	16.2 (11.9;22)	-1.5 (-5.1;2.7)
15-49 years	32.6 (26.9;38.7)	33.2 (27.4;39.5)	1.9 (-2.5;5.9)
50-69 years	81.6 (65.9;100.9)	81.6 (66;101.9)	0 (-3.2;2.9)
70+ years	198.9 (161.2;247.1)	205.6 (167.7;253.2)	3.4 (-0.4;7.7)
All Ages	35.4 (31.4;39.9)	45.7 (40.6;51.9)	29.1 (23.9;34.9)
***Female***			
Age-standardized	46.6 (41.6;52.4)	47.7 (42.7;53.8)	2.3 (0.8;3.8)
Under 5	11.3 (9;14)	11.7 (9.4;14.6)	3.7 (0.8;7.2)
5-14 years	16.6 (12.2;22.2)	17.2 (12.7;23.1)	3.7 (0.3;7.1)
15-49 years	33 (27.2;39.4)	35.2 (29;41.8)	6.4 (2.1;11)
50-69 years	83.2 (67.6;103)	85.1 (69.1;105.6)	2.3 (-1;5.3)
70+ years	206.5 (168.6;257.1)	221.6 (182.6;270.7)	7.3 (2.9;12.4)
All Ages	37.6 (33.4;42.3)	52.3 (46.6;59)	39.1 (33.3;45.3)

*Source: Global Burden of Disease Study 2017, Institute for Health Metrics and Evaluation.^188^*

**Table 4-4:** – Number of deaths and age-standardized mortality rates (per 100 000) due to cardiomyopathy and myocarditis, and percent change of rates, in Brazil and its Federative Units, 1990 and 2017

Location	1990		2017		Percent change (95% UI)
	Number (95% UI)	Rate (95% UI)	Number (95% UI)	Rate (95% UI)	
**Brazil**	**9734 (8417;10163)**	**10.9 (9.6;11.4)**	**18812 (17885;21745)**	**8.6 (8.2;9.9)**	**-21.2 (-26.8;-2.6)**
Acre	11 (10;15)	5.7 (5.2;7.9)	29 (25;45)	4.8 (4.1;7.6)	-15.2 (-28;26.1)
Alagoas	172 (137;194)	10.6 (8.4;11.9)	262 (239;289)	8.4 (7.7;9.3)	-20.7 (-32.3;1.9)
Amapá	7 (6;9)	6.7 (6;8.4)	32 (28;42)	6.7 (5.9;8.7)	-0.1 (-13.5;36)
Amazonas	55 (50;69)	6.3 (5.8;8)	135 (118;210)	4.9 (4.3;7.7)	-22.4 (-33.1;18.6)
Bahia	581 (503;639)	7.9 (6.6;8.8)	892 (800;1189)	5.6 (5;7.5)	-28.9 (-39.9;10.5)
Ceará	362 (296;425)	7.4 (5.9;8.7)	642 (592;704)	6.5 (6;7.1)	-11.8 (-27.5;15.6)
Distrito Federal	95 (71;103)	16.2 (12.5;17.5)	268 (221;299)	13.1 (10.8;14.5)	-19.4 (-28.2;-5)
Espírito Santo	138 (121;147)	10.5 (9.3;11.3)	261 (236;355)	6.4 (5.8;8.7)	-39.7 (-47.2;-13.5)
Goiás	345 (246;378)	20.1 (14.7;22.2)	730 (609;792)	11.8 (9.8;12.8)	-41.2 (-47.9;-24.8)
Maranhão	232 (193;286)	6.2 (5;8.1)	303 (268;458)	4.6 (4.1;7.1)	-25.7 (-41.7;3.6)
Mato Grosso	75 (68;83)	8.7 (7.8;9.7)	202 (182;277)	7.1 (6.3;9.5)	-18.5 (-29;14)
Mato Grosso do Sul	122 (89;133)	14.5 (10.6;15.9)	261 (236;293)	10 (9.1;11.2)	-30.8 (-39.9;2.9)
Minas Gerais	1238 (962;1334)	13.1 (10.3;14)	2059 (1903;2288)	8.4 (7.7;9.3)	-36 (-42.8;-16.7)
Pará	114 (102;162)	4.9 (4.5;7)	314 (274;482)	4.8 (4.2;7.3)	-3.6 (-15.2;29.3)
Paraíba	279 (220;318)	11.3 (8.9;12.9)	456 (392;515)	9.8 (8.4;11.1)	-12.9 (-27.5;8)
Paraná	567 (479;605)	13 (11;13.9)	769 (685;1168)	6.6 (5.9;9.9)	-49.2 (-56.2;-19.1)
Pernambuco	330 (306;367)	7.1 (6.6;8)	775 (700;884)	7.9 (7.1;9.1)	11.3 (-2.9;26.3)
Piaui	124 (102;146)	7.3 (5.9;8.9)	183 (165;233)	5.1 (4.6;6.5)	-30.7 (-43.4;3.1)
Rio de Janeiro	830 (749;1076)	8.9 (8.2;11.5)	2749 (2069;2994)	13.1 (10;14.2)	46.2 (12.6;63.3)
Rio Grande do Norte	92 (80;119)	5.1 (4.4;6.8)	176 (153;256)	4.6 (4;6.8)	-8.8 (-25.2;34.4)
Rio Grande do Sul	298 (268;487)	4.8 (4.3;7.8)	709 (617;1221)	4.9 (4.3;8.5)	2.7 (-7;14.1)
Rondônia	30 (27;36)	7.8 (7.1;9.3)	83 (70;120)	6.1 (5.2;8.8)	-22.6 (-34.2;4.1)
Roraima	10 (7;11)	17.1 (12.1;19.4)	40 (33;47)	13.5 (11.2;16.1)	-20.9 (-36.3;12.7)
São Paulo	3260 (2466;3519)	17.6 (13.5;19)	5766 (4987;6355)	11.8 (10.3;12.9)	-33.1 (-39.7;-16.4)
Santa Catarina	246 (229;264)	10.9 (10;11.8)	493 (449;681)	7 (6.3;9.5)	-36.3 (-43.8;-14.8)
Sergipe	82 (73;90)	8.7 (7.7;9.7)	139 (125;173)	6.5 (5.9;8.1)	-24.6 (-35.6;2)
Tocantins	41 (31;49)	10.2 (8.1;12.4)	87 (77;113)	6.3 (5.5;8.3)	-38.5 (-51.3;-5.2)

*Source: Global Burden of Disease Study 2017, Institute for Health Metrics and Evaluation.^188^*

**Table 4-5:** – Mortality rates (per 100 000 inhabitants) due to cardiomyopathy and myocarditis, and percent change of rates, per age and sex, Brazil, 1990-2017

	1990	2017	Percent change (95% UI)
***Both sexes***			
Age-standardized	10.9 (9.6;11.4)	8.6 (8.2;9.9)	-21.2 (-26.8;-2.6)
Under 5	4.8 (4;5.6)	2.5 (2.2;2.8)	-47.1 (-56.4;-31.1)
5-14 years	0.4 (0.4;0.5)	0.4 (0.3;0.4)	-11.3 (-22.3;2.2)
15-49 years	2.4 (2;2.6)	2.5 (2.2;2.7)	1.4 (-6.7;21.7)
50-69 years	21.2 (17.7;22.5)	16.5 (15.3;19.3)	-22.4 (-29.3;-0.2)
70+ years	84.2 (74.2;89.1)	76.6 (72.1;90)	-9 (-16.8;14.7)
All Ages	6.5 (5.6;6.8)	8.9 (8.4;10.3)	36.3 (26.6;68.8)
***Male***			
Age-standardized	12.8 (10;13.7)	11.3 (10.5;14.4)	-12.1 (-20.9;28.2)
Under 5	4.8 (3.3;5.7)	2.7 (2.3;3.2)	-43 (-57.1;-3.5)
5-14 years	0.4 (0.4;0.5)	0.4 (0.4;0.5)	-3.9 (-20;15.3)
15-49 years	3.3 (2.5;3.6)	3.6 (3.2;4.2)	9.5 (-1;43.7)
50-69 years	26.2 (19.1;28.4)	23.6 (21.4;29.7)	-10.1 (-20.9;31.7)
70+ years	91.6 (69.7;99.7)	89.3 (81.2;119.5)	-2.5 (-15.1;53.7)
All Ages	7.4 (5.6;7.9)	10.7 (10;13.6)	44.8 (30.7;109.6)
***Female***			
Age-standardized	9.2 (8.8;9.6)	6.3 (6;6.6)	-31.3 (-35.5;-26.2)
Under 5	4.7 (3.9;5.9)	2.3 (2;2.6)	-51.5 (-64.1;-37.5)
5-14 years	0.4 (0.3;0.5)	0.3 (0.3;0.4)	-19.9 (-31.7;-4.4)
15-49 years	1.6 (1.5;1.6)	1.3 (1.2;1.4)	-15.9 (-23.1;-7.1)
50-69 years	16.6 (15.6;17.6)	10.2 (9.5;11)	-38.6 (-43.7;-32.3)
70+ years	78.3 (73.7;83.3)	67.4 (63.2;71.9)	-13.9 (-21.1;-5.9)
All Ages	5.7 (5.4;5.9)	7.1 (6.8;7.5)	26.3 (18.6;35.3)

*Source: Global Burden of Disease Study 2017, Institute for Health Metrics and Evaluation.^188^*

**Table 4-6:** – Number of DALYs and age-standardized DALY rates (per 100 000) due to cardiomyopathy and myocarditis, and percent change of rates, in Brazil and its Federative Units, 1990 and 2017

Location	1990		2017		Percent change (95% UI)
	Number (95% UI)	Rate (95% UI)	Number (95% UI)	Rate (95% UI)	
Brazil	328636.2 (283325;346745.9)	286.3 (246.7;300.6)	490571.8 (465903.3;556885.7)	222.3 (211.1;250.9)	-22.4 (-27.6;-7)
Acre	497.4 (436.2;639.6)	160.9 (145;211.8)	892.9 (770.2;1353.6)	128.3 (110.6;194.8)	-20.3 (-32.5;10.3)
Alagoas	7445.3 (5810.9;8943.2)	325.1 (260.1;373.1)	7889.4 (7045.9;8603.6)	238.4 (215;260)	-26.7 (-37.3;-12.9)
Amapá	260.3 (235.6;324.2)	162.7 (147.6;202.1)	1030.8 (926.3;1349.3)	172.1 (154.9;223)	5.8 (-6.7;41)
Amazonas	2178.8 (1943.2;2658.8)	162.5 (148;203.1)	4500.2 (3953.1;6675.2)	137.2 (120.4;205.4)	-15.6 (-25.7;19.1)
Bahia	19212.5 (17344.2;21147.3)	212.7 (188;232.3)	27181.1 (24338.5;33355.6)	175.2 (156.2;214.1)	-17.6 (-29.2;10.8)
Ceará	16413.7 (13000.4;20157.3)	260.6 (210.1;309.8)	18369.4 (16682.7;20144.2)	188.2 (170.6;206.9)	-27.8 (-41.3;-10.1)
Distrito Federal	3658.3 (2766.2;3988)	376.3 (286.8;405.1)	7351.8 (6159.1;8113.1)	276.6 (233.7;305.7)	-26.5 (-33;-14.2)
Espírito Santo	4540.9 (4041.7;4871.1)	241 (213.7;258.1)	7341.7 (6712.8;9314.8)	177 (161.6;223.8)	-26.5 (-34.9;-2.3)
Goiás	11003.3 (8024.8;12004.6)	428.7 (312.4;467.9)	18299.6 (15675.5;19752.8)	272.9 (234.7;293.9)	-36.3 (-43;-16)
Maranhão	13001.5 (10067.9;16807.4)	242.9 (200.7;299.4)	10003.6 (8755.1;13529.3)	137.8 (121.5;190.6)	-43.3 (-56.7;-23.3)
Mato Grosso	3073.7 (2771.1;3438.3)	229.1 (209.1;254.1)	5796.7 (5242.4;7786.8)	177.7 (161.1;236.5)	-22.4 (-31.3;2.3)
Mato Grosso do Sul	3865.2 (2943.9;4262.8)	329.7 (247;361.6)	6710.7 (6101.4;7492.7)	242 (221.4;268.6)	-26.6 (-35.7;6.5)
Minas Gerais	39564.4 (30507.2;42826)	327.1 (253.6;352.7)	51182.3 (47035.5;56511.4)	216.8 (198.6;238.8)	-33.7 (-40.5;-13.2)
Pará	4344.5 (3801.9;5889.4)	129.8 (116.4;180.3)	9977.8 (8660.1;14959.6)	132.8 (115.4;198.5)	2.3 (-9.4;25.4)
Paraíba	10241.9 (8039.5;12095.6)	341.3 (272.3;396.2)	11470.6 (9717.3;12811.3)	257.1 (217.9;287.7)	-24.7 (-36.3;-9.2)
Paraná	17321.6 (15163;18438.9)	292.2 (250.6;309.9)	19045.2 (16983.3;28587.7)	160.6 (142.8;234.4)	-45 (-52.3;-14.1)
Pernambuco	11872 (10716.4;13371.5)	200.8 (184.9;220.9)	21914.4 (19857.6;24316.1)	218.9 (198.9;243.3)	9 (-3.7;22.2)
Piauí	5486.8 (4473.4;6582.1)	227.8 (188.3;269.8)	5211.8 (4701.4;6542.5)	145.4 (130.7;182.9)	-36.2 (-48;-13)
Rio de Janeiro	28335.5 (24866.7;35902.2)	249.5 (221.8;316.3)	68879.7 (50970.5;75152.9)	334.9 (253.6;363.3)	34.2 (2.5;48.6)
Rio Grande do Norte	3445.5 (2978.7;4084.9)	153.8 (134.2;188.6)	5076.1 (4420.6;6901.5)	137.6 (119.9;186.8)	-10.6 (-24.8;18.6)
Rio Grande do Sul	9946.1 (8932.6;15081.7)	128.4 (115.3;197.2)	16836.2 (14705.4;28348.6)	124.5 (109.3;204.3)	-3.1 (-11.8;8.7)
Rondônia	1298.7 (1165.9;1533.3)	197.3 (178.5;234.3)	2395.2 (2053.6;3399.5)	153.1 (132.1;214.9)	-22.4 (-33.3;0.6)
Roraima	435.5 (314.2;500.3)	376.2 (264.9;423.8)	1242.6 (1054;1445.5)	296.9 (253.9;344.2)	-21.1 (-35.7;10)
Santa Catarina	7573.9 (7109.4;8246.8)	244.7 (229.3;262.6)	12498.4 (11367.3;16891.4)	168.5 (153.3;225.2)	-31.2 (-38.2;-12.2)
São Paulo	98920.7 (75875.1;106392.2)	410.9 (316.9;441.8)	142836.1 (123464.2;155246.1)	288.1 (253.2;311.2)	-29.9 (-36;-15)
Sergipe	2898.6 (2556.6;3238.8)	237.8 (211.1;262.1)	4042.2 (3652.6;4946.7)	180.8 (163.1;219.5)	-24 (-34.8;-0.4)
Tocantins	1799.9 (1350.9;2222.4)	255.1 (199.3;307.4)	2595.3 (2275.4;3210.4)	174.4 (152.9;216.8)	-31.6 (-45.1;-1.4)

*Source: Global Burden of Disease Study 2017, Institute for Health Metrics and Evaluation.^188^*

**Table 4-7:** – DALY rates due to cardiomyopathy and myocarditis, per 100 000 inhabitants, and percent change of rates, by age and sex, Brazil, 1990 and 2017

Age groups		1990	2017		Percent change (95% UI)
***All***					
Age-standardized		286.3 (246.7;300.6)	222.3 (211.1;250.9)		-22.4 (-27.6;-7)
Under 5		414 (345.2;487)	219.9 (195.5;246.4)		-46.9 (-56.3;-30.9)
5-14 years		35.3 (29.9;39.3)	31.3 (28.2;34.5)		-11.3 (-21.8;1.5)
15-49 years		126.4 (107.3;133.9)	125.6 (115.7;139.5)		-0.7 (-8.1;19.3)
50-69 years		624 (522.3;660.5)	492.5 (458.8;569.8)		-21.1 (-27.9;-0.2)
70+ years		1165 (1027.8;1237.6)	957.2 (888.7;1110.9)		-17.8 (-24.6;2.9)
All Ages		219.9 (189.6;232.1)	231.6 (220;262.9)		5.3 (-2;24.8)
***Male***					
Age-standardized	343.1 (262.8;365.7)			299 (279.9;363.4)	-12.8 (-21;17.6)
Under 5	419.5 (287.2;498.1)			239.9 (202.9;283.3)	-42.8 (-57;-3.3)
5-14 years	36.9 (30.7;41.7)			35.3 (30.6;41.1)	-4.5 (-19.7;13.8)
15-49 years	172.3 (132.9;185.4)			184.3 (164.1;213)	7 (-3.2;40.7)
50-69 years	769.4 (565.3;832.5)			699.1 (634.3;864)	-9.1 (-19.9;30.9)
70+ years	1293.3 (990.1;1415.9)			1165 (1058.4;1512.1)	-9.9 (-21.7;39.8)
All Ages	258.5 (198.1;276.9)			302.3 (282.1;367.3)	16.9 (5.5;55.7)
***Female***					
Age-standardized	233.8 (222.7;247.3)			153.5 (145.5;161.5)	-34.3 (-38.6;-30)
Under 5	408.3 (336.9;516.1)			199 (171.3;225)	-51.3 (-63.9;-37.2)
5-14 years	33.6 (28.6;37.5)			27.2 (23.5;30.4)	-19.1 (-30.3;-4.4)
15-49 years	82 (77.2;87)			68.4 (64.2;73)	-16.6 (-23.2;-8.8)
50-69 years	489.8 (460.6;520)			309.6 (288.2;332.4)	-36.8 (-41.7;-30.8)
70+ years	1061.9 (993.3;1138.6)			806.3 (749.9;868.8)	-24.1 (-30.4;-17.1)
All Ages	182.2 (172.3;194.9)			164 (155.3;172.6)	-10 (-16.9;-3.3)

*Source: Global Burden of Disease Study 2017, Institute for Health Metrics and Evaluation.^188^*

**Table 4-10:** – Number of deaths and age-standardized mortality rate due to Chagas disease, per 100 000 inhabitants, for females, in 1990 and 2017, and percent change of rates, in Brazil and its Federative Units

Location	1990		2017		Percent change (95% UI)
	Number of deaths (95% UI)	Mortality Rate (95% UI)	Number of deaths (95% UI)	Mortality Rate (95% UI)	
Acre	0.4 (0.4;0.5)	0.5 (0.5;0.6)	0.6 (0.5;0.7)	0.2 (0.2;0.2)	-58.7 (-65.8;-49.8)
Alagoas	25.8 (20.2;30.7)	3.5 (2.7;4.2)	33.4 (28.8;38.2)	1.9 (1.7;2.2)	-44.4 (-57.5;-24.8)
Amapá	0.2 (0.1;0.2)	0.3 (0.3;0.3)	0.3 (0.3;0.4)	0.1 (0.1;0.1)	-58.5 (-67;-49.9)
Amazonas	0.9 (0.8;1)	0.2 (0.2;0.2)	1.3 (1.2;1.6)	0.1 (0.1;0.1)	-56.4 (-63.1;-46.9)
Bahia	232.2 (210.6;255.3)	6.1 (5.5;6.7)	276.5 (253.1;304.8)	3.2 (2.9;3.5)	-47.8 (-54.4;-40)
Brazil	2638.1 (2545.4;2737.8)	5.3 (5.1;5.5)	2399.2 (2255.9;2597.1)	1.9 (1.8;2.1)	-63.7 (-66.3;-60.7)
Ceará	14.1 (11.5;18)	0.6 (0.5;0.8)	19.3 (17;22.2)	0.4 (0.3;0.4)	-44.4 (-59.3;-27.6)
Distrito Federal	98.7 (91.8;106.5)	29.2 (27.3;31.3)	103 (91.5;121.4)	8.5 (7.6;10.1)	-70.9 (-74.4;-66.1)
Espírito Santo	3.2 (2.9;3.5)	0.4 (0.4;0.5)	3.3 (2.9;3.9)	0.1 (0.1;0.2)	-67.3 (-72.1;-61.3)
Goiás	447.5 (419.6;480.6)	50.1 (46.8;54.1)	362 (329.1;407.7)	11.1 (10.1;12.5)	-77.9 (-80;-75.4)
Maranhão	4.3 (3;6.8)	0.3 (0.2;0.5)	5.6 (4.9;6.8)	0.2 (0.1;0.2)	-47.1 (-64.2;-23.3)
Mato Grosso	12.6 (11;14.5)	3.2 (2.8;3.6)	21 (18.4;25.5)	1.4 (1.3;1.7)	-55.4 (-62.5;-45.2)
Mato Grosso do Sul	16.1 (14.6;17.8)	3.6 (3.3;4)	19.5 (17;22.6)	1.4 (1.2;1.6)	-62.8 (-68.3;-56.2)
Minas Gerais	853.2 (809.6;904.2)	15.9 (15.1;16.8)	646 (593.4;706.5)	4.6 (4.3;5.1)	-70.8 (-73.7;-67.6)
Pará	6.8 (6;7.6)	0.6 (0.5;0.7)	8.3 (7.2;10)	0.2 (0.2;0.3)	-58.6 (-65.5;-49.1)
Paraíba	13.3 (9.7;15.6)	1.1 (0.8;1.3)	15.3 (12.9;17.7)	0.6 (0.5;0.7)	-45.9 (-58.7;-22.1)
Paraná	131.4 (122;140.3)	5.3 (4.9;5.6)	111.6 (100.4;125.1)	1.7 (1.5;1.9)	-68.8 (-72.3;-64.8)
Pernambuco	60.3 (53.4;66.2)	2.4 (2.2;2.7)	59.7 (52.8;67.5)	1.1 (0.9;1.2)	-56.4 (-62.7;-47)
Piauí	19 (15.4;24.5)	2.5 (2;3.2)	26.3 (23.1;30.8)	1.3 (1.2;1.6)	-46.1 (-59.2;-30)
Rio de Janeiro	29.5 (27;32.4)	0.5 (0.5;0.6)	26.9 (23.6;30.4)	0.2 (0.2;0.2)	-60.8 (-66.1;-54.6)
Rio Grande do Norte	4 (3.5;4.8)	0.5 (0.4;0.6)	4.9 (4.2;5.6)	0.2 (0.2;0.3)	-50.5 (-63.1;-37.1)
Rio Grande do Sul	20.9 (18.9;22.7)	0.6 (0.5;0.6)	19.8 (17.5;22.6)	0.2 (0.2;0.3)	-60.7 (-65.8;-54.1)
Rondônia	6.9 (6.1;7.8)	3.9 (3.5;4.4)	9.6 (7.9;11.6)	1.4 (1.1;1.7)	-64.8 (-72.1;-56.2)
Roraima	0.1 (0.1;0.1)	0.3 (0.2;0.3)	0.2 (0.1;0.2)	0.1 (0.1;0.1)	-63 (-72.2;-52)
Santa Catarina	4.6 (4.1;5.1)	0.4 (0.3;0.4)	5.1 (4.5;6)	0.1 (0.1;0.1)	-65 (-70;-58.7)
São Paulo	614.1 (583.5;644.5)	5.4 (5.1;5.6)	593.9 (544.1;650.2)	2 (1.9;2.2)	-61.9 (-65.3;-58.1)
Sergipe	3.6 (3.1;4.2)	0.8 (0.7;0.9)	4.1 (3.6;4.7)	0.3 (0.3;0.4)	-55.9 (-63.5;-43)
Tocantins	14.5 (11.6;19)	7.9 (6.5;10.2)	21.7 (18.6;25.7)	3.2 (2.8;3.8)	-59 (-70;-46.2)

*Source: Global Burden of Disease Study 2017, Institute for Health Metrics and Evaluation.^188^*

**Table 4-11:** – Prevalence number and age-standardized prevalence rate of heart failure impairment for all causes (per 100 000 inhabitants) and percent change of rates, 1990 and 2017, in Brazil and its Federative Units

Location	1990		2017		Percent change (95% UI)
	Number (95% UI)	Rate (95% UI)	Number (95% UI)	Rate (95% UI)	
Brazil	670194.8 (589952.6;753672.6)	818.1 (718.1;922.8)	1686320.1 (1478563.8;1890537.3)	777.2 (680;874.8)	-5 (-7.1;-3)
Acre	1235.9 (1083.5;1395.6)	764.3 (668.5;869)	4025.6 (3559.4;4534.9)	728.8 (638.1;830.1)	-4.6 (-10.1;1.8)
Alagoas	9783 (8509.2;11210.5)	752.5 (654.5;861.9)	22691.5 (19784.3;25922)	764.8 (664.4;879.3)	1.6 (-5.7;8.3)
Amapá	748.2 (662;841.1)	779.1 (680.7;889.6)	3278.9 (2865.5;3672.3)	749.3 (651.9;845.2)	-3.8 (-9.5;2.6)
Amazonas	6097.6 (5376.6;6855.3)	809.8 (709.1;919)	19459.2 (17131;21872.1)	775.9 (678.8;884.1)	-4.2 (-10.2;1.9)
Bahia	52840.3 (46323.4;60082)	783.5 (685.2;893)	118062.7 (103361.3;134066.1)	753.3 (656.9;857.9)	-3.8 (-9.6;2.5)
Ceará	30093.8 (26385.1;34137.4)	739.5 (648;842.1)	77144.8 (67097.6;87800.2)	785.5 (682.6;896)	6.2 (-0.8;14.2)
Distrito Federal	4256.7 (3710.8;4838.9)	813.3 (701.7;932)	16100.7 (13996.8;18333.4)	753.5 (654.5;850.2)	-7.4 (-13.1;-0.6)
Espírito Santo	11320.9 (9847.7;12942.2)	841.5 (730.3;961.6)	31391.5 (27390.7;35566.7)	782.7 (680;889.7)	-7 (-13.2;-0.6)
Goiás	14142 (12371.7;16150.6)	800.7 (703.5;912.7)	46168.1 (40298.2;52244.1)	753 (655.9;854)	-6 (-12.9;1)
Maranhão	18235.7 (15857.1;20802.4)	747.2 (650.9;852.8)	49180.9 (43277;55993.9)	795.1 (697.6;907.8)	6.4 (0.2;13.5)
Mato Grosso	5774.8 (5067;6502.3)	819.3 (712.6;938)	21845.3 (19017.4;24622)	789.4 (688;895.4)	-3.7 (-9.6;3)
Mato Grosso do Sul	6795.1 (5934.8;7652)	846.4 (740.2;961.2)	21183 (18418.4;24002)	816 (710.9;922.6)	-3.6 (-9.3;3.4)
Minas Gerais	74411.2 (64608;84527.1)	826.9 (722;940.5)	187809.8 (163412.5;214570.5)	759.5 (659.4;867)	-8.1 (-14.2;-1.6)
Pará	16002.3 (14153.8;18014.5)	789.4 (694.3;893.2)	46324.1 (40809.4;52186.1)	746.6 (652.1;844.8)	-5.4 (-11.3;0.9)
Paraíba	17922.4 (15619.1;20442.3)	772 (675.4;881)	36827.2 (32186.6;41911.1)	794.6 (693.4;902.5)	2.9 (-3.6;10.2)
Paraná	35843.5 (31360.3;40661.2)	834.4 (726.6;948.5)	93386.5 (81689;106563.6)	779.7 (684;892.6)	-6.6 (-12.2;0.1)
Pernambuco	34084.1 (29826.4;39017.2)	793.9 (695;908.1)	72004 (62756.3;81953.9)	753.9 (655.5;862.9)	-5 (-11.2;1.3)
Piauí	11016.2 (9596.9;12503.6)	803.5 (698.9;912.6)	29097.2 (25425.9;33105.2)	812.7 (708.9;927)	1.1 (-5.6;8.5)
Rio de Janeiro	72976.8 (63619.5;83192)	850.4 (744;970.5)	162697.6 (140265.7;185508.6)	778.9 (674.2;887.1)	-8.4 (-14.1;-1.7)
Rio Grande do Norte	13462.3 (11756.9;15396.2)	827 (720.5;948.1)	31332.8 (27529.6;35470.6)	839 (734;955.6)	1.5 (-4.9;8.1)
Rio Grande do Sul	51590.7 (45263.1;58166.6)	862.8 (754.1;980)	115132.9 (100064.2;130860)	787.4 (685.2;894.6)	-8.7 (-14.7;-2.9)
Rondônia	2451.8 (2150.6;2766.5)	813.4 (710.9;925.4)	9980.6 (8742.3;11325)	766 (669.2;872.1)	-5.8 (-11.9;1)
Roraima	419.1 (368.5;471.8)	809.3 (706.3;925)	2297.9 (2000.3;2611)	774.6 (674.4;884.7)	-4.3 (-10.5;2)
Santa Catarina	19387.5 (16978;21842.4)	847 (741.4;957.7)	55662.9 (48893.1;63470.8)	779.6 (685.4;894.9)	-8 (-13.7;-2)
São Paulo	150009 (131202;169778.7)	842.8 (734.6;959.2)	387169.5 (336629;442688.8)	787.9 (685;899.2)	-6.5 (-12.8;-0.4)
Sergipe	6409.8 (5592.5;7302.6)	754.4 (657.2;860.7)	15587.4 (13661.6;17664.6)	763.8 (668.4;870.4)	1.2 (-4.7;8.8)
Tocantins	2884.2 (2501.8;3284.4)	789.5 (691.7;906.1)	10477.4 (9147.4;11899)	796.5 (692.4;907.9)	0.9 (-5.9;8.5)

*Source: Global Burden of Disease Study 2017, Institute for Health Metrics and Evaluation.^188^*

**Table 4-12:** – Prevalence rates of heart failure, per 100 000 inhabitants, and percent change of rates, by sex and age groups, Brazil, 1990 and 2017

Age group	1990	2017	Percent change (95% UI)
	Prevalence	Prevalence	
Age-standardized	818.1 (718.1;922.8)	777.2 (680;874.8)	-5 (-7.1;-3)
Under 5	46.3 (32;63.8)	45 (30.8;61.9)	-2.9 (-5.5;-0.1)
5-14 years	34.7 (24.2;47.2)	34.1 (23.6;46.7)	-1.6 (-4.5;1.3)
15-49 years	107.1 (90.7;124.8)	119 (100.2;139.3)	11.1 (5.5;15.6)
50-69 years	1391.6 (1172.1;1627.5)	1330.4 (1125.6;1570.4)	-4.4 (-6.9;-1.8)
70+ years	8249.1 (6918.9;9752.5)	8530.2 (7265.9;9922.9)	3.4 (-1;8)
All Ages	448.5 (394.8;504.4)	796.1 (698.1;892.6)	77.5 (72.3;82.4)
***Male***			
Age-standardized	811.8 (714;916.9)	750.6 (656.2;845)	-7.5 (-10.2;-4.8)
Under 5	46.8 (32.2;64.5)	45.2 (31;62.1)	-3.6 (-7.2;0.4)
5-14 years	34.3 (23.7;46.9)	33.4 (23.1;45.9)	-2.5 (-6.6;1.7)
15-49 years	105.3 (89;122.4)	114.2 (95.6;134.7)	8.5 (0.5;14.2)
50-69 years	1386.9 (1164.2;1643.5)	1311.3 (1102.4;1555.5)	-5.4 (-9.1;-1.5)
70+ years	8083.9 (6784.9;9549.3)	7926.1 (6721.2;9286.2)	-2 (-6.4;2.9)
All Ages	415.6 (367.7;466.8)	685 (602.9;770)	64.8 (59.3;70.4)
***Female***			
Age-standardized	820.9 (721;933.2)	794.7 (694.4;900.6)	-3.2 (-6.5;-0.1)
Under 5	45.8 (31.6;63.3)	44.8 (30.7;63.3)	-2.1 (-5.7;2.2)
5-14 years	35.1 (24.7;47.9)	34.9 (24.3;47.2)	-0.7 (-4.6;3.1)
15-49 years	109 (91.7;126.6)	123.7 (103.9;144)	13.5 (8.8;18.3)
50-69 years	1395.9 (1183.9;1632.2)	1347.3 (1137.1;1586.4)	-3.5 (-6.9;0.3)
70+ years	8381.9 (7012.1;9982.4)	8968.9 (7622.9;10482.3)	7 (1.1;12.8)
All Ages	480.8 (422.3;544.4)	902.3 (790.2;1020.9)	87.7 (81;94.4)

*Source: Global Burden of Disease Study 2017, Institute for Health Metrics and Evaluation.^188^*

**Table 4-13:** – Number of YLDs and age-standardized rates of YLDs (per 100 000 inhabitants) due to heart failure from all causes, and percent change of rates, 1990 and 2017, in Brazil and its Federative Units

Location	1990		2017		Percent change (95% UI)
	Number (95% UI)	Rate (95% UI)	Number (95% UI)	Rate (95% UI)	
Brazil	88114.2 (64078.1;112623.9)	112.2 (82.8;141.2)	234168.9 (174338.9;291187.7)	108.8 (81.4;134.5)	-3 (-6.7;0.3)
Acre	188.7 (140.7;234.7)	123.8 (95.3;148.9)	636.8 (490.6;767.6)	119.8 (93.4;142.4)	-3.2 (-9.6;5.2)
Alagoas	1230.4 (880.5;1587.3)	97.7 (70.9;124.1)	2951 (2189.4;3685.1)	101 (75;125.8)	3.3 (-5.8;12)
Amapá	100.2 (73.7;127.2)	112.2 (84.5;139.2)	451.5 (337.3;558.9)	108.9 (83.4;132.6)	-3 (-9.5;5)
Amazonas	810.4 (592.2;1020.8)	115.9 (87.9;141.5)	2702.1 (2050;3305.1)	112.2 (86.1;135.8)	-3.1 (-10;3.9)
Bahia	6684.8 (4893.5;8540.9)	101.7 (75.5;129)	15736.9 (11812.7;19456)	100.8 (75.3;125.3)	-0.8 (-7.7;7.9)
Ceará	3969.6 (2897.9;5084.5)	99.2 (72.8;126.2)	10451.4 (7838.4;12882.6)	106.6 (79.8;132)	7.4 (-1.3;17)
Distrito Federal	431.1 (298.6;581.8)	97.6 (70.8;128.8)	1940.3 (1366.1;2560)	96.9 (69.9;125.4)	-0.8 (-8.1;9.2)
Espírito Santo	1462.1 (1054.4;1878.9)	113.4 (84.3;143.6)	4162.7 (3063.9;5243.2)	104.9 (77.5;131.9)	-7.5 (-14.4;0.1)
Goiás	1592.7 (1128;2094.6)	103.8 (76.7;132.4)	6291.2 (4685;7868.1)	106.1 (79.5;131.7)	2.2 (-6.5;11.5)
Maranhão	2190.7 (1560.1;2873.3)	92 (66.4;119.5)	5992.2 (4402.4;7553.5)	97.8 (72;122.9)	6.3 (-1.6;15.9)
Mato Grosso	725.4 (526.7;941)	113.1 (83.9;142.3)	3003 (2248.2;3728.7)	112 (84.7;137.3)	-0.9 (-8.1;7.7)
Mato Grosso do Sul	850.1 (609.1;1106.8)	113.3 (83.8;142.8)	2829.1 (2072.2;3549.4)	110.8 (82.1;137.7)	-2.2 (-9.5;6.5)
Minas Gerais	9477.1 (6808.4;12335.3)	110.9 (82.3;141.3)	25557 (18974.8;31992.6)	103.6 (77.2;129.4)	-6.5 (-14.4;1.6)
Pará	2221.3 (1651.1;2795.8)	115.7 (88.1;142.8)	6624.9 (5036.2;8052.8)	109.8 (84.8;132.1)	-5.2 (-12.2;2.3)
Paraíba	2298.3 (1666.9;2947.6)	99.5 (72.4;126.3)	4808 (3560.3;5995.7)	103.4 (76.3;129.4)	4 (-4.2;12.5)
Paraná	4964.3 (3563.3;6413.6)	123.5 (92.3;154.2)	13883.5 (10128.6;17249.1)	117 (86.8;144.4)	-5.2 (-12.7;3.3)
Pernambuco	4627.4 (3326;5865.3)	110.9 (81.9;137.8)	10375.1 (7650.4;12784)	109.6 (81;134.6)	-1.2 (-9;7.3)
Piauí	1230.3 (866.5;1647.8)	92.1 (65.6;122)	3402.3 (2478.9;4365.3)	95.1 (69.4;122.2)	3.3 (-5;11.9)
Rio de Janeiro	9922.7 (7229.9;12662.8)	119.7 (88.6;150.3)	22953.2 (16743.8;28786)	110 (81.3;137.7)	-8.1 (-14.7;-0.4)
Rio Grande do Norte	1606.1 (1145.6;2057.5)	99.5 (71.8;126.6)	3856.8 (2838.4;4879.3)	103.5 (75.7;132)	4 (-3.9;12)
Rio Grande do Sul	8134 (5994.6;10280.5)	140.5 (106.7;171.9)	18696.6 (14241.9;22612.8)	126.9 (97;152.5)	-9.7 (-16.6;-2.5)
Rondônia	300.3 (209.4;397.4)	117.4 (86.9;145.5)	1408.5 (1062.2;1750.7)	111.9 (85.8;137)	-4.7 (-11.7;4.4)
Roraima	46.3 (32.4;62.3)	101.5 (74.8;130.8)	276.8 (197.7;358.6)	98.7 (72.2;125.2)	-2.8 (-9.7;5)
Santa Catarina	2880 (2095.3;3688.1)	133.3 (99.4;165.6)	8433.4 (6334.3;10482.5)	119.7 (90.8;147.4)	-10.2 (-16.8;-3.4)
São Paulo	18988.5 (13523.4;24849.5)	112.8 (81.9;144.5)	53360.6 (38622.8;68602.6)	109.6 (80.1;140.1)	-2.9 (-10.6;5.1)
Sergipe	853.9 (627.3;1082.5)	102.4 (75.6;129)	2085.5 (1551.2;2578.3)	103.7 (77.4;127.2)	1.3 (-6.3;9.9)
Tocantins	327.5 (226.3;438.8)	96.4 (69.6;126.2)	1298.6 (952.1;1635.1)	100.3 (73.9;125.9)	4 (-4.3;14.5)

*Source: Global Burden of Disease Study 2017, Institute for Health Metrics and Evaluation.^188^*

**Table 4-14:** – Rates of YLDs due to heart failure (per 100 000 inhabitants) and percent change of rates, by age and sex, Brazil, 1990 and 2017

**Age group**	**1990**	**2017**	**Percent Change (95% UI)**
***Both sexes***			
Age-standardized	112.2 (82.8;141.2)	108.8 (81.4;134.5)	-3 (-6.7;0.3)
Under 5	4.5 (2.7;7)	4.3 (2.6;6.8)	-3.4 (-6;-0.6)
5-14 years	3.2 (1.9;5.1)	3.2 (1.9;5)	-1.4 (-4.4;1.5)
15-49 years	8.7 (5.7;12.6)	10.4 (6.8;14.8)	18.7 (12.3;25.5)
50-69 years	165.8 (112.7;226.8)	166.4 (115.3;228.9)	0.3 (-3.8;5.5)
70+ years	1263.1 (919.3;1599.5)	1308.3 (988.6;1586)	3.6 (-3.2;10.2)
All Ages	59 (42.9;75.4)	110.6 (82.3;137.5)	87.5 (78.8;96.2)
***Male***			
Age-standardized	112.9 (86.9;137.4)	105.2 (81.6;127.1)	-6.8 (-10.9;-2.6)
Under 5	4.5 (2.7;7.1)	4.3 (2.6;6.8)	-4.2 (-7.8;-0.2)
5-14 years	3.2 (1.9;5)	3.1 (1.8;4.9)	-2.2 (-6.4;2)
15-49 years	7.9 (5.1;11.6)	9.5 (6.1;13.8)	19.8 (10.2;29.3)
50-69 years	165.5 (111.9;230.9)	165.8 (113.5;229.4)	0.2 (-5.9;6.8)
70+ years	1282.5 (973.9;1555.8)	1225.7 (980;1439.6)	-4.4 (-11.1;3.4)
All Ages	55 (40.6;68.5)	94.5 (72.6;115.7)	71.9 (63.1;81.5)
***Female***			
Age-standardized	111.2 (79.9;145)	110.9 (80.1;140.6)	-0.3 (-4.9;4.2)
Under 5	4.4 (2.7;7)	4.3 (2.6;6.7)	-2.6 (-6.3;1.7)
5-14 years	3.2 (1.9;5.2)	3.2 (1.9;5.1)	-0.6 (-4.5;3.2)
15-49 years	9.5 (6.3;13.4)	11.2 (7.4;15.9)	17.9 (10.5;26.4)
50-69 years	166.1 (112.5;225.8)	166.9 (116.6;228.9)	0.5 (-5;6.7)
70+ years	1247.5 (876.2;1659.2)	1368.3 (992.5;1714.5)	9.7 (0.6;17.6)
All Ages	62.9 (44.1;82.5)	125.9 (90.9;159.5)	100.2 (88.8;111.5)

*Source: Global Burden of Disease Study 2017, Institute for Health Metrics and Evaluation.^188^*

**Table 5-1:** – Age-standardized prevalence rates of valvular heart disease per 100 000, for both sexes and for males and females, in Brazil and its Federative Units, 1990 and 2017, and percent change of rates

Cause of death and age group	Both sexes			Female			Male		
	1990	2017	Percent change (95% UI)	1990	2017	Percent change (95% UI)	1990	2017	Percent change (95% UI)
***B.2.1 - Rheumatic heart disease***									
**Acre**	703.8 (665.8;739.9)	729.7 (693.1;768.5)	3.7 (7.7;-0.3)	775 (730.9;820.8)	815.5 (768.6;865.4)	5.2 (11.2;0.2)	637.6 (602;673.8)	643.2 (608.9;683.8)	0.9 (6.3;-4.6)
**Alagoas**	716 (680.1;754.7)	742.7 (704.3;784.6)	3.7 (8.3;-0.2)	792.6 (748;840.8)	831.5 (783;883.6)	4.9 (11.4;-0.9)	633.3 (596;671.4)	644.2 (604.8;683.5)	1.7 (8.5;-4.2)
**Amapá**	717.6 (682.8;756.8)	738.1 (699;779.4)	2.9 (7;-1.2)	788 (746;835.3)	825.5 (778.8;872.7)	4.8 (10.6;-0.4)	647.3 (612.4;686.3)	649.1 (611.5;689.5)	0.3 (6;-5.3)
**Amazonas**	725.7 (690.9;766)	745.4 (701.9;787.1)	2.7 (6.6;-1.4)	806.5 (761.8;856.7)	837.9 (785.6;893.9)	3.9 (9.7;-2.3)	646.3 (609.2;688.4)	652.9 (614.4;690.3)	1 (7.2;-5.6)
**Bahia**	698.3 (664.2;739.1)	726.2 (689.5;765.3)	4 (7.7;-0.1)	764.8 (722.7;814.7)	809.5 (763.4;859.3)	5.8 (11;0.6)	627.7 (589.6;669.6)	637.3 (602.1;676.4)	1.5 (7.7;-4.5)
**Brazil**	721.4 (688.7;754.5)	743.2 (709;778.6)	3 (4.3;1.6)	797 (758.9;835.7)	832.2 (793.3;871.2)	4.4 (6.1;2.6)	641.9 (611.4;672.8)	648.9 (619;682.4)	1.1 (2.8;-0.7)
**Ceará**	721.8 (682.3;759.9)	747.5 (708.2;785.5)	3.6 (7.4;0)	798.7 (753;847.5)	838 (787.6;887.3)	4.9 (10.2;-1)	636.7 (596.7;676)	649.9 (611.1;688.1)	2.1 (7.8;-3.5)
**Distrito Federal**	727.1 (689.8;767.7)	744.2 (705.1;781.2)	2.3 (6.5;-1.6)	802.3 (758.5;851.6)	831.2 (783.8;881.5)	3.6 (9.6;-1.8)	643.2 (607.9;683.5)	646.7 (609.8;685.7)	0.5 (6.4;-5.4)
**Espírito Santo**	723.1 (686.9;761.9)	743.5 (705.8;783.6)	2.8 (7.2;-1.2)	802.6 (759.6;849.1)	834.1 (785;885.2)	3.9 (9.9;-1.8)	642.2 (602.5;682.4)	648.9 (611;692.3)	1 (7.6;-4.8)
**Goiás**	712.2 (676.2;749.8)	731.3 (695.6;769.8)	2.7 (6.3;-1.3)	785 (744.4;829.9)	818.3 (772.2;869.3)	4.2 (10.6;-1.6)	640.6 (605.3;678.1)	641.7 (605.2;680.2)	0.2 (6;-5.5)
**Maranhão**	697.8 (662.7;735.1)	723.3 (686.3;761.1)	3.7 (8.2;0.2)	763.4 (720.7;810.6)	808.1 (761.7;855.2)	5.9 (13;0.7)	629.9 (594.6;666.9)	634.3 (598.3;671.6)	0.7 (6.9;-5.1)
**Mato Grosso**	718.2 (679.8;759.1)	741.6 (703.4;783)	3.3 (7.8;-1.2)	803.2 (754.4;856.1)	835.7 (789.3;887)	4 (10.1;-2.3)	642.9 (604;683.2)	650.5 (613.5;691.9)	1.2 (7.8;-4.9)
**Mato Grosso do Sul**	721.6 (682.2;762.9)	743.9 (707;786.6)	3.1 (7.1;-1)	801.8 (755.3;852.3)	836.1 (787.1;887.1)	4.3 (10.1;-1.4)	644.1 (604.1;685.3)	649.6 (613.7;693)	0.9 (7.1;-5.4)
**Minas Gerais**	724.4 (685;763.2)	746.6 (707;789.2)	3.1 (7.3;-0.8)	801.8 (752.5;848.6)	838 (791;889.4)	4.5 (10.1;-0.9)	643.6 (607;682.9)	651.3 (611.4;692.4)	1.2 (7.5;-5.5)
**Pará**	697.5 (659.7;733.9)	725.6 (687.9;766.2)	4 (7.8;0.4)	764.8 (718.8;811.3)	812.4 (764.3;861.1)	6.2 (13.1;0.3)	632 (596.3;670.2)	638.8 (601.1;676.1)	1.1 (6.7;-4.3)
**Paraíba**	722.5 (685.6;764.3)	745.9 (708;784.5)	3.2 (7.7;-0.7)	798.1 (752.6;848.6)	835.4 (788.5;885.1)	4.7 (11.6;-0.4)	637.5 (601.1;676.8)	648.6 (608.5;687.3)	1.7 (7.7;-3.9)
**Paraná**	726.1 (688.1;765.9)	749 (710.7;789)	3.2 (7.1;-0.8)	805.2 (761.2;853.1)	839.6 (789.9;892.7)	4.3 (10.3;-1.9)	645.8 (608.5;686.5)	653.7 (616.8;695.5)	1.2 (7;-4.8)
**Pernambuco**	698 (662.3;735)	725.4 (688.9;764.7)	3.9 (8.4;0.2)	765.2 (722.9;809.1)	807.6 (762;853.5)	5.6 (11.7;0)	622.3 (583.7;661.9)	634.3 (597.7;673.3)	1.9 (7.9;-3.3)
**Piauí**	695.7 (660.7;733)	728.9 (691.5;769.8)	4.8 (9.2;0.6)	760.9 (719.6;805.7)	812.4 (765.2;862.7)	6.8 (13.6;0.6)	625.6 (591.2;663.8)	639.7 (605.1;678.2)	2.3 (8.4;-3.5)
**Rio de Janeiro**	724 (687.3;761.5)	744.2 (706;786.6)	2.8 (6.7;-1.2)	798.6 (752.6;845.5)	832.1 (784.7;881)	4.2 (10.3;-2.1)	641 (605.8;678.1)	647.6 (609.7;690.4)	1 (7.3;-5.1)
**Rio Grande do Norte**	728.2 (691.7;770.1)	748.7 (710;789.2)	2.8 (7;-1.5)	807.1 (762.2;859.6)	840.3 (789.7;889.8)	4.1 (10.7;-2.5)	641.6 (603;682.9)	650.7 (611.7;691.2)	1.4 (7.9;-4.9)
**Rio Grande do Sul**	736 (696.2;776.3)	751.4 (711.4;791)	2.1 (6.2;-2.1)	814.7 (768.3;865.5)	842.7 (791.8;893.9)	3.4 (10.5;-2.6)	652.1 (609.9;690.5)	654.9 (615.9;693.4)	0.4 (6.3;-5.9)
**Rondônia**	715.4 (677.7;753.8)	739.2 (700.9;778.6)	3.3 (7.5;-0.1)	801.5 (755.8;853.8)	833 (785.4;884.3)	3.9 (10.5;-2)	640.7 (601.2;677.1)	647.5 (608.9;685.7)	1.1 (6.9;-4.3)
**Roraima**	708.8 (672.4;748.4)	737.8 (697.7;780.1)	4.1 (8.6;0)	798.3 (751.2;847.9)	831.4 (782.3;881.2)	4.1 (11;-2.3)	641.5 (604.8;680.2)	648.6 (610.6;689.5)	1.1 (7.1;-4.4)
**Santa Catarina**	738 (700.8;775.2)	756.4 (714.4;799.4)	2.5 (6.3;-0.9)	819.4 (771.8;869.5)	849.2 (797.9;900.5)	3.6 (9.8;-1.9)	655.2 (616;694.9)	661.3 (623.1;702.9)	0.9 (7.2;-4.3)
**São Paulo**	733.8 (694.9;775.3)	753.5 (713;794.1)	2.7 (6.6;-1.4)	813.1 (765.5;864.3)	844.5 (793.5;897.4)	3.9 (9.9;-1.9)	650.5 (611.9;690)	656.5 (615.6;697.4)	0.9 (7.4;-5)
**Sergipe**	709.6 (673;747.4)	733.5 (695.4;772.8)	3.4 (7.9;-1.2)	776.6 (733.6;822)	816 (769.1;870.3)	5.1 (11.1;-0.7)	636.9 (599.1;673.6)	642.7 (606.2;680.8)	0.9 (7.8;-5.4)
**Tocantins**	694.9 (660.5;733.7)	726.2 (688.6;767.1)	4.5 (8.4;0.7)	763.1 (720.4;810.2)	813.3 (767.8;861.3)	6.6 (12.5;1.4)	631.9 (596.2;668.5)	640.4 (600.7;679.6)	1.4 (6.8;-3.8)
***B.2.5 - Non-rheumatic valvular heart disease***									
**Acre**	205.7 (195;216.6)	224.6 (212.8;236.5)	9.2 (15.3;3.5)	204.6 (191.3;218.3)	222.1 (207.8;236)	8.6 (17.7;0.3)	206.4 (192.7;219.8)	227.3 (212.2;242.4)	10.2 (18.6;2.2)
**Alagoas**	196.1 (185.1;207.4)	217.3 (205.8;228.9)	10.8 (17.5;4.6)	196.3 (183;210.4)	215 (200.9;228.9)	9.5 (18.8;1.4)	196.1 (181.9;211.2)	220.6 (205.6;236.6)	12.5 (22.1;3.6)
**Amapá**	215 (204.2;227)	232 (218.9;243.8)	7.9 (13.9;2.2)	213.9 (200.3;228.3)	228.1 (213.2;242.2)	6.7 (15.1;-1.6)	216.6 (202.3;232)	236.6 (220.5;252.2)	9.3 (18.6;1)
**Amazonas**	212.8 (201.5;223.1)	231.9 (219.4;244.2)	9 (15.1;3)	210.1 (196.7;223.6)	227.8 (212.6;242.9)	8.4 (17.1;0.2)	215.8 (201.1;229.7)	236.5 (219.9;252.6)	9.6 (18.2;1.5)
**Bahia**	203.9 (192.4;214.8)	221.9 (210;234.1)	8.9 (15.5;2.6)	202.4 (187.9;216.5)	219 (204.8;234.2)	8.2 (17.9;-0.8)	205.7 (191.5;219.8)	226 (211;241.8)	9.9 (18.8;1.7)
**Brazil**	216.2 (207.9;224.6)	233.9 (224.6;243.2)	8.2 (10.5;6.2)	214.2 (205.6;222.8)	231 (221.7;240.5)	7.8 (10.8;5.1)	218.7 (209.3;228.2)	238.1 (227.4;249.1)	8.9 (12.1;5.8)
**Ceará**	199.9 (188.5;211.5)	220.7 (208.5;232.9)	10.4 (17.2;4.2)	200 (187;213.9)	219.1 (205;233.9)	9.5 (19.2;1.3)	199.9 (184.7;215.4)	223.3 (207.3;237.7)	11.7 (21.3;2.9)
**Distrito Federal**	240.9 (227.1;253.5)	258.6 (245.2;271.6)	7.3 (13.1;1.7)	237.1 (221.3;252.1)	254.1 (237.7;270.7)	7.2 (15.7;-0.9)	245.8 (228.3;263.5)	265.5 (248;282.5)	8 (16.6;0.2)
**Espírito Santo**	220.3 (209.2;233)	236.8 (225.2;250)	7.5 (13.5;1.8)	218 (204.7;233)	233.5 (218.6;248.8)	7.1 (16.2;-1.2)	222.9 (209.1;238.9)	241.2 (225.8;257.4)	8.2 (16.8;0)
**Goiás**	212.7 (201.9;224.4)	229.7 (217.4;242)	8 (14.4;2.2)	212.7 (197.9;227.3)	227.2 (212.7;241.9)	6.8 (15.8;-1.3)	212.6 (198.6;227.5)	232.8 (216.2;248.7)	9.5 (18.5;1.1)
**Maranhão**	195.1 (184.6;205.8)	214.5 (202.2;227.9)	9.9 (16.4;3.9)	194.9 (182.5;208.7)	211.6 (197;225.9)	8.6 (17.4;0.3)	195.7 (181.7;209)	218.1 (203.1;233.7)	11.4 (21.2;2.3)
**Mato Grosso**	214.7 (203.5;226)	233.1 (220.6;245.6)	8.6 (15.4;2.5)	212.6 (198.1;226.8)	229.7 (215;245)	8.1 (17.4;-0.4)	216.3 (202.2;231.3)	236.5 (220.9;252.5)	9.3 (18.2;1.2)
**Mato Grosso do Sul**	216.7 (204.9;228.2)	233.5 (220.7;246)	7.8 (14.1;1.8)	214 (199.5;227.8)	229.7 (214.8;244.4)	7.4 (16.6;-0.6)	219.1 (203.9;233.4)	238 (222.4;255.5)	8.6 (17.5;0.1)
**Minas Gerais**	214.7 (203.5;226.9)	232 (220.1;244.2)	8 (14.2;2.1)	212.3 (199.1;227)	228.8 (214.3;243.2)	7.8 (16.3;-0.6)	217.9 (203.8;233.2)	236.2 (220.7;253.4)	8.4 (17;0.1)
**Pará**	201.6 (191;213)	221.3 (209.1;233.7)	9.8 (16;3.7)	200.7 (187.3;215.1)	218 (202.3;232.6)	8.7 (17.9;0.4)	202.9 (189.7;217.7)	225 (210.3;240.2)	10.9 (19.4;2.5)
**Paraíba**	199.4 (188.2;210.9)	219.5 (207.7;231.3)	10.1 (16.1;4)	197.7 (183.8;211.4)	217.2 (203.3;232)	9.8 (19.2;1.6)	201.2 (186.2;216.6)	222.9 (207;238.5)	10.8 (19.7;2.8)
**Paraná**	223.8 (211.7;236.2)	239.7 (228;253)	7.1 (13.1;1.3)	220.7 (207.1;235.2)	236.4 (222;251.8)	7.1 (16.1;-0.8)	227.1 (212.7;243)	244.1 (228.7;261.2)	7.5 (15.9;-0.7)
**Pernambuco**	204.2 (193.4;215.1)	223 (209.8;235.2)	9.2 (15.5;3.2)	203.7 (190.6;217.2)	221.1 (206.2;236.5)	8.5 (17.1;0.3)	205 (191.8;220.3)	226.3 (210.5;241.5)	10.4 (20.1;2.5)
**Piauí**	197.2 (186.5;208)	215.7 (204.1;228)	9.4 (16;3)	196.4 (183.2;208.8)	213.2 (199.2;227.8)	8.5 (16.9;0.4)	198.3 (184;212.8)	219 (204.6;235.2)	10.5 (20.3;1.6)
**Rio de Janeiro**	220.5 (209.3;232.1)	238.3 (224.6;250.7)	8.1 (14.4;2)	219 (204.4;233.4)	235.5 (219.4;250.4)	7.5 (16.6;-1.2)	223.5 (209.1;239)	243.1 (227.6;261)	8.8 (17.2;0.8)
**Rio Grande do Norte**	203.5 (193;214.7)	223.7 (211.6;235.9)	9.9 (16.1;3.6)	202.9 (188.7;216.4)	221.3 (206;235.7)	9.1 (18.1;0.7)	204.2 (190.3;218.4)	227.3 (212.4;243.5)	11.3 (20.2;3.2)
**Rio Grande do Sul**	227.5 (215.2;239.3)	241.9 (229.2;254.8)	6.3 (12.6;0.6)	224.4 (208.7;239.5)	237.8 (223;253.3)	6 (14.5;-2)	232.2 (216.5;247.3)	247.8 (231.7;265.4)	6.7 (15.7;-0.9)
**Rondônia**	209.2 (197.9;220.7)	227.1 (215;239.8)	8.6 (15.4;2.6)	205 (190.9;219)	223.9 (208.6;239.1)	9.2 (18.7;0.9)	212.3 (197.7;226.5)	230.2 (214.3;246.5)	8.4 (17.7;0.1)
**Roraima**	212 (200;223.9)	229.5 (217;242.8)	8.3 (14.4;2.4)	212.3 (198.8;227.8)	226.9 (211.8;242.6)	6.9 (15.5;-1.2)	211.9 (197.6;226.9)	231.9 (216.3;247.8)	9.4 (18.6;1.3)
**Santa Catarina**	228.3 (215.6;240.1)	242.5 (229.2;256.5)	6.2 (11.8;0.5)	225.6 (210.8;239.8)	238.7 (223.3;255.3)	5.8 (14.4;-1.9)	231.7 (216.6;248.1)	247.8 (230.8;264.6)	6.9 (15.7;-1.1)
**São Paulo**	229.8 (217.3;241.6)	244.9 (232.6;258.6)	6.6 (12.6;1.1)	226.1 (211;239.7)	241.7 (225.7;257.7)	6.9 (15.2;-0.9)	234.9 (219.1;251.4)	250 (232.9;267.1)	6.4 (15.1;-1.3)
**Sergipe**	201.1 (190.2;212.8)	223.1 (210.6;235.4)	11 (17.7;5)	201.1 (187.9;215.2)	220.7 (205.4;235.6)	9.7 (18.3;1.2)	201.2 (187.4;215.7)	226.7 (211.9;242.3)	12.6 (21.7;3.9)
**Tocantins**	206.2 (194.6;217)	223.1 (210.7;236)	8.2 (14.6;2)	204.3 (190.8;218.4)	219.4 (203.7;234.3)	7.4 (16.5;-1.2)	207.8 (193.3;221.3)	226.6 (210.1;242.4)	9 (17.8;1.2)
***B.2.5.1 - Non-rheumatic calcific aortic valve disease***									
**Acre**	49.8 (44.5;55.8)	61.3 (54.1;68.9)	23.1 (31.2;16)	40.7 (36;45.6)	51.9 (45.5;58.4)	27.5 (38.3;18.7)	57.6 (51.3;64.6)	71.1 (62.4;80.2)	23.4 (34.1;14)
**Alagoas**	43.4 (38.7;49.1)	58.1 (51;66.2)	34 (42.5;26.3)	36.7 (32.4;41.7)	50 (44.1;57.1)	36.2 (46.5;26.7)	50.9 (45;57.6)	68.2 (59.6;77.9)	34 (46.2;23.8)
**Amapá**	54.6 (48.7;61.6)	65.6 (58.2;74.2)	20 (27.4;13.1)	45.3 (40.3;51.1)	54.7 (48.1;61.5)	20.7 (30.6;12.2)	64.6 (57.1;72.7)	77.6 (68.8;87.9)	20.2 (30.1;11.4)
**Amazonas**	51.3 (45.9;58)	63.9 (56.7;72)	24.5 (32.1;17.2)	41 (36.3;46.4)	53.1 (46.5;60.3)	29.4 (39.9;20.2)	62 (55.2;70.2)	75.4 (66.5;85.8)	21.7 (31.1;12.9)
**Bahia**	48.3 (43.1;54.2)	59 (52.4;67)	22.4 (30.1;15.3)	40.4 (35.8;45.5)	50.1 (43.8;57)	24.2 (35;16)	57.3 (50.9;65.1)	70 (61.5;79.3)	22.2 (31.7;13.3)
**Brazil**	53.5 (48.1;59.9)	64.4 (57.2;72.5)	20.4 (25.4;16)	44.2 (39.8;49.6)	54.9 (48.7;62)	24.2 (29.7;19.1)	64.3 (57.5;72)	76.2 (67.3;85.6)	18.5 (24.2;13.7)
**Ceará**	46 (40.9;52.2)	59.6 (52.5;67.7)	29.4 (37;22.2)	38.7 (33.9;44.3)	51 (44.8;58)	31.6 (40.6;22.4)	54.3 (47.8;62)	70.2 (61;80.2)	29.3 (39.1;19.6)
**Distrito Federal**	63.1 (56.5;70.5)	73.3 (64.7;82.1)	16.2 (23.5;9.5)	51.3 (45.7;58)	62.5 (55;70.6)	21.8 (32;12.6)	77.4 (68.7;87.1)	88.2 (77.6;99.8)	13.9 (22.5;5.8)
**Espírito Santo**	55.5 (49.5;61.9)	64.9 (57.5;73.3)	17.1 (23.9;10.8)	45.5 (40.4;51.3)	55 (48.4;62.4)	20.7 (29.8;12.5)	66.3 (58.9;74.6)	76.9 (67.6;86.9)	16 (24.5;7.8)
**Goiás**	52.9 (46.9;59.3)	62.4 (55;70.1)	18.1 (25.3;11.4)	44.8 (39.7;50.6)	52.9 (46.6;59.4)	18.2 (27.4;10.7)	60.5 (53.5;67.8)	73.1 (64.3;83)	20.8 (30.4;11.5)
**Maranhão**	43.9 (38.9;49.6)	57 (50.1;64.9)	29.9 (37.2;22.8)	36.9 (32.3;42.1)	48.5 (42.5;55.5)	31.3 (41.2;22.2)	51.8 (45.9;58.7)	66.8 (58.8;76.5)	29 (38.5;19.4)
**Mato Grosso**	52.4 (46.7;58.7)	64.1 (56.6;72.4)	22.4 (30;15.2)	41.8 (37.4;47.1)	53.8 (47.6;61)	28.6 (38.8;19.9)	61.3 (54.3;68.9)	74.3 (65.2;84.3)	21.2 (31.3;11.9)
**Mato Grosso do Sul**	55.3 (49.5;62)	65.6 (58.3;74.3)	18.7 (26.3;11.8)	45.4 (40.4;51.1)	55.4 (48.6;62.5)	21.9 (31;13.7)	64.5 (57.2;72.6)	77 (67.9;87.4)	19.4 (29.9;9.9)
**Minas Gerais**	51.9 (46.5;58.3)	63.1 (55.9;71.4)	21.6 (29.2;14.7)	42.7 (38.1;48.1)	53.5 (47.2;60.6)	25.4 (34.1;18)	62.7 (55.9;70.9)	74.7 (65.7;84.8)	19 (28.9;9.5)
**Pará**	46.9 (41.7;53)	60 (53.2;67.4)	27.8 (35.1;20.3)	38.8 (34.1;44)	50.2 (43.9;57.3)	29.4 (39.6;20.8)	55.7 (49.5;63.2)	70.4 (62;79.3)	26.4 (35.8;16.2)
**Paraíba**	46.3 (40.4;52.6)	58.8 (51.8;66.9)	26.9 (33.9;21)	38.2 (33.8;43.5)	50.2 (43.8;57.5)	31.2 (41.8;21.7)	55.5 (47.5;63.8)	69.8 (60.8;79.5)	25.7 (36.4;16.8)
**Paraná**	58.8 (52.7;66.1)	67.9 (59.9;76.5)	15.5 (23.5;8.6)	48.4 (42.9;54.4)	57.7 (50.5;65.4)	19.4 (28.9;10.9)	69.6 (62.3;78.2)	80.1 (70.3;90.4)	15.1 (25.8;6.7)
**Pernambuco**	47 (41.9;53.1)	60.2 (53;68)	28.2 (35.5;20.8)	39.2 (34.7;44.1)	51.9 (45.4;58.5)	32.2 (42.2;22.9)	56.5 (50.1;63.9)	71.4 (62.5;80.8)	26.5 (36.8;16.7)
**Piauí**	45.8 (40.5;51.5)	57.8 (51;66)	26.2 (34.8;18.5)	38.1 (33.2;43.4)	49.1 (43;56.1)	28.9 (38.8;20.1)	54.5 (48.4;61.4)	68.2 (59.9;78)	25.1 (36.2;15.5)
**Rio de Janeiro**	53.1 (47.6;59.6)	64.6 (57.1;72.9)	21.6 (29;14.8)	44.3 (39.5;49.6)	55.5 (48.9;62.9)	25.4 (34.9;16.2)	65.2 (57.8;73.7)	77.1 (67.7;87.3)	18.2 (29;8.7)
**Rio Grande do Norte**	47.8 (42.7;53.9)	61.4 (54.2;69.4)	28.3 (35.9;21.6)	39.6 (35.1;45)	52.3 (45.9;59.4)	31.9 (42.5;23)	57 (50.5;64.3)	72.8 (64.1;82.2)	27.7 (36.8;18.8)
**Rio Grande do Sul**	60.4 (54;67.4)	68.6 (60.3;77.2)	13.5 (21.4;6.7)	50.2 (44.6;56.4)	58.5 (51.3;66.5)	16.6 (26.8;7.5)	73.6 (65.3;82.2)	81.6 (71.7;92.5)	10.8 (20.8;2.1)
**Rondônia**	51.4 (45.7;57.8)	62.6 (55.5;70.1)	21.9 (29.6;14.9)	40 (35.4;45.4)	52.2 (45.7;59)	30.4 (40.4;20.9)	60.4 (53.7;67.8)	72.8 (64.3;81.9)	20.4 (30.1;11.2)
**Roraima**	53.5 (47.5;59.9)	63.7 (56.3;71.7)	19.1 (26.6;12.4)	45.4 (40.2;51.2)	54.4 (47.7;61.5)	19.7 (29.3;10.8)	59.7 (52.8;67.2)	72.3 (63.9;81.6)	21.2 (30.9;12.1)
**Santa Catarina**	61.1 (54.5;68.6)	69 (60.8;77.5)	13.1 (20.6;6.4)	50.9 (44.9;57.7)	58.8 (51.3;66.5)	15.5 (26.3;6.6)	72.7 (64.4;82.2)	81.5 (71.7;92)	12.2 (21.6;4.5)
**São Paulo**	60.2 (53.8;67.5)	68.8 (60.9;77.4)	14.3 (22.1;7.9)	49.1 (43.6;55.2)	58.7 (51.8;66.3)	19.7 (28.7;11)	73.7 (65;83.2)	82 (72.5;92.5)	11.2 (20.4;3.3)
**Sergipe**	44.7 (39.3;50.9)	59.2 (52.1;67.4)	32.5 (40;25.8)	37.5 (32.7;43)	50.6 (44.6;58.2)	34.8 (45.1;26.2)	53.1 (46.5;60.8)	70.1 (61.2;80.1)	32 (42.5;23.5)
**Tocantins**	49.8 (44.3;56)	61.4 (54.7;69.2)	23.3 (30.7;16.4)	40.6 (35.6;46.1)	51.2 (45.4;57.8)	26.2 (36.3;18)	58.2 (51.7;65.8)	71.2 (62.5;80.6)	22.2 (31.6;13.3)

*Source: Global Burden of Disease Study 2017, Institute for Health Metrics and Evaluation.^236^*

**Table 5-2:** – Death and DALY rates per 100 000 and percent change of rates, by age and cause of death, in Brazil, 1990 and 2017

Cause of death and age group	1990	2017	Percent change (95% UI)	1990					2017	Percent change (95% UI)	
**Death (age-standardized)**				**DALY (age-standardized)**							
***B.2.1- Rheumatic heart disease***											
**15-49 years**	1.4 (1.3;1.4)	0.6 (0.6;0.6)	-56.5 (-59.3;-52.9)	121.8 (104.9;143.4)					79.3 (62.4;101.4)	-34.9 (-41.7;-28.1)	
**50-69 years**	4.8 (4.6;5)	2.6 (2.5;2.8)	-45.1 (-48.2;-41.2)	179.7 (164.5;198.4)					117.5 (102.8;136.9)	-34.6 (-39;-30.1)	
**5-14 years**	0.7 (0.6;0.8)	0.2 (0.2;0.2)	-69.8 (-72.4;-65.6)	71.3 (61.1;80.6)					34.7 (28;43.5)	-51.4 (-58;-43.6)	
**70+ years**	11.4 (11;11.7)	7.5 (7.2;7.8)	-33.9 (-37.1;-30.3)	174.2 (165.3;184.2)					113.8 (105.3;123.7)	-34.7 (-38.1;-31.1)	
**Age-standardized**	2.4 (2.3;2.5)	1.2 (1.1;1.2)	-50.3 (-52.4;-47.5)	118.9 (106.1;134.7)					71.2 (59;87.2)	-40.1 (-45.4;-34.6)	
**All ages**	1.8 (1.7;1.8)	1.3 (1.2;1.3)	-28.5 (-31.7;-24.3)	109.3 (97;124.6)					76.5 (63.3;93.7)	-30 (-36.3;-23.9)	
**Under 5**	0.6 (0.5;0.8)	0.1 (0.1;0.1)	-82.5 (-87.9;-74.4)	52.8 (40.6;68.7)					11 (9.3;13.1)	-79.1 (-85.3;-70.4)	
***B.2.5- Non-rheumatic valvular heart disease***											
**15-49 years**	0.5 (0.4;0.5)	0.4 (0.4;0.5)	-23.5 (-30;1.2)		27 (21.7;28.8)		20 (18.5;24.3)				-26.1 (-32.2;-1.6)
**50-69 years**	3.5 (2.9;3.7)	3.3 (2.6;3.4)	-6.9 (-14.5;-1.6)		104.1 (86.8;111.4)		94.9 (78.6;101.4)				-8.9 (-15.4;-3.8)
**70+ years**	13.1 (11.7;15.1)	19 (14;20.2)	45.6 (10;57.1)		201 (179.1;230.6)		254.6 (194.7;280)				26.7 (1.3;35.5)
**Age-standardized**	1.7 (1.5;1.9)	1.8 (1.4;1.9)	7.1 (-9.1;13)		42.9 (36.6;45.8)		39.4 (33.2;42.2)				-8 (-15.6;-4)
**All ages**	1 (0.9;1.1)	1.9 (1.5;2)	87.5 (63.5;96.9)		30.5 (25.9;32.5)		42.3 (35.8;45.3)				38.8 (27.5;47.2)
***B.2.5.1- Non-rheumatic calcific aortic valve disease***											
**15-49 years**	0.3 (0.2;0.3)	0.2 (0.2;0.3)	-18.6 (-30.8;37.4)			14.3 (9.8;15.8)		11.3 (10.2;15.3)			-21.2 (-32.9;33.7)
**50-69 years**	2.2 (1.6;2.4)	2.1 (1.9;2.5)	-4.1 (-14.1;25.2)			64.6 (47.3;70.3)		60.6 (54.6;71.2)			-6.2 (-16;23.2)
**70+ years**	9.1 (7.5;9.9)	14.4 (11.1;15.6)	57.4 (35.7;73.3)			127.3 (102.9;138.2)		173.9 (136.8;188.7)			36.6 (22.9;52.4)
**Age-standardized**	1.1 (0.9;1.2)	1.3 (1.1;1.4)	16.3 (9.6;34)			25.4 (19.1;27.4)		25.1 (22.1;28.7)			-1.2 (-9.1;24.2)
**All ages**	0.6 (0.5;0.7)	1.3 (1.1;1.5)	108.3 (94.8;146.2)			17.7 (13;19.2)		26.9 (23.9;31)			51.5 (38.3;94.9)
***B.2.5.2- Non-rheumatic degenerative mitral valve disease***											
**15-49 years**	0.2 (0.2;0.3)	0.2 (0.1;0.2)	-31.4 (-49.9;-22.8)		11.8 (9.8;14.5)		7.8 (5.6;8.7)				-33.9 (-51.3;-25.7)
**50-69 years**	1.2 (1;1.6)	1 (0.6;1.2)	-12.7 (-47.4;3)		37.6 (32.6;49.1)		32.4 (19.6;36.8)				-14 (-44;-0.2)
**70+ years**	3.7 (3.3;5.3)	4.3 (2.2;5.2)	17.7 (-42.1;39.2)		70.5 (57.7;92.6)		77.4 (50.2;94.7)				9.8 (-31.8;24.3)
**Age-standardized**	0.5 (0.5;0.7)	0.5 (0.3;0.5)	-10.8 (-47.6;2.4)		16.4 (14.4;20.8)		13.4 (8.8;15.2)				-18.7 (-43.4;-9.3)
**All ages**	0.3 (0.3;0.5)	0.5 (0.3;0.6)	50.1 (-9.7;71.8)		12 (10.4;15.1)		14.4 (9.5;16.4)				20.3 (-15.9;35.4)

*Source: Global Burden of Disease Study 2017, Institute for Health Metrics and Evaluation .^236^*

## Overview

- Noncommunicable diseases comprise the world’s leading group of causes of death, responsible for premature deaths, loss of quality of life, and adverse economic and social impacts. The NCDs are responsible for approximately 70% of global deaths, equivalent to more than 38 million deaths annually, significantly exceeding deaths from external causes and infectious diseases.^28 -^^31^Nearly 45% of all deaths from NCDs worldwide, over 17 million, result from CVDs. The same happens in Brazil, where 72% of deaths result from NCDs, 30% being due to CVDs, 16% to neoplasms, and 6% to respiratory diseases.^32 - 34^
- The definition of CVD may vary according to the study, from including all diseases listed in ICD-10 Chapter IX to just grouping together the 3 main causes (IHD, stroke, and HF). For the GBD, the definition of total CVDs comprises 10 causes: rheumatic heart disease, IHD, cerebrovascular disease, hypertensive heart disease, cardiomyopathy, myocarditis, atrial fibrillation and flutter, aortic aneurysm, peripheral vascular disease, and endocarditis.^35^
- Cardiovascular diseases were the number 1 cause of death in Brazil in 1990 and 2017 (Chart 1-1). According to the GBD Study 2017 estimates, of the CVDs, IHD was the leading cause of death in the country, followed by stroke, in 1990 and 2017 (Chart 1-2). In fact, in 2017, IHD was the leading cause of death in all Brazilian FUs, although, in 1990, stroke was still the number 1 cause of death in the states of Alagoas and Sergipe (Charts 1-3 and 1-4).

## Prevalence

- Gonçalves *et al.* published in 2019 a cross-sectional study that analyzed information from the Brazilian National Health Survey conducted in 2013 on a sample of 60 202 adults aged over 18 years, stratified by sex and 6 age groups, using a hierarchical binary logistic regression model. The self-reported diagnosis of heart disease in Brazil was 4.2% (95% CI: 4.0 – 4.3 ) and was associated with female sex (OR = 1.1; 95% CI: 1.1 – 1.1), individuals 65 years and older, hypertension (OR = 2.4; 95% CI: 2.2 – 2.7), increased cholesterol (OR = 1.6; 95% CI: 1.5 – 1.8), overweight (OR = 1.5; 95% CI: 1.4 – 1.8) or obesity (OR = 2.0; 95% CI: 1.7 – 2.2), sedentary behavior (OR = 1.5; 95% CI: 1.02 – 2.1), and tobacco use (OR = 1.2; 95% CI: 1.03 – 1.3).^36^
- In the ELSA-Brasil, a cohort study that included 15 105 civil servants from 6 universities or research institutes (54% women, 35-74 years, with baseline assessment between 2008 and 2010), the self-reported prevalence was as follows: coronary heart disease, 4.7% (men=5.7%, women=4.0%); HF, 1.5% (men=1.9%, women=1.5%); stroke, 1.3% for both sexes; rheumatic fever, 2.9% (men=2.2%, women=3.4%); and Chagas disease, 0.4%, for both sexes.^37^
- The prevalence of CVDs increases significantly with age. In a longitudinal study in the elderly aged over 60 years, from the state of São Paulo, in 2000, 2006 and 2010, the prevalence of CVDs was defined as the individual’s positive response to the question: “Has a doctor or nurse ever told you that you have had a heart attack, coronary heart disease, angina, congestive disease, or other heart problems?” The CVD prevalence was 17.9%, 22.2% and 22.9% for 2000, 2006 and 2010, respectively. The presence of CVD was associated with older age, smoking history, presence of diabetes, and hypertension.^38^
- According to the GBD Study 2017, the age-standardized prevalence of CVD in Brazil, in 1990, was 6290 (95% UI, 6048-6549) per 100 000 inhabitants, and, in 2017, 6025 (95% UI, 5786-6275) per 100 000 inhabitants, affecting 6% of the population aged ≥ 20 years, with a slight reduction of 4.2% (95% UI, -3.2 to -5.1) from 1990 to 2017. Males had a higher age-standardized prevalence than females from 1990 to 2017 (Charts 1-5 and 1-6), although the percent of change was greater for males -5.5 (-4.2; -6.7) than for females -2.4 (-1.3; -3.4) in that period (Chart 1-6). Considering the total number in 2017, 13 702 303 individuals (95% UI, 13 110 682-14 281 540) had prevalent CVD in Brazil, 6 784 523 men (95% UI, 6 517 523-7 167 162) and 6 917 779 women (6 616 359-7 220 572) (Table 1-1).^39^
- The GBD Study 2017 reveals that the age-standardized prevalence of CVD decreased unevenly in the FUs, with a major reduction in the Southeastern and Southern regions, particularly in the states of Espírito Santo, Rio de Janeiro, Santa Catarina, and Rio Grande do Sul (Table 1-1), which are among the most developed states of the country.

## Incidence

- According to the GBD Study 2017, the age-standardized incidence rate of CVD in Brazil in 2017 was 687.5 (95% UI, 663.4-712.4) cases per 100 000 inhabitants, lower than in 1990, when there were 755.6 (95% UI, 731.6-783) cases per 100 000 inhabitants (Table 1-2). Of note, in the whole time series, the GBD incidence rates for total CVDs may be underestimated due to the entrance and exclusion criteria of the models for each specific disease included. The incidence of total CVDs is the aggregate of the incidences of all diseases contained within.^39^
- The FU with the highest incidence rate in 2017 was the state of Rio de Janeiro, with 709 (95% UI, 683.9-734.5)cases per 100 000 inhabitants, and the FU with the lowest incidence rate in 2017 was the state of Rio Grande do Sul, with 646.6 (95% UI, 621.9-674.4) cases per 100 000 inhabitants (Table 1-2).

## Mortality

- In Brazil, Mansur and Favarato have shown that the CVD age-standardized mortality rate has declined significantly in recent decades. A 2016 study analyzed CVD mortality rates for the age of 30 years and older, by sex, per 100 000 inhabitants. The annual variations in cardiovascular mortality for the periods 1980-2006 and 2007-2012 were, respectively: for both sexes: -1.5% and -0.8%; for men: -1.4% and -0.6%; for women: -1.7% and -1.0%.^40^
- Baptista *et al.* have investigated how age composition and age-specific mortality rates are related to the observed difference in deaths from CVD in the adult population, by sex, in Brazilian microregions from 1996 to 2015. They have suggested, after correcting for underreporting of death counts, that there is a decline in the rates of death from CVD over the period studied. However, the main driver of the change in mortality rates was heterogeneous across Brazilian microregions. In general, in the most socioeconomically developed areas, the age structure was more importantly related to the mortality rates, with older populations dying from CVD. Interestingly, there were differences in the main drivers of CVD mortality even within the Brazilian FUs.^41^
- Data from the GBD Study 2017 reveal that, although mortality rates from CVD in Brazil have significantly decreased over the past few years, the total number of deaths due to CVD has increased, probably as a result of population growth and aging. There were 266 958 (95% UI, 264 385-269 671) and 388 268 (95% UI, 383 815-392 698) deaths from CVD in the country in 1990 and 2017, respectively. The age-standardized mortality rate per 100 000 inhabitants was 341.8 (95% UI, 338.7-345.2) in 1990, and 178.0 (95% UI, 175.9-180) in 2017, decreasing -47.9 (95% UI, -48.5 to -47.2) in the period (Chart 1-7). Although age-standardized mortality rates were higher for men throughout the whole period, the percent decrease was similar for both sexes, and the proportional mortality due to CVD was higher for women, exceeding 30% throughout the entire period, while that for men remained always slightly over 25% (Chart 1-8).
- Table 1-3 shows the number of deaths, the age-standardized mortality rate per 100 000 inhabitants, and percent change due to CVD, by FU, in Brazil, in 1990-2017. The FUs with the highest percentages of reduction observed between 1990 and 2017 were the states of Espírito Santo, Paraná, Minas Gerais, Santa Catarina, Rio de Janeiro, São Paulo, and Rio Grande do Sul, in that order.
- Chart 1-9 shows the geographical distribution of mortality rates per 100 000 inhabitants, standardized by age in the Brazilian FUs, for both sexes, in 2000 and 2017, according to the SIM, and using the IBGE population, the redistribution of ill-defined causes and the correction for underreporting according to the GBD 2017 coefficients. There was a decrease in the standardized mortality rates for both sexes, except for the males from the states of Maranhão and Roraima. Malta *et al.* have compared a historical series of CVD mortality rates in Brazil using SIM database with and without correction (crude data) and the GBD 2017 estimates between 2000 and 2017. The authors have pointed out that the increase in the age-standardized crude CVD mortality rates from SIM observed in 2017 as compared to 2010 in most Northern and Northeastern FUs was in fact due to improvements in death registration and in the definition of underlying causes of death in recent years. When SIM corrected data or the GBD 2017 estimates were used, the trends from 2010 to 2017 were similar across all Brazilian FUs.^42^
- Brant *et al.* , analyzing GBD 2015 data, have observed a decrease in age-standardized CVD mortality rate from 429.5 (1990) to 256.0 (2015) per 100 000 inhabitants (40.4%), with marked differences across the FUs. That decrease was more pronounced in the FUs of the Southeastern and Southern regions and the Distrito Federal, regions that concentrate the largest populations and income, and more modest in most Northern and Northeastern states. Importantly, Brant *et al.* have also emphasized that the annual reduction in CVD mortality rates in Brazil was lower in the final years of the series analyzed (1990-2015). When considering the 1990-2017 period, the GBD 2017 estimates confirm the same trend for males and females. Charts 1-10 and 1-11 show the decline in age-standardized CVD mortality rate from 1990 to 2017, revealing a reduction in the decline in the last 5 years of the series, when the rates achieved a plateau or even increased in some regions.^35^
- Regarding the trend by age group, the largest reductions in the CVD mortality rates per 100 000, between 1990 and 2017, were observed in the ‘under-5’ age group [-65.4 (-71.2; -60)], followed by the ‘5-14 years’ age group [-48.3 (-52.5; -42.1)], the ‘50-69 years’ age group [-46.5 (-47.4; -45.6)], and lastly the ‘>70 years’ age group, revealing a shift in CVD mortality to older individuals.
- The FHP coverage was associated with a reduction in hospitalizations and mortality from CVD that were included in the national ambulatory care-sensitive list in Brazil, and its effect increased according to the duration of the FHP implementation in the municipality. Rasella *et al.* have observed reductions in cerebrovascular disease and heart disease mortalities of 0.82 (95% CI: 0.79 - 0.86) and 0.79 (95% CI: 0.75 - 0.80), respectively, reaching 0.69 (95% CI: 0.66 - 0.73) and 0.64 (95% CI: 0.59 - 0.68), respectively, when the FHP coverage was consolidated during all 8 years studied.^43^
- According to the SIM database, in 2017, CVD corresponded to 27.3% of total deaths, with the highest proportion in the Southeastern region and the lowest in the Northern region. Ischemic heart disease accounted for 32.1% of total deaths from CVD in Brazil, and stroke was responsible for 28.2% of those deaths. The highest proportion of IHD mortality occurred in the states of Mato Grosso do Sul, Pernambuco, and Espírito Santo, while the highest proportion of stroke deaths occurred in the states of Amazonas and Pará, and the Distrito Federal (Table 1-4).
- The proportion of deaths due to CVD decreased for men (from 30.1% to 27.6%) and women (31.1% to 29.9%) from 2000-2002 to 2015-2017. Moreover, Lotufo has noted a constant excess of premature male deaths due to CVD during that period, with a male-to-female ratio of 2:1.^44^
- The SDI is an estimate of the socioeconomic level and can be used to assess the association of socioeconomic level with CVD burden. Chart 1-12 shows the correlation between the percent change in age-standardized mortality rate 2017/1990 and the 2017 SDI. It reveals a correlation between the greater reduction in percent change in age-standardized mortality rates from CVD, between 1990 and 2017, and the higher 2017 SDI, suggesting that the decrease in mortality from CVD followed the improvement in the local socioeconomic conditions, as observed in other studies.^32 , 45 - 47^
- Lotufo *et al.* have compared 3 different household income levels (high, middle, and low) with mortality rates due to CVD, in the city of São Paulo, from 1996 to 2010. The annual percent changes and 95% CI for men living in the high, middle- and low-income areas were -4.1 (95% CI: -4.5 to -3.8), -3.0 (95% CI: -3.5 to -2.6), and -2.5 (95% CI: -2.8 to -2.1), respectively. The trend rates for women living in the high-income areas were -4.4 (95% CI: -4.8 to -3.9), in 1996-2005, and -2.6 (95% CI: -3.8 to -1.4), in 2005-2010. The reduction in deaths due to CVD was most significant for men and women living in the wealthiest neighborhoods, with a declining gradient for risk of death that was greater for people living in the wealthiest areas as compared to people living in more deprived neighborhoods.^48^
- An inverse association between the HDIm and the supplementary health coverage with mortality due to CVD was observed, suggesting a relationship between socioeconomic factors and CVD.^45^The HDIm increased between 2000 and 2010 in all FUs, being 0.7 or higher in half of the FUs. Supplementary health coverage increased in the country during the study period and was inversely associated with mortality due to CVD between 2004 and 2013.^45^
- Soares *et al.* have observed a decrease in CVD mortality in the states of Rio de Janeiro, São Paulo, and Rio Grande do Sul that preceded improvement in the socioeconomic index. The evolution of GDP per capita, the decline in child mortality, the higher educational level (represented by the years of schooling of individuals over the age of 25 years), and the HDIm showed a high correlation with the reduction in the CVD mortality rate. A reduction in the mortality rates due to CVD, stroke, and IHD in the state of Rio de Janeiro in recent decades was preceded by an increase in the HDI, with significant numbers, because a 0.1 increment in the HDI correlated with the following reductions in the number of deaths per 100 000 inhabitants: 53.5 for DCV; 30.2 for stroke; and 10.0 for IHD.^46 , 47^
- Baptista and Queiroz have investigated the relationship between crude CVD mortality rate and economic development over time and space, measured by GDP per capita, in Brazilian microregions, from 2001 to 2015. They have used the databases SIM-DATASUS and SIDRA (IBGE). The authors have observed a rapid decline in crude CVD mortality rates in the Southern and Southeastern microregions and a slower decline in the West-Central region. On the other hand, the Northern and Northeastern regions had an increase in crude CVD mortality rates over time, reflecting the later aging of the population in these Brazilian regions, and maybe lower access to healthcare and other socioeconomic factors.^49^
- Silveira *et al.* , studying the effect of ambient temperature on cardiovascular mortality in 27 Brazilian cities, have observed a higher number of cardiovascular deaths associated with low and high temperatures in most of the Brazilian cities and the West-Central, Northern, Southern, and Southeastern regions. The overall RR for Brazil was 1.26 (95% CI: 1.17–1.35) for the 1^st^percentile of temperature and 1.07 (95% CI: 1.01–1.13) for the 99^th^percentile of temperature versus the 79^th^percentile (27.7 °C), in which RR was the lowest.^50^

## Burden of Disease

- Age-standardized DALY rates in Brazil were 6907 (95% UI, 6783-7039) per 100 000 inhabitants in 1990 and decreased to 3735 (95% UI, 3621-3849) per 100 000 inhabitants in 2017. The Southeastern region had the highest DALY rates, and the Northern and Northeastern regions, the lowest DALY rates (Chart 1-13). The trend in DALY rates between 1990 and 2017 in Brazil is similar to that reported for the age-standardized mortality rate: there was a heterogeneous reduction in all FUs, more expressive in those with better SDI (Chart 1-14 and Table 1-5).

## Healthcare Utilization and Cost

### (Refer to Tables 1-6 to 1-9)

- In Brazil, CVDs have been responsible for the most substantial direct expenses with hospitalizations and indirect costs from reduced productivity due to time off work.^51 -^^53^The CVDs and their complications resulted in a US$ 4.18 billion expenditure in the Brazilian economy between 2006 and 2015.^54 , 55^
- From the SIH/SUS database, we evaluated the number of hospitalizations from 2008 to 2018 and their costs based on the procedure codes related to the topics of the present document: stroke; chronic and acute coronary artery disease; cardiomyopathies (including Chagas disease) and HF; valvular diseases; and atrial fibrillation (Table 1-6). Procedures related to congenital heart disease, peripheral artery disease, endocarditis, and arrhythmias other than atrial fibrillation were not included.
- Regarding clinical hospitalizations, HF led the admissions for CVDs with 2 862 739 hospitalizations (131 per 100 000 inhabitants), followed by cerebrovascular diseases with 2 042 195 hospitalizations (93 per 100 000 inhabitants), AMI and ACS with 1 461 388 hospitalizations (76 per 100 000 inhabitants), and atrial fibrillation with 321 866 hospitalizations (14.7 per 100 000 inhabitants) (Table 1-7).^54^The rates (per 100 000 inhabitants) of clinical admissions related to the evaluated procedure codes (324 to 293) showed a 10% decrease from 2008 to 2018, although the absolute numbers had only a slight reduction, from 615 433 in 2008 to 610 273 in 2018.
- Of the 1 149 602 cardiovascular surgical/interventional procedures performed under the selected procedure codes (Table 1-6), coronary angioplasty accounted for 66% (755 557), followed by CABG surgery (21%, 244 105), and heart valve surgery (8%, 88 280). The angioplasty/CABG surgery ratio in 2008 was 2.2 and increased to 4.3 in 2018. During the period, there was an increase in the number of procedures related to myocardial infarction and ACS (70%). The surgical hospitalization rates per 100 000 inhabitants related to the evaluated procedure codes from 2008 to 2018 (42 to 59) showed a 29% increase, while the absolute numbers grew from 80 010 in 2008 to 122 890 in 2018 (Table 1-7).
- The ELSI-Brasil, a study conducted in 2015–2016 with a nationally representative sample of the Brazilian population aged 50 years and over, has assessed the hospitalizations of 9389 participants (mean age, 63 years; 54% women) and has found that 10.2% of them had been hospitalized in the previous 12 months. The analysis of PAR for hospitalization revealed major contributions from stroke (PAR = 10.7%), CVD (PAR = 10.0%), and cancer (PAR = 8.9%).^56 , 57^
- About half of the health costs in Brazil is funded by the government.^54^The CVDs account for the highest spending on hospitalizations in the SUS and create the most significant number of disability pensions and higher morbidity burden for patients.^58 - 61^In 2015, the estimated direct public sector spending on hospitalizations and consultations for CVD in Brazil was over R$ 5 billion. It is estimated that the cost of temporary or permanent leave for CVD exceeded R$ 380 million.^62^
- According to the AHA,^62^it is projected that 61% of direct health expenditures for CVD in the United States, between 2012 and 2030, will be attributed to hospital costs. In Brazil, in 2012, the SUS spent US$ 608.9 million in highly complex therapeutic procedures performed during hospitalizations for CVD, 34% of which were associated with coronary angioplasty and 25% were related to CABG surgery.^58 , 63^Tables 1-8 and 1-9 show the amount of Reais paid for clinical and surgical procedures, respectively, in Brazil from 2008 to 2018 by the public health system. Stroke and HF were responsible for the highest cumulative values reimbursed for clinical procedures, which totalized R$ 8.4 billion. The total amount paid for surgical procedures was R$ 9.5 billion, and coronary angioplasty and CABG surgery represented the highest values paid.
- From 2008 to 2018, the inflation rate in Brazil was 88% (ranging from 76% to 96%), and unadjusted values spent by the SUS increased in a similar proportion for most clinical conditions, doubling their expenses in the period, except for HF hospitalizations, in which total values reimbursed increased only 28%, and chronic IHD, in whose costs there was a 27% reduction. For surgical admissions, all related groups increased the values expended – most of them nearly doubled the values, except for mitral valvuloplasty, which showed a 53% decrease in the amount paid by the SUS in that decade (Tables 1-8 and 1-9). However, when values spent by the SUS are calculated in international dollars, the costs for most of the clinical hospitalization groups changed less than 10%, except for that for HF, whose expenses decreased by 35%, and for myocardial infarction and cardiomyopathies, whose expenses increased by 13% and 23%, respectively. Regarding surgical/interventional procedures, there was a reduction in costs in all groups reported, except for coronary angioplasties, whose costs increased by 20%, and cardiomyopathies, whose costs increased by 30% (Tables 1-8 and 1-9).

## Future Research

- The SIM, implemented in 1975, is an essential tool for monitoring mortality statistics in Brazil, because the registration of all deaths is mandatory in the FUs, with 98% coverage of the national territory in 2017, that coverage being lower in the Northern region than in the Southern region. The Northeastern region has the poorest coverage, still under 95%.^56^Although SIM has improved through specific Ministry of Health projects,^64 , 65^problems still persist, such as ill-defined causes (around 6%), garbage codes and underreporting of deaths, which generate biases that may disrupt the metrics presented. As such, further research is needed to promote methodological adjustments for coverage, redistribution of ill-defined causes, especially in the older years of the historical series. On the other hand, the estimates from the GBD Study need additional research to implement models with better distribution of garbage codes adapted to local realities.
- It is worth mentioning that there is a lack of primary incidence data (cohorts) in Brazil, requiring research that allows us to understand how to face CVD in states and populations with low socioeconomic indices.
- Because of the reduction in the decline trend of age-standardized CVD mortality in the last 5 years, novel strategies to tackle CVD mortality must be studied. Understanding of the drivers of this reduction is essential to implement effective policies, particularly facing population aging, which will increase the number of individuals with CVD in the country.
- Most cost data were gathered from reimbursement tables of the Public Health System from 2008 to 2018 and do not capture the true cost related to those conditions. Trustworthy and comprehensive cost information of the delivery of care for cardiovascular conditions are extremely important to better understand the financial impact of those diseases and to further reevaluate prevention and management strategies. In addition, cost data from the supplementary health system, as well as ambulatory care and indirect costs are critical for a comprehensive economical appraisal of CVD in Brazil.

## 2. STROKE (CEREBROVASCULAR DISEASES)

### ICD-9 430 to 438; ICD-10 I60 to I69.

**See Tables 2-1 through 2-10 and Charts 2-1 through 2-4**

**Table:** 

Abbreviations used in Chapter 2	
ACEI/ARB	Angiotensin-Converting Enzyme Inhibitor/Angiotensin Receptor Blocker
APC	Annual Percent Change
CHD	Coronary Heart Disease
CI	Confidence Interval
CVD	Cardiovascular Diseases
DALY	Disability-Adjusted Life Year
FHP	Family Health Program
FU	Federative Unit
GBD	Global Burden of Disease
GWTG-Stroke	Get With The Guidelines-Stroke
HR	Hazard Ratio
HS	Hemorrhagic Stroke
IBGE	Brazilian Institute of Geography and Statistics
ICD	International Statistical Classification of Diseases and Related Health Problems
ICD-9	International Statistical Classification of Diseases and Related Health Problems, 9^th^Revision
ICD-10	International Statistical Classification of Diseases and Related Health Problems, 10^th^Revision
ICF	International Classification of Functioning, Disability and Health
JCI	Joint Commission International
LDL	Low-Density Lipoprotein
LV	Left Ventricular
NCEP	National Cholesterol Education Program Adult Treatment Panel
NIHSS	National Institutes of Health Stroke Scale
OR	Odds Ratio
PNS	National Health Survey (in Portuguese, Pesquisa Nacional de Saúde)
PSC	Primary Stroke Center
QALY	Quality-Adjusted Life Year
rtPA	Recombinant Tissue Plasminogen Activator
SAH	Subarachnoid Hemorrhage
SD	Standard Deviation
SIH	Brazilian Hospital Information System (in Portuguese, *Sistema de Informações Hospitalares* )
SIM	Brazilian Mortality Information System (in Portuguese, *Sistema de Informações sobre Mortalidade* )
SSQOL	Stroke Specific Quality of Life Scale
SUS	Brazilian Unified Health System (in Portuguese, Sistema Único de Saúde)
TIA	Transient Ischemic Attack
UI	Uncertainty Interval
WHO	World Health Organization

## Prevalence

- Stroke prevalence estimates may differ slightly between studies because each study selects and recruits a sample of participants to represent the target study population (e.g., state, region, or country).
- In a community-based study in Brazil, using a questionnaire applied to 4496 individuals aged over 35 years, living in a deprived neighborhood in the city of São Paulo in 2011, Abe *et al.* have found 243 individuals initially screening positive for stroke. The age-standardized prevalence rate for men was 4.6% (95% CI, 3.5-5.7) and, for women, 6.5% (95% CI, 5.5-7.5).^67^
- In a population-based cross-sectional study that included 3391 individuals aged 20 years and over, performed in the city of Porto Alegre, Southern Brazil, from July through December 2009, with systematic sampling, Copstein *et al.* have found 285 individuals who reported diagnosis or symptoms consistent with prior stroke (8.4% of the sample).^68^
- Using a screening tool, the Stroke Symptom Questionnaire, Fernandes *et al.* have studied stroke prevalence in the town of Coari, in the Brazilian Amazon Basin, and compared stroke prevalence in riverside inhabitants to that in the urban population of the same municipality. Out of 4897 respondents in the urban area and 1028 in the rural area, the authors have found a 6.3% crude prevalence of stroke in the rural area and 3.7% in the urban area, with differences maintained after sex and age adjustment.^69^
- Pereira *et al.* have conducted a study to estimate the prevalence of stroke among the elderly in the city of Vassouras, state of Rio de Janeiro, Brazil, 2007, using data from the Information System on Primary Care, the population census conducted by the IBGE, and the standardized patient form from the Ministry of Health FHP. The quality of stroke diagnoses in the FHP was analyzed. Out of 4154 elderly screened, the study detected 122 with a history of stroke diagnosis (prevalence, 2.9%; men, 3.2%; women, 2.7%) and a progressive increase with age. The prevalence rate was the same in the rural and urban areas of the municipality (2.9%).^70^
- Using the WHO Stepwise Approach to Stroke Surveillance, Goulart *et al.* have conducted a study to verify stroke mortality and morbidity rates in an area of São Paulo, Brazil. The questionnaire determining stroke prevalence was activated door-to-door in an FHP neighborhood (Step 3). Out of 3577 subjects over the age of 35 years evaluated at home, 244 cases (6.8%) of stroke survivors were identified via the questionnaire validated by a board-certified neurologist.^71^
- Benseñor et al. have analyzed a community-based epidemiological survey (PNS – 2013) with a Brazilian representative sample to assess the absolute number of stroke and post-stroke disabilities with respective prevalence rates. The authors have estimated 2 231 000 strokes and 568 000 post-stroke severe disabilities. The point prevalence for stroke was 1.6% and 1.4% for men and women, respectively.^72^
- According to the GBD Study 2017, the age-standardized prevalence of stroke decreased 44.2% (95% UI, -41.7 to -46.9) in Brazil from 1810.9 (95% UI, 1530.9-2131.5) in 1990 to 1010.6 (95% UI, 843.2-1197.8) in 2017.^7^The age-standardized prevalence decreased similarly among males and females, with a higher prevalence of stroke across the whole period in males (Table 2-1). Table 2-1 also depicts the age-standardized prevalence rates by FU in Brazil.
- Data from the ELSA-Brasil cohort study, a six-center cohort study of civil servants in Brazil that included 15 105 adults (45.8% men; age range, 35 to 74 years), have shown a stroke prevalence of 1.3% for males and females.^73^

## Incidence

### Stroke Subtypes

- In a hospital-based study held in the region of Fortaleza, in the Northeast of Brazil, ischemic stroke was the most frequent subtype (72.9%), followed by intracerebral hemorrhage (15.2%), SAH (6.0%), TIA (3.0%), and undetermined stroke (2.9%).74 This distribution is similar to that of the community-based study conducted from 2005 to 2006 in the city of Joinville, in the South of Brazil, in which, of the 759 first-ever strokes, 610 (80.3%) were ischemic strokes, 94 (12.3%) were HS, and 55 (7.2%) were SAH. In Joinville, the annual incidence per 100 000 person-years was 61.8 (95% CI, 57.0-66.9) for ischemic stroke, 9.5 (5% CI, 7.7-11.6) for HS, and 5.6 (95% CI, 4.2-7.3) for SAH. The incidence of stroke, adjusted to the world population, per 1000 inhabitants older than 55 years, was 5.8 (95% CI, 5.4 - 6.2). The incidence of infarction was 4.7 (95% CI, 4.3 - 5.1), of intracerebral hemorrhage, 0.6 (95% CI, 0.5 - 0.8), and of SAH, 0.3 (95% CI, 0.2 - 0.4).^75^
- In a population-based study conducted in Matão (Southeast Brazil) from 2003 to 2004, the crude annual incidence rate per 100 000 per year was 108 (95% CI, 85.7 - 134.1) and the rate adjusted to sex and age using the Segi Standard Population was 137 (95% CI, 112.0 - 166.4) per 100 000 inhabitants per year. Ischemic stroke occurred in 69 (85.2%) subjects, intracerebral hemorrhage in 11 (13.6%), and SAH in 1 (1.2%).^76^
- Data from the Joinville community-based study have shown that, when comparing different time periods (1995, 2005-2006, 2010-2011, and 2012-2013), the stroke incidence has decreased. Over the last 18 years, the overall stroke (e.g., all major stroke types) incidence in Joinville decreased by 37% (95% CI, 32 - 42).^75^The incidence of first-ever stroke adjusted to the Brazilian population was 86.6 per 100 000 (95% CI, 80.5 - 93.0) in 2005-2006 and 113.46 per 100 000 (95% CI, 101.5 - 126.8) in 1995.^77^The overall incidence, age-adjusted to world population per 100 000 person-years was 143.7 (95% CI, 128.4 - 160.3) in 1995, fell to 105.4 (95% CI, 98.0 - 113.2) in 2005–2006, and then to 90.9 (95% CI, 85.1 - 96.9) in 2012-2013. The age-standardized incidence of first-ever stroke stratified by gender and age also decreased significantly over time. The reduction was 11% greater in men (42%; 95% CI, 35 - 49) than in women (31%; 95% CI, 23 - 39), and 16% greater in young people (≤ 44 years: 54%; 95% CI, 41 - 66) than in older individuals (> 44 years: 38%; 95% CI, 33 - 43). From 1995 to 2013, the proportion of ischemic stroke increased by 12%, whereas that of HS decreased by 16%. Meanwhile, the proportion of SAH remained relatively stable, ranging from 7.5% in 1995 to 6% in 2012-2013. The weight of the decrease in age-standardized stroke incidence was proportionally higher for HS than for ischemic stroke, whereas that of SAH remained stable. In the last 8 years, the incidences of ischemic stroke and HS had significant absolute reductions of 15% (95% CI, 1.00 - 28.00) and 60% (95% CI, 13.00 - 86.00), respectively. Meanwhile, the incidence of SAH showed a 29% nonsignificant absolute decrease (95% CI, 15.00 - 92.00).^78^
- A study including 213 consecutive patients with Chagas disease cardiomyopathy in Brazil has explored the long-term cumulative risk of stroke and TIA and its relation to LV dysfunction in those patients from June 1999 to January 2007. After a mean 36-month follow-up, the overall incidence of ischemic stroke was 2.67 events per 100 patient/years. Independent risk factors for stroke and TIA included LV ejection fraction (HR 0.95; 95% CI, 0.91 - 0.99, p=0.009) and left atrial volume corrected for body surface area (HR 1.04; 95% CI, 1.01 - 1.07, p=0.007), which persisted after adjustment for anticoagulation use.^79^

## Mortality

- Data from the Joinville Stroke Registry (n=759 first-ever strokes) have shown that the mortality rate adjusted to the Brazilian population in the 2005-2006 period was 20.5 per 100 000 (95% CI, 17.5 - 23.8), and, when adjusted to the world population, 23.9 per 100 000 (95% CI, 20.4 - 27.8), revealing a decreasing trend from 1995 on. There was also a decrease in mortality adjusted to age, although it was much more pronounced in males (48%) than in females (3%). The case-fatality rate was 19.1% (145/759) in the 2005-2006 period, which is also lower than that found in 1995 [26.6% (84/320)]. Therefore, over a span of approximately 10 years, mortality fell by 37%. The 30-day case-fatality rate decreased by 28.2% during the period (from 26.6% to 7.5%).^77 ,^^80^
- In a population-based study conducted in Matão, in the Southeastern region (n=141), the overall 30-day case-fatality rate was 18.5% (95% CI, 10.7 - 28.7%). Regarding stroke subtypes, the 30-day case-fatality was 13% (95% CI, 6.1 - 23.3%) for ischemic stroke and 45.4% (95% CI, 16.7 - 76.2%; p=0.02) for HS. The overall 1-year case-fatality rate was 30.9% (95% CI, 21.1 - 42.1%). Regarding stroke subtypes, the 1-year case-fatality rate was 24.6% (95% CI, 23.7 - 47.2%) for ischemic stroke and 63.6% (95% CI, 30.7 - 89.0%; p=0.01) for HS.^76^
- Data from the GBD Study 2017 have shown that the age-standardized mortality rates per 100 000 for stroke in 1990 was 122.9 (95% UI, 120.6 - 125) and 56.6 (95% UI, 55.2 - 57.8) in 2017, representing a percent change of -54 (95% UI, -55.1 to -53) (Chart 2-1 and Table 2-4). The highest percent change occurred in the state of Espírito Santo, -68.3 (95% UI, -69.9 to -66.5), and the lowest, in the state of Maranhão, -31.7 (95% UI, -36.6 to -26.6) (Table 2-2). For adults, the highest percent change was observed among people aged between 50 and 69 years, -56 (95% UI, -57.5 to -54.5) (Table 2-4).
- Regarding stroke subtypes, data from the GBD Study 2017 have revealed age-standardized ischemic stroke mortality rates per 100 000 of 54.8 (95% UI, 53.6-55.9) in 1990 and of 22.6 (95% UI, 21.9-23.2) in 2017, representing a percent change of -58.7 (95% UI, -60 to -57.4) (Chart 2-2 and Table 2-2). For adults, the highest percent change was observed among people aged between 50 and 69 years, -63.7 (95% UI, -65.8 to -61.6) (Table 2-4).
- For intracerebral hemorrhage, the age-standardized mortality rates (GBD Study 2017) per 100 000 were 58.6 (95% UI, 57.3 - 59.9) and 27.9 (95% UI, 27.1 - 28.7) in 1990 and 2017, respectively, representing a percent change of -52.4 (95% UI, -53.8 to -51.1) (Chart 2-3 and Table 2-4). For adults, the highest percent change was observed among people aged between 15 and 49 years, -57.6 (95% UI, -60.9 to -55.4) (Table 2-4).
- For SAH, the age-standardized mortality rates (GBD Study 2017) per 100 000 were 9.6 (95% UI, 8.8 - 9.9) and 6.1 (95% UI, 5.0 - 6.7) in 1990 and 2017, respectively, representing a percent change of -36.5 (95% UI, -39.8 to -32.2) (Chart 2-4 and Table 2-4). For adults, the highest percent change was observed among people aged between 15 and 49 years, -39.4 (95% UI, -43.6 to -29.4) (Table 2-4).
- Analyzing the estimates from the GBD 2015 in the 27 Brazilian FUs between 1990 and 2015, Lotufo et al. have shown that, despite the increase in the absolute number of deaths due to cerebrovascular disease, the proportion of deaths under the age of 70 years was halved between 1990 and 2015. From 1990 to 2015, the risk of death attributable to stroke decreased for both men (-2.41% per year) and women (-2.51% per year). Nevertheless, the annual reduction in mortality rates adjusted to age, for both sexes, slowed between 2005 and 2015 when compared to the previous period of 1990-2005. States in the lower social development index tertile had less significant reductions (-1.23 and -1.84% per year) as compared to those in the middle tertile (-1.94 and -2.22%) and in the upper tertile (-2.85 and -2.82%), for men and women, respectively. In addition, the years lived with disability decreased among states, but less expressively.^81^
- André et al., using data from the SIM corrected for ill-defined deaths, have shown that the age-standardized stroke mortality rates consistently decreased between 1980-1982 and 2000-2002, from 68.2 to 40.9 per 100 000 inhabitants. During the same period, total cardiovascular mortality rates also declined markedly, from 208.2 to 126.1 per 100 000 inhabitants. The decline in the age-standardized stroke mortality rate was evident in both decades, with the highest decrease observed between 1990-1992 and 2000-2002. Using the 1980 rate as a reference, there was a 30% (95% CI, 30% - 31%) risk reduction in 1990 and a 55% (95% CI, 55% - 56%) risk reduction in 2000 (P<0.001 for both measures). The proportional reduction in stroke mortality was evident for both men and women, although it was more marked among men. In addition, the decrease was observed in all age strata. An interaction between sex and age was detected, with a more marked decline in age-standardized mortality rates in the young male population (up to 45 years) and a steeper decline for women of all other age strata (P<0.001 for all findings). A reduction in age-standardized stroke mortality rates occurred in all geopolitical regions. An interaction between the studied region and the reduction magnitude was detected. The wealthiest regions (South and Southeast) exhibited higher initial rates and more marked reductions during the study period. The findings were confirmed by the Poisson regression model, in which the least marked reduction in standardized stroke mortality rate was found in the Northeastern region, 41% (95% CI, 40% - 42%). The corresponding values for the other regions were as follows: North, 52% (95% CI, 51% - 52%); West-Central, 53% (95% CI, 53% - 54%); South, 57% (95% CI, 56% - 57%); and Southeast, 59% (95% CI, 58% - 59%). The total number of deaths related to stroke in Brazil has, however, steadily increased in the last 3 decades. The mean annual number of deaths attributable to stroke increased from 79 862, in 1980-1982, to 101 625, in 2000-2002. A similar trend was evident for total cardiovascular mortality: 239 876 deaths in 1980-1982 and 311 138 in 2000-2002. This increase mainly reflects the progressive aging of the Brazilian population.^82^
- In another assessment of stroke mortality trends in Brazil from 1979 to 2009, after excluding deaths due to sequelae from stroke for men, the annual percent changes (95% CI) were as follows: 1979-1984, 0.7 (–0.8 to 2.1); 1984-1994, –1.8 (–2.4 to –1.2); 1994-2007, –5.0 (–5.4 to –4.7); and 2007-2009, –0.8 (–7.0 to 5.8). For women, the annual percent changes were as follows: 1979-1994, –1.9 (–2.2 to –1.6); 1994-1997, –7.5 (–14.0 to –0.6); 1997-2007, –4.0 (–4.6 to –3.3); and 2007-2009, 1.6 (–5.5 to 9.2). For the 2006-2009 period, the average annual percent change (95%CI) for all strokes was –3.1 (–3.3 to –2.9) for men and –2.9 (–3.1 to –2.8) for women. For the same period, the average annual percent change of death rates for stroke subtypes were, for men and women, respectively: intracerebral hemorrhage, –4.0 (–4.9 to –3.1) and –2.9 (–3.4 to –2.3); and ischemic stroke, –3.2 (–3.3 to –3.0) and –1.4 (–2.0 to –0.9).^83^
- An assessment considering reallocation of deaths with non-registered sex or age, redistribution of garbage codes and underreporting correction has shown the following stroke mortality rates for 1996 and 2011, adjusted for age, pre- and post-correction, respectively: 1) for men: in 1996, 82.9 and 113.6; and, in 2011, 49.6 and 60.9; and 2) for women: in 1996, 58.2 and 84.4; and, in 2011, 34.7 and 42.3.^84^
- A study assessing regional differences in mortality transition and using data from the SIM from 1990 to 2012 has shown a variation of -48.05% in the mortality coefficient for stroke. Most regions showed a reduction in age-standardized mortality rates: –62% in the Southeast; –55.5% in the South; –26.91% in the West-Central; and –20.8% in the North. Only the Northeast had an increase (13.77%).^85^
- In the city of São Paulo, from 1996 to 2011, 77 848 stroke deaths were confirmed, 51.4% of them among individuals aged 35-74 years. In that period, age-adjusted mortality rates for cerebrovascular diseases decreased by 46.6% in men and by 47.8% in women. For men in high-income neighborhoods, the downward trend was constant; in the middle-income area, there was a sharp decline from 1996 to 2000, followed by a slower pace between 2000 and 2011. In the low-income areas, the APC was higher between 1996 and 2002, with a mild decline in 2002–2011. For women in high income areas, there was a sharp decline from 1996 to 2003, and a lower decrease in the last half of the period; in the low- and middle-income areas, the decline was constant during all periods. For the full period, both sexes and age group of 35–74 years, the decline in age-adjusted rates was more pronounced among those residing in the wealthiest area as compared to those living in the poorest area. This same pattern, but with a magnitude decline, was observed in people aged ≥75 years in all areas as compared to other age groups, for both sexes. Additionally, the temporal evolution of the ratios between the age-adjusted rates of people aged 35–74 years living in low- and high-income areas was as follows: for men, from 1996 to 1998, the rate ratio was 2.03, and, from 2009 to 2011, it was 2.34. For women, in 1996–1998, the rate ratio was 2.09, and, in 2009–2011, it was 2.58. The trend of the ratios between the age-adjusted rates of these areas showed an APC growth of 1.4 (0.5–2.4) for men and of 1.1 (0.1–2.0) for women.^86^
- In a hospital-based study conducted in the Northeastern and Southeastern regions (n=962), overall 10-day and 28-day case-fatality rates were 7.9 (95% CI, 6.2-9.7) and 12.5 (95% CI, 10.4-14.5), respectively. The death rates of HS were higher than those of ischemic stroke at both 10 days (12.3[95% CI, 7.2-14.4] vs. 7.0[95% CI, 5.3-8.8]) and 28 days (19.8[95% CI, 13.6-26.0] vs. 11.1[95% CI, 8.9-13.3]). Other than advanced age, the risk factors for ischemic stroke case-fatality at 28 days were diabetes (OR=1.69; 95% CI, 1.06–2.68) and previous heart disease (OR=1.86; 95% CI, 1.17–2.96) after adjustment for age.^87^

## Burden of Disease

- Data from the GBD Study 2017 have shown that the age-standardized DALY rates for stroke per 100 000 were, in 1990, 2511.9 (95% UI, 2457.3 - 2567.6) and, in 2017, 1145.3 (1107.8 - 1185.3), representing a percent change of -54.4 (95% UI, -55.5 to -53.2) (Table 2-6). For males, the percent change was -54 (95% UI, -55.4 to -52.5) and, for females, -54.2 (95% UI, -55.9 to -52.7) (Tables 2-9 and 2-10). The highest percent change occurred in the state of Espírito Santo, -64.7 (95% UI, -66.5 to -62.8), and the lowest, in the state of Amapá, -29.5 (-33.8 to -25.2) (Table 2-5).
- The age-standardized DALY rates for ischemic stroke per 100 000 were 871.4 (95% UI, 841.1 to 902.3) and 387.3 (95% UI, 363.9 to 411.5) in 1990 and 2017, respectively, representing a percent change of -55.6 (95% UI, 55.5 to -53.2) (Table 2-6). For adults, the highest percent change was observed among individuals aged 50-69 years, -58.7 (95% UI, -60.9 to -56.4) (Table 2-6), -58.3 (95% UI, -61.1 to -55.6) for men and -58.7 (95% UI, -61.7 to -55.6) for women (Tables 2-9 and 2-10).
- The age-standardized DALY rates per 100 000 for intracerebral hemorrhage was 1322.1 (95% UI, 1291.8 - 1358.2) in 1990 and 576.9 (95% UI, 560.7 - 594.9) in 2017, representing a percent change of -56.4 (95% UI, -57.8 to -55.1) (Table 2-6). For adults, the highest percent change was observed among people aged 15-49 years, -57.9 (95% UI, -61.3 to -55.8) (Table 2-6), -60.1 (95% UI, -64.4 to -57.1) for men and -57.1 (95% UI, -59.8 to -54.7) for women (Tables 2-9 and 2-10).
- The age-standardized DALY rates per 100 000 for SAH was 318.4 (95% UI, 287 - 332.2) in 1990 and 181.0 (95% UI, 173.1 - 191.0) in 2017, representing a percent change of -43.1 (95% UI, -46.3 to -37.2) (Table 2-6). For adults, the highest percent change was observed among people aged 15-49 years, -40 (95% UI, -43.9 to -30.9), -44.2 (95% UI, -49.5 to -22.9) for men and -36.8 (95% UI, -41.6 to -31.8) for women (Tables 2-9 and 2-10).

## Complications

- Benseñor et al. have analyzed a community-based epidemiological survey (PNS – 2013) with a Brazilian representative sample to assess the absolute number and prevalence rates of stroke and post-stroke disabilities. The authors have estimated 2 231 000 strokes and 568 000 post-stroke severe disabilities. The point prevalence for stroke was 1.6% and 1.4% in men and women, respectively. The prevalence of post-stroke disabilities was 29.5% for men and 21.5% for women. Stroke prevalence rates increased with aging, low education level, among people living in urban areas, and showed no difference according to self-reported race. The degree of post-stroke disability was not statistically different according to sex, race, education level or living area.^72^
- Carvalho-Pinto et al. have conducted a retrospective observational study that collected data from medical records and home visits of post-stroke patients followed in a primary healthcare unit in the city of Belo Horizonte, Brazil, between May 2013 and May 2014. Data included health status, care received following stroke, personal and environmental contextual factors, functioning and disability, organized according to the conceptual ICF framework. Most participants had good self-perception of manual ability (2.39 [SD, 2.29] logits) and limited walking ability (88%), were capable of improving natural gait speed, had a change in balance (51.43%) and functional mobility (54.16%) with risk of falling, and had a negative self-perception of quality of life (average score of 164.21 [SD, 35.16] points in the SSQOL-Brazil).^88^
- According to the GBD study 2016, the highest percentage of deaths due to stroke in general occurred in individuals aged 70 years or over (60.2%; 95% UI, 59.9-60.5%) and in men (52.9%; 95% UI, 52.6-53.2%). Ischemic stroke was the most common type, accounting for 61.8% (95% UI, 61.5-62.1%) of deaths due to stroke in 2016. Most of the epidemiological indicators of stroke in general or of a type of stroke (incidence, prevalence, mortality-to-incidence ratio, mortality, DALYs, years lost due to disability, and years of life lost) were higher in men and in those aged 70 years or over.^89^

## Healthcare Utilization and Cost

### (Refer to Tables 1-6 through 1-9 and Charts 1-15 through 1-16)

### Hospital Admissions

- Lopes et al. have conducted an ecological study designed with analytical approach and data collected from the SIH on stroke episodes in the 1998-2012 period. All data were stratified by sex and age, creating an indicator for stroke-related hospital admission. The authors have observed a reduction in stroke-related hospital admissions from 37.56/105 inhabitants in 1998-2001 to 10.33/105 inhabitants in 2002-2005, a 73.64% decline. The reduction occurred in both sexes and all age groups.^90^
- Using time-series analysis, Katz et al. have evaluated the relationship between stroke-related unemployment rate and hospital admission in Brazil over a recent 11-year span. Data on monthly hospital admissions due to stroke from March 2002 to December 2013 were extracted from the Brazilian Public Health System Database, revealing 1 581 675 admissions due to stroke in the period. The unemployment rate decreased from 12.9% in 2002 to 4.3% in 2013, while admissions due to stroke increased. However, the adjusted model showed no positive association between unemployment rate and admissions due to stroke (estimated coefficient = 2.40±4.34; p=0.58).^91^
- Using data from the SIH, the SIM, and the IBGE, Adami et al. have analyzed rates of stroke-related mortality and incidence of hospital admissions in Brazilians aged 15-49 years, according to region and age group, between 2008 and 2012. Stroke was defined according to the ICD-10 (I60-I64). Crude and standardized mortality (WHO reference) and incidence of hospital admissions per 100 000 inhabitants, stratified by region and age group, were estimated. The authors have found 131 344 hospital admissions due to stroke in Brazilians aged 15-49 years between 2008 and 2012. During the same time, the rate of hospital admissions stabilized: 24.67 (95% CI, 24.66 - 24.67) in 2008 and 25.11 (95% CI, 25.10 - 25.11) in 2012 (β = 0.09, p = 0.692, r2 = 0.05).^92^
- Dantas et al. have conducted a study to assess public hospitalizations for stroke in Brazil from 2009 to 2016. The authors have selected hospitalization registries according to the stroke diagnosis codes from the ICD-10. From 2009 to 2016, the number of admissions increased from 131 122 to 146 950, and the absolute number of in-hospital deaths increased from 28 731 to 31 937. Younger age and male sex were significantly associated with patient survival. The annual age-adjusted hospitalization and in-hospital mortality rates decreased by 11.8% and 12.6%, respectively, but the case-fatality rate increased for patients older than 70 years.^93^

#### Healthcare Utilization

- An analysis of the expansion trends of the FHP coverage and hospitalization for conditions sensitive to primary care in Rio de Janeiro, between 1998 and 2015, has shown a 7.6% decrease in hospitalizations for cerebrovascular diseases.^94^
- A study carried out in the city of Joinville has assessed the impact of a stroke unit, the first established in Brazil, on the acute phase of stroke management as compared to the conventional general ward treatment. The study has assessed 35 and 39 patients allocated in a stroke unit and at a general ward, respectively, in 2000, showing a 10-day mortality in the stroke unit and at the general ward of 8.5% and 12.8%, respectively (p=0.41). For the stroke unit and the general ward treatments, the mortality rates were as follows, respectively: on day 30, 14.2% and 28.2% (p=0.24); on the 3^rd^month, 17.4% and 28.7% (p=0.39); and, on the 6^th^month, 25.7% and 30.7% (p=0.41). The 30-day survival curve showed a non-significant trend towards lower lethality in the stroke unit. In order to save one death in 6 months in the stroke unit, the number needed to treat was 20; to get one more home independent patient, the number needed to treat was 15.^95^
- A study has described the characteristics and provision of care for 148 patients admitted with ischemic stroke to a large, public, academic hospital in São Paulo, at the neurology emergency department and at the neurology ward. The study has shown that ischemic stroke was diagnosed in 79.6% of the CVD patients admitted to the neurology emergency department, and that thrombolysis was provided to 2.7%. The extent of ischemic stroke investigation and management differed significantly between the neurology emergency department and the neurology ward.^96^
- A hospital-based study, evaluating 2407 consecutive patients (mean age, 67.7 ± 14.4 years; 51.8% females) admitted to 19 hospitals in the city of Fortaleza with the diagnosis of stroke or TIA, has shown that ischemic stroke was the most frequent subtype (72.9%), followed by intraparenchymal hemorrhage (15.2%), SAH (6.0%), TIA (3%), and undetermined stroke (2.9%). The median time from symptom onset to hospital admission was 12.9 (3.8-32.5) hours. Hypertension was the most common risk factor. Only 1.1% of the patients with ischemic stroke received thrombolysis. The median time from hospital admission to neuroimaging was 3.4 (1.2-26.5) hours.^74^
- A study has compared the quality indicators for stroke management from a private tertiary hospital, certified by the JCI as a PSC, with those of the American Heart Association/American Stroke Association “GWTG-Stroke” program: (1) tissue plasminogen activator use in patients who arrived <2 hours from symptom onset; (2) antithrombotic medication use within 48 hours of admission; (3) deep vein thrombosis prophylaxis within 48 hours of admission for non-ambulatory patients; (4) discharge use of antithrombotics; (5) discharge use of anticoagulation for atrial fibrillation; (6) measurement of LDL and treatment for LDL > 100 mg/dL in patients meeting the NCEP III guidelines; and (7) counseling for smoking cessation. The study assessed 343 consecutive patients with acute ischemic stroke (70.8%) or TIA (29.2%) from August 2008 to December 2010. Antithrombotic medication within 48 hours was used in 98.5% of the eligible patients, and deep vein thrombosis prophylaxis in 100%. A total of 123 patients arrived within 2 hours from symptom onset, 23 were eligible for intravenous thrombolysis, and 16 were treated (69.5%). All eligible patients were discharged using antithrombotic medication, and 86.9% of the eligible patients who had atrial fibrillation received anticoagulation. Only 56.1% of the eligible patients were treated according to the NCEP III guidelines. Counseling for smoking cessation was done in 63.6% of the eligible patients.^97^
- A study evaluating factors that influence temporal trends in quality indicators for ischemic stroke in a JCI certified PSC has assessed 551 patients discharged with ischemic stroke from a tertiary hospital from January 2009 to December 2013. Median age was 77.0 years (interquartile range, 64.0-84.0), and 58.4% were men. Ten predefined performance measures selected from the GWTG-Stroke program were assessed. The quality indicators that improved with time were the use of cholesterol-lowering therapy (P = 0.02) and stroke education (P = 0.04). The median composite outcome perfect care did not consistently improve throughout the period (P = 0.13). After a multivariable adjustment, only thrombolytic treatment (OR 2.06, P<0.01), dyslipidemia (OR 2.03, P<0.01), and discharge in a JCI visit year (OR 1.8, P<0.01) remained as predictors of a perfect care index of 85% or higher. The quality indicators with worse performance (anticoagulation for atrial fibrillation and cholesterol reduction) were similar in the tertiary and secondary community hospitals. The overall perfect care measure did not improve and was influenced by being discharged in a JCI visit year, having dyslipidemia, and having undergone thrombolytic treatment.^98^
- A study has assessed the Brazilian Ministry of Health key performance indicators for stroke in the stroke units of two centers, including the percentage of patients admitted to the stroke unit, venous thromboembolism prophylaxis in the first 48 hours after admission, pneumonia and hospital mortality due to stroke, and hospital discharge on antithrombotic therapy in patients without cardioembolic mechanism. The analysis has shown that both centers admitted over 80% of the patients in their stroke units. The incidence of venous thromboembolism prophylaxis was > 85% and that of in-hospital pneumonia was <13%. The rate of in-hospital mortality from stroke was <15%, and that of hospital discharge on antithrombotic therapy was > 70%.^99^
- A before and after study has assessed the effect on mortality rates of the implementation of a dedicated cardiovascular and stroke unit in an emergency department of a tertiary public hospital in the city of Porto Alegre. The period prior to that unit implementation (2002 through 2005) included 4164 patients, and the period after that (2007 to 2010) included 6280 patients. Overall, the case-fatality rate for acute vascular conditions decreased from 9% to 7.3% with the vascular unit implementation (p = 0.002). However, the stroke case-fatality rate did not decrease despite improvements in the quality of stroke healthcare indicators.^100^
- A cluster randomized trial has assessed the effect of a multifaceted quality improvement intervention on adherence to evidence-based therapies for the care of patients with acute ischemic stroke and TIA (including case management, reminders, a roadmap and checklist for the therapeutic plan, educational materials, and periodic audit and feedback reports to each intervention cluster). The study has evaluated 1624 patients from 36 hospitals covering all Brazilian regions. The primary outcome was a composite outcome of score adherence for acute ischemic stroke and TIA performance measures, and the secondary outcomes included an all-or-none composite endpoint of performance measures. The overall mean (SD) composite outcome adherence to the score and the 10 performance measures in the intervention group hospitals as compared to the control group hospitals was 85.3% (20.1%) vs. 77.8% (18.4%) (mean difference, 4.2%; 95% CI, −3.8% to 12.2%). As a secondary endpoint, 402 of 817 patients (49.2%) at the intervention group hospitals received all the therapies they were eligible for vs 203 of 807 (25.2%) in the control group hospitals (OR, 2.59; 95% CI, 1.22-5.53; P = 0.01). The intervention did not result in a significant increase in composite adherence score for evidence-based therapies in patients with acute ischemic stroke or TIA. However, when using an all-or-none approach, the intervention resulted in improved adherence to evidence-based therapies and in improved thrombolysis rates.^101^

## Cost

### (Refer to Tables 1-6 through 1-9 and Charts 1-15 and 1-16)

- From the SUS administrative database, total expenses attributed to cerebrovascular diseases increased from 2008 to 2018, considering the clinical procedures related to hospital admissions. In 2008 and 2018, there were 159 545 and 203 066 hospitalizations due to cerebrovascular diseases, respectively, of a total of 2 042 195 admissions in the period, representing total costs of R$ 142 061 641 (2018 Int$ 136 975 201) in 2008 and R$ 286 293 302 (2018 Int$ 141 170 268) in 2018, a cumulative amount of R$ 2 498 850 166 in the referred period. There was a real increase in total costs attributed to cerebrovascular diseases in the last decade after adjustment for inflation and local currency, probably due to an increase in complexity of the treatment delivered for those conditions.
- A cost-effectiveness study assessing thrombolytic drugs in Brazil has reported that, for a one-year result, for men, the cost of the treatment with rtPA was higher than that of the conservative treatment, with the QALY gained being 0.06 for both sexes. This result is mainly directed by the cost of the medication. Part of this additional cost is compensated by the lower cost of rehabilitation and less productivity losses as early as the first 2 years, because the patients treated with rtPA presented fewer sequelae than those who received conservative treatment. After the second post-stroke year, for both sexes, treatment with rtPA (alteplase), considering direct and indirect costs, started to have a lower cost when compared to conservative treatment. From this time horizon onward, the additional cost of the medication starts to be more than compensated by the smaller productivity losses and lower social security and patient rehabilitation costs.^102^

## Prevention

- The Prospective Urban Rural Epidemiological Study has examined rates and predictors of use of evidence-based secondary prevention medications (ACEI/ARB, antiplatelets, statins, and beta-blockers) in patients with CVD, including CHD and stroke, in South American countries, including Brazil. The study has shown that fewer stroke patients received antiplatelets (24.3%), ACEI/ARB (37.6%), and statins (9.8%) as compared with CHD patients (30.1%, 36.0%, and 18.0%, respectively). This underutilization of therapies in stroke patients varied substantially among countries, with the lowest use in Colombia (no prescription of statins). When CHD and stroke patients were combined, the proportion of utilization of antiplatelets was highest in Chile (38.1%) and lowest in Argentina (23.0%). The use of ACEI/ARB and statins was higher in Brazil (46.4% and 26.4%) and lower in Colombia (26.4% and 1.4%), respectively. Among CHD and stroke participants, the use was higher in those with higher education level relative to those with none, primary, or unknown education [35.6% vs. 23.6% for antiplatelets (p = 0.002); 20.6% vs. 10.9% for statins (p = 0.0007)]. Former smokers with CHD or stroke were more likely to receive proven therapies than current smokers or those who had never smoked [35.2% vs. 26.6% and 27.7%, respectively, for antiplatelets (p = 0.039); 19.9% vs. 10.6% and 13.0% for statins (p = 0.004)]. Only 4.1% of the patients received all 4 therapies (antiplatelets, beta-blockers, ACEI/ARB, and statins), with the highest rate in Brazil (5.5%), and the lowest in Colombia (0.5%) (p = 0.02). Moreover, the use of no medication was observed in 30% of Brazilian stroke patients.^103^

## Awareness, Treatment, Control

- Several studies have shown alarming lack of knowledge about stroke risk factors, stroke treatment, and recognition of stroke symptoms as a medical emergency. In a community-based study, Pontes-Neto et al. have interviewed subjects in public places of 4 major cities in Brazil between July 2004 and December 2005, using a structured, open-ended questionnaire in Portuguese, based on a case presentation of a typical patient with acute stroke at home. The authors have found 28 different Portuguese terms to name stroke. Twenty-two percent of the interviewees did not recognize any warning sign of stroke. Only 34.6% of the interviewees answered the correct nationwide emergency telephone number in Brazil (#192). Only 51.4% of the interviewees reported they would call an ambulance for a relative with symptoms of stroke.^104^
- Falavigna et al. have used a closed-ended, self-administered questionnaire to assess the knowledge about stroke among 952 residents of the city of Caxias do Sul, Brazil. Lower income and lower education level were independent predictors of inability to recognize that stroke affects the brain. Lower income and age <50 years were independent predictors of lack of knowledge about stroke risk factors.^105^
- Panicio et al. have interviewed 104 consecutive patients with acute stroke admitted to a tertiary public hospital in São Paulo, Brazil, from March 2012 to December 2012, to evaluate their knowledge about stroke and the impact of lack of stroke awareness on late hospital arrival. Although 66.2% of the patients knew the warning signs of stroke, only 7.8% showed some knowledge about the limited time window for reperfusion therapy. Stroke severity measured by the NIHSS was the only independent predictor of early arrival.^106^
- In a community-based and cross-sectional study, Pitton Rissardo et al. have applied a stroke knowledge survey to a convenience sample of 633 passers-by of a public square from December 2015 to October 2016, in the city of Santa Maria, state of Rio Grande do Sul. Of the respondents, 33% correctly reported the meaning of the acronym “AVC” (in Portuguese, *acidente vascular cerebral* ), the most recommended term to name stroke in Portuguese by the Brazilian Society of Cerebrovascular Diseases. Around 30% of the subjects incorrectly localized stroke in the heart. Only 50% of the respondents correctly reported a warning sign of stroke. Individuals with a higher level of education were more likely to call an ambulance for a relative with stroke symptoms.^107^
- In recent years, there have been several initiatives to improve public stroke awareness in Brazil, mainly around the World Stroke Day (October 29^th^) annual campaigns led by the World Stroke Organization. Despite these efforts, only 30–40% of patients with stroke are hospitalized within 4 hours of symptom onset.^108^

## Future Research

- The Brazilian research portfolio in vascular neurology has evolved largely in recent years. Still, there are several opportunities for improvement. The most expressive community studies on stroke prevalence and incidence derive mostly from 2 cities. While both examples represent a major achievement in stroke epidemiology, there is still a need for a broader assessment, comprising a representation of all geographical regions, diverse cultures, income levels, and ethnicities.
- Additionally, there are inherent constrains related to studies relying on stroke identification using ICD codes. It is not uncommon for users to apply a broader code on admission, that might not be adjusted during hospital stay, thus not representing the actual stroke subtype (e.g., an ischemic stroke might be coded as non-specified stroke or even as TIA). With the dawn of big data technologies (e.g., text mining), additional clinical information from admission or discharge records could provide a reliable cross-reference source, thus confirming or correcting a given code.
- As a worldwide challenge, not restricted to Brazil, health services research using robust methodology, assessing not only the reality of healthcare delivery but also adequately assessing the effectiveness of healthcare policies through randomized trials, needs to become the basis for quality improvement programs at both community and population levels.

## 3. ACUTE AND CHRONIC CORONARY HEART DISEASE

### ICD-9-CM 410 to 414; ICD-10 I10 to I25.

**See Tables 3-1 through 3-3 and Charts 3-1 through 3-6**

**Table:** 

Abbreviations Used in Chapter 3	
ACCEPT/SBC	Brazilian Registry of Clinical Practice in Acute Coronary Syndromes of the Brazilian Society of Cardiology
ACS	Acute Coronary Syndrome
AMI	Acute Myocardial Infarction
BRACE	Brazilian Registry on Acute Coronary Syndrome
BYPASS	Brazilian Registry of Adult Patients Undergoing Cardiovascular Surgery
CABG	Coronary Artery Bypass Grafting
CAD	Coronary Artery Disease
CHD	Coronary Heart Disease
CI	Confidence Interval
CVD	Cardiovascular Diseases
DALY	Disability-Adjusted Life Year
DATASUS	Brazilian Unified Health System Database
GBD	Global Burden of Disease
GDP	Gross Domestic Product
IHD	Ischemic Heart Disease
MASS	Medicine, Angioplasty, or Surgery Study
MI	Myocardial Infarction
PCI	Percutaneous Coronary Intervention
PNS	National Health Survey (in Portuguese, Pesquisa Nacional de Saúde)
RBSCA	Brazilian Registry of Acute Coronary Syndrome
STEMI	ST-Elevation Myocardial Infarction
SUS	Brazilian Unified Health System (in Portuguese, Sistema Único de Saúde)
UI	Uncertainty Interval
WHO	World Health Organization
YLL	Year of Life Lost

## Overview and Prevalence

- Coronary heart disease, also known as IHD or CAD, comprises a spectrum of symptomatic and asymptomatic clinical conditions typically related to a reduction in blood flow to the heart muscle. The most common cause is atherosclerotic disease in the coronary arteries, a chronic condition with variable presentations, progressing from a long asymptomatic phase to stable angina, MI, and unstable angina. Coronary heart disease is a common cause of heart failure, with reduced or preserved left ventricular ejection fraction, ventricular arrhythmias, and sudden cardiac arrest.
- Coronary heart disease was the leading cause of death in Brazil in the last decade, for men and women. Because of its wide range of clinical presentations, the reported CHD prevalence, incidence and mortality vary widely, depending on the population and healthcare setting studied.

### Coronary Heart Disease

- According to data from the GBD 2017, the total CHD prevalence was 1.75% in Brazilian adults >20 years. Males had a higher prevalence as compared to females, 2.33% and 1.19%, respectively. For adults aged 15-49 years, the estimated CHD prevalence was 0.53%; for those aged 50-69 years, 4.34%; and for those older than 70 years, 10.99% (Chart 3-1).
- The overall age-standardized prevalence of CHD was 1.63% (1564 per 100 000 inhabitants), being 2.35% (2229 per 100 000 inhabitants) in males and 1.05% (1008 per 100 000 inhabitants) in females. This implies that there were at least ~3.3 million people living with CHD in the Brazilian Federative Units in 2017.
- There was a difference in the age-standardized prevalence of CHD among the Brazilian regions, with the highest prevalence observed in the Southeastern and Southern regions (state of São Paulo, 1617 per 100 000 inhabitants; state of Rio Grande do Sul, 1642 per 100 000 inhabitants), and the lowest, in the Northern and West-Central regions (state of Amazonas, 1407 per 100 000 inhabitants; Distrito Federal, 1404 per 100 000 inhabitants). The Northeastern region was in an intermediate position, but in a trend towards an increase in CHD prevalence in the last decades (state of Pernambuco, 1523 per 100 000 inhabitants) (Table 3-1).
- For the 1990-2017 period, the CHD prevalence increased in both sexes (from 1.08% to 1.75%), and the slope was more prominent in males than in females. This increase was probably due to population aging, because the age-standardized prevalence rates were stable in the last 2 decades (annual change, -0.25%) for both sexes (Chart 3-2).

### Stable Angina

- Regional population surveys conducted before 2000, applying the Rose Angina Questionnaire in 2 cities (Ribeirão Preto, in the state of São Paulo, and Pelotas, in the state of Rio Grande do Sul), have reported angina prevalence of 12.3% and 8.2%, respectively, in adults aged ≥ 40 years.^110 , 111^
- The Brazilian Health Survey conducted in 2003 among 5000 subjects has shown a prevalence of self-reported angina of 6.7% in Brazilian adults aged ≥18 years, and of 13% in those > 50 years. Only 72.8% of the individuals had reported adherence to medical therapy within the previous 2 weeks.^112^
- According to the 2013 PNS, an epidemiological home-based, country-wide representative survey, the overall prevalence of class I angina was 7.6% (95% CI, 7.2% - 8.0%), and of class II angina, 4.2% (95% CI, 3.9% - 4.5%).^113^
- Self-reported angina *pectoris* was more prevalent in females than in males in all studies described.
- It is important to note the higher prevalence rates observed in prospective surveys as compared to national statistics. Self-reported assessments of angina are very sensitive, but not specific to CAD, because they require neither confirmatory exams nor health reports. In addition, national statistics might underrepresent the true epidemiology of CHD, considering its asymptomatic nature.

## Incidence

- The GBD has estimated an incidence of 84 events of CAD per 100 000 inhabitants in 2017 in Brazil. The age-standardized incidence was 79 per 100 000 inhabitants, and the age-adjusted numbers were higher for males than for females, 104 and 58 per 100 000 inhabitants, respectively. There was an exponential increase in the incidence by age group, from 19 per 100 000 inhabitants for those aged 15-49 years to 198 per 100 000 inhabitants for those aged 59-69 years, and 744 per 100 000 inhabitants for those aged 70 years or older. Temporal changes from 1990 to 2017 were small, 1.03% crude, and -1.29% age-standardized annually.
- According to data from the DATASUS, in 2018, there were 142 982 new cases of AMI and ACS.
- In a systematic review from public health data in 2009, the rates of ACS and MI per 100 000 inhabitants were 38 and 29.8, respectively.^114^

## Mortality

- According to GBD 2017 estimates, there were 175 791 deaths attributable to CHD, corresponding to 13% of total deaths in Brazil (Chart 3-3).
- The crude mortality rate attributable to CHD was 83 per 100 000 inhabitants in 2017 (GBD 2017), higher for males than for females (95 and 72, respectively). As expected, rates were higher for advanced age groups: 161 per 100 000 inhabitants for those aged 50-69 years, and 837 per 100 000 inhabitants for those aged 70 years or older.
- The age-standardized mortality rate of IHD from the GBD 2017 was 80 (95% CI, 78 – 82) per 100 000 inhabitants, representing 13% of the causes of death in Brazil. Ischemic heart disease was the number one cause of death in every Federative Unit in 2017.
- Regional variations on mortality rates are significant. The lowest death rate was observed in the state of Amazonas (59 per 100 000 inhabitants), while the highest, in the state of Pernambuco (102 per 100 000 inhabitants). In all Brazilian regions, IHD has been the number one cause of death for the last 3 decades.^115^
- According to data submitted to the WHO and World Bank, in 2015, there were 111 849 deaths due to CHD in Brazil, approximately 50 per 100 000 individuals, making CHD the leading cause of mortality from 2010 to 2015.^116^
- According to the 2009 Ministry of Health data, of 962 931 deaths over the age of 30 years, 95 449 were caused by CHD, whereas 193 309 were caused by atherosclerosis.^117^
- Data from the GBD 2017 have shown a decrease in IHD mortality from 1990 to 2017 (Chart 3-4), with unadjusted annual changes of -0.15%, corresponding to a -53% (-54% to -51%) cumulative change during that period. This decrease was observed in all Federative Units, being, though, less expressive in the Northeastern region (state of Ceará, -22%) than in the Southeastern region (state of Minas Gerais, -63%). The age-standardized rate decreased from 169 per 100 000 inhabitants in 1990 to 80 per 100 000 inhabitants in 2017. This trend was similar for both sexes and across age groups (Table 3-2).
- The temporal analysis of CVD mortality between 1981 and 2001 has shown that the coefficient of mortality from CHD remained stable for women in the Northern and West-Central regions, whereas decreased in the Southern and Southeastern regions, and increased in the Northeastern region.^118^For males, there was a trend towards fewer events in the Southern and Southeastern regions.^118^
- An analysis conducted from the DATASUS, from 1990 to 2009, has shown a reduction in deaths due to CHD in Brazil.^117^The rate reduced from 195 per 100 000 inhabitants to 149 per 100 000 inhabitants for males and from 120 per 100 000 inhabitants to 84 per 100 000 inhabitants for females (Chart 3-5).
- The proportion of deaths caused by CHD has remained stable over the last decades, with reports ranging from 26% to 32%, according to the year. An ecologic study in the city of Porto Alegre, including individuals aged 45-64 years, has shown that CVD was responsible for 28.5% of all deaths in 2009. Of those, 40% were related to CHD, whose proportion was higher among individuals with lower socioeconomic status (42.7%) than among those with higher socioeconomic status (26.3%).^119^
- In a national ecologic study including individuals aged 35-64 years, from 1999 to 2001, the rate of death related to CHD was 84 ± 30 per 100 000 inhabitants. In that study, the incidence of events related directly to poverty rate and lower educational attainment.^120^Importantly, there was wide variability in the results across the 98 participating cities, probably due to data quality.

### Mortality Related to Acute Coronary Syndrome

- According to the DATASUS, there were 142 982 hospital admissions due to AMI in 2018, with an in-hospital mortality of 11%.
- Several Brazilian ACS registries have reported the outcomes of individuals admitted with ACS. In general, mortality in registries is lower than that reported in the Brazilian Health Information Systems.
- Between 2003 and 2008, the RBSCA enrolled 2693 patients, 45% of whom had AMI. The in-hospital mortality for those with unstable angina was 3.1%, whereas, for those with MI, it was 7.7%, leading to an overall mortality of 5.5%.^121^
- Results from a cross-sectional, observational, epidemiological registry of ACS patients (BRACE) published in 2012 showed an overall in-hospital mortality of 5.2% among 1150 patients from 72 hospitals included in that registry.^122 , 123^
- In a retrospective, multicenter study of 3745 patients admitted with ACS between 2010 and 2015 to a hospital in São Paulo, the in-hospital all-cause mortality was 3.3%, and 454 (12.2%) patients experienced at least one major adverse event (reinfarction, shock, bleeding, stroke or death).^124^
- In another hospital-based registry, the ERICO Study, a cohort of ACS individuals admitted to a community regional hospital in the state of São Paulo, the mortality rates at 30 days and 1 year were 4.4% and 12%, respectively.^125^
- The ACCEPT/SBC Registry, conducted from 2010 to 2011 in 47 hospitals, has enrolled 2485 ACS patients, 35% with non-STEMI and 33% with STEMI. All-cause mortality at 30 days was 1.8% for unstable angina, 3.0% for non-STEMI, and 3.4% for STEMI.^126^
- An observational longitudinal study undertaken from 2011 to 2014 in a high-complexity hospital, in the city of Belo Horizonte, including 1129 patients with STEMI and non-STEMI, has reported an in-hospital mortality of 8.7%. Of the STEMI patients, 56% received reperfusion therapy, and 67% were treated in accordance with guideline recommended practices.^127^
- In a study from the Minas Telecardio 2 Project, conducted in 2013 and 2014 in 6 emergency units in the city of Montes Claros, state of Minas Gerais, among 593 patients with ACS, the in-hospital mortality was 9.4%, ranging from 4.9% for unstable angina to 17% for STEMI cases.^128^In a STEMI care registry in the city of Salvador (RESISST), interconnected through a Regional Integrated Care Network, from January 2011 to June 2013, only 41% of the patients underwent reperfusion therapy, and the 30-day mortality rate was 15.3%.^129^
- Statewide data from the state of Sergipe collected from 2014 to 2017 have identified 707 cases of STEMI, with an in-hospital mortality of 10.9%. There was a significantly higher mortality rate for individuals admitted to public hospitals as compared to those admitted to private ones (11.9% versus 5.9%, respectively), as described in previous studies.^129 , 130^

#### A. Mortality related to percutaneous coronary interventions

- According to the DATASUS, in 2018, 10 811 primary angioplasties were performed for AMI, with an in-hospital mortality of 6.3%, and mean length of stay of 5.1 days. When all other coronary procedures are considered, 78 575 coronary angioplasties are identified, with an in-hospital mortality of 2.96%, and mean hospital length of stay of 4.5 days.
- According to the DATASUS, in a dataset of 3874 individuals, the in-hospital mortality related to PCI was 2.33% between 2005 and 2008.^121^That rate was lower in the Southeastern region (2.03%) and higher in the Northern region (3.64%) (P<0.001). Procedure volume was not associated with outcomes in those analyses.^121^
- In a cohort study undertaken from 2009 to 2013, assessing 4806 patients undergoing PCI (Brazilian PCI multicenter registry) in 8 tertiary referral medical centers, considering all clinical conditions (69% with recent MI), the in-hospital mortality was 2.6%.^131^
- The ACCEPT/SBC Registry enrolled 2485 ACS patients from 2010 to 2011, mostly in tertiary care centers; more than 90% of them underwent cardiac catheterization and cardiovascular surgery on the site. Among those patients, the revascularization rate was 39% for unstable angina, 54% for non-STEMI, and 78% for STEMI. For the STEMI group, reperfusion therapy was used in 88% of the patients, and most patients (73%) received primary angioplasty. Treatment delay was, on average, 125 ± 90 minutes.^126^
- Another PCI registry, including 1249 consecutive patients in 2009, has shown total mortality of 2.3%, ranging from 0.2% for stable angina patients to 6.1% for those with STEMI.^132^
- In another frame series of PCI in public hospitals from 2005 to 2008, 166 514 procedures were performed in 180 hospitals. Average in-hospital mortality was 2.33%, ranging from 0% to 11.35%. This rate was lower in the Southeastern region (2.03%) and higher in the Northern region (3.64%). The in-hospital mortality rate was 2.33% in high-volume hospitals, accounting for 101 218 (60.8%) of the PCI, 2.29% in medium-volume hospitals, and 2.52% in low-volume hospitals. Mortality was higher in females and among those older than 65 years.^133^
- Most reports originate from public institutions, and data from private hospitals are limited. An analysis of 440 procedures performed between 2013 and 2014 in one public and one private hospital in the city of Rio de Janeiro has shown low mortality (0.5%), with similar rates in both institutions.^134^
- Data on long-term survival rates for patients undergoing PCI are scarce. In an analysis from procedures performed in the state of Rio de Janeiro between 1999 and 2000 in all public hospitals, including 19 263 individuals, one-year survival was 93% and 15-year survival, 57%. In that study, women had a higher survival rate than men within 15 years after PCI.^135^

#### B. Mortality related to surgical revascularization

- According to 2017 data from the National Health System, 21 474 CABG were performed in public institutions in Brazil, with an in-hospital mortality of 5.37% and mean hospital length of stay of 12.2 days (Table 1-7).
- The BYPASS Project is an ongoing database established in 2015 by the Brazilian Society of Cardiovascular Surgery and involves 17 institutions representing all Brazilian regions. Among 2292 patients enrolled until November 2018, who underwent isolated or combined CABG, the in-hospital mortality was 2.8%, while 5.3% stayed on mechanical ventilation for more than 24 hours, and 1.2% had an in-hospital stroke.^136 , 137^
- The MASS II trial was a single-center randomized clinical trial designed to compare the long-term effects of medical therapy, angioplasty, or surgical strategies for treating multivessel CAD with stable angina and preserved ventricular function, conducted before 2007. The in-hospital mortality rates for PCI and CABG were 2.4% and 2.5%, respectively.^138^The 10-year survival rates were not significantly different between the groups: 74.9% for CABG, 75.1% for PCI, and 69% for medical therapy (p=0.089).^139^In another trial (MASS III), similar 10-year survival rates have been described.^140^
- Several other single-center experiences, with both retrospective and prospective analyses, have reported in-hospital mortality for patients who underwent CABG ranging from 1.9% to 8.7%.^141 - 143^

## Burden of Disease

- The GBD 2017 has estimated 1736 (95% CI, 1689 – 1779) DALYs lost per 100 000 individuals due to CHD, with lower rates for females (1298; 95% CI, 1340 – 1250) than for males (2194; 95% CI, 2112 – 2258). This loss in DALY corresponded to 6.1% (95% CI, 5.5% – 6.7%) of all DALYs lost. These rates significantly decreased in the last 2 decades in all regions (annual change of -2.51%) (Chart 3-6).
- From 1990 to 2017, there was a decline in DALYs lost for both males (-47%) and females (-52%) in all Federative Units. More expressive relative reductions were observed in the Southern and Southeastern states, while smaller reductions were observed in the Northern and Northeastern states (Table 3-3).
- The rate of YLLs due to IHD was 1653 per 100 000 individuals (95% CI, 1607 – 1688), with lower rates for females than for males. These YLLs corresponded to 9.7% (95% CI, 9.4% - 9.9%) of all YLLs in the GBD 2017.

## Healthcare Utilization and Cost

### (Refer to Tables 1-6 through 1-9 and Charts 1-15 through 1-16)

- Analysis of the administrative database from the SUS has shown that, in 2018, the total amount reimbursed for coronary interventional procedures was R$ 569 314 580 (Int$ 280 727 110), of which, R$ 73 429 322 (13%) (Int$ 36 202 821) were related to primary angioplasties. The mean value paid per patient was R$ 6369 (Int$ 3230). Regarding CABG, the total amount was R$ 275 110 234 (Int$ 135 655 933), corresponding to a median value of R$ 13 307 (Int$ 6561) per surgical hospitalization.
- Unadjusted reimbursed values associated with coronary revascularization procedures (codes for angioplasty and CABG) increased significantly from 2008 to 2018, although in different magnitudes. For percutaneous angioplasties, the mean values raised by 16% (from R$ 5437 to R$ 6351), and for CABG, by 46% (from R$ 9192 to R$ 13 140) during that period.
- A global modelling approach was performed in 2015 to assess the economic (health system and productivity) impact of four heart conditions in Brazil, providing estimates of the annual cost for the year 2015. The four heart conditions were estimated to affect ˜45.7 million people in Brazil, corresponding to 32.0% of the adult population. Myocardial infarction implied the greatest financial cost, with an estimated prevalence of 0.2% (334 978 cases), health system cost per case of US$ 48 118, and productivity cost of US$ 18 678.^115^
- The annualized cost for an individual with chronic CAD was estimated to be around R$ 2733 ± 2307 by the SUS, with the outpatient cost representing 54% of the total. For private insurance plans, the cost was estimated to be R$ 6788 ± 7842, of which, 69% related to inpatient costs. For outpatient costs, medications were responsible for R$ 1154, representing, for public and private payers, 77% and 55% of the outpatient costs, respectively, and 42% and 17% of the total cost, respectively.^144^
- Another registry from a IHD clinic of a public hospital has shown a mean annual cost of outpatient management of US$ 1521 per patient (2015 currency). The mean cost per hospitalization was US$ 1976, and the expenses were higher in the first and last years of follow-up. Unstable angina, revascularization procedures, diabetes, hypertension, and obesity were predictors of higher hospitalization costs.^145^
- The annual healthcare costs of individuals with CVD are three times higher than those for individuals without CVD in the public healthcare system (R$ 4626 vs. R$ 1312). In the private healthcare sector, the difference is even higher (R$ 13 453 vs. R$ 1789 – adjusted to 2014).^146^
- According to data from DATASUS, from 2008 to 2014, 4 653 884 cardiac diagnostic procedures were performed in Brazil, including 3 015 993 ECGs, 862 627 invasive angiographies, and 669 969 nuclear studies, leading to an overall cost of US$ 271 million. In this national geospatial evaluation of health access, CHD mortality was associated with lower income, and performance of fewer nuclear tests and of more exercise ECG tests and cardiac catheterization procedures.^147^
- According to administrative claims data from the SUS, in the last decade, there was a 40% increase in the absolute number of primary angioplasties for AMI management, from 7648 in 2008 (4.03 per 100 000 inhabitants) to 10 811 (5.19 per 100 000 inhabitants) in 2018. A similar trend was observed for hospital admissions due to CHD. The number of coronary angioplasties almost doubled during that period, while the number of CABG remained stable.
- Regarding the angioplasties not classified as primary by the administrative claims of the SUS (procedure codes: 0406030073, 0406030014, 0406030065, 0406030022, 0406030030), the proportion of those procedures that occurred in the context of a hospitalization for MI increased from 2008 to 2018 (12% to 31%, respectively). Moreover, coronary angioplasties performed during hospitalizations for AMI increased by 518%, while for chronic CAD, they increased only by 70%, revealing a change in the profile of patients submitted to coronary interventions, following the current guidelines recommendations, in which angioplasties are more extensively recommended for acute instead of chronic CAD. In fact, in 2018, 70% of all angioplasties assigned a procedure code from those cited above occurred in the context of acute CAD.
- A cost analysis of 101 patients undergoing PCI in 2014 and 2015 has shown a median cost of R$ 6705 ± 3116 per patient. Those costs were lower for elective PCI, R$ 5085 ± 16, than those for ACS, R$ 6854 ± 3396.^148^
- A quantitative, descriptive and cross-sectional study carried out in a philanthropic hospital of São Paulo, assessing 1913 consecutive patients who underwent CABG in 2012, has reported an average total cost per patient of US$ 7993 [median, US$ 6463], revenue from the public health system of US$ 3450 [3159], and an estimated deficit of -51% of the total cost for the providers.^149^
- A retrospective analysis of medical claims of beneficiaries of health plans has been performed considering hospitalization costs for patients admitted with ACS between 2010 and 2012. The mean cost per patient on medical therapy only was R$ 18 262, for those submitted to PCI, it was R$ 30 611, and for those submitted to CABG, R$ 37 455.^150^

## Future Research

- Additional data are needed for further understanding of the epidemiological distribution of CHD in Brazil, in particular:

— Development of nationwide databases aiming to gather accurate and real time information on the epidemiology of the distinct clinical presentations of CHD, including delivery of care, and performance and outcome measurements.

— Systematic reviews of the prevalence and mortality rates of ACS, stable patients, and post-PCI and CABG, including representative samples of all geographical areas of the country.

— Assessment of the effectiveness of structured nationwide programs for quality and performance measurement of different providers (public, non-for-profit, and for-profit) to understand the current situation, as well as for designing strategies aimed at reducing CVD morbidity and mortality.

- Additional economic and cost-effectiveness analyses of the impact of CHD and its diagnostic and therapeutic interventions are required, from a macro level and using micro costing methods for both the public and the private healthcare systems.
- Development of structured programs to assess the prevalence, the incidence, and the clinical and economic impact of chronic CHD in the outpatient setting is necessary.

## 4. CARDIOMYOPATHY AND HEART FAILURE

### ICD-10 I42; I50; B57.2.

**See Tables 4-1 through 4-14 and Charts 4-1 through 4-10**

**Table:** 

Abbreviations Used in Chapter 4	
BREATHE	I Brazilian Registry of Heart Failure
ChCM	Chagas Cardiomyopathy
ChD	Chagas Disease
CI	Confidence Interval
DALY	Disability-Adjusted Life Year
FU	Federative Unit
GBD	Global Burden of Disease
HCM	Hypertrophic Cardiomyopathy
HF	Heart Failure
HF-PEF	Preserved Ejection Fraction Heart Failure
HF-REF	Reduced Ejection Fraction Heart Failure
HR	Hazard Ratio
ICD-10	International Statistical Classification of Diseases and Related Health Problems, 10th Revision
IQR	Interquartile Range
LVEF	Left Ventricular Ejection Fraction
NChC	Non-Chagas Cardiomyopathies
OR	Odds Ratio
PAR	Population Attributable Risk
REMADHE	Repetitive Education and Monitoring for ADherence in Heart Failure
SDI	Sociodemographic Index
SEADE	Data Analysis State System Foundation (in Portuguese, Fundação Sistema Estadual de Análise de Dados)
SIM	Brazilian Mortality Information System (in Portuguese, Sistema de Informações sobre Mortalidade)
SUS	Brazilian Unified Health System (in Portuguese, Sistema Único de Saúde)
UI	Uncertainty Interval
US	United States
YLD	Year Lived with Disability

## Cardiomyopathy and Myocarditis

### Prevalence and Incidence

- According to the GBD Study 2017 estimates, the age-standardized prevalence of cardiomyopathy and myocarditis increased in Brazil by 9.4% (95 UI, 15.3-4.1) from 1990 to 2017, passing from 102.8 (95% UI, 82.5-125.7) to 112.4 (95% UI, 92.2-134.2), respectively (Chart 4-1.A and Table 4-1). In absolute number, the prevalence estimates of cardiomyopathy and myocarditis in Brazil rose from less than 100 000 in 1990 to over 200 000 in 2017, mainly due to population growth and aging (Chart 4-1.B). The cardiomyopathy and myocarditis prevalence was greater in women (115; 95% UI, 95-137) than in men (109; 95% UI, 88-132) in 2017, but the prevalence increase was higher for men in that period, the increase percentage being 6.9 (95% UI, 0.2-14.2) for women and 12.3 (95% UI, 5.4-20) for men.
- According to the GBD Study 2017 estimates, the prevalence of cardiomyopathy and myocarditis is highly variable amongst the Brazilian FUs, and the percent change varied unevenly between 1990 and 2017 (Table 4-1). In 2017, the highest rates were observed in the states of São Paulo and Roraima, and the Distrito Federal. From 1990 to 2017, decreased age-standardized prevalence rates were observed in the states of Rio de Janeiro, Rio Grande do Sul, Santa Catarina, and Espírito Santo, while the age-standardized prevalence increased in all other FUs.
- Regarding age-standardized incidence rates per 100 000 per-year, the GBD Study 2017 estimates were 46.3 (95% UI, 41.5-52.1), in 1990, and 46.7 (95% UI, 41.8-52.6), in 2017, with a small change of 0.8% (95% UI, -0.3 to 1.8) during that period (Table 4-2). Absolute numbers of incident cases were 54 520 (95% UI, 48 574-61 321), in 1990, and 103 879 (95% UI, 92 496-117 294), in 2017; that increase is related to population growth and aging. Table 4-3 depicts the incidence rates of cardiomyopathy and myocarditis, per 100 000 inhabitants, by age, for both sexes, in 1990 and 2017, and the percent change of the rates. There is an almost 3-fold stepwise increase in the incidence rates from the 15-49 year group to the 50-69 year group, as well as from the latter to the 70+ year group, and these increases are similar for women and men. From 1990 to 2017, the incidence increased in all age groups for women, while tended to decrease in most age groups for men.

### Mortality

- According to the GBD Study 2017 estimates, the mortality rates due to cardiomyopathy and myocarditis seemed to increase in the 1990s but decreased in the following 2 decades (Chart 4-2). As shown in Table 4-4, the mortality rates were 10.9 (95% UI, 9.57-11.38), in 1990, and 8.59 (95% UI, 8.16-9.93), in 2017, per 100 000 inhabitants, a decrease of 21.2% (95% UI, -26.8 to -2.6). Despite this decrease in the mortality rates, the number of deaths due to cardiomyopathy and myocarditis increased in that period due to population growth and aging. Cardiomyopathy and myocarditis were responsible for 9734 (95% UI, 8417-10 163) deaths in 1990, rising to 18 812 (95% UI, 17 885-21 745) deaths in 2017. The GBD Study 2017 estimates of mortality rates due to cardiomyopathy refer to cases with cardiomyopathy listed as an underlying cause of death. Deaths due to HF resulting from other specific causes are attributed to the underlying disease, i.e., deaths related to ischemic cardiomyopathy are coded as due to ischemic heart disease. Moreover, for the GBD project, HF is not considered a primary cause of death, and all deaths coded as related to HF are recoded to the baseline condition (see below).
- Table 4-4 also depicts the total number of deaths and age-standardized mortality rate (per 100 000 inhabitants) due to cardiomyopathy and myocarditis, as well as the percent change (%), by FU, in Brazil, in 1990 and 2017. Most FUs had decreased mortality rates, with the highest percentages of reductions observed between 1990 and 2017 in the states of Paraná and Goiás. On the other hand, increased mortality rates from 1990 to 2017 were observed only in 3 FUs, with the highest increase, of 46.2% (95% UI, 12.6-63.3), observed in the state of Rio de Janeiro. In 2017, the FUs with the lowest mortality rates (below 5.0) were the states of Acre, Amazonas, Maranhão, Pará, Rio Grande do Norte, and Rio Grande do Sul.
- Table 4-5 shows mortality rates due to cardiomyopathy and myocarditis according to sex and by age groups, from the GBD Study 2017 estimates. Women had lower age-standardized mortality rates, as well as a more pronounced reduction from 1990 to 2017. The mortality rates due to cardiomyopathy and myocarditis in women were 9.20 (95% UI, 8.81-9.93) per 100 000 inhabitants, in 1990, and 6.3 (95% UI, 6-6.6) per 100 000 inhabitants, in 2017, a reduction of 31.3% (95% UI, -35.5 to -26.2). Rates in men were 12.83 (95% UI, 9.96-13.67) per 100 000 inhabitants, in 1990, and 11.27 (95% UI, 10.46-14.39) per 100 000 inhabitants, in 2017, a variation of 12.1% (95% UI, -20.9 to 28.2). As expected, the highest mortality rate was observed in the 70+ year group, with rates of 84.2 (95% UI, 74.2-89.1) per 100 000 inhabitants, in 1990, and of 76.6 (95% UI, 72.0-89.9) per 100 000 inhabitants, in 2017. For the 50-69 year group, rates were 21.2 (95% UI, 17.7-22.5) per 100 000 inhabitants, in 1990, and 16.5 (95% UI, 15.3-19.3) per 100 000 inhabitants, in 2017. Overall, mortality rates decreased from 1990 to 2017 in most age groups, remaining stable in the 15-49 year group.
- The GBD Study 2017 uses the SDI as an estimate of the socioeconomic level of a location. Chart 4-3 depicts the correlation between the SDI and the age-standardized mortality rate due to cardiomyopathy and myocarditis, per 100 000 inhabitants, both in 1990 and 2017. The correlations observed in 1990 and 2017 were similar and nonsignificant.
- In a study reporting data from the SEADE, state of São Paulo, cardiomyopathies were responsible for a total of 3571 deaths, representing 23.3% of the HF-related deaths in 2006: dilated cardiomyopathy accounted for 17.2% of the deaths; alcoholic cardiomyopathy, for 0.45%; and restrictive cardiomyopathies, for 0.37%. Chagas disease and alcoholic cardiomyopathy were responsible for 7.8% and 0.45% of the HF-related deaths, respectively.^153^
- Data of specific cardiomyopathies are scarce. A cohort study of 214 patients with HCM has reported data from a 7-year follow-up in a tertiary hospital in São Paulo, Brazil. The mean age was 37±16 years, and 52% were women. There were 22 deaths (10%), 15 directly related to HCM (11 sudden deaths). The cumulative survival rates were 94.5% at 5 years, 91% at 10 years, and 87.9% at 15 years, with an annual mortality rate of 1%, which is a low mortality considering that the study was conducted in a referral center.^154^

### Burden of Disease

- According to the GBD 2017 estimates, the trends of age-standardized DALY rates due to cardiomyopathy and myocarditis were similar to those of mortality, with a small increase in the 1990s and a decrease during the following decades. Chart 4-4 depicts DALY rates per 100 000 inhabitants, 1990-2017, in Brazil and its regions. The West-Central region had the highest DALY rates during the first 2 decades, with a reduction after that, mostly after 2004. In 2017, the Southeastern region had the highest DALY rates, while the Northern and Southern regions had the lowest DALY rates. As shown in Table 4-6, age-standardized DALY rates were 286 (95% UI, 247-301), in 1990, and 222 (95% UI, 211-251), in 2017, per 100 000 inhabitants, a decrease of 22.4% (95% UI, -27.6 to -7). These changes are similar to those observed in the mortality rates. Despite that decrease in DALY rates, cardiomyopathy and myocarditis resulted in 328 636 (95% UI, 283 325-346 746) DALYs in Brazil, in 1990, and in 490 572 (95% UI, 465 903-556 886) DALYs in Brazil, in 2017, which represents 0.81% of all DALYs.
- Table 4-7 shows the DALY rates due to cardiomyopathy and myocarditis according to sex and by age groups, from the GBD Study 2017 estimates. The age-standardized DALY rates were lower in women, and the reduction from 1990 to 2017 was more pronounced in women. The DALY rates, for women, were 234 (95% UI, 223-247) per 100 000 inhabitants, in 1990, and 153 (95% UI, 145-161) per 100 000 inhabitants, in 2017, evidencing a reduction of 34.3% (95% UI, -38.6 to -30). The DALY rates, for men, were 343 (95% UI, 262-366) per 100 000 inhabitants, in 1990, and 299 (95% UI, 280-363) per 100 000 inhabitants, in 2017, evidencing a reduction of 12.8% (95% UI, -21 to 17.6). As expected, the highest DALY rate was observed in the 70+ year group, followed by that of the 50-69 year group. Overall, DALY rates decreased from 1990 to 2017 in most age groups, increasing only in the 15-49 year group for men.
- Similarly to that observed for age-standardized mortality rates, there was no correlation between the SDI and the cardiomyopathy and myocarditis DALY rates.

### Chronic Chagas Disease and Chagas Cardiomyopathy

#### Prevalence and Incidence

- The 2010 prevalence of ChD in Brazil was estimated at 1 156 821 by the World Health Organization,^155^which is the last official available estimate, published in 2015. According to this statement, the estimated number of subjects with ChCM in Brazil is 231 364.^3^Those numbers reveal a significant decreasing trend of ChD human cases in Brazil in relation to previous estimates, which was attributed to various factors, mainly the almost complete interruption of the vectorial and transfusion-related transmission in Brazil.
- According to the GBD Study 2017 estimates, the age-standardized prevalence of ChD markedly reduced in Brazil, by 44% (95% UI, 42-27), from 1990 to 2017: from 1811 (95% UI, 1531-2131) per 100 000 inhabitants, in 1990, to 1011 (95% UI, 843-1198) per 100 000 inhabitants, in 2017. The prevalence of ChD in Brazil in 2017 was higher in men [1029 (95% UI, 863-1205)] than in women [991 (95% UI, 824-1186)].
- In a systematic review of population-based studies on ChD prevalence in Brazil, performed from 1980 to September 2012, 42 articles with relevant prevalence data were identified from a total of 4985 references.^156^The pooled estimate of ChD prevalence across studies for the entire period was 4.2% (95% CI, 3.1-5.7), ranging from 4.4% (95% CI, 2.3-8.3) in the 1980s to 2.4% (95% CI, 1.5-3.8) after 2000. The estimated ChD prevalence for males and females was similar (4.1% [95% CI, 2.6-6.6], 4.2% [95% CI, 2.6-6.8], respectively). The highest pooled prevalence was observed in individuals aged >60 years (17.7%; 95% CI, 11.4-26.5), and in the Northeastern (5.0%; 95% CI, 3.1-8.1) and Southeastern (5.0%; CI, 2.4-9.9) regions and in mixed (urban/rural) areas (6.4%; 95% CI, 4.2-9.4). About 4.6 million (95% CI, 2.9-7.2 million) people are estimated to have been infected with *T. cruzi* in 2010. These estimates are much higher than those from the World Health Organization for 2010.^155^The authors have observed a high degree of heterogeneity in most pooled estimates (I(2)>75%; P<0.001).
- In the NIH REDS-II Chagas retrospective cohort study, initially healthy blood donors with an index T. *cruzi* -seropositive donation and age, sex, and period-matched seronegative donors were followed up for 10 years.^157^The differential incidence of cardiomyopathy was of 1.85 per 100 person-years attributable to *T. cruzi* infection.

#### Mortality

- According to the GBD Study 2017, the number of deaths due to ChD in Brazil has decreased over the past decades (Chart 4-5). In the 1990s, ChD was responsible for 7049 (95% UI, 6816-7323) deaths, which reduced to 5493 (95% UI, 5221-6015) in 2017. The age-standardized mortality rate showed a more striking decrease (-67.5% change), from 7.3 (95% UI, 7.0-7.6) deaths per 100 000 inhabitants in 1990 to 2.5 (95% UI, 2.3-2.7) per 100 000 inhabitants in 2017, accounting for 0.4% of all deaths in the country. Men had higher age-standardized mortality rates in 2017 than women (3.1, 95% UI, 2.9-3.4; and 1.9, 95% UI, 1.8-2.1, respectively).
- Table 4-8 demonstrates the total number of deaths due to ChD, the age-standardized mortality rates (per 100 000 inhabitants), for both sexes, and the percent change (%), by FU, in Brazil, in 1990 and in 2017. There is striking variability among the FU regarding the number of deaths and death rates for both years. In 1990, higher mortality rates (> 10 per 100 000 inhabitants) were observed in the states of Goiás, Minas Gerais, and Bahia, and in the Distrito Federal, with a peak in the state of Goiás (60 per 100 000 inhabitants, 95% UI, 58-66). All FUs showed a reduction in mortality rates, varying from 43% (95% UI, 52-33) in the state of Bahia to 75% (95% UI, 78-72) in the states of Minas Gerais and Goiás. Tables 4-9 and 4-10 reveal data stratified by sex.
- The reduction in mortality rates was more pronounced (80% change, UI 82-77) in the age range of 5-14 years, from 2.6 (UI 2.5-2.7) to 0.5 (UI 0.5-0.6) per 100 000 inhabitants. For the other age ranges, most deaths occurred in individuals aged 70+ years, who presented the lowest percent reduction (47%, UI 51-42) during the 1990-2017 period: from 41 (UI 39-43) to 22 (20-24) per 100 000 inhabitants. The decrease in age-standardized mortality rate per 100 000 inhabitants correlates with the SDI of the Brazilian FUs (R2 = 0.40, p=0.01), and the FU with the highest SDI in 1990 presented the highest percent decrease in age-standardized mortality rate from 1990 to 2017 (Chart 4-6).
- Several population-based studies have shown a reduction in mortality due to ChD in Brazil in the last decades. Martins-Melo et al.^158^have reported that nationwide standardized mortality rates reduced gradually, from 3.78 (1999) to 2.78 (2007) deaths/year per 100 000 inhabitants (-26.4%). Nóbrega et al.^159^have shown that the nationwide standardized mortality rate decreased by 32.4%, from 3.4% in 2000 to 2.3% in 2010. The mortality rate due to cardiac involvement decreased in all regions of Brazil, except for the Northern region, where it increased by 1.6%. The Northeastern region had the smallest, and the West-Central region had the largest decrease. Simões et al.^160^have studied the evolution of ChD mortality in Brazil from 1980 to 2014 and have forecasted the mortality from years 2015 to 2034. Those authors have estimated a progressive decline in ChD mortality, which would be highest among the young. The expected average reduction was 76.1% as compared to the last period observed (2010-2014) and the last period predicted (2030-2034). The West-Central, Southeastern and Southern regions had a reduction in the rate of ChD deaths between 2000 and 2014. The mortality rate in the Northeastern region did not statistically differ in any period analyzed, while, in the Northern region, it showed an increasing trend.
- In a study analyzing all death certificates of individuals who died between 1999 and 2007 in Brazil,^158^based on the nationwide SIM, ChD was mentioned in 53 930 (0.6%) death certificates, as an underlying cause in 44 537 (82.6%) death certificates, and as an associated cause of death in 9387 (17.4%) death certificates. Acute ChD was responsible for 2.8% of the deaths. The mean standardized mortality rate was 3.36 per 100 000 inhabitants/year. This is 21% higher than the mortality rate considering merely the underlying cause of death (2.78 deaths per 100 000 inhabitants/year). The proportional mortality considering multiple causes of death was 0.6%. Individuals who died from ChD were predominantly males (57%), aged over 60 years (62.8%), and residing in the Southeastern region (53.6%). The West-Central region had the highest proportional mortality of all regions (2.17%).^158^
- In the same database, calculating the mean mortality rate for each municipality of residence and using Empirical Bayesian smoothing, spatial analysis has identified a large cluster at high risk for mortality due to ChD, involving 9 states in the central region of Brazil (Chart 4-7).^161^
- Nóbrega et al., in a descriptive study using data from the SIM of all individuals who died of ChD in Brazil between 2000 and 2010, have observed that, in the 2000-2010 period, most of the deaths (85.9%) due to ChD occurred in males aged > 60 years and were caused by cardiac involvement. During the studied period, the mortality rate decreased in all age ranges, except for that of 80 years and over (Chart 4-8).^159^
- In a retrospective cohort study, probabilistic linkage was used to identify among blood donors from 1996 to 2000 (2842 seropositive and 5684 seronegative for ChD) those who died until 2010.^162^The authors have identified 159 deaths among the 2842 seropositive blood donors (5.6%) and 103 deaths among the 5684 seronegative blood donors (1.8%). Chagas seropositive donors had a 2.3 times higher risk of death due to all causes (95% CI, 1.8-3.0) than seronegative donors. Among seropositive donors, only 26 had the ICD-10 code indicating ChD as the underlying cause of death (B57.0/B57.5).^162^The authors have concluded that ChD is an underreported cause of death in the Brazilian mortality database.
- Ayub-Ferreira et al. have compared the mechanism of death in HF due to ChCM with that of other etiologies in a prospective clinical trial, the REMADHE trial, which included patients aged 18 years or older, with irreversible chronic HF for at least 6 months and LVEF of less than 50%. Of the 342 patients analyzed, 185 (54.1%) died. Death occurred in 56.4% of ChD patients and in 53.7% of non-ChD patients. Of all ChD group deaths, 48.4% were related to HF worsening, 25.7% to sudden death, and 6.4% to stroke. The cumulative incidence of all-cause mortality and of HF mortality was significantly higher in ChD patients as compared to non-ChD patients.^163^There was no difference in the cumulative incidence of sudden death mortality between the two groups. In severe Chagas heart disease, progressive HF is the most important mechanism of death.
- In the Bambuí Cohort Study, a large population-based cohort study of elderly residents of an endemic area for ChD, 1479 subjects aged 60 years and over (38.1% with *T. cruzi* positive test) were followed up from 1997 to 2007. During a mean 8.72-year follow-up, 567 participants died. *T. cruzi* infection was a predictor of mortality among cohort members, and this association remained largely significant after adjustments for age, sex, and conventional cardiovascular risk factors (HR = 1.56; 95% CI, 1.32-1.85). Overall, the PAR for mortality due to *T. cruzi* infection was 13.2% (95% CI, 9.8-16.4).^164^
- Nadruz et al. have studied the temporal trends in PAR of ChCM for 2-year mortality among patients with HF enrolled in 2002-2004 (era 1) and in 2012-2014 (era 2) in a Brazilian university hospital. They have prospectively studied 362 (15% with ChCM) and 582 (18% with ChCM) HF patients with ejection fraction ≤ 50% in eras 1 and 2, respectively, and have estimated the PAR of ChCM for 2-year mortality. Although the absolute death rates decreased over time in the ChCM and NChC groups, the PAR of ChCM for mortality increased among patients with HF [PAR_(era 1)_= 11.0 (95% CI, 2.8-18.5%); PAR_(era 2)_= 21.9 (95% CI, 16.5-26.9); p=0.023 versus era 1], driven by increases in the HR associated with ChD.^165^

#### Burden of Disease

- Using findings from the GBD Study 2016, a study has shown that, in 2016, 141 640 DALYs (95% UI, 129 065-155 941) due to ChD were estimated in Brazil, with a relative reduction of 36.7% as compared to 1990 (223 879 DALYs; 95% UI, 209 372-238 591). Age-standardized DALY rates declined at the national level (-69.7%) and in all Brazilian FUs between 1990 and 2016, but with different regional patterns (Chart 4-9). The decrease in the DALY rates was driven primarily by a consistent reduction in the years of life lost rates, the main component of total DALY for ChD. The highest fatal and non-fatal burden due to ChD was observed among males, the elderly, and in those Brazilian FUs encompassing important endemic areas for vectorial transmission in the past, such as the states of Goiás, Tocantins, Minas Gerais, and Bahia, and the Distrito Federal.^166^

## Heart Failure

- Because HF is not considered an underlying cause of death (i.e., garbage code) in the GBD Study, all deaths attributed to HF in death certificates are reclassified and/or redistributed to other causes, according to the GBD method. As such, there is no data from GBD on mortality from HF. Because HF is classified by the GBD as an “impairment”, the only indicators we have for HF from the GBD is prevalence and YLDs, which is the morbidity component of DALYs.

### Prevalence and Incidence

- According to the GBD Study 2017 estimates, age-standardized prevalence of HF changed, in Brazil, from 818 (95% UI, 718-923), in 1990, to 772 (95% UI, 680-875), in 2017, with a decrease of 5% (95 UI, -7.1 to -3) in the period (Table 4-10). In absolute numbers, estimates for the prevalence of HF in Brazil rose from 0.67 million in 1990 to almost 1.7 million in 2017, mainly due to population growth and aging. The prevalence of HF is variable amongst Brazilian FUs, and the percent change varied unevenly between 1990 and 2017 (Table 4-11). In 2017, the highest rates were observed in the state of Rio Grande do Norte, and the lowest, in the state of Acre. From 1990 to 2017, decreased age-standardized prevalence rates were observed in most FUs, and increases in rates occurred in 8 FUs, mostly in the Northeastern region.
- Table 4-12 depicts HF prevalence according to sex and age groups, from the GBD Study 2017 estimates. The HF prevalence was higher in women (795; 95% UI, 694-901) than in men (751; 95% UI, 656-845) in 2017, and the reduction in prevalence from 1990 to 2017 was more pronounced in men [the percentage of decrease was 7.5 (95% UI, -10.2 to -4.8) for men and 3.2 (95% UI, -6.5 to -0.1) for women]. Regarding age groups, there is a 10-fold increase in the incidence rates from the 15-49 year group to the 50-69 year group, as well as a 6-fold increase from the latter to the 70+ year group, and these increases are similar for women and men. From 1990 to 2017, the prevalence increased only in the 15-49 year group, while decreased in the others, probably associated with increased ischemic events in that age group.
- A systematic review has evaluated the burden of HF in Latin America and has included 143 articles published between January 1994 and June 2014, with at least 50 participants aged ≥ 18 years; most studies included (64%) were from Brazil.^167^The patients’ mean age was 60±9 years, and the mean ejection fraction was 36±9%. The prevalence of HF was 1% (95% CI, 0.1-2.7). Of the studies included, only one assessed incidence, with 1091 individuals identified through multi-stage probability sampling in the city of Porto Alegre, Brazil. The mean age was 42.8±16.9 years, and 55% were women. The incidence of HF in the single population study providing this information was 199 cases per 100 000 person-years.^168^
- In a population-based study in primary care of a medium-sized city in Brazil, 633 individuals aged ≥ 45 years were randomly selected and registered in a primary care program of a medium-sized city in Brazil. The mean age was 59.6±10.4 years, and 62% were females; the prevalence of symptomatic HF (stage C) was 9.3%, and the prevalence of stage B HF (structural abnormalities) was 42.7%. Of the patients with HF, 59% presented with HF-PEF and 41% presented with HF-REF.^169^
- Another population-based study of residents in Zona da Mata, state of Minas Gerais, has involved 7113 frail elderly. The mean age was 72.4 ± 8.0 years, 67.6% were women, and the prevalence of HF was 7.9%.^170^
- In a study that included 166 patients from the rural area of Valença, state of Rio de Janeiro, the mean age was 61 *±14 years, and 51% were men. The main etiologies were hypertensive and ischemic, and 51% of the patients had HF-REF, showing characteristics similar to those of cohorts from non-rural tertiary centers.* ^171^

### Mortality

- In a study evaluating data from the SIM, from 2008 to 2012, HF was a frequently used garbage code in Brazil. It was listed as the underlying cause of death in 123 268 (3.7%) of those records and as a multiple cause of death in 233 197 (7%). By using 2 redistribution methods to specific causes of death, only 38.7-44.8% could be reclassified to a defined cause of death with the principal diagnosis, depending on the reclassification method.^172^Although HF should not be considered the underlying cause of death and rather be in the chain of events that led to death, any analysis of SIM data that uses HF as the underlying cause of death from death certificates must be interpreted with caution, because it may be wrongly estimating the true burden of HF.
- Data obtained from the SEADE for mortality in the state of São Paulo in 2006 evaluated 242 832 deaths in estimated 41 654 020 inhabitants.^153^Heart failure and etiologies associated with HF (except primary valvular disease) were responsible for 6.3% of the total deaths. For these data, there was no distribution or reclassification of the underlying causes of death, and all etiologies associated with HF were included when considering the impact of HF on total mortality.
- A study of mortality due to HF in 3 states of Brazil (Rio de Janeiro, São Paulo, and Rio Grande do Sul) has included data from 2 960 857 death certificates from 1999 to 2005. The percentages of death due to HF were 3.0% in the restricted form (HF as the underlying cause of death) and 9.0% in the comprehensive form (HF mentioned in any line of the death certificate) in 1999. The percentages decreased over time and were 2.4% and 8.6%, respectively, in 2005. The mortality rates decreased in most age groups, except in the group aged 80 years or older. The rates increased with age and were clearly higher among men up to 80 years of age.^173^
- A Brazilian cohort study has shown data of 1220 outpatients in a specialized HF clinic followed up for 26±26 months from 1991 to 2000. Patients were in functional classes III and IV, had a mean age of 45.5±11 years, and 78% were men. The main etiologies were dilated cardiomyopathy (37%), ChD (20%), and ischemic cardiomyopathy (17%). During the follow-up period, 415 (34%) patients died, and 71 (6%) patients underwent heart transplantation. Chagas disease was a predictor of poor prognosis.^174^
- More recent data from 700 consecutive patients with HF-REF from the outpatient clinic of a tertiary health center in São Paulo, Brazil, have shown 1-year mortality of 6.8% (47 patients). The composite outcome of death and hospitalization was 17.7% (123 patients), and 7 patients (1%) were submitted to heart transplantation. The patients’ mean age was 55.4±12.2 years, and 67% were men. The main etiologies were hypertensive (26.0%), ischemic (21.9%), and Chagasic (17.0%) forms of cardiomyopathy. High levels of blood urea nitrogen and brain natriuretic peptide, as well as low systolic blood pressure, were independent predictors of 1-year overall mortality in the sample.^175^
- In a study reporting data from a National Database of Multisite Pacemaker, including 3526 patients from 2002 to 2007 cared for at the SUS, the patients’ mean age was 59.8±13.3 years, and 66% were men. The overall survival of patients submitted to cardiac resynchronization therapy in Brazil was 80.1% (95% CI, 79.4–80.8) in 1 year and 55.6% (95% CI, 54.6–56.6) in 5 years, whereas the median overall survival was 30.3 months (IQR, 16.1–50.9). Furthermore, improved survival was observed in the cohort studied, from 2002 throughout 2007 (p=0.055).^176^

### Hospitalizations

- Hospital admissions are main consequences of decompensated HF, resulting in worse prognosis and increasing costs. The BREATHE Study has evaluated a sample of patients admitted due to acute decompensated HF. A total of 1263 patients were included from 51 centers from different Brazilian regions in 2011 and 2012. In-hospital mortality was 12.6%, and care quality indicators based on hospital discharge recommendations were reached in less than 65% of the patients.^177^
- Other studies reporting mortality rates before the BREATHE study^178 - 180^have shown similar rates of in-hospital mortality, ranging from 9% to 17%.^179^
- In a comparison of decompensated HF patients in tertiary care teaching hospitals in Brazil and in the US, US patients were older (P<0.01) and had higher prevalence of the ischemic etiology (P<0.01). Length of stay was significantly shorter (5 [IQR, 3-9] vs. 11 [6-19] days; P<0.001) and in-hospital mortality was lower (2.4% vs. 13%; P<0.001) in the US cohort, but fewer clinical events within 3 months after discharge were observed in the Brazilian patients (42% vs. 54%; p = 0.02). This study highlights the importance of improving knowledge about HF in Brazilian patients to improve care and outcomes.^181^
- In the previously cited systematic review that evaluated the burden of HF in Latin America, 64% including studies from Brazil,^167^the hospital admission rates were 33%, 28%, 31%, and 35% at 3, 6, 12, and 24 to 60 months of follow-up, respectively. The median hospital length of stay was 7.0 [IQR, 5.20-11.00] days. In-hospital mortality was 11.7% (95% CI, 10.4%-13.0%), and the rate was higher in patients with reduced ejection fraction, ischemic heart disease, or ChD. The 1-year mortality rate was 24.5% (95% CI, 19.4-30.0).
- Using data from the SUS, the number of hospitalizations and deaths due to HF were described in São Paulo, Brazil, from 1992 to 2010. The in-hospital mortality rate due to HF was 15%. Comparing between the 1992-1993 and the 2008-2009 periods, there was a 32% decrease in the number of hospitalizations due to HF (p = 0.002), a 15% increase in mortality (p = 0.004), and increased hospital length of stay due to HF (from 8.8 to 11.3 days, p = 0.001).^182^
- In 2019, a more recent study with data from DATASUS evaluated HF admissions over a 10-year period (2007 to 2016) in Brazil, as compared to those in the state of Rio Grande do Sul and in the city of Porto Alegre (a city with several referral centers). As depicted in Chart 4-10, that study showed a decline in in-hospital mortality rates from 2007 to 2016 both in Brazil (19% decline) and in the state of Rio Grande do Sul (25% decline), and a more pronounced decline in the city of Porto Alegre (65%).^183^

### Burden of Disease

- According to the GBD 2017 estimates (Table 4-13), the age-standardized YLD rates for HF were 112 (95% UI, 83-141) in 1990 and 109 (95% UI, 81-134) in 2017, per 100 000 inhabitants, corresponding to a 3% decrease (95% UI, -6.7 to 0.3). These changes are similar to those observed in the HF prevalence rates. Despite this decrease in YLD rates, HF resulted in 88 114 (95% UI, 64 078-112 624) DALYs in Brazil, in 1990, and in 234 169 (95% UI, 174 338-291 188) DALYs, in 2017, due to population growth and aging.
- The age-standardized YLD rates were similar in women and men in 1990, but the 2017 rates were 105 (95% UI, 82-127) for men and 111 (95% UI, 80-141) for women, due to a 6.8% reduction (95% UI, 10.9 to -2.6) for men as compared to almost no reduction for women (-0.3%, 95% CI, -4.9 to 4.2). As expected, the highest YLD rate was observed in the 70+ year group, followed by the 50-69 year group. Similarly to the changes observed in prevalence, from 1990 to 2017, the greatest YLD increases were observed in the 15-49 year group.

## Health Care Utilization and Cost

### (Refer to Tables 1-6 through 1-9 and Charts 1-15 through 1-16)

- According to data from the SUS, there were 2 862 739 hospitalizations due to HF from 2008 to 2018. This number represents one third of total clinical admissions related to cardiovascular conditions in the period studied. Unadjusted costs were R$ 3 597 824 618. In international dollars, adjusted values converted to purchasing power parity for 2018US$ were $ 866 945 691.
- During the period observed, there was a reduction in the number of clinical admissions due to HF from 298 474 (157 per 100 000) in 2008 to 222 394 (107 per 100 000) in 2018, with an even reduction over the years. Despite that reduction in the number of admissions, unadjusted healthcare expenditure estimates from the direct payment for care of HF patients increased from 2008 to 2018 by almost 28%, from R$ 272 280 662 (2018 Int$ 65 609 798), in 2008, to R$ 348 832 330 (2018 Int$ 172 008 052), in 2018. The decreasing number of admissions and increased expenditure represent higher costs per admission throughout the observed period (R$ 912 in 2008 to R$ 1568 in 2018). Heart failure accounted for most costs related to clinical admissions due to cardiovascular diseases.
- The economic burden of HF in Brazil has been assessed using standard cost of illness framework to assess the costs in 2015. The analysis has assessed the prevalence and associated expenditures on healthcare treatment, productivity losses from reduced employment, costs of providing formal and informal care, and lost wellbeing. The study has found that HF imposes a financial cost of R$ 22.1 billion/US$ 6.8 billion, the second of the four major heart conditions in Brazil: myocardial infarction, HF, hypertension, and atrial fibrillation.^184^
- In the study by Nicolao et al.,^183^the DATASUS data of HF admissions over a 10-year period (2007 to 2016) have shown a 97% increase in the mean per-patient cost of HF-related hospitalizations from 2007 to 2016. Data from the city of Porto Alegre (a city with several referral hospitals) have shown an even more pronounced increase (135%), but also a more pronounced decrease in mortality as compared to data of Brazil (see above).

### Open Heart Transplantation and Assist Device Placement

- The number of heart transplantations performed in Brazil increased from 149 in 2006 to 357 in 2016. Although the number of heart transplantations increased significantly in that period, it represents approximately one-fifth of the estimated population need. The 1-year survival was 73% (data for survival collected from 2010).^185^
- An analysis of cost for heart transplantation in Brazil, of all consecutive heart transplant recipients at a single center from July 2015 to June 2017, has shown an average total cost for the 27 patients included of US$ 74 341, which is lower than those reported for developed countries, but exceeds the reimbursement value per patient by 60%.^186^
- In a descriptive study of a public reference hospital in cardiology, located in the city of Fortaleza, Brazil, 16 patients have been submitted to ventricular assist device implantation from 2008 to 2015. The mean age was 40.1 ± 3.4 years, and 87.5% were men. Chagas heart disease was the main etiology (37.5%). All patients experienced complications during the use of the device, bleeding being the most often [11 (68.8%)]. Regarding the clinical outcome, 10 patients (62.5%) underwent cardiac transplantation and 5 patients (31.3%) died.^187^

### Future Research

- Because HF is considered a garbage code when assigned as the underlying cause of death, studies investigating the better method to reclassify and redistribute this cause are needed to reduce bias and promote better data comparability to enhance health policies.
- Brazilian cohort studies for cardiomyopathies are scarce, and some clinical studies in Brazil have reported HF data, but there are few multicenter studies with data from the Brazilian population. It is worth noting the importance of having data for both HF and cardiomyopathy and for both outpatients and hospitalized patients, in addition to fully understand the increasing burden of HF on cardiovascular diseases. More multicenter large-scale studies are needed to better describe the burden, outcomes, and costs of HF in the Brazilian population.
- In addition, studies exploring quality of care and costs in HF would help in the development of health policies to improve awareness, access to life-saving interventions, organ donation, and the better use of resources in this complex and demanding disease.
- Although mortality rates due to ChD decreased substantially in the last decades, ChD remains an important cause of death in Brazil. Indeed, there is evidence that ChD is an underreported cause of death, and, probably, of hospitalization as well. More data on the hospitalization rates and outcomes in ChCM patients are needed.

## 5. VALVULAR HEART DISEASE

### ICD-9 424; ICD-10 I34 to I38

**See Tables 5-1 through 5-4 and Charts 5-1 through 5-10**

**Table:** 

Abbreviations Used in Chapter 5	
AF	Atrial Fibrillation
ARF	Acute Rheumatic Fever
CABG	Coronary Artery Bypass Graft
CAD	Coronary Artery Disease
CI	Confidence Interval
DALY	Disability-Adjusted Life Year
ECG	Electrocardiogram
FU	Federative Unit
GBD	Global Burden of Disease
HIC	High-Income Countries
ICD	International Statistical Classification of Diseases and Related Health Problems
ICD-9	International Statistical Classification of Diseases and Related Health Problems, 9th Revision
ICD-10	International Statistical Classification of Diseases and Related Health Problems, 10th Revision
NRVD	Non-Rheumatic Valvular Disease
OR	Odds Ratio
RHD	Rheumatic Heart Disease
SDI	Sociodemographic Index
SIM	Brazilian Mortality Information System (in Portuguese, *Sistema de Informações sobre Mortalidade* )
SUS	Brazilian Unified Health System (in Portuguese, *Sistema Único de Saúde* )
TAVI	Transcatheter Aortic Valve Implantation
UI	Uncertainty Interval
YLL	Year of Life Lost

## Prevalence

### Rheumatic Heart Disease

- According to the Global Atlas on Cardiovascular Disease Prevention and Control, RHD is estimated to currently affect about 33 million people worldwide, accounting for 1% to 1.5% (319 400 deaths) of all cardiovascular deaths.^1^^89^Until the middle of the 20^th^century, RHD was the leading cause of heart valve disease in the world. Improved health conditions, early identification of Streptococcus pyogenes infections, and antibiotic use have decreased significantly RHD prevalence in HIC. Published data from 2016 have estimated that RHD is the primary cause of 2.5% of valvular heart disease in the United States of America and Canada, reaching up to 22% in Europe.^190^Even higher rates have been reported in Brazil, accounting for around 50% of the heart valve surgeries in the SUS.^191 - 193^
- In low- to middle-income countries, on the other hand, the prevalence of RHD is estimated at 444 per 100 000 inhabitants.^194^In Brazil, RHD persists as the main etiology of heart valve diseases, especially in patients from the SUS. Previous evaluations have shown a prevalence of 3.6 per 1000 inhabitants in Brazil.^195^Other evaluations have found a prevalence ranging from 1 to 7 per 1000 schoolchildren.^196^
- In Brazil, of 174 patients presenting with acute valvular heart disease to the emergency room of the São Paulo Heart Institute, rheumatic involvement was observed in 60%, followed by degenerative aortic valve disease (15%), and mitral valve prolapse (13%). In total, 27.5% of the patients had isolated mitral regurgitation and 11% had mitral stenosis, with aortic valve disease present in the remaining patients.^197^
- A recent study in Brazil has shown that the regression rates of heart valve disease, especially in patients with moderate/severe ARF, may be lower than previously described. Only 22/69 patients had total regression of mitral regurgitation after rheumatic carditis (31.9%). Aortic regurgitation also has a lower total regression rate than that observed in studies prior to the echocardiographic era (18%). However, most cases have persisted with mild mitral or residual aortic regurgitation.^198^In another study involving 258 children and adolescents with ARF followed up from 2 to 15 years, valve lesions healed in 25% of the patients with mild carditis, in 2.5% of those with moderate carditis, while no healing was observed in those with severe carditis.^199^
- According to the GBD 2017, from 1990 to 2017, the age-standardized prevalence of RHD had a slight 3.0% (95% UI, 1.6 - 4.3) increase, from 721.4 (95% UI, 688.7 - 754.5) to 743.2 (95% UI, 709 - 778.6) per 100 000 inhabitants, remaining higher in women than in men throughout the whole period (Table 5-1 and Chart 5-1.A). Although small for both sexes, the percent increase was numerically more pronounced in women (4.4%) than in men. The percent increases were higher in the states of Piauí (Northeastern region), Tocantins and Roraima (Northern region). Although the central estimates were higher in these states, the 95% UIs were wide and overlapped with those of other FUs (Table 5-1).^2^^00^However, it may be hypothesized that the small increase in the RHD prevalence observed in the period may reflect the progress of epidemiological data collection and health statistics.^201^
- The crude prevalence of RHD, however, increased 16.0% (95% UI, 14.4 – 17.5%) from 1990 to 2017, from 690.2 (657.9 – 723.6) to 800.3 (764.1 – 838.2) per 100 000 inhabitants, also remaining higher in women in the period (Chart 5-1.B). Similarly to the trend of age-standardized prevalence rates, the increase in crude prevalence was more pronounced for women than for men.^200^
- The proportional prevalence of RHD in the Brazilian population, thus, showed the same pattern observed for age-standardized and crude prevalence, with a slight increase for both sexes from 1990 to 2017 (Chart 5-1.C).^200^
- Even with the relatively stable trends depicted by the GBD 2017 modeling, RHD is the most prevalent cause of mitral valve disease in Brazil according to published data, when both mitral stenosis (over 90%) and mitral regurgitation (around 55-60%) are considered.^197^
- Mitral stenosis occurs most often in women than in men, in a ratio of 3 to 2. It is a frequent sequela of ARF, affecting more than 85% of the cases even in HIC, such as those in Europe,^202^with a similar pattern still observed in Brazil.^193 , 197^More rarely, mitral stenosis is associated with other diseases, such as mitral annulus calcification, mucopolysaccharidosis, rheumatoid arthritis, and congenital carcinoid syndrome.^190 , 197^
- More recent large-scale screening studies, looking at subclinical RHD, have shown a prevalence of 42 per 1000 in schoolchildren with an average age of 11 years in the Southeastern state of Minas Gerais, being 37 per 1000 for borderline RHD and 5 per 1000 for definite RHD (0.5%). In that study, a higher prevalence was observed in girls (48 per 1000 vs. 35 per 1000) and in children older than 12 years.^191^The same project has concluded that primary care centers are the ideal scenario for RHD screening, considering the higher population participation and involvement rates.^203^

### Non-Rheumatic Valvular Heart Disease

- According to the GBD 2017, the age-standardized prevalence of NRVD remained relatively stable in Brazil from 1990 to 2017, with a borderline 8.2% increase from 216.2 (95% UI, 207.9 - 224.6) per 100 000 in 1990 to 233.9 (224.6 - 243.2) per 100 000 in 2017. The percent change was similar for men and women (8.9% vs. 7.8%) (Chart 5-2.A). Calcific aortic valve disease, conversely, showed an increasing trend (20.4%), from 53.5 (95% UI, 48.1 - 59.9) per 100 000 in 1990 to 64.4 (95% UI, 57.2 - 72.5) per 100 000 in 2017, for both men (18.5%) and women (24.2%). For mitral degenerative valve disease and other NRVDs, the age-standardized prevalence was also stable, with slight 4.0% and 3.8% increases, respectively (Table 5-1).^200^
- In contrast with age-standardized rates, the crude prevalence showed a compelling 89.4% (95% UI, 85.3 – 93.8) increase from 1990 [131.9 (95% UI, 126.7 – 137.5)] to 2017 [249.9 (95% UI, 239.8 – 260.1)] per 100 000 (Table 5-1 and Chart 5-2.B). The increase was homogeneous for men and women, and suggests that prevalence is increasing disproportionally in older ages (Chart 5-2.B).^200^
- Unlike mitral valve disease, aortic valve disease is predominantly degenerative or calcific. Observational studies have shown that aortic stenosis is seen in 4.5% of the population aged over 75 years in HIC, such as the United States of America.^204^According to observational studies^197 , 205^and the GBD 2017 data,^200^in Brazil, as well as in the rest of the world, an increasing trend towards degenerative aortic valve disease has been observed, as compared to other etiologies, such as RHD.
- Thus, the increase in all-age prevalence of NRVD has been mostly driven by calcific aortic valve disease [114.2% (95% UI, 105.5 – 124.3)], especially for older age groups (Table 5-2), but the rates have been also significant for mitral degenerative valve disease, 79.8% (95% UI, 78.3 – 81.1), and other NRVDs, despite the limited data quality for the latter (Table 5-1). The proportional prevalence of NRVD also increased significantly for both sexes, from 1990 to 2017 (Chart 5-2.C).^200^
- Contrary to that observed for the rheumatic etiology, there has been an increase in mitral valve prolapse as the etiology of primary mitral regurgitation in Brazil: although in the overall population it reaches rates around 1% to 2.5%, with good prognosis in most cases, among patients admitted with heart valve disease in a Brazilian emergency department in 2009 (56 ± 17 years, 54% females), 13% had that etiology.^196^Conversely, in a hospital registry of heart surgeries in one of the largest capitals of the country (city of Salvador), from 2002 to 2005, only a small proportion of cases was associated with mitral prolapse,^193^similarly to the results of a study with 78 808 patients using 2 large national databases (the Brazilian Hospital Information System and the SIM) from 2001 to 2007, in which only 0.24% (187) of the cases had reported that underlying cause.^206^However, data may be biased by the absence of coding for etiologies of heart valve disease in the public health system and most of the private health system.

## Incidence

- According to a study based on hospital medical charts in Northeastern Brazil, from 2002 to 2005 (1320 surgeries), the mean annual incidence of cardiac valve surgeries was 4.75 per 100 000 residents and was positively associated with age. The mean annual incidences of RHD and degenerative valvular disease were 2.86 and 0.73 per 100 000 population, respectively.^193^

### Rheumatic Heart Disease

- For RHD, the age-specific incidence followed a bimodal distribution according to the source of surgery reimbursement, increasing almost linearly by 1 case per 100 000 population for each decade of life until the age of 40–49 years, peaking at 4.85 cases per 100 000 population. Following a decline, a second peak occurred at 60–69 years of age (6.54 cases per 100 000 population).^193^The incidence of RHD had a small 1.9% (95% UI, 0.8 – 3.1) increase in Brazil from 21.4 (95% UI, 20.4 – 22.4) per 100 0000 in 1990 to 21.8 (95% UI, 20.8 – 22.8) per 100 000 in 2017, according to GBD 2017 data. This small increase was relatively homogeneous across the country, with overlapping 95% UIs even in the poorer states of the Northern and Northeastern states.^2^^00^
- In general, the increase in incidence was driven by the age groups ‘under 5 years’, 4.3% (95% UI, 2.5 – 6.2), and ‘5-14 years’, 2.9% (95% UI, 1.5 – 4.3), while, in individuals aged 15-49 years, a marked 12.1% (95% UI, -13.7 to -10.5) decrease was observed. Although this pattern is unexpected, because, in general, the incidence in younger ages declines first, followed by young adults, it may be hypothetically associated with the improvement in early diagnosis and incorporation of data on subclinical disease in the GBD modeling.^200^

### Non-Rheumatic Valvular Heart Disease

- In a different pattern as compared to that of RHD, the NRVD incidence had a significant 7.9% (95% UI, 6.7 – 9.3) increase from 224.5 (95% UI, 217.5 – 231.6) per 100 000 in 1990 to 242.4 (95% UI, 233.8 – 250.8) per 100 000 in 2017, according to the GBD 2017 estimates. This increase was mostly driven by the 20.4% (95% UI, 16.0 – 25.4) increase in calcific aortic valve disease, markedly in individuals aged >70 years, 37.2% (95% UI, 30.6 – 44.2).^2^^00^
- However, the increasing incidence of calcific aortic valve disease also in individuals aged 15-49 years, 23.4% (95% UI, 18.1 – 28.7), is atypical considering disease epidemiology, and may be cautiously interpreted as a possible limitation of the GBD modeling,^200^because primary data for this cause are scarce in Brazil.

## Mortality

- Valvular heart disease is one of the leading causes of cardiovascular death in Brazil, particularly in economically underserved regions, and RHD – the most socially driven etiology – ranked as the 8^th^/9^th^cause in past decades.^19^In the most underserved setting, RHD has been playing an important role for decades, with decreasing trends – not always adequately captured by statistical modeling – following socioeconomic improvement.^189 , 194 , 206^

### Rheumatic Heart Disease

- Contrasting with the increasing trend of prevalence, the age-standardized mortality rates attributable to RHD significantly decreased by 50.3% in Brazil, from 2.4 (95% UI, 2.3 – 2.5) to 1.2 (95% UI, 1.1 – 1.2) per 100 000, according to the GBD 2017 study. The percent decrease was similar for men and women (Table 5-3 and Chart 5-3.A). A similar trend was observed for the crude mortality rates (Chart 5-3.B). During the period, the total number of deaths remained stable [2648 (95% UI, 2550 - 2728) and 2682 (95% UI, 2585 – 2797) in 1990 and 2017, respectively], despite population growth (Table 5-3). These trends may reflect improvement in health conditions, and better and earlier access to healthcare.^207 , 208^
- The proportional mortality attributable to RHD in Brazil also showed a decreasing pattern in the same period, with a less steep trend (Chart 5-3.C).^207 , 208^
- In 1990, RHD ranked the 9^th^among the causes of death in Brazil (8^th^to 9^th^in different states), and moved to the 12^th^in 2017 (10^th^to 12^th^in most states, remaining as the 9^th^only in the state of Paraíba).^207 , 208^
- The more compelling decrease in mortality rates was observed in lower ages, especially the ‘under-5’ and ‘5-14 year’ age groups, -82.5% (95% UI, -87.9 to -74.4) and -69.8% (95% UI, -72.4 to -65.6) per 100 000, respectively (Table 5-2).^207 , 208^This may be associated with the better treatment of early and acute presentations, while chronic sequelae still persist as a challenge.
- According to the GBD 2017 data, there was no correlation between age-standardized mortality rates and SDI in 1990 (r^2^=0.275, p=0.59) and in 2017 (r^2^=0.233, p=0.83). However, there was a significant correlation between the percent change in age-standardized mortality rates and SDI in 1990 (Chart 5-4) (r^2^=0.073, P<0.001), which did not reach statistical significance in 2017 (r^2^=0.064, p=0.06). Considering RHD as the most socially-driven etiology of heart valve disease, this mismatch was probably associated with: a) differences in the SDI within the FUs, with discrepant socioeconomic patterns in subregions of the states, which could bias the model; b) the percent change over the decades may represent a more precise measure of the model’s improvement, and the reduction in socioeconomic gap between the Brazilian FUs from 1990 to 2017 may have contributed to reduce the magnitude of the statistical correlation.

### Non-Rheumatic Valvular Heart Disease

- According to the GBD 2017 study, age-standardized mortality rates attributable to NRVD remained relatively stable from 1990 to 2017, with a small 7.1% (95% UI, -9.1 – 13.0) increase (Table 5-3 and Chart 5-5.A). However, for crude mortality rates, the increase was significant, 87.5% (95% UI, 63.5 – 96.9), with a considerable contribution of older ages, markedly over 70 years of age, 45.6% (95% UI, 10.0 – 57.1) (Table 5-3 and Chart 5-5.B). The patterns were similar for men and women. Similar trends were observed for calcific aortic valve disease mortality rates, with a marked 108% increase in the elderly (≥70 years), reflecting the association with population aging (Table 5-2). For mitral degenerative valve disease, the age-standardized mortality rates decreased by 10.8% (95% UI, -47.6 to 2.4), as opposed to a 50.1% increase in the crude prevalence (Tables 5-1 and 5-3), as a result of the increasing rates, 17.7% (95% UI, -42.1 to 39.2), in septuagenarians and older (Table 5-2).^207 , 20^^8^
- The increasing mortality rates in older ages due to NRVD noticeably contrasts with the trends observed for RHD, possibly reflecting a higher prevalence and, consequently, mortality in the age groups >70 years, for both aortic and mitral NRVD (Table 5-2). From 1990 to 2017, an increasing burden of calcific aortic valve disease, in both males and females, associated with an increase in mortality in that age group. The 95% UIs are overall wide for NRVD mortality estimates, especially for each specific disease in separate.^207 , 208^
- The proportional mortality attributable to NRVD in Brazil showed a marked increasing pattern in the same period, possibly driven by the steeper trends of calcific aortic valve disease (Chart 5-5.C).^208^
- In 1990, NRVD ranked as the 10^th^cause of death in Brazil (8^th^to 11^th^in different states), and moved to the 9^th^in 2017 (8^th^to 10^th^in most states, remaining as the 11^th^only in the state of Sergipe) (Chart 5-4).^208^
- The GBD 2017 data demonstrated significant and strong correlations between the SDI and the age-standardized mortality rates of NRVD in general in 1990 (r^2^=0.62, p=0.002) and 2017 (r^2^=0.618, p=0.001), and a similar pattern for calcific aortic valve disease (1990: r^2^=0.65, p=0.002; and 2017: r^2^=0.65, P<0.001) (Chart 5-6). As the socioeconomic development correlates with epidemiological transition and life expectancy, a higher SDI is associated with more elderly individuals at risk of degenerative valvular conditions and less prone to infectious etiologies, such as RHD.
- Additionally, the percent changes in age-standardized mortality rates from 1990 to 2017 also correlated with SDI in 1990 and 2017 for calcific aortic valve disease (1990: r^2^=0.17, p=0.005; and 2017: r^2^= 0.23, p = 0.003), but not for NRVD in general.
- For degenerative mitral valve disease and other NRVDs, no significant correlations were observed between the SDI and the age-standardized mortality rates – or their percent changes over time.

## Burden of Disease

### Rheumatic Heart Disease

- According to the GBD 2017 data, the age-standardized DALY rate attributable to RHD significantly decreased 40% in Brazil, from 118.9 (95% UI, 106.1 – 134.7) per 100 000 in 1990 to 76.5 (95% UI, 63.3 – 93.7) per 100 000 in 2017 (Chart 5-7.A). The decrease rates observed in the period were similar for men and women, 44.4% (95% UI, 31.2 – 42.3) and 37.9% (95% UI, 31.8 – 44.0), respectively (Table 5-4).^209^
- Age-standardized DALY rates decreased in all Brazilian states, with a steeper trend in the regions with the highest rates in 1990: West-Central and Southeast (Table 5-2). The DALY proportion showed a more stable trend for Brazil, with a slight decrease, although an increase in the Northern and Northeastern regions persisted (Chart 5-7.B). The Southeastern and West-Central regions had the highest age-standardized DALY rates and proportional DALY during the whole period analyzed.^209^
- A similar downward pattern was observed for the RHD age-standardized YLL rates, which ranged from 84.9 (95% UI, 81.1 – 87.8) per 100 000 in 1990 to 36.1 (95% UI, 34.8 – 37.8) per 100 000 in 2017, with a 57.4% (95% UI, 54.8 – 59.4) decrease.^200 , 209^
- The GBD 2017 estimates showed a significant correlation between age-standardized DALY rates and SDI in 1990 (r^2^=0.056, p=0.001) and in 2017 (r^2^=0.068, p=0.001). In addition, there was a significant correlation between the percent change in age-standardized DALY rates and SDI in 1990 (r^2^=0.03, P<0.008), although this was not observed in 2017 (r^2^=0.019, p=0.146) (Chart 5-8), suggesting that the less socially developed areas in 1990 – bearing a higher disease burden – had greater room for improvement following social development over the decades.

### Non-Rheumatic Valvular Heart Disease

- According to the GBD 2017, the age-standardized DALY rate attributable to NRVD decreased slightly (8.0%) in Brazil, from 42.8 (95% UI, 36.6 – 45.8) per 100 000 in 1990 to 39.4 (95% UI, 39.4 – 42.2) per 100 000 in 2017 (Table 5-4 and Chart 5-9.A). The discrete decrease in rates observed in the period was similar for men and women. Regarding specific diseases, rates decreased more significantly for mitral degenerative valve disease, -18.7% (95% UI, -9.3 to -43.4), as compared to calcific aortic valve disease and other valve diseases, although the UIs were wide in this case. The trends observed for YLLs were similar.^200 , 209^
- The relatively stable trend was similar in all Brazilian states, and the age-standardized DALY rates remained higher for the Southern and Southeastern regions during the whole period, followed by the West-Central, Northern and Northeastern regions (Chart 5-9.A).
- Similarly to that observed for mortality, the stable age-standardized DALY rates contrast with the increase of crude rates [38.7% (95% UI, 27.5 – 47.2)] in the period, again suggesting that morbidity associated with NRVD is shifting to the elderly, presumably following changes in the population age composition.^209 , 210^
- The proportional DALY rates in Brazil increased, and, from 1990 to 2017, the Southern and Southeastern regions accounted for the highest DALY proportions in the period, according to the GBD estimates (Chart 5-9.B).^209 , 210^
- Still according to the GBD 2017 data, there were significant correlations between the age-standardized DALY rates of NRVD in general and SDI in 1990 (r^2^=0.65, p=0.043), but not in 2017 (r^2^=0.49, p=0.067). Percent changes in age-standardized DALY rates (1990 – 2017) correlated with SDI in 1990 (r^2^=0.32, p=0.003) and 2017 (r^2^=0.33, p=0.002). For calcific aortic valve disease, significant correlations were observed between DALY and SDI in 1990 (r^2^=0.67, p=0.002) and 2017 (r^2^=0.57, p=0.004) (Chart 5-10), as well as between percent changes in DALY rates and SDI in both years, suggesting the effect of socioeconomic development also as a determinant of some types of NRVD.
- For degenerative mitral valve disease and other NRVDs, however, no correlations were observed between age-standardized DALY rates – and their percent changes over time – and SDI, except for the weak association between DALY rates and SDI in 2017 (r^2^=0.08, p=0.02), based on the GBD estimates.

## Complications and Associated Diseases

### Arrhythmias associated with Valve Disease

- Atrial fibrillation is also a complicating factor for patients with valvular heart disease, usually occurring in those with more advanced natural history. It is more commonly associated with mitral valve disease, especially mitral stenosis. Atrial fibrillation was observed in 34% of a cohort of 427 patients (mean age 50±16 years, 84% females) with severe mitral stenosis, being more frequent in patients who died during follow-up (27/41, 66%) as compared to survivors (114/378, 30%), reinforcing its role as a prognosis marker in heart valve disease.^211^
- Atrial fibrillation can also develop in severe aortic valve disease, especially in older and postoperative patients. In a retrospective cohort of 348 patients, with a mean age of 76.8±4.6 years, postoperative AF was observed in 32.8% (n = 114), but rates were higher in patients aged 80 years and older (42.9% vs. 28.8% in patients aged 70-79 years, p=0.017).^212^
- In another retrospective assessment conducted in the state of Pernambuco (Northeastern Brazil), involving 491 consecutive patients after surgery for valvular heart disease, the incidence of AF was 31.2% and associated with age >70 years (OR=6.82; 95% CI, 3.34 - 14.10, p<0.001), mitral valve disease (OR=3.18; 95% CI, 1.83 - 5.20, P<0.001), and no perioperative use of beta-blockers, among other factors.^213^
- Valvular heart disease (17.5%) and arrhythmias (AF and atrial flutter - 50.7%) were the main cardioembolic source of stroke in a study involving 256 patients (60.2 ± 6.9 years, 132 males) in the Southern region of Brazil.^214^

### Association between Valvular Heart Disease and Coronary Artery Disease

- Due to the increased surgical risk of combined valve procedures and coronary revascularization, it is essential to recognize the prevalence of obstructive CAD in association with valvular heart disease. Studies have shown a lower prevalence of CAD in patients with RHD as compared to those with NRVD, possibly as a reflection of the lower median age of RHD patients and the higher prevalence of coronary risk factors in individuals with NRVD.^215^
- In a study in Rio de Janeiro (Southeastern Brazil) including 1412 candidates for cardiac surgery of any indication, 294 cases with primary valvular heart disease of rheumatic and non-rheumatic etiologies were selected. All patients were aged ≥40 years and had coronary angiography performed. The prevalence of CAD in RHD and NRVD patients was 4% and 33.6% (p <0.0001), respectively. Characteristics and risk factors, such as age, typical chest pain, hypertension, diabetes mellitus, and dyslipidemia, were significantly associated with obstructive CAD.^216^
- In another study in Brazil evaluating 712 patients with valvular heart disease, mean age of 58±13 years, the incidence of obstructive CAD was 20%. However, in younger patients (<50 years), the prevalence was much lower (3.3%).^217^These data are similar to those observed in another study that included 3736 patients (mean age of 43.7 years), in which the prevalence of obstructive CAD combined with valvular heart disease was 3.42%.^215^

## Healthcare Utilization and Cost

### (Refer to Tables 1-6 through 1-9 and Charts 1-15 through 1-16)

- According to the SUS administrative database, the total crude expenses (reimbursement) with hospital admissions for the clinical treatment of valvular heart disease in Brazil showed a significant 94% increase, from R$ 1 051 959 34, in 2008, to R$ 2 043 358, in 2018, in an almost-linear pattern. Adjusting and converting these values to international dollars in 2018, the total costs for the public health system were $ 1 014 294, in 2008, and $ 1 007 587, in 2018, for hospitalization due to valvar conditions.
- Similarly, unadjusted costs associated with valvular surgical/interventional procedures (codes related to valve surgery, percutaneous mitral commissurotomy, other types of valvuloplasty) also increased from 2008 to 2018 (with a decrease after correction for international dollar), from R$ 130 588 598 (2018 Int$ 125 912 942) to R$ 180 735 108 (2018 Int$ 89 119 874), although with less magnitude as compared to clinical admissions (94% vs. 38%).^201^
- The number of surgical/interventional admissions related to valve diseases did not grow much in Brazil from 2008 to 2018, ranging from 13 129 in 2008 to 14 294 in 2018. This is presumably associated with the growing complexity and costs of interventions (markedly, devices and prostheses) and denotes the economic burden posed by the incorporation of new procedures and technologies, but is also a marked effect of inflation on healthcare costs, considering values adjusted to Int$. In this scenario, the future incorporation of well-established therapies not yet reimbursed by the SUS, such as TAVI, will contribute to increase the economic burden, although expenses with judicial demands may overcome ordinary costs.^218^
- The total number of admissions in this period was 172 126, and most of them occurred in the Southeastern region (41.2%), followed by the Northeastern (25.7%), Southern (20.2%), West-Central (7.5%), and Northern (5.4%) regions.^201^
- A dramatic drop was observed for some types of procedures, despite their growing indications, such as the percutaneous mitral commissurotomy. For this specific procedure, the downward numbers may be associated with the lagged reimbursement tables of SUS, limiting the number of hospitals that perform this intervention. The absolute number of open valve surgeries remained stable in the period, from 12 201 (2008) to 12 088 (2018), despite the growing number of cases of valvular heart disease – especially NRVD – and the growing burden in the elderly.^200 , 201^
- In none of the periods, the increase in the number of admissions paralleled the increasing expenses, suggesting a progressive complexity – and, consequently, cost – of the procedures to treat heart valve disease.^201^
- From the SUS administrative database, valve procedures associated with RHD sequelae cannot be differentiated from those associated with other etiologies, since no specific coding is available, and the reporting of ICD coding is imprecise.^201^
- Interestingly, observational studies have reinforced that RHD remain as the main etiology associated with cardiac surgery in young people in Brazil, reaching up to 60% in a study performed in the city of Salvador, Bahia (Northeastern Brazil).^193^Moreover, at the São Paulo Heart Institute (Southeastern region), the number of heart valve surgeries associated with RHD has increased substantially over the past 10 years, from around 400 surgeries/year in 1990 to almost 600 after 2000.^219^Between 2008 and 2015, there were 26 054 hospital admissions due to ARF sequelae, 45% of which due to heart disease, leading to a possibly underestimated total cost of US$ 3.5 million annually.^192 , 201^
- Overall, valve diseases of rheumatic origin account for about 90% of the cardiac surgeries in children and over 30% of the cardiac surgeries in adults, most of them in young ages,^22^^0^also according to observational studies and hospital-based registries. However, few epidemiological studies have estimated the cause-specific burden of valve diseases in Brazil.

### Mitral Valve Disease

- Based on SUS administrative data from 2001 to 2007 and regarding mitral valve surgery, in a retrospective series of 78 808 consecutive surgical patients, the mean age was 50.0 years (35.9 - 62.5) and 40 106 were females (50.9%). Again, RHD was the main etiology, accounting for 53.7% of the total patients undergoing surgery, and over 94% of the patients undergoing procedures due to mitral stenosis. Mitral stenosis was the largest single surgical indication, accounting for 38.9% of the total. Overall, valve replacement was done in 69.1% of the surgeries. In-hospital mortality was 7.6%.^206^
- Surgical mortality was slightly higher in women than in men (7.8% vs. 7.3%; p<0.001), and considerably higher in people aged ≥80 years. On the other hand, the lowest mortality was observed for those between 20 and 39.9 years (p<0.001). Patients with combined aortic and mitral surgeries (reflecting rheumatic etiology) were the youngest (median, 43.3 years). Surgery for aortic stenosis was more common in older individuals (median, 58.0 years) (p<0.001). Valve repair had lower mortality (3.5%) as compared to valve replacement (6.9%), multiple valve repair and/or replacement (8.2%), and concomitant CABG (14.6%) (P<0.001). Associated CABG occurred in 7147 patients (9.1% of the sample).^206^
- Regarding percutaneous commissurotomy, studies in Brazil have shown a much higher proportion of females (85%) – coincident with the epidemiology of RHD and noticeably mitral stenosis – and of young people (<40 years).^221 , 222^

### Aortic Valve Disease

- A cohort of 724 consecutive patients, who underwent cardiac surgery at the São Paulo Heart Institute, has evidenced, similarly to other studies, a higher rate of women (55%) and predominance of RHD (60%). However, in that series, there was a great proportion of aortic valve disease (396 cases) as compared to mitral valve disease (306 cases) and other series. Of the patients with mitral valve disease, 39.9% had stenosis, 38.4% regurgitation, and 21.7% mitral prosthesis dysfunction. In patients undergoing aortic valve interventions, stenosis was observed in 51.6%, regurgitation in 29.3%, and prosthesis dysfunction in 19.1%. The study suggests an increase in aortic valve disease as compared to mitral valve disease in a tertiary hospital in the Southeastern region of Brazil.^223^
- Another retrospective cohort study has been conducted in the city of Porto Alegre (Southern Brazil), with 1065 patients (mean age, 61.4 ± 11.8 years; 38% women). Aortic valve replacement was done in 18.8% and mitral valve replacement in 13.4%. Concomitant coronary revascularization was performed in 60.3% of the sample, and valve surgeries in 32.7%. Overall in-hospital mortality was 7.8%, being lower for isolated CABG (5.9%), intermediate for valve surgery (aortic and/or mitral and/or tricuspid = 8.6%), and higher for combined valvular and CABG procedures (20.0%).^224^

### Transcatheter Aortic Valve Implantation in Brazil

- As in other countries, TAVI gained importance in Brazil in the past 20 years. It is estimated that over 100 000 percutaneous aortic valve implantations have been performed worldwide to date.^205 ,2018^The first TAVI implantation in Brazil occurred in 2008. The Brazilian TAVI registry has reported 418 TAVI performed in 18 centers until 2014, and this number has grown exponentially since then. Femoral access was the choice in 96.2% of the procedures, and the prostheses used were CoreValve^®^(86.1%) and Sapien XT^®^(13.9%). All-cause mortality of this initial experience at 30 days and 1 year was 9.1% and 21.5%, respectively.^225^
- Data from the TAVI registry updated in 2017 revealed a total of 819 patients under clinical follow-up, demonstrating that the procedure has a low incidence of complications – especially early hard clinical outcomes – and highlighting rates of postprocedural renal failure around 18%.^226 , 227^
- In another assessment performed in the city of Rio de Janeiro, of 136 patients undergoing TAVI [median age, 83 (80-87) years; 51% males], perioperative mortality was 1.5%; 30-day mortality, 5.9%; in-hospital mortality, 8.1%; and 1-year mortality, 15.5%.^228^
- Of 819 percutaneous aortic valves implanted until 2017, 135 patients (20.1%) required permanent pacemaker implantation. These patients were older (82.5 vs. 81.1 years; p=0.047), predominantly men (59.3% vs 45%; p=0.003), and had previous right bundle-branch block (OR=6.19; 95% CI, 3.56 - 10.75, p≤0.001). The use of CoreValve^®^prosthesis (OR=3.16; 95% CI, 1.74 - 5.72, p≤0.001) and baseline transaortic gradient >50 mm Hg (OR=1.86; 95% CI, 1.08 - 3.20, p=0.025) were independent predictors of permanent pacemaker implantation.^227^

## Future Research

- Despite the noticeable improvement in past decades, there is still paucity of primary data about the epidemiology of valvular heart disease in Brazil, and plenty of room for future research. There are some administrative challenges for data collection and the development of nationwide registries associated with coding of hospital admissions and procedures. Especially in the SUS, the current codes do not allow discrimination of crucial variables, such as the valve involved, type of valvular dysfunction, type of prosthesis, and, especially, etiology and association with systemic diseases. Thus, refining the coding system or implementing mandatory clinical and surgical reports – as previously done for percutaneous coronary interventions – may be an initial step to improve accuracy in data acquisition.
- As the country has some significant cohorts of patients with valvular heart disease, mid- and long-term follow-up of these samples should be warranted. Of note, there are research initiatives that require incentives and funding for their continuation, such as ongoing studies on long-term prognosis of subclinical RHD in children and adolescents,^191 , 203^genetic and immune determinants of response to streptococcal infections leading to RHD,^229^clinical and procedural predictors of short- and long-term events after percutaneous mitral commissurotomy,^211 , 230^and a national TAVI registry.^225^
- Regarding echo screening for RHD, primary data suggest that the strategy seems to be cost-effective in Brazil,^231^but its application outside research and integration into health systems need continuing investigative efforts – as for other countries. An echocardiographic risk score to predict RHD progression has been derived from a Brazilian cohort,^232^but its broader validation in other settings is still pending. In addition, continuing efforts have been directed to the development of vaccines for streptococcal infections,^229^and collaborative studies on their efficacy and clinical application to reduce RHD burden should be warranted. In the opposite spectrum of valvular heart disease, the incorporation of TAVI in the Brazilian SUS seems to be close,^232^and the evaluation of its actual clinical, budgetary and social impact on public healthcare outcomes will require extensive research.
- Finally, other promising strategies to provide early diagnosis and prioritization of referrals in low-resourced areas should be further investigated in Brazil. As an example, the availability of imaging modalities for the management of valvular heart disease – markedly echocardiography – is limited and unequally distributed in the country. In this scenario, the implementation of tele-echocardiography, with task-shifting of imaging acquisition to non-physicians (still not allowed by Brazilian healthcare regulations outside research) and remote reading, has been evaluated.^233^Despite its good overall diagnostic performance and discrimination of patients at higher cardiovascular risk,^234^the impact on clinical outcomes and the feasibility and cost-effectiveness of the strategy are yet to be explored. Similarly to that observed for other novel modalities such as tele-ECG, AF screening,^235^and remote consultations, the incorporation of imaging innovations to improve access to cardiovascular care in Brazil may require extensive discussions, based on robust scientific evidence.

## 6. ATRIAL FIBRILLATION AND ATRIAL FLUTTER

### ICD-10 I48

**See Tables 6-1 through 6-7 and Charts 6-1 through 6-3**

**Table:** 

Abbreviations Used in Chapter 6	
AF	Atrial Fibrillation
BNP	B-type Natriuretic Peptide
CABG	Coronary Artery Bypass Grafting
ChD	Chagas Disease
CI	Confidence Interval
DALY	Disability-Adjusted Life Year
ECG	Electrocardiogram
ELSA-Brasil	The Brazilian Longitudinal Study of Adult Health
FU	Federative Unit
GARFIELD-AF	The Global Anticoagulant Registry in the FIELD-AF
GBD	Global Burden of Disease
HF	Heart Failure
HR	Hazard Ratio
ICD-10	International Statistical Classification of Diseases and Related Health Problems, 10th Revision
ICU	Intensive Care Unit
IMPACT-AF	A Multifaceted Intervention to Improve Treatment with Oral Anticoagulants in Atrial Fibrillation
INR	International Normalized Ratio
NOAC	New Oral Anticoagulant
OR	Odds Ratio
PPP	Purchasing Power Parity
SD	Standard Deviation
SDI	Sociodemographic Index
SUS	Brazilian Unified Health System (in Portuguese, Sistema Único de Saúde)
TIA	Transient Ischemic Attack
TTR	Time in Therapeutic Range
UI	Uncertainty Interval
VKA	Vitamin K Antagonist

## Prevalence and Incidence

- According to the GBD Study 2017 estimates, the age-standardized prevalence rate of AF and flutter had a small increase in Brazil: from 619 (95% UI, 516-728) in 1990 to 641 (95% UI, 537-751) in 2017 per 100 000 inhabitants, for both sexes, with a 3.5% (95% UI, 1.7-5.3) change in that period. The prevalence of AF and flutter was higher in men [in 1990: 759 (95% UI, 630-893); in 2017: 787 (95% UI, 657-925)] than in women [in 1990: 499 (95% UI, 418-587); in 2017: 522 (95% UI, 440-610)], although the percent change was greater for women (4.6%; 95% UI, 2.3-7) than for men (3.7%; 95% UI, 1.4-5.9) in the period. In absolute numbers, estimates for the prevalence of AF and flutter in Brazil rose from 0.5 million in 1990 to 1.4 million in 2017, mainly due to population growth and aging (Table 6-1 and Chart 6-1). In 2017, the proportion of patients with prevalent AF was 0.67% (95% UI, 0.56-0.78).
- According to the GBD Study 2017, the prevalence rate of AF and flutter is different among the Brazilian FUs, being highest in the FUs of the Southeastern region in 1990 and 2017 (states of São Paulo, Rio de Janeiro and Minas Gerais), and lowest in the FUs of the Northeastern or Northern regions (states of Maranhão, Pernambuco and Alagoas in 1990, and Maranhão, Pernambuco and Pará in 2017). That might be due to a delayed epidemiologic transition and a lower rate of diagnosis in the lowest-income FUs. Men have consistently higher prevalence rates in both time periods across the FUs (Chart 6-1).
- According to the GBD Study 2017 estimates, the age-standardized incidence rates per 100 000 per-year were 44 (95% UI, 36-54) in 1990 and 46 (95% UI, 38-55) in 2017. Those rates were also higher for men in both time periods [women, 1990: 38 (95% UI, 31-45), 2017: 39 (95% UI, 32-47); men, 1990: 53 (95% UI, 43-64), 2017: 54 (95% UI, 44-65)].
- Data from the ELSA-Brasil, a six-center cohort study of civil servants in Brazil that included 14 424 adults with valid ECG (45.8% men, age range 35 to 74 years), have shown a prevalence of 0.3% (48 cases) of AF and atrial flutter (men, 0.5%; women, 0.2%) in the baseline ECG. The odds of presenting AF or atrial flutter increased with age, for both sexes, with the highest prevalence in the oldest age strata, between 65 and 74 years (women: OR: 17; 95% CI, 2.1-135.9; men: OR: 52.3; 95% CI, 3.1-881.8). There was no difference in prevalence according to self-reported race, for both sexes (men: Black 0.3%, Mixed 0.4%, White 0.6%; p=0.42; and women: Black 0.3%, Mixed 0.2%, White 0.2%; p=0.84).^237^
- The GARFIELD-AF Registry is a worldwide study that included patients (≥18 years) with AF diagnosed within the previous 6 weeks and had at least 1 additional risk factor for stroke as evaluated by the study investigator. In Brazil, 41 sites (82.3% cardiologists) included 1065 patients with non-valvular AF between 2010 and 2014 (mean age [SD]: 68 [13] years, 55% males). The prevalence of the AF types was described as follows: new-onset AF in 52% of the patients; paroxysmal AF in 25%; persistent AF in 14%; and permanent AF in 8%.^238^The high prevalence of new-onset AF might be attributed to the inclusion criterion: AF diagnosed within the previous 6 weeks.
- Another cross-sectional population-based study in a deprived neighborhood of São Paulo, including 1524 individuals aged 65 years or older, has found AF in 2.4% of the participants (men, 3.9%; women, 2.0%).^239^
- Telehealth systems in Brazil have provided vast epidemiological data on arrhythmias, including patients seen in primary care facilities and emergency departments. Among 262 685 patients in primary care centers, who underwent ECGs using telehealth in the state of Minas Gerais in 2011, the prevalence of AF was 1.8%, increased sharply with age (OR: 1.08; 95% CI, 1.07-1.08), and was higher in men (2.4%) in all age groups, ranging from 0.2% (20-29 years of age) to 14.6% in nonagenarians as compared to women (1.3%), ranging from 0.1% (20-29 years of age) to 8.7% in nonagenarians (OR: 1.77; 95% CI, 1.68-1.89).^240 , 241^
- A total of 676 621 ECGs from patients (mean age 51±19 years, 57.5% women) routinely visiting 125 primary care centers from January 2009 through April 2016 were analyzed by the telemedicine service of the Federal University of São Paulo. The 7-year prevalence of AF was 2.2% (n = 14 968). The 2025 prevalence of AF in Brazil projected by that study is 1.7%.^246^
- Of 260 879 ECGs performed between March and September 2015 at primary care centers using a telehealth system in the state of Minas Gerais, 304 (0.1%) were stratified as a cardiovascular emergency. Atrial fibrillation and flutter with low- or high-ventricular response were the cause of 22% of those emergencies.^243^
- The prevalence of AF in 1518 individuals (mean age 58±16 years, 66% female), who were in a waiting list for echocardiogram in primary care and were screened for AF with a portable device, was 6.4%. Older age was a risk factor (9.3% vs. 4.8% in those aged over and under 65 years, respectively, P=0.001), and AF was associated with heart disease on echocardiogram (OR: 3.9; 95% CI, 2.1 - 7.2, p<0.001). The authors suggest that AF screening could be a useful primary care tool to stratify risk and prioritize echocardiogram.^244^

## Mortality

- According to the GBD Study 2017, the number of AF deaths in Brazil have increased over the past few years, due to population growth and aging. In the 1990s, AF was responsible for 2649 (95% UI, 2428-2836) deaths, which rose to 10 059 (95% UI, 9390-10 698) in 2017. However, the age-standardized mortality rate due to AF remained stable, with a rate of 4.7 (95% UI, 4.4-5.1) deaths per 100 000 inhabitants in 1990, and of 4.8 (95% UI, 4.5-5.2) per 100 000 inhabitants, in 2017, accounting for 0.7% of all deaths in the country. Although age-standardized prevalence rates were consistently higher in men, women had higher age-standardized mortality rates in 2017 (women: 4.9, 95% UI, 4.8-5.3; men: 4.6, 95% UI, 3.9-5.2), consistent with data from other countries.^245 , 246^When considering individuals aged > 70 years, the mortality rate increases from 1990 (52, 95% UI, 48-55) to 2017 (74, 95% UI, 69-79). Of note, because mortality based on vital registration data alone provides an implausibly steep increase over time, possibly due to changes in ascertainment rather than the epidemiology of AF, the GBD Study 2017 assumes, *a priori* , that age and sex-specific mortality rates are not increasing or decreasing over time.^247^As such, the small changes over time herein reported are intentionally lower than the real changes in raw data.
- Table 6-2 demonstrates the total number of deaths, the age-standardized mortality rate due to AF (per 100 000 inhabitants, both sexes), and percent change, by FU, in Brazil, in 1990-2017. The FUs with the highest percentages of reduction observed between 1990 and 2017 were the states of Espírito Santo, Roraima, Goiás, and Minas Gerais, in that order. On the other hand, the FUs with the highest percentages of increase observed between 1990 to 2017 were the Distrito Federal, and the states of Alagoas and Sergipe. However, these data should be interpreted with caution because they can be inconsistent due to reporting issues. Tables 6-3 and 6-4 reveal data stratified by sex.
- Regarding AF mortality rates according to age groups, the most significant increase was observed in the eldest: 70+ year group (51.6, 95% UI, 47.7-55.3 per 100 000 in 1990; and 74, 95% UI, 69.3-78.7 per 100 000 in 2017) with a percent change of 43.4 (95% UI, 35.9-50.6), followed by the 50-69 year group (2.6, 95% UI, 2.3-2.8 per 100 000 in 1990; and 2.8, 95% UI, 2.5-3.0 per 100 000 in 2017) with a percent change of 6.0 (95% UI, 0.3 - 10.9).
- The GBD Study 2017 uses the SDI as an estimate of the socioeconomic level of a location. As demonstrated in Chart 6-2, there is no statistically significant correlation between the age-standardized mortality rate due to AF and flutter per 100 000 inhabitants and the SDI of the Brazilian FUs (p=0.37), possibly because the prevalence is lower in the lowest income FUs, as mentioned above.
- In a prospective analysis of the Bambuí Cohort Study, – which included all dwellers aged ≥ 60 years on January 1, 1997, in Bambuí, Brazil, located in an endemic area for ChD – a total of 1462 participants with (38%) and without ChD, mean age of 69 (63-74) years, 61% women, were followed up for 10 years. Death occurred in 556 participants. The presence of AF or flutter at baseline was independently associated with an increase in all-cause mortality (HR: 2.35; 95% CI, 1.53-3.62) among patients with ChD and among those without ChD (HR: 1.92; 95% CI, 1.05-3.51).^248^
- A study assessing 302 patients [mean age (SD), 58 (15) years; 53% women] with both valvular (32%) and non-valvular AF, followed up for 1 year, has shown a mortality rate of 10% and no difference between valvular and non-valvular AF. The causes of death were HF in 25 patients (83%), sudden cardiac death in 3 (10%), and thrombosis in a mechanical valve prosthesis in 2 (7%).^249^

## Burden of Disease

- According to the GBD 2017 estimates, AF resulted in 226 810 (95% UI, 187 976-272 166) DALYs in Brazil in 2017, which represent 0.37% of all DALYs. The age-standardized DALY rate was 104 (95% UI, 86-124) per 100 000 in 2017, greater for men (115; 95% UI, 92-141) than for women (93; 95% UI, 79-109), although the proportion of DALYs is higher for women (0.43%; 95% UI, 0.39-0.48) as compared to that for men (0.33%; 95% UI, 0.28-0.39). Comparing to 1990, there was a small increase in DALY rates in the country by 4.6% (UI 95%, 1.7-7.2). The number of DALYs and DALY rates due to AF and flutter in 1990 and 2017, according to the Brazilian FUs, for both sexes, are reported in Tables 6-5 to 6-7.
- No association between age-standardized DALY rates due to AF and SDI was found in Brazil, similarly to that reported for the age-standardized mortality rates (Chart 6-3).

## Health Care Utilization and Cost

### (Refer to Tables 1-6 through 1-9 and Charts 1-15 through 1-16)

- From 2008 to 2018, there were 321 866 hospitalizations for AF, and 1250 ablation procedures for AF and flutter were performed by the SUS, with unadjusted costs of R$ 231 850 160 and 6 950 612, respectively. After adjusting for Brazilian inflation, the costs were R$ 418 504 911 and R$ 12 546 315, respectively. In international dollars converted to PPP-adjusted to US$ 2018, $ 403 520 568 and $ 6 701 749, respectively.
- An analysis of the economic burden of heart conditions in Brazil estimated an AF prevalence of 0.8% (1 202 151 cases) in 2015. The authors estimated a total cost for AF of R$ 3921 million (US$ 1.2 billion).^250^

## Complications

### Stroke

- A stroke registry in the city of Joinville has described all 429 cases of stroke that occurred in 2015. Of those, 87.2% (374/429) were ischemic strokes. Atrial fibrillation was detected in 11.4% (49/429) of the patients and in 58% (49/84) of all cardioembolic strokes.^251^Similarly, AF has been detected in 58% of 359 patients with cardioembolic stroke in a one-center, consecutive sample in the city of Curitiba, Brazil.^252^
- In a retrospective analysis of 215 patients hospitalized for stroke, AF occurred in 16.3%, and there was an association of AF with increasing age: while in patients aged <65 years, the AF prevalence was 5%, in those aged > 80 years, it increased to 26% (p=0.01).^253^
- Among patients hospitalized for acute ischemic stroke or TIA, a score system for AF diagnosis, based on clinical and echocardiographic variables, has been developed and validated in a Brazilian cohort. Age (OR: 1.04; 95% CI, 1.02-1.08), National Institutes of Health Stroke Scores at admission (OR: 1.10; 95% CI, 1.05-1.16), and the presence of left atrial enlargement (OR: 2.5; 95% CI, 1.01-6.29) were predictors of AF and TIA (C-statistic = 0.76; 95% CI, 0.69-0.83).^254^

### Heart Failure

- In a retrospective study of 659 patients with consecutive hospitalizations due to decompensated HF in 2011, the prevalence of AF was 40% (73% permanent AF). Atrial fibrillation was associated with increasing age (P<0.0001), non-ischemic etiology (p = 0.02), right ventricular dysfunction (p = 0.03), lower systolic blood pressure (p = 0.02), higher ejection fraction (P<0.0001), and enlarged left atrium (P<0.0001). Patients with AF had higher in-hospital mortality (11.0% versus 8.1%, p = 0.21) and longer hospital length of stay (20.5 ± 16 days versus 16.3 ± 12, p = 0.001).^255^

### Falls

- Data from a retrospective study including 107 patients with AF and mean age of 78 years have revealed that 51% had reported at least one fall in the previous year. The risk was higher among patients with diabetes and using amiodarone.^256^

### Dementia

- In a cross-sectional study with 1524 participants aged > 65 years, dementia was diagnosed in 11% of those with AF versus 4% of those without AF (p=0.07). The authors have found an OR of dementia of 2.8 (95% CI, 1.0-8.1; p = 0.06) among subjects with AF.^257^

### Atrial fibrillation type and complications

- In a retrospective study, data from 407 patients treated for AF in a cardiology emergency department during the first trimester of 2012 have revealed that the prevalence of HF and stroke was higher in patients with persistent AF (n=188) and flutter (n=51) as compared to patients with paroxysmal AF (n=168) (HF: 51.2% vs. 45.1% vs. 19.7%; P<0.01; and stroke: 10.7% vs. 9.8% vs. 1.6%; P<0.01).^2^^58^

## Influence of the Risk Factors on Atrial Fibrillation and Flutter

- The GARFIELD-AF Registry, a non-interventional, observational, worldwide study, has included 1065 patients (≥ 18 years) with non-valvular AF diagnosed within the previous 6 weeks at 41 sites in Brazil (82.3% cardiologists). The mean (SD) age was 68 (13) years, and 55% were males. Current/previous smoking was found in 32%, hypercholesterolemia in 42%, obesity in 29%, diabetes in 25%, and hypertension in 81% of the patients.^2^^38^
- Among 262 685 patients in primary care centers who underwent ECGs using telehealth in the state of Minas Gerais in 2011, the self-reported prevalence of risk factors among AF patients was: hypertension, 51.8%; diabetes, 7.3%; smoking, 6.7%; dyslipidemia, 3.5%. Only hypertension had a significant association with AF in comparison with non-AF subjects (51.8% vs 31.7%; OR adjusted for age and sex: 1.32; 95% CI, 1.24–1.40).
- A cross-sectional study comparing AF subjects with healthy controls has found a higher frequency of sleep apnea in the AF group as compared to the control group (81.6% versus 60%, p = 0.03).^2^^59^

## Associated Diseases and Comorbidities

- The incidence of AF among 300 elderly patients (mean age, 75±8 years; 56% women) monitored with pacemakers, free of AF at baseline, was 22% in a 435-day follow-up,^2^^60^and reached 85% in patients with pacemakers and chronic kidney disease in a 1-year follow-up.^261^
- In patients with cardiovascular disease who visit the emergency department, the prevalence of AF is 40% in those with decompensated HF^255^and 44% in those with valvular heart disease.^262^
- A study including patients from an ICU has found an AF incidence of 11% during the ICU length of stay.^263^

### Perioperative atrial fibrillation and cardiovascular surgery

- In the postoperative period of cardiac surgery, AF has occurred in 12% to 33% of the patients.^264 -^^266^Valve replacement surgeries were associated with a higher occurrence of AF (31%-33%) during hospitalization as compared to CABG (12%-16%).
- Advanced age, mitral valve disease, and no beta-blocker use were associated with postoperative AF in valvular surgery.^267^Among those who underwent CABG, the postoperative AF incidence was associated with a left atrium > 40.5 mm and age > 64.5 years.^268^

### Atrial fibrillation and Chagas disease

- Case reports have described AF in patients with orally acquired acute ChD,^269^probably related to acute Chagas myocarditis.
- In the Bambuí Cohort Study, 1462 participants aged 60 years and over (mean age, 69 years; ChD n=557, 38.1%) with baseline ECG were followed up for 10 years; the endpoint was mortality. Atrial fibrillation was more frequently observed in ChD subjects: 6.1% vs 3.4% (OR: 3.43; 95% CI, 1.87-6.32, adjusted for age, sex, and clinical variables), and was an independent risk factor for death (HR: 2.35; 95% CI, 1.53-3.62, adjusted for age, sex, clinical variables, and BNP levels) in ChD subjects.^248^
- In a large sample of 264 324 patients who underwent tele-ECG in a primary healthcare unit in 2011, ChD was self-reported by 7590 (2.9%). The mean age of ChD subjects was 57.0 (SD: 13.7) years, and that of non-ChD subjects was 50.4 (SD: 19.1) years, with 5% of octogenarians in both groups. Atrial fibrillation was observed in 5.35% of the ChD subjects and in 1.65% of non-ChD ones (OR: 3.15; 95% CI, 2.83-3.51, adjusted for age, sex, and self-reported co-morbidities). In octogenarians, the prevalence of AF reached 16.26% of the patients: 15.47% of women and 17.95% of men.^270^
- In the baseline of the NIH-SaMi-Trop Cohort Study, which recruited ChD patients (n=1959, 67.5% females, median age 59, Q1-Q3: 49-69 years) with suspected cardiac disease in 21 municipalities of the Northern Minas Gerais, the prevalence of AF was 4.5%, being higher in men than in women (5.6 vs 2.6%, P<0.001) and in those with high versus normal NT-ProBNP levels (14.6% vs 2.1%, P<0.001).^271^
- In a systematic review and meta-analysis, Rojas et al. have evaluated the frequency of electrocardiographic abnormalities in ChD in the general population. Forty-nine studies were selected, including 34 023 (12 276 ChD and 21 747 non-ChD) patients. The AF prevalence was significantly higher in ChD patients (OR: 2.11; 95% CI, 1.40-3.19).^272^
- In a sample of 424 ChD patients under the age of 70 years (41.7% females; mean age, 47 [SD:11]), followed up for 7.9 (SD: 3.2) years, Rassi et al. have found an AF prevalence of 13.3 (SD 3.1%), with strong association with the risk of death (HR: 5.43 [2.91–10.13]) in univariate analysis.^273^
- In 330 patients with ChD (mean age 49 ± 12 years; 58% men), 26 of whom had AF, an analysis exploring the risk factors for ischemic cerebrovascular events has found no increase in the risk of stroke among ChD patients with AF as compared to ChD patients in sinus rhythm.^274^

## Awareness, Treatment and Control

### Anticoagulation

- There was a high variation in the use of anticoagulation in patients with AF, from 1.5% to 91%. Studies with samples from primary care were more likely to have low use of anticoagulation as compared to samples recruited from tertiary centers or cardiologists, as detailed below.
- Among 4638 subjects with AF in primary care centers of 658 municipalities in the state of Minas Gerais, Brazil [mean (SD) age, 70 (14) years; 54% men], who underwent ECG using telehealth in 2011, the use of VKAs was reported by 1.5%, and that of aspirin, by 3.1%.^241^
- In a study of patients from 125 primary care centers in 9 states of four Brazilian geographic regions, from January 2009 through April 2016, a subset of patients with AF (n=301) was identified, 189 (63%) of whom were at high risk for stroke; only 28 (15%) were regular oral anticoagulant users, and 102 (54%) were using aspirin.^242^
- The GARFIELD-AF Registry is a worldwide study that included patients (≥18 years) with AF diagnosed within the previous 6 weeks and at least 1 additional risk factor for stroke as judged by the study investigator. In Brazil, among 1041 patients included (82.3% by cardiologists) between 2010 and 2014, the mean (SD) age was 68 (13) years, 55% were males, 86% had CHA_2_DS_2_-VASc score ≥ 2, 19% were not using anticoagulation therapy at baseline, 26% were only receiving antiplatelet therapy, 29% were using VKAs, and 26% were receiving NOACs.^238^
- The IMPACT-AF,^275^a clustered randomized trial to improve treatment with anticoagulants in patients with AF conducted in Argentina, Brazil, China, India and Romania, has shown that two-thirds of the patients were on oral anticoagulation at baseline: 83% were on a VKA and 15% were on a NOAC. Patients from Brazil (n=360) were most often on oral anticoagulation at baseline (91%), and 27% were on NOACs. Of all patients taking VKAs in Brazil, 40.3% had INR values between 2 and 3 prior to the baseline visit.
- A cross-sectional study including 162 patients from a university hospital in the city of Porto Alegre, Brazil [mean age (SD), 69 (12) years; 57% males; 96% with CHA_2_DS_2_-VASc ≥ 2], has found that 55 (34%) patients were using anticoagulants and 80 (50%) patients were on aspirin.^276^
- A cross-sectional study from a university hospital in the city of Rio de Janeiro, Brazil, has included 659 consecutive hospitalizations due to decompensated HF, from January 2006 to December 2011. Patients with AF (n=264; 40%) had a median CHA_2_DS_2_-VASc score of 4, which was ≥ 2 in 90% of the patients. The anticoagulation rate was 53% on admission and 67% on discharge.^255^
- A stroke registry in the city of Joinville has described all 429 cases of stroke that occurred in 2015, of which, 87.2% (374/429) were ischemic strokes. Atrial fibrillation was detected in 11.4% (49/429) of the patients and in 58% (49/84) of all cardioembolic strokes. Of the 26 patients with known prior AF, 19 (73%) were not anticoagulated, 20 (77%) had a CHA_2_DS_2_-VASc score ≥ 3, and 21 (81%) had a HAS-BLED score < 3.^251^
- The quality of warfarin therapy has been evaluated using the parameter TTR in different samples in Brazil. In general, samples are not composed by only AF patients. Patients with AF using anticoagulants have worse TTR as compared to other indications, such as prosthetic valves. The TTR varied between 46% and 67% in the studies.^249 , 277 - 279^Age > 65 years, but not health literacy, was associated with a higher TTR.^278^

### Rhythm or rate control (medication, cardioversion, catheter ablation)

- A cross-sectional study with 167 AF patients has found that rate control was more common than rhythm control as treatment strategy (79% vs. 21%; P<0.001). In the subgroup with paroxysmal AF, both strategies were equally used (rate control, 53%; rhythm control, 47%; p = 0.69). Patients with persistent AF were more likely to be receiving rate control treatment (96% vs. 4%; P<0.001). Among those in rhythm control, amiodarone (43%), sotalol (16%), and propafenone (14%) were the drugs mostly prescribed. Beta-blockers were prescribed for 81% of the patients in rate control.^280^Amiodarone was mentioned by 83% of the doctors as the choice for rhythm control strategy.^281^
- Data from 125 primary care centers have found that, among the patients with AF (n=301), 91 (30.2%) were not receiving any type of treatment for rate or rhythm control. Of the remaining 210 patients on treatment, 147 (70%) used rate control agents (beta-blockers, digoxin, diltiazem, or verapamil) and 25 (12%) used at least one antiarrhythmic drug (amiodarone or propafenone). The simultaneous use of antiarrhythmic drugs and beta-blockers was reported by 36 (17%) respondents.^242^
- Atrial fibrillation ablation has been reported in the first Latin American Transcatheter Ablation Registry that included 742 patients for AF ablation, either first-time or re-do procedures, performed between January 1^st^and December 31^st^, 2012, in 18 centers in Brazil.^282^
- In a one-center case series, a total of 225 patients with paroxysmal AF [64 women (29%) and 161 men (61%)] underwent catheter ablation with a recurrence of 21% among women and of 20% among men (p = 0.89) in a 1-year follow-up.^283^

## Future Research

- The First Brazilian Cardiovascular Registry of Atrial Fibrillation: the RECALL study is a registry in AF that finished inclusion of patients in 2019 with 4584 subjects and will probably be published in 2020. It will be the largest Brazilian registry with data from all country regions regarding the characteristics and treatment of AF patients from 73 centers in Brazil.^284^
- Ongoing cohort studies, such as the ELSA-Brasil, have the potential to fill the information gaps on incidence, risk factors, risk prediction – including genetics – and prevention of AF in Brazil. To our knowledge, there is neither an original published study with information on the incidence of AF in Brazil nor longitudinal data on risk factors.
- Studies designed to screen AF in a population base or selected populations by use of ECG or screening devices are ongoing and should bring information on the relevance of including this strategy in primary care or specialized centers.
- Implementation strategies to enhance anticoagulation use among AF patients should be encouraged, particularly in primary care settings.

**Chart 1-1:** Data derived from Global Burden of Disease Study 2017 (GBD 2017).^66^

**Chart 1-2:** Data derived from Global Burden of Disease Study 2017 (GBD 2017).^66^

**Chart 1-3:** Data derived from Global Burden of Disease Study 2017 (GBD 2017).^66^

**Chart 1-4:** Data derived from Global Burden of Disease Study 2017 (GBD 2017).^66^

**Chart 1-5:** Data derived from Global Burden of Disease Study 2017 (GBD 2017).^66^

**Chart 1-6:** Data derived from Global Burden of Disease Study 2017 (GBD 2017).^66^

**Chart 1-7:** Data derived from Global Burden of Disease Study 2017 (GBD 2017).^66^

**Chart 1-8:** Data derived from Global Burden of Disease Study 2017 (GBD 2017).^66^

**Chart 1-9:** Source: Brazilian Mortality Information System (Sistema de Informações sobre Mortalidade – SIM) with redistribution of ill-defined causes and correction for underreporting (according to GBD 2017 coefficients) and IBGE population.^56^

**Chart 1-10:** Data derived from Global Burden of Disease Study 2017 (GBD 2017).^66^

**Chart 1-11:** Data derived from Global Burden of Disease Study 2017 (GBD 2017).^66^

**Chart 1-12:** Data derived from DATASUS.^56^

**Chart 1-13:** Data derived from Global Burden of Disease Study 2017 (GBD 2017).^66^

**Chart 1-14:** Data derived from Global Burden of Disease Study 2017 (GBD 2017).^66^

**Chart 1-15:** Data derived from DATASUS.^56^

**Chart 1-16:** Data derived from DATASUS.^56^

**Chart 2-1:** Data derived from the Global Burden of Disease Study 2017 (GBD 2017).^109^

**Chart 2-2:** Data derived from the Global Burden of Disease Study 2017 (GBD 2017).^109^

**Chart 2-4:** Data derived from the Global Burden of Disease Study 2017 (GBD 2017).^109^

**Table 2-2:** – Number of deaths and age-standardized mortality rates (per 100 000) for stroke and ischemic stroke in Brazil and Brazilian Federative Units, 1990 and 2017, and percent change

Cause of death and location	1990		2017		Percent change (95% UI)
	Number (95% UI)	Rate (95% UI)	Number (95% UI)	Rate (95% UI)	
***B.2.3 - Stroke***					
Acre	146 (140;152)	101 (96,3;104,9)	318 (297;337)	59,3 (55,4;63,1)	-41,3 (-45,4;-36,8)
Alagoas	1767 (1697;1843)	138,2 (133;143,6)	2341 (2227;2457)	79,4 (75,4;83,5)	-42,6 (-45,8;-39,3)
Amapá	74 (70;77)	90,1 (85;93,9)	242 (226;256)	57,7 (54;61,3)	-35,9 (-40;-31,5)
Amazonas	679 (636;714)	99 (92,7;103,9)	1407 (1332;1476)	58,7 (55,7;61,7)	-40,7 (-44,4;-36,6)
Bahia	6405 (6059;6787)	96 (90,8;101,7)	9239 (8841;9681)	58,3 (55,7;61,4)	-39,3 (-43,1;-35)
Brazil	97101 (95272;98780)	122,9 (120,6;125)	122783 (119899;125348)	56,6 (55,2;57,8)	-54 (-55,1;-53)
Ceará	3266 (3038;3496)	80,7 (74,7;86,7)	5444 (5193;5691)	54,8 (52,2;57,2)	-32,2 (-37,5;-26,3)
Distrito Federal	536 (516;556)	117,2 (113;121,2)	961 (902;1028)	53,5 (49,9;57,1)	-54,4 (-57,5;-51)
Espírito Santo	1972 (1914;2030)	172,1 (166,9;177,1)	2188 (2079;2303)	54,6 (51,9;57,4)	-68,3 (-69,9;-66,5)
Goiás	1920 (1845;2004)	121,4 (116,7;126,6)	2925 (2779;3091)	48,2 (45,8;50,9)	-60,3 (-62,4;-58)
Maranhão	2563 (2374;2779)	101 (93,1;110,6)	4261 (4020;4592)	68,9 (65;74,3)	-31,7 (-36,6;-26,6)
Mato Grosso	637 (596;683)	93,3 (87,3;99,6)	1407 (1328;1491)	51,9 (48,9;55)	-44,4 (-48,8;-38,9)
Mato Grosso do Sul	937 (904;973)	121,6 (117,6;126,2)	1554 (1477;1642)	60,4 (57,4;63,8)	-50,3 (-53,4;-47,3)
Minas Gerais	10859 (10542;11270)	127 (123,3;131,2)	12638 (12067;13238)	50,5 (48,2;53)	-60,2 (-62,2;-58,1)
Pará	2210 (2066;2326)	117,3 (109,8;123,5)	3993 (3776;4201)	66 (62,3;69,5)	-43,8 (-47,6;-39,4)
Paraíba	2070 (1958;2194)	92,5 (87,4;98,1)	2856 (2642;3073)	60,8 (56,2;65,4)	-34,3 (-40,6;-27,8)
Paraná	6603 (6394;6801)	174,4 (169;179,4)	7455 (7111;7818)	63,8 (60,9;66,8)	-63,4 (-65,2;-61,5)
Pernambuco	5744 (5538;5948)	145,5 (140,2;150,6)	6553 (6236;6877)	68,7 (65,3;72,2)	-52,8 (-55,1;-50)
Piaui	1422 (1322;1536)	106,4 (98,8;115,1)	2294 (2169;2512)	63,7 (60,2;69,6)	-40,1 (-44,6;-35,2)
Rio de Janeiro	13533 (13076;13920)	160,9 (155,4;165,5)	12650 (12074;13184)	60 (57,3;62,5)	-62,7 (-64,6;-60,8)
Rio Grande do Norte	1180 (1106;1257)	74,2 (69,7;79,2)	1704 (1604;1806)	45 (42,3;47,7)	-39,4 (-44,7;-33,7)
Rio Grande do Sul	7282 (6967;7528)	135,4 (129,5;139,9)	8818 (8373;9257)	60,3 (57,2;63,3)	-55,5 (-57,9;-53)
Rondônia	384 (357;413)	140,1 (131,2;149,5)	764 (691;849)	60,9 (55,2;67,3)	-56,5 (-61,2;-51,4)
Roraima	50 (45;54)	126,6 (117,3;136,8)	152 (135;171)	61,3 (54,7;68,5)	-51,6 (-58;-44,9)
São Paulo	20625 (19917;21335)	121,8 (117,5;126,1)	24963 (23878;26094)	51 (48,7;53,3)	-58,1 (-60,4;-55,8)
Santa Catarina	2947 (2832;3048)	148,9 (142,8;153,8)	3539 (3358;3729)	51,9 (49,3;54,6)	-65,1 (-66,9;-63)
Sergipe	919 (877;957)	110,6 (105,7;115,2)	1328 (1269;1392)	65,5 (62,5;68,7)	-40,8 (-44,4;-37,2)
Tocantins	373 (330;409)	121,4 (110,6;132,1)	790 (741;853)	60,1 (56,3;64,9)	-50,5 (-55,4;-44,6)
***B.2.3.1 - Ischemic stroke***					
Acre	51 (48;54)	43,7 (41,2;45,9)	110 (102;120)	22,5 (20,6;24,4)	-48,5 (-53,1;-43,4)
Alagoas	708 (669;750)	61,4 (58,2;64,9)	906 (851;964)	31,9 (29,9;34,1)	-48 (-52,3;-43,7)
Amapá	29 (27;31)	43,2 (40,2;45,5)	83 (76;89)	22,9 (21;24,6)	-46,9 (-51,3;-42,1)
Amazonas	247 (228;263)	43,4 (40;46,1)	492 (461;525)	22,6 (21,1;24,2)	-47,8 (-52;-42,6)
Bahia	2466 (2310;2638)	40,2 (37,7;42,9)	3488 (3281;3697)	21,9 (20,6;23,3)	-45,4 (-50;-40,4)
Brazil	37045 (36222;37909)	54,8 (53,6;55,9)	47453 (45939;48713)	22,6 (21,9;23,2)	-58,7 (-60;-57,4)
Ceará	1279 (1165;1399)	33,5 (30,5;36,6)	2266 (2119;2411)	22,5 (21,1;24)	-32,7 (-39,9;-24,2)
Distrito Federal	153 (144;162)	50,9 (48,2;53,5)	325 (299;353)	22,4 (20,6;24,3)	-56,1 (-60,1;-51,7)
Espírito Santo	746 (712;782)	80,9 (77,5;84,4)	810 (759;869)	21,1 (19,7;22,6)	-74 (-75,8;-71,9)
Goiás	623 (589;658)	52,2 (49,4;55,1)	1005 (940;1081)	18,1 (16,9;19,5)	-65,3 (-67,9;-62,5)
Maranhão	812 (712;937)	37,2 (32,6;43)	1582 (1457;1733)	26,3 (24,3;28,9)	-29,2 (-36,4;-21,1)
Mato Grosso	226 (209;245)	42 (39;45,2)	492 (459;529)	20,5 (19,1;22)	-51,2 (-55,9;-46)
Mato Grosso do Sul	317 (300;334)	51,3 (48,8;54)	543 (508;584)	22,7 (21,2;24,4)	-55,8 (-59,7;-51,7)
Minas Gerais	3976 (3792;4182)	56,4 (53,8;59)	4748 (4435;5040)	19,2 (17,9;20,4)	-65,9 (-68,5;-63,4)
Pará	959 (890;1015)	58 (53,8;61,2)	1551 (1442;1657)	27,5 (25,5;29,3)	-52,6 (-56,7;-48,4)
Paraíba	853 (787;928)	40 (37;43,3)	1163 (1065;1266)	24,2 (22,1;26,5)	-39,4 (-46,7;-31,4)
Paraná	2537 (2416;2649)	82,5 (78,8;86,1)	3107 (2930;3296)	28,1 (26,5;29,8)	-65,9 (-68,2;-63,6)
Pernambuco	2296 (2183;2413)	65,8 (62,7;69)	2429 (2266;2589)	26,3 (24,4;28,1)	-60,1 (-63,1;-56,7)
Piaui	523 (472;587)	43,8 (39,6;49,1)	922 (859;1023)	25,6 (23,8;28,4)	-41,7 (-47,5;-35,4)
Rio de Janeiro	4769 (4552;4985)	68,3 (65,2;71,3)	4520 (4224;4807)	22 (20,6;23,4)	-67,8 (-70,1;-65,2)
Rio Grande do Norte	565 (523;611)	36,9 (34,2;39,8)	699 (648;751)	18,2 (16,8;19,6)	-50,7 (-56,3;-44,9)
Rio Grande do Sul	3219 (3048;3377)	70,1 (66,5;73,2)	3691 (3446;3933)	25,6 (23,9;27,4)	-63,4 (-66,1;-60,6)
Rondônia	110 (101;120)	61,3 (57;65,7)	282 (253;314)	25,1 (22,7;28)	-59 (-63,6;-53,7)
Roraima	15 (13;16)	59,1 (54,5;64,2)	52 (46;58)	26,2 (23,2;29,3)	-55,7 (-62,1;-48,8)
São Paulo	7894 (7483;8299)	56,8 (53,9;59,6)	9945 (9357;10577)	21,3 (20;22,7)	-62,5 (-65,3;-59,5)
Santa Catarina	1164 (1104;1224)	71,1 (67,5;74,5)	1419 (1328;1513)	22,4 (20,9;23,8)	-68,6 (-70,8;-66,1)
Sergipe	389 (367;411)	49,1 (46,2;51,8)	526 (494;559)	26,9 (25,2;28,6)	-45,3 (-49,7;-40,5)
Tocantins	119 (107;132)	53,8 (48,5;59,7)	297 (275;320)	23,6 (21,9;25,5)	-56 (-60,9;-49,9)

*Source: Global Burden of Disease Study 2017, Institute for Health Metrics and Evaluation.^109^*

**Table 2-6:** – Age-standardized DALY rates (per 100 000) for stroke and stroke subtypes in Brazil, 1990 and 2017, and percent change

Cause of death and age group	1990	2017	Percent change (95% UI)
***B.2.3- Stroke***			
15-49 years	900.8 (875.2;928)	427.2 (412;442.6)	-52.6 (-54.4;-50.8)
50-69 years	6472.1 (6299.3;6661.2)	2930.7 (2823.1;3041.8)	-54.7 (-56.2;-53.1)
5-14 years	76.6 (65.6;82.7)	34.2 (31;37.5)	-55.3 (-59.6;-49.8)
70+ years	15340.3 (14965.2;15714.6)	8044.9 (7764.8;8361)	-47.6 (-49;-46.2)
Age-standardized	2511.9 (2457.3;2567.6)	1145.3 (1107.8;1185.3)	-54.4 (-55.5;-53.2)
All Ages	1638.6 (1601.9;1675.6)	1225 (1185.3;1267.6)	-25.2 (-27.2;-23.1)
Under 5	374.3 (302.2;473.7)	60.2 (51.7;70.9)	-83.9 (-88.5;-77.8)
***B.2.3.1- Ischemic stroke***			
15-49 years	125.4 (118.1;134)	60.6 (54.5;67.2)	-51.6 (-56.6;-48.1)
50-69 years	1815 (1728.7;1901.4)	750.4 (693.5;808.9)	-58.7 (-60.9;-56.4)
5-14 years	9.2 (7.6;11.3)	4.2 (3.3;5.5)	-53.9 (-59.4;-48.5)
70+ years	8120.6 (7833.9;8406.8)	4071.1 (3858.5;4306.3)	-49.9 (-51.8;-47.8)
Age-standardized	871.4 (841.1;902.4)	387.3 (363.8;411.5)	-55.6 (-57.3;-53.8)
All Ages	489.8 (471.5;508.9)	399.7 (375.2;425.1)	-18.4 (-21.8;-15.1)
Under 5	21.6 (15.5;30.9)	2.7 (2.2;3.4)	-87.4 (-92.3;-79.9)
***B.2.3.2- Intracerebral hemorrhage***			
15-49 years	551.1 (532.8;582.6)	231.9 (219.7;241.4)	-57.9 (-61.3;-55.8)
50-69 years	3889.2 (3785.6;4001.8)	1681.4 (1626.7;1736.2)	-56.8 (-58.4;-55.1)
5-14 years	22.7 (19;25.3)	9.7 (8.4;11)	-57.2 (-64.5;-49.3)
70+ years	6557.5 (6353.6;6729.9)	3451.2 (3343.6;3564.8)	-47.4 (-49.2;-45.5)
Age-standardized	1322.1 (1291.8;1358.2)	576.9 (560.7;594.9)	-56.4 (-57.8;-55.1)
All Ages	893 (872.1;919.4)	626.5 (608.5;645.9)	-29.9 (-32.3;-27.7)
Under 5	89.5 (56.4;137.1)	10 (7.9;12.6)	-88.8 (-93.9;-79.4)
***B.2.3.3- Subarachnoid hemorrhage***			
15-49 years	224.3 (198.4;234.1)	134.6 (127.8;146.1)	-40 (-43.9;-30.9)
50-69 years	767.9 (694.1;806.4)	499 (472.4;530.5)	-35 (-39.1;-28.4)
5-14 years	44.7 (38;48.3)	20.3 (18.3;22.3)	-54.7 (-58.9;-47.7)
70+ years	662.2 (627.5;712.9)	522.6 (484.9;553)	-21.1 (-27.7;-15.6)
Age-standardized	318.4 (287;332.2)	181 (173.1;191)	-43.1 (-46.3;-37.2)
All Ages	255.7 (228.7;267.6)	198.8 (189.9;210.2)	-22.2 (-26.8;-13.2)
Under 5	263.3 (204;315.7)	47.5 (40.8;56.2)	-82 (-86.4;-74)

*Source: Global Burden of Disease Study 2017, Institute for Health Metrics and Evaluation.^109^*

**Table 2-7:** – Number of DALYs and age-standardized DALY rates (per 100 000) for stroke and stroke subtypes for men, in Brazil and its Federative Units, 1990 and 2017, and percent change

Cause of death and location	1990		2017		Percent change (95% UI)
	Number (95% UI)	Rate (95% UI)	Number (95% UI)	Rate (95% UI)	
***B.2.3- Stroke***					
Acre	2185.8 (2055.7;2310)	2076.4 (1959.4;2190.7)	4056.6 (3718;4393)	1351.9 (1238.4;1462.5)	-34.9 (-40.6;-28.6)
Alagoas	23037.9 (21383.3;24816.4)	3180.3 (2982.5;3389.5)	25242.2 (23619.6;26883.8)	1808.9 (1691.8;1922.6)	-43.1 (-47.4;-38.5)
Amapá	1034.6 (965.2;1094.9)	1809.2 (1692.8;1912.7)	3381.8 (3134.1;3607)	1374.3 (1282.1;1461.9)	-24 (-29.8;-17.9)
Amazonas	9553.6 (8816.9;10213.4)	2093.4 (1938.6;2239.5)	17349.2 (15990.9;18542.4)	1276.7 (1184.2;1362.4)	-39 (-44;-33.1)
Bahia	79746.6 (73741.8;86039.8)	2199.7 (2029.8;2372.4)	105759.2 (99250.7;112353.9)	1484.6 (1393.9;1577.1)	-32.5 (-38.3;-26.2)
Brazil	1340726.1 (1301608;1381197.7)	2901.8 (2824.3;2983.3)	1365881.1 (1320048.1;1415334.4)	1334.5 (1289.5;1381.5)	-54 (-55.4;-52.5)
Ceará	40155.1 (36356.6;44353)	1867.9 (1704.3;2040.7)	52142.8 (49017.4;55405.8)	1187.3 (1115.9;1260.7)	-36.4 (-42.4;-29.7)
Distrito Federal	8537.6 (8039.6;9059.3)	2500.7 (2380;2633.5)	11737.5 (10824.2;12680.1)	1133 (1049;1220.2)	-54.7 (-58.4;-50.9)
Espírito Santo	27222.3 (26107.5;28325.5)	3603.3 (3461.5;3739.1)	24872.8 (23306.7;26481.7)	1282 (1202.4;1361.2)	-64.4 (-66.8;-61.8)
Goiás	29376.9 (27812.5;31309.7)	2478.4 (2351.1;2632)	36251.9 (33907.6;38650.9)	1128.3 (1056.4;1200.3)	-54.5 (-57.7;-50.9)
Maranhão	48618.1 (43268.6;54586.2)	3233.4 (2920.4;3559.2)	52331.8 (48765.6;56040.7)	1736.2 (1619.1;1857)	-46.3 (-52.5;-39.3)
Mato Grosso	10626.9 (9676.9;11652.5)	2101.4 (1919.4;2294.5)	18983.8 (17675.4;20267.4)	1183.4 (1106.4;1259.7)	-43.7 (-49;-37.5)
Mato Grosso do Sul	14122.2 (13298.1;14951)	2705.6 (2569.5;2851.5)	18664 (17425.5;19960.7)	1387.8 (1299.8;1482.5)	-48.7 (-52.3;-44.6)
Minas Gerais	161794.5 (154754.4;170766.6)	3124.2 (2993.3;3285.8)	143536.1 (135085.5;153049.9)	1231.9 (1157.6;1313.9)	-60.6 (-63.5;-57.7)
Pará	28382.9 (26206.8;30473.3)	2437.9 (2254.7;2606.9)	51273.2 (47548.3;54706.6)	1571.3 (1462.4;1670.3)	-35.5 (-41.2;-29.3)
Paraíba	22193 (20368;24263.9)	1936.3 (1764.3;2121.9)	27109.7 (24423.5;30026.9)	1350.3 (1216.8;1495.6)	-30.3 (-38.8;-20.4)
Paraná	92749.7 (88831.5;96784.9)	3678.5 (3538.6;3825.8)	80945 (75854.2;86058.7)	1400.7 (1313.7;1487)	-61.9 (-64.5;-59.2)
Pernambuco	65395.8 (62156.5;68861.5)	2991 (2847;3142.5)	69666.6 (65443.1;74126)	1614.1 (1513.1;1713.9)	-46 (-49.6;-42.1)
Piauí	19669.4 (17738.3;21781.8)	2619.3 (2388.7;2872.8)	23916.7 (22435.2;25778.9)	1448.6 (1356.9;1561.6)	-44.7 (-50;-38.8)
Rio de Janeiro	184276.5 (175451.6;192077)	3962.5 (3782.2;4120.9)	137791.2 (129209;147280.8)	1484.8 (1394;1583.2)	-62.5 (-65.1;-59.8)
Rio Grande do Norte	13443.1 (12350.1;14560.8)	1633.8 (1495.3;1773.6)	18039.1 (16816.2;19404)	1085.1 (1009.3;1170.2)	-33.6 (-40.6;-26.1)
Rio Grande do Sul	90124.8 (85087.9;94419.2)	2897.9 (2730.7;3029.6)	85557 (78513.9;91514.3)	1310.6 (1204.4;1399.5)	-54.8 (-58.2;-51.3)
Rondônia	7265.8 (6579.4;8016.9)	3011.3 (2742.2;3291.6)	9560.1 (8267.2;11057.2)	1294.1 (1124.2;1488.5)	-57 (-63.3;-49.7)
Roraima	949.2 (833.8;1085.1)	2284.8 (2043.1;2564.1)	2144.1 (1803.1;2514.8)	1185.9 (1008.1;1374.6)	-48.1 (-57.3;-38.1)
Santa Catarina	37772.9 (35903.6;39627.8)	3023.9 (2877.4;3170.4)	37174.2 (34286.7;39901.5)	1075 (995.5;1150.9)	-64.4 (-67;-61.7)
São Paulo	306031.7 (291022;320718.4)	2950.4 (2815.7;3084)	284773.8 (266487.2;302539.1)	1234.2 (1156.7;1308.1)	-58.2 (-61;-55)
Sergipe	10495.6 (9904.8;11088.7)	2433.8 (2298.2;2564.6)	14207 (13364.2;15173.1)	1497.7 (1410.4;1596.9)	-38.5 (-43.4;-33.2)
Tocantins	5963.4 (4989.1;6786.8)	2363.3 (2064.7;2648.7)	9413.7 (8582.8;10360.7)	1316 (1200;1448.6)	-44.3 (-51.5;-34.9)
***B.2.3.1- Ischemic stroke***					
Acre	618.6 (570.6;666.7)	717.8 (663.9;771.4)	1173.2 (1053.7;1293.6)	441.9 (397;487.4)	-38.4 (-45.2;-31.1)
Alagoas	7174.6 (6632.2;7849.1)	1129.9 (1044.1;1234.1)	8207.6 (7511.2;8930.5)	631.6 (579.3;686.7)	-44.1 (-50.1;-37.8)
Amapá	321.8 (294.1;347.8)	700 (641.2;755.6)	983.4 (886.2;1080.5)	479 (432.3;523.2)	-31.6 (-38.3;-24.5)
Amazonas	2794 (2546.9;3043.9)	750.3 (685.8;817.2)	4980.4 (4506.6;5491.5)	422.3 (382.8;464.2)	-43.7 (-49.3;-37.3)
Bahia	23360.8 (21326.4;25677.3)	723.9 (659.9;793.4)	32950.3 (30333.4;35703.4)	486 (447.3;526.4)	-32.9 (-40;-25.2)
Brazil	399936.6 (384258.7;417610.5)	1011 (973.7;1050.4)	444686.8 (419137.4;471530)	463.4 (436.9;490.2)	-54.2 (-56.5;-52.2)
Ceará	11960.3 (10725.4;13374.7)	616.7 (550.4;690.6)	17787 (16310.4;19333.8)	418.8 (383.3;456.6)	-32.1 (-41;-23.5)
Distrito Federal	2081.9 (1927.3;2271.5)	863.5 (801.5;934.9)	3486.6 (3111.4;3853.3)	415.1 (374.3;456)	-51.9 (-56.9;-46.2)
Espírito Santo	8136.6 (7612.8;8726.9)	1280.8 (1203.2;1368.2)	7872 (7206.4;8608.3)	435.9 (399.3;475.3)	-66 (-69.1;-62.6)
Goiás	7772.7 (7148.3;8470.7)	799.3 (741.5;864.3)	10856.7 (9840;11962.2)	369.8 (336.1;405.8)	-53.7 (-58.1;-48.6)
Maranhão	11361.5 (10150.7;12898.4)	901.9 (799.8;1020.4)	16393.4 (14900.7;17870.1)	578.9 (527.5;630.6)	-35.8 (-43.4;-26.8)
Mato Grosso	3275.8 (2935.7;3611.7)	809.8 (728.8;890)	5907.8 (5344;6475.2)	410.9 (371.6;449.3)	-49.3 (-54.9;-43.2)
Mato Grosso do Sul	4003.7 (3719.2;4320.3)	920.9 (857.5;990.1)	5616.4 (5139.2;6154.2)	451.4 (413.3;493.6)	-51 (-55.7;-45.4)
Minas Gerais	45397.8 (42188;49142.4)	1032 (966.8;1106.1)	45377.1 (41325.5;49776.8)	404.9 (369;443.4)	-60.8 (-64.6;-56.6)
Pará	9746.8 (8897.6;10553.4)	988.9 (905.2;1067)	16640.4 (15023.9;18274.6)	567 (512.9;618.9)	-42.7 (-48.5;-35.9)
Paraíba	7338.7 (6537.4;8331.7)	662.5 (589.7;752.4)	8861 (7875.9;9862.6)	451.6 (402;502.2)	-31.8 (-42.2;-19.6)
Paraná	29844.1 (27803.5;31821.9)	1395.8 (1309.4;1479.1)	29745 (27186.2;32365.5)	545.8 (499;592.4)	-60.9 (-64.7;-56.8)
Pernambuco	20621.5 (19138.5;22088.7)	1056.1 (986.5;1126.5)	20528.9 (18761.4;22301)	512.2 (469;555.5)	-51.5 (-56.3;-46.5)
Piauí	5761.5 (5099.4;6525)	885.1 (784;1003.8)	7811.8 (7126.5;8529.2)	487.7 (445.2;532.9)	-44.9 (-51.4;-37.5)
Rio de Janeiro	49857.6 (46267.7;53745.2)	1274.3 (1191;1361.4)	41594.5 (37885.7;45512.7)	476.1 (433.5;520.1)	-62.6 (-66.3;-58.8)
Rio Grande do Norte	5114.5 (4628.4;5664.3)	660.2 (597.4;731)	6177.2 (5613.2;6753.1)	387.4 (351.2;423.7)	-41.3 (-48.9;-33.4)
Rio Grande do Sul	31368.4 (29142.6;33607.5)	1192.5 (1112.2;1269.8)	29881.3 (26896.7;32904.7)	479.7 (434.4;526.7)	-59.8 (-63.4;-55.4)
Rondônia	1976.1 (1763.6;2194.1)	1102.7 (997.2;1205.7)	3130.3 (2728.8;3606.5)	471.6 (411.8;543.1)	-57.2 (-63.5;-49.8)
Roraima	232.6 (203.3;266.8)	829.1 (738.7;936)	622.5 (532.3;717.9)	422.1 (361.6;485.3)	-49.1 (-57.1;-39.7)
Santa Catarina	12066 (11272.6;12969.4)	1161.9 (1090.7;1245.2)	12855.1 (11617.8;14085.1)	406.9 (370.4;444.8)	-65 (-68.3;-61.2)
São Paulo	92643.3 (86120.5;99963.6)	1076.5 (1003.8;1149)	97487.5 (88497;106189.9)	452.3 (411.4;491.7)	-58 (-62;-53.9)
Sergipe	3514.4 (3243.3;3784.8)	896.1 (827.4;963)	4688.6 (4301.9;5103)	537 (492.6;584.8)	-40.1 (-46.2;-32.9)
Tocantins	1590.9 (1380.1;1806.6)	799.2 (704.9;903.2)	3070.7 (2762.7;3412.2)	454.9 (409.6;504.6)	-43.1 (-50.8;-33.4)
***B.2.3.2- Intracerebral hemorrhage***					
Acre	1167.7 (1091.3;1249.8)	1103.5 (1027.3;1177)	2307.8 (2068.3;2536.5)	749.5 (674.7;823.3)	-32.1 (-39.6;-23.9)
Alagoas	12772.1 (11548.1;13954.3)	1755.3 (1608.7;1896.2)	14334.6 (13275.7;15483.4)	1004.8 (929.5;1085.5)	-42.8 (-48.1;-36.7)
Amapá	560.5 (514.3;598.7)	932.7 (858.7;997.3)	1937.6 (1732.9;2099.3)	748.9 (677.5;810.1)	-19.7 (-27.5;-11)
Amazonas	5416 (4917.7;5858.5)	1146.7 (1043.6;1240.1)	10027.2 (8951.6;10869.8)	713.8 (645.2;772.7)	-37.8 (-44;-30.6)
Bahia	44882.2 (40975.9;49072.8)	1234.7 (1126.8;1348.4)	59717.2 (55175.3;64266.8)	825.2 (762.5;886.5)	-33.2 (-40.1;-25.3)
Brazil	770369.3 (745080;807453.6)	1601 (1552.2;1665.3)	746738 (720304;771926)	709.9 (684.6;733.7)	-55.7 (-57.5;-53.9)
Ceará	21836.7 (19497.2;24283.5)	1026.3 (913.7;1136)	27995 (25907.6;30104.9)	631 (584.4;678.5)	-38.5 (-44.8;-30)
Distrito Federal	5129.2 (4782;5672.3)	1378.5 (1293.1;1493.8)	6413 (5860.8;7055.5)	577.3 (530.7;632.9)	-58.1 (-62.2;-53.6)
Espírito Santo	15950.1 (15082.9;17014.5)	2001.8 (1896;2121.2)	13712.2 (12714;14769.9)	687.1 (637.9;738.7)	-65.7 (-68.6;-62.6)
Goiás	17762.5 (16634;19235.8)	1430.3 (1342.8;1543.6)	20310.6 (18792.9;21876.5)	613.1 (566.7;659.2)	-57.1 (-61.3;-52.8)
Maranhão	28903.2 (24480.3;33872.2)	1930 (1685.5;2188.7)	28870.1 (26565.9;31653.8)	946.4 (871.3;1037.4)	-51 (-58.3;-42.1)
Mato Grosso	5628.1 (5070.6;6250.4)	1062.2 (956.7;1176.1)	10360.1 (9507.4;11268.1)	622.6 (573.7;675.4)	-41.4 (-48.1;-33.7)
Mato Grosso do Sul	8383 (7809.6;8980.7)	1528.5 (1437.3;1632.1)	10743.1 (9937.4;11597)	776.6 (718.3;836.1)	-49.2 (-53.7;-44.1)
Minas Gerais	96380.6 (91111.2;106323.6)	1776.3 (1681.8;1936)	78424.5 (72806.4;84694.7)	659.8 (613.3;712.3)	-62.9 (-66.6;-59.4)
Pará	15142.2 (13771.6;16408.1)	1245 (1128.4;1348.2)	28065.2 (25497.2;30355.1)	832.9 (758.6;901.5)	-33.1 (-40.6;-24.6)
Paraíba	11940.1 (10701.6;13143.1)	1052.4 (943.8;1157.3)	15086.6 (13425.1;17023.7)	746.3 (664.9;841.2)	-29.1 (-39;-17.3)
Paraná	52954.5 (49873.2;56180.2)	1974.1 (1867.5;2087)	41469.1 (38186.9;44696.4)	694.9 (641.3;748.6)	-64.8 (-68.2;-61.6)
Pernambuco	38213.7 (35818.5;40711.7)	1697.4 (1591.6;1806.2)	40983.9 (38031;44233.3)	927.6 (861.4;998.2)	-45.4 (-49.9;-40.2)
Piauí	11080.4 (9834.8;12452)	1461.3 (1308.5;1628.8)	13195.7 (12198.6;14309.5)	791.9 (731.9;859.9)	-45.8 (-51.9;-38.3)
Rio de Janeiro	112722 (106431.2;121878.2)	2297.5 (2175.7;2458.4)	79599.8 (73519.4;85665.3)	832.6 (772;895.4)	-63.8 (-66.8;-60.5)
Rio Grande do Norte	6460 (5806.7;7074.2)	795 (712.9;873.8)	9624.8 (8747.5;10531.3)	571.4 (519.9;624.3)	-28.1 (-37.1;-17.4)
Rio Grande do Sul	49620.5 (46236.1;52719.3)	1469.8 (1370.4;1556.4)	46196.2 (41693.4;49971.8)	687 (620.3;740.7)	-53.3 (-57.8;-48.8)
Rondônia	4291.7 (3827.1;4779.5)	1649.2 (1485.9;1829.5)	5219.4 (4433.5;6116.6)	681.4 (579.1;794.2)	-58.7 (-65.9;-50.3)
Roraima	540.1 (467.9;624.4)	1208.3 (1063.9;1371.2)	1177.9 (971;1399.6)	616 (514.5;726.2)	-49 (-59.4;-37.6)
Santa Catarina	21310.9 (20042.4;22571.8)	1599.1 (1505.4;1692.3)	19391.8 (17418.6;21070.9)	538.4 (485.1;583.1)	-66.3 (-69.7;-63)
São Paulo	172309 (162089.8;184997.1)	1566.5 (1474;1673.6)	148579.1 (137505.7;159473)	622.3 (576.6;667)	-60.3 (-63.5;-56.5)
Sergipe	5586 (5171.6;5990.8)	1301.8 (1199.4;1396)	7869.5 (7245.8;8471.6)	807.4 (743.9;870.3)	-38 (-44.4;-30.7)
Tocantins	3426.4 (2825.3;3946.7)	1306.7 (1114.8;1488.8)	5125.9 (4593.1;5697.7)	703.9 (629.4;782.3)	-46.1 (-53.8;-35.7)
***B.2.3.3- Subarachnoid hemorrhage***					
Acre	399.5 (338.4;452.9)	255 (224.7;282.1)	575.6 (512.3;647.3)	160.5 (142.9;180.2)	-37.1 (-46.3;-25.3)
Alagoas	3091.2 (2560.5;3728.8)	295.1 (256.9;338.7)	2700.1 (2424.9;3128.4)	172.5 (155.2;198.2)	-41.5 (-51.6;-28)
Amapá	152.3 (137.1;172.9)	176.6 (159.2;208.9)	460.9 (405.1;563.3)	146.5 (129;179.3)	-17.1 (-28.4;-3.6)
Amazonas	1343.6 (1203.1;1505.9)	196.3 (176.8;224.8)	2341.6 (2099;2674.1)	140.6 (126.5;160.4)	-28.4 (-37.6;-17.3)
Bahia	11503.7 (10112.3;12962.3)	241.1 (213.5;272.3)	13091.8 (11817.2;15500)	173.4 (156.8;204.8)	-28.1 (-37.4;-15.5)
Brazil	170420.1 (137803.5;181911.2)	289.9 (237.9;307.2)	174456.3 (165617.4;190897.1)	161.2 (152.9;175.4)	-44.4 (-48.2;-31.2)
Ceará	6358.1 (4960.5;8487.7)	224.9 (181.7;281.6)	6360.8 (5714;7533.9)	137.4 (123.6;162)	-38.9 (-51.8;-23.8)
Distrito Federal	1326.5 (1012.9;1471.9)	258.7 (192.2;284.4)	1837.9 (1527.4;2085.5)	140.6 (113.6;158.6)	-45.7 (-52.6;-36.5)
Espírito Santo	3135.6 (2225.9;3470.1)	320.7 (225.1;353.4)	3288.6 (2923.6;3700.8)	159 (141.6;178.1)	-50.4 (-57.2;-31.6)
Goiás	3841.6 (3321.9;4212.2)	248.8 (213.8;272.9)	5084.6 (4525.1;5969.8)	145.5 (130.1;169.2)	-41.5 (-48.9;-27.5)
Maranhão	8353.4 (6626.3;10082.5)	401.5 (324.6;464)	7068.2 (6109.5;7958.4)	210.9 (181.9;237.7)	-47.5 (-55.7;-34.1)
Mato Grosso	1723 (1532.7;1937.7)	229.3 (203.9;259.4)	2715.9 (2409.9;3105)	149.9 (133.5;169.8)	-34.6 (-43.9;-24.4)
Mato Grosso do Sul	1735.6 (1459.8;1929.6)	256.1 (216.3;282.8)	2304.4 (2053.5;2587.7)	159.7 (142.7;178.4)	-37.7 (-46.5;-26.4)
Minas Gerais	20016 (14378.2;22114)	315.9 (228;348.2)	19734.6 (17598.5;21729.3)	167.2 (149.8;183.9)	-47.1 (-53.4;-30.5)
Pará	3493.9 (3052.8;3985.1)	204 (180.5;243)	6567.6 (5796.3;7642.6)	171.5 (151.3;200.7)	-15.9 (-26.5;-2.6)
Paraíba	2914.1 (2445;3552.5)	221.5 (186.3;261.9)	3162 (2739.4;3774.1)	152.3 (132;181.2)	-31.2 (-44.8;-12.3)
Paraná	9951.1 (7039.7;10916.8)	308.7 (213.4;339.7)	9730.8 (8612.3;10810.2)	160 (141.5;176.9)	-48.2 (-54.6;-32.2)
Pernambuco	6560.6 (5947.2;7353.4)	237.5 (216.1;264.8)	8153.8 (7346.1;9731.2)	174.3 (157.3;207.5)	-26.6 (-35.5;-15.6)
Piauí	2827.6 (2252.6;3497.2)	273 (224.6;324.2)	2909.2 (2626.4;3236.3)	168.9 (152.7;187.7)	-38.1 (-49;-24.2)
Rio de Janeiro	21696.8 (12606.3;24282.3)	390.7 (228.4;436.3)	16596.8 (14887;18587.5)	176.1 (157.6;196.4)	-54.9 (-61.3;-27.9)
Rio Grande do Norte	1868.7 (1569.2;2321.5)	178.6 (151.8;242.9)	2237 (1953.7;3000.2)	126.3 (110.4;169.3)	-29.3 (-41.4;-13.1)
Rio Grande do Sul	9135.9 (8004;9984.6)	235.6 (207.7;257.1)	9479.5 (8496.8;10627.1)	143.9 (129.5;161.1)	-38.9 (-46;-29.1)
Rondônia	998 (861.6;1120.5)	259.4 (226.5;292.9)	1210.5 (998.6;1457.5)	141.1 (116.3;169)	-45.6 (-56.4;-32.5)
Roraima	176.6 (151.6;207.6)	247.4 (214.1;288.5)	343.6 (283.1;418.6)	147.7 (122.5;178.1)	-40.3 (-52.6;-26)
Santa Catarina	4396 (3777.2;4803.4)	262.9 (223.5;286.9)	4927.3 (4405.4;5845.1)	129.8 (117;151.9)	-50.6 (-57.1;-36.4)
São Paulo	41079.4 (30860;45135.8)	307.4 (238.8;335.9)	38707.2 (35088.4;43735.2)	159.6 (144.9;178.4)	-48.1 (-54.5;-31.5)
Sergipe	1395.2 (1215.1;1623.4)	235.9 (209.6;267.2)	1648.9 (1489.4;1907.3)	153.3 (138.8;176.8)	-35 (-44.4;-23.1)
Tocantins	946.1 (683.9;1191.2)	257.4 (195.8;315.3)	1217 (1071.4;1397.6)	157.3 (138.6;181.4)	-38.9 (-51.6;-20.1)

*Source: Global Burden of Disease Study 2017, Institute for Health Metrics and Evaluation.^109^*

**Table 2-8:** – Number of DALYs and age-standardized DALY rates (per 100 000) for stroke and stroke subtypes for women, in Brazil and its Federative Units, 1990 and 2017, and percent change

Cause of death and location	1990		2017		Percent change (95% UI)
	Number (95% UI)	Rate (95% UI)	Number (95% UI)	Rate (95% UI)	
***B.2.3- Stroke***					
Acre	1767.8 (1667.5;1870.6)	1862.6 (1754.2;1968.1)	3210.2 (2987;3441.9)	1026.8 (954.3;1102.8)	-44.9 (-49.1;-40.5)
Alagoas	20195.9 (18814.5;21710)	2493.7 (2346.1;2633.1)	24797.1 (23207.9;26570.3)	1438.5 (1347.2;1540)	-42.3 (-46.8;-36.9)
Amapá	885.9 (829.3;941.5)	1535.4 (1426.6;1629.7)	2656 (2406.8;2886.7)	998.8 (904.3;1088)	-34.9 (-40.6;-29)
Amazonas	8123.3 (7479.5;8682.2)	1784.6 (1640.3;1906.5)	14351.6 (13285.1;15406.9)	1007.4 (932.8;1079.1)	-43.5 (-48.1;-38.3)
Bahia	77106.2 (71098.5;83950.6)	1933.3 (1780.5;2103.5)	89411.6 (83626.9;95362.2)	1033.8 (966.7;1102)	-46.5 (-51.4;-41.3)
Brazil	1107653.4 (1072336.2;1140018.5)	2162.9 (2095.1;2225.5)	1228780.5 (1175631.6;1283687.7)	990.7 (947.7;1034.7)	-54.2 (-55.9;-52.7)
Ceará	37337.5 (33441.1;40681.5)	1555.4 (1404.6;1695.6)	51423.9 (47690.4;54659.7)	951.5 (881.9;1012.3)	-38.8 (-44.5;-32.1)
Distrito Federal	8067.4 (7639.3;8468)	2039.3 (1936;2134.4)	10665.5 (9807.7;11556.2)	770.8 (709.6;836.4)	-62.2 (-65.4;-58.8)
Espírito Santo	21284.7 (20412.8;22164.8)	2644.9 (2544.4;2750.1)	21595.1 (20097.5;23121.8)	941.9 (877;1008)	-64.4 (-67;-61.7)
Goiás	23755.9 (22547.1;25022.6)	2280.3 (2172.2;2397.1)	30858.1 (28577.4;33385.6)	870.2 (806.8;940.7)	-61.8 (-64.7;-58.9)
Maranhão	26645.2 (23289;30127.1)	1598.1 (1391.9;1827.3)	38732.2 (35890.3;42214.2)	1118.5 (1036;1221.3)	-30 (-37.8;-20.8)
Mato Grosso	7767.3 (7044.7;8529.5)	1700 (1544;1865.8)	14325.6 (13207.9;15664.7)	918.2 (846.6;1003.1)	-46 (-52.2;-38.9)
Mato Grosso do Sul	10877.7 (10222.6;11460.6)	2195.2 (2083.8;2306.8)	15366.3 (14302.6;16510.3)	1046.1 (973.5;1122.9)	-52.3 (-56.3;-48.4)
Minas Gerais	123754.1 (117958.9;129630.2)	2196.3 (2102.1;2298.1)	127819.1 (119352.2;136487.9)	939 (876.7;1002.2)	-57.2 (-60.1;-54.4)
Pará	25165.5 (23222.5;26905.8)	2068.3 (1909.5;2211.6)	37338 (34648.9;39990.2)	1071.3 (992.2;1149.1)	-48.2 (-52.7;-43.4)
Paraíba	22951.4 (21103.1;24889.3)	1790 (1649;1937.3)	27800.7 (25165.5;30858.9)	1093.1 (988.4;1214.1)	-38.9 (-45.7;-30.9)
Paraná	68876 (65849.9;71792.3)	2694.2 (2581.2;2801.1)	70346.7 (65904.8;75129.8)	1027.9 (964.2;1097.5)	-61.8 (-64.3;-59.1)
Pernambuco	65476.7 (62212.1;68590.4)	2548.1 (2424;2666.7)	66471.3 (61893.2;70853.2)	1189.6 (1108.5;1266.9)	-53.3 (-56.3;-50)
Piauí	14762.7 (13259.2;16152.3)	1745.7 (1576.7;1913.4)	21016.8 (19347.1;23129.4)	1080.9 (996;1188.6)	-38.1 (-44;-30.8)
Rio de Janeiro	163177.5 (155826.3;170272.8)	2857.2 (2732.3;2972.6)	135969.3 (127343.8;144657.3)	1113.5 (1043.6;1182.9)	-61 (-63.6;-58.3)
Rio Grande do Norte	12237.7 (11243.8;13264.5)	1354.1 (1243.1;1464.4)	16462.8 (15243.4;17811.1)	800.9 (741;867.3)	-40.9 (-46.3;-34.5)
Rio Grande do Sul	82214.5 (78371.8;85849.8)	2219.2 (2112.8;2314.9)	84773.5 (78795.6;90367.8)	1020.9 (950.7;1088.9)	-54 (-57.2;-50.6)
Rondônia	4807.8 (4391.3;5259.5)	2385 (2193.9;2590.8)	7762.8 (6762.2;8807.3)	1058.4 (926.2;1198.1)	-55.6 (-62.1;-48.7)
Roraima	619.9 (547.3;696.3)	2014.6 (1792.5;2238)	1500.1 (1285.1;1737.4)	914.8 (787.5;1048.3)	-54.6 (-62;-45.2)
Santa Catarina	32138 (30574;33582.2)	2345.2 (2228.7;2447.9)	34801.4 (32383.6;37454.8)	846.2 (788.9;910.2)	-63.9 (-66.6;-61.2)
São Paulo	233492.6 (222621.3;243756.9)	2013.9 (1920.5;2105.9)	257929.8 (239779.5;274998.1)	894 (832.2;952.5)	-55.6 (-58.7;-52.6)
Sergipe	9537.5 (8911.3;10080.5)	1935.3 (1815.6;2042.8)	13345 (12402.1;14314.6)	1112.9 (1034.8;1191.9)	-42.5 (-47.1;-37.1)
Tocantins	4626.6 (3885.4;5271.5)	2097.4 (1835.7;2348.9)	8049.8 (7349.6;8763.6)	1151.2 (1051.9;1253.8)	-45.1 (-52.1;-36.8)
***B.2.3.1- Ischemic stroke***					
Acre	444.9 (409.5;479.3)	608.7 (561.8;654.7)	902.7 (811.5;996.9)	326.8 (294.5;360.3)	-46.3 (-51.5;-40.3)
Alagoas	6023.2 (5547.1;6516)	856.7 (788.5;926.5)	7766.5 (7055.1;8495.2)	470.6 (428;514.9)	-45.1 (-50.8;-38.9)
Amapá	265.9 (242.6;289.3)	581.6 (530;629.3)	765.2 (674.4;854.7)	336.3 (297;375.7)	-42.2 (-48.1;-35.8)
Amazonas	2290.6 (2070;2488.4)	624 (566.8;677.8)	4150 (3727.4;4582.2)	329.5 (295.8;363.5)	-47.2 (-52.5;-41.2)
Bahia	22713.7 (20772.4;24878.4)	623.8 (569.8;683.6)	27251.9 (24803.3;29866.6)	310.7 (282.1;341)	-50.2 (-55.4;-44.5)
Brazil	331981.3 (316368.4;347711)	747.7 (713.7;780.1)	401935.6 (371989.2;433351)	326.3 (302.2;351.8)	-56.4 (-58.8;-54.1)
Ceará	10460.7 (9309.4;11614.4)	483.2 (430.3;536)	17418.5 (15794.8;18990.5)	315.7 (285.6;344.3)	-34.7 (-42.2;-25.5)
Distrito Federal	1824.5 (1676.1;1966.4)	685.8 (636.7;737.7)	3106.5 (2760.9;3501.9)	266.2 (237.8;298.3)	-61.2 (-65.4;-56.8)
Espírito Santo	6145.4 (5729.6;6600.7)	927.7 (871.2;989.9)	6859.1 (6208.3;7536.9)	304 (275.3;334.3)	-67.2 (-70.2;-63.9)
Goiás	6143.4 (5644.8;6602.7)	804.8 (746.4;862.9)	9041.8 (8039.9;10070.3)	277 (247.5;307)	-65.6 (-69;-61.8)
Maranhão	6015.4 (5053;7099.9)	444.2 (367.5;530.1)	10822.4 (9615.6;12156.4)	324.4 (288.3;364.7)	-27 (-37.1;-14.1)
Mato Grosso	1936.9 (1730.7;2144.1)	579.9 (519.9;639.6)	4094.2 (3653.7;4555.7)	300.5 (269;333.6)	-48.2 (-54.3;-40.9)
Mato Grosso do Sul	2719.9 (2519.2;2923.8)	696.2 (644.8;746.8)	4484.7 (4031.8;4957.1)	324.5 (292.4;358.5)	-53.4 (-58.3;-48.2)
Minas Gerais	35992.5 (33442.6;38488.3)	745.3 (697.4;793.9)	41120.2 (37036.1;45138.4)	296.9 (267;326.4)	-60.2 (-63.8;-56.4)
Pará	8549.8 (7800.8;9221)	821.7 (750.3;883.5)	11848 (10668.4;13072.2)	370.9 (334.4;408.8)	-54.9 (-60;-49.8)
Paraíba	6790.1 (6100.7;7542.4)	562.3 (505.8;621.7)	9145.2 (8214.6;10210.5)	345.4 (310;386.5)	-38.6 (-46.9;-30)
Paraná	21328.3 (19837.5;22825.2)	1018.3 (954.7;1085.5)	25351.3 (22978.4;27818.6)	381.4 (346;418.7)	-62.5 (-65.9;-58.8)
Pernambuco	20813.8 (19337.3;22311.5)	897.4 (835.9;959.7)	20486 (18571.5;22484.9)	371.3 (336.9;407.6)	-58.6 (-62.6;-54.3)
Piauí	3806.7 (3359.2;4298.2)	518 (458.2;583.8)	6861.3 (6175.8;7614.2)	348.2 (313.2;386.4)	-32.8 (-40.7;-24)
Rio de Janeiro	45951.2 (42906.6;49127.3)	911.1 (852.2;970.1)	42263.4 (38418.9;46502.1)	336.1 (305.4;369.6)	-63.1 (-66.4;-59.5)
Rio Grande do Norte	4389.4 (3954;4826.9)	516.8 (465.8;567.9)	5556.1 (4975.4;6161.1)	265.8 (237.2;294.6)	-48.6 (-54.5;-41.7)
Rio Grande do Sul	29458.4 (27295.1;31550.4)	895.7 (833.5;953.9)	31776.4 (28691.4;34814.6)	367.6 (331.6;403.4)	-59 (-62.7;-55.1)
Rondônia	1058.6 (955.1;1174.3)	804 (731.9;882.3)	2282.1 (1981;2588.8)	357.4 (311.9;404)	-55.5 (-62.1;-48.7)
Roraima	140 (122.6;157.4)	739.9 (656.3;827)	433 (371.9;499.1)	331.8 (287.4;379.6)	-55.2 (-62.2;-46.2)
Santa Catarina	10349.1 (9571.8;11121)	910.1 (847;972.5)	12306.8 (11098;13645.2)	310.4 (281;342.7)	-65.9 (-69.2;-62.4)
São Paulo	72124.2 (66732.6;77418.9)	733.7 (681.9;784.3)	89212.1 (80399.2;97949)	310.1 (280.1;340.3)	-57.7 (-61.6;-53.7)
Sergipe	3143.7 (2870.4;3420.7)	683.8 (624.9;743.8)	4299.2 (3883.7;4707.7)	372 (335.7;406.8)	-45.6 (-51.6;-39.2)
Tocantins	1101.1 (958.3;1252.5)	685.3 (606.7;768.8)	2331.2 (2078.4;2585.9)	360.1 (321.8;399.9)	-47.5 (-54.6;-39.4)
***B.2.3.2- Intracerebral hemorrhage***					
Acre	874.7 (812.9;936.3)	923.1 (856.1;989.3)	1599.2 (1461.9;1744.8)	505.7 (461;552.2)	-45.2 (-51.2;-39.3)
Alagoas	9872.6 (9062.5;10730.1)	1226.4 (1127.7;1322.2)	12763.3 (11791.5;13850.8)	733.8 (676.7;796.4)	-40.2 (-46.2;-33.1)
Amapá	439.7 (406.5;470)	729.9 (668.2;779.6)	1319.2 (1165.7;1460.5)	485.9 (429.7;538.1)	-33.4 (-40.7;-24.9)
Amazonas	4241.5 (3841.4;4559.2)	918 (830.8;989.4)	7271.3 (6627.5;7883.3)	503.5 (459.3;545.9)	-45.1 (-50.5;-38.7)
Bahia	39495.1 (36010.5;43294.5)	993.4 (903.6;1090.2)	44388.6 (40962.7;47759)	515.9 (475.7;555.3)	-48.1 (-53.8;-42.1)
Brazil	563999.7 (545387.7;584682.8)	1070.2 (1035.3;1106.6)	580172.8 (555092.6;606176.7)	465.2 (445;485.9)	-56.5 (-58.7;-54.5)
Ceará	18233.6 (16299.5;20228.9)	772.8 (688.7;861.4)	24338.9 (22304.9;26159.7)	453.1 (415.4;487.5)	-41.4 (-48.8;-33.2)
Distrito Federal	4240.9 (3979.9;4508.9)	1005.6 (949;1069.6)	4945.6 (4453.2;5505.7)	345.3 (314.1;384.1)	-65.7 (-69.4;-61.7)
Espírito Santo	11142.1 (10603;11788.2)	1321.2 (1257.8;1394.8)	10272.9 (9453.4;11232.8)	445.3 (409.9;487.5)	-66.3 (-69.3;-63.3)
Goiás	12855.7 (12049.1;13698)	1147.9 (1079.5;1224.7)	15021.7 (13681.7;16488.2)	415.3 (379.8;455)	-63.8 (-67.3;-60.4)
Maranhão	12042.9 (9978.3;14209.6)	765.5 (599.9;923.2)	19249 (17310;21828.7)	557.1 (499.7;633.4)	-27.2 (-37.8;-11.3)
Mato Grosso	3853.5 (3437.3;4288.6)	817 (728.9;905.6)	6957.4 (6329.9;7833.2)	433.6 (394.8;486.6)	-46.9 (-54.1;-38.6)
Mato Grosso do Sul	5943.8 (5561.7;6313.3)	1148.3 (1080.5;1215)	7653.1 (7025.6;8338.4)	513.4 (471.2;560.2)	-55.3 (-59.8;-50.7)
Minas Gerais	64366 (60674.3;68220.4)	1099.1 (1037.2;1163.7)	60695 (56080.7;65783.6)	445.7 (411.9;483)	-59.4 (-63;-55.4)
Pará	12175.4 (11139.9;13184)	978.4 (892.1;1059.1)	18001 (16375.4;19576.3)	509.9 (464;555.1)	-47.9 (-54;-41.2)
Paraíba	11111.2 (10082.5;12219.4)	868.2 (787.5;953.5)	13504.5 (12040;15189.3)	535.8 (476.9;602.6)	-38.3 (-46.7;-28.4)
Paraná	35261.9 (33355.6;37203.2)	1301.5 (1228.2;1375.8)	31095.5 (28671.9;33664.6)	447.4 (412.9;484.6)	-65.6 (-68.7;-62.4)
Pernambuco	35167.1 (32910.5;37242.7)	1332.3 (1249;1406.7)	33631.6 (31062.3;36327.8)	599.2 (553.1;646.5)	-55 (-59;-50.7)
Piauí	7420.7 (6473.6;8346.5)	888.3 (775.7;998.5)	10247.3 (9366.6;11531.5)	529 (483;595.7)	-40.4 (-47.8;-31.4)
Rio de Janeiro	88936.8 (84337.9;93896)	1501.8 (1423.9;1582.9)	68013.5 (62977;73431)	555.9 (515.3;599.1)	-63 (-66.3;-59.6)
Rio Grande do Norte	5639.4 (5056.1;6175)	626.4 (561.9;686.3)	7840.9 (7108.5;8599.3)	383.7 (347.4;421.2)	-38.7 (-46.2;-29.8)
Rio Grande do Sul	40495.5 (37779.2;42885.8)	1036.9 (967.6;1098.7)	39133.7 (35903.4;42395.5)	472.7 (432.7;511.1)	-54.4 (-58.6;-49.5)
Rondônia	2628.7 (2371.6;2911.8)	1227.9 (1117.7;1354.8)	3779.6 (3245.4;4369.3)	501 (433.1;579.1)	-59.2 (-65.6;-51.6)
Roraima	328.6 (286.2;373.3)	992.7 (870.9;1116.1)	736.9 (617.6;867)	430 (361.4;504.1)	-56.7 (-64.8;-46.4)
Santa Catarina	16220.7 (15245.9;17192.1)	1118.6 (1052.1;1188.3)	15768.8 (14405.3;17123)	377.2 (345.2;409.4)	-66.3 (-69.7;-62.9)
São Paulo	113907.8 (107421.3;120928.2)	942.3 (886.5;1000.5)	111325 (103027.9;120702.2)	383.3 (354.6;414.6)	-59.3 (-63;-55.4)
Sergipe	4704.6 (4381.8;5022.3)	963.7 (897.4;1029.7)	6663.5 (6108.6;7225.7)	552.1 (505.7;600.2)	-42.7 (-48.5;-35.7)
Tocantins	2399.1 (1989.3;2787)	1052.1 (902.5;1202.8)	3955.6 (3568.2;4425.3)	560.4 (506.6;627.1)	-46.7 (-54.7;-36.9)
***B.2.3.3- Subarachnoid hemorrhage***					
Acre	448.2 (407.7;497.6)	330.8 (302.6;363.3)	708.2 (638.6;792.2)	194.2 (174.9;216.9)	-41.3 (-48.2;-34)
Alagoas	4300.1 (3589.8;5036.8)	410.6 (351;468.2)	4267.3 (3820.8;4772.1)	234.1 (208.7;261.8)	-43 (-52.3;-29.4)
Amapá	180.3 (163.7;201.9)	223.8 (202.9;252.3)	571.6 (511;643.2)	176.7 (158;198.7)	-21.1 (-31.2;-9.9)
Amazonas	1591.2 (1418;1789.1)	242.6 (217.8;270.9)	2930.3 (2642.6;3273.2)	174.4 (156.1;192.6)	-28.1 (-37.5;-18.8)
Bahia	14897.5 (13287.5;16507.2)	316.1 (283.5;351.9)	17771.1 (16139.9;19611.7)	207.1 (188.6;228.5)	-34.5 (-42.6;-24.3)
Brazil	211672.4 (199208.9;221899.8)	345.1 (324.7;363.2)	246672.1 (231937.6;260803.4)	199.2 (187.4;210.1)	-42.3 (-46.1;-39)
Ceará	8643.3 (6852.4;10486.6)	299.4 (244.9;357.4)	9666.5 (8677.7;10600.5)	182.6 (164.3;200.1)	-39 (-50.4;-26.6)
Distrito Federal	2002 (1803;2168.1)	347.8 (316.2;375.5)	2613.4 (2319;2911.4)	159.4 (141;177.5)	-54.2 (-59.3;-47.8)
Espírito Santo	3997.2 (3523.8;4296.8)	396 (345.6;425)	4463.2 (4009.2;4908.2)	192.6 (173.2;211.2)	-51.4 (-56.9;-43.7)
Goiás	4756.8 (4346.9;5221.2)	327.6 (300.5;359.9)	6794.6 (6134.7;7577.9)	177.8 (160.7;197.9)	-45.7 (-51.6;-38.3)
Maranhão	8586.9 (6391.4;11138.3)	388.5 (299.2;491.9)	8660.9 (7731.3;9594.6)	237 (211.2;263.3)	-39 (-51.4;-23.6)
Mato Grosso	1976.9 (1748.4;2227.8)	303.1 (269.1;341.7)	3274 (2926.5;3658.6)	184.1 (165.4;204.9)	-39.2 (-47.7;-29.8)
Mato Grosso do Sul	2214 (1954.7;2430)	350.7 (315.4;383.4)	3228.6 (2883.8;3619.9)	208.3 (186.4;233)	-40.6 (-48.1;-31.7)
Minas Gerais	23395.6 (21454;25318.2)	351.9 (322.6;381.9)	26003.9 (23613.8;28559.9)	196.4 (178.8;214.9)	-44.2 (-50;-37.7)
Pará	4440.4 (3942.4;4957.1)	268.2 (240.2;299.8)	7488.9 (6775.2;8285.9)	190.5 (171.6;211.1)	-29 (-37.1;-19.3)
Paraíba	5050.1 (4155.5;5968.1)	359.5 (295.4;422.2)	5151 (4436.2;5838.5)	211.9 (182.7;240.2)	-41.1 (-53;-27.7)
Paraná	12285.8 (11347.4;13264.7)	374.4 (343.4;405.7)	13900 (12510.5;15373.5)	199.1 (179.6;219.1)	-46.8 (-53;-40.1)
Pernambuco	9495.7 (8636;10672.6)	318.4 (290.2;361.1)	12353.7 (11186.9;13775.6)	219.1 (198.7;243.7)	-31.2 (-39.6;-22.6)
Piauí	3535.2 (2860;4197.6)	339.4 (278.5;403.6)	3908.2 (3521;4310.2)	203.7 (183.8;224.5)	-40 (-49.9;-27.8)
Rio de Janeiro	28289.5 (25607.4;30525.9)	444.3 (403.8;479.4)	25692.3 (23122.1;28299.6)	221.6 (200.2;244.4)	-50.1 (-55.6;-43.3)
Rio Grande do Norte	2208.9 (1905.2;2660.8)	210.9 (182.9;259.7)	3065.8 (2734.3;3771.2)	151.5 (135.3;186)	-28.2 (-39.4;-16.6)
Rio Grande do Sul	12260.7 (11247.3;14498.1)	286.6 (262.9;339.3)	13863.3 (12280.1;15559.9)	180.6 (160.3;202.5)	-37 (-44.8;-29)
Rondônia	1120.6 (1002.6;1250.6)	353.1 (315.9;395.2)	1701.2 (1439.6;1988.5)	200 (169.9;234.3)	-43.4 (-53.1;-32.1)
Roraima	151.2 (130.8;181.3)	282 (242.8;345.6)	330.2 (273;406.4)	153 (127.5;188.8)	-45.8 (-56.1;-32.4)
Santa Catarina	5568.2 (5137.7;6024.6)	316.5 (290.8;342.4)	6725.8 (6071.4;7486.2)	158.6 (143.3;176.2)	-49.9 (-55.5;-43.4)
São Paulo	47460.6 (43883.2;51172.3)	337.9 (312.7;365.3)	57392.7 (51539.2;62558)	200.5 (180.5;218)	-40.7 (-47.3;-33.9)
Sergipe	1689.1 (1486.9;1900.9)	287.8 (256.9;325.8)	2382.3 (2149.2;2638.5)	188.8 (170.3;210.1)	-34.4 (-43.9;-25.1)
Tocantins	1126.4 (839.2;1386.1)	360 (280.2;438.7)	1763.1 (1534.2;1962.8)	230.6 (201.3;256.2)	-35.9 (-48.5;-18.3)

*Source: Global Burden of Disease Study 2017, Institute for Health Metrics and Evaluation.^109^*

**Table 2-9:** – Age-standardized DALY rates (per 100 000) for stroke and stroke subtypes, for men, in Brazil, 1990 and 2017, and percent change

Cause of death and age group	1990	2017	Percent change (95% UI)
***B.2.3- Stroke***			
15-49 years	986.5 (948.5;1024.5)	432.8 (415.2;452.5)	-56.1 (-58.4;-53.6)
50-69 years	7867.2 (7612.7;8119.2)	3538 (3397.9;3689.8)	-55 (-56.9;-53.1)
5-14 years	83.1 (69.7;91.1)	35.7 (31.8;39.7)	-57.1 (-62.4;-50.4)
70+ years	17208.4 (16711.1;17728.9)	9407.6 (9006.8;9773.9)	-45.3 (-47.4;-43.3)
Age-standardized	2901.8 (2824.3;2983.3)	1334.5 (1289.5;1381.5)	-54 (-55.4;-52.5)
All Ages	1814.8 (1761.9;1869.6)	1319.5 (1275.2;1367.3)	-27.3 (-29.6;-24.7)
Under 5	410.2 (325.6;536.1)	70.8 (58.7;84.7)	-82.8 (-87.9;-75.9)
***B.2.3.1- Ischemic stroke***			
15-49 years	146.5 (137.3;158.8)	66.3 (60;73.2)	-54.7 (-59.7;-50.7)
50-69 years	2283.9 (2165.8;2401.8)	952.4 (887.3;1020)	-58.3 (-61.1;-55.6)
5-14 years	10.9 (8.9;13.6)	4.2 (3.3;5.5)	-61.2 (-66.6;-55.8)
70+ years	8926.8 (8590.6;9283.7)	4660.5 (4394.5;4925)	-47.8 (-50.4;-45.3)
Age-standardized	1011 (973.7;1050.4)	463.4 (436.9;490.2)	-54.2 (-56.5;-52.2)
All Ages	541.4 (520.1;565.3)	429.6 (404.9;455.5)	-20.6 (-25;-17)
Under 5	24.6 (16.8;34.3)	3 (2.2;3.9)	-87.6 (-93.3;-78.5)
***B.2.3.2- Intracerebral hemorrhage***			
15-49 years	644.8 (614.9;701.4)	257.6 (241.6;271.2)	-60.1 (-64.4;-57.1)
50-69 years	4886.7 (4702.7;5077.9)	2143 (2055.1;2228.8)	-56.1 (-58.2;-54)
5-14 years	25 (20.4;28.9)	10 (8.2;11.8)	-60.1 (-69.4;-49.4)
70+ years	7674.2 (7361.5;7956.2)	4238.2 (4048.2;4415.7)	-44.8 (-47.4;-41.9)
Age-standardized	1601 (1552.2;1665.3)	709.9 (684.6;733.7)	-55.7 (-57.5;-53.9)
All Ages	1042.8 (1008.5;1093)	721.4 (695.9;745.7)	-30.8 (-34.2;-27.8)
Under 5	108.9 (61.6;169)	12.1 (8.8;16)	-88.9 (-94.1;-76.9)
***B.2.3.3- Subarachnoid hemorrhage***			
15-49 years	195.1 (150.6;208.4)	108.9 (102;128.8)	-44.2 (-49.5;-22.9)
50-69 years	696.6 (566.1;745.5)	442.6 (414.3;479.7)	-36.5 (-41.7;-23.2)
5-14 years	47.2 (39.3;52.5)	21.4 (18.9;24.2)	-54.6 (-60.4;-44.4)
70+ years	607.4 (558;657.4)	508.9 (436.7;551.9)	-16.2 (-24.4;-7.6)
Age-standardized	289.9 (237.9;307.2)	161.2 (152.9;175.4)	-44.4 (-48.2;-31.2)
All Ages	230.7 (186.5;246.2)	168.5 (160;184.4)	-26.9 (-32.5;-7.7)
Under 5	276.7 (203.1;344.3)	55.7 (46.8;65.9)	-79.9 (-85.1;-70.4)

*Source: Global Burden of Disease Study 2017, Institute for Health Metrics and Evaluation.^109^*

**Chart 2-3:** Data derived from the Global Burden of Disease Study 2017 (GBD 2017).^109^

**Chart 3-1:** Data derived from Global Burden of Disease Study 2017 (GBD 2017).^151^

**Chart 3-2:** Data derived from Global Burden of Disease Study 2017 (GBD 2017).^151^

**Chart 3-3:** Data derived from Global Burden of Disease Study 2017 (GBD 2017).^151^

**Chart 3-4:** Data derived from Global Burden of Disease Study 2017 (GBD 2017).^151^

**Chart 3-5:** Data derived from DATASUS.^152^

**Chart 3-6:** Data derived from Global Burden of Disease Study 2017 (GBD 2017).^151^

**Chart 4-1:** Data derived from Global Burden of Disease Study 2017 (GBD 2017).^188^

**Chart 4-2:** Data derived from Global Burden of Disease Study 2017 (GBD 2017).^188^

**Table 4-2:** – Number of incident cases and age-standardized incidence rate (per 100 000) of cardiomyopathy and myocarditis, and percent change of rates, in Brazil and its Federative Units, 1990 and 2017

	1990		2017		Percent change (95% UI)
	Number (95% UI)	Rate (95% UI)	Number (95% UI)	Rate (95% UI)	
Brazil	54520.4 (48574.3;61320.7)	46.3 (41.5;52.1)	103879.4 (92495.6;117294.5)	46.7 (41.8;52.6)	0.8 (-0.3;1.8)
Acre	125.8 (111;143)	44.8 (40;50.7)	340.7 (303.3;383.1)	46 (41.1;52)	2.8 (-0.5;6.4)
Alagoas	826.5 (732.4;929.4)	43.6 (38.8;49.3)	1551.4 (1378.8;1749)	46.5 (41.5;52.4)	6.8 (3.5;10.2)
Amapá	85.4 (75.2;97.2)	47.5 (42.5;53.4)	311.1 (274.3;352.6)	47.6 (42.5;53.6)	0.3 (-2.8;3.9)
Amazonas	645.3 (565.6;732.6)	46.4 (41.5;52.6)	1586.5 (1410.7;1793.3)	46.6 (41.6;52.8)	0.5 (-2.9;4.3)
Bahia	4183 (3713.7;4708.9)	45.3 (40.3;50.9)	7177 (6366.6;8059.8)	45.1 (40;50.7)	-0.3 (-3.4;2.7)
Ceará	2232.8 (1990.7;2511.3)	43.2 (38.7;48.7)	4589.2 (4084.6;5197.2)	46.4 (41.4;52.7)	7.5 (3.4;11)
Distrito Federal	502.7 (439.4;574.4)	45.2 (40.3;51.3)	1258.3 (1107.5;1428.3)	45.3 (40.4;51)	0.2 (-3.3;3.7)
Espírito Santo	976.1 (856.5;1104.3)	48.1 (42.8;54.4)	1992.5 (1757;2263.2)	48.2 (42.9;54.4)	0.2 (-3.2;3.8)
Goiás	1391.1 (1220.9;1574.2)	46.3 (41.4;52.2)	3233.1 (2869.8;3671.6)	47.3 (42.2;53.5)	2.3 (-0.8;5.4)
Maranhão	1568.5 (1390.4;1771.6)	43.3 (38.6;48.9)	3314.6 (2958.6;3716.8)	46.7 (41.7;52.6)	8 (4.5;12)
Mato Grosso	628.1 (549.8;715.5)	45.6 (40.7;51.7)	1567.2 (1394.8;1780.2)	46.8 (41.9;52.9)	2.7 (-1;6.3)
Mato Grosso do Sul	648.6 (572.4;728)	48.6 (43.4;54.8)	1426.5 (1264.1;1616.6)	50.6 (45.2;57)	4.1 (0.8;7.7)
Minas Gerais	5880.7 (5207.1;6646.3)	46 (41;52)	11181 (10003.5;12646.6)	47 (42.2;53.2)	2.2 (-1;5.5)
Pará	1548.2 (1365.8;1749.4)	45.4 (40.6;51.3)	3561 (3155.4;4003.7)	45.5 (40.5;51.4)	0.2 (-3.2;3.9)
Paraíba	1181.3 (1051.4;1331.4)	43.2 (38.6;48.9)	2068.4 (1841.2;2347.2)	46.3 (41.2;52.5)	7.1 (3.6;10.5)
Paraná	3140.9 (2777.5;3572.6)	47.1 (41.9;53.3)	5728 (5060.1;6535.4)	47 (41.9;53.2)	-0.2 (-3.8;3)
Pernambuco	2639 (2333.6;2961.8)	45.6 (40.5;51.3)	4609.7 (4098.5;5160.5)	46.1 (41.1;51.6)	1.1 (-2.4;4.5)
Piauí	865.4 (765.7;974.5)	43.8 (39;49.4)	1652.7 (1471;1863.8)	45.8 (40.7;51.6)	4.5 (0.8;8.3)
Rio de Janeiro	5321.9 (4706.8;6019.7)	46.7 (41.6;52.5)	9236.1 (8183.4;10524.9)	46.4 (41.3;52.5)	-0.5 (-4.1;3.1)
Rio Grande do Norte	886.2 (790.4;995.2)	44.3 (39.7;49.8)	1723.7 (1535.1;1947.9)	46.2 (41.2;52.1)	4.2 (1;7.7)
Rio Grande do Sul	3927.6 (3482.4;4424.5)	49.5 (44.1;55.8)	6475.7 (5749;7325.7)	48.5 (43.2;54.5)	-2.1 (-5.5;1.4)
Rondônia	330.5 (289.3;379.1)	45.6 (40.6;51.4)	746.3 (660.9;847.2)	46 (41.2;52.2)	0.9 (-2.6;4.4)
Roraima	61.2 (53.3;70.9)	46.1 (41.2;52)	212.3 (187.3;240.4)	46.4 (41.3;52.4)	0.9 (-2.4;4.2)
Santa Catarina	1694 (1503.4;1912.2)	47.8 (42.9;54.2)	3554.7 (3148.4;4033.5)	47.5 (42.4;53.4)	-0.7 (-4.2;3.5)
São Paulo	12421.8 (10980.5;13984.7)	47.6 (42.4;53.5)	22999.9 (20352.4;26154.9)	46.6 (41.6;52.7)	-1.9 (-5.5;1.4)
Sergipe	520.8 (463.6;584.1)	45.3 (40.5;51.4)	1075.4 (957.1;1218.6)	47.3 (42.1;53.4)	4.5 (1;7.8)
Tocantins	287 (253.9;325.8)	44.8 (40;50.5)	706.6 (629;798)	47.1 (42.1;53.1)	5.1 (1.6;8.9)

*Source: Global Burden of Disease Study 2017, Institute for Health Metrics and Evaluation.^188^*

**Table 4-8:** – Number of deaths and age-standardized mortality rate per 100 000 inhabitants due to Chagas disease, in 1990 and 2017, and percent change of rates, in Brazil and its Federative Units

Location	1990		2017		Percent change (95% UI)
	Number of deaths (95% UI)	Mortality Rate (95% UI)	Number of deaths (95% UI)	Mortality Rate (95% UI)	
Acre	1.8 (1.6;1.9)	0.9 (0.9;1)	2.1 (1.8;2.4)	0.3 (0.3;0.4)	-63.9 (-69.7;-56.2)
Alagoas	68.4 (54.3;78.8)	4.8 (3.8;5.5)	79.9 (72;90.2)	2.5 (2.3;2.9)	-46.6 (-56.5;-28.3)
Amapá	0.5 (0.5;0.5)	0.5 (0.4;0.5)	1 (0.9;1.1)	0.2 (0.2;0.2)	-58.2 (-63.9;-52)
Amazonas	3.8 (3.5;4.1)	0.4 (0.4;0.5)	4.6 (4.1;5.5)	0.2 (0.1;0.2)	-61.3 (-66.6;-53.6)
Bahia	682.2 (629.5;736.3)	9.2 (8.5;9.9)	788.1 (728;871.6)	5 (4.6;5.5)	-45.7 (-51.7;-39.2)
Brazil	7049.3 (6816.6;7323.9)	7.3 (7;7.6)	5493.6 (5221.2;6014.7)	2.5 (2.3;2.7)	-66.3 (-68.3;-63.5)
Ceará	44.2 (36.7;55.8)	1 (0.9;1.3)	53.8 (48.3;61.5)	0.5 (0.5;0.6)	-47.6 (-59.4;-34.1)
Distrito Federal	235.6 (223.8;249.1)	34.9 (33.1;36.8)	211 (191.3;248.7)	9.7 (8.8;11.5)	-72.2 (-74.9;-67.6)
Espírito Santo	10.7 (9.9;11.6)	0.7 (0.7;0.8)	9.2 (8.2;10.9)	0.2 (0.2;0.3)	-69.8 (-73.4;-64.5)
Goiás	1131.6 (1067;1212.8)	55.2 (51.9;59.4)	815.2 (753.8;917.7)	13 (12;14.6)	-76.4 (-78.2;-74.2)
Maranhão	15.7 (12.4;19.5)	0.6 (0.5;0.7)	17.5 (15.5;20.8)	0.3 (0.2;0.3)	-52.4 (-61.3;-32.7)
Mato Grosso	39.6 (35.8;44.1)	4.4 (4;4.9)	57 (51.1;68.5)	1.8 (1.7;2.2)	-58.3 (-63.6;-50.8)
Mato Grosso do Sul	55 (50.5;59.3)	5.7 (5.3;6.1)	53 (47.6;60.1)	1.9 (1.7;2.2)	-66.4 (-70.5;-61.6)
Minas Gerais	2195.4 (2095;2316.9)	21 (20;22.1)	1390.3 (1294.6;1519.6)	5.5 (5.1;6)	-73.6 (-75.7;-71.4)
Pará	24.1 (21.9;26.3)	1 (1;1.1)	29.1 (25.4;35.6)	0.4 (0.4;0.5)	-58.3 (-64.6;-48.2)
Paraíba	35.3 (27.2;42.3)	1.5 (1.2;1.8)	38.3 (34.3;42.9)	0.8 (0.7;0.9)	-45.1 (-57.6;-24.7)
Paraná	384.7 (365.1;406.4)	7.4 (7;7.8)	251.4 (232.2;277.9)	2 (1.9;2.2)	-72.7 (-75.1;-69.5)
Pernambuco	163.5 (149.3;175.1)	3.5 (3.2;3.8)	143.4 (130.4;157.7)	1.5 (1.3;1.6)	-58.7 (-63.5;-51.8)
Piauí	59.5 (49.7;71.3)	4.1 (3.4;4.9)	71 (64;81.2)	2 (1.8;2.3)	-51.4 (-60.2;-38.5)
Rio de Janeiro	77 (71.5;82.6)	0.8 (0.7;0.8)	58.6 (53.1;65.7)	0.3 (0.2;0.3)	-64.8 (-68.7;-60)
Rio Grande do Norte	14.7 (12.8;17.1)	0.9 (0.8;1)	16.2 (14.3;18.3)	0.4 (0.4;0.5)	-51.4 (-62.5;-39.8)
Rio Grande do Sul	61.3 (56.8;65.7)	0.9 (0.9;1)	48.7 (44.4;55.6)	0.3 (0.3;0.4)	-64.8 (-68.6;-58.9)
Rondônia	25 (22.3;27.9)	5.8 (5.2;6.4)	24.1 (20.7;28.4)	1.7 (1.4;1.9)	-71.3 (-75.8;-65.3)
Roraima	0.5 (0.5;0.6)	0.7 (0.6;0.8)	0.8 (0.6;1)	0.2 (0.2;0.3)	-69.2 (-75.4;-60.8)
Santa Catarina	13.1 (12.1;14.1)	0.5 (0.5;0.5)	12.4 (11.1;14.3)	0.2 (0.1;0.2)	-68.2 (-72.2;-62.2)
São Paulo	1653.4 (1583.9;1727.4)	7.4 (7.1;7.8)	1248.9 (1168.1;1356.1)	2.4 (2.3;2.7)	-67 (-69.2;-64)
Sergipe	10.6 (9.4;12.1)	1.2 (1.1;1.4)	11.8 (10.7;13.3)	0.5 (0.5;0.6)	-55.3 (-62.9;-44.5)
Tocantins	42.3 (34.1;52.3)	10.1 (8.4;12.3)	56.3 (50.2;64.6)	4.1 (3.6;4.7)	-59.6 (-68.4;-48.4)

*Source: Global Burden of Disease Study 2017, Institute for Health Metrics and Evaluation.^188^*

**Table 4-9:** – Number of deaths and age-standardized mortality rate for males due to Chagas disease, per 100 000 inhabitants, in 1990 and 2017, and percent change of rates, in Brazil and its Federative Units

Location	1990		2017		Percent change (95% UI)
	Number of deaths (95% UI)	Mortality Rate (95% UI)	Number of deaths (95% UI)	Mortality Rate (95% UI)	
Acre	1.3 (1.2;1.5)	1.3 (1.2;1.5)	1.4 (1.2;1.7)	0.5 (0.4;0.6)	-64.1 (-71.8;-55.1)
Alagoas	42.6 (32.8;50.5)	6.2 (4.8;7.4)	46.5 (40.2;54.3)	3.3 (2.8;3.8)	-47.3 (-58.1;-26.3)
Amapá	0.3 (0.3;0.4)	0.6 (0.6;0.7)	0.7 (0.6;0.8)	0.3 (0.2;0.3)	-57.3 (-64.9;-48)
Amazonas	2.9 (2.5;3.2)	0.6 (0.6;0.7)	3.3 (2.8;4.1)	0.2 (0.2;0.3)	-62.6 (-69.1;-53.3)
Bahia	449.9 (402.3;501.3)	12.7 (11.3;14.2)	511.7 (459;580.7)	7.2 (6.4;8.1)	-43.3 (-51.7;-33)
Brazil	4411.2 (4207.8;4651.3)	9.5 (9;10)	3094.4 (2868.4;3448.7)	3.1 (2.9;3.4)	-67.5 (-70.1;-63.8)
Ceará	30.1 (24;40.2)	1.5 (1.2;2)	34.5 (29.7;40.3)	0.8 (0.7;0.9)	-48.3 (-62;-32.1)
Distrito Federal	136.9 (127;147.8)	41.3 (38.2;44.7)	108 (93.7;132.2)	11.2 (9.7;13.7)	-72.9 (-76.6;-67.7)
Espírito Santo	7.5 (6.8;8.4)	1 (0.9;1.1)	5.9 (5.1;7.3)	0.3 (0.3;0.4)	-70.4 (-75;-63.1)
Goiás	684.1 (634.4;744.1)	60.3 (55.7;66)	453.1 (406.4;522.9)	15.1 (13.6;17.4)	-75 (-77.7;-71.7)
Maranhão	11.4 (8.5;13.6)	0.9 (0.7;1.1)	11.9 (10.3;14.3)	0.4 (0.3;0.5)	-54.2 (-63.5;-31.4)
Mato Grosso	27 (23.5;30.8)	5.5 (4.8;6.2)	36 (31.3;43.8)	2.2 (2;2.7)	-58.8 (-65.7;-49.3)
Mato Grosso do Sul	38.9 (34.9;42.8)	7.6 (6.9;8.4)	33.5 (29.3;39.5)	2.5 (2.2;3)	-66.8 (-71.9;-60.5)
Minas Gerais	1342.2 (1256.4;1447)	26.6 (24.9;28.7)	744.3 (672;833.9)	6.5 (5.9;7.3)	-75.5 (-78.1;-72.3)
Pará	17.3 (15.4;19.4)	1.5 (1.3;1.7)	20.8 (17.5;26)	0.6 (0.5;0.8)	-58 (-66.1;-47)
Paraíba	22.1 (16.8;27.9)	2 (1.5;2.5)	23 (19.9;26.6)	1.1 (1;1.3)	-43.6 (-58.9;-17.5)
Paraná	253.3 (234.6;273.2)	9.6 (8.9;10.3)	139.8 (125;159.6)	2.5 (2.2;2.8)	-74.2 (-77.5;-70.2)
Pernambuco	103.2 (92.7;113)	4.8 (4.3;5.3)	83.8 (73.8;95)	2 (1.7;2.2)	-59.4 (-65.6;-51.2)
Piauí	40.4 (33.2;49.7)	5.8 (4.8;7.2)	44.6 (39.4;52.1)	2.7 (2.4;3.1)	-53.9 (-63.5;-40.1)
Rio de Janeiro	47.4 (42.8;52.3)	1.1 (1;1.2)	31.7 (27.2;36.8)	0.3 (0.3;0.4)	-67.4 (-72.5;-61.1)
Rio Grande do Norte	10.6 (9;12.8)	1.4 (1.1;1.6)	11.3 (9.6;13.1)	0.7 (0.6;0.8)	-50.4 (-63.4;-35.3)
Rio Grande do Sul	40.4 (36.3;44.5)	1.3 (1.2;1.5)	28.9 (25.3;34.2)	0.4 (0.4;0.5)	-67.3 (-72.2;-60.5)
Rondônia	18.1 (15.5;20.7)	7.2 (6.2;8.2)	14.5 (11.8;18)	1.9 (1.6;2.4)	-73.2 (-78.8;-65.6)
Roraima	0.5 (0.4;0.5)	1.1 (0.9;1.3)	0.6 (0.5;0.8)	0.3 (0.3;0.4)	-68.4 (-76;-58.3)
Santa Catarina	8.6 (7.7;9.5)	0.7 (0.6;0.8)	7.3 (6.2;8.7)	0.2 (0.2;0.2)	-69.9 (-74.9;-62.9)
São Paulo	1039.3 (976.2;1108.4)	9.7 (9.1;10.4)	655 (592.6;739.1)	2.9 (2.7;3.3)	-69.8 (-73.2;-65.6)
Sergipe	7 (6;8.3)	1.7 (1.5;2)	7.7 (6.7;8.8)	0.8 (0.7;0.9)	-54.3 (-64.5;-42)
Tocantins	27.7 (21.6;34.8)	12 (9.6;15.1)	34.6 (29.6;40.9)	4.9 (4.2;5.7)	-59.4 (-69.7;-45.9)

*Source: Global Burden of Disease Study 2017, Institute for Health Metrics and Evaluation.^188^*

**Chart 4-3:** Data derived from Global Burden of Disease Study 2017 (GBD 2017).^188^

**Chart 4-4:** Data derived from Global Burden of Disease Study 2017 (GBD 2017).^188^

**Chart 4-5:** Data derived from Global Burden of Disease Study 2017 (GBD 2017).^188^

**Chart 4-6:** 

**Chart 4-7:** Data derived from Global Burden of Disease Study 2017 (GBD 2017).^188^

*Source: Martins-Melo et al.^161^*

**Chart 4-8:** *Source: Nóbrega et al.^159^*

**Chart 4-9:** *Source: Martins-Melo et al.^166^*

**Chart 4-10:** *Source: Nicolao et al.^183^*

**Table 5-3:** – Number of deaths, age-standardized mortality rate (per 100 000), and percent change of rates, by cardiovascular groups of causes of death, in Brazil and its Federative Units, 1990 and 2017

Cause of death and Location	1990		2017		Percent change (95% UI)
	Number (95% UI)	Rate (95% UI)	Number (95% UI)	Rate (95% UI)	
***B.2.1- Rheumatic heart disease***					
**Acre**	6.8 (6.3;7.3)	2.6 (2.5;2.8)	9.6 (8.8;10.4)	1.4 (1.3;1.5)	-46.2 (-51.8;-40.2)
**Alagoas**	43.8 (37.3;51.6)	2.2 (1.9;2.5)	37.5 (34.9;40.2)	1.1 (1.1;1.2)	-47.3 (-54.4;-36.5)
**Amapá**	2.7 (2.5;2.9)	1.8 (1.7;2)	6.6 (6;7.3)	1.1 (1;1.3)	-37.3 (-44.6;-29.1)
**Amazonas**	18.3 (16.9;19.7)	1.5 (1.4;1.6)	26 (23.7;28.2)	0.8 (0.8;0.9)	-44.7 (-49.7;-39)
**Bahia**	171.9 (155.9;186.2)	1.8 (1.6;1.9)	172 (159.2;185.3)	1.1 (1;1.2)	-40.3 (-46;-33.1)
**Brazil**	2647.8 (2549.9;2727.7)	2.4 (2.3;2.5)	2682.3 (2584.5;2796.7)	1.2 (1.1;1.2)	-50.3 (-52.4;-47.5)
**Ceará**	62.6 (51.4;72)	1.2 (1;1.3)	64.6 (60.2;69.2)	0.7 (0.6;0.7)	-44.3 (-51.3;-33.7)
**Distrito Federal**	33.4 (31.2;35.8)	3.7 (3.5;4)	38.9 (35.8;42.7)	1.6 (1.5;1.8)	-56.8 (-61;-52.4)
**Espírito Santo**	40.3 (38.1;42.6)	2.2 (2.1;2.4)	46.8 (43.4;50.8)	1.1 (1;1.2)	-50.6 (-54.7;-45.8)
**Goiás**	74.6 (69.8;79.9)	2.8 (2.6;3)	100.8 (93.9;108.4)	1.5 (1.4;1.6)	-46.8 (-51.2;-42)
**Maranhão**	100.4 (80.9;123.3)	2.3 (2;2.8)	59.8 (55.6;64.6)	0.9 (0.8;0.9)	-62.6 (-68.9;-55.4)
**Mato Grosso**	23.9 (22.1;26)	1.9 (1.8;2.1)	32.3 (30;34.9)	1 (0.9;1.1)	-47.7 (-52.6;-42)
**Mato Grosso do Sul**	25 (22;27.3)	2.1 (2;2.3)	33.3 (30.8;36.2)	1.2 (1.1;1.3)	-44.7 (-50;-38.8)
**Minas Gerais**	331.2 (311.9;350.1)	2.8 (2.6;2.9)	343 (321;367.1)	1.4 (1.3;1.5)	-50.6 (-54.5;-46.2)
**Pará**	52.4 (47;57.1)	1.7 (1.6;1.8)	70.3 (65.5;75.3)	1 (0.9;1)	-43.5 (-48.8;-37.2)
**Paraíba**	61.5 (53.4;70.2)	2.2 (2;2.5)	49.1 (44.5;53.8)	1.1 (1;1.2)	-51.8 (-58.3;-43.7)
**Paraná**	186.5 (177.8;196)	3.3 (3.1;3.4)	206.6 (193;221.9)	1.6 (1.5;1.8)	-49.5 (-53.4;-45.3)
**Pernambuco**	113.9 (105.4;122.5)	2 (1.8;2.1)	132.3 (122.1;143.1)	1.3 (1.2;1.4)	-33.3 (-38.9;-26.3)
**Piauí**	26.2 (19.9;31.3)	1.3 (1.1;1.5)	25.1 (23.3;27.1)	0.7 (0.6;0.7)	-47.5 (-55.3;-34.8)
**Rio de Janeiro**	242.4 (230.8;253.8)	2.2 (2.1;2.3)	230.3 (215.4;247)	1.1 (1;1.2)	-49.2 (-53;-44.7)
**Rio Grande do Norte**	31.9 (28.4;35.1)	1.6 (1.5;1.8)	44.1 (40.4;48.1)	1.2 (1.1;1.3)	-28.4 (-37.4;-16.5)
**Rio Grande do Sul**	136.7 (129.5;143.6)	1.9 (1.8;2)	128.1 (118.5;137.7)	0.9 (0.8;1)	-52.3 (-56.1;-48.1)
**Rondônia**	14.5 (13.3;15.8)	2.4 (2.2;2.6)	18.6 (16.5;21)	1.2 (1.1;1.4)	-49.5 (-56.3;-41.7)
**Roraima**	1.8 (1.6;2)	1.8 (1.7;2)	3.5 (3;4)	0.9 (0.8;1.1)	-49.1 (-56.6;-39.5)
**Santa Catarina**	72.3 (68.4;76)	2.4 (2.3;2.6)	86.2 (79.9;92.9)	1.2 (1.1;1.2)	-52.9 (-56.6;-48.6)
**São Paulo**	735.7 (704.1;764.8)	3.1 (3;3.2)	671.8 (632;718.7)	1.3 (1.2;1.4)	-57.8 (-60.6;-54.7)
**Sergipe**	24.3 (22.2;26.6)	2.1 (2;2.3)	28.2 (26.2;30.6)	1.3 (1.2;1.4)	-41.2 (-46.6;-34.5)
**Tocantins**	12.6 (8.5;15.5)	2.3 (1.8;2.7)	16.9 (15.3;18.6)	1.1 (1;1.3)	-49.7 (-57.7;-37.2)
***B.2.5- Non-rheumatic valvular heart disease***					
**Acre**	2.4 (2.2;2.8)	1.4 (1.3;1.7)	8.6 (7.3;9.5)	1.5 (1.3;1.7)	6.3 (-18.1;22)
**Alagoas**	14.4 (12.8;19)	1.1 (0.9;1.4)	42.3 (37.9;50.9)	1.4 (1.2;1.7)	31.4 (8.4;50.9)
**Amapá**	1.9 (1.3;2.1)	1.9 (1.4;2.1)	9.8 (6.4;10.9)	2.1 (1.4;2.4)	10.7 (-13.2;24.9)
**Amazonas**	11.9 (10.2;13.6)	1.5 (1.3;1.7)	40.5 (33.5;44.9)	1.5 (1.3;1.7)	5.5 (-12.3;18.3)
**Bahia**	96.1 (85.1;116.3)	1.4 (1.2;1.7)	197.5 (179.7;255.7)	1.2 (1.1;1.6)	-9 (-19.7;14.4)
**Brazil**	1495.1 (1284.4;1620.4)	1.7 (1.5;1.9)	3974.4 (3116.8;4165.1)	1.8 (1.4;1.9)	7.1 (-9.1;13)
**Ceará**	33.7 (26;60.7)	0.8 (0.6;1.5)	106.6 (96.7;136.7)	1.1 (1;1.4)	33.1 (-13.6;68.6)
**Distrito Federal**	13.4 (10.3;14.5)	2.3 (1.8;2.5)	41.6 (36.3;47.7)	2.1 (1.9;2.5)	-6.6 (-18.4;16.3)
**Espírito Santo**	30.9 (20.9;33.9)	2.4 (1.7;2.6)	86.1 (59.1;95.1)	2.1 (1.4;2.4)	-10.9 (-19.2;-2.6)
**Goiás**	31.4 (26.2;34.1)	1.7 (1.4;1.9)	97.3 (83.6;106.9)	1.5 (1.3;1.7)	-9.9 (-17;-0.9)
**Maranhão**	26.1 (19.5;46)	1 (0.7;1.8)	62.2 (52.7;100.7)	1 (0.8;1.6)	-1.9 (-16.9;19)
**Mato Grosso**	12.9 (11.4;14.5)	1.6 (1.4;1.8)	46.2 (39.6;51.2)	1.6 (1.4;1.8)	3.3 (-10.8;15.6)
**Mato Grosso do Sul**	16.5 (13;18)	1.9 (1.5;2.1)	50.6 (39.1;56)	1.9 (1.5;2.1)	-0.4 (-12.2;10.7)
**Minas Gerais**	153.8 (135.2;169.9)	1.6 (1.5;1.8)	419.1 (332.3;454.1)	1.7 (1.3;1.8)	2.8 (-15.8;13.1)
**Pará**	27.3 (24.8;34.4)	1.3 (1.1;1.6)	90.3 (81.5;107)	1.4 (1.3;1.7)	11.1 (-5.4;24.9)
**Paraíba**	17.5 (13.9;31.9)	0.8 (0.6;1.4)	39.3 (32.8;68.9)	0.8 (0.7;1.5)	10.1 (-11.1;33.2)
**Paraná**	105.3 (71.9;114.2)	2.4 (1.7;2.6)	293.1 (178.1;325.9)	2.5 (1.5;2.7)	4.1 (-14.8;14.3)
**Pernambuco**	66.5 (59.1;75.4)	1.5 (1.4;1.8)	172.1 (138.3;188.5)	1.8 (1.4;2)	17.2 (-4;31)
**Piauí**	13.1 (10.2;24.1)	0.9 (0.7;1.7)	34 (29.9;51.7)	0.9 (0.8;1.4)	1.1 (-22.9;23.9)
**Rio de Janeiro**	150.6 (135.6;201.8)	1.6 (1.5;2.3)	357.5 (305.4;408)	1.7 (1.5;1.9)	3.7 (-15.2;14.2)
**Rio Grande do Norte**	16.5 (13.9;24.9)	1 (0.8;1.5)	49 (44.2;62.1)	1.3 (1.2;1.6)	28.6 (-9.9;57.7)
**Rio Grande do Sul**	140.6 (98.8;152.4)	2.3 (1.6;2.5)	389.2 (217.1;436.1)	2.7 (1.5;3)	15.2 (-12.5;27.8)
**Rondônia**	5.9 (5.2;7.1)	1.6 (1.4;1.9)	19.5 (16.9;24.1)	1.4 (1.2;1.8)	-9.1 (-20.9;3.3)
**Roraima**	0.9 (0.8;1.2)	1.7 (1.4;2.4)	4.3 (3.7;5.6)	1.5 (1.3;1.9)	-6.7 (-32.7;15.8)
**Santa Catarina**	61 (37.5;67.1)	2.7 (1.6;3)	177.4 (104.2;198.4)	2.5 (1.5;2.9)	-4.4 (-15.1;4.4)
**São Paulo**	431.6 (309.8;460.1)	2.2 (1.7;2.3)	1097 (770;1195.5)	2.2 (1.6;2.4)	1.9 (-14.5;10.9)
**Sergipe**	7.7 (6.6;12.2)	0.9 (0.8;1.4)	23.3 (20.8;33.8)	1.1 (1;1.6)	24.3 (-0.4;44.1)
**Tocantins**	5.2 (4.1;7.4)	1.5 (1.2;2.3)	19.9 (17.6;22.7)	1.5 (1.3;1.7)	-3.3 (-38.6;21.9)
***B.2.5.1- Non-rheumatic calcific aortic valve disease***					
**Acre**	1.4 (1.1;1.6)	0.9 (0.7;1)	5.6 (4.8;6.8)	1 (0.9;1.2)	14.9 (-4.1;52.3)
**Alagoas**	7.2 (6.1;9.1)	0.6 (0.5;0.7)	26.8 (23.5;34.2)	0.9 (0.8;1.1)	60.5 (35.4;93.6)
**Amapá**	1.1 (0.6;1.3)	1.2 (0.7;1.4)	6.3 (4.5;7.4)	1.4 (1;1.7)	17.3 (-0.1;59.6)
**Amazonas**	7.9 (4.9;8.9)	1 (0.7;1.1)	29.4 (24.2;33)	1.2 (0.9;1.3)	13 (-2.6;56.4)
**Bahia**	62.9 (40.6;72.3)	0.9 (0.6;1)	134.5 (120.5;178)	0.8 (0.8;1.1)	-7.2 (-24.2;59)
**Brazil**	950.4 (722.7;1016.7)	1.1 (0.9;1.2)	2806 (2349.2;3132)	1.3 (1.1;1.4)	16.3 (9.6;34)
**Ceará**	17.3 (12.9;29.7)	0.4 (0.3;0.7)	68.6 (59.3;99.2)	0.7 (0.6;1)	62.7 (21;108.7)
**Distrito Federal**	8.3 (7.1;9.9)	1.5 (1.3;1.8)	29.2 (24.8;40.5)	1.6 (1.3;2.2)	1.4 (-14.4;36.2)
**Espírito Santo**	17.6 (11.7;19.9)	1.4 (1;1.6)	56.9 (43.8;64.3)	1.4 (1.1;1.6)	-1.9 (-14.3;21.5)
**Goiás**	19.4 (13.8;21.9)	1.1 (0.8;1.3)	67.5 (60;77.2)	1.1 (1;1.2)	-3.8 (-16.4;24.2)
**Maranhão**	13.2 (10.3;18.6)	0.5 (0.4;0.8)	41.1 (34.9;62.2)	0.7 (0.6;1)	25.3 (5.3;61.9)
**Mato Grosso**	7.7 (6;8.8)	1 (0.8;1.1)	30.9 (27.5;37)	1.1 (1;1.3)	10.3 (-4.6;41.6)
**Mato Grosso do Sul**	10.6 (7.1;12)	1.3 (0.9;1.5)	36 (28.9;40.3)	1.4 (1.1;1.5)	6.4 (-7.6;38.8)
**Minas Gerais**	98 (73.1;109)	1.1 (0.8;1.2)	295 (242.1;330.1)	1.2 (1;1.3)	10.4 (0.3;30.5)
**Pará**	15.1 (12.3;17.6)	0.7 (0.6;0.9)	55.4 (48;72.2)	0.9 (0.8;1.1)	19.9 (3.5;47.8)
**Paraíba**	8.9 (6.7;17.1)	0.4 (0.3;0.8)	25.8 (20.1;49.3)	0.6 (0.4;1.1)	39.6 (10.9;72.5)
**Paraná**	66.8 (42.3;75.1)	1.6 (1;1.8)	213.8 (136.2;240.2)	1.8 (1.2;2)	15.2 (3;34.3)
**Pernambuco**	35.8 (29.4;41.1)	0.8 (0.7;1)	111.3 (95.1;129.6)	1.2 (1;1.3)	36.7 (19.2;56.2)
**Piauí**	6 (4.6;10.6)	0.4 (0.3;0.8)	20 (16.4;33.7)	0.6 (0.5;0.9)	24 (-0.4;61.9)
**Rio de Janeiro**	94.3 (81.2;132.4)	1.1 (0.9;1.5)	262.7 (234.5;331)	1.3 (1.1;1.6)	17.9 (1.2;32.7)
**Rio Grande do Norte**	10.2 (8.3;13.1)	0.6 (0.5;0.8)	34.5 (30.4;44.4)	0.9 (0.8;1.2)	44 (17.3;86.2)
**Rio Grande do Sul**	93.5 (61.7;103.8)	1.6 (1.1;1.8)	289 (173.5;325.4)	2 (1.2;2.2)	25.1 (4.5;43.9)
**Rondônia**	3.5 (2.6;4.1)	1 (0.8;1.2)	13.1 (11;17.3)	1 (0.8;1.3)	-3 (-21.4;30.3)
**Roraima**	0.6 (0.4;0.7)	1.2 (0.9;1.4)	3.3 (2.7;4.1)	1.2 (1;1.5)	3.7 (-16.7;46.1)
**Santa Catarina**	40.2 (21.4;46.1)	1.8 (1;2.1)	132.6 (80.9;149.9)	1.9 (1.1;2.2)	4.8 (-6.6;29.4)
**São Paulo**	296.7 (198;326.2)	1.5 (1.1;1.7)	790.7 (611;885)	1.6 (1.2;1.8)	5.1 (-4;27.3)
**Sergipe**	3.8 (3.1;6.3)	0.5 (0.4;0.7)	14 (11.4;23.5)	0.7 (0.5;1.1)	48.6 (25.2;76.3)
**Tocantins**	2.5 (2;3.5)	0.8 (0.6;1.2)	12 (10.3;15.9)	0.9 (0.8;1.2)	13.4 (-11.5;49.4)
***B.2.5.2- Non-rheumatic degenerative mitral valve disease***					
**Acre**	0.8 (0.7;1.4)	0.4 (0.4;0.8)	2.3 (1.7;2.8)	0.4 (0.3;0.5)	-13.6 (-53.6;12)
**Alagoas**	6.4 (5.2;9.7)	0.4 (0.4;0.7)	13.8 (10.5;16.7)	0.4 (0.3;0.5)	-1 (-35.6;24.3)
**Amapá**	0.6 (0.5;0.9)	0.6 (0.5;0.9)	2.8 (1.5;3.5)	0.6 (0.3;0.7)	-5.4 (-54.4;19)
**Amazonas**	3.6 (2.9;6.3)	0.4 (0.3;0.7)	9.6 (7.2;12)	0.3 (0.3;0.4)	-13.8 (-52.1;11.2)
**Bahia**	30.9 (24.8;55.3)	0.4 (0.3;0.8)	58.3 (44.5;75.6)	0.4 (0.3;0.5)	-12.5 (-45.7;11.9)
**Brazil**	510.6 (450.7;676.7)	0.5 (0.5;0.7)	1086.1 (621.9;1214.9)	0.5 (0.3;0.5)	-10.8 (-47.6;2.4)
**Ceará**	14.5 (6.7;28.8)	0.3 (0.2;0.7)	34.4 (22.8;39.9)	0.3 (0.2;0.4)	2.3 (-51.5;71.2)
**Distrito Federal**	4.8 (2.8;5.5)	0.7 (0.4;0.8)	11.4 (4.4;14.4)	0.5 (0.2;0.7)	-25.4 (-61.7;-7.7)
**Espírito Santo**	12.7 (7.9;16.4)	0.9 (0.6;1.2)	27.2 (12.3;32.9)	0.7 (0.3;0.8)	-25.7 (-53.2;-11.9)
**Goiás**	11.2 (9.8;15.1)	0.5 (0.5;0.8)	27.1 (17.8;31.5)	0.4 (0.3;0.5)	-23.2 (-50.5;-5.8)
**Maranhão**	10.8 (4.8;26.3)	0.4 (0.2;1)	17.1 (9.6;37.7)	0.3 (0.1;0.6)	-33.4 (-48.1;-10.8)
**Mato Grosso**	4.8 (4;6.5)	0.5 (0.4;0.7)	14.1 (8.2;16.5)	0.5 (0.3;0.5)	-10.3 (-50.3;12.3)
**Mato Grosso do Sul**	5.6 (4.7;7.5)	0.6 (0.5;0.8)	13.7 (7.5;16.9)	0.5 (0.3;0.6)	-14.8 (-51.4;5.8)
**Minas Gerais**	52.7 (46.2;74.3)	0.5 (0.5;0.8)	116.6 (70;135.7)	0.5 (0.3;0.5)	-12.1 (-50.7;7.4)
**Pará**	11.1 (8.9;16.6)	0.5 (0.4;0.7)	30.4 (20.2;35.2)	0.5 (0.3;0.5)	-3.9 (-40.7;18.8)
**Paraíba**	7.3 (3.7;14.5)	0.3 (0.2;0.6)	11.2 (7.1;19.1)	0.2 (0.2;0.4)	-22 (-45;5.2)
**Paraná**	36.5 (25.4;49.7)	0.7 (0.5;1)	74.2 (32.6;98.5)	0.6 (0.3;0.8)	-18.9 (-53.8;-3.3)
**Pernambuco**	29.2 (24.3;36)	0.6 (0.5;0.8)	57.2 (32.8;66.9)	0.6 (0.3;0.7)	-8.1 (-41.3;8.9)
**Piauí**	6 (2.9;12.6)	0.4 (0.2;0.9)	12 (8.1;17)	0.3 (0.2;0.5)	-18.7 (-50.7;20.3)
**Rio de Janeiro**	54 (41.3;69.8)	0.6 (0.4;0.7)	89.8 (52;104.2)	0.4 (0.2;0.5)	-23 (-52;-9)
**Rio Grande do Norte**	5.5 (3.1;11.5)	0.3 (0.2;0.7)	12.5 (9.9;16.1)	0.3 (0.3;0.4)	1.3 (-46.2;62.2)
**Rio Grande do Sul**	44.8 (30.8;61.3)	0.7 (0.5;0.9)	94.5 (39;135.6)	0.6 (0.3;0.9)	-6.7 (-49.3;10.9)
**Rondônia**	2.1 (1.7;3.4)	0.5 (0.4;0.8)	5.3 (3.9;6.7)	0.4 (0.3;0.5)	-25.1 (-52.5;-2.4)
**Roraima**	0.2 (0.2;0.6)	0.4 (0.2;1)	0.7 (0.5;1.5)	0.2 (0.2;0.5)	-41.8 (-65.4;-12.9)
**Santa Catarina**	19.9 (13.3;30.3)	0.8 (0.5;1.3)	42.4 (18.9;60.8)	0.6 (0.3;0.9)	-25.7 (-54.3;-10.7)
**São Paulo**	128.9 (98.6;180)	0.6 (0.5;0.9)	292.7 (118.9;351.9)	0.6 (0.2;0.7)	-5.7 (-55.2;16.5)
**Sergipe**	3.4 (2.1;5.8)	0.4 (0.2;0.7)	8 (5.3;9.6)	0.4 (0.2;0.4)	-2.4 (-41.6;23.1)
**Tocantins**	2.3 (1.4;3.7)	0.4 (0.2;0.7)	6.8 (4.2;8)	0.5 (0.3;0.6)	-19.2 (-63.8;33.1)

*Source: Global Burden of Disease Study 2017, Institute for Health Metrics and Evaluation.^236^*

**Table 5-4:** – Number of DALY, age-standardized DALY rates (per 100 000), and percent change of rates by cardiovascular groups of causes of death, in Brazil and its Federative Units, 1990 and 2017

Cause of death and Location		1990								Percent change (95% UI)
		Number (95% UI)		Rate (95% UI)		Rate (95% UI)				
***B.2.1- Rheumatic heart disease***										
Acre	460.8 (410.3;524.6)			127.1 (114.1;144.3)	671 (557.8;816.4)	79.5 (67.1;95.5)			-37.5 (-43.8;-30.7)	
Alagoas	3143.3 (2600.7;3851.6)			125 (105.1;149.3)	2632.8 (2168.9;3233.3)	73.8 (61.1;90.1)			-40.9 (-50;-30.9)	
Amapá	204.8 (173.1;242.1)			91.4 (78.4;106.6)	551.5 (440.6;687.6)	71.1 (57.7;87.5)			-22.2 (-29.7;-14.4)	
Amazonas	1528 (1267.3;1848.2)			85.2 (71.4;102.2)	2460.5 (1938.2;3170.4)	62.1 (49.5;78.9)			-27.1 (-33.3;-20.8)	
Bahia	12095.4 (10485.4;13973.7)			103.7 (90.3;120.3)	11460 (9428.5;14129.4)	70.5 (58.1;86.8)			-32.1 (-38.7;-24.8)	
Brazil	163388 (144922.3;186177.2)			118.9 (106.1;134.7)	162013.6 (134153.5;198443.9)	71.2 (59;87.2)			-40.1 (-45.4;-34.6)	
Ceará	5090.4 (4101.6;6158.3)			81.2 (65.9;98.4)	5664.1 (4357.1;7338.2)	56.4 (43.5;72.8)			-30.6 (-39.8;-20.8)	
Distrito Federal	2073.5 (1853.8;2341.2)			151.6 (137.6;168.9)	2439.1 (2023.3;2978.4)	78.8 (65.8;94.9)			-48 (-53.7;-42)	
Espírito Santo	2628.8 (2295.3;3051.6)			109.2 (96.1;125.8)	2974.6 (2426.3;3661.6)	70.1 (57.1;86.3)			-35.8 (-42;-29.9)	
Goiás	4735.6 (4205.1;5429.8)			128 (114.7;145.2)	5840.8 (4903.6;7144.8)	79.8 (67.3;97.3)			-37.7 (-43.5;-31.5)	
Maranhão	7473 (5921.2;9327.9)			142.1 (116.4;171.6)	4842.9 (3887.8;6116.6)	63.2 (51;79.3)			-55.6 (-64.4;-46.3)	
Mato Grosso	1828.6 (1579.2;2132.2)			101.6 (88.5;117.3)	2466 (1996.8;3072)	66.9 (54.5;82.8)			-34.2 (-40.9;-27.8)	
Mato Grosso do Sul	1669 (1402.1;1974.2)			104.5 (90.1;121.8)	2109.8 (1733.1;2612.1)	71.2 (58.6;87.9)			-31.8 (-38.6;-24.4)	
Minas Gerais	19249.1 (17181.8;21938.6)			130.8 (117.6;148)	18154.8 (15183.7;21952.5)	76.5 (63.5;92.8)			-41.5 (-47.4;-35.5)	
Pará	3950.3 (3349.3;4663.4)			91.5 (78.6;107.5)	5808.4 (4645;7300.4)	65.9 (53.3;82)			-27.9 (-34.8;-20.8)	
Paraíba	3916.4 (3288.8;4699.1)			125.1 (106.1;147.5)	3123.4 (2541.7;3837.2)	70.6 (57.5;86.9)			-43.5 (-52;-34.8)	
Paraná	10399.6 (9331.5;11680)			136.1 (123.1;152.3)	10256.3 (8682.8;12479.2)	81.7 (68.6;99.4)			-40 (-45.5;-33.8)	
Pernambuco	7748.4 (6752.1;8887.8)			110.2 (95.9;126.4)	8150.9 (6836.5;9867.3)	78.7 (66.2;95.3)			-28.5 (-34.6;-22.1)	
Piauí	2106.2 (1603.1;2601.3)			83.8 (66;103.3)	2139 (1699.7;2745.7)	58.6 (46.7;75.1)			-30.1 (-40.3;-17.3)	
Rio de Janeiro	14806.5 (13066.6;16943.4)			113.9 (100.9;129.8)	13785.8 (11373.1;17049.6)	70.7 (58;88)			-37.9 (-43.9;-31.8)	
Rio Grande do Norte	2163 (1817.4;2559.8)			94.3 (79.8;111.1)	2852.8 (2365.9;3507.2)	75.4 (62.7;92.4)			-20.1 (-28.4;-10.9)	
Rio Grande do Sul	8371.7 (7163.3;9922.7)			94.4 (81.5;110.9)	7857.9 (6265.8;9956.8)	62 (48.9;78.6)			-34.4 (-40.8;-27.9)	
Rondônia	1066 (924.3;1232.7)			113.4 (100;129.4)	1317 (1055;1625.9)	73 (58.9;89.6)			-35.7 (-43.4;-28.3)	
Roraima	153.9 (127.6;187.4)			88.8 (75.3;105.5)	335.3 (262.1;430.8)	61.7 (48.9;77.9)			-30.5 (-38.5;-22.8)	
Santa Catarina	4413.5 (3816.7;5148.1)			108.6 (95.7;125)	5283.7 (4296.7;6556.9)	68.4 (55.6;85)			-37 (-43.4;-30.6)	
São Paulo	39592.8 (35476.3;45103.4)			134.5 (121.5;151.7)	35783 (29557.6;44123.8)	70.9 (58.2;87.7)			-47.3 (-53.3;-41.3)	
Sergipe	1627.5 (1401.2;1913.6)			114.8 (100.3;133)	1870.6 (1561.8;2277.1)	77 (64.6;93.1)			-33 (-40.2;-26.3)	
Tocantins	891.8 (595.4;1118.1)			109.4 (80.7;133.7)	1181.6 (973.8;1459.3)	72.9 (60.4;89.7)			-33.3 (-44.6;-15.9)	
***B.2.5- Non-rheumatic valvular heart disease***										
Acre		76.6 (68.6;89.8)		34.6 (31.1;41)	231.2 (202.9;258.1)	34.9 (30.4;39)		1.1 (-16.5;14.5)		
Alagoas		435.5 (385.3;565.4)		27.1 (24;35.1)	1122.2 (1002.4;1359.9)	34.4 (30.7;41.5)		26.9 (7.9;43.8)		
Amapá		60 (41.1;66.2)		43.9 (31;48.5)	276 (187.7;307.7)	48.3 (32.6;53.9)		10 (-7.8;24.5)		
Amazonas		383.1 (320.1;430.5)		35.8 (30.9;40.2)	1120.8 (942.5;1248.8)	36.4 (30.5;40.4)		1.7 (-10.3;14)		
Bahia		2839.9 (2502.2;3279.7)		35.1 (31.1;40.7)	4949.5 (4486.8;6140.9)	30.8 (28;38.3)		-12.2 (-22.2;12)		
Brazil		45589.2 (38652.3;48617.4)		42.9 (36.6;45.8)	89684.3 (75761.8;95891.8)	39.4 (33.2;42.2)		-8 (-15.6;-4)		
Ceará		979.5 (757.8;1644.8)		21.4 (16.6;36)	2601.6 (2333.2;3343)	26.2 (23.5;33.6)		22.2 (-13.9;51.7)		
Distrito Federal		475.3 (369.5;517.4)		53 (41.8;57.3)	1026.2 (904.4;1164.8)	40.2 (35.3;45.4)		-24.1 (-32.1;-7.6)		
Espírito Santo		954.5 (637.9;1049.7)		53.2 (36.8;58.2)	1994.2 (1441.6;2200)	46.7 (33.9;51.6)		-12.2 (-19.7;-3.4)		
Goiás		1044.4 (870.5;1136.7)		40.3 (34;43.8)	2513.9 (2156.8;2780.7)	36.3 (31.4;40.2)		-10 (-17;-1)		
Maranhão		841.7 (638.4;1353.6)		27.4 (21;44.5)	1646 (1410.8;2450.2)	24.5 (21;36.5)		-10.4 (-24;7.8)		
Mato Grosso		448.3 (391.9;502.9)		39.1 (34.6;43.8)	1235 (1068.3;1378.4)	37.4 (32.3;41.7)		-4.3 (-15.6;7.1)		
Mato Grosso do Sul		518 (401.4;571.5)		45.4 (35.9;49.7)	1237.3 (960.8;1368.1)	43.2 (33.7;47.7)		-4.9 (-15.2;6.1)		
Minas Gerais		4805.1 (4079.6;5198.5)		41.1 (35.6;44.6)	9545.6 (7991.5;10392.3)	38 (31.9;41.5)		-7.7 (-17.5;-0.1)		
Pará		871.1 (775.6;1053.9)		31.3 (28.2;38.5)	2471.2 (2222.8;2874.6)	33.9 (30.5;39.6)		8.3 (-6.6;20.6)		
Paraíba		485 (382.9;858.3)		19.9 (15.8;34.8)	978.9 (818.3;1667.4)	21.5 (17.9;36.6)		8.1 (-11.5;28.3)		
Paraná		3211.1 (2218.8;3517.2)		54.6 (38.2;59.6)	6327.7 (4281.6;6982.9)	49.8 (33.6;55)		-8.8 (-18.1;-1.4)		
Pernambuco		2014.5 (1698.1;2212.9)		38.6 (33.3;42.7)	4112.2 (3493.4;4518.3)	41 (34.7;44.9)		6.1 (-4.3;16.3)		
Piauí		381.9 (291.7;687.1)		23.2 (18.1;41)	879.2 (770.3;1289.3)	24.2 (21.2;35.5)		4.3 (-18.9;27.5)		
Rio de Janeiro		4639.3 (4053.3;5965.9)		41.8 (37.1;54.1)	7855.6 (7028.2;9622.4)	37.1 (33.3;45.6)		-11.2 (-18.7;-3.6)		
Rio Grande do Norte		446.7 (374.6;658.4)		25.3 (21.2;37)	1205.9 (1082.5;1495.1)	31.9 (28.6;39.6)		26.3 (-7.3;51.8)		
Rio Grande do Sul		4017.2 (2844.8;4352.4)		53.9 (38.7;58.5)	7588.9 (4941.1;8430.1)	51.9 (34.2;57.5)		-3.7 (-19.7;5.2)		
Rondônia		221.1 (193.3;264.1)		39.1 (34.9;46.8)	524.4 (452.9;662.3)	33.5 (29;41.7)		-14.5 (-25.9;-1.6)		
Roraima		32 (27.8;41.7)		35.3 (30.8;48.9)	120.3 (104;161.8)	31.5 (27.3;41)		-10.9 (-28.3;7.3)		
Santa Catarina		1766.7 (1153.7;1937.8)		58.3 (37.4;63.9)	3758.7 (2515;4171.6)	49.2 (32.4;54.7)		-15.6 (-22.8;-7.7)		
São Paulo		13252 (9281.2;14232.8)		54.6 (39;58.4)	23226.5 (18142.4;25116.5)	44.9 (35.1;48.6)		-17.7 (-24.1;-10.8)		
Sergipe		218.2 (184.3;330.5)		22.6 (19.1;34.2)	615.9 (541.8;879.7)	27.4 (24.2;39.1)		21.2 (1.3;39)		
Tocantins		170.7 (124.4;233.6)		33.3 (26.2;46.2)	519.5 (455.3;586.8)	35.4 (31.1;40)		6.4 (-24;35.7)		
***B.2.5.1- Non-rheumatic calcific aortic valve disease***										
Acre		40.5 (30;46.2)		19.1 (14.6;21.7)	133.9 (112.7;168.1)	20.8 (17.7;25.7)		9 (-10.8;51.7)		
Alagoas		189.8 (160.5;241.4)		12.2 (10.4;15.3)	625.7 (549.3;827.4)	19.4 (17.1;25.3)		58.6 (33.4;93.9)		
Amapá		32.1 (18.2;37)		24.9 (14.5;28.3)	161.6 (122.6;192)	29.4 (21.7;34.5)		17.8 (-0.4;68.2)		
Amazonas		231.6 (136.2;266.9)		22.4 (13.8;25.4)	739.3 (617.9;840)	24.4 (20.1;27.5)		9.1 (-6.7;58.5)		
Bahia		1658.3 (1011.9;1940.1)		20.9 (13.1;24.3)	3000.9 (2707.6;3816.1)	18.8 (16.9;23.9)		-10.4 (-27;61.2)		
Brazil		26502.4 (19395.6;28697.2)		25.4 (19.1;27.4)	56930.9 (50545.9;65681.9)	25.1 (22.1;28.7)		-1.2 (-9.1;24.2)		
Ceará		429.8 (315.7;695.5)		9.6 (7.1;15.5)	1451.9 (1234;2158.8)	14.7 (12.5;21.7)		52.5 (18.4;95)		
Distrito Federal		271.8 (234.1;332.2)		32.1 (27.4;38.2)	655.9 (552.3;920.4)	26.3 (22.3;36.6)		-18.1 (-30.1;8.5)		
Espírito Santo		488.7 (313.1;557)		28.5 (18.8;32.2)	1182.1 (959.5;1371.1)	27.8 (22.4;32)		-2.4 (-15.2;28.3)		
Goiás		589.3 (415.1;674)		23.7 (17;26.7)	1582.9 (1418.5;1838.7)	23 (20.6;26.6)		-3 (-15.7;29.8)		
Maranhão		377.9 (295.1;491.2)		12.6 (10;16.7)	951.9 (812.4;1361.8)	14.3 (12.2;20.4)		13.4 (-5.9;44.1)		
Mato Grosso		243.7 (182.5;280.4)		22.4 (17.3;25.6)	749.8 (666.3;912.9)	23 (20.5;27.7)		2.6 (-12.2;37)		
Mato Grosso do Sul		302.3 (199.2;348.4)		27.5 (18.3;31.2)	806 (658.2;903.3)	28.3 (23;31.6)		2.8 (-12;39)		
Minas Gerais		2808.8 (1974.5;3167.3)		24.5 (17.8;27.4)	6005.2 (5096.3;6815.2)	23.9 (20.4;27.2)		-2.5 (-13.6;22.8)		
Pará		423.2 (333.3;485.2)		16 (13;18.4)	1351.8 (1171.1;1746.7)	19 (16.5;24.4)		18.6 (1.6;53.1)		
Paraíba		206.3 (155.1;396.3)		8.6 (6.5;16.2)	547.1 (426.1;1049)	12 (9.3;23.1)		40.1 (11.5;73.1)		
Paraná		1874.7 (1154.1;2123.9)		32.9 (20.9;36.9)	4232.1 (3003.5;4719)	33.4 (23.7;37.2)		1.4 (-9;23.8)		
Pernambuco		965.9 (746.3;1081.1)		19 (15.1;21.3)	2374.9 (2111.6;2875.8)	23.8 (21.2;28.7)		25.5 (9.4;53.7)		
Piauí		152.4 (113.5;255.6)		9.6 (7.3;16.1)	449.8 (369.5;734.1)	12.4 (10.2;20.3)		29.1 (2.6;69.1)		
Rio de Janeiro		2651.5 (2230.2;3550.9)		24.2 (20.8;33)	5140.9 (4615.8;7109.1)	24.2 (21.8;33.6)		0 (-10.3;18.3)		
Rio Grande do Norte		241.5 (194.2;300.7)		13.8 (11.2;17.3)	750.5 (657;955.2)	19.9 (17.4;25.4)		43.9 (15.2;93.5)		
Rio Grande do Sul		2457.2 (1619.1;2728)		33.6 (22.2;37.2)	5106.1 (3487.4;5690.3)	34.8 (23.9;38.6)		3.8 (-6.2;22.4)		
Rondônia		121.9 (90.7;143.3)		22.8 (17.7;26.2)	317.1 (262.3;435)	20.6 (17.2;27.3)		-9.8 (-28;26.9)		
Roraima		20.4 (14.5;23.9)		23.2 (17.5;26.6)	82.2 (68.4;107.1)	22.1 (18.7;27.4)		-4.7 (-23.2;36.4)		
Santa Catarina		1071.1 (587;1233.8)		36.6 (19.9;41.7)	2571.3 (1727.2;2869.9)	33.9 (22.4;37.9)		-7.3 (-17.4;18.6)		
São Paulo		8484.5 (5317.1;9384.8)		35.5 (23.3;39.1)	15357.3 (13142.4;17600.3)	29.7 (25.2;33.9)		-16.2 (-26;10.5)		
Sergipe		93.2 (75.2;149.4)		9.9 (8;15.8)	324.7 (258.7;557.4)	14.6 (11.7;24.7)		47.2 (20.2;81.9)		
Tocantins		74 (53.8;103.7)		15.3 (11.9;21)	278.2 (234.9;375.9)	19.2 (16.3;25.7)		25.8 (-2.9;66.6)		
***B.2.5.2- Non-rheumatic degenerative mitral valve disease***										
Acre		30 (25.2;46)		13.1 (10.9;20.6)	75.1 (60.5;90.7)	11.2 (8.9;13.4)		-14.8 (-45.7;3.5)		
Alagoas		220.5 (183.7;311.8)		13.5 (11.2;19)	444.4 (356.2;522.7)	13.4 (10.8;15.8)		-0.1 (-29.4;20.1)		
Amapá		23.8 (19.6;30.9)		16.6 (14;21.9)	91.8 (53.3;112.4)	15.6 (9.2;19)		-6 (-44.1;13.9)		
Amazonas		136 (113;215.8)		12.2 (10.1;19.4)	331.9 (263.6;401.4)	10.6 (8.4;12.7)		-13.3 (-43;4.9)		
Bahia		1104.1 (905.1;1731.5)		13.3 (10.9;21.1)	1828.7 (1488.1;2227.1)	11.3 (9.2;13.8)		-14.9 (-42.1;2.9)		
Brazil		17936 (15500.4;22621.4)		16.4 (14.4;20.8)	30580.8 (20064.3;34729.6)	13.4 (8.8;15.2)		-18.7 (-43.4;-9.3)		
Ceará		493.4 (278.6;898.4)		10.7 (6.1;19.1)	1052.9 (767.3;1230.3)	10.6 (7.7;12.4)		-1.2 (-43.9;35.8)		
Distrito Federal		190.7 (115.1;218.4)		19.7 (12.4;22.5)	339.3 (159.2;421.1)	12.8 (6.2;15.9)		-35.1 (-59.2;-22.7)		
Espírito Santo		443.7 (279.5;557.8)		23.6 (15.4;29.7)	762.6 (387;901.9)	17.7 (9.1;21)		-24.9 (-45.7;-13.2)		
Goiás		424.4 (369.9;542.4)		15.6 (13.6;20)	855.1 (613.7;989.7)	12.2 (8.9;14.2)		-21.6 (-43.6;-8.5)		
Maranhão		385.9 (203.6;833)		12.5 (6.8;26.7)	582.5 (380.8;1068.8)	8.6 (5.7;15.7)		-31 (-45.2;-11)		
Mato Grosso		190.4 (160.8;242.1)		15.7 (13.3;20.6)	447.5 (286.6;524.3)	13.3 (8.7;15.7)		-14.8 (-44.4;3.3)		
Mato Grosso do Sul		204.1 (169.1;260.1)		17 (14.4;21.8)	406.4 (251.7;480.2)	14.1 (8.8;16.6)		-17.4 (-46;-0.6)		
Minas Gerais		1890.6 (1645.4;2507.9)		15.8 (13.9;21.1)	3353.1 (2245.4;3860.9)	13.3 (9;15.3)		-15.7 (-44.4;-0.2)		
Pará		404.9 (333.3;563.2)		14 (11.7;19.8)	983 (706.3;1136.6)	13.3 (9.6;15.4)		-5.5 (-35.3;12.4)		
Paraíba		237.4 (142.8;443.7)		9.7 (5.9;17.8)	370 (265.7;569.3)	8.1 (5.8;12.5)		-16.5 (-38.4;3.1)		
Paraná		1268 (887.1;1627.5)		20.6 (14.9;26.9)	1968.9 (1084.3;2414.3)	15.4 (8.5;18.9)		-25.5 (-48;-13.3)		
Pernambuco		999.8 (786.7;1162.9)		18.8 (15.1;22)	1641.1 (1077;1887.1)	16.2 (10.7;18.7)		-13.7 (-34;-1.5)		
Piauí		195.7 (108.4;388.3)		11.8 (6.8;22.7)	376.3 (274.4;508.7)	10.3 (7.5;14)		-12.3 (-42.4;17.5)		
Rio de Janeiro		1909.1 (1454.4;2345.8)		16.9 (13;20.8)	2593.4 (1731.6;3056.8)	12.3 (8.2;14.5)		-27.2 (-47.3;-16.1)		
Rio Grande do Norte		183.1 (118.7;339.9)		10.3 (6.7;18.9)	401.6 (333.7;489.9)	10.6 (8.8;12.9)		3 (-38.3;45.6)		
Rio Grande do Sul		1483.2 (1058.6;1927.1)		19.4 (13.9;25.2)	2354.6 (1214.6;2951.9)	16.1 (8.4;20.2)		-16.7 (-46.7;-2.1)		
Rondônia		87 (71.6;132.9)		14.6 (12.1;22.1)	172.4 (133;219)	10.9 (8.5;13.5)		-25.6 (-47.3;-7.7)		
Roraima		9.1 (6.4;21.2)		10 (7.1;23)	27 (19.8;52.4)	7 (5.2;12.6)		-30.3 (-53.4;-10)		
Santa Catarina		664.1 (474.2;931.5)		20.8 (14.7;29.9)	1126.1 (625.8;1436.1)	14.5 (8.2;18.8)		-30.2 (-50.8;-18.5)		
São Paulo		4562.6 (3474.3;6176.4)		18.3 (14.3;24.5)	7528.5 (4049.6;8857.7)	14.5 (7.8;17)		-20.8 (-50.7;-5.8)		
Sergipe		111.2 (75.5;172)		11.4 (7.8;17.5)	256 (187.3;305.8)	11.3 (8.3;13.7)		-0.9 (-32.9;20.8)		
Tocantins		83.2 (52.6;126.5)		15.7 (10.1;24)	210.5 (142.8;246.4)	14.2 (9.7;16.6)		-9.5 (-49.6;31)		

*Source: Global Burden of Disease Study 2017, Institute for Health Metrics and Evaluation.^236^*

**Chart 5-1:** Data derived from Global Burden of Disease Study 2017 (GBD 2017).^236^

**Chart 5-2:** Data derived from Global Burden of Disease Study 2017 (GBD 2017).^236^

**Chart 5-3:** – A: Age-standardized mortality rates attributable to Rheumatic Heart Disease in Brazil in 1990 – 2017. B: Crude mortality rates attributable to Rheumatic Heart Disease in Brazil in 1990 – 2017. C: Proportional mortality (%) attributable to Rheumatic Heart Disease in the Brazilian population in 1990 – 2017.

Data derived from Global Burden of Disease Study 2017 (GBD 2017).^236^

**Chart 5-4:** Data derived from Global Burden of Disease Study 2017 (GBD 2017).^236^

**Chart 5-5:** Data derived from Global Burden of Disease Study 2017 (GBD 2017).^236^

**Chart 5-6:** Data derived from Global Burden of Disease Study 2017 (GBD 2017).^236^

**Chart 5-7:** Data derived from Global Burden of Disease Study 2017 (GBD 2017).^236^

**Chart 5-8:** Data derived from Global Burden of Disease Study 2017 (GBD 2017).^236^

**Chart 5-9:** Data derived from Global Burden of Disease Study 2017 (GBD 2017).^236^

**Chart 5-10:** Data derived from Global Burden of Disease Study 2017 (GBD 2017).^236^

**Table 6-1:** – Number of prevalent cases and age-standardized prevalence of atrial fibrillation and flutter, per 100 000 inhabitants, in 1990 and 2017, with percent change, by sex, in Brazil and Brazilian Federative Units

	Both Sexes			Female			Male		
Brazil and Federative Units	1990 (95% UI)	2017 (95% UI)	Percent change (95% UI)	1990 (95% UI)	2017 (95% UI)	Percent change (95% UI)	1990 (95% UI)	2017 (95% UI)	Percent change (95% UI)
Acre	585.2 (482.8;692.5)	600.8 (493.1;714.2)	2.7 (7.1;-2)	487.9 (408.1;569.3)	508.3 (427;596.5)	4.2 (10.3;-2.2)	669.8 (540.1;806.8)	697.2 (555;849.8)	4.1 (10.6;-2.5)
Alagoas	426.1 (308.3;568)	428.7 (312.2;556.5)	0.6 (5.9;-5)	320.2 (221.7;432.7)	322.5 (225.4;434.5)	0.7 (7.5;-6.3)	546.6 (398.9;735.7)	562.5 (421.5;734.3)	2.9 (10.7;-5.3)
Amapá	673.7 (580.9;766.1)	696.7 (600.5;800.2)	3.4 (7.3;-0.5)	538.9 (464;616.7)	559.2 (483.3;643.2)	3.8 (8.8;-1.3)	816.7 (703.6;936.5)	848.1 (726.9;976.7)	3.8 (10.1;-1.8)
Amazonas	482.5 (375.5;610.5)	503 (385.7;636.4)	4.3 (9.9;-0.4)	349.6 (257.3;461.3)	360.5 (264.6;476.8)	3.1 (9.9;-3.1)	620 (490.9;766.8)	655.3 (510;813)	5.7 (13;-0.7)
Bahia	447.3 (332.2;579.6)	469.7 (346.1;606.8)	5 (10.1;0.1)	336.9 (242.4;445.2)	359.7 (258.1;475.1)	6.8 (13.6;-0.5)	574.2 (436.2;733.4)	605.7 (454;772.5)	5.5 (14;-1.6)
Brazil	619 (515.5;727.6)	640.9 (537;751)	3.5 (5.3;1.7)	499.3 (417.9;586.5)	522.1 (439.9;610.1)	4.6 (7;2.3)	758.9 (630;893.1)	786.9 (656.6;925)	3.7 (5.9;1.4)
Ceará	470.1 (361.8;598.3)	481.7 (368.6;612.8)	2.5 (7.1;-1.9)	338.8 (242.3;450.9)	348.8 (250.2;463.5)	2.9 (9.6;-3.7)	618.3 (486.1;767.3)	645.7 (505.6;803.7)	4.4 (10.7;-2.2)
Distrito Federal	649.2 (560.8;745.1)	665.2 (569.5;769.2)	2.5 (7.2;-1.6)	528.1 (456.8;602.9)	547.3 (468.8;629.1)	3.6 (9.8;-1.9)	796.8 (686.4;920.9)	824.8 (698.9;968.1)	3.5 (10.7;-2.1)
Espírito Santo	483.6 (371.6;608.6)	505 (386.6;644.5)	4.4 (9.4;-0.7)	381 (295.8;478.9)	401.9 (311.9;509.7)	5.5 (13.1;-0.8)	597 (453;756.4)	629.5 (479.9;809)	5.4 (12.5;-0.9)
Goiás	487.3 (378.1;605.3)	511.8 (397.7;643.5)	5 (10.8;-0.1)	438.8 (359.7;524.3)	461.1 (377.2;557.3)	5.1 (12;-1.8)	533.3 (381.5;695.6)	568.7 (412.3;754.8)	6.6 (14.8;-1.1)
Maranhão	377.7 (251.2;546.2)	351.4 (234.6;502)	-7 (-1.9;-13.4)	300.6 (197.9;439.7)	281.1 (185.6;398.6)	-6.5 (0.1;-14.7)	467.9 (312.4;676.6)	432.4 (290.1;621.1)	-7.6 (0.1;-16)
Mato Grosso	537.9 (432.1;652)	558.1 (443.3;686)	3.8 (8.6;-1.1)	456.4 (381.1;543.6)	477.2 (393.2;572.4)	4.6 (11.2;-1.6)	607.2 (471.4;759.2)	637.4 (491.4;812.9)	5 (11.6;-1.8)
Mato Grosso do Sul	678.5 (588.7;774.3)	690.4 (594.9;796.2)	1.8 (6.6;-2.3)	532.6 (459.2;608.8)	551.4 (477.8;631.7)	3.5 (9.7;-1.9)	815.8 (706.7;938.3)	845.2 (719.9;986)	3.6 (10.2;-2.2)
Minas Gerais	716.3 (620.7;815.9)	732.2 (646.8;820.9)	2.2 (7.8;-2.6)	554.1 (476.8;635)	565.7 (498.8;638.4)	2.1 (9;-3.9)	907.1 (783.5;1034.5)	930.7 (822.7;1040.7)	2.6 (9;-2.9)
Pará	426.2 (310.8;561)	434.7 (314;575.1)	2 (7.4;-3.1)	312 (218.4;434.1)	309.5 (215.6;429.8)	-0.8 (6.5;-7.7)	549.8 (406.6;719.6)	569.2 (416;743)	3.5 (9.7;-3.5)
Paraíba	507.8 (405.5;622)	527.4 (418.9;650.3)	3.9 (8.1;-0.2)	417.8 (341.3;505.7)	438.5 (352.9;531.2)	5 (11;-1.2)	610.3 (472.6;762.7)	640.1 (498.3;810.1)	4.9 (11.5;-1.8)
Paraná	588.3 (488.1;692.4)	607.7 (502.3;721.1)	3.3 (7.6;-0.9)	493.9 (418.7;573.1)	514.7 (434.7;602.9)	4.2 (10;-2)	686.9 (554.8;827.8)	719 (576.4;870.9)	4.7 (12.5;-1)
Pernambuco	422.7 (304.4;559.1)	442 (317.7;588.2)	4.6 (10.1;-0.4)	335.9 (238.6;447.7)	354.9 (255.2;473.6)	5.7 (14;-1.6)	528.9 (379.7;703.9)	558.1 (400.4;750.7)	5.5 (11.9;-1.7)
Piauí	429.2 (311.3;569)	437.6 (318.1;577.5)	2 (7.4;-2.4)	322 (227.4;434.3)	327.8 (229.5;439.6)	1.8 (9.4;-5.2)	550.2 (408.1;719.3)	568.7 (417.2;750.2)	3.4 (9.7;-2.6)
Rio de Janeiro	723.3 (626.3;827)	742.4 (646.8;845.8)	2.6 (6.6;-1.4)	567.3 (487;652.3)	585 (506.3;671.8)	3.1 (8.3;-2.2)	934.3 (812;1071.9)	956.7 (831.5;1094.2)	2.4 (8.2;-3.3)
Rio Grande do Norte	458.2 (345.8;587)	467.1 (346.5;603.8)	1.9 (7.6;-3.1)	337.9 (242.7;446.4)	347.7 (247;466.6)	2.9 (11.2;-4.7)	592.8 (458.7;744.5)	617 (468.6;789.3)	4.1 (11;-3)
Rio Grande do Sul	509.1 (399;629.9)	534.9 (418.9;668)	5.1 (9.5;0.3)	435.5 (351.1;526.3)	456.5 (367.2;558.1)	4.8 (11.9;-1.6)	604.9 (449;773)	635.8 (476.4;823.5)	5.1 (11.8;-0.9)
Rondônia	515.5 (408.2;637)	529.1 (416;655)	2.6 (7.3;-2.3)	436.6 (357.1;521.5)	455.1 (371.5;547.6)	4.2 (11.1;-2.2)	580.5 (446;741.2)	601.5 (453.1;767.6)	3.6 (10.8;-3)
Roraima	702.9 (609.8;804.9)	741.4 (646.1;844.4)	5.5 (9.5;1.6)	523.1 (451.2;603.1)	562.9 (488.7;641.4)	7.6 (13.8;2)	843.5 (728.4;969)	909.8 (793.3;1039.6)	7.9 (13.4;2.4)
Santa Catarina	617.1 (516.7;722.4)	641.9 (539.1;754.4)	4 (8.5;-0.2)	561.6 (482.9;640.9)	582.5 (504;667.2)	3.7 (9.4;-2.4)	679.5 (544.4;820.5)	711.7 (566.7;873)	4.7 (11.1;-1.6)
São Paulo	849.8 (736.7;972.7)	865.6 (753.4;984.3)	1.9 (6.1;-1.9)	702.6 (605.4;807.1)	726.4 (629.4;832.9)	3.4 (9.7;-1.6)	1028.3 (890.9;1176.3)	1042.9 (903.6;1190.2)	1.4 (6.8;-4.1)
Sergipe	518.5 (419.8;629.8)	536.3 (436.6;654.6)	3.4 (8.5;-1.3)	390.7 (312.6;481.3)	408.3 (322.8;515.6)	4.5 (11.9;-2.6)	669.2 (543.8;805.3)	699.4 (570.4;847.3)	4.5 (10.9;-1.6)
Tocantins	613.3 (518.7;713.7)	632.8 (532.1;743)	3.2 (8.2;-1.6)	448.1 (372.1;527.4)	465.7 (385.1;557.3)	3.9 (9.7;-1.4)	765.1 (651.1;890.6)	794.5 (668.7;927.2)	3.8 (10.3;-2.9)

*Source: Global Burden of Disease Study 2017, Institute for Health Metrics and Evaluation.^285^*

**Table 6-2:** – Number of deaths and age-standardized mortality rate due to atrial fibrillation and flutter, per 100 000 inhabitants, in 1990 and 2017, with percent change, in Brazil and Brazilian Federative Units

	1990		2017		
	Number of deaths (95% UI)	Mortality rate (95% UI)	Number of deaths (95% UI)	Mortality rate (95% UI)	Percent change (95% UI)
Acre	4 (4;5)	4.9 (4.5;5.5)	22 (20;24)	4.5 (4.1;4.8)	-8.6 (-18.4;-0.9)
Alagoas	35 (33;41)	3.5 (3.3;4.1)	119 (112;135)	4.1 (3.8;4.7)	16.1 (4.2;26.2)
Amapá	3 (3;3)	5.8 (5.4;6.5)	18 (16;19)	5.3 (4.7;5.6)	-9.6 (-20.2;-2.2)
Amazonas	19 (17;22)	3.9 (3.7;4.7)	87 (82;96)	4 (3.8;4.5)	3.2 (-11.6;13)
Bahia	236 (219;265)	4.2 (3.9;4.8)	719 (675;798)	4.3 (4.1;4.8)	2.3 (-6.1;10.2)
Brazil	2649 (2428;2836)	4.7 (4.4;5.1)	10059 (9390;10698)	4.8 (4.5;5.2)	2.5 (-2.9;7.3)
Ceará	128 (117;149)	3.5 (3.2;4.1)	404 (380;443)	3.9 (3.6;4.3)	10.9 (-1.7;21.9)
Distrito Federal	13 (12;14)	6.4 (6.1;7)	97 (88;108)	7.6 (6.8;8.4)	19.8 (5.5;32.9)
Espírito Santo	41 (37;44)	6.2 (5.7;6.7)	169 (157;188)	4.5 (4.1;5)	-28.3 (-33.7;-21)
Goiás	43 (38;46)	4.9 (4.5;5.2)	226 (206;246)	4.3 (3.9;4.6)	-13.7 (-20.1;-5.6)
Maranhão	85 (55;101)	4.9 (3;5.8)	305 (219;342)	5.1 (3.6;5.7)	4.1 (-6.7;26.3)
Mato Grosso	18 (16;21)	4.1 (3.8;5.1)	99 (92;111)	4.4 (4.1;4.9)	5.6 (-8.3;16.1)
Mato Grosso do Sul	24 (22;26)	5.1 (4.7;5.5)	110 (103;122)	4.8 (4.5;5.3)	-6.6 (-13.3;2.2)
Minas Gerais	279 (253;294)	5.2 (4.7;5.4)	1144 (1058;1223)	4.6 (4.3;4.9)	-11.5 (-17.2;-4.6)
Pará	63 (59;71)	4.5 (4.2;5)	258 (232;279)	4.6 (4.1;5)	2.5 (-8.3;11.9)
Paraíba	74 (69;85)	3.8 (3.5;4.3)	196 (179;222)	3.9 (3.6;4.5)	4.1 (-8.4;16.3)
Paraná	123 (115;137)	5.4 (5.1;5.9)	516 (487;575)	4.9 (4.6;5.5)	-8.3 (-14.2;-2)
Pernambuco	135 (127;151)	4.7 (4.5;5.3)	402 (377;446)	4.3 (4;4.8)	-9 (-15.1;-2.3)
Piaui	43 (39;51)	4.1 (3.7;5)	138 (129;157)	3.8 (3.5;4.3)	-9.1 (-20.7;0.5)
Rio de Janeiro	290 (266;306)	5.3 (4.9;5.6)	1052 (972;1124)	5.2 (4.8;5.6)	-0.2 (-6.7;6.5)
Rio Grande do Norte	60 (54;67)	4.1 (3.7;4.6)	169 (158;188)	4.2 (3.9;4.6)	1.5 (-8.4;12.4)
Rio Grande do Sul	183 (168;195)	4.9 (4.5;5.2)	698 (655;761)	4.9 (4.6;5.3)	0.5 (-6.6;7.6)
Rondônia	5 (5;6)	4.5 (4.2;5)	45 (41;52)	4.3 (3.8;4.9)	-5.4 (-16.1;6.2)
Roraima	1 (1;2)	7.7 (7;9.2)	11 (10;13)	6.6 (5.8;7.4)	-14.5 (-30.2;-1.2)
São Paulo	637 (571;670)	5.8 (5.2;6.1)	2605 (2372;2755)	5.8 (5.3;6.2)	1.2 (-6.3;8.4)
Santa Catarina	69 (64;74)	5.2 (4.8;5.6)	312 (293;340)	5.1 (4.8;5.6)	-1.3 (-8.7;6.5)
Sergipe	27 (25;31)	3.6 (3.4;4.1)	81 (75;87)	4 (3.7;4.4)	11.4 (-0.8;21.6)
Tocantins	10 (9;12)	7.1 (6.4;8.3)	55 (50;60)	4.4 (4;4.8)	-37.4 (-46.5;-30.2)

*Source: Global Burden of Disease Study 2017, Institute for Health Metrics and Evaluation .^285^*

**Table 6-3:** – Number of deaths and age-standardized mortality rate due to atrial fibrillation and flutter, per 100 000 inhabitants, for males, in 1990 and 2017, with percent change, in Brazil and Brazilian Federative Units

	1990		2017		
	Number of deaths (95% UI)	Mortality rate (95% UI)	Number of deaths (95% UI)	Mortality rate (95% UI)	Percent change (95% UI)
Acre	2.4 (2;2.9)	5.2 (4.5;6.3)	9.9 (7.4;11)	4.3 (3.2;4.7)	-18.5 (-36;-6.3)
Alagoas	16.8 (14.9;21.8)	3.9 (3.5;5.1)	43.9 (36;51)	3.6 (3;4.2)	-6.8 (-25.1;7.4)
Amapá	1.4 (1.2;1.7)	5.3 (4.7;6.9)	8 (5.9;8.9)	5.7 (4.2;6.3)	7.2 (-22.4;24.4)
Amazonas	9 (8;12.5)	4.1 (3.7;5.9)	37.1 (31.5;44.3)	3.8 (3.2;4.5)	-8.9 (-31.4;6.1)
Bahia	106.6 (93.5;133.7)	4.5 (3.9;5.6)	300.3 (246.4;345.5)	4.5 (3.7;5.2)	2 (-12.8;14.6)
Brazil	1134.1 (952.8;1309.1)	4.7 (4.1;5.6)	3773.7 (3076.2;4312)	4.6 (3.8;5.2)	-3.5 (-12.9;1.8)
Ceará	60.8 (52.3;85.1)	3.7 (3.2;5.2)	150.3 (124.7;172.3)	3.6 (3;4.1)	-3.7 (-27.5;14.2)
Distrito Federal	5.1 (4.4;6.3)	5.8 (5;6.9)	33.1 (27.6;44.6)	6.5 (5.5;8.3)	12.6 (-5.3;28.7)
Espírito Santo	18.7 (15.5;21.5)	6 (5;7.1)	68.3 (59.6;85.4)	4.5 (3.9;5.6)	-25.5 (-35.5;-12.6)
Goiás	19.6 (15.7;22.2)	3.6 (2.8;4)	104.4 (86.7;121.5)	4.3 (3.6;5.1)	20.7 (5.6;36.8)
Maranhão	37.7 (28.4;47.6)	6 (4.1;7.9)	152.5 (121.6;180.5)	6.1 (4.8;7.2)	0.5 (-17.7;21.6)
Mato Grosso	9.7 (8.6;13.3)	4.5 (3.9;6.5)	45.7 (39.9;56.6)	4 (3.5;4.9)	-12.1 (-31.4;2)
Mato Grosso do Sul	12 (10;13.7)	4.8 (3.9;5.4)	46.7 (37.9;53)	4.5 (3.7;5.2)	-4.6 (-15;4.9)
Minas Gerais	117.9 (91.5;129.6)	4.9 (3.8;5.3)	448.6 (360.9;504.4)	4.4 (3.6;5)	-8.8 (-18.3;5.3)
Pará	27.4 (24.5;34.2)	4.4 (3.9;5.6)	118.4 (98.2;138.8)	4.7 (3.8;5.5)	6.5 (-11.5;21.6)
Paraíba	34.5 (30.2;43.6)	3.7 (3.2;4.6)	73.6 (58.5;86.4)	3.7 (3;4.4)	1.3 (-16.8;20)
Paraná	56.9 (48.1;67.9)	5.1 (4.3;6.1)	207 (179.8;248.3)	4.7 (4.1;5.6)	-7.7 (-19.6;2.2)
Pernambuco	60.6 (51.5;72.6)	5 (4.2;5.9)	146.5 (118.1;165.8)	4 (3.2;4.5)	-19.1 (-30.3;-10.8)
Piauí	21.7 (18.5;31.3)	5.2 (4.4;7.7)	51.5 (41.7;59.5)	3.3 (2.7;3.8)	-36.2 (-55.3;-22.8)
Rio de Janeiro	111.5 (86.2;124.2)	5.4 (4.3;6.1)	348.2 (286.2;400.2)	4.9 (4.1;5.7)	-9.5 (-20.4;2.3)
Rio Grande do Norte	28.1 (25.1;36.5)	4.4 (3.9;5.6)	64.6 (52.2;74.5)	4 (3.2;4.6)	-7.7 (-27.1;6.6)
Rio Grande do Sul	72.6 (59.1;83.3)	5.2 (4.2;6)	244.4 (205.8;288.7)	4.8 (4;5.6)	-8 (-20.4;2.1)
Rondônia	3.1 (2.7;3.7)	4.9 (4.1;5.7)	21.6 (18;25.5)	4 (3.3;4.7)	-18.7 (-32.2;-4.5)
Roraima	0.7 (0.6;0.9)	7.4 (6.4;10.2)	5.2 (3.5;6.1)	5.6 (3.7;6.6)	-24.1 (-55.7;-2.4)
Santa Catarina	29 (23.2;33.3)	4.9 (3.9;5.6)	111.5 (95.9;136.1)	4.6 (3.9;5.5)	-6.4 (-21.3;9.3)
São Paulo	253.3 (189.4;278.3)	5.4 (4.1;6)	877.3 (668.2;969.6)	5.2 (4;5.8)	-4.1 (-16.6;9.7)
Sergipe	12.1 (10.9;15.6)	3.8 (3.4;4.8)	29.6 (24;33.9)	3.7 (3;4.3)	-1.3 (-19.8;13.2)
Tocantins	5 (4.3;7)	6.7 (5.6;10.1)	25.5 (19.7;28.6)	4.1 (3.1;4.5)	-39.9 (-59.5;-25)

*Source: Global Burden of Disease Study 2017, Institute for Health Metrics and Evaluation.^285^*

**Table 6-4:** – Number of deaths and age-standardized mortality rate due to atrial fibrillation and flutter, per 100 000 inhabitants, for females, in 1990 and 2017, with percent change, in Brazil and Brazilian Federative Units

	1990		2017		
	Number of deaths (95% UI)	Mortality rate (95% UI)	Number of deaths (95% UI)	Mortality rate (95% UI)	Percent change (95% UI)
Acre	2.1 (1.9;2.2)	4.7 (4.3;5)	12.1 (11;13.8)	4.7 (4.3;5.4)	0.1 (-12.3;20.5)
Alagoas	18.6 (17.2;20.4)	3.3 (3;3.6)	74.9 (68.7;87)	4.4 (4;5.1)	33.7 (18.1;51.9)
Amapá	1.8 (1.6;1.9)	6.2 (5.7;6.6)	9.8 (8.9;10.9)	5 (4.6;5.6)	-19.1 (-28.5;-6.7)
Amazonas	9.5 (8.8;10.3)	3.8 (3.5;4)	50.3 (46.4;55.1)	4.2 (3.9;4.7)	13 (1.5;26.5)
Bahia	129.5 (119;141.4)	4.1 (3.8;4.5)	419 (386.8;470)	4.1 (3.8;4.6)	1 (-10.1;14.6)
Brazil	1515 (1453.7;1558)	4.7 (4.5;4.9)	6285.3 (6014.2;6615.7)	5 (4.8;5.2)	5.4 (0;12.3)
Ceará	67.3 (55.6;76.4)	3.4 (2.8;3.8)	253.8 (235.8;280.5)	4.1 (3.8;4.5)	21.2 (2.5;55.8)
Distrito Federal	7.9 (7.4;8.5)	6.8 (6.3;7.5)	64.3 (55.9;72)	8 (6.9;9)	17.8 (-4.1;36.2)
Espírito Santo	22.2 (21.1;23.4)	6.3 (5.9;6.6)	100.3 (91.8;109.2)	4.4 (4;4.8)	-29.8 (-36.4;-22.7)
Goiás	23.4 (21.9;24.8)	6.4 (6;6.8)	121.8 (112.8;132.6)	4.2 (3.9;4.6)	-34.1 (-39.9;-27.8)
Maranhão	47.5 (19.2;62)	4.6 (1.8;6)	152.1 (84.2;183)	4.5 (2.5;5.4)	-2.7 (-16.2;44.7)
Mato Grosso	7.9 (7.1;8.8)	3.8 (3.4;4.2)	53 (48.6;58.4)	4.7 (4.3;5.2)	23.7 (7;43.6)
Mato Grosso do Sul	12.3 (11.7;13)	5.4 (5.1;5.7)	63.5 (58.3;71.8)	5 (4.5;5.6)	-8.2 (-16.6;3.4)
Minas Gerais	161 (153.7;169.7)	5.3 (5.1;5.6)	695.6 (644.5;746.7)	4.7 (4.3;5)	-12.6 (-19.7;-5.3)
Pará	35.7 (33;38.6)	4.5 (4.2;4.8)	139.2 (126.4;151.5)	4.5 (4.1;4.9)	-0.5 (-10.9;10.8)
Paraíba	40 (36.6;44)	3.9 (3.5;4.2)	122.3 (108.1;146.1)	4.1 (3.6;4.8)	5.2 (-10.7;27.9)
Paraná	66 (62.6;70.3)	5.6 (5.3;6)	309.3 (287.2;338.3)	5 (4.7;5.5)	-10 (-16.8;-2)
Pernambuco	74.4 (69.4;82.4)	4.6 (4.3;5)	255.8 (234.3;293.1)	4.5 (4.1;5.1)	-2.1 (-10.7;9)
Piauí	21.2 (17.8;24.3)	3.5 (3;4.1)	86.9 (80.1;99)	4 (3.7;4.6)	14.2 (-2.7;46.6)
Rio de Janeiro	178.9 (169.9;190.3)	5.2 (4.9;5.5)	703.5 (652;753.2)	5.3 (5;5.7)	3.5 (-4.7;12.5)
Rio Grande do Norte	31.7 (27.6;35.2)	4 (3.4;4.4)	104.6 (95.4;119)	4.2 (3.8;4.8)	7.2 (-7.4;35.9)
Rio Grande do Sul	110 (104.6;115.1)	4.7 (4.5;5)	454 (421.1;488)	4.9 (4.6;5.3)	3.6 (-4.2;12)
Rondônia	2 (1.8;2.2)	4.1 (3.8;4.5)	23.7 (20.5;28.3)	4.5 (3.9;5.4)	9.7 (-6.2;28.3)
Roraima	0.5 (0.5;0.6)	7.8 (7.1;8.7)	6.1 (5.1;7.7)	7.3 (6.1;9.2)	-6.5 (-26.1;18.5)
Santa Catarina	39.8 (37.7;42.4)	5.4 (5.1;5.7)	200.6 (185.2;217.9)	5.4 (5;5.9)	0.7 (-8;10.2)
São Paulo	384 (366;404.7)	5.9 (5.6;6.2)	1728 (1597.3;1849.2)	6.1 (5.7;6.6)	3.3 (-4.6;11.7)
Sergipe	14.7 (13.6;16)	3.5 (3.2;3.8)	51 (46.9;55.6)	4.2 (3.9;4.6)	20 (5.5;34.7)
Tocantins	5 (4.2;5.6)	7.3 (6;8.4)	29.9 (27.3;33.6)	4.8 (4.4;5.4)	-34.3 (-44.6;-13.8)

*Source: Global Burden of Disease Study 2017, Institute for Health Metrics and Evaluation.^285^*

**Table 6-5:** – Number of DALYs and age-standardized DALY rates due to atrial fibrillation and flutter, per 100 000 inhabitants, in 1990 and 2017, with percent change, in Brazil and Brazilian Federative Units

	1990		2017		
	Number of DALYs (95% UI)	DALY Rate (95% UI)	Number of DALYs (95% UI)	DALY Rate (95% UI)	Percent change (95% UI)
Acre	140.3 (113.7;174.5)	95.2 (79;115.3)	542.9 (445.2;660.8)	99.2 (82.4;119.4)	4.2 (-2.5;9.6)
Alagoas	909.3 (730.5;1123.5)	72.4 (59.1;88.5)	2456 (2031.2;2952.7)	82.7 (68.8;99.8)	14.3 (5.6;21.9)
Amapá	93.7 (76;114.6)	108.7 (91.2;129.2)	499.4 (411.6;598.4)	114.8 (96.3;135.5)	5.6 (-0.8;10.9)
Amazonas	560.7 (445.6;698.8)	80.3 (65.3;98.1)	2138.5 (1735.6;2644.7)	86 (70.9;104.7)	7 (-2.3;13.8)
Bahia	5285.8 (4366.4;6397.1)	81.3 (67.7;97.7)	13791.8 (11451.4;16610.3)	87.7 (72.8;105.1)	7.9 (1.8;14.3)
Brazil	79208.5 (64514;96525.8)	99 (82.3;118.9)	226809.7 (187976.8;272166.3)	103.6 (86.3;123.8)	4.6 (1.7;7.2)
Ceará	3014.4 (2422;3741.1)	75.8 (61.3;93.5)	8054.1 (6619.6;9756.6)	81.9 (67.2;99.4)	8.1 (0.8;15.1)
Distrito Federal	543.8 (441.9;662.7)	115.2 (98.8;135.3)	2469.3 (2043.2;2968.8)	121.7 (104.3;143.6)	5.6 (-0.4;12.4)
Espírito Santo	1099.3 (895.3;1344.2)	92 (77.3;109.3)	3570.3 (2938.2;4327.6)	88 (73;106.3)	-4.3 (-9.7;2)
Goiás	1394.8 (1125.9;1736.9)	88.5 (74.3;106.4)	5412.9 (4410.7;6618)	87.9 (72.8;106.9)	-0.7 (-6;4.2)
Maranhão	1889.2 (1401.3;2435)	82.3 (59.9;105.9)	5041.4 (3929.4;6224.5)	82.1 (63.9;101)	-0.3 (-8.1;9.3)
Mato Grosso	598.8 (479.5;750)	88.1 (72.5;107.8)	2612.4 (2125.1;3202.6)	93.2 (76.9;113.4)	5.7 (-1.8;12.3)
Mato Grosso do Sul	826.2 (672.8;1002.9)	106.5 (89.4;126.4)	2822.2 (2306.6;3401.7)	107.2 (88.5;128.2)	0.7 (-4.2;5.9)
Minas Gerais	9413 (7638.3;11547.3)	108.3 (90.1;129.8)	27059.3 (22340.6;32657.7)	108.2 (89.5;130.4)	-0.1 (-5;4.7)
Pará	1539.4 (1262.2;1889.2)	81.5 (67.9;98.9)	5393.3 (4478.1;6529.9)	87.8 (73.5;105.3)	7.7 (-0.5;15.3)
Paraíba	1821.5 (1468.3;2223.4)	80.9 (65.7;98.4)	4007.5 (3263.1;4821.6)	86.5 (70.3;104.3)	6.9 (-0.9;14.3)
Paraná	3979.9 (3220;4841.8)	99.3 (83.5;118.5)	12001.8 (9814.5;14534.6)	99.9 (82.5;120)	0.6 (-4.5;5.2)
Pernambuco	3220.7 (2629.5;3992.3)	80.7 (67.7;97.7)	7977.1 (6643;9724)	83.5 (69.8;101.6)	3.5 (-2.1;8.8)
Piauí	1028.8 (830.3;1272.4)	78.1 (63.8;95.4)	2789.9 (2291.5;3412.7)	77.8 (64;95)	-0.4 (-9.6;6.5)
Rio de Janeiro	9520.4 (7787.9;11658.2)	111.4 (93.3;133.4)	24316.8 (20202.2;29195.3)	115 (95.9;137.6)	3.2 (-1.1;7.7)
Rio Grande do Norte	1263.4 (1031.4;1535.4)	80.1 (65.7;96.9)	3165.6 (2620.9;3796.6)	84.7 (70.1;101.5)	5.8 (-1;13)
Rio Grande do Sul	5078.4 (4134.3;6186.3)	89.5 (74.8;107.2)	13838.5 (11505.8;16723.4)	93.9 (78.3;113)	5 (-0.4;10.1)
Rondônia	253.4 (200.9;317.6)	90.3 (75.1;109.6)	1179 (948.7;1453.8)	89.7 (73.1;108.8)	-0.7 (-9.2;6.7)
Roraima	56.1 (44.6;69.3)	126.8 (106.6;149.8)	348.9 (280.1;430.2)	121.6 (100.7;145.4)	-4.1 (-14.3;4.4)
Santa Catarina	2181.6 (1779.3;2657.9)	102 (85.4;121.3)	7392.7 (6024.1;8973)	103.4 (85.4;123.9)	1.3 (-4.1;6.7)
São Paulo	22477.6 (18132.5;27717.9)	126.9 (105.3;152.6)	64766.7 (53494.2;78258.7)	129.7 (107.8;155.7)	2.2 (-1.9;6.8)
Sergipe	669 (542.7;814.8)	81.1 (66.1;98.5)	1828.2 (1484.9;2230)	88.9 (72.8;108.1)	9.6 (2;16.3)
Tocantins	349.1 (280.8;431.7)	109.5 (92.1;130.9)	1333.2 (1097.2;1609.2)	100.5 (83.2;120.9)	-8.2 (-17.2;-1.2)

*Source: Global Burden of Disease Study 2017, Institute for Health Metrics and Evaluation.^285^*

**Table 6-6:** – Number of DALYs and age-standardized DALY rates due to atrial fibrillation and flutter, per 100 000 inhabitants, for males, in 1990 and 2017, with percent change, in Brazil and Brazilian Federative Units

	1990		2017		Percent change (95% UI)
	Number of DALYs (95% UI)	DALY Rate (95% UI)	Number of DALYs (95% UI)	DALY Rate (95% UI)	
Acre	82.4 (64.8;104.4)	103.4 (83.4;127.4)	285.5 (225.7;358.6)	105.6 (83.6;130.8)	2.1 (-9.8;11)
Alagoas	499.5 (389.5;638.7)	85 (67.3;107.9)	1190.5 (947.6;1474.1)	90.7 (72.7;112.3)	6.7 (-5.5;16.7)
Amapá	51.1 (40;63.7)	118 (95.2;144.5)	271.4 (214;333.8)	130.5 (104.5;157)	10.6 (-3;20.7)
Amazonas	329.6 (252.8;421.6)	94 (74.1;118)	1188.9 (930.8;1511.3)	97.5 (77.3;121.8)	3.7 (-9.4;13)
Bahia	2825.4 (2259;3508.4)	93.6 (75.8;114.9)	7090.6 (5714.9;8706.2)	103.8 (83.9;126.9)	10.8 (0.8;20.6)
Brazil	41514.9 (32715.2;51721.2)	111.1 (89.5;136.9)	110643.1 (88439.8;136579.6)	115.1 (92.9;141.4)	3.6 (-1.4;6.8)
Ceará	1685.5 (1306.6;2141)	90 (70.3;113.6)	4062.6 (3213.5;5079.7)	94.3 (74.8;117.5)	4.8 (-9.7;15.1)
Distrito Federal	279.4 (219.3;350.7)	121.6 (100;148)	1168.9 (907.8;1460.2)	129.5 (104.1;159.2)	6.5 (-3.6;16.9)
Espírito Santo	599.5 (468.5;752.8)	102.1 (82.7;126.1)	1800.5 (1424.3;2230.4)	99.9 (79.9;123)	-2.1 (-9.8;6.8)
Goiás	751.4 (581.6;960.7)	85.2 (67.9;107.5)	2773.6 (2210.3;3516.4)	95.5 (77.1;119.9)	12.1 (3.3;20.3)
Maranhão	1002.8 (794.7;1292)	97.2 (76.4;124.3)	2672.9 (2147.4;3307.8)	95.8 (77.1;117.9)	-1.4 (-12.1;9.3)
Mato Grosso	361.1 (280.9;464.2)	97.1 (77.2;122.4)	1416.9 (1121.4;1780.2)	98 (78;122.3)	0.9 (-10.9;10.3)
Mato Grosso do Sul	481.8 (382.6;600.1)	116.5 (94.2;143.5)	1485.7 (1183.7;1835.7)	118.9 (95.7;145.8)	2 (-5;8.3)
Minas Gerais	5075.7 (3941.5;6388.2)	123.2 (98.4;152.2)	13931.2 (11083.4;17144.6)	124.9 (100.2;152.9)	1.4 (-5.6;8.7)
Pará	848 (666.8;1072.3)	92 (73.9;114.8)	3030.6 (2434.6;3752.2)	102.1 (83.5;125.9)	11 (-0.9;21.2)
Paraíba	959.6 (751.8;1199.7)	89.6 (70.4;112.2)	1908.7 (1508.1;2391.4)	96.4 (76.2;120.1)	7.6 (-3.9;19.3)
Paraná	2155.1 (1663;2723.3)	106.1 (85.2;131)	5923.6 (4690.3;7392.9)	109.3 (87.5;134.3)	3 (-4.7;10.2)
Pernambuco	1666.7 (1313.2;2134)	92.1 (74.4;115.9)	3745.8 (2945.1;4730.6)	92.8 (73.4;117.1)	0.8 (-8.9;8.4)
Piauí	576.9 (446.6;737.6)	95.2 (74.8;122.4)	1384.7 (1098.5;1744.7)	85.8 (68.1;107.6)	-9.9 (-26.6;0.8)
Rio de Janeiro	4817.5 (3787.4;6124)	130.7 (106;161.9)	11413.5 (9010.4;14162.3)	132.1 (105.6;162.3)	1.1 (-6.1;7.9)
Rio Grande do Norte	693 (547.8;864.6)	93.1 (74.1;115.7)	1566.9 (1250.7;1936.6)	97.1 (77.6;119.6)	4.3 (-9.9;14.5)
Rio Grande do Sul	2490.2 (1952.9;3132)	100.7 (81.3;123.8)	6341.6 (5040.4;7933.5)	103.5 (83.6;128.3)	2.8 (-6;10)
Rondônia	162 (126.2;207.2)	99.7 (81.4;123.1)	641.9 (500.4;812.1)	94.9 (75.2;118.3)	-4.9 (-14.9;5)
Roraima	37.3 (29;46.7)	140.1 (113.5;170.8)	206.6 (161.2;261.9)	132.4 (105.1;163.7)	-5.5 (-24.5;7.6)
Santa Catarina	1083.7 (847.3;1362.7)	106.2 (85.8;130.2)	3431.7 (2674.2;4328.4)	107 (85.2;133.9)	0.8 (-8.3;9.9)
São Paulo	11426.4 (8857.6;14374.4)	139.5 (111.1;173.2)	30035.6 (23857;37379.4)	140.2 (112.5;172.2)	0.5 (-6.5;7.5)
Sergipe	360.2 (280.2;450.3)	95.4 (74.6;119)	913.8 (721.5;1145.2)	101.8 (81.3;126.9)	6.7 (-4.6;16.1)
Tocantins	213.1 (162.9;269.7)	120.8 (96.4;150)	758.9 (602.2;940.5)	111.6 (89.1;137.6)	-7.6 (-24.8;3.3)

*Source: Global Burden of Disease Study 2017, Institute for Health Metrics and Evaluation.^285^*

**Table 6-7:** – Number of DALYs and age-standardized DALY rates due to atrial fibrillation and flutter, per 100 000 inhabitants, for females, in 1990 and 2017, with percent change, in Brazil and Brazilian Federative Units

	1990		2017		Percent change (95% UI)
	Number of DALYs (95% UI)	DALY Rate (95% UI)	Number of DALYs (95% UI)	DALY Rate (95% UI)	
Acre	57.9 (47.4;70.5)	86.1 (72.7;102.9)	257.4 (215.1;306.5)	92.9 (77.9;110.2)	7.9 (-0.3;18.8)
Alagoas	409.8 (338.1;497)	61.8 (51.4;75)	1265.4 (1065.7;1512.4)	76 (64.2;90.7)	23 (11;37.8)
Amapá	42.6 (35.6;51.1)	99.4 (85;116.6)	227.9 (189.3;270.1)	101.1 (84.9;118.6)	1.6 (-6.7;11.1)
Amazonas	231.1 (189.1;286.3)	67.1 (56.5;81.1)	949.6 (793.8;1139.7)	74.8 (62.7;88.8)	11.4 (2.8;20.8)
Bahia	2460.4 (2058.7;2986.8)	70.7 (59.5;85.1)	6701.3 (5667.8;8010.2)	74.7 (62.9;89.5)	5.6 (-2.8;15)
Brazil	37693.5 (31770.2;45054.9)	88.6 (76.2;103.9)	116166.6 (98895.8;135791.7)	93.7 (79.8;109.5)	5.8 (2.4;9.7)
Ceará	1329 (1065.5;1653.3)	63.3 (51;78.8)	3991.4 (3369.5;4733.9)	71.4 (60.1;85.2)	12.8 (0.7;31.8)
Distrito Federal	264.4 (221.2;315.2)	109.1 (94.7;125)	1300.4 (1089.6;1554.4)	113.9 (96.7;132.4)	4.4 (-7.5;15.1)
Espírito Santo	499.9 (418.4;598.1)	82.4 (71.3;96.2)	1769.9 (1488;2115)	78 (65.7;93)	-5.3 (-11.9;1.5)
Goiás	643.3 (529.2;783.9)	92.3 (80.3;107)	2639.3 (2200.5;3167.7)	81.1 (68.2;96.8)	-12.2 (-18.5;-5.6)
Maranhão	886.4 (483.4;1176.4)	72.3 (38.6;96)	2368.5 (1549.5;2987.1)	71.1 (46.4;89.5)	-1.8 (-12.9;25)
Mato Grosso	237.7 (192.1;291.2)	78.2 (64.8;94.1)	1195.4 (994.5;1433.5)	88.1 (74.1;103.9)	12.7 (3.3;24)
Mato Grosso do Sul	344.4 (290.2;410.1)	95.3 (82.6;111.1)	1336.5 (1121.2;1582.5)	96.4 (81.4;113.9)	1.2 (-5.8;9.8)
Minas Gerais	4337.2 (3603.5;5206.8)	95 (81.1;111.6)	13128.1 (11052.7;15567.4)	93.7 (78.7;111.3)	-1.3 (-7.3;4.6)
Pará	691.4 (579.5;847.2)	71.2 (60.2;85.5)	2362.7 (2004;2820.5)	74.2 (63.4;87.9)	4.2 (-4.6;13.7)
Paraíba	861.9 (711.9;1040.7)	73.2 (61.1;87.8)	2098.8 (1736.5;2506.6)	78.4 (64.5;94)	7.1 (-4;21.5)
Paraná	1824.7 (1515.5;2188.5)	92.4 (79.6;107.7)	6078.2 (5072;7195.1)	91.6 (76.9;107.9)	-0.9 (-6.8;5.3)
Pernambuco	1554 (1305.2;1877.1)	71.4 (61;84.8)	4231.3 (3587.3;5077.2)	76.1 (64.6;91)	6.6 (-0.4;14.6)
Piauí	451.9 (362.3;565.1)	64.6 (52.1;80.5)	1405.3 (1184.9;1674.4)	70.6 (59.4;84.4)	9.2 (-2.9;28)
Rio de Janeiro	4703 (3930;5630.4)	97 (82.5;114.7)	12903.3 (10976.9;15227.6)	101.6 (86.2;120)	4.7 (-1.2;11)
Rio Grande do Norte	570.4 (472.9;691.2)	68.7 (57;82.9)	1598.7 (1346;1893.9)	74.5 (62.2;88.8)	8.4 (-3.2;27.5)
Rio Grande do Sul	2588.2 (2156.8;3097.6)	81.1 (69.1;95.9)	7496.9 (6386.8;8904.7)	86 (72.8;102.1)	5.9 (0;12.8)
Rondônia	91.4 (72.4;113.5)	79.2 (66.7;94.3)	537.1 (434.2;655.1)	84.2 (68.9;101.2)	6.3 (-4.1;18.8)
Roraima	18.8 (15.1;22.9)	108.9 (92.8;126.8)	142.3 (114.4;173.6)	108.9 (89.6;130.4)	0 (-14.6;17.9)
Santa Catarina	1097.9 (912.9;1328.2)	97.8 (83.5;116.1)	3961 (3331.6;4682.9)	99.3 (83.9;116.5)	1.5 (-4.6;8.4)
São Paulo	11051.2 (9167;13335.7)	115.9 (98;137.3)	34731.2 (29277.5;41069.7)	120.4 (101.6;142.3)	3.9 (-1.4;9.9)
Sergipe	308.8 (258.3;371.5)	69 (58;82.6)	914.3 (770.8;1097.2)	78.3 (65.9;93.7)	13.3 (4.5;23.3)
Tocantins	136 (110.1;166.5)	96.4 (80.4;114.7)	574.3 (479.9;679.9)	89 (74.9;105.6)	-7.7 (-18.2;8.4)

*Source: Global Burden of Disease Study 2017, Institute for Health Metrics and Evaluation.^285^*

**Chart 6-1:** Data derived from Global Burden of Disease Study 2017 (GBD 2017).^285^

**Chart 6-2:** Data derived from Global Burden of Disease Study 2017 (GBD 2017).^285^

**Chart 6-3:** Data derived from Global Burden of Disease Study 2017 (GBD 2017).^285^
